# Diastereoselective Substitution Reactions of Acyclic Acetals Controlled by Remote Participation of an Acyloxy Group

**DOI:** 10.1021/acs.orglett.4c03766

**Published:** 2024-12-02

**Authors:** Khoi B. Luu, Amanda Ramdular, Eli Finkelstein, Alexander G. Shtukenberg, K. A. Woerpel

**Affiliations:** Department of Chemistry, New York University, 100 Washington Square East, New York, New York 10003, United States

## Abstract

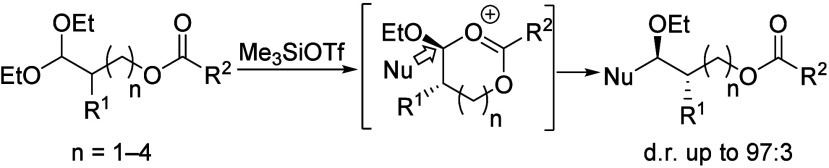

A remote carbonyl group up to six atoms away from the acetal group can induce 1,2-asymmetric induction in nucleophilic substitution reactions of acyclic acetals. Isolation of a cyclic carbonate under Lewis acidic conditions and computational studies suggested that the remote carbonyl group participated through the formation of a cyclic dioxocarbenium ion intermediate. The stereochemical outcomes depended on the size of the alkyl substituent and that of the nucleophile employed.

Remote participation is a common approach for controlling stereoselectivity in nucleophilic substitution reactions of acetals.^1−4^ The participation of carbonyl groups, in particular, has been studied extensively in carbohydrates^5−7^ but not in acyclic acetals.^8^ In this paper, we demonstrate that the remote participation of a carbonyl group up to six atoms away from the electrophilic carbon atom of an acyclic acetal can induce 1,2-asymmetric induction in substitution reactions (eq 1).
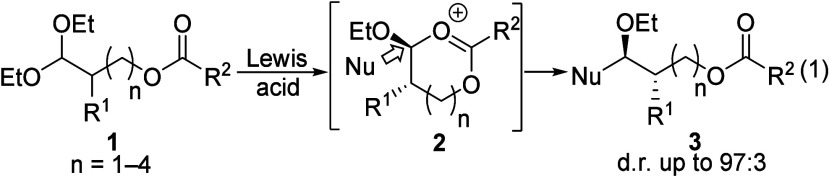
1

The isolation of a cyclic carbonate from a reaction of γ-acyloxy acetal **4** provided evidence for remote participation (eq 2). In the presence of a Lewis acid, acetal **4** was converted into cyclic carbonate **6** as a single diastereomer. Carbonate **6** was likely a product of the participation of the carbonyl group via seven-membered-ring dioxocarbenium ion **5** followed by loss of a *tert*-butyl group.^9,10^ The stereochemical configuration of carbonate **6**, which could not be established unambiguously due to the instability of this carbonate and its rapid decarboxylation, was tentatively assigned on the basis of analyses of the *J* coupling constants in its ^1^H nuclear magnetic resonance (NMR) spectrum.


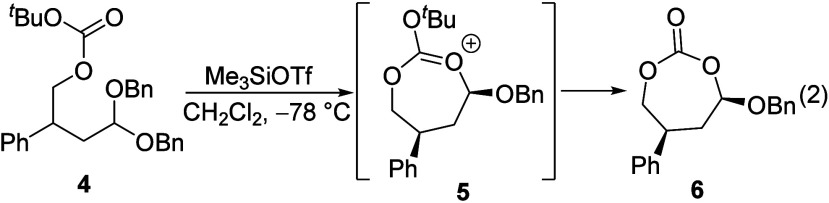
2

To test whether 1,2-asymmetric induction via a six-membered-ring dioxocarbenium ion could be achieved, substitution reactions of β-pivaloyl acetal **7a** were performed. In general, reactions of more sterically hindered or more reactive nucleophiles, as reflected by their higher N number,^11^ were more stereoselective. Allyltrimethylsilane reacted with acetal **7a** to give a 53:47 mixture of diastereomers (Table 1, entry 1), whereas the reaction of the stronger nucleophile allyltributylstannane gave a 75:25 *syn*:*anti* product mixture (entry 5). The *syn* selectivity increased further with more reactive silyl ketene acetal **14** and trimethylsilyl cyanide^12^ (entries 10 and 11, respectively). With the most sterically hindered nucleophile, **13**, however, high *anti* selectivity was observed instead. The relative stereochemistry of the products was assigned using a combination of X-ray crystallography, chemical correlations, and calculations of NMR spectra,^13,14^ which are detailed in the Supporting Information.

**Table 1:**
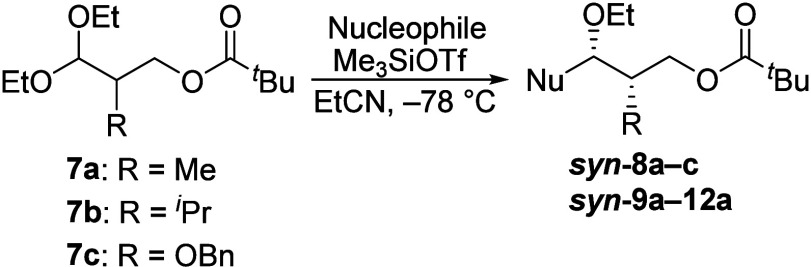

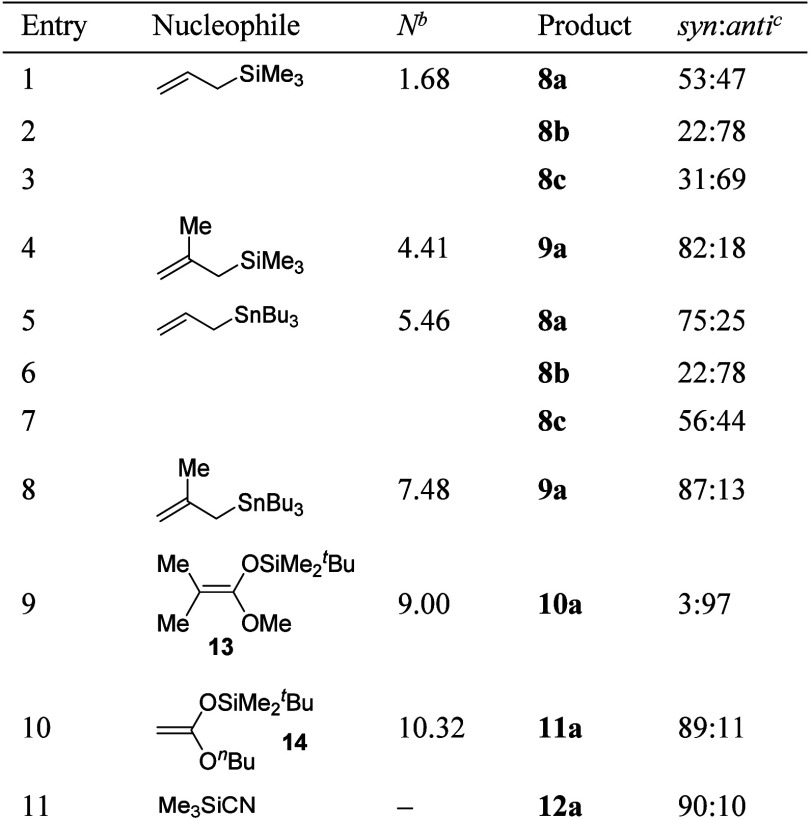
Nucleophilic Substitution Reactions of β-Pivaloyl Acetals Bearing an α-Substituent^a^

a Isolated yields of 58–86% on a preparative scale.

b Nucleophilicity parameters; a higher *N* indicates a higher nucleophilicity.^11^

c Diastereomeric ratios were determined from ^1^H and ^13^C NMR spectroscopic analysis of the crude reaction mixtures.^17^

The effect of the size and the electronic nature of the α-substituent on the diastereoselectivity was studied using acetals **7b** and **7c**. In contrast to the *syn*-selective reactions of methyl-substituted acetal **7a**, reactions of isopropyl-substituted acetal **7b** were *anti*-selective (entries 2 and 6). Benzyloxy-substituted acetal **7c** also reacted with allyltrimethylsilane with low *anti* selectivity (entry 3; the relative stereochemistry was tentatively assigned on the basis of the outcomes of similar reactions of alkoxy-substituted acetals^15,16^).

Reactions of weak nucleophiles likely occurred through an S_N_1 substitution pathway that involves reactive oxocarbenium ion **15** (Scheme 1). The low stereoselectivity of the reaction of acetal **7a** could be explained by the small difference in size between the methyl substituent and the longer side chain. The more selective reaction of isopropyl-substituted acetal **7b** is consistent with reported reactions of acyclic oxocarbenium ions, which were more selective with larger α-substituents.^18,19^

**Scheme 1:**
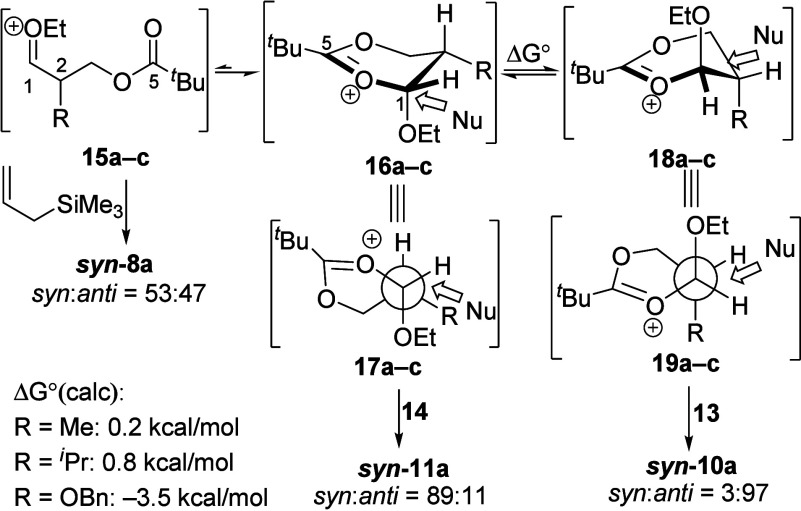
Substitution Pathways of the Oxocarbenium Ion and Dioxocarbenium Ion Intermediates Newman projections viewed down the C1–C2 bond.

Stronger nucleophiles could react through S_N_2 substitution pathways with six-membered-ring dioxocarbenium ions. The *cis* conformers **16a** and **16b** were calculated to be the lowest-energy conformers, in which the ethoxy group is positioned in an axial orientation and the α-alkyl substituent is positioned in an equatorial orientation to minimize steric interactions.^20^ Substitutions of the *cis* conformers resulted in the observed *syn* selectivities. With a sterically hindered nucleophile or α-substituent, however, reactions through conformer **16** resulted in significant eclipsing interactions between the nucleophile and the α-alkyl substituent. Additions to *trans* conformer **18**, which would avoid such eclipsing interactions, became more favorable, leading to *anti* selectivity. The dioxocarbenium ion of alkoxy-substituted acetal **7c**, on the contrary, prefers to be in *trans* conformer **18c**, likely due to the electronic stabilization provided by the axial alkoxy group.^21,22^ The expected highly *anti*-selective substitution of conformer **18c** was not observed, however. The presence of the inductively withdrawing benzyloxy substituent might lead to stronger participation of the carbonyl group,^23^ which increases the energy barrier of the S_N_2 substitution reaction. Reactions of acetal **7c** would then proceed by an S_N_1 pathway instead and, therefore, have lower selectivity upon reacting with stronger nucleophiles due to the erosion of selectivity when reaction rates approach the diffusion limit.^24^

With some degree of stereochemical control achieved, the participating group was optimized. In general, participating groups with better donating ability induced higher selectivity. Reactions of acetal **20** bearing the electron-withdrawing tosyl group were unselective (Table 2, entry 1). This result is consistent with the unselective reactions of the analogous β-benzyloxy acetal, in which remote participation is unlikely.^25^ Reactions of carbamate **25** gave the highest selectivities (entry 6). The difference in diastereoselectivity between reactions of acyloxy acetals **21**–**27** is not significant, however, due to the inherent inductive withdrawing ability of the participating carbonyl groups, which destabilize the oxocarbenium ion. The small difference in selectivity is also consistent with the small difference in the hydrolysis rate that these carbonyl groups imposed upon hydrolysis of carbohydrate derivatives.^26^

**Table 2:**
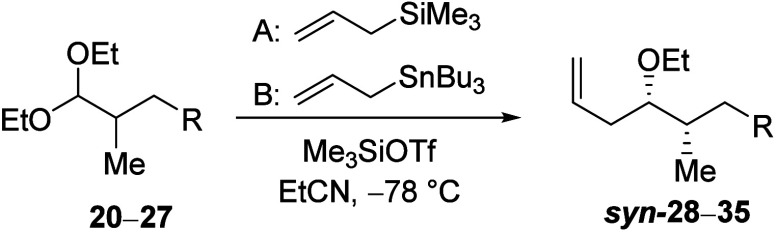
Effect of Different Participating Groups on the Stereoselectivity of Allylation of β-Acyloxy Acetals^a^

			*syn*:*anti*^b^	
Entry	R	Acetal	A	B
1	OTs	**20**	52:48	48:52
2	4-NO_2_C_6_H_4_CO_2_	**21**	60:40	72:28
3	4-OMeC_6_H_4_CO_2_	**22**	62:38	75:25
4	OCO_2_Et	**23**	53:47	74:46
5	OCO^*c*^Pr	**24**	58:42	76:24
6	OCONH^*t*^Bu	**25**	72:28	84:16
7	OCONHPh	**26**	60:40	82:18
8	NHBoc	**27**	53:47	70:30

a Isolated yields of 58–87% on a preparative scale.

b Diastereomeric ratios were determined from ^1^H and ^13^C NMR spectroscopic analysis of the crude reaction mixtures.^17^

The extent of remote participation was tested with reactions of γ-acyloxy acetals that should proceed through seven-membered-ring intermediates. Allyltributylstannane reacted with γ-pivaloyl acetal **36** to give moderate *anti* selectivity (Table 3, entry 1). Using bulky silyl ketene acetal **13**, higher *anti* selectivity was achieved (entry 2). The reaction of carbamoyloxy acetal **37** also proceeded with moderate *anti* selectivity (entry 3). Reactions of dibenzyl acetal **38** (entries 4 and 5) resulted in stereoselectivities similar to those of reactions of diethyl acetal **36**. The products derived from acetal **38** have the advantage that they can be debenzylated to reveal a hydroxyl group,^27^ enabling further modifications. In contrast to reactions of acyloxy acetals, reactions of benzyl-protected acetal **39** occurred with lower selectivities (entries 6 and 7), which indicate that the carbonyl group is necessary for participation.

**Table 3:**
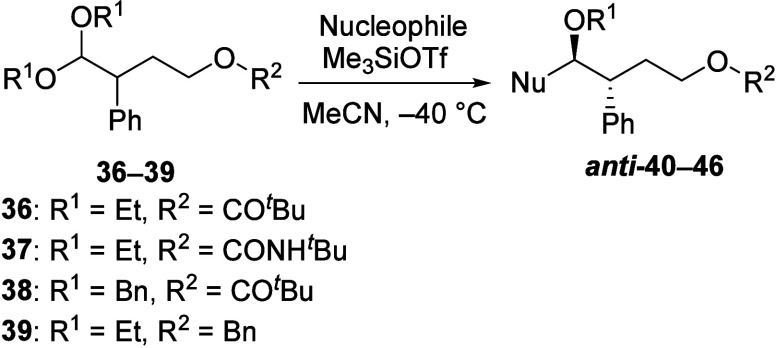

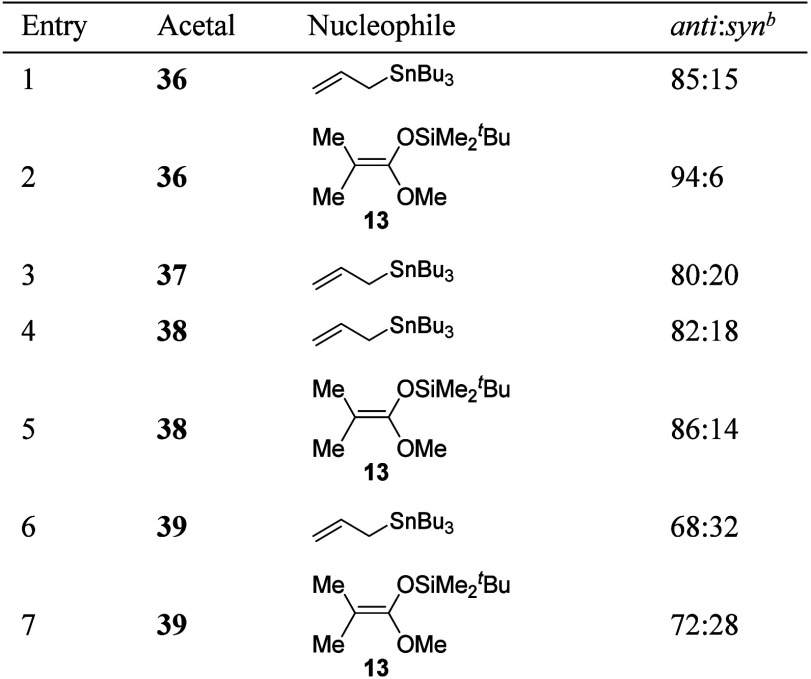
Substitution Reactions of γ-Acyloxy and γ-Alkoxy Acetals Bearing an α-Phenyl Substituent^a^

a Isolated yields of 76–95% on a preparative scale.

b Diastereomeric ratios were determined from ^1^H and ^13^C NMR spectroscopic analysis of the crude reaction mixtures.^17^

The *anti*-selective reactions of γ-acyloxy acetals could also be explained by considering the substitution pathways of the dioxocarbenium ion intermediates. The large α-phenyl substituent of the carbocation derived from acetal **36** also experienced eclipsing interactions with the nucleophile in the pathway that involved the lowest-energy *cis* conformer.^20^ The major *anti* product was, therefore, formed through the substitution pathway that involves *trans* conformer **47** (Scheme 2), which avoids such eclipsing interactions.

**Scheme 2:**
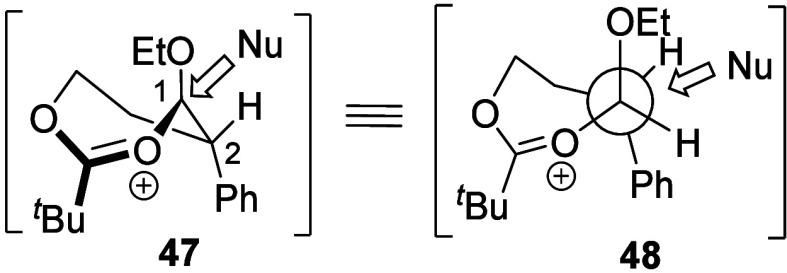
Preferred Reaction Pathway of the Seven-Membered-Ring Dioxocarbenium Intermediate Newman projections viewed down the C1–C2 bond.

1,2-Asymmetric induction through eight- and nine-membered-ring intermediates was not as selective as that of smaller analogues. Reactions of δ-acyloxy acetals **49** and **50** with allyltributylstannane both resulted in low *syn* selectivity (Table 4, entries 1 and 2, respectively). Using bulkier nucleophile **13**, low *anti* selectivity was observed instead (entries 3 and 4). Reactions of ε-acyloxy acetals, which could proceed via nine-membered-ring dioxocarbenium ions, followed the same trend in selectivity (entries 5–7).

**Table 4:**
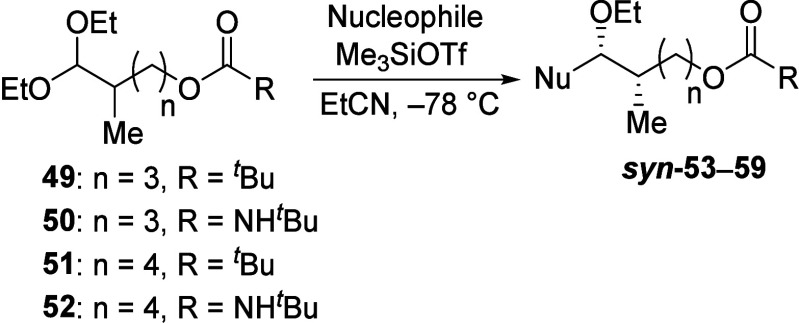

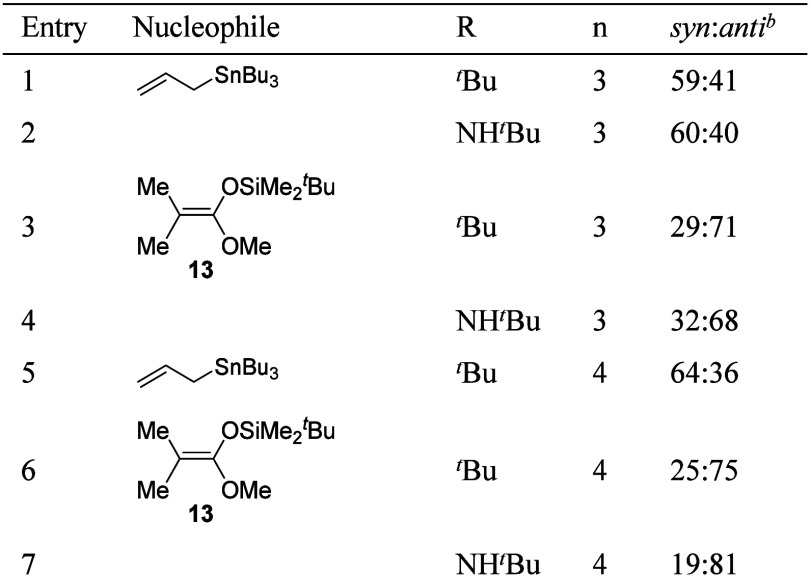
Substitution Reactions of δ-Acyloxy and ε-Acyloxy Acetals Bearing an α-Methyl Substituent^a^

a Isolated yields of 59–97%.

b Diastereomeric ratios were determined from ^1^H and ^13^C NMR spectroscopic analysis of the crude reaction mixtures.^17^

Aside from the covalent cyclic intermediates, reactions of the eight- and nine-membered ring also involve electronically stabilized intermediates (Scheme 3).^20^ Intermediate **62**, in which the C1–O8 bond distance is ∼2.20 Å, could avoid unfavorable transannular interactions present in covalent intermediates such as that between the H5 atom and the ethoxy group.^28,29^ The longer bond distance and the trigonal geometry of the C1 atom of intermediate **62** suggest that the remote carbonyl group participates by electrostatic stabilization.^25^ Nucleophilic addition to this intermediate can occur via S_N_1 substitution pathways on either face of the electrophilic carbonyl group, which is consistent with the less selective reactions of δ- and ε-acyloxy acetals compared to those of β- and γ-acyloxy acetals.

**Scheme 3:**
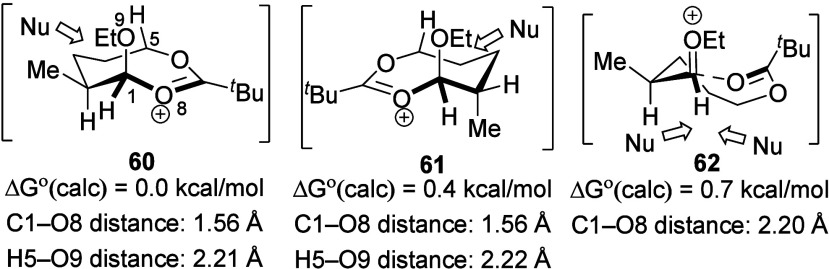
Reaction Pathways of Eight-Membered-Ring Intermediates Bearing an α-Methyl Substituent

1,3-Asymmetric induction was also observed in reactions of δ-acyloxy acetals, which could react through an eight-membered cyclic intermediate. δ-Acyloxy acetal **63** reacted with allyltributylstannane to give an 88:12 mixture of products (Table 5, entry 1). Diastereoselectivities were lower in the reactions of related dibenzyl acetals **64** and **65** (entries 2 and 3, respectively).

**Table 5:**
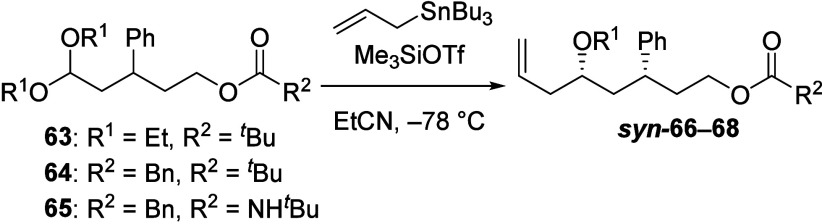
Substitution Reactions of δ-Pivaloyl Acetals Bearing a β-Phenyl Substituent^a^

Entry	R^1^	R^2^	*syn*:*anti*^b^
1	Et	^*t*^Bu	88:12
2	Bn	^*t*^Bu	58:42
3	Bn	NH^*t*^Bu	65:35

a Isolated yields of >74%.

b Diastereomeric ratios were determined from ^1^H and ^13^C NMR spectroscopic analysis of the crude reaction mixtures.^17^

Electrostatic stabilization was predicted to be the preferred mode of participation in the eight-membered cyclic intermediate of acetal **63** (Scheme 4). The major *syn* product is likely formed from nucleophilic addition to higher-energy *trans* conformer **70**.^20^ Nucleophilic addition to conformer **70** likely placed the nucleophile farther from the phenyl substituent compared to nucleophilic addition to conformer **69**.

**Scheme 4:**
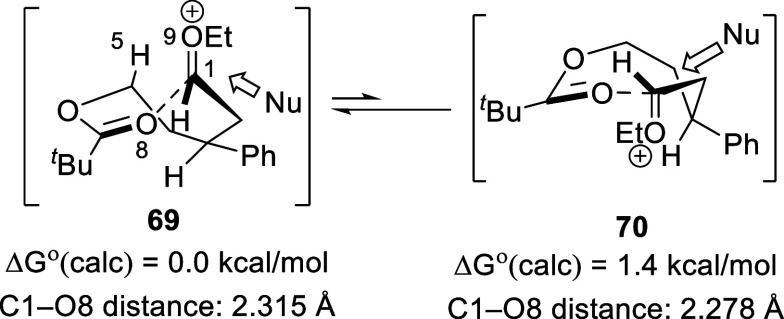
Substitution Pathways of the Eight-Membered-Ring Oxocarbenium Ion Intermediates

1,3-Asymmetric induction was not observed in reactions of acyloxy acetals that could proceed through seven- and nine-membered intermediates. γ-Acyloxy acetals **71** and **72** bearing a β-alkyl substituent reacted unselectively with all nucleophiles tested (Table 6). The β-methyl substituent in this case could be too small to differentiate between the nucleophilic additions to either conformer of the dioxocarbenium ion intermediate. Similarly, reactions of methyl-substituted ε-acyloxy acetals **77** and **79** also proceeded with low selectivity (eqs 3 and 4).
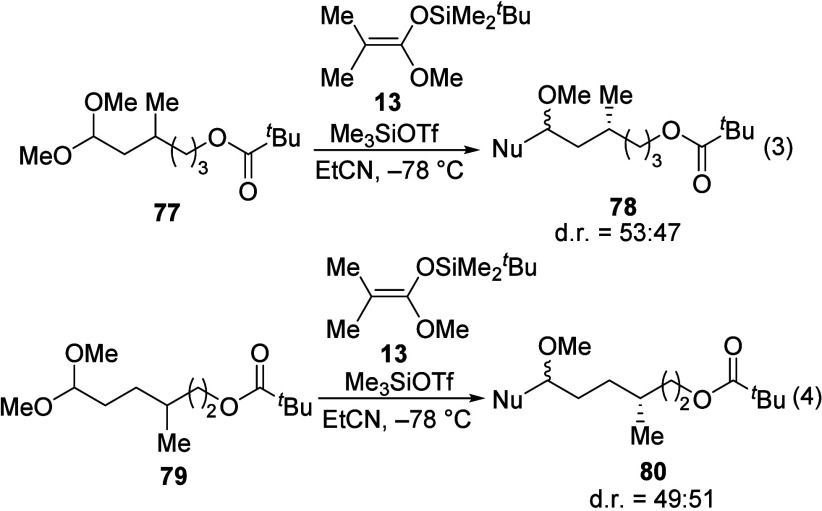
3

**Table 6:**
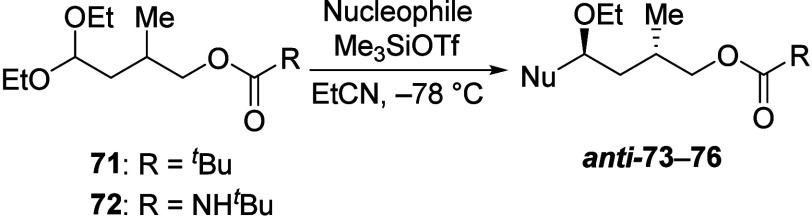

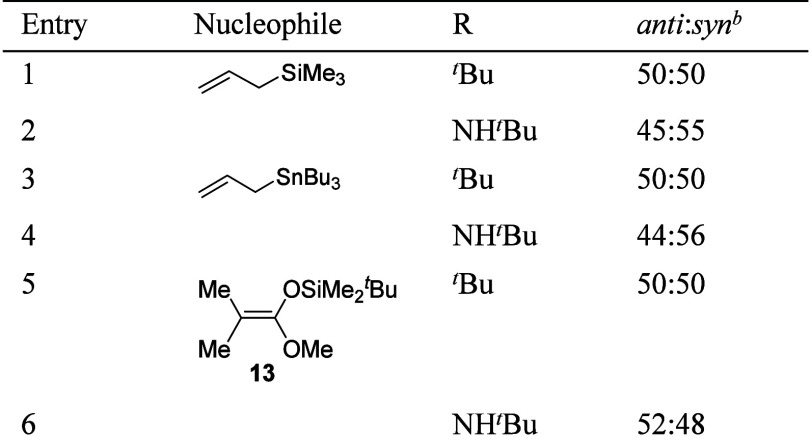
Substitution Reactions of γ-Acyloxy Acetals Bearing a β-Methyl Substituent^a^

a Isolated yield of >60%.

b Diastereomeric ratios were determined from ^1^H and ^13^C NMR spectroscopic analysis of the crude reaction mixtures.^17^

In summary, the presence of a remote carbonyl protecting group can induce 1,2-asymmetric induction in reactions of acyclic acetals with strong nucleophiles. The diastereoselectivity of the substitution reactions depended on the size of the nucleophile and that of the alkyl substituent. 1,3-Asymmetric induction was observed only for reactions that proceed through an eight-membered-ring intermediate.

## Data Availability

The data underlying this study are available in the published article and its Supporting Information.

## Supporting Information Available

Detailed experimental procedures, computational analysis, X-ray data, and ^1^H and ^13^C NMR spectra of new compounds (PDF)
