# Supplementary material for: Diastereoselective Substitution Reactions of Acyclic Acetals Controlled by Remote Participation of an Acyloxy Group
Source: Org Lett. 2024 Dec 2;26(49):10470–4. doi: 10.1021/acs.orglett.4c03766 (PMC11650769; doi:10.1021/acs.orglett.4c03766)
Supplement: Supplementary file 1 — ol4c03766_si_001.pdf [file ol4c03766_si_001.pdf]

Supporting Information

Diastereoselective Substitution Reactions of Acyclic Acetals Controlled by Remote Participation of an Acyloxy Group

Khoi B. Luu, Amanda Ramdular, Eli Finkelstein, Alexander G. Shtukenberg, and K. A. Woerpel*

Department of Chemistry, New York University 100 Washington Square East, New York, NY 10003, United States *Email: kw63@nyu.edu

Contents

General procedure 1

Substrate synthesis and substitution reactions 3

Derivatization and stereochemical correlation 50

Computational analysis 55

X-ray crystallography 114

References...118

^{1}H and ^{13}C NMR spectra 119

General procedure

^{1}H and ^{13}C NMR spectra were obtained at 22 °C using a Bruker AV-400 spectrometer (400 and 100 MHz). The data are reported as follows: chemical shift in ppm referenced to residual solvent (^{1}H NMR: $CDCl_3$ δ 7.26; ^{13}C NMR: $CDCl_3$ δ 77.16)[1], multiplicity (br = broad, s = singlet, d = doublet, t = triplet, q = quartet, qnt = quintet, sxt = sextet, spt = septet, m = multiplet), coupling constants (Hz), and integration. Multiplicity of carbon peaks were determined using HSQC, DEPT 135, HMBC, and COSY experiments. Infrared (IR) spectra were recorded using a Nicolet iS50 FT-IR spectrometer through attenuated total reflectance (ATR). High resolution mass spectra (HRMS) were acquired on an Agilent 6224 Accurate-Mass time-of-flight LC/TOF spectrometer with atmospheric pressure chemical ionization (APCI) or electrospray (ESI) ionization source as indicated. Analytical thin layer chromatography (TLC) was performed on silica gel 60 Å F254 plates. Acetal substrates were visualized by incubating in an iodine chamber for 5 min and then immediately dipping in cerium ammonium molybdate (CAM) stain. Products appeared as white to yellow spots without heating. All reactions were performed under an atmosphere of nitrogen in glassware that had been flame-dried under vacuum unless otherwise stated. Aqueous solutions were prepared from nanopure water with a resistivity over 18 MΩ-cm. Unless otherwise stated, all reagents and substrates were commercially available.

General procedure for ozonolysis

To a cooled (–78 °C) solution of alkene (1.0 equiv) in CH_2Cl_2 (0.25 M) was applied a steady stream of O_3 until saturation was reached and a purple color appeared (30 min). The reaction mixture was warmed to 22 °C over 30 min, allowed to stir for 30 min, and purged with nitrogen for 10 min. Dimethyl sulfide was added (10 equiv) and the reaction mixture was stirred for an additional 5 h. The resulting mixture was concentrated *in vacuo* to provide the corresponding aldehyde, which was used directly in the next step without further purification.

General procedure for esterification of alcohols

To a cooled (0 ºC) solution of alcohol (1.0 equiv) in CH_2Cl_2 (1.0 M) were added Et_3N (2.0 equiv) and 4-dimethylaminopyridine (0.10 equiv). After 30 min, the corresponding acyl chloride (2.0 equiv) was added dropwise. The reaction mixture was warmed to 22 °C and stirred for an additional 8 h. A 5% HCl (20 mL per 10 mmol of alcohol) was added, and the aqueous layer was extracted with CH_2Cl_2 (2 x 30 mL per 10 mmol of alcohol). The combined organic layers were washed with brine (20 mL per 10 mmol of alcohol) and dried over anhydrous Na_2SO_4. The resulting mixture was filtered, concentrated *in vacuo*, and purified by column chromatography (EtOAc:hexanes) to provide the corresponding ester.

General procedure for diethyl acetal formation

To a solution of aldehyde (1.0 equiv) in triethyl orthoformate (10 equiv) was added *p*-toluenesulfonic acid (0.5 equiv) or camphorsulfonic acid (0.5 equiv) and EtOH (0.10 M). After 8 h, saturated aqueous $NaHCO_3$ solution (1 mL per mmol of aldehyde) was added, and the aqueous layer was extracted with CH_2Cl_2 (2 × 5 mL per mmol of aldehyde). The combined organic layers were washed with brine (1 mL per mmol of aldehyde) and dried over anhydrous Na_2SO_4. The resulting mixture was filtered, concentrated *in vacuo*, and purified by flash column chromatography (EtOAc:hexanes) to provide the corresponding acetal.

General procedure for dibenzyl acetal formation

To a solution of diethyl acetal (1.0 equiv) in CH_2Cl_2 (0.10 M) at −78 ºC was added benzyl alcohol (6.0 equiv). After 30 min, trimethylsilyl trifluoromethanesulfonate (4.0 equiv) was added. After 16 h, a 1:1:1 (v/v/v) mixture of CH_2Cl_2:EtOH:Et_3N was added (10 mL per 1 mmol of acetal), and the reaction mixture was warmed to 22 °C over 30 min. The reaction mixture was then washed with 5% HCl (10 mL per mmol of acetal) and the aqueous layer was extracted with Et_2O (2 × 10 mL per mmol of acetal). The combined organic layers were washed with saturated aqueous $NaHCO_3$ solution (10 mL per mmol of acetal) and brine (10 mL per mmol of solution) and dried over anhydrous Na_2SO_4. The resulting mixture was filtered, concentrated *in vacuo*, and purified by flash column chromatography (Et_2O:hexanes) to provide the corresponding dibenzyl acetal**.**

General procedure for ester reduction

To a cooled (0 °C) solution of lithium aluminium hydride (1.5 equiv) in Et_2O (0.10 M) was added the ester (1.0 equiv) dropwise. The mixture was stirred at 22 °C for 16 h, after which saturated aqueous NH_4Cl solution was added dropwise (5 mL per mmol of ester). The layers were separated, and the aqueous layer was extracted with Et_2O (3 × 10 mL per mmol of ester). The combined organic layers were washed with brine (10 mL per mmol of ester) and dried over anhydrous

Na_2SO_4. The resulting mixture was filtered and concentrated *in vacuo* to provide the corresponding alcohol.

General procedure for carbamate formation

To a cooled (0 °C) solution of alcohol (1.0 equiv) in THF (0.10 M) was added NaH (2.0 equiv, 60% dispersion in mineral oil). After 30 min, the corresponding isocyanate (1.5 equiv) was added, and the mixture was stirred at 22 °C for 16 h. H_2O (10 mL per mmol of alcohol) was added dropwise, and the mixture was extracted with EtOAc (2 × 10 mL per mmol of alcohol). The combined organic layers were dried over anhydrous Na_2SO_4, filtered, and concentrated *in vacuo*. The crude mixture was purified by flash column chromatography (EtOAc:hexanes) to provide the corresponding carbamate.

General procedure for acetal substitution reaction

To a cooled (−78 °C) solution of the acetal (1.0 equiv) in solvent (0.10 M) and activated molecular sieves (about 0.10 g per mmol of acetal) was added the nucleophile (4.0 equiv), followed by trimethylsilyl trifluoromethanesulfonate (2.0 equiv). After 16 h, a 1:1:1 (v/v/v) mixture of CH_2Cl_2:EtOH:Et_3N (1 mL per mmol of acetal) was added, and the reaction mixture was warmed to 22 °C over 30 min. The reaction mixture was washed with 5% HCl (5 mL per mmol of acetal) and the aqueous layer was extracted with Et_2O (2 × 10 mL per mmol of acetal). The combined organic layers were washed with saturated aqueous $NaHCO_3$ (10 mL per mmol of acetal) and brine (10 mL per mmol of acetal) and dried over anhydrous Na_2SO_4. The resulting mixture was filtered and concentrated *in vacuo*. The diastereomeric and product ratios were determined by ^1H and ^{13}C NMR spectroscopic analysis of the crude reaction mixture.[2] The residue was purified by flash column chromatography (EtOAc:hexanes) to provide the product.

Substrate synthesis and substitution reactions

Scheme 1: Synthesis of δ-acyloxy acetals bearing an β-phenyl substituent

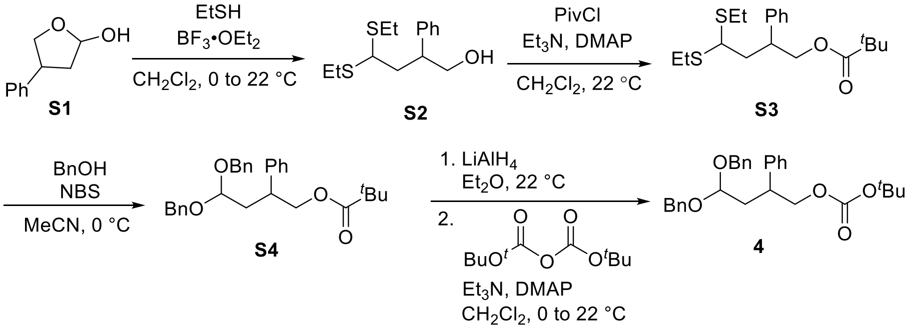

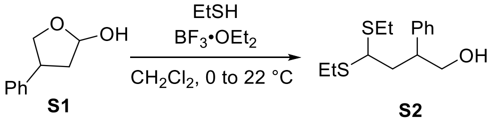

4,4-Bis(ethylthio)-2-phenylbutan-1-ol (S2). Lactol **S1** was prepared according to a literature procedure (7.7 g, 93%).[3] To a cooled (0 °C) solution of lactol **S1** (7.7 g, 47.0 mmol) in CH_2Cl_2 (100 mL) were added ethanethiol (14 mL, 190 mmol) and BF_3•OEt_2 (4.9 mL, 47 mmol). The reaction

mixture warmed to 22 °C over 1 h and stirred for an additional 2 h. A 5% HCl solution (100 mL) was added, and the aqueous layer was extracted with CH_2Cl_2 (2 × 50 mL). The combined organic layers were washed with brine (100 mL) and dried over anhydrous Na_2SO_4. The resulting mixture was filtered, concentrated *in vacuo*, and purified by column chromatography (20:80 EtOAc:hexanes) to provide thioacetal **S2** as an amber oil (13.4 g, 99%):

^1H NMR (400 MHz, $CDCl_3$) δ 7.34–7.30 (m, 2H), 7.25–7.12 (m, 3H), 4.30–4.13 (m, 2H), 3.53–3.38 (m, 2H), 2.67–2.43 (m, 4H), 2.21–2.08 (m, 2H), 1.17 (t, *J* = 7.4 Hz, 3H), 1.16 (t, *J* = 7.4 Hz, 3H);

^{13}C NMR (100 MHz, $CDCl_3$) δ 140.0 (C), 128.9 (CH), 128.2 (CH), 127.4 (CH), 71.5 (CH_2), 48.8 (CH), 42.7 (CH), 38.6 (CH_2), 24.4 (CH_2), 23.6 (CH_2), 14.6 (CH_3), 14.5 (CH_3);

IR (ATR) 3375 (br), 2963, 2924, 2868, 1452 cm^{-1};

HRMS (ESI/TOF) m/z: $[M + Na]^+$ Calcd for $C_{14}H_{22}NaOS_2$ 293.1010; Found 293.1015.

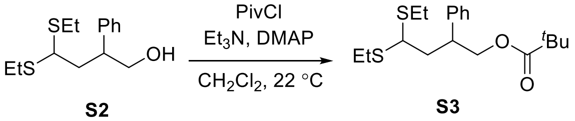

4,4-Bis(ethylthio)-2-phenylbutyl pivalate (S3). Following the general procedure for esterification of alcohols, thioacetal **S2** (13.4 g, 49 mmol) in CH_2Cl_2 (100 mL) was mixed with triethylamine (14 mL, 99 mmol), 4-dimethylaminopyridine (0.60 g, 5.0 mmol), and pivaloyl chloride (12 mL, 99 mmol) to provide ester **S3** as a colorless oil (15.6 g, 90%):

^1H NMR (400 MHz, $CDCl_3$) δ 7.36–7.28 (m, 2H), 7.24–7.22 (m, 3H), 4.25 (dd, *J* = 10.9, 6.7 Hz, 1H), 4.12 (dd, *J* = 10.9, 7.0 Hz, 1H), 3.55–3.38 (m, 2H), 2.71–2.44 (m, 4H), 2.21–2.07 (m, 2H), 1.19 (t, *J* = 7.5 Hz, 3H), 1.15 (t, *J* = 7.4 Hz, 3H), 1.13 (s, 9H);

^{13}C NMR (100 MHz, $CDCl_3$) δ 178.4 (C), 140.6 (C), 128.8 (CH), 128.2 (CH), 127.2 (CH), 67.9 (CH_2), 48.8 (CH), 42.8 (CH), 38.9 (C), 38.7 (CH_2), 27.3 (CH_3), 24.3 (CH_2), 23.6 (CH_2), 14.61 (CH_3), 14.56 (CH_3);

IR (ATR) 2988, 2928, 2871, 1727, 1280 cm^{-1};

HRMS (ESI/TOF) m/z: $[M + Na]^+$ Calcd for $C_{19}H_{30}NaO_2S_2$ 377.1585; Found 377.1586.

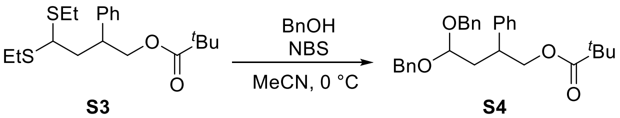

4,4-Bis(benzyloxy)-2-phenylbutyl pivalate (S4). To a cooled (0 ºC) solution of ester **S3** (3.5 g, 10 mmol) in MeCN (200 mL) were added benzyl alcohol (8.3 mL, 80 mmol) and *N*-bromosuccinamide (8.7 g, 40 mmol). After 2 h, a saturated aqueous solution of $Na_2S_2O_3$ (150 mL) was added, and the aqueous layer was extracted with EtOAc (2 × 75 mL). The combined organic layers were washed with brine (100 mL) and dried over anhydrous Na_2SO_4 The resulting mixture was filtered, concentrated *in vacuo*, and purified by column chromatography (5:95 Et_2O:hexanes) to provide dibenzyl acetal **S4** (3.3 g, 74%) as a colorless oil:

^{1}H NMR (400 MHz, $CDCl_3$) δ 7.46–7.26 (m, 10H), 7.25–7.18 (m, 3H), 7.15–7.11 (m, 2H), 4.64 (d, *J* = 11.6 Hz, 1H), 4.61–4.53 (m, 2H), 4.46 (d, *J* = 11.6 Hz, 1H), 4.45 (d, *J* = 11.6 Hz, 1H), 4.21 (dd, *J* = 10.9, 7.2 Hz, 1H), 4.11 (dd, *J* = 10.9, 6.7 Hz, 1H), 3.20–3.09 (m, 1H), 2.27–2.21 (m, 1H), 2.04 (ddd, *J* = 14.0, 6.8, 3.9 Hz, 1H), 1.09 (s, 9H);

^{13}C NMR (100 MHz, $CDCl_3$) δ 178.4, 141.2, 138.2, 138.1, 128.64, 128.59, 128.56, 128.12, 128.07, 127.9, 127.82, 127.77, 126.9, 100.5, 68.1, 67.7, 67.6, 41.2, 38.9, 36.2, 27.3;

IR (ATR) 3031, 2970, 1723, 1283, 1024 cm^{-1};

HRMS (ESI/TOF) m/z: [M + H − BnOH]$^+$ Calcd for $C_{22}H_{27}O_3$ 339.1955; Found 339.1950.

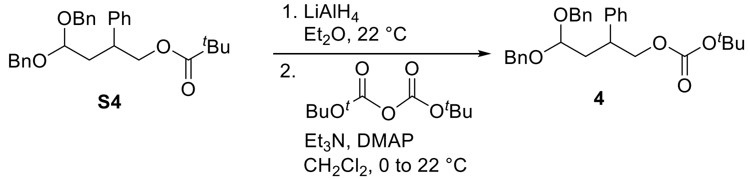

4,4-Bis(benzyloxy)-2-phenylbutyl *tert*-butyl carbonate (4). Following the general procedure for ester reduction, ester **S4** (1.7 g, 3.8 mol) in Et_2O (40 mL) was mixed with lithium aluminum hydride (0.22 g, 5.7 mol) to provide the crude alcohol (1.45 g, quantitative), which was used in the next step without further purification.

Following the general procedure for esterification of alcohols, the crude alcohol (0.73 g, 2.0 mmol) in CH_2Cl_2 (20 mL) was mixed with triethylamine (0.50 mL, 4.0 mol) and 4-dimethylaminopyridine (0.024 g, 0.2 mmol), and di-*tert*-butyl dicarbonate (0.87 g, 4.0 mol) to provide carbonate **4** as a colorless oil (0.42 g, 45%):

^{1}H NMR (400 MHz, $CDCl_3$) δ 7.40–7.28 (m, 10H), 7.25–7.21 (m, 3H), 7.15–7.12 (m, 2H), 4.63 (d, *J* = 8.5 Hz, 1H), 4.71–4.59 (m, 3H), 4.41 (d, *J* = 11.6 Hz, 1H), 4.31–4.11 (m, 2H), 3.22–3.11 (m, 1H), 2.36–2.24 (m, 1H), 2.07–2.01 (m, 1H), 1.42 (s, 9H);

^{13}C NMR (100 MHz, $CDCl_3$) δ 153.6 (C), 140.8 (C), 138.24 (C), 138.15 (C), 128.8 (CH), 128.6 (CH), 128.5 (CH), 128.13 (CH), 128.12 (CH), 127.9 (CH), 127.8 (CH), 127.7 (CH), 127.1 (CH), 100.5 (CH), 82.1 (C), 70.63 (CH_2), 67.8 (CH_2), 67.4 (CH_2), 41.3 (CH), 36.3 (CH_2), 27.9 (CH_3);

IR (ATR) 3030, 2956, 1737, 1252, 1037 cm^{-1};

HRMS (ESI/TOF) m/z: [M + NH_4]$^+$ Calcd for $C_{29}H_{38}NO_5$ 480.2750; Found 480.2737.

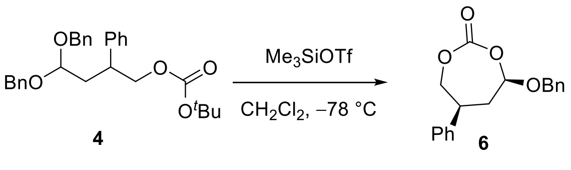

(4*R,6*S**)-4-(Benzyloxy)-6-phenyl-1,3-dioxepan-2-one (6)**. Following the general procedure for acetal substitution, acetal **4** (0.046 g, 0.10 mmol) in CH_2Cl_2 (1.0 mL) was mixed with trimethylsilyl trifluoromethanesulfonate (0.020 mL, 0.11 mmol) to provide cyclic carbonate **6** as a colorless oil (0.009 g, 30%):

^{1}H NMR (400 MHz, $CDCl_3$) δ 7.43–7.28 (m, 7H), 7.25–7.16 (m, 3H), 5.39 (d, *J* = 5.0 Hz, 1H), 4.81 (d, *J* = 11.8 Hz, 1H), 4.53 (d, *J* = 11.8 Hz, 1H), 4.37 (t, *J* = 8.3 Hz, 1H), 3.88 (dd, *J* = 8.3, 7.2 Hz, 1H), 3.70 (dddd, *J* = 9.4, 8.3, 7.7, 7.2 Hz, 1H), 2.42 (dd, *J* = 13.0, 7.7 Hz, 1H), 2.12 (ddd, *J* = 13.0, 9.4, 5.0 Hz, 1H);

^{13}C NMR (100 MHz, $CDCl_3$) δ (100 MHz, $CDCl_3$) δ 155.6 (C), 140.6 (C), 138.2 (C), 128.8 (CH), 128.58 (CH), 128.55 (CH), 128.13 (CH), 128.10 (CH), 127.9 (CH), 100.5 (CH), 71.5 (CH_2), 67.8 (CH_2), 41.3 (CH), 36.2 (CH_2);

IR (ATR) 3062, 3029, 2953, 1741, 1252 cm^{-1};

HRMS (ESI/TOF) m/z: $[M + K]^+$ Calcd for $C_{18}H_{18}KO_4$ 337.0842; Found 337.0827.

*Scheme 2: Synthesis of β-acyloxy acetal **7c** bearing an α-benzyloxy substituent.*

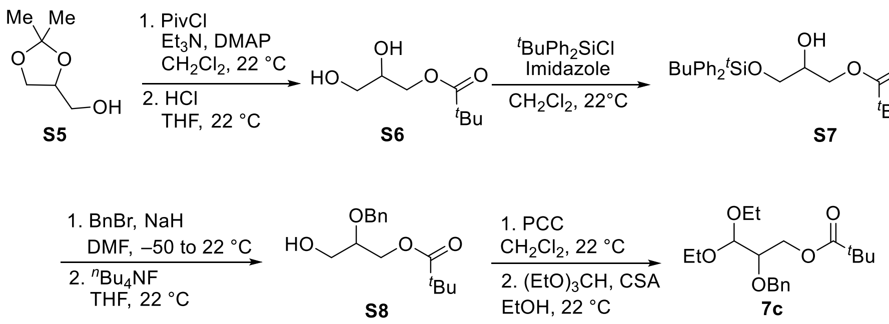

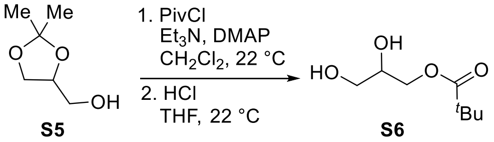

2,3-Dihydroxypropyl pivalate (S6). Following the general procedure for esterification of alcohols, solketal (10 g, 76 mmol) in CH_2Cl_2 (250 mL) was mixed with triethylamine (11.6 mL, 8.4 mmol), 4-dimethylaminopyridine (0.9 g, 7.6 mmol), and pivaloyl chloride (11 mL, 9.1 mmol) to provide the corresponding ester, which was used directly in the next step without further purification.

The crude ester was dissolved in THF (50 mL). A 2.0 M HCl solution (20 mL) was added slowly, and the mixture was stirred at 22 °C for 16 h. Anhydrous NaCl was added until saturation (1.0 g) and the mixture was extracted with EtOAc (4 × 50 mL). The combined organic layers were dried over anhydrous Na_2SO_4, filtered, and concentrated *in vacuo* to provide dihydroxy ester **S6** as a colorless oil (13.5 g, quantitative), which was used directly in the next step without further purification. The spectroscopic data of ester **S6** are consistent with literature data:[4]

^{1}H NMR (400 MHz, $CDCl_3$) δ 4.21–4.09 (m, 2H), 3.95–3.89 (m, 1H), 3.69 (dd, *J* = 11.5, 3.9 Hz, 1H), 3.59 (dd, *J* = 11.5, 5.8 Hz, 1H), 3.53–3.19 (s, 2H), 1.21 (s, 9H);

^{13}C NMR (100 MHz, $CDCl_3$) δ 179.3, 70.5, 65.3, 63.5, 39.0, 27.3.

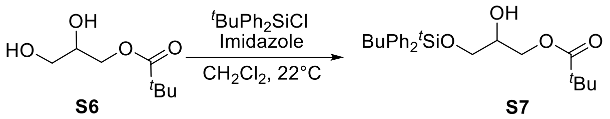

3-((*tert*-Butyldiphenylsilyl)oxy)-2-hydroxypropyl pivalate (S7). To a cooled (0 °C) solution of dihydroxyl ester **S6** (5.3 g, 30 mmol) in DMF were added imidazole (6.1 g, 90 mmol) and *tert*-butyl(chloro)diphenylsilane dropwise (8.6 mL, 33 mmol). The reaction mixture was stirred at 22 °C for 16 h. H_2O (20 mL) was added, and the layers were separated. The aqueous layer was extracted with EtOAc (3 × 30 mL). The combined organic layers were washed with H_2O (3 × 10 mL) and brine (10 mL) and dried over anhydrous Na_2SO_4. The resulting mixture was filtered, concentrated *in vacuo*, and purified by flash column chromatography (15:85 EtOAc:hexanes) to provide hydroxy ester **S7** as a colorless oil (10.4 g, 84%):

^{1}H NMR (400 MHz, $CDCl_3$) δ 7.67– 7.64 (m, 4H), 7.47–7.37 (m, 6H), 4.19–4.16 (m, 2H), 3.93 (quind, *J* = 5.5, 4.7 Hz, 1H), 3.71 (dd, *J* = 10.3, 4.7 Hz, 1H), 3.66 (dd, *J* = 10.3, 5.5 Hz, 1H), 2.50 (d, *J* = 5.5 Hz, 1H), 1.16 (s, 9H), 1.07 (s, 9H);

^{13}C NMR (100 MHz, $CDCl_3$) δ 178.7 (C), 135.7 (CH), 133.0 (C), 130.1 (CH), 128.0 (CH), 70.3 (CH), 65.0 (CH_2), 64.6 (CH_2), 38.0 (C), 27.3 (CH_3), 27.0 (CH_3), 19.4 (C);

IR (ATR) 3409 (br), 2957, 1729, 1156, 700 cm^{-1};

HRMS (ESI/TOF) m/z: $[M + Na]^+$ Calcd for $C_{24}H_{34}NaO_4Si$ 437.2119; Found 437.2133.

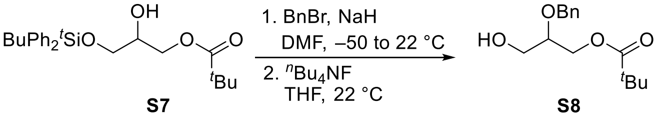

2-(Benzyloxy)-3-hydroxypropyl pivalate (S8). To a cooled solution (–50 °C) of hydroxy ester **S7** (7.6 g, 15.2 mmol) in DMF (90 mL) were added benzyl bromide (6.5 mL, 53 mmol) and NaH (1.5 g, 60% dispersion in mineral oil, 36 mmol). The reaction mixture was warmed to –10 °C over 1 h, stirred for 30 min, and warmed to 22 °C. After 1.5 h, saturated aqueous NH_4Cl (10 mL) was added dropwise. The mixture was extracted with Et_2O (3 × 30 mL). The combined organic layers were washed with brine (10 mL) and dried over anhydrous Na_2SO_4. The resulting mixture was filtered, concentrated *in vacuo*, and purified by flash column chromatography (5:95 EtOAc:hexanes) to provide the corresponding benzylated ester as a colorless oil (6.2 g, 66%), which was used in the next step without further purification.

To a solution of the crude benzylated ester (6.0 g, 12 mmol) in THF (12 0 mL) was added a solution of tetrabutylammonium fluoride (24 mL, 1.0 M in THF). The reaction mixture was stirred at 22 °C for 16 h. The resulting mixture was concentrated *in vacuo* and purified by flash column chromatography (30:70 EtOAc:hexanes) to provide hydroxy ester **S8** as a colorless oil (2.0 g, 63%):

^{1}H NMR (400 MHz, $CDCl_3$) δ 7.67–7.64 (m, 1H), 7.32–7.28 (m, 4H), 4.48 (s, 2H), 4.10–4.07 (m, 2H), 3.99–3.92 (m, 1H), 3.48 (dd, *J* = 9.6, 4.4 Hz, 1H), 3.42 (dd, *J* = 9.6, 6.0 Hz, 1H), 2.65 (s, 1H), 1.13 (s, 9H);

^{13}C NMR (100 MHz, $CDCl_3$) δ 178.7 (C), 137.8 (C), 134.9 (CH), 128.6 (CH), 127.8 (CH), 73.5 (CH_2), 70.9 (CH_2), 69.0 (CH), 65.4 (CH_2), 38.9 (C), 27.2 (CH_3);

IR (ATR) 3436 (br), 2969, 1725, 1454, 1283 cm^{-1};

HRMS (ESI/TOF) m/z: $[M + H]^+$ Calcd for $C_{15}H_{23}O_4$ 267.1591; Found 267.1597.

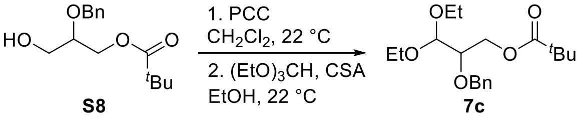

S8 **7c**

2-(Benzyloxy)-3,3-diethoxypropyl pivalate (7c). To a cooled (0 °C) solution of pyridinium chlorochromate (2.3 g, 11 mmol) in CH_2Cl_2 (16 mL) were added molecular sieves (5.0 g) and a solution of hydroxy ester **S8** (1.0 g, 3.8 mmol) in CH_2Cl_2 (7.5 mL) dropwise over 30 min. The reaction mixture was warmed to 22 °C over 1 h and stirred for an additional 3 h. The resulting mixture was filtered over diatomaceous earth, washed with 25:75 EtOAc:hexanes, and concentrated *in vacuo* to provide the crude aldehyde, which was used directly in the next step without further purification.

Following the general procedure for diethyl acetal formation, the crude aldehyde (0.50 g, 1.9 mmol) in triethyl orthoformate (3.2 mL) was mixed with ethanol (0.33 mL) and camphorsulfonic acid (0.22 g, 0.95 mmol) to provide acetal **7c** as a colorless oil (0.15 g, 12% over two steps):

^1H NMR (400 MHz, $CDCl_3$) δ 7.38–7.28 (m, 5H), 4.71 (s, 2H), 4.50 (d, *J* = 6.0 Hz, 1H), 4.41 (dd, *J* = 11.9, 2.8 Hz, 1H), 4.12 (dd, *J* = 11.9, 6.5 Hz, 1H), 3.79–3.65 (m, 3H), 3.65–3.53 (m, 2H), 1.23 (t, *J* = 7.1 Hz, 6H), 1.21 (s, 9H);

^{13}C NMR (100 MHz, $CDCl_3$) δ 178.5 (C), 138.6 (C), 128.5 (CH), 127.9 (CH), 127.7 (CH), 102.8 (CH), 78.4 (CH), 73.3 (CH_2), 64.1 (CH_2), 63.93 (CH_2), 63.89 (CH_2), 38.9 (C), 27.4 (CH_3), 15.6 (CH_3), 15.4 (CH_3);

IR (ATR) 2946, 2874, 1729, 1152, 1110 cm^{-1};

HRMS (ESI/TOF) m/z: $[M + K]^+$ Calcd for $C_{19}H_{30}KO_5$ 377.1725; Found 377.1744.

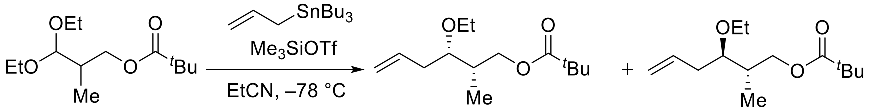

7a ***syn*-8a** ***anti*-8a**

(2*R,3*R**)-3-Ethoxy-2-methylhex-5-en-1-yl pivalate (*syn*-8a) and (2*R**,3*S**)-3-ethoxy-2-methylhex-5-en-1-yl pivalate (*anti*-8a)**. Following the general procedure for acetal substitution, acetal **7a** (0.37 g, 1.5 mmol) in EtCN (15 mL) was mixed with allyltributylstannane (1.9 mL, 6.0 mmol) and trimethylsilyl trifluoromethanesulfonate (0.54 mL, 3.0 mmol) to provide a 75:25 mixture of diastereomers of ester **8a**. Ester **8a** was isolated as a 74:26 mixture of ***syn*-8a**:***anti*-8a** with some stannane impurities as a colorless oil (0.21 g, 58%):

^{1}H NMR (400 MHz, $CDCl_3$) δ 5.93–5.74 (m, 1.4H), 5.14–5.01 (m, 2.8H), 4.12 (dd, *J* = 10.8, 4.7 Hz, 0.4H), 4.06–4.02 (m, 1.4H), 3.97 (dd, *J* = 10.8, 6.5 Hz, 1H), 3.63–3.53 (m, 1.4H), 3.46–3.37 (m, 1.4H), 3.32 (td, *J* = 6.5, 3.9 Hz, 1H), 3.22 (td, *J* = 6.7, 4.3 Hz, 0.4H), 2.39–2.30 (m, 1.4H), 2.26–2.18 (m, 1.4H), 2.05–1.91 (m, 1.4H), 1.20 (s, 12.6H), 1.17 (t, *J* = 7.0 Hz, 4.2H), 1.16 (t, *J* = 7.0 Hz, 1.2H), 0.95–0.93 (m, 3H);

Peaks attributed to major diastereomer (***syn*-8a**): ^{13}C NMR (100 MHz, $CDCl_3$) δ 178.6 (C), 135.4 (CH), 117.0 (CH_2), 79.3 (CH), 66.6 (CH_2), 65.9 (CH_2), 38.96 (C), 36.30 (CH_2), 36.0 (CH), 27.4 (CH_3), 15.7 (CH_3), 11.3 (CH_3);

Peaks attributed to minor diastereomer (***anti*-8a**): ^{13}C NMR (100 MHz, $CDCl_3$) δ 178.7 (C), 135.0 (CH), 117.1 (CH_2), 80.2 (CH), 66.5 (CH_2), 65.7 (CH_2), 39.03 (C), 36.25 (CH_2), 35.4 (CH), 27.3 (CH_3), 15.6 (CH_3), 13.5 (CH_3);

IR (ATR) 2958, 2924, 1729, 1461, 1284 cm^{-1};

HRMS (ESI/TOF) m/z: $[M + H]^+$ Calcd for $C_{14}H_{27}O_3$ 243.1955; Found 243.1965.

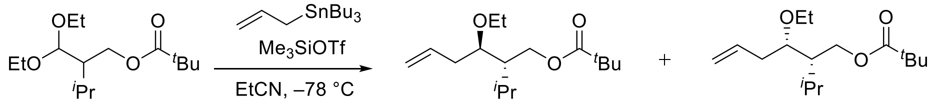

7b anti-8b syn-8b

(2*R,3*S**)-3-Ethoxy-2-isopropylhex-5-en-1-yl pivalate (*anti*-8b) and (2*R**,3*R**)-3-ethoxy-2-isopropylhex-5-en-1-yl pivalate (*syn*-8b).** Acetal **7b** was prepared according to a literature procedure (2.5 g, 29% after four steps). Following the general procedure for acetal substitution, acetal **7b** (0.053 g, 0.21 mmol) in EtCN (2.1 mL) was mixed with allyltributylstannane (0.13 mL, 0.84 mmol) and trimethylsilyl trifluoromethanesulfonate (0.076 mL, 0.42 mmol) to provide a 78:22 mixture of diastereomers of ester **8b**. Ester **8b** was isolated as an 80:20 mixture of ***anti*-8b**:***syn*-8b** as a colorless oil (0.035 g, 61%):

^{1}H NMR (400 MHz, $CDCl_3$) δ 5.92–5.77 (m, 1.3H), 5.11–5.02 (m, 2.6H), 4.24 (dd, *J* = 11.6, 4.3 Hz, 1H), 4.18–4.12 (m, 1.6H), 3.59 (dq, *J* = 9.0, 6.9 Hz, 1H), 3.54–3.38 (m, 1.9H), 3.34 (dq, *J* = 9.0, 7.0 Hz, 1H), 2.36–2.33 (m, 2.3H), 2.26–2.18 (m, 0.3H), 2.00 (sptd, *J* = 6.9, 5.5 Hz, 0.3H), 1.86 (sptd, *J* = 6.9, 4.9 Hz, 1H), 1.69–1.59 (m, 1.3H), 1.19 (s, 11.7H), 1.16 (t, *J* = 7.0 Hz, 0.9H), 1.13 (t, *J* = 7.0 Hz, 3H), 0.97 (d, *J* = 6.9 Hz, 0.9H), 0.96 (d, *J* = 7.0 Hz, 3H), 0.93–0.89 (m, 3.9H);

Peaks attributed to major diastereomer (***anti*-8b**): ^{13}C NMR (100 MHz, $CDCl_3$) δ 178.7 (C), 135.3 (CH), 117.2 (CH_2), 78.5 (CH), 65.25 (CH_2), 62.8 (CH_2), 46.20 (CH), 38.8 (C), 36.5 (CH_2), 27.6 (CH), 27.4 (CH_3), 21.4 (CH_3), 19.8 (CH_3), 15.71 (CH_3);

Peaks attributed to minor diastereomer (***syn*-8b**): ^{13}C NMR (100 MHz, $CDCl_3$) δ 178.6 (C), 135.9 (CH), 116.6 (CH_2), 78.9 (CH), 65.30 (CH_2), 62.1 (CH_2), 46.17 (CH), 38.9 (C), 36.1 (CH_2), 27.3 (CH_3), 26.0 (CH), 22.0 (CH_3), 19.6 (CH_3), 15.69 (CH_3);

IR (ATR) 2970, 1727, 1480, 1284, 1155 cm^{-1};

HRMS (ESI/TOF) m/z: $[M + Na]^+$ Calcd for $C_{16}H_{30}NaO_3$ 293.2087; Found 293.2097.

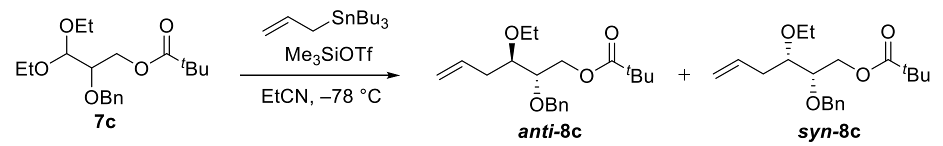

(2*R,3*S**)-2-(Benzyloxy)-3-ethoxyhex-5-en-1-yl pivalate (*anti*-8c) and (2*R**,3*R**)-2-(benzyloxy)-3-ethoxyhex-5-en-1-yl pivalate (*syn*-8c).** Following the general procedure for acetal substitution, acetal **7c** (0.039 g, 0.11 mmol) in EtCN (1.1 mL) was mixed with allyltributylstannane (0.14 mL, 0.46 mmol) and trimethylsilyl trifluoromethanesulfonate (0.042 mL, 0.23 mmol) to provide a 44:56 mixture of diastereomers of ester **8c**. Ester **8c** was isolated as a 22:78 mixture of ***anti*-8c**:***syn*-8c** (0.011 g, 30%) and a 73:27 mixture of ***anti*-8c**:***syn*-8c** (0.016 g, 43%) with some stannane impurities as a colorless oil. Characterization was done on the 73:27 mixture of ***anti*-8c**:***syn*-8c**:

^{1}H NMR (400 MHz, $CDCl_3$) δ 7.38–7.27 (m, 6.8H), 5.91–5.70 (m, 1.4H), 5.13–4.99 (m, 2.8H), 4.74–4.69 (m, 1.4H), 4.61–4.54 (m, 1.4H), 4.49 (dd, *J* = 11.8, 2.9 Hz, 0.4H), 4.31 (dd, *J* = 11.5, 4.5 Hz, 1H), 4.21 (dd, *J* = 11.6, 6.5, Hz, 1H), 4.13 (dd, *J* = 11.9, 5.5 Hz, 0.4H), 3.69–3.64 (m, 1H), 3.63–3.43 (m, 3.6H), 3.43–3.38 (m, 1H), 2.48–2.34 (m, 1.6H), 2.33–2.22 (m, 1H), 1.21 (s, 12.6H), 1.17 (t, *J* = 7.0 Hz, 4.2H);

Peaks attributed to major diastereomer (***anti*-8c**): ^{13}C NMR (100 MHz, $CDCl_3$) δ 178.5 (C), 138.5 (C), 135.2 (CH), 128.5 (CH overlapping with ***syn*-8c** as determined by HSQC), 128.1 (CH), 127.83 (CH), 117.2 (CH_2), 79.3 (CH), 77.8 (CH), 73.2 (CH_2), 66.4 (CH_2), 64.1 (CH_2), 38.9 (C), 35.0 (CH_2), 27.37 (CH_3), 15.7 (CH_3);

Peaks attributed to minor diastereomer (***syn*-8c**): ^{13}C NMR (100 MHz, $CDCl_3$) δ 178.6 (C), 138.4 (C), 134.9 (CH), 128.5 (CH overlapping with ***anti*-8c** as determined by HSQC), 128.0 (CH), 127.79 (CH), 117.4 (CH_2), 78.6 (two CH overlapping, as determined by HSQC), 72.6 (CH_2), 66.0 (CH_2), 63.6 (CH_2), 39.0 (C), 35.3 (CH_2), 27.40 (CH_3), 13.8 (CH_3);

IR (ATR) 2972, 1728, 1283, 1154, 1108 cm^{-1};

HRMS (ESI/TOF) m/z: $[M + H]^+$ Calcd for $C_{20}H_{30}NaO_4$ 357.2036; Found 357.2045.

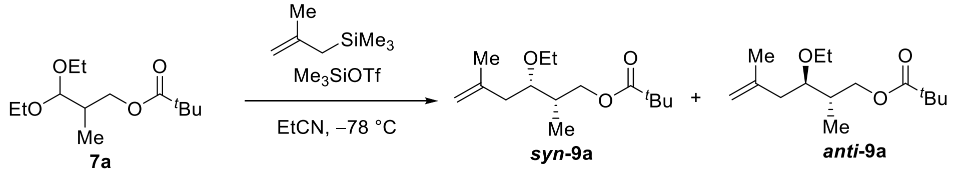

(2*R,3*R**)-3-Ethoxy-2,5-dimethylhex-5-en-1-yl pivalate (*syn*-9a) and (2*R**,3*S**)-3-ethoxy-2,5-dimethylhex-5-en-1-yl pivalate (*anti*-9a).** Following the general procedure for acetal substitutions, acetal **7a** (0.25 g, 1.0 mmol) in EtCN (10 mL) was mixed with methallyltrimethylsilane (0.70 mL, 4.0 mmol) and trimethylsilyl trifluoromethanesulfonate (0.36 mL, 2.0 mmol) to provide an 82:18 mixture of diastereomers of ester **9a**. Ester **9a** was isolated as an 80:20 mixture of ***syn*-9a**:***anti*-9a** as a colorless oil (0.18 g, 69%):

^{1}H NMR (400 MHz, $CDCl_3$) δ 4.83–4.73 (m, 2.4H), 4.14–3.93 (m, 2.4H), 3.64–3.32 (m, 3.6H), 2.30 (ddd, *J* = 14.0, 6.6, 1.1 Hz, 1H), 2.23–2.10 (m, 1.4H), 2.05 (ddd, *J* = 12.4, 7.0, 5.8 Hz, 0.2H), 2.01–1.90 (m, 1H), 1.78 (s, 0.6H), 1.75 (s, 3H), 1.21 (s, 10.8H), 1.15 (t, *J* = 7.0 Hz, 3.6H), 0.95 (d, *J* = 6.9 Hz, 0.6H), 0.91 (d, *J* = 7.0 Hz, 3H);

Peaks attributed to major diastereomer (***syn*-9a**): ^{13}C NMR (100 MHz, $CDCl_3$) δ 178.6 (C), 142.9 (C), 112.83 (CH_2), 77.3 (CH), 66.7 (CH_2), 65.8 (CH_2), 40.2 (CH_2), 38.9 (C), 35.7 (CH), 27.4 (CH_3 overlapping with ***anti*-9a**, as determined by HSQC), 22.8 (CH_3), 15.69 (CH_3), 10.6 (CH_3);

Peaks attributed to minor diastereomer (***anti*-9a**): ^{13}C NMR (100 MHz, $CDCl_3$) δ 178.7 (C), 143.3 (C), 112.80 (CH_2), 79.5 (CH), 66.4 (CH_2), 65.6 (CH_2), 39.6 (CH_2), 39.0 (C), 36.4 (CH), 27.4 (CH_3 overlapping with ***syn*-9a**, as determined by HSQC), 23.2 (CH_3), 15.66 (CH_3), 13.4 (CH_3);

IR (ATR) 2976, 2873, 1721, 1283, 1155 cm^{-1};

HRMS (ESI/TOF) m/z: [M + Na]$^+$ Calcd for $C_{15}H_{28}NaO_3$ 279.1936; Found 279.1936.

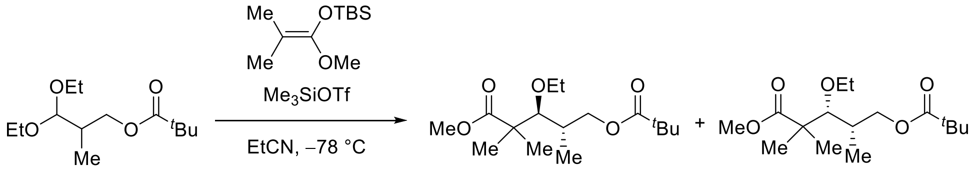

7a ***anti*-10a** ***syn*-10a**

Methyl (3*R,4*R**)-3-ethoxy-2,2,4-trimethyl-5-(pivaloyloxy)pentanoate (*anti*-10a) and methyl (3*R**,4*S**)-3-ethoxy-2,2,4-trimethyl-5-(pivaloyloxy)pentanoate (*syn*-10a).** Following the general procedure for acetal substitutions, acetal **7a** (0.25 g, 1.0 mmol) in EtCN (10 mL) was mixed with silyl ketene acetal *tert*-butyl((1-methoxy-2-methylprop-1-en-1-yl)oxy)dimethylsilane (1.0 mL, 4.0 mmol) and trimethylsilyl trifluoromethanesulfonate (0.36 mL, 2.0 mmol) to provide a 97:3 mixture of diastereomers of ester **10a**. Ester ***anti*-10a** was isolated as a single diastereomer and as a colorless oil (0.23 g, 77%):

^{1}H NMR (400 MHz, $CDCl_3$) δ 3.95 (dd, *J* = 10.8, 7.6 Hz, 1H), 3.88 (dd, *J* = 10.8, 6.8 Hz, 1H), 3.67 (s, 3H), 3.63–3.56 (m, 1H), 3.54 (d, *J* = 2.4 Hz, 1H), 3.51–3.38 (m, 1H), 2.00 (qd, *J* = 7.0, 2.4 Hz, 1H), 1.22 (s, 9H), 1.21 (s, 3H), 1.16 (t, *J* = 7.0 Hz, 3H), 1.15 (s, 3H), 0.95 (d, *J* = 6.9 Hz, 3H);

^{13}C NMR (100 MHz, $CDCl_3$) δ 178.6 (C), 177.6 (C), 83.0 (CH), 69.0 (CH_2), 68.7 (CH_2), 51.9 (CH_3), 48.5 (CH), 38.9 (C), 34.4 (C), 27.4 (CH_3), 21.8 (CH_3), 21.5 (CH_3), 15.7 (CH_3), 12.2 (CH_3);

IR (ATR) 2974, 1727, 1283, 1147, 1109 cm^{-1};

HRMS (ESI/TOF) m/z: [M + K]$^+$ Calcd for $C_{16}H_{30}KO_5$ 341.1730; Found 341.1731.

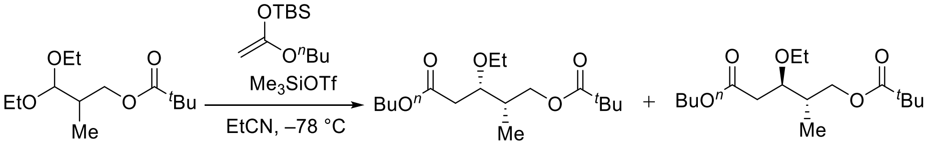

7a ***syn*-11a** ***anti*-11a**

Butyl (3*R,4*R**)-3-ethoxy-4-methyl-5-(pivaloyloxy)pentanoate (*syn*-11a) and butyl (3*R**,4*S**)-3-ethoxy-4-methyl-5-(pivaloyloxy)pentanoate (*anti*-11a)**. Following the general procedure for acetal substitution, acetal **7a** (0.28 g, 1.1 mmol) in EtCN (11 mL) was mixed with ((1-butoxyvinyl)oxy)(*tert*-butyl)dimethylsilane (1.0 g, 4.4 mmol) and trimethylsilyl trifluoromethanesulfonate (0.40 mL, 2.2 mmol) to provide an 89:11 mixture of diastereomers of ester **11a**. Ester **11a** was isolated as an 80:20 mixture of ***syn*-11a**:***anti*-11a** as a colorless oil (0.20 g, 63%):

^{1}H NMR (400 MHz, $CDCl_3$) δ 4.09–4.00 (m, 3.75H), 3.99–3.93 (m, 1H), 3.81 (ddd, *J* = 8.0, 5.3, 3.7 Hz, 1H), 3.72–3.61 (m, 0.75H), 3.58–3.50 (m, 1.25H), 3.48–3.41 (m, 1H), 2.81–2.72 (m, 0.25H), 2.64 (d, *J* = 6.0 Hz, 0.25H), 2.54 (dd, *J* = 15.0, 7.8 Hz, 1H), 2.47–2.45 (m, 0.25H), 2.41 (dd, *J* = 15.0, 5.3 Hz, 1H), 2.03–1.93 (m, 1H), 1.63–1.55 (m, 2.5H), 1.41–1.32 (m, 2.5H), 1.19 (d, *J* = 1.4 Hz, 11.25H), 1.14–1.10 (m, 3.75H), 0.93–0.89 (m, 7.5H);

Peaks attributed to major diastereomer (***syn*-11a**): ^{13}C NMR (100 MHz, $CDCl_3$) δ 178.5 (C), 172.1 (C), 76.4 (CH), 66.1 (CH_2), 66.0 (CH_2), 64.5 (CH_2), 38.9 (C), 37.8 (CH_2), 36.6 (CH), 30.8 (CH_2 overlapping with ***anti*-11a**, as determined by HSQC), 27.3 (CH_3), 19.23 (CH_2), 15.59 (CH_3), 13.8 (CH_3), 11.4 (CH_3);

Peaks attributed to minor diastereomer (***anti*-11a**): ^{13}C NMR (100 MHz, $CDCl_3$) δ 178.2 (C), 170.2 (C), 77.5 (CH), 65.7 (CH_2), 62.0 (CH_2), 60.7 (CH_2), 40.1 (CH_2), 39.3 (CH), 39.0 (C), 30.8 (CH_2 overlapping with ***syn*-11a**, as determined by HSQC), 27.2 (CH_3), 19.17 (CH_2), 15.57 (CH_3) 14.3 (CH_3), 13.1 (CH_3);

IR (ATR) 2965, 1730, 1282, 1152, 1091 cm^{-1};

HRMS (ESI/TOF) m/z: $[M + H]^+$ Calcd for $C_{17}H_{33}O_5$ 317.2323; Found 317.2332.

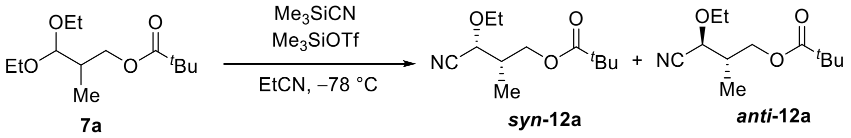

(2*R,3*S**)-3-Cyano-3-ethoxy-2-methylpropyl pivalate (*syn*-12a) and (2*R**,3*R**)-3-cyano-3-ethoxy-2-methylpropyl pivalate (*anti*-12a)**. Following the general procedure for acetal substitutions, acetal **7a** (0.12 g, 0.50 mmol) in EtCN (5 mL) were mixed with trimethylsilyl cyanide (0.25 mL, 2.0 mmol) and trimethylsilyl trifluoromethanesulfonate (0.18 mL, 1.0 mmol) to provide a 91:9 mixture of diastereomers of ester **12a**. Ester **12a** was isolated as a 91:9 mixture of ***syn*-12a**:***anti*-12a** as a colorless oil (0.098 g, 86%):

^{1}H NMR (400 MHz, $CDCl_3$) δ 4.18 (d, *J* = 4.4 Hz, 1H), 4.16 (d, *J* = 5.0 Hz, 0.1H), 4.14–4.11 (m, 0.2H), 4.09 (dd, *J* = 6.4, 3.6 Hz, 2H), 3.91–3.77 (m, 1.1H), 3.50 (dq, *J* = 9.1, 6.9 Hz, 1.1H), 2.34–2.20 (m, 1.1H), 1.25 (td, *J* = 7.0, 2.5 Hz, 3.3H), 1.21 (s, 9.9H), 1.15 (d, *J* = 7.0, 0.3H), 1.13 (d, *J* = 7.0, 3.0H);

Peaks attributed to major diastereomer (***syn*-12a**): ^{13}C NMR (100 MHz, $CDCl_3$) δ 178.2 (C overlapping with ***anti*-12a**), 117.9 (C), 69.8 (CH), 66.9 (CH_2), 64.43 (CH_2), 39.0 (C), 36.9 (CH), 27.3 (CH_3 overlapping with ***anti*-12a**, as determined by HSQC), 14.77 (CH_3), 12.1 (CH_3);

Peaks attributed to minor diastereomer (***anti*-12a**): ^{13}C NMR (100 MHz, $CDCl_3$) δ 178.2 (C overlapping with ***syn*-12a**), 117.5 (C), 70.9 (CH), 66.8 (CH_2), 64.41 (CH_2), 39.1 (C), 36.8 (CH), 27.3 (CH_3 overlapping with ***syn*-12a**, as determined by HSQC), 14.81 (CH_3), 12.9 (CH_3);

IR (ATR) 2976, 1728, 1282, 1102, 1035 cm^{-1};

HRMS (ESI/TOF) m/z: $[M + Na]^+$ Calcd for $C_{12}H_{21}NNaO_3$ 250.1419; Found 250.1417.

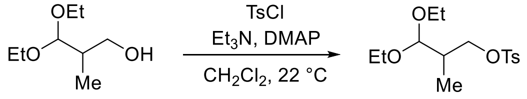

S9 **20**

3,3-Diethoxy-2-methylpropyl 4-methylbenzenesulfonate (20). Alcohol **S9** was prepared according to a literature procedure (1.5 g, 40% over 4 steps).[4] To a cooled (0 °C) solution of alcohol **S9** (0.34 g, 2.3 mmol) in CH_2Cl_2 (15 mL) were added triethylamine (0.45 mL, 3.2 mmol), 4-dimethylaminopyridine (0.018 g, 0.15 mmol), and *p*-toluenesulfonyl chloride (0.50 g, 2.6 mmol). After stirring at 22 °C for 4 h, the mixture was diluted with CH_2Cl_2 (20 mL). The resulting mixture was washed with H_2O (10 mL), brine (10 mL) and dried over anhydrous Na_2SO_4. The mixture was filtered, concentrated *in vacuo*, and purified by flash column chromatography (20:80 EtOAc:hexanes) to provide acetal **20** as a colorless oil (0.40 g, 56%):

^1H NMR (400 MHz, $CDCl_3$) δ 7.79 (d, J = 8.0 Hz, 2H), 7.34 (d, J = 8.0 Hz, 2H), 4.30 (d, J = 6.1 Hz, 1H), 4.05 (dd, J = 9.4, 5.1 Hz, 1H), 3.96 (dd, J = 9.4, 6.1 Hz, 1H), 3.66–3.58 (m, 2H), 3.46–3.36 (m, 2H), 2.45 (s, 3H), 2.06 (qtd, J = 7.0, 6.1, 5.1 Hz, 1H), 1.14 (t, J = 7.0 Hz, 3H), 1.12 (t, J = 7.0 Hz, 3H), 0.95 (d, J = 7.0 Hz, 3H);

^{13}C NMR (100 MHz, $CDCl_3$) δ 144.8 (C), 133.1 (C), 129.9 (CH), 128.1 (CH), 103.6 (CH), 72.3 (CH_2), 63.5 (CH_2), 63.0 (CH_2), 37.2 (CH), 21.8 (CH_3), 15.4 (CH_3), 15.3 (CH_3), 11.8 (CH_3);

IR (ATR) 2975, 2882, 1174, 1057, 963 cm^{-1};

HRMS (ESI/TOF) m/z: $[M + Na]^+$ Calcd for $C_{15}H_{24}NaO_5S$ 339.1237; Found 339.1248.

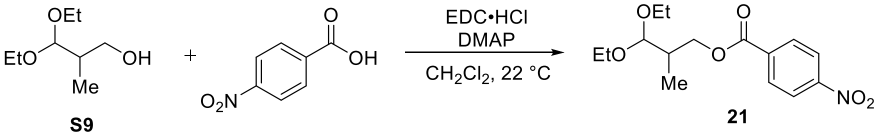

S9 **21**

3,3-Diethoxy-2-methylpropyl 4-nitrobenzoate (21). To a cooled (0 °C) solution of 4-nitrobenzoic acid (0.14 g, 0.83 mmol) in CH_2Cl_2 (3.4 mL) were added *N*-(3-Dimethylaminopropyl)-*N′*-ethylcarbodiimide hydrochloride (0.17 g, 0.9 mmol) and 4-dimethylaminopyridine (0.16 g, 1.3 mmol). The reaction mixture was stirred for 30 min at 22 °C. A solution of alcohol **S9** (0.1 g, 0.7 mmol) in CH_2Cl_2 (1.0 mL) was added dropwise and the resulting mixture was stirred for an additional 2.5 h at 22 °C. The mixture was then diluted with CH_2Cl_2 (10 mL), filtered, and washed with saturated aqueous $NaHCO_3$ (2 × 10 mL), H_2O (2 × 10 mL) and brine (2 × 10 mL). The organic layer was then dried over anhydrous Na_2SO_4, filtered, and concentrated *in vacuo*. The crude

mixture was purified by flash column chromatography (10:90 EtOAc:hexanes) to provide ester **21** as a colorless oil (0.17 g, 81%):

^{1}H NMR (400 MHz, $CDCl_3$) δ 8.31–8.27 (m, 2H), 8.22–8.18 (m, 2H), 4.47 (dd, *J* = 10.9, 5.0 Hz, 1H), 4.42 (d, *J* = 6.2 Hz, 1H), 4.29 (dd, *J* = 10.9, 6.6 Hz, 1H), 3.76–3.67 (m, 2H), 3.58–3.47 (m, 2H), 2.33–2.23 (m, 1H), 1.22 (t, *J* = 7.0 Hz, 3H), 1.20 (t, *J* = 7.0 Hz, 3H), 1.09 (d, *J* = 6.9 Hz, 3H);

^{13}C NMR (100 MHz, CDCl3) δ 164.8 (C), 150.7 (C), 136.0 (C), 130.8 (CH), 123.7 (CH), 104.3 (CH), 67.6 (CH_2), 63.1 (CH_2), 62.5 (CH_2), 36.8 (CH), 15.5 (CH_3), 15.4 (CH_3), 12.6 (CH_3);

IR (ATR) 2975, 2880, 1723, 1527, 1272 cm^{-1};

HRMS (ESI/TOF) m/z: $[M + Na]^+$ Calcd for $C_{15}H_{21}NNaO_6$ 334.1261; Found 334.1272.

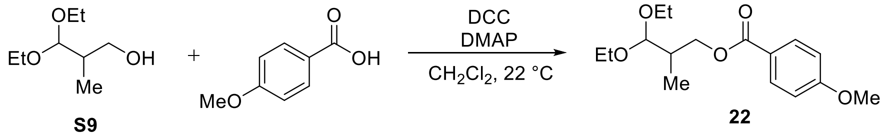

3,3-Diethoxy-2-methylpropyl 4-methoxybenzoate (22). To a cooled (0 °C) solution of 4-methoxybenzoic acid (0.092 mL, 0.83 mmol) in CH_2Cl_2 (3.4 mL) were added *N,N′*-dicyclohexylcarbodiimide (0.19 g, 0.90 mmol) and 4-dimethylaminopyridine (0.16 g, 1.3 mmol). The reaction mixture was stirred for 30 min at 22 °C. A solution of alcohol **S9** (0.10 g, 0.70 mmol) in CH_2Cl_2 (1.0 mL) was added dropwise and the resulting mixture was stirred for an additional 5 h at 22 °C. The mixture was then diluted with CH_2Cl_2 (10 mL), filtered, and washed with saturated aqueous $NaHCO_3$ (2 × 10 mL), H_2O (2 × 10 mL) and brine (2 × 10 mL). The organic layer was then dried over anhydrous Na_2SO_4, filtered, and concentrated *in vacuo*. The crude mixture was purified by flash column chromatography (10:90 EtOAc:hexanes) to provide ester **22** as a colorless oil (0.14 g, 71%):

^{1}H NMR (400 MHz, $CDCl_3$) δ 7.99 (d, *J* = 8.9 Hz, 2H), 6.92 (d, *J* = 8.9 Hz, 2H), 4.44 (d, *J* = 6.4 Hz, 1H), 4.36 (dd, *J* = 10.9, 5.0 Hz, 1H), 4.21 (dd, *J* = 10.9, 6.4 Hz, 1H), 3.86 (s, 3H), 3.75–3.67 (m, 2H), 3.58–3.48 (m, 2H), 2.25 (quind, *J* = 6.4, 5.0 Hz, 1H), 1.22 (t, *J* = 7.1 Hz, 3H), 1.20 (t, *J* = 7.1 Hz, 3H), 1.07 (d, *J* = 6.4 Hz, 3H);

^{13}C NMR (100 MHz, $CDCl_3$) δ 166.3 (C), 163.3 (C), 131.5 (CH), 122.9 (C), 113.6 (CH), 104.2 (CH), 66.3 (CH_2), 62.8 (CH_2), 62.3 (CH_2), 55.4 (CH_3), 36.8 (CH), 15.33 (CH_3), 15.31 (CH_3), 12.3 (CH_3);

IR (ATR) 2974, 1710, 1605, 1511, 1098 cm^{-1};

HRMS (ESI/TOF) m/z: $[M + K]^+$ Calcd for $C_{16}H_{24}KO_5$ 335.1255; Found 335.1240.

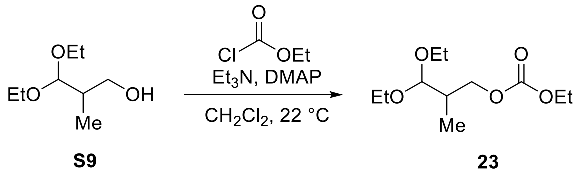

3,3-Diethoxy-2-methylpropyl ethyl carbonate (23). To a cooled (0 °C) solution of alcohol **S9** (0.38 g, 2.6 mmol) in CH_2Cl_2 (15 mL) were added triethylamine (0.90 mL, 6.4 mmol), 4-dimethylaminopyridine (0.031 g, 0.26 mmol), and ethyl chloroformate (0.48 mL, 5.1 mmol). The reaction mixture was warmed to 22 °C. After 4 h, CH_2Cl_2 (20 mL) was added and the mixture was washed with H_2O (10 mL) and brine (10 mL) and dried over anhydrous Na_2SO_4. The resulting mixture was filtered, concentrated *in vacuo,* and purified by flash column chromatography (20:80 EtOAc:hexanes) to provide acetal **23** as a colorless oil (0.16 g, 27%):

^1H NMR (400 MHz, $CDCl_3$) δ 4.36 (d, *J* = 6.2 Hz, 1H), 4.23–4.16 (m, 3H), 4.05 (dd, *J* = 10.5, 6.6 Hz, 1H), 3.68 (dq, *J* = 9.3, 7.0 Hz, 2H), 3.55–3.44 (m, 2H), 2.19–2.07 (m, 1H), 1.31 (t, *J* = 7.1 Hz, 3H), 1.20 (t, *J* = 7.1 Hz, 3H), 1.19 (t, *J* = 7.0 Hz, 3H), 1.00 (d, *J* = 6.8 Hz, 3H);

^{13}C NMR (100 MHz, $CDCl_3$) δ 155.4 (C), 104.0 (CH), 69.6 (CH_2), 64.0 (CH_2), 63.0 (CH_2), 62.8 (CH_2), 36.9 (CH), 15.4 (two CH_3 overlapping, as determined by HSQC), 14.4 (CH_3), 12.1 (CH_3);

IR (ATR) 2977, 2881, 1744, 1251, 1058 cm^{-1};

HRMS (ESI/TOF) m/z: $[M + Na]^+$ Calcd for $C_{11}H_{22}NaO_5$ 257.1359; Found 257.1361.

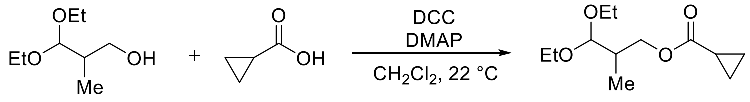

S9 **24**

3,3-Diethoxy-2-methylpropyl cyclopropanecarboxylate (24). To a cooled (0 °C) solution of cyclopropanecarboxylic acid (1.0 mL, 13 mmol) in CH_2Cl_2 (50 mL) were added *N,N'*-dicyclohexylcarbodiimide (2.8 g, 14 mmol) and 4-dimethylaminopyridine (2.5 g, 20 mmol). The reaction mixture was stirred for 30 min at 22 °C. A solution of alcohol **S9** (1.6 g, 10 mmol) in CH_2Cl_2 (1.0 mL) was added dropwise and the resulting mixture was stirred for an additional 5 h at 22 °C. The mixture was then diluted with CH_2Cl_2 (20 mL), filtered, and washed with saturated aqueous $NaHCO_3$ (2 × 10 mL), H_2O (2 × 10 mL), and brine (2 × 10 mL). The organic layer was then dried over anhydrous Na_2SO_4, filtered, and concentrated *in vacuo*. The crude mixture was purified by flash column chromatography (10:90 EtOAc:hexanes) to provide ester **24** as a colorless oil (1.4 g, 59%):

^1H NMR (400 MHz, $CDCl_3$) δ 4.34 (d, *J* = 6.6 Hz, 1H), 4.14 (dd, *J* = 10.8, 5.1 Hz, 1H), 3.98 (dd, *J* = 10.8, 6.6 Hz, 1H), 3.68 (dq, *J* = 9.4, 7.0 Hz, 2H), 3.50 (dqd, *J* = 9.3, 7.0, 5.8 Hz, 2H), 2.10 (sxtd, *J* = 6.6, 5.1 Hz, 1H), 1.60 (tt, *J* = 8.0, 4.7 Hz, 1H), 1.21 (t, *J* = 7.0 Hz, 3H), 1.20 (t, *J* = 7.0 Hz, 3H), 1.01–0.96 (m, 5H), 0.87–0.81 (m, 2H);

^{13}C NMR (100 MHz, $CDCl_3$) δ 175.0 (C), 104.2 (CH), 66.2 (CH_2), 62.8 (CH_2), 62.5 (CH_2), 36.7 (CH), 15.42 (CH_3), 15.41 (CH_3), 13.0 (CH), 12.3 (CH_3), 8.4 (two CH_2 overlapping, as determined by HSQC);

IR (ATR) 2975, 2880, 1726, 1172, 1057 cm^{-1};

HRMS (ESI/TOF) m/z: $[M + Na]^+$ Calcd for $C_{12}H_{22}NaO_4$ 253.1410; Found 253.1407.

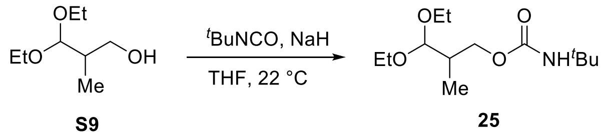

3,3-Diethoxy-2-methylpropyl *tert*-butylcarbamate (25). Following the general procedure for carbamate formation, alcohol **S9** (0.18 g, 1.2 mmol) in THF (12 mL) was mixed with NaH (0.10 g, 60% dispersion in mineral oil, 2.4 mmol) and *tert*-butyl isocyanate (0.20 mL, 1.8 mmol) to provide carbamate **25** as a colorless oil (0.28 g, 89%):

^{1}H NMR (400 MHz, $CDCl_3$) δ 4.59 (s, 1H), 4.32 (d, *J* = 6.4 Hz, 1H), 4.10–4.06 (m, 1H), 3.93–3.98 (m, 1H), 3.71–3.63 (m, 2H), 3.54–3.45 (m, 2H), 2.10–2.03 (m, 1H), 1.31 (s, 9H), 1.20 (t, *J* = 7.0 Hz, 6H), 0.97 (d, *J* = 6.9 Hz, 3H);

^{13}C NMR (100 MHz, $CDCl_3$) δ 155.2 (C), 104.2 (CH), 65.9 (CH_2), 62.7 (CH_2), 62.5 (CH_2), 50.4 (C), 37.0 (CH), 29.1 (CH_3), 15.4 (two CH_3 overlapping, as determined by HSQC), 12.2 (CH_3);

IR (ATR) 3346 (br), 2972, 1702, 1523, 1266 cm^{-1};

HRMS (ESI/TOF) m/z: [M + Na]$^+$ Calcd for $C_{13}H_{27}NNaO_4$ 284.1832; Found 284.1843.

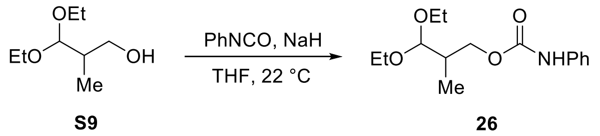

3,3-Diethoxy-2-methylpropyl phenylcarbamate (26). Following the general procedure for carbamate formation, alcohol **S9** (0.10 g, 0.70 mmol) in THF (7.0 mL) was mixed with NaH (0.054 g, 60% dispersion in mineral oil, 1.3 mmol) and phenyl isocyanate (0.11 mL, 1.0 mmol) to provide carbamate **26** as a colorless oil (0.13 g, 69%):

^{1}H NMR (400 MHz, $CDCl_3$) δ 7.39–7.37 (m, 2H), 7.32–7.27 (m, 2H), 7.11–7.00 (m, 1H), 6.71 (s, 1H), 4.36 (d, *J* = 6.6 Hz, 1H), 4.26 (dd, *J* = 10.7, 5.2 Hz, 1H), 4.07 (dd, *J* = 10.7, 6.6 Hz, 1H), 3.74–3.64 (m, 2H), 3.55–3.46 (m, 2H), 2.20–2.10 (m, 1H), 1.21 (t, *J* = 7.1 Hz, 3H), 1.20 (t, *J* = 7.0 Hz, 3H), 1.02 (d, *J* = 6.8 Hz, 3H);

^{13}C NMR (100 MHz, $CDCl_3$) δ 153.7 (C), 138.1 (C), 129.1 (CH), 123.5 (CH), 118.8 (CH), 104.2 (CH), 67.0 (CH_2), 62.8 (CH_2), 62.5 (CH_2), 36.9 (CH), 15.4 (two CH_3 overlapping, as determined by HSQC), 12.2 (CH_3);

IR (ATR) 3314 (br), 2975, 1707, 1531, 1218 cm^{-1};

HRMS (ESI/TOF) m/z: [M + Na]$^+$ Calcd for $C_{15}H_{23}NNaO_4$ 304.1519; Found 304.1525.

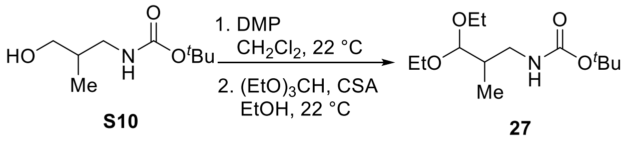

***N*-(3,3-diethoxy-2-methylpropyl)pivalamide (27).** Hydroxy carbamate **S10** was prepared according to a literature procedure (0.35 g, 19% after two steps).[5] To a solution of hydroxy carbamate **S10** (0.19 g, 1.0 mmol) in CH_2Cl_2 (10 mL) was added Dess–Martin periodinane (0.55 g, 1.3 mmol). The mixture was stirred at 22 °C and diluted with CH_2Cl_2 (5.0 mL). The mixture was washed with saturated aqueous $Na_2S_2O_3$ (2 × 10 mL) and saturated aqueous $NaHCO_3$ (2 × 10 mL) and dried over anhydrous Na_2SO_4. The resulting mixture was filtered and concentrated *in vacuo* to provide the corresponding aldehyde, which was used directly in the next step without further purification.

Following the general procedure for diethyl acetal formation, the crude aldehyde (0.12 g, 0.70 mmol) in triethyl orthoformate (1.2 mL) was mixed with ethanol (0.12 mL) and camphorsulfonic acid (0.81 g, 0.35 mmol) to provide acetal **27** as a colorless oil (0.090 g, 37% after two steps):

^{1}H NMR (400 MHz, $CDCl_3$) δ 5.08 (s, 1H), 4.22 (d, *J* = 5.8 Hz, 1H), 3.76–3.57 (m, 2H), 3.51–3.42 (m, 2H), 3.13–3.08 (m, 2H), 1.94–1.87 (m, 1H), 1.40 (s, 9H), 1.19 (t, *J* = 7.1 Hz, 3H), 1.18 (t, *J* = 7.1 Hz, 3H), 0.90 (d, *J* = 7.0 Hz, 3H);

^{13}C NMR (100 MHz, $CDCl_3$) δ 156.2 (C), 106.5 (CH), 78.8 (C), 63.3 (CH_2), 62.2 (CH_2), 42.8 (CH_2), 36.9 (CH), 28.5 (CH_3), 15.4 (CH_3), 15.3 (CH_3), 13.5 (CH_3);

IR (ATR) 3357 (br), 2975, 1693, 1513, 1168 cm^{-1};

HRMS (ESI/TOF) m/z: $[M + Na]^+$ Calcd for $C_{13}H_{27}NNaO_4$ 284.1832; Found 284.1835.

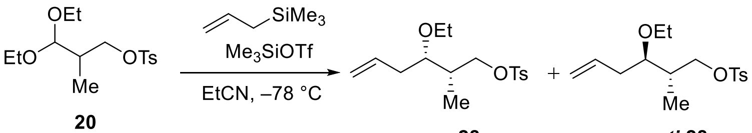

(2*R,3*R**)-3-Ethoxy-2-methylhex-5-en-1-yl 4-methylbenzenesulfonate (*syn*-28) and (2*R**,3*S**)-3-ethoxy-2-methylhex-5-en-1-yl 4-methylbenzenesulfonate (*anti*-28).** Following the general procedure for acetal substitution, acetal **20** (0.068 g, 0.21 mmol) in EtCN (2.1 mL) was mixed with allyltrimethylsilane (0.13 mL, 0.84 mmol) and trimethylsilyl trifluoromethanesulfonate (0.076 mL, 0.42 mmol) to provide a 52:48 mixture of diastereomers of ester **28**. Ester **28** was isolated as a 53:47 mixture of ***syn*-28**:***anti*-28** as a colorless oil (0.056 g, 85%):

^{1}H NMR (400 MHz, $CDCl_3$) δ 7.80–7.78 (m, 4H), 7.38–7.32 (m, 4H), 5.8 –5.65 (m, 2H), 5.09–4.99 (m, 4H), 4.09 (dd, *J* = 9.2, 5.6 Hz, 1H), 4.05–4.00 (m, 2H), 3.88 (dd, *J* = 9.3, 6.1 Hz, 1H), 3.52 (dqd, *J* = 9.2, 7.0, 0.9 Hz, 2H), 3.33 (td, *J* = 6.6, 3.5 Hz, 1H), 3.30–3.16 (m, 3H), 2.45 (s, 6H), 2.35–2.24 (m, 2H), 2.18–2.09 (m, 2H), 2.02–1.86 (m, 2H), 1.05 (t, *J* = 7.0 Hz, 6H), 0.91 (d, *J* = 7.0 Hz, 3H), 0.86 (d, *J* = 7.0 Hz, 3H);

^{13}C NMR (100 MHz, $CDCl_3$) δ 144.82 (C), 144.75 (C), 134.8 (CH), 134.2 (CH), 133.194 (C), 133.187 (C), 129.94 (CH), 129.90 (CH), 128.1 (two CH overlapping, as determined by HSQC), 117.5 (CH_2), 117.2 (CH_2), 79.1 (CH), 77.9 (CH), 72.7 (CH_2), 72.6 (CH_2), 65.7 (CH_2), 65.3 (CH_2), 36.4 (CH), 36.0 (CH), 35.9 (CH_2), 35.1 (CH_2), 21.7 (two CH_3 overlapping, as determined by HSQC), 15.54 (CH_3), 15.45 (CH_3), 13.4 (CH_3), 10.5 (CH_3);

IR (ATR) 2975, 1358, 1175, 1096, 963 cm^{-1};

HRMS (ESI/TOF) m/z: $[M + H]^+$ Calcd for $C_{16}H_{25}O_4S$ 313.1468; Found 313.1477.

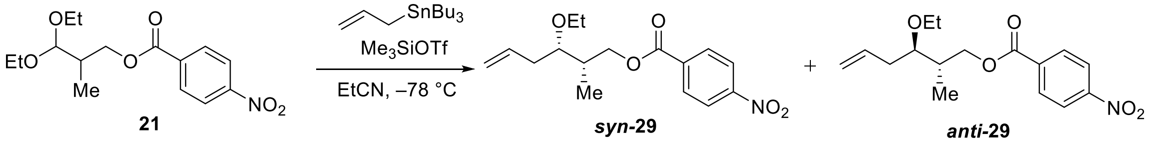

(2*R,3*R**)-3-Ethoxy-2-methylhex-5-en-1-yl 4-nitrobenzoate (*syn*-29) and (2*R**,3*S**)-3-ethoxy-2-methylhex-5-en-1-yl 4-nitrobenzoate (*anti*-29).** Following the general procedure for acetal substitution, acetal **21** (0.040 g, 0.13 mmol) in EtCN (1.3 mL) was mixed with allyltributylstannane (0.16 mL, 0.52 mmol) and trimethylsilyl trifluoromethanesulfonate (0.047 mL, 0.26 mmol) to provide a 72:28 mixture of diastereomers of ester **29**. Ester **29** was isolated as a 68:32 mixture of ***syn*-29**:***anti*-29** with some stannane impurities as a colorless oil (0.035 g, 93%):

^{1}H NMR (400 MHz, $CDCl_3$) δ 8.30 (dd, *J* = 8.8, 1.6 Hz, 3H), 8.23–8.18 (m, 3H), 5.97–5.71 (m, 1.5H), 5.17–5.03 (m, 3H), 4.48 (dd, *J* = 10.8, 4.5 Hz, 0.5H), 4.41–4.33 (m, 1.5H), 4.28 (dd, *J* = 10.8, 6.8 Hz, 1H), 3.66–3.58 (m, 1.5H), 3.48–3.38 (m, 2.5H), 3.32–3.28 (m, 0.5H), 2.47–2.36 (m, 1.5H), 2.33–2.24 (m, 1.5H), 2.19–2.11 (m, 1.5H), 1.17 (t, *J* = 7.0 Hz, 1.5H), 1.16 (t, *J* = 7.0 Hz, 3H), 1.05 (d, *J* = 7.4 Hz, 1.5H), 1.04 (d, *J* = 7.1 Hz, 3H);

Peaks attributed to major diastereomer (***syn*-29**): ^{13}C NMR (100 MHz, $CDCl_3$) δ 164.8 (C), 150.7 (C overlapping with ***anti*-29**), 135.95 (C), 135.1 (CH), 130.78 (CH), 123.7 (CH overlapping with ***anti*-29** as determined by HSQC), 117.3 (CH_2), 79.4 (CH), 68.4 (CH_2), 65.8 (CH_2), 36.1 (CH_2), 35.9 (CH), 15.7 (CH_3), 11.4 (CH_3);

Peaks attributed to minor diastereomer (***anti*-29**): ^{13}C NMR (100 MHz, $CDCl_3$) δ 164.9 (C), 150.7 (C overlapping with ***syn*-29**), 136.01 (C), 134.5 (CH), 130.76 (CH), 123.7 (CH overlapping with ***syn*-29** as determined by HSQC), 117.5 (CH_2), 80.3 (CH), 68.1 (CH_2), 65.5 (CH_2), 36.3 (CH_2), 35.5 (CH), 15.6 (CH_3), 14.1 (CH_3);

IR (ATR) 2973, 1724, 1528, 1270, 1100 cm^{-1};

HRMS (ESI/TOF) m/z: $[M + H]^+$ Calcd for $C_{16}H_{22}NO_5$ 308.1492; Found 308.1507.

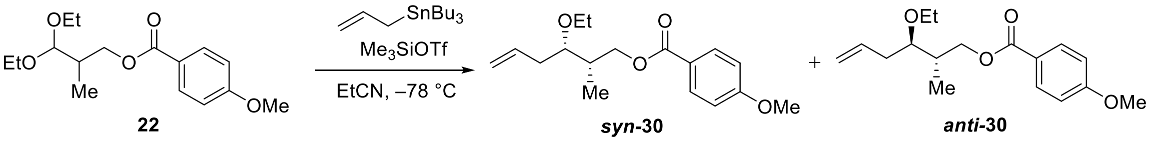

(2*R,3*R**)-3-Ethoxy-2-methylhex-5-en-1-yl 4-methoxybenzoate (*syn*-30) and (2*R**,3*S**)-3-ethoxy-2-methylhex-5-en-1-yl 4-methoxybenzoate (*anti*-30).** Following the general procedure for acetal substitution, acetal **22** (0.027 g, 0.090 mmol) in EtCN (1.0 mL) was mixed with allyltributylstannane (0.12 mL, 0.36 mmol) and trimethylsilyl trifluoromethanesulfonate (0.033 mL, 0.18 mmol) to provide a 75:25 mixture of diastereomers of ester **30**. Ester **30** was isolated as a 70:30 mixture of ***syn*-30**:***anti*-30** with some stannane impurities as a colorless oil (0.025 g, 95%):

^{1}H NMR (400 MHz, $CDCl_3$) δ 8.03–7.95 (m, 2.8H), 6.97–6.88 (m, 2.8H), 5.96–5.76 (m, 1.4H), 5.15–5.02 (m, 2.8H), 4.36 (dd, *J* = 10.8, 4.7 Hz, 0.4H), 4.31–4.25 (m, 1.4H), 4.20 (dd, *J* = 10.8, 6.5 Hz, 1H), 3.86 (s, 4.2H), 3.65–3.56 (m, 1.4H), 3.51–3.39 (m, 2.4H), 3.31 (td, *J* = 6.6, 4.5 Hz, 0.4H), 2.45–2.35 (m, 1.3H), 2.32–2.22 (m, 1.4H), 2.17–2.07 (m, 1.4H), 1.17 (t, *J* = 7.0 Hz, 4.2H), 1.03 (d, *J* = 6.9 Hz, 1.2H), 1.02 (d, *J* = 7.0 Hz, 3H);

Peaks attributed to major diastereomer (***syn*-30**): ^{13}C NMR (100 MHz, $CDCl_3$) δ 166.45 (C), 163.5 (C), 135.4 (CH), 131.66 (CH), 123.0 (C), 117.0 (CH_2), 113.76 (CH), 79.4 (CH), 67.1 (CH_2), 65.9 (CH_2), 55.5 (CH_3 overlapping with ***anti*-30** as determined by HSQC), 36.36 (CH), 36.1 (CH_2), 15.7 (CH_3), 11.5 (CH_3);

Peaks attributed to minor diastereomer (***anti*-30**): ^{13}C NMR (100 MHz, $CDCl_3$) δ 166.53 (C), 163.4 (C), 135.0 (CH), 131.65 (CH), 123.1 (C), 117.2 (CH_2), 113.75 (CH), 80.4 (CH), 66.8 (CH_2), 65.5 (CH_2), 55.5 (CH_3 overlapping with ***syn*-30** as determined by HSQC), 36.39 (CH), 35.6 (CH_2), 15.6 (CH_3), 13.8 (CH_3);

IR (ATR) 2970, 1711, 1605, 1253, 1166 cm^{-1};

HRMS (ESI/TOF) m/z: [M + Na]$^+$ Calcd for $C_{17}H_{24}NaO_4$ 315.1567; Found 315.1552.

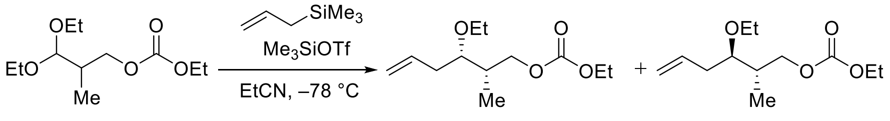

23 *syn*-**31** *anti*-**31**

(2*R,3*R**)-3-Ethoxy-2-methylhex-5-en-1-yl ethyl carbonate (*syn*-31) and (2*R**,3*S**)-3-ethoxy-2-methylhex-5-en-1-yl ethyl carbonate (*anti*-31).** Following the general procedure for acetal substitution, acetal **23** (0.050 g, 0.21 mmol) in EtCN (2.1 mL) was mixed with allyltrimethylsilane (0.13 mL, 0.84 mmol) and trimethylsilyl trifluoromethanesulfonate (0.076 mL, 0.42 mmol) to provide a 53:47 mixture of diastereomers of ester **31**. Ester **31** was isolated as a 59:41 mixture of ***syn*-31**:***anti*-31** as a colorless oil (0.042 g, 87%):

^{1}H NMR (400 MHz, $CDCl_3$) δ 5.90–5.71 (m, 1.7H), 5.13–4.99 (m, 3.4H), 4.23–4.15 (m, 4.1 H), 4.14–4.07 (m, 1.7H), 4.01 (dd, *J* = 10.4, 6.5 Hz, 1H), 3.62–3.53 (m, 1.7H), 3.45–3.33 (m, 2.7H), 3.27–3.20 (m, 0.7H), 2.37–2.31 (m, 1.7H), 2.26–2.16 (m, 1.7H), 2.02–1.96 (m, 1.7H), 1.30 (d, *J* = 7.1 Hz, 3H), 1.30 (d, *J* = 7.2 Hz, 2.1H), 1.15 (t, *J* = 7.0 Hz, 2.1H), 1.14 (t, *J* = 7.0 Hz, 3H), 0.96 (d, *J* = 7.0 Hz, 2.1H), 0.93 (d, *J* = 7.0 Hz, 3H);

Peaks attributed to major diastereomer (***syn*-31**): ^{13}C NMR (100 MHz, $CDCl_3$) δ 155.4 (C), 135.2 (CH), 117.0 (CH_2), 78.8 (CH), 70.3 (CH_2), 65.8 (CH_2), 63.98 (CH_2), 36.3 (CH overlapping with ***anti*-31** as determined by HSQC), 35.9 (CH_2), 15.63 (CH_3), 14.4 (CH_3), 11.0 (CH_3);

Peaks attributed to minor diastereomer (***anti*-31**): ^{13}C NMR (100 MHz, $CDCl_3$) δ 155.5 (C), 134.8 (CH), 117.2 (CH_2), 79.9 (CH), 70.0 (CH_2), 65.4 (CH_2), 63.95 (CH_2), 36.3 (CH overlapping with ***syn*-31** as determined by HSQC), 35.4 (CH_2), 15.57 (CH_3), 14.2 (CH_3), 13.6 (CH_3);

IR (ATR) 2976, 1743, 1250, 1086, 1001 cm^{-1};

HRMS (ESI/TOF) m/z: $[M + Na]^+$ Calcd for $C_{12}H_{22}NaO_4$ 253.1410; Found 253.1412.

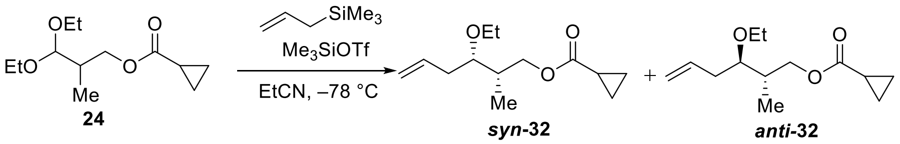

(2*R,3*R**)-3-Ethoxy-2-methylhex-5-en-1-yl cyclopropanecarboxylate (*syn*-32) and (2*R**,3*S**)-3-ethoxy-2-methylhex-5-en-1-yl cyclopropanecarboxylate (*anti*-32)**. Following the general procedure for acetal substitution, acetal **24** (0.23 g, 1.0 mmol) in EtCN (10 mL) was mixed with allyltrimethylsilane (0.64 mL, 4.0 mmol) and trimethylsilyl trifluoromethanesulfonate (0.36 mL, 2.0 mmol) to provide a 58:42 mixture of diastereomers of ester **32**. Ester **32** was isolated as a 59:41 mixture of ***syn*-32**:***anti*-32** (0.18 g, 80%) as a colorless oil:

^1H NMR (400 MHz, $CDCl_3$) δ 5.94–5.74 (m, 1.7H), 5.14–5.01 (m, 3.4H), 4.15 (dd, *J* = 10.8, 4.7 Hz, 0.7H), 4.09–4.02 (m, 1.7H), 3.97 (dd, *J* = 10.8, 6.6 Hz, 1H), 3.62–3.53 (m, 1.7H), 3.46–3.38 (m, 1.7H), 3.33 (td, *J* = 6.5, 3.9 Hz, 1H), 3.22 (td, *J* = 6.6, 4.5 Hz, 0.7H), 2.38–2.30 (m, 1.7H), 2.27–2.18 (m, 1.7H), 2.05–1.91 (m, 1.7H), 1.64–1.57 (m, 1.7H), 1.17 (t, *J* = 7.0 Hz, 2.1H), 1.16 (t, *J* = 7.0 Hz, 3H), 1.01–0.96 (m, 3.4H), 0.94 (d, *J* = 6.8 Hz, 2.1H), 0.93 (d, *J* = 6.9 Hz, 3H), 0.87–0.81 (m, 3.4H);

Peaks attributed to major diastereomer (***syn*-32**): ^{13}C NMR (100 MHz, $CDCl_3$) δ 175.0 (C), 135.0 (CH), 117.0 (CH_2), 79.3 (CH), 66.9 (CH_2), 65.8 (CH_2), 36.3 (CH_2), 35.9 (CH), 15.7 (CH_3), 13.1 (CH overlapping with ***anti*-32**), 11.3 (CH_3), 8.44 (two CH_2 overlapping, as determined by HSQC);

Peaks attributed to minor diastereomer (***anti*-32**): ^{13}C NMR (100 MHz, $CDCl_3$) δ 175.1 (C), 135.4 (CH), 117.1 (CH_2), 80.3 (CH), 66.6 (CH_2), 65.4 (CH_2), 36.2 (CH_2), 35.5 (CH), 15.6 (CH_3), 13.6 (CH_3), 13.1 (CH overlapping with ***syn*-32**), 8.40 (two CH_2 overlapping, as determined by HSQC);

IR (ATR) 2975, 1726, 1173, 1073, 911 cm^{-1};

HRMS (ESI/TOF) m/z: $[M + Na]^+$ Calcd for $C_{13}H_{22}NaO_3$ 249.1461; Found 249.1466.

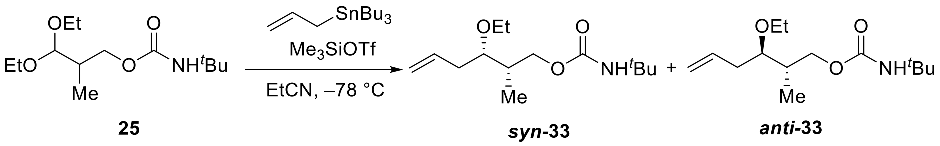

(2*R,3*R**)-3-Ethoxy-2-methylhex-5-en-1-yl *tert*-butylcarbamate (*syn*-33) and (2*R**,3*S**)-3-ethoxy-2-methylhex-5-en-1-yl *tert*-butylcarbamate (*anti*-33)**. Following the general procedure for acetal substitution, acetal **25** (0.031 g, 0.12 mmol) in EtCN (1.2 mL) was mixed with allyltributylstannane (0.15 mL, 0.48 mmol) and trimethylsilyl trifluoromethanesulfonate (0.043 mL, 0.24 mmol) to provide an 84:16 mixture of diastereomers of ester **33**. Ester **33** was isolated as an 85:15 mixture of ***syn*-33**:***anti*-33** (0.031 g, quantitative) with some stannane impurities as a colorless oil. Characterization was done on a 63:37 mixture of ***syn*-33**:***anti*-33**, which was isolated from the reaction with allyltrimethylsilane:

^{1}H NMR (400 MHz, $CDCl_3$) δ 5.94–5.72 (m, 1.6H), 5.14–4.99 (m, 3.2H), 4.59 (s, 1.6H), 4.11–3.88 (m, 3.2H), 3.56–3.51 (m, 1.6H), 3.46–3.37 (m, 1.6H), 3.31 (td, *J* = 6.4, 3.8 Hz, 1H), 3.20 (td, *J* = 6.7, 4.4 Hz, 0.6H), 2.38–2.28 (m, 1.6H), 2.25–2.16 (m, 1.6H), 2.01–1.86 (m, 1.6H), 1.31 (s, 14.4H), 1.16 (t, *J* = 7.0 Hz, 1.8H), 1.15 (t, *J* = 7.0 Hz, 3H), 0.92 (d, *J* = 6.9 Hz, 4.8H);

Peaks attributed to major diastereomer (***syn*-33**): ^{13}C NMR (100 MHz, $CDCl_3$) δ 155.2 (C), 135.3 (CH), 116.75 (CH_2), 79.3 (CH), 66.5 (CH_2), 65.7 (CH_2), 50.3 (C), 36.3 (CH overlapping with ***anti*-33**, as determined by HSQC), 36.2 (CH_2), 29.0 (CH_3, overlapping with ***anti*-33**, as determined by HSQC), 15.54 (CH_3), 11.21 (CH_3);

Peaks attributed to minor diastereomer (***anti*-33**): ^{13}C NMR (100 MHz, $CDCl_3$) δ 155.1 (C), 135.1 (CH), 116.79 (CH_2), 80.2 (CH), 66.2 (CH_2), 65.3 (CH_2), 50.2 (C), 36.3 (CH overlapping with ***syn*-33**, as determined by HSQC), 35.3 (CH_2), 29.0 (CH_3, overlapping with ***syn*-33**, as determined by HSQC), 15.49 (CH_3), 13.3 (CH_3);

IR (ATR) 3345 (br), 2971, 1701, 1266, 1083 cm^{-1};

HRMS (ESI/TOF) m/z: $[M + Na]^+$ Calcd for $C_{14}H_{27}NNaO_3$ 280.1883; Found 280.1871.

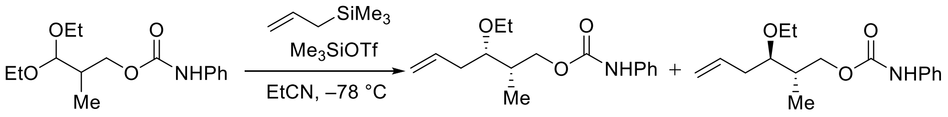

26 ***syn*-34** ***anti*-34**

(2*R,3*R**)-3-Ethoxy-2-methylhex-5-en-1-yl phenylcarbamate (*syn*-34) and (2*R**,3*S**)-3-ethoxy-2-methylhex-5-en-1-yl phenylcarbamate (*anti*-34)**. Following the general procedure for acetal substitution, acetal **26** (0.064 g, 0.23 mmol) in EtCN (2.3 mL) was mixed with allyltrimethylsilane (0.14 mL, 0.92 mmol) and trimethylsilyl trifluoromethanesulfonate (0.081 mL, 0.46 mmol) to provide a 60:40 mixture of diastereomers of ester **34**. Ester **34** was isolated as a 61:39 mixture of ***syn*-34**:***anti*-34** as a colorless oil (0.042 g, 87%):

^{1}H NMR (400 MHz, $CDCl_3$) δ 7.40–7.38 (m, 3.2H), 7.33–7.28 (m, 3.2H), 7.08–7.04 (m, 1.6H), 6.69 (s, 1.6H), 5.95–5.74 (m, 1.6H), 5.16–5.02 (m, 3.2H), 4.27 (dd, *J* = 10.6, 4.8 Hz, 0.6H), 4.22–4.12 (m, 1.6H), 4.07 (dd, *J* = 10.6, 6.6 Hz, 1H), 3.65–3.54 (m, 1.6H), 3.47–3.40 (m, 1.6H), 3.36 (td, *J* = 6.5, 3.8 Hz, 1H), 3.25 (td, *J* = 6.6, 4.6 Hz, 0.6H), 2.40–2.35 (m, 1.6H), 2.29–2.20 (m, 1.6H), 2.09–1.96 (m, 1.6H), 1.17 (t, *J* = 7.0 Hz, 1.8H), 1.16 (t, *J* = 7.0 Hz, 3H), 0.98 (d, *J* = 6.9 Hz, 1.8H), 0.97 (d, *J* = 6.9 Hz, 3H);

Peaks attributed to major diastereomer (***syn*-34**): ^{13}C NMR (100 MHz, $CDCl_3$) δ 153.7 (C), 138.05 (C), 135.3 (CH), 129.1 (CH overlapping with ***anti*-34** as determined by HSQC), 123.49 (CH), 118.8 (CH overlapping with ***anti*-34** as determined by HSQC), 117.0 (CH_2), 79.2 (CH), 67.7 (CH_2), 65.8 (CH_2), 36.3 (CH), 36.1 (CH_2), 15.7 (CH_3), 11.2 (CH_3);

Peaks attributed to minor diastereomer (***anti*-34**): ^{13}C NMR (100 MHz, $CDCl_3$) δ 153.8 (C), 138.08 (C), 134.9 (CH), 129.1 (CH overlapping with ***syn*-34** as determined by HSQC), 123.46 (CH), 118.8

(CH overlapping with ***syn*-34** as determined by HSQC), 117.1 (CH_2), 80.3 (CH), 67.4 (CH_2), 65.4 (CH_2), 36.4 (CH), 35.5 (CH_2), 15.6 (CH_3), 13.6 (CH_3);

IR (ATR) 3315 (br), 2974, 1703, 1537, 1313 cm^{-1};

HRMS (ESI/TOF) m/z: $[M + NH_4]^+$ Calcd for $C_{16}H_{27}N_2O_3$ 295.2016; Found 295.2014.

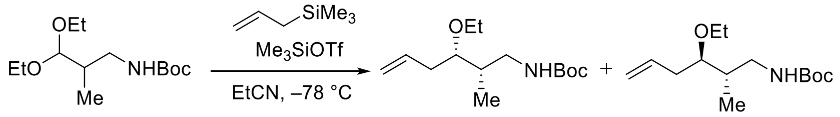

27 ***syn*-35** ***anti*-35**

tert*-Butyl ((2*R**,3*R**)-3-ethoxy-2-methylhex-5-en-1-yl)carbamate (*syn*-35) and *tert*-butyl ((2*R**,3*S**)-3-ethoxy-2-methylhex-5-en-1-yl)carbamate (*anti*-35)**. Following the general procedure for acetal substitution, acetal **27** (0.063 g, 0.24 mmol) in EtCN (2.4 mL) was mixed with allyltrimethylsilane (0.15 mL, 0.96 mmol) and trimethylsilyl trifluoromethanesulfonate (0.087 mL, 0.48 mmol) to provide a 53:47 mixture of diastereomers of ester **35**. Ester **35** was isolated as a 53:47 mixture of ***syn*-35**:anti*-35** as a colorless oil (0.035 g, 54%):

^{1}H NMR (400 MHz, $CDCl_3$) δ 5.88–5.75 (m, 2H), 5.14–4.98 (m, 6H), 3.63–3.53 (m, 2H), 3.49–3.37 (m, 2H), 3.27 (td, J = 6.5, 3.3 Hz, 1H), 3.20–3.06 (m, 5H), 2.37–2.15 (m, 4H), 1.87–1.82 (m, 1H), 1.81–1.76 (m, 1H), 1.44 (s, 18H), 1.18 (t, J = 7.0 Hz, 3H), 1.17 (t, J = 7.0 Hz, 3H), 0.92 (d, J = 7.2 Hz, 3H), 0.90 (d, J = 6.7 Hz, 3H);

^{13}C NMR (100 MHz, $CDCl_3$) δ 156.4 (C), 156.3 (C), 135.6 (CH), 134.8 (CH), 117.2 (CH_2), 116.9 (CH_2), 83.0 (CH), 81.8 (CH), 79.0 (C), 78.9 (C), 65.5 (CH_2), 65.4 (CH_2), 44.3 (CH_2), 43.9 (CH_2), 36.8 (CH), 36.5 (CH), 35.8 (CH_2), 35.6 (CH_2), 28.59 (CH_3), 28.58 (CH_3), 15.7 (CH_3), 15.6 (CH_3), 14.9 (CH_3), 12.6 (CH_3);

IR (ATR) 3410 (br), 2956, 1689, 1456, 1246 cm^{-1};

HRMS (ESI/TOF) m/z: $[M + Na]^+$ Calcd for $C_{14}H_{27}NNaO_3$ 280.1883; Found 280.1882.

Scheme 3: Synthesis of δ-acyloxy acetals bearing an α-phenyl substituent

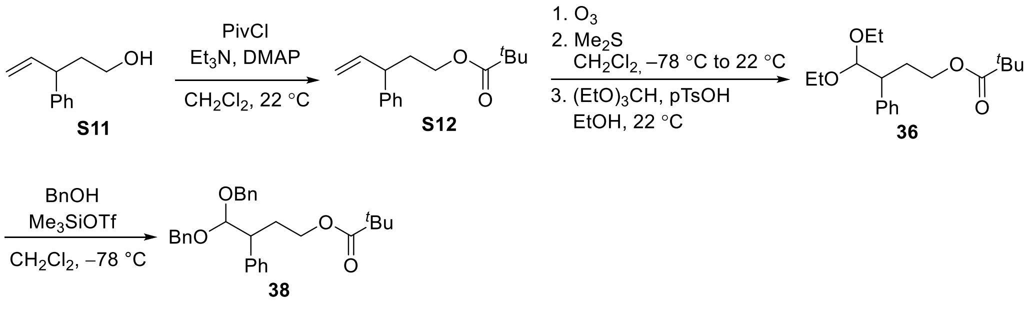

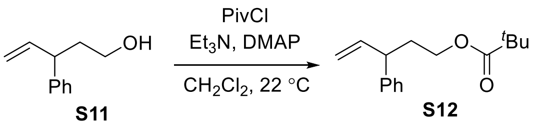

S11 **S12**

3-Phenylpent-4-en-1-yl pivalate (S12). Alcohol **S11** was prepared according to a literature procedure (8.2 g, 98% after two steps).[6] Following the general procedure for esterification of alcohols, alcohol **S11** (9.9 g, 63 mmol) in CH_2Cl_2 (130 mL) was mixed with triethylamine (17 mL, 120 mmol), 4-dimethylaminopyridine (0.75 g, 6.1 mmol), and pivaloyl chloride (15 mL, 120 mmol) to provide ester **S12** (14.3 g, 95%) as a colorless oil after impurities were removed by distillation (2.5 mmHg, 97−103 ºC):

^{1}H NMR (400 MHz, $CDCl_3$) δ 7.31 (m, 2H), 7.22–7.10 (m, 3H), 5.96 (ddd, *J* = 17.4, 12.6, 7.5 Hz, 1H), 5.11–5.02 (m, 2H), 4.02 (ddt, *J* = 12.9, 10.9, 6.2 Hz, 2H), 3.42 (q, *J* = 7.6 Hz, 1H), 2.04 (sept, *J* = 6.8 Hz, 2H), 1.20 (s, 9H);

^{13}C NMR (100 MHz, $CDCl_3$) δ 178.6 (C), 143.4 (C), 141.3 (CH), 128.8 (CH), 127.7 (CH), 126.7 (CH), 114.9 (CH_2), 62.6 (CH_2), 46.5 (CH), 38.9 (C), 34.1 (CH_2), 27.4 (CH_3);

IR (ATR) 2972, 1725, 1479, 1282, 1032 cm^{-1};

HRMS (ESI/TOF) m/z: [M + Na]$^+$ Calcd for $C_{16}H_{22}NaO_2$ 269.1517; Found 269.1518.

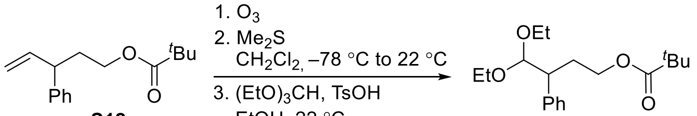

S12 36

4,4-Diethoxy-3-phenylbutyl pivalate (36). Following the general procedure for ozonolysis, alkene **S12** (7.1 g, 29 mmol) in CH_2Cl_2 (120 mL) was treated with O_3, then dimethyl sulfide (21 mL, 290 mmol), to provide the corresponding aldehyde as a yellow oil (7.1 g, quantitative).

Following the general procedure for diethyl acetal formation, the crude aldehyde (7.1 g, 29 mmol) in triethyl orthoformate (48 mL, 29 mmol) was mixed with ethanol (3.0 mL) and *p*-toluenesulfonic acid (2.7 g, 15 mmol) to provide acetal **36** as a colorless oil (5.76 g, 62%):

^{1}H NMR (400 MHz, $CDCl_3$) δ 7.38–7.27 (m, 2H), 7.23–7.21 (m, 3H), 4.50 (d, *J* = 6.1 Hz, 1H), 3.98 (ddd, *J* = 8.2, 7.4, 4.8 Hz, 1H), 3.81 (ddd, *J* = 10.8, 8.4, 6.6 Hz, 1H), 3.77–3.64 (m, 1H), 3.55 (dq, *J* = 8.7, 7.0 Hz, 1H), 3.45 (dq, *J* = 8.7, 6.9 Hz, 1H), 3.33 (dq, *J* = 8.8, 7.0 Hz, 1H), 2.97 (ddd, *J* = 10.3, 4.6, 1.6 Hz, 1H), 2.32–2.18 (m, 1H), 2.01–1.88 (m, 1H), 1.21 (t, *J* = 7.1 Hz, 3H), 1.16 (s, 9H), 1.03 (t, *J* = 7.0 Hz, 3H);

^{13}C NMR (100 MHz, $CDCl_3$) δ 178.6 (C), 140.5 (C), 128.8 (CH), 128.4 (CH), 126.8 (CH), 106.3 (CH), 63.1 (CH_2), 63.0 (CH_2), 62.9 (CH_2), 46.9 (CH), 38.8 (C), 29.6 (CH_2), 27.3 (CH_3), 15.4 (CH_3), 15.3 (CH_3);

IR (ATR) 2973, 2931, 1726, 1283, 1058 cm^{-1};

HRMS (ESI/TOF) m/z: [M + Na]$^+$ Calcd for $C_{19}H_{30}NaO_4$ 345.2042; Found 345.2050.

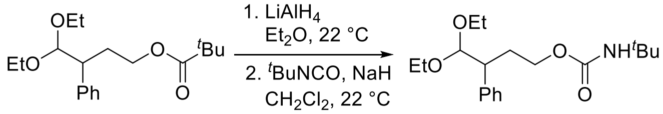

36 37

4,4-Diethoxy-3-phenylbutyl *tert*-butylcarbamate (37). Following the general procedure for ester reduction, ester **36** (0.19 g, 0.59 mmol) in Et_2O (6.0 mL) was mixed with lithium aluminum hydride (0.034 g, 0.9 mmol) to provide the crude alcohol (0.13 g, 90%), which was used in the next step without further purification.

Following the general procedure for carbamate formation, the crude alcohol (0.13 g, 0.53 mmol) in THF (5.3 mL) was mixed with NaH (0.064 g, 60% dispersion in mineral oil, 1.6 mmol) and *tert*-butyl isocyanate (0.16 mL, 1.1 mmol) to provide carbamate **37** as a colorless oil (0.12 g, 67%):

^{1}H NMR (400 MHz, $CDCl_3$) δ 7.30–7.25 (m, 2H), 7.22–7.17 (m, 3H), 4.51 (d, *J* = 6.3 Hz, 1H), 4.48 (s, 1H), 3.93–3.90 (m, 1H), 3.79 (dt, *J* = 10.6, 7.3 Hz, 1H), 3.70 (dq, *J* = 9.3, 7.0 Hz, 1H), 3.54 (dq, *J* = 9.3, 7.0 Hz, 1H), 3.45 (dq, *J* = 9.3, 7.0 Hz, 1H), 3.32 (dq, *J* = 9.3, 7.0 Hz, 1H), 2.95 (ddd, *J* = 10.6, 6.3, 4.1 Hz, 1H), 2.26–2.18 (m, 1H), 1.97–1.85 (m, 1H), 1.27 (s, 9H), 1.20 (t, *J* = 7.0 Hz, 3H), 1.01 (t, *J* = 7.0 Hz, 3H);

^{13}C NMR (100 MHz, $CDCl_3$) δ 155.0 (C), 140.7 (C), 128.8 (CH), 128.4 (CH), 126.7 (CH), 106.2 (CH), 63.1 (CH_2), 62.9 (CH_2), 62.7 (CH_2), 50.3 (C), 47.0 (CH), 30.3 (CH_2), 29.1 (CH_3), 15.4 (CH_3), 15.2 (CH_3);

IR (ATR) 3348 (br), 2973, 1709, 1265, 1058 cm^{-1};

HRMS (ESI/TOF) m/z: $[M + Na]^+$ Calcd for $C_{19}H_{31}NNaO_4$ 360.2145; Found 360.2162.

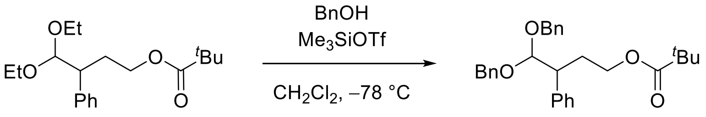

36 **38**

4,4-Bis(benzyloxy)-3-phenylbutyl pivalate (38). Following the general procedure for dibenzyl acetal formation, acetal **36** (3.2 g, 10 mmol) in CH_2Cl_2 (100 mL) was mixed with benzyl alcohol (5.2 mL, 50 mmol) and trimethylsilyl trifluoromethanesulfonate (3.6 mL, 20 mmol) to provide dibenzyl acetal **38** as a colorless oil (3.3 g, 75%):

^{1}H NMR (400 MHz, $CDCl_3$) δ 7.45–7.28 (m, 8H), 7.24–7.20 (m, 5H), 7.02 (dd, *J* = 6.6, 2.9 Hz, 2H), 4.78 (d, *J* = 6.7 Hz, 1H), 4.69 (d, *J* = 11.7 Hz, 1H), 4.57 (d, *J* = 4.5 Hz, 1H), 4.54 (d, *J* = 4.6 Hz, 1H), 4.39 (d, *J* = 11.7 Hz, 1H), 4.01 (ddd, *J* = 13.9, 7.4, 2.5, 1H), 3.83–3.76 (m, 1H), 3.16 (ddd, *J* = 10.7, 4.0, 3.7 Hz, 1H), 2.47–2.26 (m, 1H), 2.00–1.91 (m, 1H), 1.16 (s, 9H);

^{13}C NMR (100 MHz, $CDCl_3$) δ 178.5, 140.0, 138.1, 137.9, 128.9, 128.59, 128.55, 128.49, 128.4, 127.9, 127.8, 127.7, 126.9, 105.0, 69.1, 68.3, 62.6, 46.5, 38.8, 29.9, 27.3;

IR (ATR) 2973, 1722, 1284, 1154, 1038 cm^{-1};

HRMS (ESI/TOF) m/z: $[M + Na]^+$ Calcd for $C_{29}H_{34}NaO_4$ 469.2355; Found 469.2350.

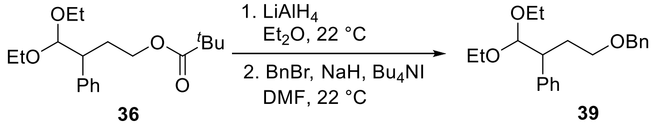

36 **39**

(4-(Benzyloxy)-1,1-diethoxybutan-2-yl)benzene (39). Following the general procedure for ester reduction, ester **36** (0.85 g, 2.6 mmol) in Et_2O (30 mL) was mixed with lithium aluminum hydride (0.15 g, 4.0 mmol) to provide the crude alcohol (0.62 g, quantitative), which was used in the next step without further purification.

To a cooled solution (–50 °C) of the crude alcohol (0.62 g, 2.6 mmol) in DMF (13 mL) were added benzyl bromide (0.94 mL, 7.9 mmol) and NaH (0.2 g, 60% dispersion in mineral oil, 5.3 mmol). The reaction mixture was warmed to –10 °C over 1 h, stirred 30 min, and warmed to 22 °C. After 3 h, tetrabutylammonium iodide (0.20 g, 0.54 mmol) was added and the mixture was stirred for an additional 16 h. Saturated aqueous NH_4Cl (10 mL) was added dropwise, and the mixture was extracted with Et_2O (3 × 30 mL). The combined organic layers were washed with brine (10 mL) and dried over anhydrous Na_2SO_4. The resulting mixture was filtered, concentrated *in vacuo*, and purified by flash column chromatography (10:90 EtOAc:hexanes) to provide acetal **39** as a colorless oil (0.47 g, 53%):

^1H NMR (400 MHz, $CDCl_3$) δ 7.36–7.27 (m, 6H), 7.26–7.18 (m, 4H), 4.53 (d, *J* = 6.3 Hz, 1H), 4.42 (d, *J* = 11.9 Hz, 1H), 4.35 (d, *J* = 11.9 Hz, 1H), 3.76–3.66 (m, 1H), 3.59–3.51 (m, 1H), 3.51–3.43 (m, 1H), 3.41–3.23 (m, 3H), 3.05 (ddd, *J* = 10.7, 6.3, 4.1 Hz, 1H), 2.28 (dddd, *J* = 13.8, 8.2, 7.4, 4.1 Hz, 1H), 1.89 (dddd, *J* = 13.8, 11.0, 6.3, 4.6 Hz, 1H), 1.20 (t, *J* = 7.0 Hz, 3H), 1.02 (t, *J* = 7.0 Hz, 3H);

^{13}C NMR (100 MHz, $CDCl_3$) δ 141.1 (C), 138.8 (C), 129.0 (CH), 128.4 (CH), 128.3 (CH), 127.7 (CH), 127.5 (CH), 126.5 (CH), 106.3 (CH), 72.9 (CH_2), 68.4 (CH_2), 62.9 (CH_2), 62.7 (CH_2), 46.6 (CH), 31.0 (CH_2), 15.4 (CH_3), 15.3 (CH_3);

IR (ATR) 2974, 2872, 1728, 1283, 1152 cm^{-1};

HRMS (ESI/TOF) m/z: $[M + Na]^+$ Calcd for $C_{21}H_{28}NaO_3$ 351.1931; Found 351.1944.

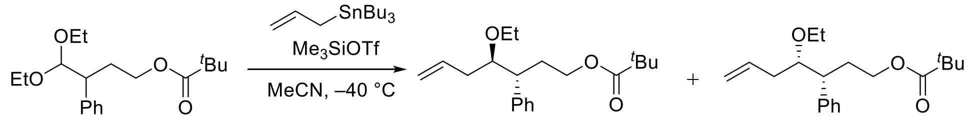

36 anti-40 syn-40

(3*R,4*R**)-4-Ethoxy-3-phenylhept-6-en-1-yl pivalate (*anti*-40) and (3*R**,4*S**)-4-ethoxy-3-phenylhept-6-en-1-yl pivalate (*syn*-40)**. Following the general procedure for acetal substitution, acetal **36** (0.10 g, 0.31 mmol) in MeCN (3.1 mL) was mixed with allyltributylstannane (0.38 mL, 1.2 mmol) and trimethylsilyl trifluoromethanesulfonate (0.11 mL, 0.62 mmol) to provide an 85:15 mixture of diastereomers of ester **40**. Ester **40** was isolated as an 84:16 mixture of ***anti*-40**:***syn*-40** with some stannane impurities as a colorless oil (0.075 g, 76%):

^1H NMR (400 MHz, $CDCl_3$) δ 7.32–7.25 (m, 3H), 7.23–7.14 (m, 3H), 5.88–5.70 (m, 1.2H), 5.14–4.91 (m, 2.4H), 4.17–3.98 (m, 0.6H), 3.99–3.90 (m, 1H), 3.76 (ddd, *J* = 10.9, 8.5, 6.6 Hz, 1H), 3.67–3.52 (m, 1.4H), 3.47–3.36 (m, 2H), 2.92–2.86 (m, 0.2H), 2.80 (ddd, *J* = 11.3, 7.5, 2.8 Hz, 1H), 2.39–2.27 (m, 1H), 2.23–2.16 (m, 1H), 2.12–1.98 (m, 1.4H), 1.90 (dddd, *J* = 13.8, 11.2, 6.6, 4.7 Hz, 1H), 1.75–1.69 (m, 0.4H), 1.23–1.13 (m, 14.4H);

Peaks attributed to major diastereomer (***anti*-40**): ^{13}C NMR (100 MHz, $CDCl_3$) δ 178.6 (C), 142.0 (C), 134.9 (CH), 128.63 (CH), 128.55 (CH), 126.8 (CH), 117.2 (CH_2), 83.0 (CH), 65.7 (CH_2), 63.2 (CH_2), 46.6 (CH), 38.8 (C), 36.5 (CH_2), 30.5 (CH_2), 27.32 (CH_3), 15.6 (CH_3);

Peaks attributed to minor diastereomer (***syn*-40**): ^{13}C NMR (100 MHz, $CDCl_3$, characteristic peaks) δ 140.8 (C), 135.4 (CH), 129.4 (CH), 128.3 (CH), 126.6 (CH), 117.0 (CH_2), 82.9 (CH), 66.1 (CH_2), 62.9 (CH_2), 45.8 (CH), 38.9 (C), 36.4 (CH_2), 30.8 (CH_2), 27.26 (CH_3);

IR (ATR) 2971, 2930, 1727, 1152, 701 cm^{-1};

HRMS (ESI/TOF) m/z: [M + Na]$^+$ Calcd for $C_{20}H_{30}NaO_3$ 341.2087; Found 341.2072.

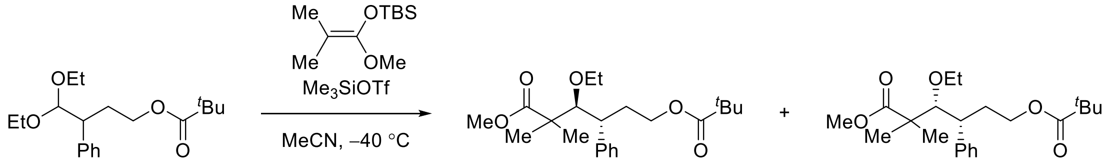

36 ***anti*-41** ***syn*-41**

Methyl (3*R,4*S**)-3-ethoxy-2,2-dimethyl-4-phenyl-6-(pivaloyloxy)hexanoate (*anti*-41) and methyl (3*R**,4*R**)-3-ethoxy-2,2-dimethyl-4-phenyl-6-(pivaloyloxy)hexanoate (*syn*-41).** Following the general procedure for acetal substitution, acetal **36** (0.50 g, 1.5 mmol) in EtCN (15 mL) was mixed with silyl ketene acetal *tert*-butyl((1-methoxy-2-methylprop-1-en-1-yl)oxy)dimethylsilane (1.5 mL, 6.0 mmol) and trimethylsilyl trifluoromethanesulfonate (0.55 mL, 3.0 mmol) to provide a 94:6 mixture of diastereomers of ester **41**. Ester **41** was isolated as a 90:10 mixture of ***anti*-41**:***syn*-41** as a colorless oil (0.54 g, 95%):

^{1}H NMR (400 MHz, $CDCl_3$) δ 7.28–7.26 (m, 1.2H), 7.25 (m, 1.3H), 7.19–7.14 (m, 3H), 3.90 (m, 1.1H), 3.83–3.54 (m, 4.2H), 3.46–3.36 (m, 0.1H), 3.27 (s, 3.3H), 3.08 (dq, J = 8.7, 7.0 Hz, 0.1H), 2.95–2.79 (m, 1.1H), 2.34 (dddd, J = 13.9, 9.2, 6.8, 3.6 Hz, 1.2H), 1.92–1.76 (m, 1H), 1.24–1.18 (m, 6.6H), 1.17 (s, 9.9H), 1.10 (s, 0.3H), 1.07 (s, 3H);

Peaks attributed to major diastereomer (***anti*-41**): ^{13}C NMR (100 MHz, $CDCl_3$) δ 178.6, 177.0, 142.4, 128.9, 128.6, 126.8, 87.6, 69.86, 62.7, 51.4, 48.4, 45.4, 38.8, 31.6, 27.4, 23.5, 20.0, 15.7;

Peaks attributed to minor diastereomer (***syn*-41**): ^{13}C NMR (100 MHz, $CDCl_3$, characteristic peaks) δ 129.5, 128.2, 126.7, 87.8, 69.90, 62.4, 51.9, 47.8, 45.8, 32.5, 24.5, 19.2;

IR (ATR) 2973, 1725, 1454, 1282, 1033 cm^{-1}.

HRMS (ESI/TOF) m/z: [M + Na]$^+$ Calcd for $C_{22}H_{34}NaO_5$ 401.2304; Found 401.2306.

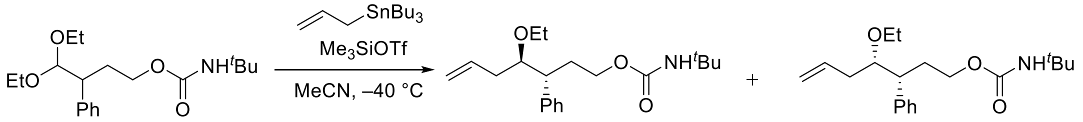

37 ***anti*-42** ***syn*-42**

(3*R,4*R**)-4-Ethoxy-3-phenylhept-6-en-1-yl *tert*-butylcarbamate (*anti*-42) and (3*R**,4*S**)-4-ethoxy-3-phenylhept-6-en-1-yl *tert*-butylcarbamate (*syn*-42)**. Following the general procedure for acetal substitution, acetal **37** (0.021 g, 0.062 mmol) in MeCN (0.62 mL) was mixed with

allyltributylstannane (0.077 mL, 0.25 mmol) and trimethylsilyl trifluoromethanesulfonate (0.022 mL, 0.13 mmol) to provide an 80:20 mixture of diastereomers of ester **42**. Ester **42** was isolated as an 80:20 mixture of ***anti*-42**:***syn*-42** with some stannane impurities as a colorless oil (0.018 g, 87%):

^{1}H NMR (400 MHz, $CDCl_3$) δ 7.29–7.27 (m, *J* = 7.0 Hz, 1.6H), 7.26–7.12 (m, 4.9H), 5.84–5.70 (m, 1.3H), 5.05–4.90 (m, 2.6H), 4.53–4.42 (m, 1.3H), 3.99–3.84 (m, 1.3H), 3.80–3.68 (m, 1.3H), 3.67–3.51 (m, 1.3H), 3.48–3.28 (m, 2.6H), 2.87–2.82 (m, 0.3H), 2.77 (ddd, *J* = 11.5, 8.0, 3.6 Hz, 1H), 2.32 (dtd, *J* = 13.7, 7.6, 3.6 Hz, 1H), 2.24–2.15 (m, 1.3H), 2.11–1.94 (m, 1.9H), 1.91–1.81 (m, 1H), 1.29 (s, 2.7H), 1.28 (m, 9H), 1.20 (t, *J* = 7.0 Hz, 3H), 1.12 (t, *J* = 7.0 Hz, 0.9H);

Peaks attributed to major diastereomer (***anti*-42**): ^{13}C NMR (100 MHz, $CDCl_3$) δ 155.1 (C), 142.1 (C), 134.9 (CH), 128.61 (CH), 128.57 (CH), 126.7 (CH), 117.2 (CH_2), 82.94 (CH), 65.8 (CH_2), 62.9 (CH_2), 50.3 (C), 47.0 (CH), 36.4 (CH_2), 31.3 (CH_2), 29.1 (CH_3), 15.6 (CH_3);

Peaks attributed to minor diastereomer (***syn*-42**): ^{13}C NMR (100 MHz, $CDCl_3$, characteristic peaks) δ 141.1 (C), 135.5 (CH), 129.4 (CH), 128.2 (CH), 126.5 (CH), 116.9 (CH_2), 82.90 (CH), 66.2 (CH_2), 46.1 (CH), 36.6 (CH_2), 31.4 (CH_2), 15.7 (CH_3);

IR (ATR) 3346 (br), 2960, 2925, 1708, 1087 cm^{-1};

HRMS (ESI/TOF) m/z: [M + H]$^+$ Calcd for $C_{20}H_{32}NO_3$ 334.2377; Found 334.2366.

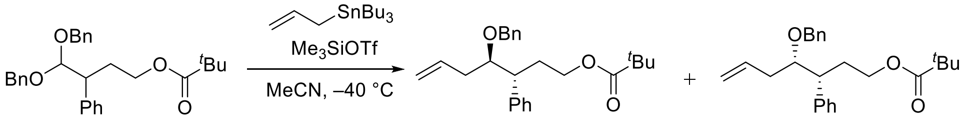

38 ***anti*-43** ***syn*-43**

(3*R,4*R**)-4-(Benzyloxy)-3-phenylhept-6-en-1-yl pivalate (*anti*-43) and (3*R**,4*S**)-4-(benzyloxy)-3-phenylhept-6-en-1-yl pivalate (*syn*-43)**. Following the general procedure for acetal substitution, acetal **38** (0.088 g, 0.20 mmol) in MeCN (2.0 mL) was mixed with allyltributylstannane (0.24 mL, 0.80 mmol) and trimethylsilyl trifluoromethanesulfonate (0.070 mL, 0.40 mmol) to provide an 82:18 mixture of diastereomers of ester **43**. Ester **43** was isolated as an 82:18 mixture of ***anti*-43**:***syn*-43** with some stannane impurities as a colorless oil (0.080 g, quantitative):

^{1}H NMR (400 MHz, $CDCl_3$) δ 7.36–7.28 (m, 8H), 7.23–7.15 (m, 4H), 5.90–5.70 (m, 1.2H), 5.08–4.97 (m, 2.4H), 4.63 (d, *J* = 11.2 Hz, 1H), 4.58 (d, *J* = 11.4 Hz, 0.2H), 4.45 (d, *J* = 11.2 Hz, 1H), 4.38 (d, *J* = 11.4 Hz, 0.2H), 4.21–4.09 (m, 0.4H), 3.95 (ddd, *J* = 10.9, 7.2, 4.8 Hz, 1H), 3.75 (ddd, *J* = 10.9, 8.6, 6.4 Hz, 1H), 3.63–3.59 (m, 1H), 3.48–3.40 (m, 0.2H), 2.99–2.93 (m, 0.2H), 2.90 (ddd, *J* = 11.2, 7.6, 3.7 Hz, 1H), 2.39 (dddd, *J* = 13.9, 8.6, 7.2, 3.7 Hz, 1H), 2.34–2.26 (m, 1H), 2.23–2.16 (m, 0.4H), 2.14–1.99 (m, 1.4H), 1.90 (dddd, *J* = 13.9, 11.2, 6.4, 4.7 Hz, 1H), 1.15 (s, 10.8H);

Peaks attributed to major diastereomer (***anti*-43**): ^{13}C NMR (100 MHz, $CDCl_3$) δ 178.6 (C), 141.8 (C), 138.7 (C), 134.6 (CH), 128.7 (CH), 128.63 (CH), 128.5 (CH), 128.0 (CH), 127.7 (CH), 126.9 (CH), 117.5 (CH_2), 82.6 (CH), 72.1 (CH_2), 63.1 (CH_2), 46.4 (CH), 38.8 (C), 36.09 (CH_2), 30.4 (CH_2), 27.33 (CH_3);

Peaks attributed to minor diastereomer (***syn*-43**): ^{13}C NMR (100 MHz, $CDCl_3$, characteristic peaks) δ 128.56 (CH), 128.40 (CH), 128.37 (CH), 127.8 (CH), 127.6 (CH), 126.7 (CH), 117.4 (CH_2), 82.4 (CH), 72.4 (CH_2), 62.8 (CH_2), 36.05 (CH_2), 30.8 (CH_2), 27.26 (CH_3);

IR (ATR) 2958, 2871, 1725, 1284, 1152 cm^{-1};

HRMS (ESI/TOF) m/z: $[M + Na]^+$ Calcd for $C_{25}H_{32}NaO_3$ 403.2244; Found 403.2262.

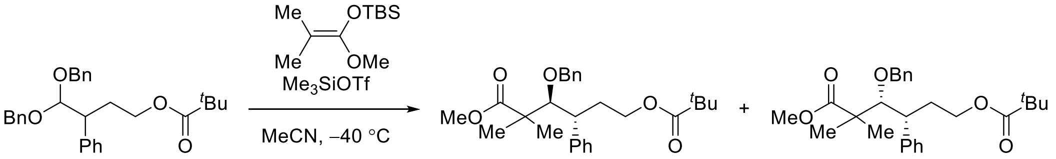

38 ***anti*-44** ***syn*-44**

Methyl (3*R,4*S**)-3-(benzyloxy)-2,2-dimethyl-4-phenyl-6-(pivaloyloxy)hexanoate (*anti*-44) and methyl (3*R**,4*R**)-3-(benzyloxy)-2,2-dimethyl-4-phenyl-6-(pivaloyloxy)hexanoate (*syn*-44).** Following the general procedure for acetal substitution, acetal **38** (0.45 g, 1.0 mmol) in EtCN (10 mL) was mixed with silyl ketene acetal *tert*-butyl((1-methoxy-2-methylprop-1-en-1-yl)oxy)dimethylsilane (1.0 mL, 4.0 mmol) and trimethylsilyl trifluoromethanesulfonate (0.36 mL, 2.0 mmol) to provide an 86:14 mixture of diastereomers of ester **44**. Ester **44** was isolated as a 90:10 mixture of ***anti*-44**:***syn*-44** as a colorless oil (0.34 g, 86%). A pure sample of ester ***anti*-44** was isolated in small fractions by flash column chromatography and incorporated into a guanidinium sulfonate framework host for crystal structure determination. Characterization was done on the pure sample of ***anti*-44**:

^1H NMR (400 MHz, $CDCl_3$) δ 7.38–7.26 (m, 7H), 7.22–7.14 (m, 3H), 4.74 (d, *J* = 11.1 Hz, 1H), 4.65 (d, *J* = 11.1 Hz, 1H), 4.00 (d, *J* = 7.0 Hz, 1H), 3.93–3.88 (m, 1H), 3.63 (ddd, *J* = 10.9, 9.3, 5.7 Hz, 1H), 3.30 (s, 3H), 2.99 (ddd, *J* = 11.9, 7.0, 3.5 Hz, 1H), 2.41 (dddd, *J* = 13.9, 9.3, 6.7, 3.5 Hz, 1H), 1.97–1.84 (m, 1H), 1.29 (s, 3H), 1.16 (s, 9H), 1.12 (s, 3H);

^{13}C NMR (100 MHz, $CDCl_3$) δ 178.5, 176.9, 142.5, 138.6, 128.9, 128.7, 128.5, 127.7, 127.4, 126.9, 87.6, 76.0, 62.7, 51.5, 48.7, 45.4, 38.8, 31.4, 27.3, 23.2, 20.4;

IR (ATR) 2955, 2930, 1727, 1067 cm^{-1};

HRMS (ESI/TOF) m/z: $[M + H]^+$ Calcd for $C_{27}H_{37}O_5$ 441.2641; Found 441.2646.

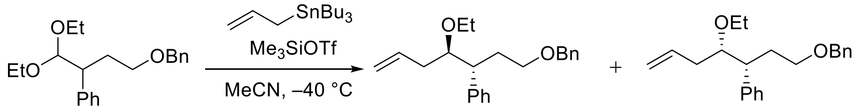

39 ***anti*-45** ***syn*-45**

((3*R,4*R**)-1-(Benzyloxy)-4-ethoxyhept-6-en-3-yl)benzene (*anti*-45) and ((3*R**,4*S**)-1-(benzyloxy)-4-ethoxyhept-6-en-3-yl)benzene (*syn*-45)**. Following the general procedure for acetal substitution, acetal **39** (0.13 g, 0.40 mmol) in MeCN (4.0 mL) was mixed with allyltributylstannane (0.49 mL, 1.6 mmol) and trimethylsilyl trifluoromethanesulfonate (0.14 mL, 0.80 mmol) to provide a 68:32 mixture of diastereomers of ester **45**. Ester **45** was isolated as a 67:33 mixture of ***anti*-45**:***syn*-45** with some stannane impurities as a colorless oil (0.088 g, 68%):

^{1}H NMR (400 MHz, $CDCl_3$) δ 7.40–7.26 (m, 10H), 7.26–7.13 (m, 5H), 5.89–5.73 (m, 1.5H), 5.13–4.92 (m, 3H), 4.56–4.38 (m, 2H), 4.34 (d, J = 11.9 Hz, 1H), 3.67–3.51 (m, 2H), 3.51–3.33 (m, 3.5H), 3.32–3.22 (m, 2H), 2.97–2.89 (m, 0.5H), 2.84 (ddd, J = 11.4, 7.7, 3.7 Hz, 1H), 2.39 (dtd, J = 13.6, 8.0, 3.7 Hz, 1H), 2.35–2.13 (m, 2H), 2.12–2.05 (m, 1H), 2.05–1.95 (m, 1H), 1.87 (dddd, J = 13.6, 11.4, 7.1, 4.7 Hz, 1H), 1.20 (t, J = 7.1 Hz, 3H), 1.12 (t, J = 7.0 Hz, 1.5H);

Peaks attributed to major diastereomer (***anti*-45**): ^{13}C NMR (100 MHz, $CDCl_3$) δ 142.5 (C), 138.8 (C), 135.1 (CH), 128.7 (CH), 128.5 (CH), 128.39 (CH), 127.69 (CH), 127.5 (CH), 126.5 (CH), 117.0 (CH_2), 83.1 (CH), 72.9 (CH_2), 68.9 (CH_2), 66.2 (CH_2), 46.7 (CH), 36.6 (CH_2), 31.8 (CH_2), 15.6 (CH_3);

Peaks attributed to minor diastereomer (***syn*-45**): ^{13}C NMR (100 MHz, $CDCl_3$) δ 141.5 (C), 138.7 (C), 135.7 (CH), 129.5 (CH), 128.44 (CH), 128.1 (CH), 127.74 (CH), 127.6 (CH), 126.4 (CH), 116.8 (CH_2), 83.2 (CH), 73.0 (CH_2), 68.7 (CH_2), 65.7 (CH_2), 46.0 (CH), 36.9 (CH_2), 32.3 (CH_2), 15.7 (CH_3);

IR (ATR) 2857, 1453, 1094, 910, 607 cm^{-1};

HRMS (ESI/TOF) m/z: $[M + Na]^+$ Calcd for $C_{22}H_{28}NaO_2$ 347.1982; Found 347.1973.

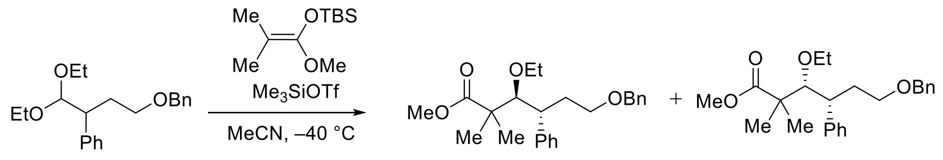

39 ***anti*-46** ***syn*-46**

Methyl (3*R,4*S**)-6-(benzyloxy)-3-ethoxy-2,2-dimethyl-4-phenylhexanoate (*anti*-46) and methyl (3*R**,4*R**)-6-(benzyloxy)-3-ethoxy-2,2-dimethyl-4-phenylhexanoate (*syn*-46).** Following the general procedure for acetal substitution, acetal **39** (0.10 g, 0.30 mmol) in MeCN (3.0 mL) was mixed with silyl ketene acetal *tert*-butyl((1-methoxy-2-methylprop-1-en-1-yl)oxy)dimethylsilane (0.29 mL, 1.2 mmol) and trimethylsilyl trifluoromethanesulfonate (0.11 mL, 0.60 mmol) to provide a 72:28 mixture of diastereomers of ester **46**. Ester **46** was isolated as a 78:22 mixture of ***anti*-46**:***syn*-46** as a colorless oil (0.098 g, 85%):

^{1}H NMR (400 MHz, $CDCl_3$) δ 7.36–7.26 (m, 5H), 7.25–7.12 (m, 8H), 4.37 (d, J = 11.9 Hz, 1H), 4.36 (d, J = 11.9 Hz, 0.3H), 4.31–4.28 (m, 1.3H), 3.84–3.70 (m, 2.6H), 3.66 (s, 0.9H), 3.60–3.51 (m, 1H), 3.47–3.38 (m, 0.3H), 3.23 (s, 3H), 3.22–3.04 (m, 2.6H), 2.94–2.88 (m, 1.3H), 2.40 (dtd, J = 13.4, 8.1, 3.5 Hz, 1H), 1.94–1.87 (m, 0.6H), 1.79 (dddd, J = 13.4, 11.6, 7.0, 4.3 Hz, 1H), 1.24–1.17 (m, 6H), 1.06 (s, 1.8H), 1.04 (s, 3H), 0.98 (t, J = 7.0 Hz, 0.9H);

Peaks attributed to major diastereomer (***anti*-46**): ^{13}C NMR (100 MHz, $CDCl_3$) δ 177.0 (C), 142.6 (C), 138.7 (C), 129.1 (CH), 128.38 (CH), 128.3 (CH), 127.71 (CH), 127.5 (CH), 126.5 (CH), 87.5 (CH), 72.92 (CH_2), 69.76 (CH_2), 68.6 (CH_2), 51.3 (CH_3), 48.3 (C), 45.62 (CH), 32.9 (CH_2), 23.6 (CH_3), 19.9 (CH_3), 15.67 (CH_3);

Peaks attributed to minor diastereomer (***syn*-46**): ^{13}C NMR (100 MHz, $CDCl_3$) δ 178.2 (C), 142.4 (C), 138.4 (C), 129.7 (CH), 128.44 (CH), 128.0 (CH), 127.66 (CH), 127.6 (CH), 126.4 (CH), 88.1

(CH), 72.90 (CH_2), 69.78 (CH_2), 68.2 (CH_2), 51.8 (CH_3), 47.9 (C), 45.60 (CH), 34.1 (CH_2), 24.1 (CH_3), 19.7 (CH_3), 15.72 (CH_3);

IR (ATR) 2974, 2948, 1727, 1269, 1081 cm^{-1};

HRMS (ESI/TOF) m/z: $[M + Na]^+$ Calcd for $C_{24}H_{32}NaO_4$ 407.2193; Found 407.2174.

Scheme 4: Synthesis of δ-acyloxy acetal bearing an α-methyl substituent.

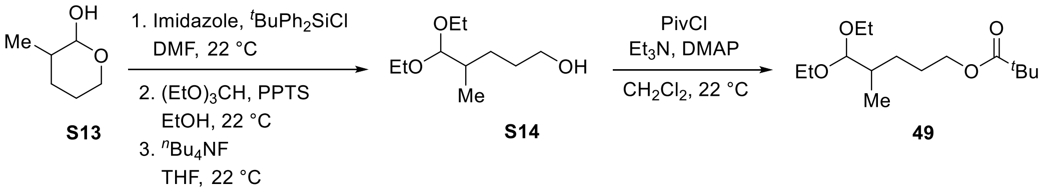

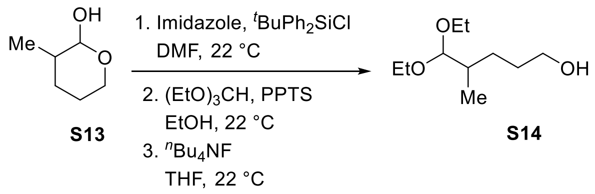

5,5-Diethoxy-4-methylpentan-1-ol (S14). Lactol **S13** was prepared according to a literature procedure (0.93 g, 36% after two steps).[7] To a cooled (0 °C) solution of lactol **S13** (0.60 g, 5.2 mmol) in DMF (10 mL) were added imidazole (0.53 g, 7.8 mmol) and *tert*-butyl(chloro)diphenylsilane dropwise (2.0 mL, 7.8 mmol). The reaction mixture was stirred for 30 min, warmed to 22 °C, and stirred for an additional 2 h. Et_2O (20 mL) was added. The mixture was washed with H_2O (2 × 5 mL) and brine (10 mL) and dried over anhydrous Na_2SO_4. The resulting mixture was filtered and concentrated *in vacuo* to provide a mixture of the corresponding aldehyde and silane impurity as a colorless oil (2.4 g), which was used directly in the next step without further purification.

The crude aldehyde mixture (2.4 g) was dissolved in triethyl orthoformate (10 mL) and ethanol (1.0 mL). Pyridinium p-toluenesulfonate (0.20 g, 0.78 mmol) was added, and the mixture was stirred at 22 °C for 16 h. Saturated aqueous $NaHCO_3$ (10 mL) was added, and the resulting mixture was extracted with CH_2Cl_2 (3 × 10 mL). The combined organic layers were washed with brine (10 mL) and dried over anhydrous Na_2SO_4. The resulting mixture was filtered, concentrated *in vacuo*, and purified by flash column chromatography (5:95 EtOAc:hexanes) to provide a 1:2.5 mixture of the corresponding acetal and unreacted triethyl orthoformate as a colorless oil (2.3 g), which was used directly in the next step without further purification.

To a solution of the crude acetal mixture (2.3 g) in THF (30 mL) was added tetrabutylammonium fluoride (4.4 mL, 1.0 M in THF, 4.4 mmol). The reaction mixture was stirred at 22 °C for 16 h. The resulting mixture was concentrated *in vacuo* and purified by flash column chromatography (40:60 EtOAc:hexanes) to provide hydroxy acetal **S14** as a colorless oil (0.32 g, 32% after three steps):

^1H NMR (400 MHz, $CDCl_3$) δ 4.18 (d, J = 6.3 Hz, 1H), 3.71–3.62 (m, 4H), 3.54–3.44 (m, 2H), 1.80–1.47 (m, 4H), 1.40 (s, 1H), 1.21 (t, J = 7.1 Hz, 3H), 1.20 (t, J = 7.1 Hz, 3H), 1.12–1.18 (m, 1H), 0.93 (d, J = 6.7 Hz, 3H);

^{13}C NMR (100 MHz, $CDCl_3$) δ 107.0 (CH), 63.4 (CH_2), 62.6 (CH_2), 62.2 (CH_2), 36.4 (CH), 30.4 (CH_2), 28.0 (CH_2), 15.48 (CH_3), 15.47 (CH_3), 14.8 (CH_3);

IR (ATR) 3392 (br), 2974, 2873, 1112, 1064 cm^{-1};

HRMS (ESI/TOF) m/z: $[M + Na]^+$ Calcd for $C_{10}H_{22}NaO_3$ 213.1461; Found 213.1454.

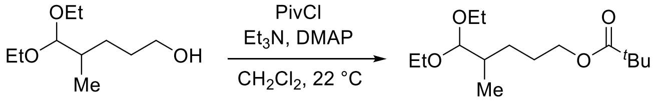

S14 **49**

5,5-Diethoxy-4-methylpentyl pivalate (49). To a cooled (0 °C) solution of hydroxy acetal **S14** (0.20 g, 1.1 mmol) in CH_2Cl_2 (5.0 mL) were added triethylamine (0.22 mL, 1.6 mmol) and 4-dimethylaminopyridine (0.013 g, 0.11 mmol). Pivaloyl chloride (0.19 mL, 1.6 mmol) was added dropwise, and the mixture was stirred at 22 °C for 16 h. H_2O (10 mL) was added and the mixture was extracted with CH_2Cl_2 (3 × 10 mL). The combined organic layers were washed with brine (10 mL) and dried over anhydrous Na_2SO_4. The resulting mixture was filtered, concentrated in vacuo, and purified by flash column chromatography (10:90 EtOAc:hexanes) to provide ester **49** as a colorless oil (0.25 g, 87%):

^1H NMR (400 MHz, $CDCl_3$) δ 4.16 (d, J = 6.3 Hz, 1H), 4.04 (td, J = 6.3, 1.6 Hz, 2H), 3.69–3.61 (m, 2H), 3.52–3.43 (m, 2H), 1.78–1.52 (m, 5H), 1.20 (t, J = 7.0 Hz, 6H), 1.19 (s, 9H), 0.92 (d, J = 6.8 Hz, 3H);

^{13}C NMR (100 MHz, $CDCl_3$) δ 178.8 (C), 106.9 (CH), 64.8 (CH_2), 62.4 (CH_2), 62.2 (CH_2), 38.9 (C), 36.3 (CH), 28.2 (CH_2), 27.3 (CH_3), 26.3 (CH_2), 15.47 (CH_3), 15.46 (CH_3), 14.7 (CH_3);

IR (ATR) 2973, 2874, 1728, 1153, 1058 cm^{-1};

HRMS (ESI/TOF) m/z: $[M + Na]^+$ Calcd for $C_{15}H_{30}NaO_4$ 297.2036; Found 297.2035.

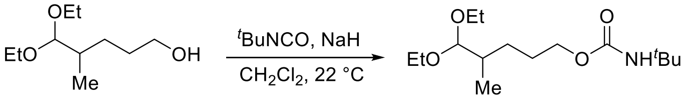

49 **50**

5,5-Diethoxy-4-methylpentyl *tert*-butylcarbamate (50). Following the general procedure for carbamate formation, the crude alcohol **49** (0.080 g, 0.42 mmol) in THF (4.2 mL) was mixed with NaH (0.034 g, 60% dispersion in mineral oil, 0.84 mmol), and *tert*-butyl isocyanate (0.096 mL, 0.84 mmol) to provide carbamate **50** as a colorless oil (0.12 g, quantitative):

^1H NMR (400 MHz, $CDCl_3$) δ 4.58 (s, 1H), 4.16 (d, J = 6.2 Hz, 1H), 3.99 (t, J = 6.7 Hz, 2H), 3.65 (dq, J = 9.3, 7.1 Hz, 2H), 3.53–3.44 (m, 2H), 1.79–1.51 (m, 5H), 1.31 (s, 9H), 1.20 (t, J = 7.1 Hz, 6H), 0.91 (d, J = 6.8 Hz, 3H);

^{13}C NMR (100 MHz, $CDCl_3$) δ 155.3 (C), 106.9 (CH), 64.6 (CH_2), 62.5 (CH_2), 62.2 (CH_2), 50.4 (C), 36.4 (CH), 29.1 (CH_3), 28.2 (CH_2), 26.8 (CH_2), 15.5 (two CH_3 overlapping, as determined by HSQC), 14.6 (CH_3);

IR (ATR) 3349 (br), 2962, 1635, 1538, 1205 cm^{-1};

HRMS (ESI/TOF) m/z: [M + Na]$^+$ Calcd for $C_{15}H_{31}NNaO_4$ 312.2145; Found 312.2143.

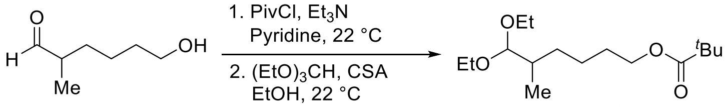

6,6-Diethoxy-5-methylhexyl pivalate (51). Hydroxy aldehyde **S15** was prepared according to a literature procedure (0.28g, 43% after two steps).[8] To a cooled (0 °C) solution of hydroxy aldehyde **S15** (0.14 g, 1.1 mmol) in CH_2Cl_2 (2.5 mL) were added triethylamine (0.30 mL, 2.2 mmol) and 4-dimethylaminopyridine (0.013 g, 0.11 mmol). Pivaloyl chloride (0.27 mL, 2.2 mmol) was added dropwise, and the mixture was stirred at 0 °C for 3 h. The mixture was warmed to 22 °C and stirred for an additional 1 h. The resulting mixture was poured into saturated aqueous $NaHCO_3$ solution (10 mL) and extracted with CH_2Cl_2 (3 × 10 mL). The combined organic layers were washed with brine (10 mL) and dried over anhydrous Na_2SO_4. The resulting mixture was filtered and concentrated *in vacuo* to provide the corresponding ester (0.28 g, quantitative), which was used directly in the next step without further purification.

The crude ester (0.14 g, 1.1 mmol) was dissolved in triethyl orthoformate (1.8 mL) and ethanol (0.20 mL). Camphorsulfonic acid (0.12 g, 0.55 mmol) was added, and the mixture was stirred at 22 °C for 16 h. Saturated aqueous $NaHCO_3$ (10 mL) was added, and the resulting mixture was extracted with CH_2Cl_2 (3 × 10 mL). The combined organic layers were washed with brine (10 mL) and dried over anhydrous Na_2SO_4. The resulting mixture was filtered, concentrated *in vacuo*, and purified by flash column chromatography (10:90 EtOAc:hexanes) to provide acetal **51** as a colorless oil (0.16 g, 50% over two steps):

^{1}H NMR (400 MHz, $CDCl_3$) δ 4.15 (d, *J* = 6.3 Hz, 1H), 4.05 (t, *J* = 6.6 Hz, 2H), 3.65 (dqd, *J* = 9.4, 7.1, 0.9 Hz, 2H), 3.48 (dqd, *J* = 9.4, 7.1, 1.3 Hz, 2H), 1.71 (dquind, *J* = 9.2, 6.7, 3.9 Hz, 1H), 1.65–1.50 (m, 3H), 1.47–1.37 (m, 1H), 1.36–1.28 (m, 1H), 1.22–1.81 (m, 15H), 1.16–1.04 (m, 1H), 0.90 (d, *J* = 6.7 Hz, 3H);

^{13}C NMR (100 MHz, $CDCl_3$) δ 178.8 (C), 107.0 (CH), 64.50 (CH_2), 62.45 (CH_2), 62.1 (CH_2), 38.9 (C), 36.6 (CH), 31.5 (CH_2), 29.1 (CH_2), 27.4 (CH_3), 26.7 (CH_3), 23.5 (CH_2), 15.5 (CH_3), 14.6 (CH_3);

IR (ATR) 2973, 2868, 1729, 1453, 1057 cm^{-1};

HRMS (ESI/TOF) m/z: [M + Na]$^+$ Calcd for $C_{16}H_{32}NaO_4$ 311.2193; Found 311.2184.

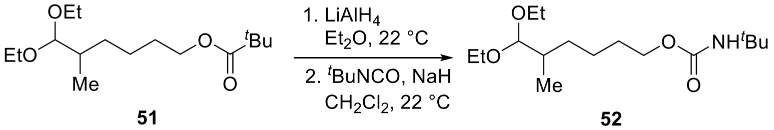

6,6-Diethoxy-5-methylhexyl *tert*-butylcarbamate (52). Following the general procedure for ester reduction, ester **51** (0.075 g, 0.26 mmol) in Et_2O (3.0 mL) was mixed with lithium aluminum hydride (0.020 g, 0.52 mmol) to provide the crude alcohol (0.047 g, 88%), which was used in the next step without further purification.

Following the general procedure for carbamate formation, the crude alcohol (0.047 g, 0.23 mmol) in THF (2.3 mL) was mixed with NaH (0.018 g, 60% dispersion in mineral oil, 0.46 mmol) and *tert*-butyl isocyanate (0.053 mL, 0.46 mmol) to provide carbamate **52** as a colorless oil (0.062 g, 89%):

^{1}H NMR (400 MHz, $CDCl_3$) δ 4.59 (s, 1H), 4.15 (d, *J* = 6.5 Hz, 1H), 3.99 (t, *J* = 6.8 Hz, 2H), 3.69–3.61 (m, 2H), 3.52–3.44 (m, 2H), 1.74–1.67 (m, 1H), 1.61–1.50 (m, 5H), 1.31–1.26 (s, 10H), 1.20 (t, *J* = 7.1 Hz, 3H), 1.19 (t, *J* = 7.1 Hz, 3H), 0.91–0.89 (m, 3H);

^{13}C NMR (100 MHz, $CDCl_3$) δ 155.3 (C), 107.0 (CH), 64.4 (CH_2), 62.4 (CH_2), 62.2 (CH_2), 50.3 (C), 36.6 (CH), 31.6 (CH_2), 29.5 (CH_2), 29.1 (CH_3), 23.6 (CH_2), 15.5 (two CH_3 overlapping, as determined by HSQC), 14.6 (CH_3);

IR (ATR) 3347 (br), 2970, 1704, 1266, 1059 cm^{-1};

HRMS (ESI/TOF) m/z: [M + Na]$^+$ Calcd for $C_{16}H_{33}NNaO_4$ 326.2302; Found 326.2296.

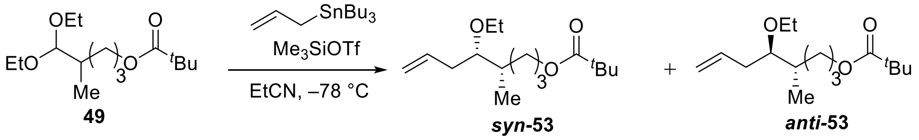

(4*R,5*R**)-5-Ethoxy-4-methyloct-7-en-1-yl pivalate (*syn*-53) and (4*R**,5*S**)-5-ethoxy-4-methyloct-7-en-1-yl pivalate (*anti*-53).** Following the general procedure for acetal substitution, acetal **49** (0.059 g, 0.21 mmol) in EtCN (2.1 mL) was mixed with allyltributylstannane (0.27 mL, 0.48 mmol) and trimethylsilyl trifluoromethanesulfonate (0.076 mL, 0.42 mmol) to provide a 59:41 mixture of diastereomers of ester **53**. Ester **53** was isolated as a 59:41 mixture of ***syn*-53**:***anti*-53** with some stannane impurities as a colorless oil (0.052 g, 91%):

^{1}H NMR (400 MHz, $CDCl_3$) δ 5.93–5.76 (m, 1.7H), 5.11–4.99 (m, 3.4H), 4.06–4.02 (m, 3.4H), 3.59–3.48 (m, 1.7H), 3.48–3.40 (m, 1.7H), 3.13 (ddd, *J* = 7.0, 5.5, 4.4 Hz, 1H), 3.08 (ddd, *J* = 7.0, 5.9, 4.7 Hz, 0.7H), 2.31–2.15 (m, 3.4H), 1.78–1.47 (m, 8.5H), 1.19 (s, 15.3H), 1.17 (t, *J* = 7.0 Hz, 2.1H), 1.16 (t, *J* = 7.0 Hz, 3H), 0.91–0.87 (m, 5.1H);

Peaks attributed to major diastereomer (***syn*-53**): ^{13}C NMR (100 MHz, $CDCl_3$) δ178.8 (C overlapping with ***anti*-53**), 136.0 (CH), 116.5 (CH_2 overlapping with ***anti*-53** as determined by HSQC), 82.9 (CH), 65.6 (CH_2), 64.76 (CH_2), 38.88 (C) 35.9 (CH), 35.7 (CH_2), 29.1 (CH_2), 28.0 (CH overlapping with ***anti*-53** as determined by HSQC), 27.4 (CH_3), 15.8 (CH_3), 14.8 (CH_3);

Peaks attributed to minor diastereomer (***anti*-53**): ^{13}C NMR (100 MHz, $CDCl_3$) δ 178.8 (C overlapping with ***syn*-53**), 135.9 (CH), 116.5 (CH_2 overlapping with ***syn*-53** as determined by HSQC), 83.3 (CH), 65.3 (CH_2), 64.81 (CH_2), 38.87 (C), 35.8 (CH), 35.3 (CH_2), 28.8 (CH_2), 28.0 (CH overlapping with ***syn*-53** as determined by HSQC), 27.0 (CH_3), 15.7 (CH_3), 15.3 (CH_3);

IR (ATR) 2959, 2872, 1728, 1283, 1153 cm^{-1};

HRMS (ESI/TOF) m/z: [M + Na]$^+$ Calcd for $C_{16}H_{30}NaO_3$ 293.2087; Found 293.2080.

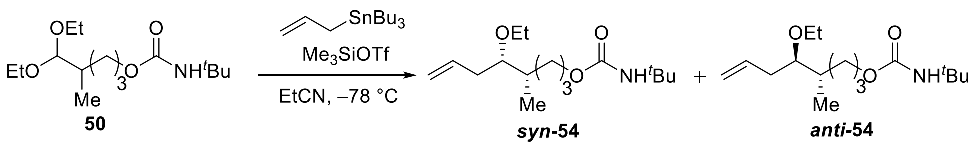

(4*R,5*R**)-5-Ethoxy-4-methyloct-7-en-1-yl *tert*-butylcarbamate (*syn*-54) and (4*R**,5*S**)-5-ethoxy-4-methyloct-7-en-1-yl *tert*-butylcarbamate (*anti*-54)**. Following the general procedure for acetal substitution, acetal **50** (0.026 g, 0.09 mmol) in EtCN (0.9 mL) was mixed with allyltributylstannane (0.11 mL, 0.36 mmol) and trimethylsilyl trifluoromethanesulfonate (0.033 mL, 0.18 mmol) to provide a 60:40 mixture of diastereomers of ester **54**. Ester **54** was isolated as a 59:41 mixture of ***syn*-54**:***anti*-54** with some stannane impurities as a colorless oil (0.025 g, 97%):

^{1}H NMR (400 MHz, $CDCl_3$) δ 5.91–5.77 (m, 1.7H), 5.10–4.99 (m, 3.4H), 4.58 (s, 1.7H), 4.00–3.97 (m, 3.4H), 3.58–3.40 (m, 3.4H), 3.13 (ddd, *J* = 7.0, 5.4, 4.3 Hz, 1H), 3.09 (ddd, *J* = 6.9, 5.6, 4.6 Hz, 0.7H), 2.29–2.18 (m, 3.4H), 1.68–1.60 (m, 8.5H), 1.31 (s, 15.3H), 1.17 (t, *J* = 7.0 Hz, 2.1H), 1.16 (t, *J* = 7.0 Hz, 3H), 0.90–0.86 (m, 5.1H);

Peaks attributed to major diastereomer (***syn*-54**): ^{13}C NMR (100 MHz, $CDCl_3$) δ 155.1 (C overlapping with ***anti*-54**), 136.1 (CH), 116.5 (CH_2), 83.0 (CH), 65.6 (CH_2), 64.6 (CH_2), 50.4 (C), 35.9 (CH), 35.8 (CH_2), 29.13 (CH_3), 28.0 (CH_2 overlapping with ***anti*-54** as determined by HSQC), 27.1 (CH_2), 15.8 (CH_3), 14.7 (CH_3);

Peaks attributed to minor diastereomer (***anti*-54**): ^{13}C NMR (100 MHz, $CDCl_3$) δ 155.1 (C overlapping with ***syn*-54**), 136.0 (CH), 116.4 (CH_2), 83.3 (CH), 65.3 (CH_2), 64.5 (CH_2), 53.6 (C), 35.8 (CH), 35.2 (CH_2), 29.06 (CH_3), 28.0 (CH_2 overlapping with ***syn*-54** as determined by HSQC), 27.0 (CH_2), 15.7 (CH_3), 15.2 (CH_3);

IR (ATR) 3348 (br), 2958, 1701, 1267, 1089 cm^{-1};

HRMS (ESI/TOF) m/z: $[M + Na]^+$ Calcd for $C_{16}H_{31}NNaO_3$ 308.2196; Found 308.2184.

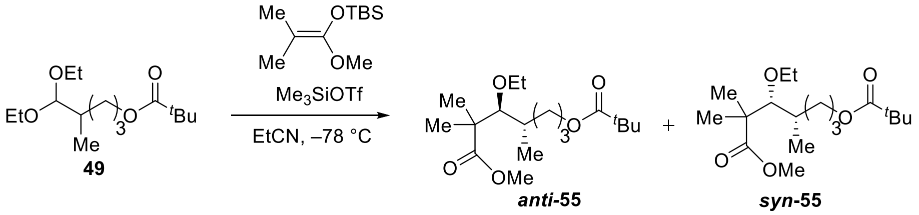

Methyl (3*R,4*R**)-3-ethoxy-2,2,4-trimethyl-7-(pivaloyloxy)heptanoate (*anti*-55) and methyl (3*R**,4*S**)-3-ethoxy-2,2,4-trimethyl-7-(pivaloyloxy)heptanoate (*syn*-55)**. Following the general procedure for acetal substitution, acetal **49** (0.073 g, 0.27 mmol) in EtCN (2.7 mL) was mixed with silyl ketene acetal *tert*-butyl((1-methoxy-2-methylprop-1-en-1-yl)oxy)dimethylsilane (0.26 mL, 1.1 mmol) and trimethylsilyl trifluoromethanesulfonate (0.098 mL, 0.54 mmol) to provide a 71:29 mixture of diastereomers of ester **55**. Ester **55** was isolated as a 71:29 mixture of ***anti*-55**:***syn*-55** as a colorless oil (0.076 g, 85%):

^{1}H NMR (400 MHz, $CDCl_3$) δ 4.04–4.00 (m, 2.8H), 3.64 (s, 4.2H), 3.62–3.50 (m, 1.8H), 3.46 (dq, *J* = 8.9, 7.0 Hz, 1H), 3.38 (d, *J* = 3.0 Hz, 1H), 3.33 (d, *J* = 5.6 Hz, 0.4H), 1.68–1.49 (m, 4.2H),

1.48–1.33 (m, 1.4H), 1.30 (s, 1.4H), 1.19–1.18 (m, 16.8H), 1.14–1.09 (m, 8.4H), 0.91 (d, *J* = 6.8 Hz, 3H), 0.87 (d, *J* = 7.1 Hz, 1.2H);

Peaks attributed to major diastereomer (***anti*-55**): ^{13}C NMR (100 MHz, $CDCl_3$) δ 178.68 (C), 177.9 (C), 87.0 (CH), 69.1 (CH_2), 64.5 (CH_2), 51.72 (CH_3), 48.5 (C), 38.8 (C overlapping with ***syn*-55**), 35.0 (CH), 34.1 (CH_2), 27.3 (CH_3 overlapping with ***syn*-55** as determined by HSQC), 27.0 (CH_2), 22.5 (CH_3), 20.7 (CH_3), 15.8 (CH_3), 15.4 (CH_3);

Peaks attributed to minor diastereomer (***syn*-55**): ^{13}C NMR (100 MHz, $CDCl_3$) δ 178.74 (C), 178.0 (C), 88.1 (CH), 69.5 (CH_2), 64.9 (CH_2), 51.69 (CH_3), 48.1 (C), 38.8 (C overlapping with ***anti*-55**), 35.8 (CH), 28.8 (CH_2), 27.3 (CH_3 overlapping with ***anti*-55** as determined by HSQC), 26.8 (CH_2), 23.6 (CH_3), 19.5 (CH_3), 18.9 (CH_3), 15.7 (CH_3);

IR (ATR) 2973, 1726, 1283, 1148, 1110 cm^{-1};

HRMS (ESI/TOF) m/z: $[M + Na]^+$ Calcd for $C_{18}H_{34}NaO_5$ 353.2298; Found 353.2304.

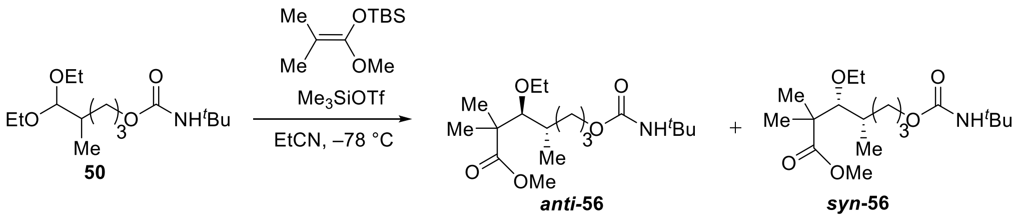

Methyl (3*R,4*R**)-7-((*tert*-butylcarbamoyl)oxy)-3-ethoxy-2,2,4-trimethylheptanoate (*anti*-56) and methyl (3*R**,4*S**)-7-((*tert*-butylcarbamoyl)oxy)-3-ethoxy-2,2,4-trimethylheptanoate (*syn*-56)**. Following the general procedure for acetal substitution, acetal **50** (0.021 g, 0.073 mmol) in EtCN (0.73 mL) was mixed with silyl ketene acetal *tert*-butyl((1-methoxy-2-methylprop-1-en-1-yl)oxy)dimethylsilane (0.70 mL, 0.29 mmol) and trimethylsilyl trifluoromethanesulfonate (0.026 mL, 0.15 mmol) to provide a 68:32 mixture of diastereomers of ester **56**. Ester **56** was isolated as a 67:33 mixture of ***anti*-56**:***syn*-56** as a colorless oil (0.015 g, 59%):

^{1}H NMR (400 MHz, $CDCl_3$) δ 4.62 (s, 1H), 4.59 (s, 0.5H), 4.00–3.95 (m, 3H), 3.65 (s, 4.5H), 3.63–3.41 (m, 3H), 3.39 (d, *J* = 2.7 Hz, 1H), 3.34 (d, *J* = 5.5 Hz, 0.5H), 1.71–1.34 (m, 7.5H), 1.31 (s, 13.5H), 1.20 (s, 1.5H), 1.18 (s, 3H), 1.16–1.10 (m, 9H), 0.92 (d, *J* = 6.8 Hz, 3H), 0.87 (d, *J* = 6.9 Hz, 1.5H);

Peaks attributed to major diastereomer (***anti*-56**): ^{13}C NMR (100 MHz, $CDCl_3$) δ 178.0 (C), 155.2 (C overlapping with ***syn*-56**), 87.1 (CH), 69.1 (CH_2), 64.3 (CH_2), 51.74 (CH_3), 50.4 (C), 48.6 (C), 35.0 (CH), 34.2 (CH_2), 29.1 (CH_3 overlapping with ***syn*-56** as determined by HSQC), 27.1 (CH_2), 22.5 (CH_3), 20.7 (CH_3), 15.8 (CH_3), 15.4 (CH_3);

Peaks attributed to minor diastereomer (***syn*-56**): ^{13}C NMR (100 MHz, $CDCl_3$) δ 178.1 (C), 155.2 (C overlapping with ***anti*-56**), 88.2 (CH), 69.5 (CH_2), 64.7 (CH_2), 51.71 (CH_3), 50.3 (C), 48.1 (C), 35.9 (CH), 29.1 (CH_3 overlapping with ***anti*-56** as determined by HSQC), 28.8 (CH_2), 27.3 (CH_2), 23.7 (CH_3), 19.5 (CH_3), 18.9 (CH_3), 15.7 (CH_3);

IR (ATR) 3374 (br), 2971, 1722, 1265, 1084 cm^{-1};

HRMS (ESI/TOF) m/z: [M + Na]+ Calcd for $C_{18}H_{35}NNaO_5$ 368.2407; Found 368.2427.

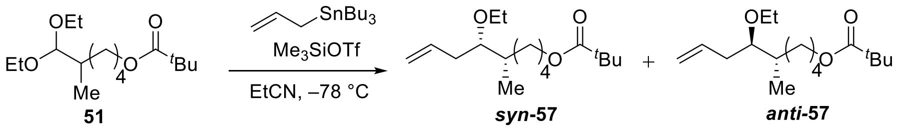

(5*R,6*R**)-6-Ethoxy-5-methylnon-8-en-1-yl pivalate (*syn*-57) and (5*R**,6*S**)-6-ethoxy-5-methylnon-8-en-1-yl pivalate (*anti*-57).** Following the general procedure for acetal substitution, acetal **51** (0.043 g, 0.15 mmol) in EtCN (1.5 mL) was mixed with allyltributylstannane (0.19 mL, 0.60 mmol) and trimethylsilyl trifluoromethanesulfonate (0.054 mL, 0.30 mmol) to provide a 64:36 mixture of diastereomers of ester **57**. Ester **57** was isolated as a 58:42 mixture of ***syn*-57**:***anti*-57** with some stannane impurities as a colorless oil (0.037 g, 87%):

^{1}H NMR (400 MHz, $CDCl_3$) δ 5.59–5.77 (m, 1.7H), 5.11–4.98 (m, 3.4H), 4.05 (t, *J* = 6.6 Hz, 3.4H), 3.58–3.40 (m, 3.4H), 3.14–3.06 (m, 1.7H), 2.29–2.14 (m, 3.4H), 1.68–1.58 (m, 8.5H), 1.50–1.41 (m, 3.4H), 1.19 (s, 15.3H), 1.17–1.14 (m, 5.1H), 0.93–0.90 (m, 5.1H);

Peaks attributed to major diastereomer (***syn*-57**): ^{13}C NMR (100 MHz, $CDCl_3$) δ 178.75 (C), 136.1 (CH), 116.40 (CH_2), 83.0 (CH), 65.6 (CH_2), 64.46 (CH_2), 38.9 (C, overlapping with ***anti*-57**), 36.0 (CH), 35.90 (CH_2), 32.4 (CH_2), 29.0 (CH_2), 27.34 (CH_3), 23.844 (CH_2), 15.74 (CH_3), 14.7 (CH_3);

Peaks attributed to minor diastereomer (***anti*-57**): ^{13}C NMR (100 MHz, $CDCl_3$) δ 178.76 (C), 136.0 (CH), 116.37 (CH_2), 83.3 (CH), 65.2 (CH_2), 64.48 (CH_2), 38.9 (C, overlapping with ***syn*-57**), 35.93 (CH), 35.2 (CH_2), 32.2 (CH_2), 29.1 (CH_2), 27.33 (CH_3), 23.836 (CH_2), 15.70 (CH_3), 15.1 (CH_3);

IR (ATR) 2972, 2871, 1728, 1284, 1152 cm^{-1};

HRMS (ESI/TOF) m/z: [M + Na]+ Calcd for $C_{17}H_{32}NaO_3$ 307.2244; Found 307.2239.

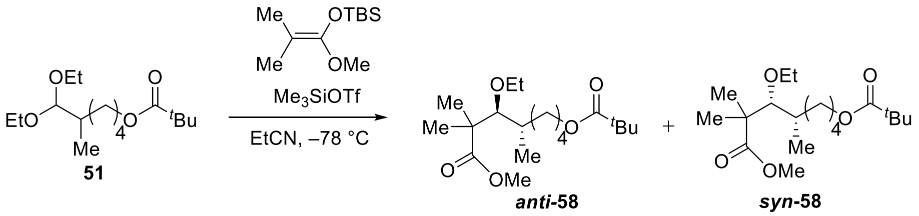

Methyl (3*R,4*R**)-3-ethoxy-2,2,4-trimethyl-8-(pivaloyloxy)octanoate (*anti*-58) and methyl (3*R**,4*S**)-3-ethoxy-2,2,4-trimethyl-8-(pivaloyloxy)octanoate (*syn*-58)**. Following the general procedure for acetal substitution, acetal **51** (0.043 g, 0.15 mmol) in EtCN (1.5 mL) was mixed with silyl ketene acetal *tert*-butyl((1-methoxy-2-methylprop-1-en-1-yl)oxy)dimethylsilane (0.14 mL, 0.60 mmol) and trimethylsilyl trifluoromethanesulfonate (0.054 mL, 0.30 mmol) to provide a 75:25 mixture of diastereomers of ester **58**. Ester **58** was isolated as a 75:25 mixture of ***anti*-58**:***syn*-58** as a colorless oil (0.043 g, 83%):

^{1}H NMR (400 MHz, $CDCl_3$) δ 4.05 (t, *J* = 6.6 Hz, 0.6H), 4.04 (t, *J* = 6.6 Hz, 2H), 3.65 (s, 3.9H), 3.63–3.41 (m, 2.6H), 3.38 (d, *J* = 3.0 Hz, 1H), 3.33 (d, *J* = 5.6 Hz, 0.3H), 1.70–1.46 (m, 5.2H),

1.40–1.27 (m, 3.9H), 1.20–1.17 (m, 15.6H), 1.15–1.09 (m, 7.8H), 0.90 (d, J = 6.8 Hz, 3H), 0.86 (d, J = 7.0 Hz, 0.9H);

Peaks attributed to major diastereomer (***anti*-58**): ^{13}C NMR (100 MHz, $CDCl_3$) δ 178.7 (C), 177.9 (C), 87.1 (CH), 69.0 (CH_2), 64.3 (CH_2), 51.71 (CH_3), 48.6 (C), 38.9 (C), 37.6 (CH_2 overlapping with ***syn*-58** as determined by HSQC), 35.1 (CH), 28.9 (CH_2), 27.3 (CH_3 overlapping with ***syn*-58** as determined by HSQC), 24.1 (CH_2), 22.4 (CH_3), 20.8 (CH_3), 15.8 (CH_3), 15.4 (CH_3);

Peaks attributed to minor diastereomer (***syn*-58**): ^{13}C NMR (100 MHz, $CDCl_3$) δ 178.8 (C), 178.1 (C), 88.3 (CH), 69.5 (CH_2), 64.5 (CH_2), 51.69 (CH_3), 48.1 (C), 37.6 (CH_2 overlapping with ***syn*-58** as determined by HSQC), 36.1 (CH), 32.1 (C), 29.2 (CH_2), 27.3 (CH_3 overlapping with ***anti*-58** as determined by HSQC), 24.0 (CH_2), 23.7 (CH_3), 19.5 (CH_3), 18.9 (CH_3), 15.7 (CH_3);

IR (ATR) 2973, 1726, 1283, 1149, 1112 cm^{-1};

HRMS (ESI/TOF) m/z: $[M + Na]^+$ Calcd for $C_{19}H_{36}NaO_5$ 367.2455; Found 367.2459.

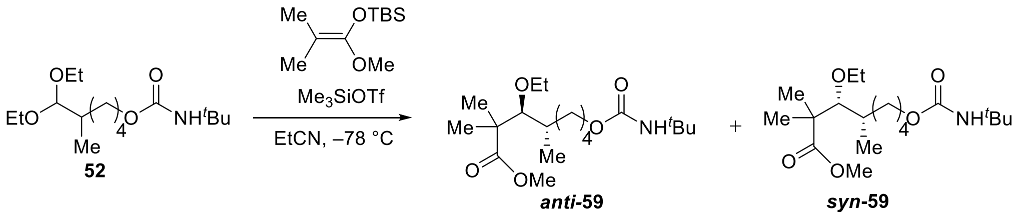

Methyl (3*R,4*S**)-8-((*tert*-butylcarbamoyl)oxy)-3-ethoxy-2,2,4-trimethyloctanoate (*anti*-59) and methyl (3*R**,4*R**)-8-((*tert*-butylcarbamoyl)oxy)-3-ethoxy-2,2,4-trimethyloctan (*syn*-59).** Following the general procedure for acetal substitution, acetal **52** (0.024 g, 0.079 mmol) in EtCN (0.80 mL) was mixed with silyl ketene acetal *tert*-butyl((1-methoxy-2-methylprop-1-en-1-yl)oxy)dimethylsilane (0.078 mL, 0.32 mmol) and trimethylsilyl trifluoromethanesulfonate (0.029 mL, 0.16 mmol) to provide an 81:19 mixture of diastereomers of ester **59**. Ester **59** was isolated as an 81:19 mixture of ***anti*-59**:***syn*-59** as a colorless oil (0.025 g, 88%):

^1H NMR (400 MHz, $CDCl_3$) δ 4.64 (s, 1H), 4.60 (s, 0.2H) 3.99 (t, J = 6.7 Hz, 2.4H), 3.65 (s, 3.6H), 3.63–3.56 (m, 1.2H), 3.54–3.50 (m, 0.2H), 3.45 (dq, J = 9.0, 7.0 Hz, 1H), 3.37 (d, J = 3.2 Hz, 1H), 3.33 (d, J = 5.6 Hz, 0.2H), 1.63–1.48 (m, 4H), 1.38–1.24 (s, 15.2H), 1.20 (s, 0.6H), 1.18 (s, 3H), 1.13 (t, J = 7.0 Hz, 3.6H), 1.10 (s, 3.6H), 0.90 (d, J = 6.8 Hz, 3H), 0.85 (d, J = 7.0 Hz, 0.6H);

Peaks attributed to major diastereomer (***anti*-59**): ^{13}C NMR (100 MHz, $CDCl_3$) δ 178.0 (C), 155.3 (C), 87.2 (CH), 69.1 (CH_2), 64.3 (CH_2), 51.72 (CH_3), 50.4 (C), 48.6 (C), 37.6 (CH_2), 35.2 (CH), 29.3 (CH_2), 29.1 (CH_3), 24.2 (CH_2), 22.5 (CH_3), 20.7 (CH_3), 15.8 (CH_3), 15.4 (CH_3);

Peaks attributed to minor diastereomer (***syn*-59**): ^{13}C NMR (100 MHz, $CDCl_3$, characteristic peaks) δ 178.1 (C), 88.3 (CH), 69.5 (CH_2), 64.4 (CH_2), 51.69 (CH_3), 48.1 (C), 36.1 (CH_2), 32.2 (CH), 29.6 (CH_2), 24.1 (CH_2), 23.8 (CH_3), 19.5 (CH_3), 18.9 (CH_3), 15.7 (CH_3);

IR (ATR) 3374 (br), 2970, 1727, 1265, 1084 cm^{-1};

HRMS (ESI/TOF) m/z: $[M + Na]^+$ Calcd for $C_{19}H_{37}NNaO_5$ 382.2564; Found 382.2572.

Scheme 5: Synthesis of δ-acyloxy acetals bearing a β-phenyl substituent

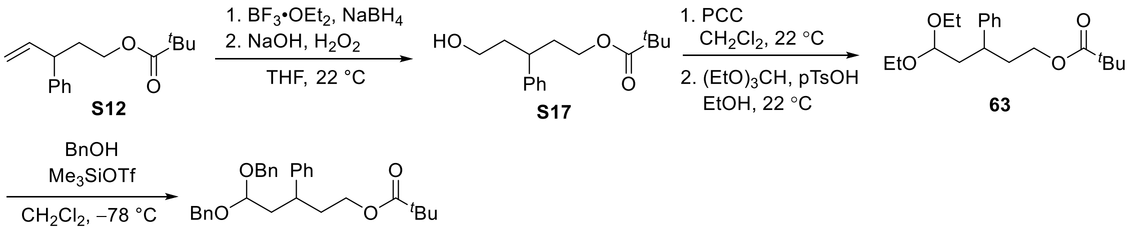

64

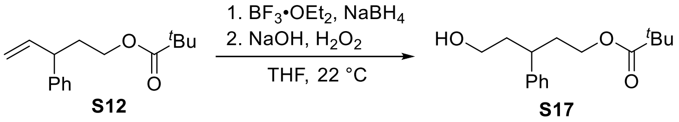

5-Hydroxy-3-phenylpentyl pivalate (S17). To a cooled (0 ºC) solution of ester **S12** (7.1 g, 29 mmol) in THF (290 mL) was added sodium borohydride (1.4 g, 38 mmol). After 15 min, BF_3•OEt_2 (4.0 mL, 38 mmol) was added dropwise over 30 min. The reaction mixture was warmed to 22 ºC over 30 min and stirred for an additional 16 h. The mixture was cooled to 0 °C and NaOH (32 mL, 32 mol, 1.0 M solution in H_2O) and H_2O_2 (32 mL, 0.032 mol, 50% solution in H_2O) were added successively over 1 h using an addition funnel. The mixture was then allowed to warm to 22 °C over 1 h and stirred for an additional 3 h. H_2O (50 mL) was added, and the aqueous layer was extracted with EtOAc (2 x 50 mL). The combined organic layers were washed with brine (60 mL) and dried over anhydrous Na_2SO_4. The resulting mixture was filtered, concentrated *in vacuo*, and purified by column chromatography (20:80 EtOAc:hexanes) to provide the alcohol **S17** as a colorless oil (6.03 g, 79%):

^{1}H NMR (400 MHz, $CDCl_3$) δ 7.33–7.27 (m, 2H), 7.23–7.14 (m, 3H), 3.98 (ddd, *J* = 10.9, 6.9, 5.4 Hz, 1H), 3.84 (ddd, *J* = 10.9, 7.8, 6.5 Hz, 1H), 3.54 (ddd, *J* = 10.6, 6.7, 5.4 Hz, 1H), 3.46 (ddd, *J* = 10.6, 7.6, 6.3 Hz, 1H), 2.86 (tt, *J* = 10.0, 5.2 Hz, 1H), 2.08–1.79 (m, 4H), 1.38 (s, 1H), 1.17 (s, 9H);

^{13}C NMR (100 MHz, $CDCl_3$) δ 178.7 (C), 143.8 (C), 128.8 (CH), 127.7 (CH), 126.7 (CH), 62.7 (CH_2), 61.0 (CH_2), 39.5 (CH_2), 39.2 (CH), 38.9 (C), 35.7 (CH_2), 27.3 (CH_3);

IR (ATR) 3437 (br), 3027, 2959, 1725, 1283 cm^{-1};

HRMS (ESI/TOF) m/z: [M + Na]$^+$ Calcd for $C_{16}H_{24}NaO_3$ 287.1623; Found 287.1621.

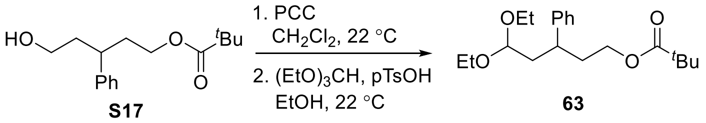

5,5-Diethoxy-3-phenylpentyl pivalate (63). To a solution of pyridinium chlorochromate (11 g, 48 mmol) and crushed 4Å molecular sieves (10 g) in CH_2Cl_2 (50 mL) was added a 0.25 M solution of alcohol **S17** (4.8 g, 16 mol, in 65 mL of CH_2Cl_2). After 4 h, the mixture was concentrated in vacuo and then resuspended in Et_2O (200 mL). This solution was filtered over a pad of

diatomaceous earth to provide the corresponding aldehyde (1.8 g, 44%), which was used directly in the next step without further purification.

Following the general procedure for diethyl acetal formation, the crude aldehyde (1.8 g, 6.9 mmol) in triethyl orthoformate (12 mL, 69 mmol) was mixed with ethanol (1.0 mL) and *p*-toluenesulfonic acid (0.67 g, 3.5 mmol) to provide acetal **63** as a colorless oil (1.85 g, 84%):

^{1}H NMR (400 MHz, $CDCl_3$) δ 7.31–7.27 (m, 2H), 7.25–7.10 (m, 3H), 4.19 (dd, *J* = 8.0, 3.9 Hz, 1H), 3.99–3.92 (m, 1H), 3.81 (ddd, *J* = 10.9, 8.0, 6.3 Hz, 1H), 3.61 (dq, *J* = 9.2, 7.0 Hz, 1H), 3.56–3.43 (m, 1H), 3.41–3.33 (m, 2H), 2.87 (spt, *J* = 5.1 Hz, 1H), 2.15–1.94 (m, 2H), 1.92–1.83 (m, 2H), 1.20 (t, *J* = 7.4 Hz, 3H), 1.18 (s, 9H), 1.11 (t, *J* = 7.0 Hz, 3H);

^{13}C NMR (100 MHz, $CDCl_3$) δ 178.6 (C), 143.8 (C), 128.7 (CH), 127.8 (CH), 126.6 (CH), 101.2 (CH), 62.6 (CH_2), 61.4 (CH_2), 61.0 (CH_2), 40.6 (CH_2), 38.8 (C), 38.7 (CH), 35.7 (CH_2), 27.3 (CH_3), 15.5 (two CH_3 overlapping, as determined by HSQC);

IR (ATR) 2973, 1726, 1480, 1283, 1152 cm^{-1};

HRMS (ESI/TOF) m/z: $[M + Na]^+$ Calcd for $C_{20}H_{32}NaO_4$ 359.2198; Found 359.2211.

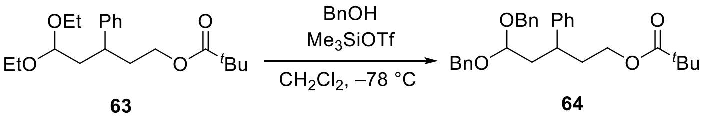

5,5-Bis(benzyloxy)-3-phenylpentyl pivalate (64). Following the general procedure for dibenzyl acetal formation, acetal **63** (1.0 g, 2.9 mmol) in CH_2Cl_2 (30 mL) was mixed with benzyl alcohol (1.6 mL, 15 mmol) and trimethylsilyl trifluoromethanesulfonate (1.1 mL, 4.9 mmol) to provide dibenzyl acetal **64** as a colorless oil (1.15 g, 83%):

^{1}H NMR (400 MHz, $CDCl_3$) δ 7.61–7.28 (m, 8H), 7.25–7.14 (m, 5H), 7.08–6.98 (m, 2H), 4.63 (d, *J* = 11.6 Hz, 1H), 4.51–4.43 (m, 3H), 4.40 (d, *J* = 11.6 Hz, 1H), 3.99–3.90 (m, 1H), 3.88–3.72 (m, 1H), 2.90 (spt, *J* = 5.0 Hz, 1H), 2.15 (ddd, *J* = 13.3, 8.0, 2.9 Hz, 1H), 2.08–1.91 (m, 2H), 1.91–1.82 (m, 1H), 1.15 (s, 9H);

^{13}C NMR (100 MHz, $CDCl_3$) δ 178.6 (C), 143.5 (C), 138.3 (C), 138.2 (C), 128.8 (CH), 128.6 (CH), 128.5 (CH), 128.1 (CH), 127.9 (CH), 127.8 (CH), 127.7 (CH), 127.6 (CH), 126.7 (CH), 100.7 (CH), 68.0 (CH_2), 67.2 (CH_2), 62.6 (CH_2), 40.5 (CH_2), 38.8 (C), 38.7 (CH), 35.6 (CH_2), 27.3 (CH_3);

IR (ATR) 3029, 2957, 1724, 1282, 1151 cm^{-1};

HRMS (ESI/TOF) m/z: $[M + Na]^+$ Calcd for $C_{30}H_{36}NaO_4$ 483.2511; Found 483.2515.

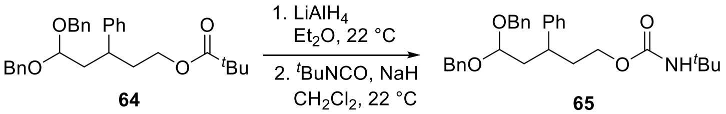

5,5-Bis(benzyloxy)-3-phenylpentyl *tert*-butylcarbamate (65). Following the general procedure for ester reduction, ester **64** (0.57 g, 1.2 mmol) in Et_2O (17 mL) was mixed with lithium aluminum

hydride (0.097 g, 2.4 mmol) to provide the crude alcohol (0.42 g, 93%), which was used in the next step without further purification.

Following the general procedure for carbamate formation, the crude alcohol (0.42 g, 1.1 mmol) in THF (16 mL) was mixed with NaH (0.19 g, 60% dispersion in mineral oil, 4.4 mmol) and *tert*-butyl isocyanate (0.49 mL, 3.3 mmol) to provide carbamate **65** as a colorless oil (0.37 g, 71%):

^{1}H NMR (400 MHz, $CDCl_3$) δ 7.37–7.28 (m, 9H), 7.25–7.07 (m, 6H), 4.64 (dd, *J* = 11.6, 1.8 Hz, 1H), 4.56–4.45 (m, 4H), 4.41 (dd, *J* = 11.6, 1.8 Hz, 1H), 3.94–3.84 (m, 1H), 3.83–3.76 (m, 1H), 2.90–2.85 (m, 1H), 2.21–2.13 (m, 1H), 2.06–1.81 (m, 3H), 1.28 (s, 9H);

^{13}C NMR (100 MHz, $CDCl_3$) δ 155.0 (C), 143.8 (C), 138.31 (C), 138.26 (C), 128.7 (CH), 128.6 (CH), 128.5 (CH), 128.1 (CH), 127.9 (CH), 127.8 (CH), 127.74 (CH), 127.69 (CH), 126.6 (CH), 100.7 (CH), 67.8 (CH_2), 67.3 (CH_2), 62.4 (CH_2), 50.3 (C), 40.4 (CH_2), 38.8 (CH), 36.1 (CH_2), 29.1 (CH_3);

IR (ATR) 3348 (br), 2955, 1705, 1496, 1268 cm^{-1};

HRMS (ESI/TOF) m/z: [M + Na]$^+$ Calcd for $C_{30}H_{37}NNaO_4$ 498.2615; Found 498.2591.

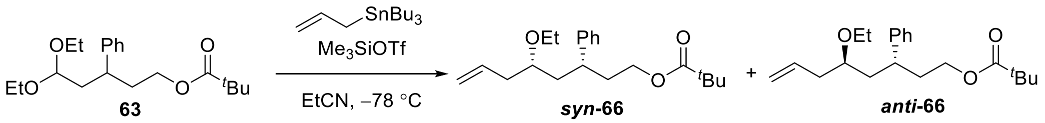

(3*R,5*S**)-5-Ethoxy-3-phenyloct-7-en-1-yl pivalate (*syn*-66) and (3*R**,5*R**)-5-ethoxy-3-phenyloct-7-en-1-yl pivalate (*anti*-66).** Following the general procedure for acetal substitution, acetal **63** (0.34 g, 1.0 mmol) in EtCN (10 mL) was mixed with allyltributylstannane (1.2 mL, 4.0 mmol) and trimethylsilyl trifluoromethanesulfonate (0.36 mL, 2.0 mmol) to provide an 88:12 mixture of diastereomers of ester **66**. Ester **66** was isolated as a 70:30 mixture of ***syn*-66**:***anti*-66** as a colorless oil (0.262 g, 79%):

^{1}H NMR (400 MHz, $CDCl_3$) δ 7.31–7.27 (m, 2.8H), 7.25–7.09 (m, 4.2H), 5.87–5.62 (m, 1.4H), 5.10–4.95 (m, 2.8H), 3.94 (dtd, *J* = 11.8, 6.8, 5.0 Hz, 1.4H), 3.79 (dtd, *J* = 11.0, 8.3, 6.3 Hz, 1.4H), 3.52 (dq, *J* = 7.7, 6.4 Hz, 0.4H), 3.49–3.36 (m, 1H), 3.32 (dq, *J* = 9.1, 7.0 Hz, 1H), 3.25–3.10 (m, 1.4H), 3.08–2.89 (m, 0.8H), 2.89–2.77 (m, 1H), 2.30–2.15 (m, 2.8H), 2.08–1.71 (m, 5.6H), 1.18 (s, 9H), 1.17 (s, 3.6H), 1.12–1.10 (m, 1.2H), 1.10 (t, *J* = 7.0 Hz, 3H);

Peaks attributed to major diastereomer (***syn*-66**): ^{13}C NMR (100 MHz, $CDCl_3$) δ 178.6 (C overlapping with ***anti*-99**), 144.4 (C), 134.9 (CH), 128.71 (CH), 127.7 (CH), 126.53 (CH), 117.1 (CH_2), 76.5 (CH), 64.2 (CH_2), 62.7 (CH_2), 41.1 (CH_2), 38.9 (CH), 38.84 (C), 38.2 (CH_2), 35.4 (CH_2), 27.3 (CH_3), 15.6 (CH_3);

Peaks attributed to minor diastereomer (***anti*-66**): ^{13}C NMR (100 MHz, $CDCl_3$) δ 178.6 (C overlapping with ***syn*-66**), 144.2 (C), 134.8 (CH), 128.66 (CH), 128.0 (CH), 126.49 (CH), 117.2 (CH_2), 76.2 (CH), 64.4 (CH_2), 62.8 (CH_2), 41.8 (CH_2), 39.0 (CH), 38.83 (C), 38.76 (CH_2), 36.3 (CH_2), 31.1 (CH_3), 15.8 (CH_3);

IR (ATR) 2958, 2929, 2871, 1726, 1283 cm^{-1};

HRMS (ESI/TOF) m/z: [M + Na]+ Calcd for $C_{21}H_{32}NaO_3$ 355.2249; Found 355.2259.

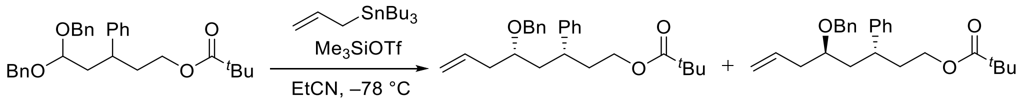

64 *syn*-**67** *anti*-**67**

(3*R,5*S**)-5-(Benzyloxy)-3-phenyloct-7-en-1-yl pivalate (*syn*-67) and (3*R**,5*R**)-5-(benzyloxy)-3-phenyloct-7-en-1-yl pivalate (*anti*-67)**. Following the general procedure for acetal substitution, acetal **64** (0.040 g, 0.087 mmol) in EtCN (0.90 mL) was mixed with allyltributylstannane (0.11 mL, 0.35 mmol) and trimethylsilyl trifluoromethanesulfonate (0.031 mL, 0.17 mmol) to provide a 58:42 mixture of diastereomers of ester **67**. Ester **67** was isolated as a 58:42 mixture of ***syn*-67**:***anti*-67** with some stannane impurities as a colorless oil (0.035 g, quantitative):

^{1}H NMR (400 MHz, $CDCl_3$) δ 7.37–7.26 (m, 8.3H), 7.26–7.05 (m, 8.7H), 5.88–5.64 (m, 1.7H), 5.12–4.96 (m, 3.4H), 4.52 (d,*J* = 11.2 Hz, 0.7H), 4.44 (d, *J* = 11.6 Hz, 1H), 4.36 (d, *J* = 11.6 Hz, 1H), 4.20 (d, *J* = 11.2 Hz, 0.7H), 3.98–3.89 (m, 1.7H), 3.81–3.73 (m, 1.7H), 3.37–3.30 (m, 1H), 3.18–3.01 (m, 1.4H), 2.82 (dddd, *J* = 10.7, 8.3, 6.4, 4.7 Hz, 1H), 2.40–2.16 (m, 3.4H), 2.03–1.86 (m, 3.4H), 1.86–1.77 (m, 3.4H), 1.16–1.14 (m, 15.3H);

Peaks attributed to major diastereomer (***syn*-67**): ^{13}C NMR (100 MHz, $CDCl_3$) δ 178.56 (C), 144.5 (C), 138.8 (C), 134.7 (CH), 128.8 (CH), 128.69 (CH), 128.4 (CH), 127.9 (CH), 127.7 (CH), 126.6 (CH), 117.3 (CH_2), 76.1 (CH overlapping with ***anti*-67**, as determined by HSQC), 70.8 (CH_2), 62.7 (CH_2), 41.0 (CH_2), 38.98 (CH), 38.8 (C), 37.99 (CH_2 overlapping with ***anti*-67**, as determined by HSQC), 35.4 (CH_2), 27.3 (CH_3 overlapping with ***anti*-67**, as determined by HSQC);

Peaks attributed to minor diastereomer (***anti*-67**): ^{13}C NMR (100 MHz, $CDCl_3$) δ 178.60 (C), 144.1 (C), 138.9 (C), 134.5 (CH), 128.71 (CH), 128.5 (CH), 127.96 (CH), 127.95 (CH), 127.6 (CH), 126.5 (CH), 117.4 (CH_2), 76.1 (CH overlapping with ***syn*-67**, as determined by HSQC), 71.2 (CH_2), 62.8 (CH_2), 41.8 (CH_2), 38.95 (CH), 38.5 (C), 37.99 (CH_2 overlapping with ***syn*-67**, as determined by HSQC), 36.3 (CH_2), 27.3 (CH_3 overlapping with ***syn*-67**, as determined by HSQC);

IR (ATR) 2957, 2871, 1727, 1154, 698 cm^{-1};

HRMS (ESI/TOF) m/z: [M + Na]+ Calcd for $C_{26}H_{34}NaO_3$ 417.2400; Found 417.2382.

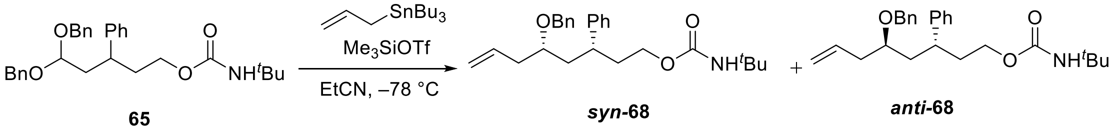

65 *syn*-**68** *anti*-**68**

(3*R,5*S**)-5-(Benzyloxy)-3-phenyloct-7-en-1-yl *tert*-butylcarbamate (*syn*-68) and (3*R**,5*R**)-5-(benzyloxy)-3-phenyloct-7-en-1-yl *tert*-butylcarbamate**. Following the general procedure for acetal substitution, acetal **65** (0.056 g, 0.12 mmol) in EtCN (1.2 mL) was mixed with allyltributylstannane (0.15 mL, 0.48 mmol) and trimethylsilyl trifluoromethanesulfonate (0.043 mL, 0.24 mmol) to provide a 65:35 mixture of diastereomers of ester **68**. Ester **68** was isolated as a 65:35 mixture of ***syn*-68**:***anti*-68** with some stannane impurities as a colorless oil (0.036 g, 74%):

^{1}H NMR (400 MHz, $CDCl_3$) δ 7.38–7.26 (m, 8H), 7.26–7.12 (m, 7H), 7.09–7.07 (m, 1H), 5.89–5.64 (m, 1.6H), 5.11–4.99 (m, 3.2H), 4.52–4.42 (m, 3.2H), 4.36 (d, *J* = 11.6 Hz, 1H), 4.20 (d, *J* = 11.2 Hz, 0.6H), 3.91–3.82 (m, 1.6H), 3.82–3.72 (m, 1.6H), 3.36–3.33 (m, 1H), 3.17–3.09 (m, 0.6H), 3.03–2.98 (m, 0.6H), 2.86–2.76 (m, 1H), 2.38–2.18 (m, 3.2H), 1.98–1.85 (m, 3.2H), 1.85–1.74 (m, 3.2H), 1.28 (s, 14.4H);

Peaks attributed to major diastereomer (***syn*-68**): ^{13}C NMR (100 MHz, $CDCl_3$) δ 155.0 (C), 144.6 (C), 138.8 (C), 134.8 (CH), 128.71 (CH), 128.6 (CH), 128.4 (CH), 127.9 (CH), 127.7 (CH), 126.5 (CH), 117.3 (CH_2), 76.3 (CH), 70.7 (CH_2), 62.6 (CH_2), 50.3 (C), 41.0 (CH_2), 39.08 (CH), 38.1 (CH_2), 35.8 (CH_2), 29.1 (CH_3);

Peaks attributed to minor diastereomer (***anti*-68**): ^{13}C NMR (100 MHz, $CDCl_3$, characteristic peaks) δ 144.3 (C), 138.9 (C), 134.6 (CH), 128.65 (CH), 128.5 (CH), 128.01 (CH), 127.98 (CH), 127.6 (CH), 126.4 (CH), 117.4 (CH_2), 76.2 (CH), 71.1 (CH_2), 41.7 (CH_2), 39.10 (CH), 38.5 (CH_2), 36.8 (CH_2);

IR (ATR) 3349 (br), 2960, 1709, 1265, 1086 cm^{-1};

HRMS (ESI/TOF) m/z: $[M + Na]^+$ Calcd for $C_{26}H_{35}NNaO_3$ 432.2509; Found 432.2492.

Scheme 6: Synthesis of γ-acyloxy acetal bearing a β-methyl substituent

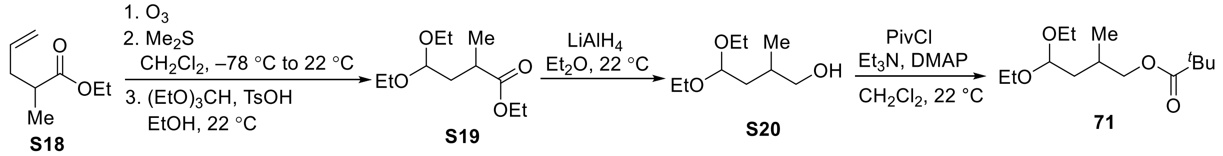

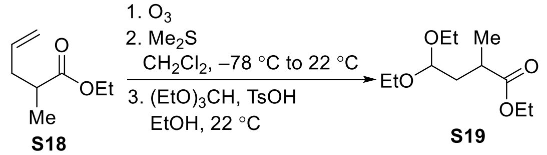

Ethyl 4,4-diethoxy-2-methylbutanoate (S19). Following the general procedure for ozonolysis, alkene **S18** (8.1 mL, 50 mmol) in CH_2Cl_2 (200 mL, 0.25 M) was treated with O_3 and Me_2S (37 mL, 500 mmol) to provide the corresponding aldehyde (7.2 g, quantitative), which was used directly in the next step without further purification.

Following the general procedure for diethyl acetal formation, the crude aldehyde (7.2 g, 50 mmol) in triethyl orthoformate (80 mL, 500 mmol) was mixed with ethanol (5.0 mL) and *p*-toluenesulfonic acid (4.75 g, 0.0250 mol) to provide acetal **S19** as a colorless oil (8.4 g, 77%). The spectroscopic data of acetal **S19** are consistent with literature data:[9]

^{1}H NMR (400 MHz, $CDCl_3$) δ 4.52 (t, *J* = 5.8 Hz, 1H), 4.19–4.07 (m, 2H), 3.72–3.58 (m, 2H), 3.55–3.43 (m, 2H), 2.65–2.51 (m, 1H), 2.16–1.99 (m, 1H), 1.73–1.59 (m, 1H), 1.26 (t, *J* = 7.2 Hz, 3H), 1.21–1.17 (m, 9H);

^{13}C NMR (100 MHz, $CDCl_3$) δ 176.6, 101.4, 61.77, 61.68, 60.4, 37.6, 35.9, 17.8, 15.4, 14.4;

IR (ATR) 2977, 2935, 2882, 1730, 1180 cm^{-1};

HRMS (ESI/TOF) m/z: $[M + Na]^+$ Calcd for $C_{11}H_{22}NaO_4$ 241.1416; Found 241.1421.

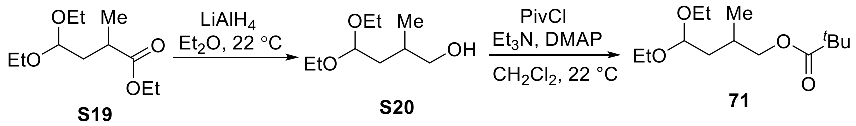

4,4-Diethoxy-2-methylbutyl pivalate (71). Following the general procedure for ester reduction, ester **S19** (3.1 g, 14 mmol) in Et_2O (140 mL) was mixed with lithium aluminum hydride (0.81 g, 21 mmol) to provide alcohol **S20** as a colorless oil (1.89 g, 77%), which was used directly in the next step without further purification.

Following the general procedure for esterification of alcohols, the crude alcohol **S20** (2.5 g, 14 mol) in CH_2Cl_2 (80 mL) was mixed with triethylamine (3.6 mL, 28 mmol), 4-dimethylaminopyridine (0.17 g, 1.4 mmol), and pivaloyl chloride (4.2 mL, 28 mmol) to provide ester **71** as a colorless oil (3.35 g, 92%):

^1H NMR (400 MHz, $CDCl_3$) δ 4.59 (dd, *J* = 6.6, 5.2 Hz, 1H), 3.99–3.84 (m, 2H), 3.63 (dq, *J* = 9.3, 7.0 Hz, 2H), 3.48 (dq, *J* = 9.4, 7.1 Hz, 2H), 1.96 (qd, *J* = 6.2, 1.8 Hz, 1H), 1.79–1.68 (m, 1H), 1.45 (dddd, *J* = 13.7, 6.8, 5.4, 2.7 Hz, 1H), 1.28–1.10 (m, 15H), 0.97 (d, *J* = 6.8 Hz, 3H);

^{13}C NMR (100 MHz, $CDCl_3$) δ 178.7 (C), 101.3 (CH), 69.0 (CH_2), 61.4 (CH_2), 60.7 (CH_2), 39.0 (C), 37.2 (CH_2), 29.3 (CH), 27.4 (CH_3), 27.1 (CH_3), 17.3 (CH_3), 15.5 (CH_3);

IR (ATR) 2971, 1720, 1284, 1151, 1033 cm^{-1};

HRMS (ESI/TOF) m/z: $[M + NH_4]^+$ Calcd for $C_{14}H_{32}NO_4$ 278.2331; Found 278.2338.

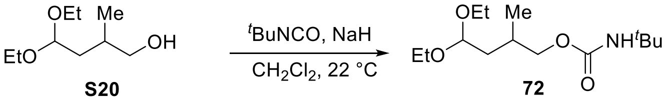

4,4-Diethoxy-2-methylbutyl *tert*-butylcarbamate (72). Following the general procedure for carbamate formation, the crude alcohol **S20** (0.22 g, 1.2 mmol) in THF (12 mL) was mixed with NaH (0.10 g, 60% dispersion in mineral oil, 2.4 mmol) and *tert*-butyl isocyanate (0.20 mL, 1.8 mmol) to provide carbamate **72** as a colorless oil (0.35 g, quantitative):

^1H NMR (400 MHz,$CDCl_3$) δ 4.61–4.58 (m, 2H), 3.91–3.81 (m, 2H), 3.68–3.59 (m, 2H), 3.49 (dq, *J* = 9.4, 7.1 Hz, 2H), 1.94–1.87 (m, 1H), 1.74 (dt, *J* = 14.0, 6.3 Hz, 1H), 1.43 (ddd, *J* = 14.0, 8.0, 5.3 Hz, 1H), 1.31 (s, 9H), 1.20 (t, *J* = 7.0 Hz, 3H), 1.19 (t, *J* = 7.0 Hz, 3H), 0.96 (d, *J* = 6.7 Hz, 3H);

^{13}C NMR (100 MHz, $CDCl_3$) δ 155.2 (C), 101.4 (CH), 68.9 (CH_2), 61.4 (CH_2), 60.8 (CH_2), 50.4 (C), 37.3 (CH_2), 29.5 (CH), 29.1 (CH_3), 17.3 (two CH_3 overlapping, as determined by HSQC), 15.5 (CH_3);

IR (ATR) 3348 (br), 2971, 1708, 1527, 1265 cm^{-1};

HRMS (ESI/TOF) m/z: $[M + K]^+$ Calcd for $C_{14}H_{29}KNO_4$ 314.1728; Found 314.1736.

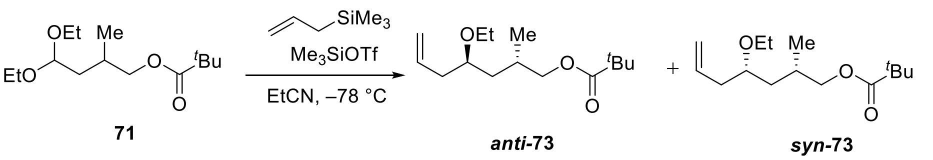

(2*R,4*S**)-4-Ethoxy-2-methylhept-6-en-1-yl pivalate (*anti*-73) and (2*R**,4*R**)-4-ethoxy-2-methylhept-6-en-1-yl pivalate (*syn*-73).** Following the general procedure for acetal substitution, acetal **71** (0.080 g, 0.31 mmol) in EtCN (3.0 mL) was mixed with allyltrimethylsilane (0.20 mL, 1.2 mmol) and trimethylsilyl trifluoromethanesulfonate (0.11 mL, 0.32 mmol) to provide a 50:50 mixture of diastereomers of ester **73**. Ester **73** was isolated as a 50:50 mixture of ***anti*-73**:***syn*-73** as a colorless oil (0.080 g, quantitative):

^{1}H NMR (400 MHz, $CDCl_3$) δ 5.83– 5.72 (m, 2H), 5.10–4.99 (m, 4H), 3.96–3.88 (m, 3H), 3.84 (dd, *J* = 10.7, 6.7 Hz, 1H), 3.63–3.53 (m, 2H), 3.45–3.32 (m, 4H), 2.33–2.17 (m, 4H), 2.06–1.91 (m, 2H), 1.59–1.45 (m, 2H), 1.36 (ddd, *J* = 14.3, 8.2, 6.3 Hz, 1H), 1.27–1.22 (m, 1H), 1.20–1.13 (m, 24H), 0.95 (d, *J* = 6.8 Hz, 3H), 0.93 (d, *J* = 6.7 Hz, 3H);

^{13}C NMR (100 MHz, $CDCl_3$) δ 178.7 (C), 178.6 (C), 134.9 (CH), 134.8 (CH), 117.2 (CH_2), 117.1 (CH_2), 76.9 (CH), 76.4 (CH), 69.6 (CH_2), 68.7 (CH_2), 64.34 (CH_2), 64.25 (CH_2), 38.98 (C), 38.96 (C), 38.9 (CH_2), 38.6 (CH_2), 38.1 (CH_2), 37.9 (CH_2), 29.7 (CH), 29.6 (CH), 27.3 (two CH_3 overlapping, as determined by HSQC), 17.9 (CH_3), 16.9 (CH_3), 15.7 (two CH_3 overlapping, as determined by HSQC);

IR (ATR) 2972, 2874, 1728, 1284, 1151 cm^{-1};

HRMS (ESI/TOF) m/z: $[M + H]^+$ Calcd for $C_{15}H_{29}O_3$ 257.2111; Found 257.2124.

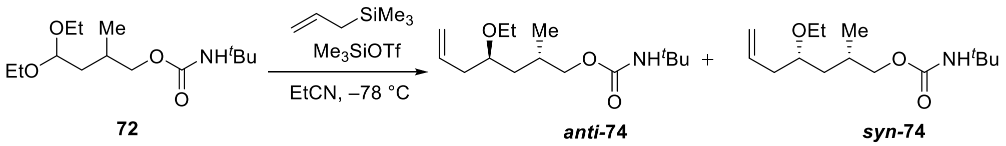

(2*R,4*S**)-4-Ethoxy-2-methylhept-6-en-1-yl *tert*-butylcarbamate (*anti*-74) and (2*R**,4*R**)-4-ethoxy-2-methylhept-6-en-1-yl *tert*-butylcarbamate (*syn*-74).** Following the general procedure for acetal substitution, acetal **72** (0.061 g, 0.22 mmol) in EtCN (2.2 mL) was mixed with allyltrimethylsilane (0.14 mL, 8.8 mmol) and trimethylsilyl trifluoromethanesulfonate (0.080 mL, 0.44 mmol) to provide a 45:55 mixture of diastereomers of ester **74**. Ester **74** was isolated as a 45:55 mixture of ***anti*-74**:***syn*-74** as a colorless oil (0.045 g, 75%):

^{1}H NMR (400 MHz, $CDCl_3$) δ 5.84–5.73 (m, 2H), 5.10–4.97 (m, 4H), 4.61–4.59 (m, 2H), 3.95–3.76 (m, 4H), 3.64–3.51 (m, 2H), 3.46–3.31 (m, 4H), 2.35–2.16 (m, 4H), 2.01–1.85 (m, 2H), 1.57 (ddd, *J* = 13.9, 9.3, 4.5 Hz, 1H), 1.47–1.42 (m, 1H), 1.40–1.32 (m, 1H), 1.31–1.27 (m, 18H), 1.23–1.18 (m, 1H), 1.18–1.13 (m, 6H), 0.94 (d, *J* = 6.8 Hz, 3H), 0.91 (d, *J* = 6.8 Hz, 3H);

^{13}C NMR (100 MHz, $CDCl_3$) δ 155.3 (C), 155.2 (C), 135.0 (CH), 134.9 (CH), 117.1 (CH_2), 117.0 (CH_2), 76.9 (CH), 76.4 (CH), 69.4 (CH_2), 68.6 (CH_2), 64.38 (CH_2), 64.36 (CH_2), 50.30 (C), 50.27 (C), 39.0 (CH_2), 38.6 (CH_2), 38.3 (CH_2), 38.1 (CH_2), 29.9 (CH), 29.6 (CH), 29.1 (two CH_3

overlapping, as determined by HSQC), 18.0 (CH_3), 16.8 (CH_3), 15.7 (two CH_3 overlapping, as determined by HSQC);

IR (ATR) 3347 (br), 2970, 1708, 1265, 1082 cm^{-1};

HRMS (ESI/TOF) m/z: $[M + K]^+$ Calcd for $C_{15}H_{29}KNO_3$ 310.1779; Found 310.1785.

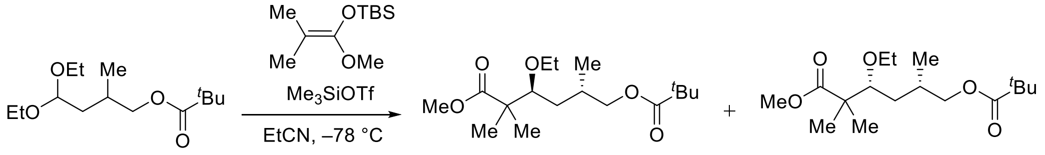

72 *anti*-75 *syn*-75

Methyl (3*R,5*R**)-3-ethoxy-2,2,5-trimethyl-6-(pivaloyloxy)hexanoate (*anti*-75) and methyl (3*R**,5*S**)-3-ethoxy-2,2,5-trimethyl-6-(pivaloyloxy)hexanoate (*syn*-75).** Following the general procedure for acetal substitution, acetal **72** (0.055 g, 0.21 mmol) in EtCN (2.1 mL) was mixed with silyl ketene acetal *tert*-butyl((1-methoxy-2-methylprop-1-en-1-yl)oxy)dimethylsilane (0.20 mL, 0.084 mmol) and trimethylsilyl trifluoromethanesulfonate (0.076 mL, 0.42 mmol) to provide a 50:50 mixture of diastereomers of ester **75**. Ester **75** was isolated as a 50:50 mixture of ***anti*-75**:***syn*-75** as a colorless oil (0.040 g, 60%):

^{1}H NMR (400 MHz,$CDCl_3$) δ 4.05 (dd, *J* = 10.9, 4.8 Hz, 1H), 3.90–3.83 (m, 3H), 3.66 (s, 3H), 3.65 (s, 3H), 3.62–3.49 (m, 6H), 2.00–1.94 (m, 2H), 1.54 (ddd, *J* = 13.7, 10.1, 3.3 Hz, 1H), 1.37–1.33 (m, 2H), 1.20 (s, 9H), 1.19 (s, 9H), 1.18 (s, 3H), 1.18 (s, 3H), 1.13 (t, *J* = 7.0 Hz, 6H), 1.13 (t, *J* = 7.0 Hz, 6H), 1.08 (s, 6H), 1.02–0.95 (m, 7H);

^{13}C NMR (100 MHz, $CDCl_3$) δ 178.7 (C), 178.6 (C), 177.7 (C), 177.6 (C), 82.5 (CH), 82.1 (CH), 69.8 (CH_2), 68.7 (CH_2), 68.44 (CH_2), 68.39 (CH_2), 51.80 (CH_3), 51.78 (CH_3), 48.0 (C), 47.9 (C), 39.02 (C), 38.99 (C), 35.8 (CH_2), 35.6 (CH_2), 30.1 (CH), 30.0 (CH), 27.4 (CH_3), 27.4 (CH_3), 21.5 (CH_3), 21.2 (CH_3), 20.8 (CH_3), 20.6 (CH_3), 18.7 (CH_3), 16.4 (CH_3), 16.03 (CH_3), 15.97 (CH_3);

IR (ATR) 2973, 1726, 1689, 1147, 1088 cm^{-1};

HRMS (ESI/TOF) m/z: $[M + H]^+$ Calcd for $C_{17}H_{33}O_5$ 317.2323; Found 317.2329.

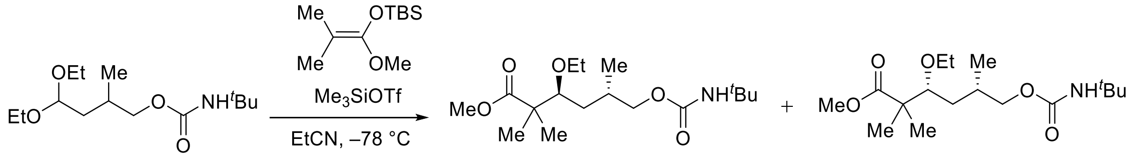

72 *anti*-76 *syn*-76

Methyl (3*R,5*R**)-6-((*tert*-butylcarbamoyl)oxy)-3-ethoxy-2,2,5-trimethylhexanoate (*anti*-76) and methyl (3*R**,5*S**)-6-((*tert*-butylcarbamoyl)oxy)-3-ethoxy-2,2,5-trimethylhexanoate (*syn*-76).** Following the general procedure for acetal substitution, acetal **72** (0.066 g, 0.24 mmol) in EtCN (2.4 mL) was mixed with silyl ketene acetal *tert*-butyl((1-methoxy-2-methylprop-1-en-1-yl)oxy)dimethylsilane (0.23 mL, 0.96 mmol) and trimethylsilyl trifluoromethanesulfonate (0.087 mL, 0.48 mmol) to provide a mixture of unreacted **72** and ester **76** (52:48 mixture of diastereomers).

Ester **76** was isolated as 1:4 mixture of acetal **72** (0.008 g, 12%) and ester **76** (63:37 mixture of diastereomers, 0.037 g, 47%) and a 1:6 mixture of acetal **72** (0.003 g, 4%) and ester **76** (27:73 mixture of diastereomers, 0.020 g, 25%) as a colorless oil. Characterization was done on the 1:4 mixture of acetal **72** and ester **76**:

^{1}H NMR (400 MHz, $CDCl_3$) δ 4.63 (s, 1.6H), 3.97–3.94 (m, 1H), 3.87–3.78 (m, 1.6H, overlapping with acetal **72**), 3.65 (s, 1.8H), 3.64 (s, 3H), 3.62–3.43 (m, 5.4H overlapping with acetal **72**), 1.95–1.86 (m, 1.6H, overlapping with acetal **72**), 1.58 (ddd, *J* = 13.6, 10.2, 3.0 Hz, 0.6H), 1.35–1.22 (m, 16.4H, overlapping with acetal **72**), 1.20–1.15 (m, 4.8H, overlapping with acetal **72**), 1.12 (t, *J* = 7.0 Hz, 4.8H), 1.08–1.06 (m, 4.8H), 1.00–0.92 (m, 5.4H, overlapping with acetal **72**);

Peaks attributed to major diastereomer (***anti*-76**): ^{13}C NMR (100 MHz, $CDCl_3$) δ 177.6 (C), 155.2 (C), 82.6 (CH), 68.5 (CH_2), 68.3 (CH_2), 51.75 (CH_3), 50.34 (C), 47.9 (C), 36.0 (CH_2), 30.3 (CH), 29.1 (CH_3), 21.4 (CH_3), 18.9 (CH_3), 16.3 (CH_3), 15.96 (CH_3);

Peaks attributed to minor diastereomer (***syn*-76**): ^{13}C NMR (100 MHz, $CDCl_3$, characteristic peaks) δ 177.7 (C), 82.1 (CH), 69.6 (CH_2), 68.8 (CH_2), 51.77 (CH_3), 50.31 (C), 35.8 (CH_2), 30.1 (CH), 20.7 (CH_3) 20.6 (CH_3), 16.01 (CH_3);

IR (ATR) 3360 (br), 2970, 1720, 1264, 1083 cm^{-1};

HRMS (ESI/TOF) m/z: $[M + Na]^+$ Calcd for $C_{17}H_{33}NNaO_5$ 354.2251; Found 354.2243.

Scheme 7: Synthesis of ε-acyloxy acetal bearing a β-methyl substituent

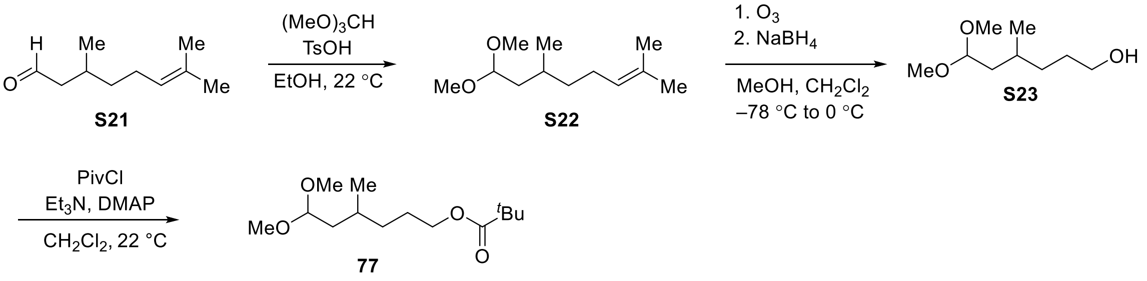

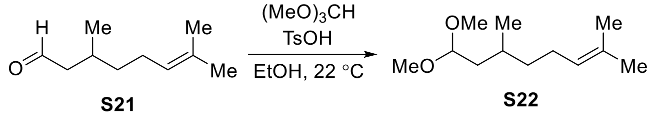

8,8-Dimethoxy-2,6-dimethyloct-2-ene (S22). Following a literature procedure,[10] citronellal **S21** (18 mL, 100 mmol) in trimethyl orthoformate (55 mL, 500 mol) was mixed with methanol (10 mL) and *p*-toluenesulfonic acid (1.9 g, 100 mol) to provide acetal **S22** as a yellow oil (20 g, quantitative). The spectroscopic data of acetal **S22** are consistent with literature data:[10]

^{1}H NMR (400 MHz, $CDCl_3$) δ 5.09 (t, *J* = 7.4 Hz, 1H), 4.46 (dd, *J* = 6.4, 5.1 Hz, 1H), 3.32 (s, 3H), 3.30 (s, 3H), 2.06–1.90 (m, 2H), 1.68 (d, *J* = 1.2 Hz, 3H), 1.67–1.61 (m, 1H), 1.62 (s, 3H), 1.59–1.52 (m, 1H), 1.40–1.30 (m, 2H), 1.22–1.14 (m, 1H), 0.92 (d, *J* = 6.6 Hz, 3H).

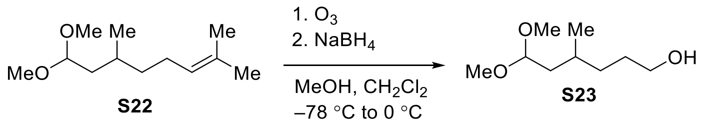

6,6-Dimethoxy-4-methylhexan-1-ol (S23). To a cooled (–78 °C) solution of alkene **S22** (20 g, 100 mmol) in a 1:1 mixture of MeOH:CH_2Cl_2 (500 mL) was applied a steady stream of ozone until saturation was reached and a purple color appeared (30 min). The reaction mixture was warmed to 0 ºC over 1 h and purged with nitrogen. Sodium borohydride (3.7 g, 98 mmol) was then added portion-wise over 30 min. The mixture was allowed to warm to 22 ºC over 1 h and stirred for an additional 3 h. A 5% HCl aqueous solution (100 mL) was added, and the aqueous layer was extracted with CH_2Cl_2 (100 mL). The combined organic layers were washed with brine (100 mL) and dried over anhydrous Na_2SO_4. The resulting mixture was filtered, concentrated *in vacuo*, and purified by flash column chromatography (40:60 EtOAc:hexanes) to provide hydroxy acetal **S23** as a colorless oil (8.0 g, 46%). The spectroscopic data of hydroxy acetal **S23** are consistent with literature data:[11]

^{1}H NMR (400 MHz, $CDCl_3$) δ 4.47 (dd, J = 6.4, 5.1 Hz, 1H), 3.64 (dt, J = 11.4, 5.5 Hz, 2H), 3.31 (s, 6H), 1.70–1.47 (m, 4H, overlapped with H_2O), 1.46–1.33 (m, 2H), 1.30 (s, 1H), 1.29–1.14 (m, 1H), 0.93 (d, J = 6.5 Hz, 3H);

^{13}C NMR (100 MHz, $CDCl_3$) δ 103.2, 63.4, 52.7, 52.5, 39.6, 33.3, 30.2, 29.0, 19.9;

IR (ATR) 3409, 2934, 1378, 1124, 1051 cm^{-1};

HRMS (ESI/TOF) m/z: [M + Na]$^+$ Calcd for $C_9H_{20}NaO_3$ 199.1310; Found 199.1311.

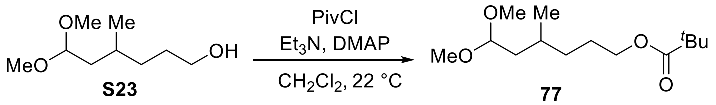

6,6-Dimethoxy-4-methylhexyl pivalate (77). Following the general procedure for esterification of alcohols, hydroxy acetal **S23** (4.8 g, 28 mmol) in CH_2Cl_2 (130 mL) was mixed with triethylamine (7.7 mL, 5.5 mmol), 4-dimethylaminopyridine (0.34 g, 2.8 mmol), and pivaloyl chloride (6.7 mL, 55 mmol) to provide ester **77** as a colorless oil (6.87 g, 96%):

^{1}H NMR (400 MHz, $CDCl_3$) δ 4.42 (dd, J = 6.4, 5.0 Hz, 1H), 4.01 (t, J = 6.6 Hz, 2H), 3.28 (s, 3H), 3.26 (s, 3H), 1.70–1.50 (m, 4H), 1.42–1.29 (m, 2H), 1.22–1.16 (m, 1H), 1.16 (s, 9H), 0.90 (d, J = 6.5 Hz, 3H);

^{13}C NMR (100 MHz, $CDCl_3$) δ 178.7, 103.1, 64.6, 52.7, 52.4, 39.5, 38.8, 33.4, 28.8, 27.3, 26.1, 19.8;

IR (ATR) 2958, 1727, 1481, 1283, 1151 cm-1;

HRMS (ESI/TOF) m/z: [M + Na]$^+$ Calcd for $C_{14}H_{28}NaO_4$ 283.1885; Found 283.1892.

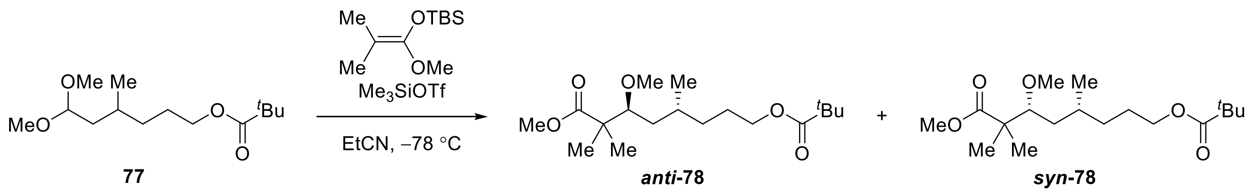

Methyl (3*R,5*S**)-3-methoxy-2,2,5-trimethyl-8-(pivaloyloxy)octanoate (*anti*-78) and methyl (3*R**,5*R**)-3-methoxy-2,2,5-trimethyl-8-(pivaloyloxy)octanoate (*syn*-78)**. Following the general procedure for acetal substitution, acetal **77** (0.026 g, 0.10 mmol) in EtCN (1.0 mL) was mixed with silyl ketene acetal *tert*-butyl((1-methoxy-2-methylprop-1-en-1-yl)oxy)dimethylsilane (0.036 mL, 0.20 mmol) and trimethylsilyl trifluoromethanesulfonate (0.10 mL, 0.40 mmol) to provide a 50:50 mixture of diastereomers of ester **78** and some stannane impurities as a colorless oil (0.021 g, 67%):

^{1}H NMR (400 MHz, $CDCl_3$) δ 4.11–4.00 (m, 4H), 3.67 (s, 3H), 3.66 (s, 3H), 3.49 (dd, *J* = 10.1, 2.0 Hz, 1H), 3.46 (dd, *J* = 9.5, 2.7 Hz, 1H), 3.42 (s, 3H), 3.40 (s, 3H), 1.75–1.51 (m, 8H), 1.47 (ddd, *J* = 13.4, 10.0, 3.0 Hz, 1H), 1.38–1.29 (m, 2H), 1.20 (s, 9H), 1.18 (s, 9H), 1.18 (s, 6H), 1.08 (s, 6H), 1.01–0.95 (m, 1H), 0.94 (s, 3H), 0.92 (s, 3H);

^{13}C NMR (100 MHz, $CDCl_3$) δ 178.8 (C), 178.7 (C), 177.64 (C), 177.57 (C), 84.3 (CH), 84.2 (CH), 64.8 (CH_2), 64.7 (CH_2), 61.2 (CH_3), 60.8 (CH_3), 51.82 (CH_3), 51.79 (CH_3), 47.92 (C), 47.90 (C), 39.5 (CH_2), 39.3 (CH_2), 38.87 (C), 38.86 (C), 34.6 (CH_2), 31.9 (CH_2), 29.8 (CH), 29.7 (CH), 27.3 (two CH_3 overlapping, as determined by HSQC), 26.4 (CH_2), 26.0 (CH_2), 21.34 (CH_3), 21.28 (CH_3), 20.9 (CH_3), 20.8 (CH_3), 20.7 (CH_3), 19.2 (CH_3);

IR (ATR) 2954, 1726, 1283, 1151, 1097 cm^{-1};

HRMS (ESI/TOF) m/z: $[M + Na]^+$ Calcd for $C_{18}H_{34}NaO_5$ 353.2298; Found 353.2284.

Scheme 8: Synthesis of ε-acyloxy acetal bearing a γ-methyl substituent

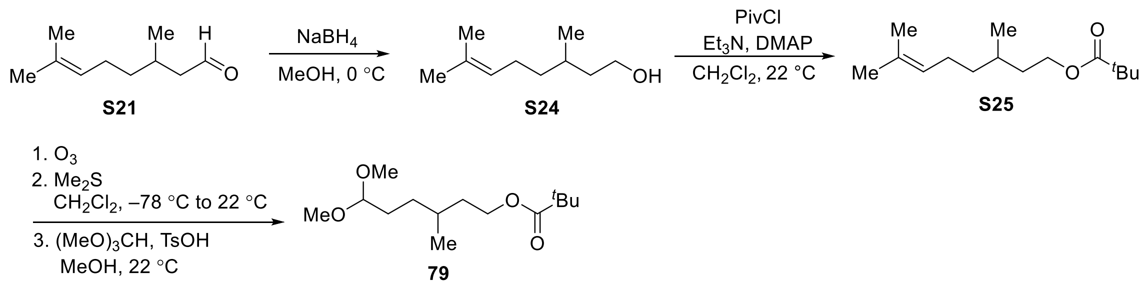

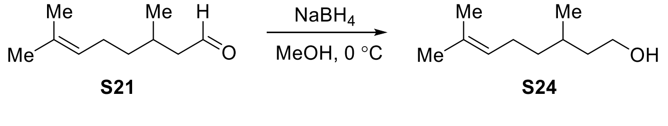

3,7-Dimethyloct-6-en-1-ol (S24). Following a literature procedure,[12] citronellal **S21** (18 mL, 100 mmol) in methanol (100 mL) was mixed with sodium borohydride (5.7 g, 150 mol) to provide citronellol **S24**, as a colorless oil (16 g, quantitative). The spectroscopic data of citronellol **S24** are consistent with literature data:[12]

^{1}H NMR (400 MHz, $CDCl_3$) δ 5.10 (t, J = 7.2 Hz, 1H), 3.68 (t, J = 6.9 Hz, 2H), 2.07–1.89 (m, 2H), 1.69–1.63 (m, 4H), 1.60 (s, 3H), 1.45–1.28 (m, 2H), 1.25–1.11 (m, 2H), 0.92–0.90 (m, 4H).

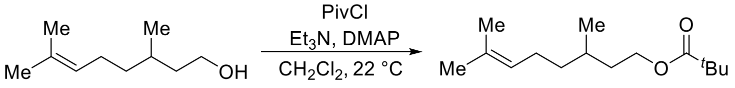

S24 **S25**

3,7-Dimethyloct-6-en-1-yl pivalate (S25). Following the general procedure for esterification of alcohols, citronellol **S24** (16 g, 100 mmol) in CH_2Cl_2 (200 mL) was mixed with triethylamine (28 mL, 200 mmol), *N*,*N*-dimethylaminopyridine (1.2 g, 10 mmol), and pivaloyl chloride (25 mL, 200 mol) to provide ester **S25** as a colorless oil (23 g, 95%) with traces of a pivaloyl byproduct. This mixture was used directly in the next step without further purification. The spectral data for pivalate **S25** obtained are consistent with literature data:[13]

^{1}H NMR (400 MHz, $CDCl_3$) δ 5.08 (t, J = 6.8 Hz, 1H), 4.16–3.92 (m, 2H), 2.04–1.88 (m, 2H), 1.67–1.61 (m, 2H), 1.68 (s, 3H), 1.60 (s, 3H), 1.55 (dddd, J = 13.1, 6.6, 3.8, 1.3 Hz, 1H), 1.49–1.28 (m, 2H), 1.19 (s, 9H), 0.91 (d, J = 6.6 Hz, 3H).

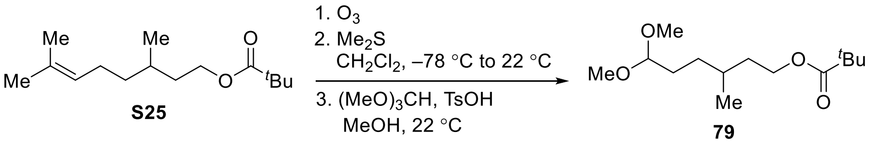

S25 **79**

6,6-Dimethoxy-3-methylhexyl pivalate (79). Following the general procedure for ozonolysis, ester **S25** (23 g, 100 mmol) in CH_2Cl_2 (100 mL) was treated with O_3 and Me_2S (62 mL, 1.0 mol) to provide the corresponding aldehyde (23 g, quantitative), which was used directly in the next step without further purification.

Following the general procedure for diethyl acetal formation, the crude aldehyde (23 g, 100 mmol) was mixed with trimethyl orthoformate (55 mL, 500 mol), methanol (10 mL), and *p*-toluenesulfonic acid (1.9 g, 0.010 mol) to provide acetal **79** as a colorless oil (15 g, 58%):

^{1}H NMR (400 MHz, $CDCl_3$) δ 4.33 (t, J = 5.7 Hz, 1H), 4.13–4.04 (m, 2H), 3.31 (s, 6H), 1.85–1.30 (m, 7H), 1.19 (s, 9H), 0.92 (d, J = 6.6 Hz, 3H);

^{13}C NMR (100 MHz, $CDCl_3$) δ 178.8 (C), 104.9 (CH), 62.9 (CH_2), 52.9 (CH_3), 52.8 (CH_3), 38.9 (C), 35.6 (CH_2), 31.7 (CH_2), 30.2 (CH_2), 30.0 (CH), 27.3 (CH_3), 19.6 (CH_3);

IR (ATR) 2955, 2872, 1725, 1283, 1087 cm^{-1};

HRMS (ESI/TOF) m/z: $[M + Na]^+$ Calcd for $C_{14}H_{28}NaO_4$ 283.1885; Found 283.1886.

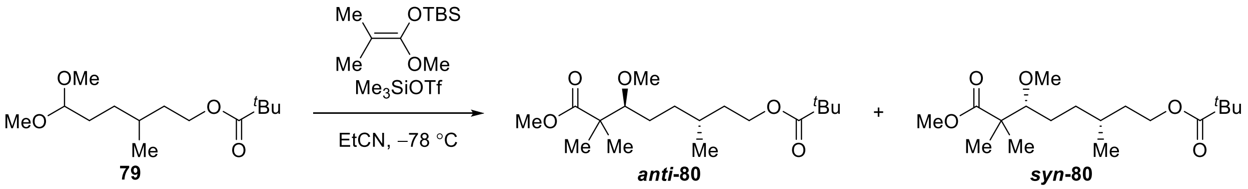

79 ***anti*-80** ***syn*-80**

Methyl (3*R,6*S**)-3-methoxy-2,2,6-trimethyl-8-(pivaloyloxy)octanoate (*anti*-80) and methyl (3*R**,6*R**)-3-methoxy-2,2,6-trimethyl-8-(pivaloyloxy)octanoate (*syn*-80).** Following the general procedure for acetal substitution, acetal **79** (0.13 g, 0.50 mmol) in EtCN (5.0 mL) was mixed with silyl ketene acetal *tert*-butyl((1-methoxy-2-methylprop-1-en-1-yl)oxy)dimethylsilane (0.50 mL, 2.0 mmol) and trimethylsilyl trifluoromethanesulfonate (0.18 mL, 1.0 mmol) to provide a 50:50 mixture of diastereomers of esters **80**. Ester **80** was isolated as a 49:51 mixture of diastereomers as a colorless oil (0.17 g, quantitative):

^{1}H NMR (400 MHz, $CDCl_3$) δ 4.17–4.02 (m, 4H), 3.68 (s, 6H), 3.42 (d, *J* = 1.1 Hz, 6H), 3.37–3.28 (m, 2H), 1.71–1.60 (m, 2H), 1.59–1.30 (m, 12H), 1.19 (s, 24H), 1.10 (s, 6H), 0.96–0.88 (m, 6H);

^{13}C NMR (100 MHz, $CDCl_3$) δ 178.8 (two C overlapping), 177.7 (two C overlapping), 87.1 (CH), 86.8 (CH), 62.9 (CH_2), 61.2 (CH_2), 61.2 (two CH_3 overlapping, as determined by HSQC), 51.9 (two CH_3 overlapping, as determined by HSQC), 47.9 (two CH_2 overlapping, as determined by HSQC), 38.9 (two C overlapping), 35.9 (CH), 35.5 (CH), 34.7 (CH_2), 34.4 (CH_2), 30.4 (CH_2), 30.2 (CH_2), 29.2 (two CH_2 overlapping, as determined by HSQC), 27.4 (two CH_3 overlapping, as determined by HSQC), 25.8 (CH_3), 21.4 (CH_3), 20.91 (CH_3), 20.88 (CH_3), 19.7 (CH_3), 19.5 (CH_3);

IR (ATR) 2955, 2931, 1726, 1282, 1106 cm^{-1};

HRMS (ESI/TOF) m/z: [M + Na]$^{+}$ Calcd for $C_{18}H_{34}NaO_5$ 353.2304; Found 353.2308.

Derivatization and stereochemical correlation

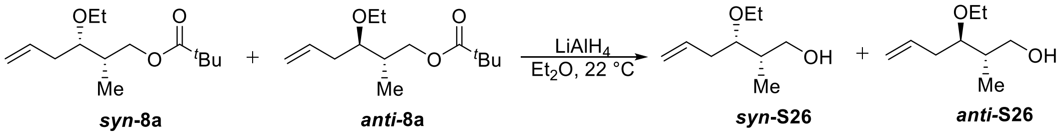

(2*R,3*R**)-3-Ethoxy-2-methylhex-5-en-1-ol (*syn*-S26) and (2*R**,3*S**)-3-ethoxy-2-methylhex-5-en-1-ol (*anti*-S26)**. Following the general procedure for ester reduction, a 74:26 mixture of ***syn*-8a**:***anti*-8a** (1.1 g, 4.5 mmol) in Et_2O (45 mL) was mixed with lithium aluminum hydride (0.25 g, 6.8 mmol) to provide a 72:28 mixture of alcohol **S26** (0.75 g, quantitative):

^{1}H NMR (400 MHz, $CDCl_3$) δ 5.90–5.75 (m, 1.4H), 5.15–5.01 (m, 2.8H), 3.73–3.64 (m, 1.8H), 3.64–3.49 (m, 3.4H), 3.49–3.39 (m, 1.4H), 3.30 (dt, *J* = 7.2, 5.0 Hz, 0.4H), 3.13 (dd, *J* = 7.1, 4.2 Hz, 0.4H), 2.70–2.68 (m, 1H), 2.48–2.33 (m, 1.4H), 2.33–2.17 (m, 1.4H), 2.02–1.93 (m, 1H), 1.84 (sxtd, *J* = 7.0, 3.5 Hz, 0.4H), 1.20 (t, *J* = 7.0 Hz, 1.2H), 1.18 (t, *J* = 7.0 Hz, 3H), 0.91 (d, *J* = 7.1 Hz, 3H), 0.90 (t, *J* = 7.2 Hz, 1.2H);

Peaks attributed to major diastereomer (***syn*-S26**): ^{13}C NMR (100 MHz, $CDCl_3$) δ 135.5 (CH), 116.9 (CH_2), 82.5 (CH), 66.6 (CH_2), 65.5 (CH_2), 37.2 (CH), 35.2 (CH_2), 15.7 (CH_3), 11.5 (CH_3);

Peaks attributed to minor diastereomer (***anti*-S26**): ^{13}C NMR (100 MHz, $CDCl_3$) δ 134.3 (CH), 117.4 (CH_2), 84.7 (CH), 67.1 (CH_2), 65.4 (CH_2), 37.9 (CH), 35.8 (CH_2), 15.6 (CH_3), 14.2 (CH_3);

IR (ATR) 3364 (br), 2958, 1459, 1077, 1018 cm^{-1};

HRMS (ESI/TOF) m/z: [2M + H]$^{+}$ Calcd for $C_{18}H_{37}O_4$ 317.2686; Found 317.2682.

Substitution products **29–34** were also reduced using the general procedure for ester reduction to provide alcohol ***syn*-S26** as the major product.

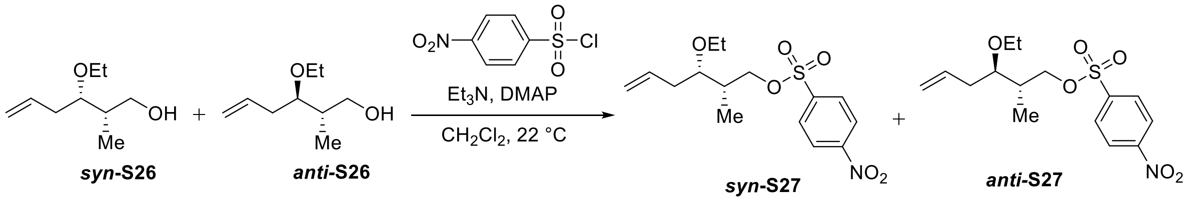

syn-**S26** *anti*-**S26** *syn*-**S27** *anti*-**S27**

(2*R,3*R**)-3-Ethoxy-2-methylhex-5-en-1-yl 4-nitrobenzenesulfonate (*syn*-S27) and (2*R**,3*S**)-3-ethoxy-2-methylhex-5-en-1-yl 4-nitrobenzenesulfonate (*anti*-S27)**. To a cooled (0 °C) solution of a 70:30 mixture of alcohols ***syn*-S26**:***anti*-S26** (0.080 g, 0.50 mmol) in CH_2Cl_2 (5.0 mL) were added triethylamine (0.18 mL, 1.3 mmol) and 4-dimethylaminopyridine (0.006 g, 0.05 mmol). After 30 min, 4-nitrobenzenesulfonyl chloride (0.28 g, 1.3 mmol) was added and the mixture was warmed to 22 °C. After 4 h, the mixture was diluted with CH_2Cl_2 (10 mL). The resulting mixture was washed with H_2O (5 mL) and brine (5 mL) and was dried over anhydrous Na_2SO_4. The mixture was filtered, concentrated in *vacuo*, and purified by flash column chromatography (25:75 Et_2O:hexanes) to provide a 57:43 mixture of sulfonates ***syn*-S27**:***anti*-S27** (0.11 g, 64%) as a colorless oil and sulfonate ***syn*-S27** as a white solid (0.050 g, 29%). Sulfonate ***syn*-S27** was incorporated into a guanidinium sulfonate framework host for crystal structure determination. Characterization was done on the pure sample of ***syn*-S27**:

mp = 62–64 °C;

^{1}H NMR (400 MHz, $CDCl_3$) δ 8.41 (d, *J* = 8.7 Hz, 2H), 8.11 (d, *J* = 8.7 Hz, 2H), 5.71 (ddt, *J* = 17.2, 10.2, 7.0 Hz, 1H), 5.13–4.98 (m, 2H), 4.16 (dd, *J* = 9.3, 7.3 Hz, 1H), 3.98 (dd, *J* = 9.3, 6.3 Hz, 1H), 3.56 (dq, *J* = 9.3, 7.0 Hz, 1H), 3.36–3.25 (m, 2H), 2.37–2.27 (m, 1H), 2.19–2.11 (m, 1H), 2.02 (sxtd, *J* = 7.0, 3.4 Hz, 1H), 1.08 (t, *J* = 7.0 Hz, 3H), 0.88 (d, *J* = 7.0 Hz, 3H);

^{13}C NMR (100 MHz, $CDCl_3$) δ 150.9 (C), 142.1 (C), 134.6 (CH), 129.4 (CH), 124.6 (CH), 117.5 (CH_2), 77.9 (CH_2), 74.0 (CH_2), 65.6 (CH_2), 36.1 (CH), 35.7 (CH_2), 15.6 (CH_3), 10.5 (CH_3);

IR (ATR) 2977, 2872, 1527, 1350, 1186 cm^{-1};

HRMS (ESI/TOF) m/z: [M + Na]$^+$ Calcd for $C_{15}H_{21}NNaO_6S$ 366.0982; Found 366.0967.

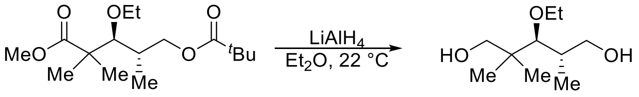

anti-**10a** *anti*-**S28**

(3*R,4*R**)-3-Ethoxy-2,2,4-trimethylpentane-1,5-diol (*anti*-S28)**. Following the general procedure for ester reduction, ester ***anti*-10a** (0.050 g, 0.17 mmol) in Et_2O (1.7 mL) was mixed with lithium aluminum hydride (0.025 g, 0.64 mmol) to provide alcohol ***anti*-S28** (0.030 g, quantitative). The spectroscopic data of ester ***anti*-S28** are consistent with literature data:[4]

^1H NMR (400 MHz, $CDCl_3$) δ 3.68–3.47 (m, 5H), 3.41 (d, J = 11.1 Hz, 1H), 3.32 (d, J = 1.8 Hz, 1H), 2.67 (s, 2H), 2.03–1.93 (m, 1H), 1.18 (t, J = 7.0 Hz, 3H), 0.96 (d, J = 6.6 Hz, 3H), 0.95 (s, 3H), 0.91 (s, 3H);

^{13}C NMR (100 MHz, $CDCl_3$) δ 86.1, 71.6, 69.1, 68.6, 40.1, 36.7, 22.9, 21.6, 15.8, 11.9.

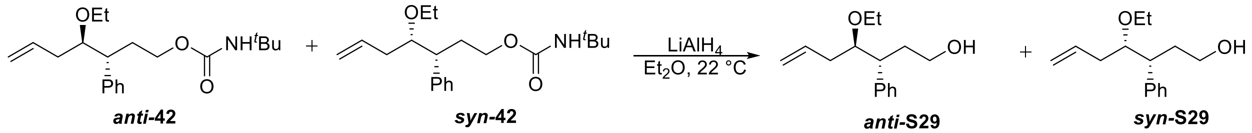

(3*R,4*R**)-4-Ethoxy-3-phenylhept-6-en-1-ol (*anti*-S29) and (3*R**,4*S**)-4-ethoxy-3-phenylhept-6-en-1-ol (*syn*-S29)**. Following the general procedure for ester reduction, an 80:20 mixture of ***anti*-42**:***syn*-42** (0.015 g, 0.050 mmol) in Et_2O (0.50 mL) was mixed with lithium aluminum hydride (0.0040 g, 0.10 mmol) to provide a 77:23 mixture of alcohol **S29** (0.0090 g, 77%):

^1H NMR (400 MHz, $CDCl_3$) δ 7.32–7.20 (m, 3H), 7.17–7.07 (m, 3.5H), 5.77–5.67 (m, 1.3H), 5.07–4.79 (m, 2.6H), 3.82–3.71 (m, 0.6H), 3.67–3.54 (m, 1.6H), 3.54–3.33 (m, 5.6H), 2.94–2.87 (m, 0.3H), 2.75 (td, J = 9.0, 4.4 Hz, 1H), 2.24–2.12 (m, 2H), 2.06–1.75 (m, 3.2H), 1.15 (t, J = 7.0 Hz, 3H), 1.08 (t, J = 7.0 Hz, 0.9H);

Peaks attributed to major diastereomer (***anti*-S29**): ^{13}C NMR (100 MHz, $CDCl_3$) δ 142.9 (C), 134.6 (CH), 128.7 (CH), 128.5 (CH), 126.7 (CH), 117.3 (CH_2), 83.0 (CH), 65.7 (CH_2), 61.69 (CH_2), 47.2 (CH), 36.2 (CH_2), 35.7 (CH_2), 15.56 (CH_3);

Peaks attributed to minor diastereomer (***syn*-S29**): ^{13}C NMR (100 MHz, $CDCl_3$) δ 141.7 (C), 135.7 (CH), 129.3 (CH), 128.3 (CH), 126.6 (CH), 116.9 (CH_2), 83.5 (CH), 66.2 (CH_2), 61.65 (CH_2), 46.4 (CH), 36.1 (CH_2), 34.5 (CH_2), 15.62 (CH_3);

IR (ATR) 3351 (br), 2925, 1073, 911, 700 cm^{-1};

HRMS (ESI/TOF) m/z: $[M + H]^+$ Calcd for $C_{15}H_{23}O_2$ 235.1693; Found 235.1684.

Substitution products **40** was also reduced using the general procedure for ester reduction while substitution products **45** was debenzylated according to literature procedure[14] to provide alcohol ***anti*-S29** as the major product.

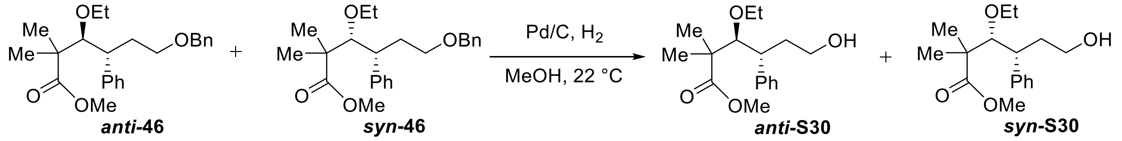

Methyl (3*R,4*S**)-3-ethoxy-6-hydroxy-2,2-dimethyl-4-phenylhexanoate (*anti*-S30) and methyl (3*R**,4*R**)-3-ethoxy-6-hydroxy-2,2-dimethyl-4-phenylhexanoate (*syn*-S30)**. To a solution of a 78:22 mixture of diastereomers of ester **46** (0.095 mg, 0.25 mmol) in methanol (2.0 mL) was added Pd/C (10% wt, 0.079 mg, 0.07 mmol). The mixture was stirred for 48 h at 22 °C under H_2 atmosphere (1.0 atm). The mixture was filtered through a plug of diatomaceous earth and washed with methanol (10 mL). The resulting mixture was concentrated *in vacuo* to afford a

78:22 mixture of the corresponding hydroxy ester **S30** as a colorless oil (0.063 mg, 86%), which was used directly in the next step without further purification:

^{1}H NMR (400 MHz, $CDCl_3$) δ 7.32–7.17 (m, 6.5H), 3.82–3.77 (m, 2.6H), 3.70 (s, 0.9H), 3.67–3.61 (m, 1.3H), 3.45–3.40 (m, 1.3H), 3.35–3.31 (m, 1.3H), 3.25 (s, 3H), 2.95–2.87 (m, 1.3H), 2.36–2.24 (m, 1H), 1.91–1.75 (m, 1.6H), 1.64 (s, 1.3H), 1.24 (s, 6H), 1.10–1.07 (m, 4.8H), 0.99 (t, *J* = 7.0 Hz, 0.9H);

Peaks attributed to major diastereomer (***anti*-S30**): ^{13}C NMR (100 MHz, $CDCl_3$) δ 177.0 (C), 142.7 (C), 129.0 (CH), 128.5 (CH), 126.7 (CH), 87.5 (CH), 69.9 (CH_2), 61.3 (CH_2), 51.4 (CH_3), 48.3 (C), 45.9 (CH), 36.1 (CH_2), 23.6 (CH_3), 19.9 (CH_3), 15.6 (CH_3);

Peaks attributed to minor diastereomer (***syn*-S30**): ^{13}C NMR (100 MHz, $CDCl_3$) δ 178.2 (C), 142.3 (C), 129.5 (CH), 128.2 (CH), 126.5 (CH), 87.9 (CH), 69.8 (CH_2), 60.9 (CH_2), 51.9 (CH_3), 47.9 (C), 45.7 (CH), 36.5 (CH_2), 24.4 (CH_3), 19.4 (CH_3), 15.7 (CH_3);

IR (ATR) 3436 (br), 2973, 1722, 1116, 701 cm^{-1};

HRMS (ESI/TOF) m/z: [M + Na]$^+$ Calcd for $C_{17}H_{26}NaO_4$ 317.1723; Found 317.1720.

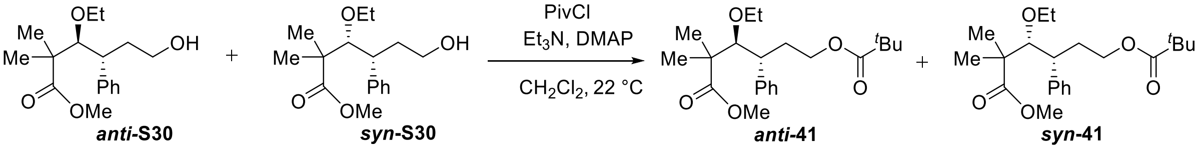

anti-**S30** *syn*-**S30** *anti*-**41** *syn*-**41**

Methyl (3*R,4*S**)-3-(benzyloxy)-2,2-dimethyl-4-phenyl-6-(pivaloyloxy)hexanoate (*anti*-41) and methyl (3*R**,4*R**)-3-(benzyloxy)-2,2-dimethyl-4-phenyl-6-(pivaloyloxy)hexanoate (*syn*-41).** Following the general procedure for esterification of alcohols, a 78:22 mixture of diastereomers of alcohols **S30** (0.060 g, 0.20 mmol) in CH_2Cl_2 (2.0 mL) was mixed with triethylamine (0.043 mL, 0.30 mmol), 4-dimethylaminopyridine (0.0030 g, 0.020 mmol), and pivaloyl chloride (0.037 mL, 0.3 mmol) to provide ester **41** as 75:25 mixtures of diastereomers as a colorless oil (0.090 g, quantitative). The spectroscopic data of ester **41** are consistent with those of nucleophilic substitution products of acetals **36**.

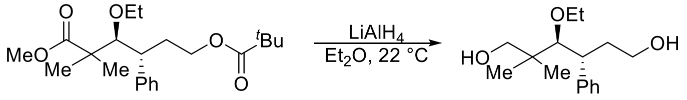

anti-**41** *anti*-**S31**

(3*R,4*S**)-3-Ethoxy-2,2-dimethyl-4-phenylhexane-1,6-diol (*anti*-S31).** Following the general procedure for ester reduction, ester ***anti*-41** (0.23 g, 0.60 mmol) in Et_2O (6.0 mL) was mixed with lithium aluminum hydride (0.034 g, 0.90 mmol) to provide diol ***anti*-S31** as a white solid (0.056 g, 35%):

mp = 82–85 ºC;

^{1}H NMR (400 MHz, $CDCl_3$) δ 7.43–7.34 (m, 2H), 7.30–7.18 (m, 3H), 3.75 (qd, *J* = 7.0, 3.2 Hz, 2H), 3.51 (ddd, *J* = 10.8, 6.1, 1.5 Hz, 1H), 3.41 (d, *J* = 2.5 Hz, 1H), 3.34 (ddd, *J* = 10.6, 8.4, 5.7 Hz,

1H), 3.13–3.01 (m, 2H), 2.95 (d, *J* = 11.0 Hz, 1H), 2.11 (ddd, *J* = 13.4, 10.8, 4.8 Hz, 1H), 2.08–1.86 (m, 1H), 1.28 (t, *J* = 7.0 Hz, 3H), 1.01 (s, 3H), 0.72 (s, 3H);

^{13}C NMR (100 MHz, $CDCl_3$) δ 142.0 (C), 129.7 (CH), 128.4 (CH), 126.8 (CH), 91.9 (CH), 70.9 (CH_2), 70.3 (CH_2), 60.8 (CH_2), 44.0 (CH), 40.6 (C), 39.4 (CH_2), 24.8 (CH_3), 22.3 (CH_3), 16.1 (CH_3);

IR (ATR) 3351 (br), 3026, 2967, 2927, 2874 cm^{-1};

HRMS (ESI/TOF) m/z: [M + K]$^+$ Calcd for $C_{16}H_{26}KO_3$ 305.1519; Found 305.1518.

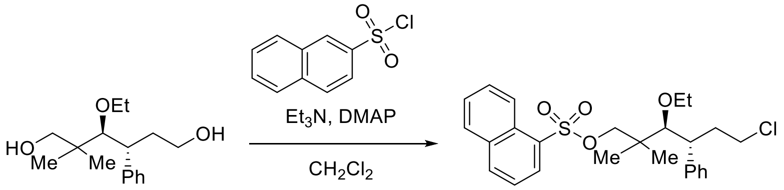

***anti*-S31** ***anti*-S32**

(3*R,4*S**)-6-chloro-3-ethoxy-2,2-dimethyl-4-phenylhexyl naphthalene-1-sulfonate (*anti*-S32).** Following the general procedure for esterification of alcohols, diol ***anti*-S31** (0.028 g, 0.10 mmol) in CH_2Cl_2 (0.50 mL) was mixed with triethylamine (0.056 mL, 0.40 mmol), 4-dimethylaminopyridine (0.010 g, 0.080 mmol), and 2-naphthylsulfonyl chloride (0.10 g, 0.40 mmol) to provide sulfonate ***anti*-S32** as a white solid. Sulfonate ***anti*-S32** was crystalized from Et_2O to give white needles, which were used for X-ray crystallography (0.040 g, 83%):

mp = 83–86 ºC;

^{1}H NMR (400 MHz, $CDCl_3$) δ 8.43 (d, *J* = 1.8 Hz, 1H), 8.02–7.90 (m, 3H), 7.78 (dd, *J* = 8.7, 1.9 Hz, 1H), 7.67 (m, 2H), 7.27–7.05 (m, 5H), 3.92 (d, *J* = 8.9 Hz, 1H), 3.65–3.53 (m, 2H), 3.47–3.32 (m, 3H), 3.16–2.99 (m, 2H), 2.32 (ddd, *J* = 13.7, 10.0, 6.7 Hz, 1H), 2.09–1.91 (m, 1H), 1.05 (t, *J* = 7.0 Hz, 3H), 0.87 (s, 3H), 0.76 (s, 3H);

^{13}C NMR (100 MHz, $CDCl_3$) δ 143.6 (C), 135.4 (C), 132.8 (C), 132.1 (C), 129.9 (CH), 129.7 (CH), 129.5 (CH), 128.9 (two CH overlapping, as determined by HSQC), 128.4 (CH), 128.1 (CH), 127.9 (CH), 126.8 (CH), 122.7 (CH), 86.2 (CH), 76.8 (CH_2, overlapped with $CHCl_3$, as determined by HSQC and DEPT), 69.3 (CH_2), 44.6 (CH), 43.7 (CH_2), 41.1 (C), 35.2 (CH_2), 22.2 (CH_3), 19.9 (CH_3), 15.5 (CH_3);

IR (ATR) 2970, 2877, 1340, 1173, 1102 cm^{-1};

HRMS (ESI/TOF) m/z: [M + NH_4]$^+$ Calcd for $C_{26}H_{35}ClNO_4S$ 492.1977; Found 492.1974.

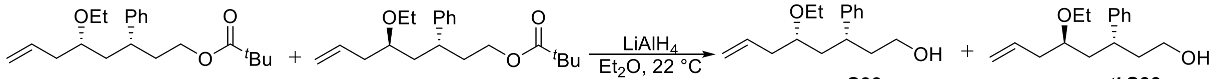

***syn*-66** ***anti*-66** ***syn*-S33** ***anti*-S33**

(3*R,5*S**)-5-Ethoxy-3-phenyloct-7-en-1-ol (*syn*-S33) and (3*R**,5*R**)-5-ethoxy-3-phenyloct-7-en-1-ol (*anti*-S33)**. Following the general procedure for ester reduction, a 70:30 mixture of ***syn*-66**:***anti*-66** (0.14 g, 0.41 mmol) in Et_2O (4.0 mL) was mixed with lithium aluminum hydride (0.031 g, 8.2 mmol) to provide a 70:30 mixture of alcohol **S33** as a colorless oil (0.091 g, 90%). A pure

sample of alcohol ***syn*-S33** was isolated in small fractions by flash column chromatography (40:60 EtOAc:hexanes) and was used for characterization:

^{1}H NMR (400 MHz, $CDCl_3$) δ 7.39–7.32 (m, 2H), 7.28–7.23 (m, 3H), 5.85 (ddt, *J* = 17.3, 10.3, 7.1 Hz, 1H), 5.16–5.07 (m, 2H), 3.63–3.44 (m, 3H), 3.38 (dq, *J* = 9.2, 7.0 Hz, 1H), 3.30–3.22 (m, 1H), 2.90 (dddd, *J* = 9.9, 8.4, 6.3, 5.1 Hz, 1H), 2.39–2.25 (m, 2H), 2.06–1.90 (m, 2H), 1.89–1.78 (m, 2H), 1.44 (s, 1H), 1.16 (t, *J* = 7.0 Hz, 3H);

^{13}C NMR (100 MHz, $CDCl_3$) δ 145.1 (C), 134.9 (CH), 128.7 (CH), 127.7 (CH), 126.4 (CH), 117.1 (CH_2), 76.7 (CH), 64.1 (CH_2), 61.1 (CH_2), 41.0 (CH_2), 39.5 (CH_2), 38.9 (CH), 38.1 (CH_2), 15.6 (CH_3);

IR (ATR) 3382 (br), 2929, 1076, 911, 700 cm^{-1};

HRMS (ESI/TOF) m/z: $[M + Na]^+$ Calcd for $C_{16}H_{24}NaO_2$ 271.1669; Found 271.1658.

Computational analysis

Calculations of oxocarbenium ions were performed to support the formation of cyclic dioxocarbenium ions and explains the high diastereoselectivity of these intermediates in substitution reactions. An initial conformer search using molecular mechanics (MMFF) in Spartan ’20 or Spartan ’24 was used to generate different conformers for the oxocarbenium ion. Conformers whose energy were 20 kcal/mol or higher than the lowest-energy conformer were removed. The remaining conformers were optimized using the Hartree–Fock method and 3-21G basis set. Duplicated conformers with the same energy were removed and the remaining conformers were subjected to single-point energy calculations using density functional theory (DFT) with the ωB97X-D hybrid functional and Pople’s 6-31+G* basis set. High-energy conformers (≥10 kcal/mol higher than the lowest-energy conformer) were removed. Equilibrium geometries on the remaining conformers were calculated using the ωB97X-D hybrid functional and Pople’s 6-31+G*. The ωB97X-D functional was chosen due to its accuracy in determining geometries of small organic molecules,[15] including cyclic oxocarbenium ions,[16] while accounting for significant non-covalent interactions.[17] The cost-effectiveness and accuracy of Pople’s 6-31+G* basis set made the geometries optimized using this basis set a good starting point for subsequent energy calculations using larger basis sets.[18] After removal of high-energy conformers (≥10 kcal/mol higher than the lowest-energy conformer), the remaining conformers were subjected to single-point energy calculations using the ωB97X-V or ωB97X-D functional and 6-311+G(2df,2p)[19] basis set. Vibrational frequency calculations were performed on the lowest energy conformers using the ωB97X-D hybrid functional and Pople’s 6-31+G* basis set. In some cases, tight restrictions on tolerances of geometry and gradient (“GEOMTOL=TIGHT”) was used to remove the imaginary low-frequency vibrations.

^{13}C NMR calculations were performed to support the stereochemistry assignments of the substitution products. The NMR spectrum calculation method in Spartan ’24 was used to calculate the Boltzmann-weighted ^{13}C NMR chemical shifts of each diastereomer. This method followed the same calculation sequence discussed above, which includes a conformer search, equilibrium geometry optimizations, and energy calculations. The final NMR calculations were conducted on

the low-energy conformers using the ωB97X-D hybrid functional and Pople's 6-31+G* basis set, which were previously used for NMR calculations of flexible small molecules.[20] The Boltzmann-weighted ^{13}C NMR chemical shifts and the experimental ^{13}C NMR chemical shifts of both diastereomers were used to calculate the CP3 and $MAE_{\Delta\Delta\delta}$ parameters following Goodman's procedure and Bifulco's procedure, respectively.[21,22] These parameters compare the difference between the experimental chemical shifts of the diastereomers to the difference between the computed chemical shifts of the diastereomers.[21] This method of comparison could reduce systematic errors that arise from calibration and computing procedures. A large and positive CP3 value suggests good agreement between the experimental and computed values and the assignment is likely to be correct, whereas a large negative value indicated the assignment is likely to be incorrect. For the $MAE_{\Delta\Delta\delta}$ parameter, a lower value indicates a better assignment.[15]

Cis-dioxocarbenium ion **16a**

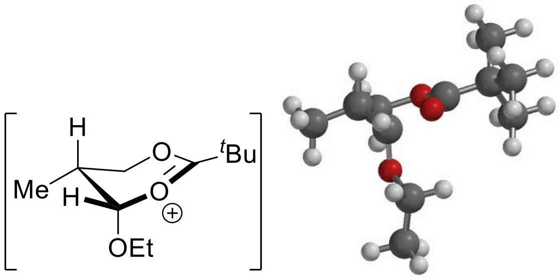

16a

Spartan '20: ωB97X-V/6-311+G(2df,2p)

Number of imaginary vibrations: 0

Number of basis functions: 665

Charge : +1

Number of electrons: 110

Relative energy: 0 kcal/mol

SCF total energy (ωB97X-V/gas): –657.299238 hartrees

Table S1. Cartesian Coordinates (Angstroms) for **16a**

**Atom**	**X**	**Y**	**Z**
C	1.071180	-1.975818	0.071394
C	1.974269	-0.818861	-0.308760
H	2.805298	-1.119007	-0.945017
H	2.344126	-0.280049	0.565409
O	1.247156	0.167465	-1.109852
C	-0.026802	0.300239	-1.043394

O	-0.771393	-0.401534	-0.279399
C	-0.161658	-1.380343	0.724540
H	-0.992351	-2.081574	0.845687
O	0.159882	-0.697814	1.837076
C	-0.933747	-0.319321	2.704001
H	-1.564910	0.404029	2.176492
H	-1.529183	-1.214649	2.919617
C	-0.341798	0.271366	3.962505
H	0.257887	1.156191	3.733042
H	0.288607	-0.459728	4.474890
H	-1.147196	0.568050	4.639714
H	0.748400	-2.496912	-0.837531
C	-0.650373	1.361522	-1.909896
C	-0.142097	1.183583	-3.354393
H	-0.454334	0.221359	-3.772380
H	0.945876	1.261868	-3.415878
H	-0.576581	1.973669	-3.972384
C	-0.167614	2.718062	-1.340506
H	0.919724	2.814967	-1.395646
H	-0.487835	2.856054	-0.302684
H	-0.614473	3.516864	-1.938475
C	-2.179428	1.272093	-1.855047
H	-2.543581	0.309307	-2.224717
H	-2.595454	2.056722	-2.492211
H	-2.561550	1.422502	-0.841835
C	1.786812	-2.957938	0.999360
H	2.660518	-3.385138	0.500146
H	1.121876	-3.783480	1.267675
H	2.110748	-2.463746	1.918458

Trans-dioxocarbenium ion **18a**

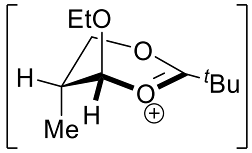
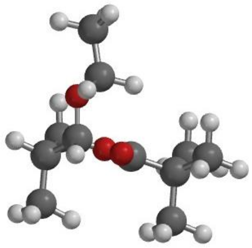

18a

Spartan ’20: ωB97X-V/6-311+G(2df,2p)

Number of imaginary vibrations: 0

Number of basis functions: 665

Charge : +1

Number of electrons: 110

Relative energy: 0.2 kcal/mol

SCF total energy (ωB97X-V/gas): –657.298953 hartrees

Table S2. Cartesian Coordinates (Angstroms) for **18a**

Atom	X	Y	Z
C	-1.058565	0.299475	2.013373
C	-0.390001	1.580317	1.546822
H	0.630759	1.678771	1.917219
H	-0.966295	2.474394	1.780148
O	-0.287328	1.596984	0.084341
C	-0.239026	0.518457	-0.605826
O	-0.246828	-0.651564	-0.091023
C	-0.250933	-0.843350	1.424104
H	-0.732248	-1.823278	1.493915
O	1.019854	-0.811311	1.861566
C	1.824876	-1.981013	1.584388
H	1.306574	-2.858892	1.988318
H	1.917156	-2.094670	0.498818
C	3.172069	-1.779461	2.238386
H	3.065154	-1.659923	3.319387
H	3.673443	-0.896707	1.832915
H	3.803372	-2.651427	2.047681

H	-0.933596	0.243668	3.099395
C	-0.257941	0.640823	-2.104441
C	0.338677	1.986172	-2.541958
H	1.386414	2.077640	-2.240309
H	-0.217326	2.833651	-2.134788
H	0.296512	2.044852	-3.632638
C	-1.758343	0.569237	-2.491272
H	-2.326726	1.393780	-2.049693
H	-2.206266	-0.382689	-2.190293
H	-1.834044	0.651286	-3.579013
C	0.508434	-0.532258	-2.734684
H	1.563471	-0.522704	-2.443331
H	0.460688	-0.434054	-3.822235
H	0.076309	-1.497523	-2.461087
C	-2.545248	0.221712	1.659279
H	-3.090394	1.033555	2.147376
H	-2.975904	-0.722637	2.003156
H	-2.726750	0.298687	0.582003

Cis-dioxocarbenium ion **16b**

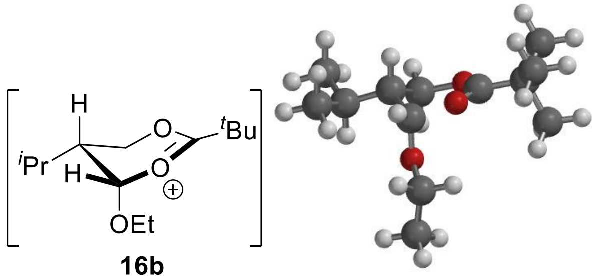

16b

Spartan '24: ωB97X-V/6-311+G(2df,2p)

Number of imaginary vibrations: 0

Number of basis functions: 769

Charge : +1

Number of electrons: 126

Relative energy: 0.0 kcal/mol

SCF total energy (ωB97X-V/gas): –735.933142 hartrees

Table S3. Cartesian Coordinates (Angstroms) for **16b**

**Atom**	**X**	**Y**	**Z**
C	0.757685	-1.363084	0.377773
C	1.681524	-0.432893	-0.387233
H	2.477888	-0.954278	-0.913255
H	2.098845	0.348173	0.251100
O	0.966579	0.268599	-1.457628
C	-0.299023	0.460583	-1.427526
O	-1.049612	0.065612	-0.472761
C	-0.451217	-0.545142	0.791701
H	-1.302890	-1.137374	1.134310
O	-0.098463	0.456361	1.618621
C	-1.172867	1.114465	2.325106
H	-1.812943	1.625068	1.597224
H	-1.767149	0.348884	2.838841
C	-0.556085	2.087657	3.302647
H	0.041433	2.838970	2.779575
H	0.082821	1.564660	4.018770
H	-1.347225	2.601233	3.855575
H	0.406276	-2.158395	-0.292794
C	-0.915054	1.239451	-2.557262
C	-0.732978	2.726938	-2.156678
H	0.323967	2.993631	-2.066317
H	-1.243799	2.956214	-1.216213
H	-1.175185	3.346267	-2.941842
C	-2.407381	0.903634	-2.683441
H	-2.959488	1.139552	-1.770667
H	-2.562638	-0.152724	-2.922455
H	-2.828588	1.498141	-3.498207

C	-0.172003	0.949536	-3.870457
H	0.885439	1.216588	-3.810802
H	-0.628494	1.544774	-4.665502
H	-0.253357	-0.104841	-4.151779
C	1.468083	-2.012373	1.588083
H	1.852785	-1.200309	2.216577
C	2.644067	-2.881966	1.129898
H	3.105445	-3.368076	1.993639
H	3.430622	-2.305852	0.632104
H	2.309136	-3.670462	0.445690
C	0.483697	-2.837488	2.424051
H	1.016546	-3.346215	3.231787
H	-0.008863	-3.606383	1.817060
H	-0.287534	-2.217126	2.892672

Trans-dioxocarbenium ion **18b**

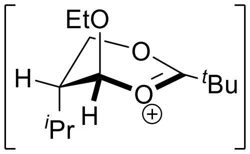

18b

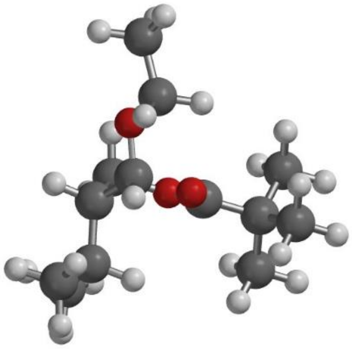

Spartan ’24: ωB97X-V/6-311+G(2df,2p)

Number of imaginary vibrations: 0

Number of basis functions: 769

Charge : +1

Number of electrons: 126

Relative energy: 0.8 kcal/mol

SCF total energy (ωB97X-V/gas): –735.931829 hartrees

Table S4. Cartesian Coordinates (Angstroms) for **18b**

Atom	X	Y	Z
C	-1.181764	-1.284518	-0.233081
C	-0.737360	-0.781289	-1.594612
H	-1.434191	-0.048405	-2.003830
H	-0.560437	-1.571814	-2.319318
O	0.552379	-0.100014	-1.481041
C	0.870685	0.538720	-0.414241
O	0.110392	0.628450	0.605416
C	-1.274705	-0.047748	0.645265
H	-1.345553	-0.242246	1.718213
O	-2.184393	0.820440	0.176891
C	-2.544051	1.915617	1.050619
H	-2.851031	1.499160	2.017538
H	-1.662154	2.546853	1.203119
C	-3.668341	2.678979	0.389864
H	-4.537961	2.034766	0.238395
H	-3.351362	3.078063	-0.577244
H	-3.964929	3.516661	1.026854
H	-2.202540	-1.664799	-0.356190
C	2.231256	1.181817	-0.385062
C	2.446564	1.924494	0.937634
H	2.394195	1.250012	1.796575
H	1.713198	2.723633	1.078212
H	3.440787	2.378380	0.923961
C	2.319003	2.156590	-1.579142
H	1.577527	2.957644	-1.496923
H	2.182910	1.642312	-2.533601
H	3.310958	2.616011	-1.575772
C	3.269852	0.048445	-0.547658
H	3.205682	-0.675570	0.271493
H	4.269084	0.491362	-0.525096
H	3.148469	-0.476604	-1.498409

C	-0.301434	-2.391477	0.400233
H	0.601477	-1.924056	0.819210
C	0.149443	-3.435627	-0.625363
H	-0.707247	-3.868913	-1.154956
H	0.667887	-4.253628	-0.118058
H	0.844191	-3.024750	-1.365146
C	-1.055606	-3.069895	1.550272
H	-0.407852	-3.791868	2.054415
H	-1.928529	-3.612585	1.170464
H	-1.404498	-2.362602	2.310101

Trans-dioxocarbenium ion **18c**

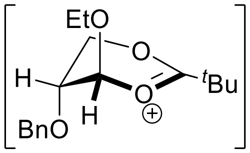

18c

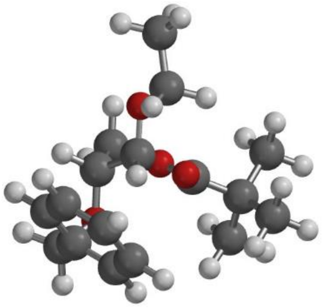

Spartan '20: ωB97X-V/6-311+G(2df,2p)

Number of imaginary vibrations: 0

Number of basis functions: 939

Charge : +1

Number of electrons: 158

Relative energy: 0 kcal/mol

SCF total energy (ωB97X-V/gas): –963.571368 hartrees

Table S5. Cartesian Coordinates (Angstroms) for **18c**

**Atom**	**X**	**Y**	**Z**
C	0.790138	0.169776	1.601373
C	1.855634	1.210496	1.333748
H	2.803671	0.766410	1.030034
H	1.985289	1.884809	2.177235

O	1.421020	2.073349	0.240148
C	0.610403	1.661739	-0.665758
O	0.168884	0.467810	-0.733725
C	0.566104	-0.584475	0.291766
H	-0.321147	-1.217711	0.282057
O	1.701222	-1.187269	-0.103831
C	1.550390	-2.222159	-1.103970
H	0.780805	-2.922605	-0.757489
H	1.211721	-1.759474	-2.037230
C	2.890300	-2.899559	-1.270903
H	3.202091	-3.376397	-0.338263
H	3.655063	-2.178929	-1.572272
H	2.818691	-3.668487	-2.044839
H	1.161111	-0.541718	2.350633
C	0.185730	2.661240	-1.707981
C	1.445860	3.016675	-2.530988
H	1.851697	2.138438	-3.043590
H	2.226179	3.457866	-1.905350
H	1.162561	3.748992	-3.291890
C	-0.348564	3.914148	-0.984443
H	0.418371	4.381536	-0.362382
H	-1.214006	3.674087	-0.358883
H	-0.665343	4.637823	-1.740443
C	-0.893192	2.059998	-2.615056
H	-0.533551	1.176567	-3.148798
H	-1.179835	2.809776	-3.356422
H	-1.787298	1.783260	-2.049773
O	-0.363556	0.847968	2.004314
C	-1.222180	0.133690	2.910741
H	-0.660808	-0.101039	3.824381
H	-2.001571	0.855816	3.160424

C	-1.789881	-1.115085	2.291243
C	-2.648615	-3.395445	0.923160
C	-1.281333	-2.370917	2.624253
C	-2.748970	-1.011672	1.278685
C	-3.176190	-2.145485	0.597766
C	-1.707198	-3.509446	1.941840
H	-0.548389	-2.464357	3.422623
H	-3.152483	-0.036499	1.017824
H	-3.924584	-2.060136	-0.183467
H	-1.308736	-4.482768	2.210316
H	-2.985507	-4.280634	0.393181

Cis-dioxocarbenium ion **16c**

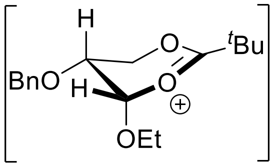

16c

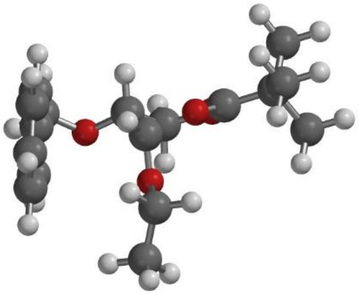

Spartan ’20: ωB97X-V/6-311+G(2df,2p)

Number of imaginary vibrations: 0

Number of basis functions: 939

Charge : +1

Number of electrons: 158

Relative energy: 3.51 kcal/mol

SCF total energy (ωB97X-V/gas): –963.565781 hartrees

Table S6. Cartesian Coordinates (Angstroms) for **16c**

**Atom**	**X**	**Y**	**Z**
C	0.224837	1.130400	1.267081
C	-1.131381	1.818892	1.277613
H	-1.105614	2.770404	1.803807

H	-1.886998	1.161500	1.709209
O	-1.572803	2.147044	-0.071264
C	-1.118262	1.550476	-1.113932
O	-0.268581	0.603187	-1.068351
C	0.140046	-0.037589	0.278246
H	1.098093	-0.478545	-0.001722
O	-0.805206	-0.908097	0.641271
C	-0.766512	-2.206765	0.005057
H	-1.150194	-2.103267	-1.015850
H	0.277595	-2.537206	-0.034960
C	-1.609838	-3.149367	0.830627
H	-1.617395	-4.138282	0.363820
H	-2.641278	-2.794361	0.902588
H	-1.199756	-3.245029	1.839410
H	1.008530	1.818656	0.912908
C	-1.634865	2.029366	-2.445356
C	-0.971300	1.254618	-3.588852
H	-1.180801	0.183125	-3.530499
H	0.113032	1.394102	-3.600465
H	-1.369278	1.625445	-4.536800
C	-1.327972	3.537777	-2.559049
H	-0.250352	3.728749	-2.540358
H	-1.805433	4.109230	-1.759446
H	-1.713563	3.898408	-3.516284
C	-3.163419	1.795717	-2.444963
H	-3.407593	0.733360	-2.342404
H	-3.561199	2.140944	-3.403011
H	-3.658893	2.352894	-1.645550
O	0.432510	0.739599	2.582554
C	1.792283	0.481152	2.952304
H	2.413010	1.355658	2.712153

H	1.752467	0.379312	4.038696
C	2.342070	-0.762606	2.300347
C	3.258291	-3.066406	1.008779
C	3.418185	-0.693806	1.415112
C	1.731238	-1.997013	2.541046
C	2.188001	-3.143330	1.900574
C	3.875712	-1.842160	0.770311
H	3.906745	0.260789	1.230900
H	0.891971	-2.053424	3.229047
H	1.717393	-4.100617	2.103046
H	4.718941	-1.780413	0.089988
H	3.617486	-3.962501	0.512637

Cis-dioxocarbenium ion **S47**

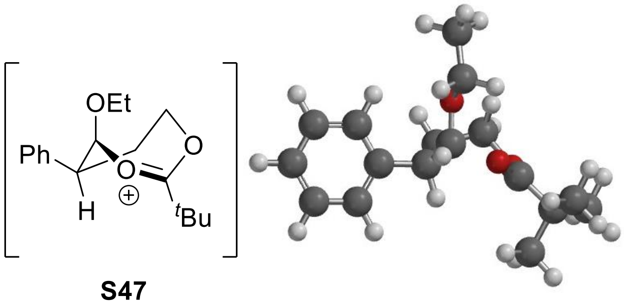

S47

Spartan '24: ωB97X-V/6-311+G(2df,2p)

Number of imaginary vibrations: 0

Number of basis functions: 905

Charge : +1

Number of electrons: 150

Relative energy: 0.0 kcal/mol

SCF total energy (ωB97X-V/gas): –888.350130 hartrees

Table S7. Cartesian Coordinates (Angstroms) for **S47**

**Atom**	**X**	**Y**	**Z**
C	-0.695234	-0.085593	0.336665
O	-1.442697	0.983317	0.613523

C	-2.184723	0.952953	1.856013
H	-2.780963	0.033667	1.873375
H	-1.470329	0.931256	2.685649
C	-3.058262	2.184733	1.898382
H	-3.751070	2.196838	1.052873
H	-2.453325	3.095166	1.873731
H	-3.642767	2.186488	2.822343
H	-1.074894	-1.020797	0.755245
C	-0.318228	-0.204491	-1.130257
C	0.138465	1.138264	-1.725830
H	0.702876	0.938683	-2.640575
H	-0.729084	1.741323	-2.010732
C	0.948773	1.982219	-0.762187
H	0.330039	2.402049	0.031671
H	1.493865	2.777850	-1.266642
O	2.013959	1.215148	-0.107858
C	1.790617	0.376737	0.838811
O	0.640932	-0.032663	1.197661
C	3.000008	-0.207657	1.526265
C	3.313859	-1.500038	0.728914
H	3.559912	-1.279173	-0.314565
H	2.477577	-2.205594	0.762310
H	4.181962	-1.983111	1.186012
C	4.190035	0.758210	1.461491
H	4.478186	0.985312	0.432639
H	5.045126	0.293121	1.959236
H	3.971013	1.697755	1.977917
C	2.658608	-0.558434	2.982622
H	1.837008	-1.274955	3.049026
H	2.392684	0.334485	3.556798
H	3.540557	-1.006336	3.448054

H	-2.849278	0.786087	-1.561193
C	-2.700410	-0.189784	-2.015026
C	-2.344763	-2.696692	-3.172714
C	-1.468327	-0.838781	-1.894750
C	-3.744016	-0.791144	-2.709829
C	-3.568118	-2.044857	-3.290393
C	-1.300984	-2.096319	-2.475286
H	-4.696632	-0.278960	-2.799656
H	-4.383036	-2.511350	-3.834232
H	-0.346644	-2.610734	-2.390505
H	-2.200143	-3.672426	-3.624995
H	0.521170	-0.909162	-1.169369

Trans-dioxocarbenium ion **47**

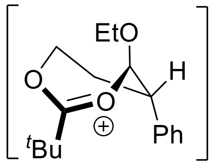

47

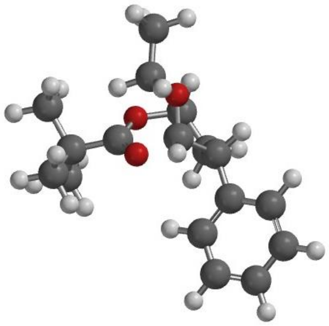

Spartan '24: ωB97X-V/6-311+G(2df,2p)

Number of imaginary vibrations: 0

Number of basis functions: 905

Charge : +1

Number of electrons: 150

Relative energy: 0.9 kcal/mol

SCF total energy (ωB97X-V/gas): –888.348671 hartrees

Table S8. Cartesian Coordinates (Angstroms) for **47**

**Atom**	**X**	**Y**	**Z**
C	0.004755	-0.136114	1.202989

O	0.524190	0.929448	1.852699
C	-0.427646	1.843241	2.438777
H	-1.068240	2.250431	1.646656
H	-1.056643	1.282427	3.139684
C	0.348813	2.937115	3.134577
H	0.968493	3.489177	2.422352
H	0.992805	2.519615	3.912531
H	-0.346275	3.639725	3.601908
H	-0.917675	-0.516584	1.649641
C	1.044437	-1.229061	0.974683
C	1.998651	-0.895883	-0.187446
H	2.929404	-1.452659	-0.042964
H	1.586643	-1.253579	-1.138161
C	2.351757	0.570496	-0.322100
H	3.198387	0.723631	-0.989399
H	2.529355	1.070545	0.629170
O	1.292087	1.335937	-1.001470
C	0.032667	1.119986	-0.956151
O	-0.567961	0.349693	-0.134149
C	-0.798465	1.838926	-1.992837
C	-0.343623	1.329182	-3.377837
H	-0.514238	0.252616	-3.484373
H	0.712846	1.542327	-3.559078
H	-0.932178	1.837692	-4.146140
C	-2.290103	1.561107	-1.780721
H	-2.524269	0.496983	-1.870484
H	-2.857290	2.098187	-2.545459
H	-2.633363	1.908118	-0.801634
C	-0.507429	3.349802	-1.862176
H	0.548358	3.575366	-2.028718
H	-0.801269	3.730943	-0.878489

H	-1.095513	3.880398	-2.615769
H	1.345190	-3.403830	2.574355
C	0.676489	-3.601938	1.740317
C	-1.036489	-4.126067	-0.386105
C	0.407021	-2.596160	0.812488
C	0.098509	-4.861337	1.606391
C	-0.759911	-5.124916	0.544158
C	-0.454895	-2.870251	-0.253795
H	0.319483	-5.635136	2.334335
H	-1.211691	-6.105853	0.439108
H	-0.682145	-2.102170	-0.989647
H	-1.705642	-4.326143	-1.216847
H	1.622613	-1.225435	1.905131

Cis-dioxocarbenium ion **60**

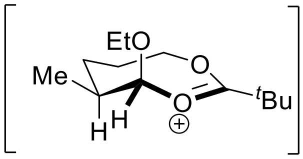

60

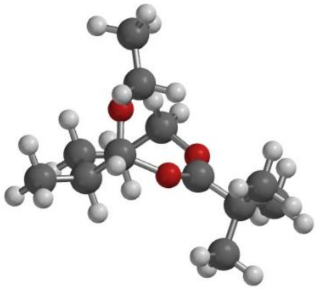

Spartan '24: ωB97X-V/6-311+G(2df,2p)

Number of imaginary vibrations: 0

Number of basis functions: 769

Charge : +1

Number of electrons: 126

Relative energy: 0.0 kcal/mol

SCF total energy (ωB97X-V/gas): –735.918786 hartrees

Table S9. Cartesian Coordinates (Angstroms) for **60**

**Atom**	**X**	**Y**	**Z**
C	-1.936287	1.069203	1.874582

H	-1.900121	1.724996	0.996822
H	-1.486123	1.596173	2.723837
C	0.319850	-2.478577	1.211358
C	0.044569	-2.681580	-0.278338
H	0.969559	-2.635120	-0.866448
H	-0.339008	-3.698342	-0.411766
C	-0.981687	-1.740674	-0.898418
H	-1.668530	-1.311356	-0.169925
H	-1.539489	-2.224811	-1.697412
O	-0.346270	-0.637948	-1.630140
C	0.273331	0.382802	-1.180961
O	0.464248	0.708571	0.033513
C	0.853563	1.330948	-2.212828
C	0.535798	0.864119	-3.637015
H	0.972205	1.576572	-4.342434
H	0.961059	-0.121152	-3.846347
H	-0.541136	0.822940	-3.821931
C	0.247135	2.726479	-1.956380
H	0.491800	3.094099	-0.956660
H	0.658887	3.426049	-2.688857
H	-0.841385	2.718380	-2.077904
C	2.380595	1.365577	-1.988680
H	2.829068	0.377095	-2.134782
H	2.633284	1.727547	-0.988961
H	2.825289	2.044339	-2.721386
C	1.007200	-1.176391	1.635895
H	1.935717	-1.041677	1.066930
C	0.169702	0.079713	1.425881
O	-1.146157	-0.110779	1.604357
C	-3.346816	0.621700	2.180437
H	-3.793376	0.110028	1.323184

H	-0.616254	-2.583182	1.771158
H	-3.963048	1.493102	2.416576
H	-3.361071	-0.052950	3.040201
H	0.965604	-3.299453	1.538527
C	1.359430	-1.230767	3.131394
H	1.968914	-2.112669	3.341537
H	1.928427	-0.350440	3.445239
H	0.452661	-1.294207	3.741775
H	0.558862	0.921642	2.004368

Trans-dioxocarbenium ion **61**

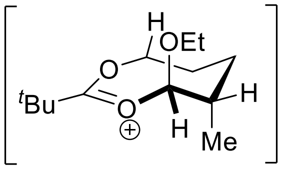

61

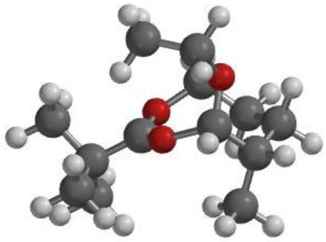

Spartan '24: ωB97X-V/6-311+G(2df,2p)

Number of imaginary vibrations: 0

Number of basis functions: 769

Charge : +1

Number of electrons: 126

Relative energy: 0.4 kcal/mol

SCF total energy (ωB97X-V/gas): –735.918183 hartrees

Table S10. Cartesian Coordinates (Angstroms) for **61**

**Atom**	**X**	**Y**	**Z**
C	-1.144355	-2.164868	2.243610
H	-0.928565	-3.195169	2.528076
H	-2.223714	-1.997073	2.336734
C	-0.231418	-2.347794	-1.899890
C	1.155824	-1.706996	-1.960592

H	1.184474	-0.845663	-2.637955
H	1.842361	-2.443318	-2.390590
C	1.737274	-1.292924	-0.615340
H	1.329937	-1.860777	0.220619
H	2.824188	-1.349815	-0.611014
O	1.527976	0.132158	-0.345466
C	0.480645	0.733724	0.075469
O	-0.655193	0.230061	0.328607
C	0.656544	2.222423	0.308951
C	1.741593	2.393168	1.393850
H	1.880385	3.460717	1.585199
H	2.699114	1.975406	1.073660
H	1.443739	1.920010	2.335548
C	1.128860	2.854551	-1.017220
H	1.266798	3.927996	-0.862128
H	2.079822	2.432620	-1.350319
H	0.383614	2.725957	-1.809465
C	-0.658940	2.860926	0.764370
H	-0.493336	3.930903	0.917206
H	-1.012036	2.436752	1.708768
H	-1.446591	2.743936	0.014246
C	-1.397010	-1.487597	-1.394298
H	-2.284326	-2.133195	-1.448606
C	-1.350524	-1.151363	0.092992
O	-0.790118	-2.109336	0.842793
C	-0.349379	-1.188415	3.090001
H	-0.571721	-1.367750	4.145733
H	-0.494041	-2.673198	-2.911856
H	-0.607420	-0.149601	2.866025
H	0.726099	-1.332937	2.945513
H	-0.175624	-3.255188	-1.290758

C	-1.680478	-0.256710	-2.258739
H	-1.911212	-0.571580	-3.279719
H	-0.819382	0.417821	-2.313436
H	-2.534217	0.315902	-1.882939
H	-2.329647	-0.833763	0.462359

Cis-oxocarbenium ion **62**

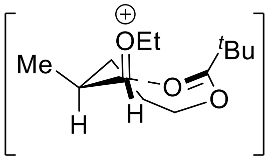

62

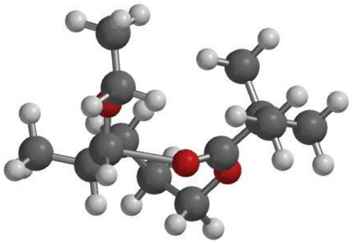

Spartan ’24: ωB97X-V/6-311+G(2df,2p)

Number of imaginary vibrations: 0

Number of basis functions: 769

Charge : +1

Number of electrons: 126

Relative energy: 0.7 kcal/mol

SCF total energy (ωB97X-V/gas): –735.917705 hartrees

Table S11. Cartesian Coordinates (Angstroms) for **62**

**Atom**	**X**	**Y**	**Z**
C	-0.277186	0.913510	2.696421
H	-0.206444	1.689222	1.932502
H	0.502954	1.052457	3.449838
C	0.472541	-2.333461	-0.146042
C	1.051586	-2.382327	-1.563678
H	1.887124	-3.092971	-1.598857
H	0.286026	-2.767021	-2.245179
C	1.570803	-1.060315	-2.106823
H	1.960299	-1.193336	-3.116346
H	2.353745	-0.623375	-1.482956

O	0.508809	-0.098496	-2.255698
C	0.285257	0.777312	-1.296948
O	0.917941	0.801887	-0.240167
C	-0.873826	1.718492	-1.586375
C	-1.116047	1.881389	-3.091500
H	-1.942413	2.580612	-3.247757
H	-0.233036	2.286890	-3.593415
H	-1.375259	0.934183	-3.569139
C	-2.102459	1.058516	-0.921949
H	-1.945691	0.919917	0.153650
H	-2.978683	1.699438	-1.058025
H	-2.322710	0.084440	-1.371302
C	-0.594637	3.084212	-0.944128
H	0.286844	3.558361	-1.386213
H	-0.429480	3.004762	0.133598
H	-1.451711	3.743650	-1.109006
C	1.495592	-1.844660	0.906754
H	2.487250	-1.743856	0.455343
C	1.167123	-0.525685	1.495506
O	0.019572	-0.352531	1.996670
C	-1.651670	0.788695	3.299781
H	-2.409854	0.624748	2.529809
H	-0.425223	-1.706518	-0.125573
H	-1.888571	1.719999	3.821197
H	-1.694831	-0.029990	4.021952
H	0.139989	-3.339231	0.125620
C	1.619056	-2.816342	2.111421
H	1.973126	-3.781258	1.740276
H	2.336324	-2.463588	2.859213
H	0.646061	-2.959893	2.589595
H	1.951708	0.192162	1.737931

Cis-oxocarbenium ion **69**

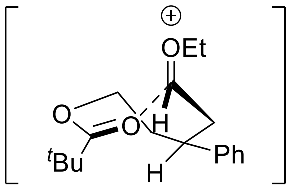

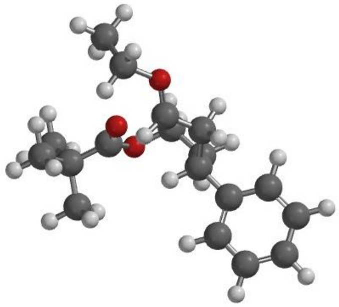

69

Spartan ’24: ωB97X-V/6-311+G(2df,2p)

Number of imaginary vibrations: 0

Number of basis functions: 957

Charge : +1

Number of electrons: 158

Relative energy: 0.0 kcal/mol

SCF total energy (ωB97X-V/gas): –927.661585 hartrees

Table S12. Cartesian Coordinates (Angstroms) for **69**

**Atom**	**X**	**Y**	**Z**
C	2.382581	-2.610325	1.840132
H	1.743835	-2.108086	2.568290
H	2.430679	-3.679604	2.043681
C	3.741323	-1.971294	1.711074
H	4.340375	-2.472948	0.949789
H	4.259082	-2.064957	2.668177
H	3.648479	-0.912134	1.469320
C	-0.950458	-0.667608	-1.134753
C	-0.217032	0.258918	-2.121701
H	-0.075352	-0.274696	-3.068583
H	-0.864663	1.111512	-2.344882
C	1.138329	0.781113	-1.678099
H	1.609253	1.333349	-2.490908

H	1.819827	-0.009551	-1.354599
O	1.005908	1.751642	-0.617679
C	1.104609	1.351524	0.638767
O	1.242275	0.174656	0.949375
C	1.020352	2.493893	1.636896
C	-0.366784	3.145764	1.488669
H	-1.167536	2.426603	1.689201
H	-0.508232	3.560072	0.488929
H	-0.459561	3.957881	2.213492
C	1.199216	1.945537	3.053231
H	0.430961	1.207955	3.296439
H	1.122201	2.764941	3.771145
H	2.177471	1.475231	3.177091
C	2.121461	3.518248	1.315503
H	1.999552	3.936614	0.315441
H	3.115627	3.067875	1.386755
H	2.071669	4.335376	2.038632
H	-1.636990	-2.331167	-3.218479
C	-2.499941	-1.866469	-2.747011
C	-4.729936	-0.688633	-1.586402
C	-2.334085	-1.001085	-1.665043
C	-3.767966	-2.143516	-3.240570
C	-4.886546	-1.554315	-2.660545
C	-3.458831	-0.414727	-1.091949
H	-3.883634	-2.819546	-4.080495
H	-5.876344	-1.770958	-3.046202
H	-3.345218	0.264495	-0.250914
H	-5.597801	-0.226774	-1.128711
H	-1.087321	-0.134213	-0.188279
C	-0.118721	-1.949440	-0.861138
H	-0.810949	-2.803705	-0.780129

H	0.588050	-2.188410	-1.659261
C	0.574383	-1.981340	0.434105
H	0.051512	-1.699983	1.348267
O	1.704889	-2.517715	0.523931

Trans-oxocarbenium ion **70**

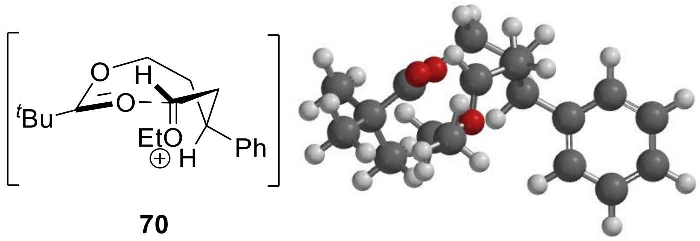

70

Spartan ’24: ωB97X-V/6-311+G(2df,2p)

Number of imaginary vibrations: 0

Number of basis functions: 957

Charge : +1

Number of electrons: 158

Relative energy: 1.4 kcal/mol

SCF total energy (ωB97X-V/gas): –927.659380 hartrees

Table S13. Cartesian Coordinates (Angstroms) for **70**

**Atom**	**X**	**Y**	**Z**
C	1.056609	-1.765345	2.544117
H	2.002195	-1.222005	2.509597
H	0.345638	-1.226628	3.167805
C	1.192646	-3.217162	2.930214
H	0.226945	-3.723539	2.905017
H	1.582178	-3.272174	3.948895
H	1.890810	-3.742364	2.273577
C	-0.512385	-0.569678	-1.338381
C	-0.133517	0.471363	-2.405484
H	-0.000622	-0.027777	-3.372280
H	-0.972650	1.162758	-2.525147

C	1.127150	1.270959	-2.120670
H	1.323605	1.963996	-2.938175
H	2.010013	0.645260	-1.975598
O	0.965508	2.116548	-0.961334
C	1.375853	1.685717	0.220063
O	1.826377	0.561064	0.404494
C	1.224525	2.733698	1.311151
C	-0.262049	3.122382	1.400884
H	-0.885115	2.259565	1.658473
H	-0.621860	3.545588	0.461982
H	-0.389065	3.870358	2.186766
C	1.699496	2.157037	2.645093
H	1.090905	1.299892	2.944999
H	1.608042	2.920271	3.420841
H	2.743684	1.840597	2.597238
C	2.067015	3.963095	0.929815
H	1.732350	4.396526	-0.013737
H	3.126877	3.708302	0.842295
H	1.966898	4.720211	1.710799
H	-1.104928	-2.307973	-3.386381
C	-1.966583	-2.083104	-2.761733
C	-4.194253	-1.522630	-1.202915
C	-1.840905	-1.224650	-1.668797
C	-3.192130	-2.657661	-3.070438
C	-4.309611	-2.377467	-2.290802
C	-2.964206	-0.950273	-0.894621
H	-3.277096	-3.322249	-3.923189
H	-5.266802	-2.824408	-2.533784
H	-2.880050	-0.281963	-0.041504
H	-5.061850	-1.299088	-0.591997
H	-0.643226	-0.046722	-0.386824

C	0.634642	-1.605730	-1.159279
H	0.244240	-2.623654	-1.304538
H	1.442355	-1.475329	-1.880872
C	1.220343	-1.624417	0.192418
H	2.292729	-1.748414	0.351170
O	0.459289	-1.672777	1.190777

Trans-dioxocarbenium ion **S34**

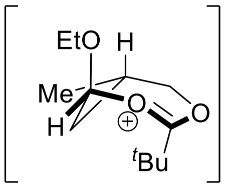

S34

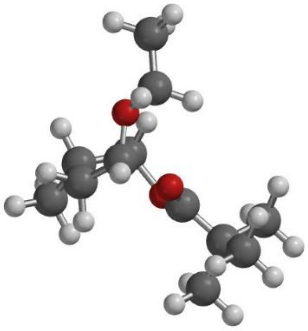

Spartan ’24: ωB97X-V/6-311+G(2df,2p)

Number of imaginary vibrations: 0

Number of basis functions: 717

Charge : +1

Number of electrons: 118

Relative energy: 0.0 kcal/mol

SCF total energy (ωB97X-V/gas): –696.608143 hartrees

Table S14. Cartesian Coordinates (Angstroms) for **S34**

Atom	X	Y	Z
C	-1.153087	-0.753733	1.012294
O	-2.204625	0.037192	0.789070
C	-2.556763	0.964328	1.843978
H	-2.666320	0.398643	2.776668
H	-1.738253	1.682648	1.960358
C	-3.845966	1.644199	1.447020
H	-4.644746	0.910998	1.312275

H	-3.720852	2.205454	0.517533
H	-4.147873	2.342587	2.231599
H	-1.034759	-1.052140	2.057350
C	-1.054083	-1.902366	0.039250
C	-1.275846	-1.527372	-1.437079
H	-2.354993	-1.464362	-1.618415
C	-0.757102	-0.133133	-1.748830
H	-1.352286	0.646617	-1.272930
H	-0.694858	0.049483	-2.821006
O	0.628258	0.059972	-1.302148
C	0.951758	0.260185	-0.075082
O	0.184075	0.089398	0.924498
C	2.365866	0.736610	0.162805
C	2.551337	2.071180	-0.589918
H	1.876124	2.844200	-0.208430
H	2.390160	1.956183	-1.664615
H	3.576633	2.416798	-0.432807
C	2.628459	0.922076	1.659692
H	1.963050	1.669526	2.099981
H	3.657789	1.264320	1.793029
H	2.509161	-0.014958	2.210350
C	3.317567	-0.333733	-0.417890
H	3.163162	-0.479109	-1.489743
H	3.201611	-1.295081	0.093588
H	4.345429	0.004683	-0.262433
H	-0.076184	-2.379178	0.172859
H	-1.797357	-2.644937	0.346224
C	-0.672929	-2.593257	-2.353589
H	-0.929125	-2.404113	-3.400034
H	-1.051771	-3.584662	-2.089319
H	0.419337	-2.615146	-2.266152

Cis-dioxocarbenium ion **S35**

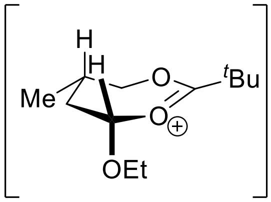

S35

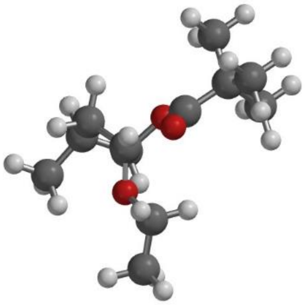

Spartan '24: ωB97X-V/6-311+G(2df,2p)

Number of imaginary vibrations: 0

Number of basis functions: 717

Charge : +1

Number of electrons: 118

Relative energy: 0.6 kcal/mol

SCF total energy (ωB97X-V/gas): –696.607207 hartrees

Table S15. Cartesian Coordinates (Angstroms) for **S35**

**Atom**	**X**	**Y**	**Z**
C	-0.010368	-1.402872	0.777290
O	1.172686	-1.740227	0.253664
C	2.320713	-1.646296	1.129017
H	2.447505	-0.599747	1.425426
H	2.119310	-2.247030	2.023427
C	3.520667	-2.159425	0.367381
H	3.706983	-1.551429	-0.522173
H	3.370905	-3.198107	0.061746
H	4.407087	-2.113075	1.005710
H	-0.111979	-1.653430	1.836766
C	-1.192465	-1.848312	-0.050555
C	-1.082818	-1.577764	-1.564867
H	-2.099965	-1.432898	-1.943728
C	-0.287120	-0.317151	-1.860392

H	-0.410620	0.006889	-2.892820
H	0.773641	-0.437746	-1.633850
O	-0.756989	0.846545	-1.098934
C	-0.531026	1.033623	0.150398
O	-0.082010	0.159438	0.958455
C	-0.840699	2.416983	0.674956
C	-2.345090	2.672780	0.427680
H	-2.969468	1.964696	0.982567
H	-2.597372	2.617507	-0.634057
H	-2.583238	3.678125	0.785190
C	-0.524314	2.511932	2.170018
H	-1.119127	1.806345	2.756246
H	-0.764059	3.521307	2.513578
H	0.534061	2.328111	2.373997
C	0.004403	3.430634	-0.125102
H	-0.218294	3.389439	-1.194080
H	1.076559	3.265646	0.023885
H	-0.230957	4.435905	0.234338
H	-1.310679	-2.922526	0.125531
H	-2.085330	-1.374974	0.370564
C	-0.440492	-2.741772	-2.330960
H	-0.364364	-2.517047	-3.399380
H	-1.055017	-3.639859	-2.228950
H	0.559340	-2.964219	-1.947984

Substitution product ***syn*-9a**

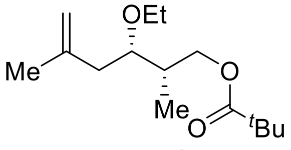

***syn*-9a**

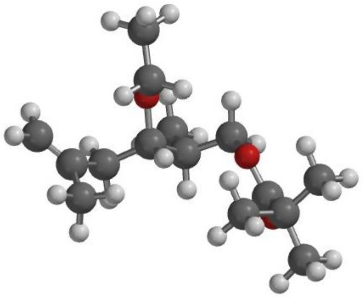

Spartan ’24: ωB97X-V/6-311+G(2df,2p)

Number of imaginary vibrations: 0

Number of basis functions: 326

Charge : 0

Number of electrons: 142

SCF total energy (ωB97X-V/gas): –813.899931 hartrees

Table S16. Cartesian Coordinates (Angstroms) for ***syn*-9a**

**Atom**	**X**	**Y**	**Z**
C	0.938638	-1.528995	-2.040202
H	0.026238	-2.142107	-2.092729
H	1.792966	-2.212422	-2.080834
C	0.956864	-0.817543	-0.668491
H	0.199037	-0.022712	-0.667777
C	0.623220	-1.771432	0.499883
H	-0.351760	-2.228820	0.287030
O	2.241731	-0.238801	-0.428600
C	0.979886	-0.595763	-3.233048
C	2.084853	-0.461769	-3.974960
H	2.991746	-1.017528	-3.750737
H	2.121423	0.205815	-4.832751
C	-0.290693	0.155653	-3.553138
H	-0.632004	0.761572	-2.702989
H	-1.108743	-0.540399	-3.785775
H	-0.159135	0.823806	-4.409841
C	1.665544	-2.886113	0.682704

H	2.664653	-2.461407	0.823565
H	1.425870	-3.504002	1.555845
H	1.701419	-3.550691	-0.186116
C	0.491965	-0.999205	1.817172
H	0.224468	-1.676608	2.631694
H	1.427515	-0.486603	2.050889
O	-0.497194	0.056541	1.750085
C	2.315251	1.175907	-0.571481
H	2.005679	1.473058	-1.583260
H	1.633505	1.662250	0.145300
C	3.751461	1.601596	-0.316020
H	4.070256	1.309122	0.690290
H	3.849512	2.688929	-0.409134
H	4.423267	1.128047	-1.039119
C	-1.782836	-0.288822	2.009317
C	-2.715119	0.928693	1.948458
O	-2.127905	-1.422313	2.269424
C	-2.311717	1.915002	3.067748
H	-1.281130	2.259501	2.942308
H	-2.973967	2.788184	3.046276
H	-2.400957	1.449761	4.056288
C	-4.161520	0.454714	2.158918
H	-4.464763	-0.252852	1.380806
H	-4.840483	1.314558	2.128608
H	-4.277088	-0.047111	3.123878
C	-2.576794	1.617636	0.573655
H	-2.826106	0.928589	-0.242219
H	-3.266789	2.467217	0.516052
H	-1.560268	1.987863	0.413028

Substitution product ***anti*-9a**

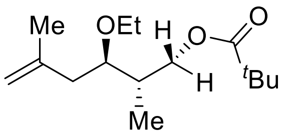

***anti*-9a**

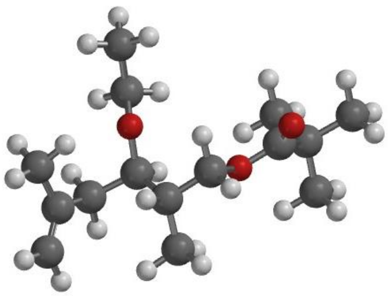

Spartan ’24: ωB97X-V/6-311+G(2df,2p)

Number of imaginary vibrations: 0

Number of basis functions: 326

Charge : 0

Number of electrons: 142

SCF total energy (ωB97X-V/gas): –813.900655 hartrees

Table S17. Cartesian Coordinates (Angstroms) for ***anti*-9a**

**Atom**	**X**	**Y**	**Z**
C	1.801277	1.945569	-1.238269
H	1.875145	1.685650	-2.304449
H	1.196206	2.858187	-1.189312
C	1.014204	0.800611	-0.550975
H	-0.004954	0.809025	-0.967296
C	0.898557	0.931749	0.982649
H	1.918613	0.918934	1.392319
O	1.609394	-0.471232	-0.825247
C	3.190710	2.232160	-0.701780
C	3.478508	3.404499	-0.123430
H	2.732992	4.188094	-0.009577
H	4.475861	3.630143	0.246671
C	4.244530	1.166395	-0.886822
H	5.210491	1.486836	-0.483953
H	4.378087	0.936306	-1.953350
H	3.950847	0.227042	-0.407963
C	0.223195	2.244299	1.409281

H	0.830562	3.112734	1.142423
H	0.081836	2.262720	2.496117
H	-0.763848	2.355359	0.946435
C	0.195583	-0.281983	1.588714
H	0.151984	-0.207608	2.680455
H	0.705902	-1.208825	1.324536
O	-1.159351	-0.332573	1.076589
C	1.179433	-1.096196	-2.026732
H	0.087582	-1.244857	-1.998104
H	1.399034	-0.460744	-2.899977
C	1.897865	-2.429733	-2.154772
H	1.683577	-3.066034	-1.289958
H	2.980995	-2.280774	-2.210753
H	1.571681	-2.952407	-3.060619
C	-1.912993	-1.376832	1.493098
C	-3.313801	-1.356289	0.865049
O	-1.504047	-2.218823	2.264717
C	-4.004349	-0.014372	1.190429
H	-5.013139	-0.001610	0.760735
H	-3.445887	0.832260	0.781616
H	-4.100453	0.130153	2.272780
C	-3.171908	-1.512069	-0.666010
H	-4.163075	-1.521824	-1.134744
H	-2.671677	-2.453412	-0.923001
H	-2.596029	-0.688055	-1.097371
C	-4.128917	-2.525055	1.439380
H	-4.241404	-2.433725	2.524111
H	-5.127183	-2.540005	0.987015
H	-3.641638	-3.483686	1.239343

Table S18. Comparison between calculated and experimental chemical shifts of diastereomers of ester **9a**

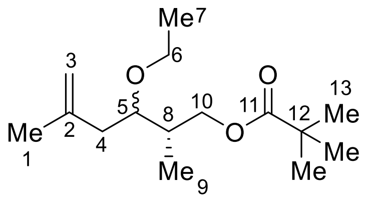

9a

	$\Delta exp_{major–minor}$	$\Delta exp_{minor–major}$	$\Delta calc_{anti–syn}$	$\Delta exp_{syn–anti}$ **8a**
C4	0.6	-0.6	-2.4	
C5	-2.2	2.2	2.7	-0.9
C8	-0.7	0.7	-0.2	
C6	0.2	-0.2	-0.6	0.2
C2	-0.4	0.4	-0.5	
C3	0.0	0.0	0.7	
C1	-0.4	0.4	0.8	
C9	-2.8	2.8	3.4	-2.2
C10	0.3	-0.3	-0.7	
C7	0.0	0.0	0.1	
C11	-0.1	0.1	-0.1	
C12	-0.1	0.1	0.0	
C13	0.0	0.0	0.0	
$MAE_{\Delta\Delta\delta}$ *syn*-major[a]	0.54			
$MAE_{\Delta\Delta\delta}$ *anti*-major	1.43			
CP3 *syn*-major[b]	0.73			
CP3 *anti*-major	–1.23			

[a]A lower value indicates a better assignment

[b]A more positive value indicates a better assignment

Substitution product ***syn*-11a**

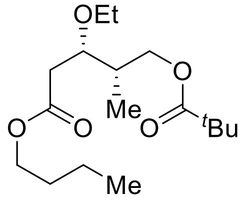

***syn*-11a**

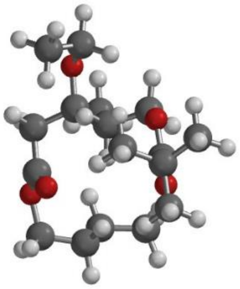

Spartan '24: ωB97X-V/6-311+G(2df,2p)

Number of imaginary vibrations: 0

Number of basis functions: 394

Charge : 0

Number of electrons: 174

SCF total energy (ωB97X-V/gas): –1043.303452 hartrees

Table S19. Cartesian Coordinates (Angstroms) for ***syn*-11a**

**Atom**	**X**	**Y**	**Z**
C	-0.136700	3.683525	1.000595
H	0.189781	4.102074	0.046205
H	-0.501499	4.484793	1.647994
O	-1.293133	2.852286	0.778913
C	-1.273241	2.081199	-0.315106
C	0.975389	2.887374	1.668216
H	1.284780	2.075585	0.998689
H	1.846888	3.546615	1.774846
C	-2.400928	1.078772	-0.326532
H	-2.922343	1.148444	-1.285278
H	-3.114531	1.274954	0.475716
O	-0.412336	2.134650	-1.167066
C	0.577905	2.322184	3.033752
H	-0.388542	1.812934	2.942680
H	0.422301	3.149138	3.739769
C	1.614819	1.345439	3.583968
H	1.308446	0.950343	4.558458
H	2.588236	1.833450	3.711622

H	1.754631	0.505843	2.894965
C	-1.797305	-0.330683	-0.209581
H	-1.095156	-0.437116	-1.043016
O	-2.822186	-1.299795	-0.317151
C	-1.008230	-0.544219	1.091951
H	-0.277567	0.267533	1.176424
C	-0.209805	-1.845588	1.025238
H	0.459774	-1.926921	1.886046
H	-0.877654	-2.709684	0.997042
C	-2.722070	-2.143806	-1.450378
H	-1.717797	-2.590821	-1.498038
H	-3.439125	-2.950428	-1.271427
C	-3.045197	-1.434959	-2.759780
H	-3.025875	-2.148497	-3.590418
H	-2.315508	-0.648133	-2.980541
H	-4.040609	-0.982492	-2.712537
C	-1.891260	-0.543622	2.339006
H	-1.294447	-0.745841	3.235205
H	-2.673429	-1.304496	2.258145
H	-2.372236	0.429067	2.479853
O	0.554327	-1.941582	-0.184956
C	1.640342	-1.153604	-0.288066
O	2.067100	-0.493555	0.632505
C	2.236354	-1.200048	-1.691711
C	2.661026	-2.643838	-2.009922
H	1.807492	-3.326087	-1.969196
H	3.421383	-2.997705	-1.304976
H	3.089127	-2.685418	-3.017449
C	3.445117	-0.262540	-1.742507
H	4.204798	-0.555772	-1.012488
H	3.891978	-0.293327	-2.742032

H	3.150369	0.767961	-1.525201
C	1.165313	-0.734345	-2.696234
H	0.783692	0.260113	-2.439920
H	1.603947	-0.685996	-3.699063
H	0.323392	-1.433357	-2.727234

Substitution product ***anti*-11a**

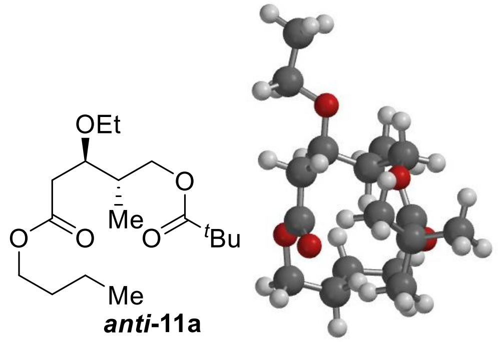

Spartan ’24: ωB97X-V/6-311+G(2df,2p)

Number of imaginary vibrations: 0

Number of basis functions: 394

Charge : 0

Number of electrons: 174

SCF total energy (ωB97X-V/gas): –1043.304261 hartrees

Table S20. Cartesian Coordinates (Angstroms) for ***anti*-11a**

**Atom**	**X**	**Y**	**Z**
C	-2.948649	0.240125	-1.703023
H	-3.649346	-0.405498	-2.238141
H	-2.980896	1.245942	-2.126221
O	-1.654428	-0.337667	-1.967033
C	-0.598237	0.477871	-1.824742
C	-3.224480	0.251539	-0.206630
H	-2.429836	0.818840	0.293869
H	-4.155107	0.806182	-0.030624
C	0.704826	-0.278170	-1.904792

H	1.502821	0.392300	-1.583518
H	0.876331	-0.537734	-2.956431
O	-0.695433	1.672027	-1.651070
C	-3.330575	-1.151820	0.397824
H	-2.522419	-1.777662	0.000234
H	-4.267762	-1.620907	0.070047
C	-3.252481	-1.133065	1.922747
H	-2.308044	-0.686633	2.253636
H	-4.066321	-0.537177	2.351787
H	-3.322105	-2.144389	2.337140
C	0.712982	-1.567398	-1.061381
H	-0.038520	-2.252214	-1.483066
O	1.986335	-2.177715	-1.111435
C	0.359565	-1.361957	0.419013
H	-0.577993	-0.802230	0.471446
C	1.411597	-0.577117	1.201140
H	2.409661	-0.969500	1.000001
H	1.196922	-0.649722	2.270947
C	2.243692	-2.946124	-2.265522
H	2.236621	-2.315373	-3.168149
H	1.457080	-3.707423	-2.391778
C	3.600948	-3.600955	-2.098695
H	3.842124	-4.210888	-2.974803
H	4.377455	-2.839938	-1.978673
H	3.606827	-4.242368	-1.212544
C	0.140098	-2.715077	1.101126
H	1.063158	-3.303823	1.107664
H	-0.625286	-3.293312	0.572429
H	-0.198376	-2.580204	2.133298
O	1.487015	0.812530	0.850214
C	0.522038	1.608578	1.341005

O	-0.419135	1.182759	1.976989
C	0.793203	3.091760	1.106381
C	1.492009	3.590988	2.388534
H	1.687344	4.665530	2.303631
H	0.863219	3.422280	3.268477
H	2.450275	3.081955	2.540543
C	1.693715	3.340684	-0.108583
H	1.853333	4.418990	-0.222249
H	1.222763	2.962555	-1.019508
H	2.669478	2.861669	0.010725
C	-0.551807	3.808395	0.933276
H	-1.205211	3.620772	1.789176
H	-0.385135	4.887960	0.848466
H	-1.055854	3.461832	0.026846

Table S21. Comparison between calculated and experimental chemical shifts of diastereomers of ester **11a**

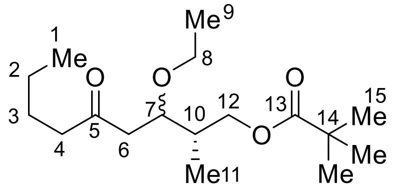

11a

	$\Delta exp_{major-minor}$	$\Delta exp_{minor-major}$	$\Delta calc_{anti-syn}$	$\Delta exp_{syn-anti}$ **8a**
C4	4.0	-4.0	-0.4	
C2	0.1	-0.1	0.9	
C5	1.9	-1.9	0.2	
C3	0.0	0.0	0.0	
C6	-2.3	2.3	-3.2	
C1	-1.8	1.8	4.6	
C7	-1.1	1.1	4.3	-0.9
C8	3.8	-3.8	-0.5	0.2
C10	-0.1	0.1	-1.0	
C12	0.4	-0.4	0.2	

C9	0.0	0.0	0.3	
C11	-0.5	0.5	-0.1	-2.2
C13	0.0	0.0	1.7	
C14	-0.1	0.1	0.7	
C15	0.1	-0.1	-0.3	
$MAE_{\Delta\Delta\delta}$ *syn*-major[a]	1.77			
$MAE_{\Delta\Delta\delta}$ *anti*-major	1.91			
CP3 *syn*-major[b]	–0.07			
CP3 *anti*-major	–0.28			

[a]A lower value indicates a better assignment

[b]A more positive value indicates a better assignment

Substitution product ***syn*-12a**

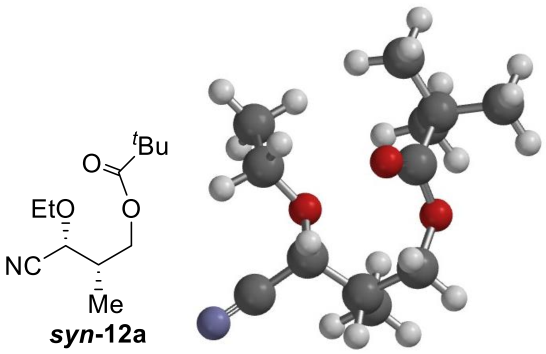

***syn*-12a**

Spartan '24: ωB97X-V/6-311+G(2df,2p)

Number of imaginary vibrations: 0

Number of basis functions: 282

Charge : 0

Number of electrons: 124

SCF total energy (ωB97X-V/gas): –750.384625 hartrees

Table S22. Cartesian Coordinates (Angstroms) for ***syn*-12a**

Atom	X	Y	Z
C	0.167380	3.628615	-0.775971
C	-0.291647	2.216878	-0.662887
H	-1.321243	2.234573	-0.276380
C	-0.283185	1.553921	-2.055232
H	-0.733864	2.284221	-2.740997

O	0.548115	1.513793	0.213187
N	0.535402	4.722689	-0.861473
C	1.139169	1.245811	-2.524026
H	1.605394	0.503665	-1.872793
H	1.124492	0.852881	-3.545615
H	1.756753	2.149290	-2.515201
C	-1.199481	0.331393	-2.124646
H	-2.207612	0.573189	-1.778192
H	-1.248188	-0.020261	-3.158229
O	-0.694750	-0.777449	-1.381474
C	0.357324	1.870456	1.578736
H	0.333504	2.965473	1.673935
H	-0.608243	1.471358	1.918070
C	1.509229	1.311567	2.387477
H	1.545536	0.220485	2.324232
H	1.394425	1.588661	3.439850
H	2.459600	1.711227	2.022704
C	-1.108639	-0.909343	-0.108660
C	-0.444572	-2.103865	0.566864
O	-1.934191	-0.185710	0.401997
C	1.065903	-2.090581	0.281463
H	1.515525	-1.136532	0.572205
H	1.554003	-2.892294	0.846365
H	1.267536	-2.245014	-0.781510
C	-1.072656	-3.383305	-0.018957
H	-2.150803	-3.416685	0.171534
H	-0.617190	-4.262288	0.449947
H	-0.907488	-3.445074	-1.098603
C	-0.714683	-2.032202	2.072082
H	-0.287984	-1.124565	2.509953
H	-1.787679	-2.027044	2.280263

H	-0.265192	-2.897935	2.569982

Substitution product ***anti*-12a**

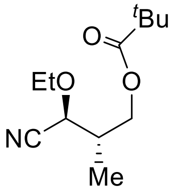

***anti*-12a**

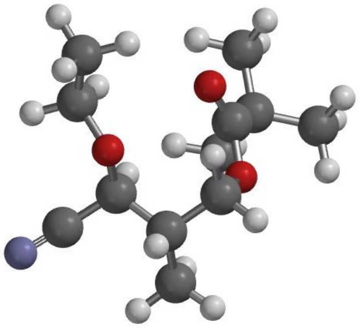

Spartan ’24: ωB97X-V/6-311+G(2df,2p)

Number of imaginary vibrations: 0

Number of basis functions: 282

Charge : 0

Number of electrons: 124

SCF total energy (ωB97X-V/gas): –750.383301 hartrees

Table S23. Cartesian Coordinates (Angstroms) for ***anti*-12a**

Atom	X	Y	Z
C	3.361717	0.513342	0.310776
C	2.039011	0.183627	-0.281026
H	1.279307	0.502475	0.452660
C	1.889098	-1.327312	-0.525426
H	2.607166	-1.591779	-1.311608
O	1.865142	0.844777	-1.496622
N	4.392046	0.789309	0.760031
C	2.183233	-2.146079	0.731816
H	1.513837	-1.863115	1.550352
H	2.034416	-3.211755	0.532081
H	3.214414	-2.004317	1.066969
C	0.499269	-1.618124	-1.092107
H	0.429853	-2.672010	-1.373013
H	0.285693	-0.984790	-1.953431

O	-0.505578	-1.420272	-0.088871
C	1.524492	2.219257	-1.378664
H	2.384272	2.782756	-0.987692
H	0.688199	2.324589	-0.673463
C	1.117523	2.710484	-2.750990
H	0.246275	2.148831	-3.097861
H	0.858139	3.772743	-2.709337
H	1.934552	2.579160	-3.466330
C	-1.271807	-0.313275	-0.157525
C	-2.228359	-0.235667	1.029939
O	-1.191991	0.503948	-1.044587
C	-3.135349	-1.478622	1.026042
H	-3.827181	-1.430367	1.873939
H	-2.549562	-2.397827	1.109288
H	-3.730562	-1.528217	0.107733
C	-3.070054	1.036575	0.906955
H	-2.439702	1.930644	0.905397
H	-3.763078	1.102483	1.752455
H	-3.650790	1.037927	-0.019604
C	-1.396699	-0.200014	2.324900
H	-0.718166	0.661178	2.336193
H	-2.064365	-0.109160	3.188354
H	-0.804409	-1.111405	2.442276

Table S24. Comparison between calculated and experimental chemical shifts of diastereomers of ester **12a**

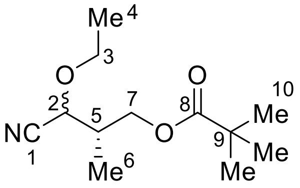

12a

	$\Delta exp_{major–minor}$	$\Delta exp_{minor–major}$	$\Delta calc_{anti–syn}$	$\Delta exp_{syn–anti}$ **8a**
C1	0.4	-0.4	-0.6	

C2	-1.1	1.1	0.3	-0.9
C5	0.1	-0.1	-0.8	
C3	0.1	-0.1	0.8	0.2
C6	-0.8	0.8	1.3	2.2
C7	0.0	0.0	-0.8	
C4	0.0	0.0	-0.1	
C8	0.0	0.0	-0.7	
C9	-0.1	0.1	0.0	
C10	0.0	0.0	0.0	
$MAE_{\Delta\Delta\delta}$ *syn*-major[a]	0.48			
$MAE_{\Delta\Delta\delta}$ *anti*-major	0.78			
CP3 *syn*-major[b]	0.37			
CP3 *anti*-major	–0.83			

[a]A lower value indicates a better assignment

[b]A more positive value indicates a better assignment

Substitution product ***anti*-8b**

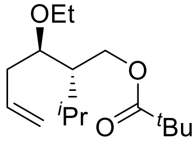

***anti*-8b**

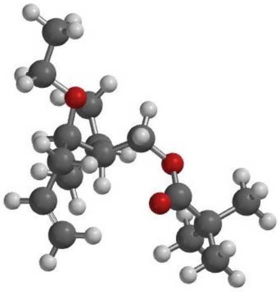

Spartan '24: ωB97X-V/6-311+G(2df,2p)

Number of imaginary vibrations: 0

Number of basis functions: 345

Charge : 0

Number of electrons: 150

SCF total energy (ωB97X-V/gas): –853.485917 hartrees

Table S25. Cartesian Coordinates (Angstroms) for ***anti*-8b**

Atom	X	Y	Z
C	0.777557	0.875820	-1.633626
H	1.317428	0.309891	-2.411131

C	0.567692	-0.023440	-0.408788
H	-0.356964	-0.581993	-0.595899
C	0.312849	0.826048	0.839842
H	-0.292509	1.706438	0.620627
H	1.241678	1.147626	1.311053
C	1.684474	-1.064820	-0.175793
H	1.517345	-1.467793	0.832781
C	3.097759	-0.474170	-0.214736
H	3.204459	0.386710	0.451774
H	3.832747	-1.229385	0.084554
H	3.360091	-0.140272	-1.225613
C	1.559340	-2.233125	-1.158140
H	2.321857	-2.994649	-0.960629
H	0.575232	-2.708957	-1.083975
H	1.689321	-1.899892	-2.195239
O	1.577574	1.973517	-1.226442
C	2.351529	2.557467	-2.247163
H	1.710568	2.958701	-3.047608
H	3.004494	1.795113	-2.704002
C	3.181845	3.666256	-1.631111
H	3.830337	3.262399	-0.848022
H	2.532244	4.424397	-1.184332
H	3.806810	4.145163	-2.391259
C	-0.564796	1.349849	-2.226641
H	-0.367172	2.178200	-2.918608
H	-1.198913	1.737732	-1.422528
C	-1.276857	0.244833	-2.954224
H	-0.827371	-0.078484	-3.894363
C	-2.373262	-0.366238	-2.510933
H	-2.839226	-1.172178	-3.071331
H	-2.836837	-0.077933	-1.570118

O	-0.353253	0.033397	1.836675
C	-1.681751	-0.099567	1.700227
C	-2.247020	-1.118968	2.684989
O	-2.330088	0.500871	0.871434
C	-3.772781	-1.131396	2.569201
H	-4.190209	-1.867990	3.264406
H	-4.194676	-0.150899	2.808849
H	-4.089554	-1.389916	1.555126
C	-1.674228	-2.497203	2.301035
H	-2.086707	-3.265382	2.964305
H	-0.584198	-2.511240	2.390539
H	-1.940988	-2.760800	1.271098
C	-1.819296	-0.757908	4.116434
H	-0.730788	-0.742001	4.212985
H	-2.219785	-1.498736	4.817325
H	-2.206002	0.224909	4.406997

Substitution product ***syn*-8b**

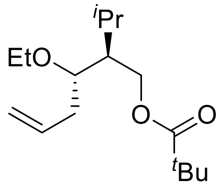

***syn*-8b**

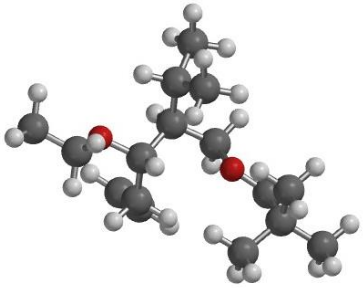

Spartan ’24: ωB97X-V/6-311+G(2df,2p)

Number of imaginary vibrations: 0

Number of basis functions: 345

Charge : 0

Number of electrons: 150

SCF total energy (ωB97X-V/gas): –853.484466 hartrees

Table S26. Cartesian Coordinates (Angstroms) for ***syn*-8b**

Atom	X	Y	Z
C	-0.636332	1.070270	-0.740458
H	0.357758	1.229387	-0.297789
C	-0.705834	-0.359547	-1.304831
H	-1.672689	-0.435683	-1.821913
C	-0.755888	-1.401343	-0.192812
H	-0.595496	-2.413769	-0.575362
H	-1.723762	-1.395568	0.314849
C	0.390128	-0.613623	-2.366991
H	0.382208	0.273061	-3.010720
C	0.044422	-1.819126	-3.246474
H	-0.943005	-1.705415	-3.707716
H	0.043338	-2.755066	-2.674344
H	0.779920	-1.933460	-4.050347
C	1.802142	-0.764811	-1.789539
H	1.894053	-1.673545	-1.184570
H	2.082693	0.082657	-1.155093
H	2.534372	-0.829476	-2.601987
O	-0.809616	1.952234	-1.838232
C	-0.090794	3.160253	-1.740207
H	-0.354094	3.700579	-0.816758
H	0.990461	2.950014	-1.697672
C	-0.422970	4.006003	-2.953875
H	-0.159519	3.472046	-3.871608
H	-1.493548	4.228766	-2.980474
H	0.130385	4.949901	-2.926815
C	-1.688998	1.374768	0.341139
H	-1.567129	2.428528	0.627654
H	-1.481505	0.784711	1.240493
C	-3.096969	1.151409	-0.128905
H	-3.368883	1.665136	-1.049937

C	-3.992066	0.385340	0.489196
H	-5.000426	0.261412	0.105090
H	-3.751271	-0.144047	1.409510
O	0.268901	-1.098671	0.765138
C	0.280811	-1.833540	1.889178
C	1.367621	-1.350470	2.847739
O	-0.490663	-2.741523	2.091045
C	1.359857	-2.235238	4.095833
H	2.132389	-1.895306	4.794159
H	1.558313	-3.279742	3.839350
H	0.391121	-2.196339	4.601587
C	2.732632	-1.432190	2.142626
H	2.962107	-2.461627	1.846041
H	2.756777	-0.803764	1.247989
H	3.520081	-1.094768	2.825608
C	1.063709	0.110577	3.226323
H	1.816265	0.473474	3.935031
H	0.081395	0.197970	3.704380
H	1.077598	0.759162	2.345472

Table S27. Comparison between calculated and experimental chemical shifts of diastereomers of ester **8b**

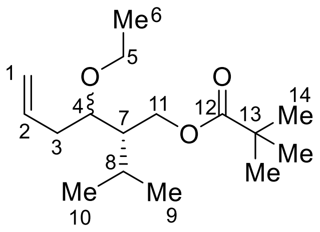

8b

	$\Delta exp_{major-minor}$	$\Delta exp_{minor-major}$	$\Delta calc_{anti-syn}$
C4	-0.4	0.4	-0.1
C7	0.0	0.0	0.6
C11	0.1	-0.1	0.6
C8	1.6	-1.6	0.7

C10 (or C9)	-0.6	0.6	-2.4
C9 (or C10)	0.2	-0.2	0.7
C5	0.7	-0.7	-0.7
C6	0.0	0.0	-0.1
C3	0.4	-0.4	-1.8
C2	-0.6	0.6	-1.3
C1	0.6	-0.6	3.1
C12	0.1	-0.1	1.1
C13	-0.1	0.1	0.2
C14	0.1	-0.1	0.0
$MAE_{\Delta\Delta\delta}$ *syn*-major[a]	1.18		
$MAE_{\Delta\Delta\delta}$ *anti*-major	0.90		
CP3 *syn*-major[b]	–1.11		
CP3 *anti*-major	0.06		

[a]A lower value indicates a better assignment

[b]A more positive value indicates a better assignment

Substitution product ***anti*-40**

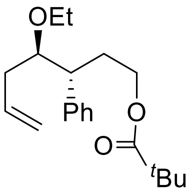

***anti*-40**

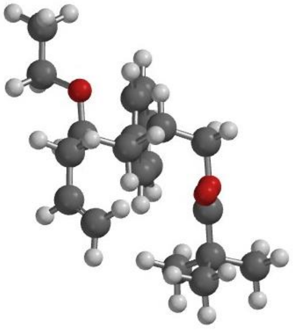

Spartan ’24: ωB97X-V/6-311+G(2df,2p)

Number of imaginary vibrations: 0

Number of basis functions: 405

Charge : 0

Number of electrons: 174

SCF total energy (ωB97X-V/gas): –1005.913405 hartrees

Table S28. Cartesian Coordinates (Angstroms) for ***anti*-40**

**Atom**	**X**	**Y**	**Z**

C	-1.770035	1.163204	-0.016223
H	-2.253584	0.974577	-0.987879
C	-0.308953	0.699034	-0.142276
C	0.519874	1.055366	1.101111
H	0.041097	0.649487	1.995520
O	-1.789678	2.553978	0.255927
C	-2.563904	0.425879	1.078094
H	-2.102252	0.619025	2.051534
H	-3.565241	0.875392	1.111278
C	-2.783051	3.271896	-0.441745
H	-2.612430	3.184599	-1.526320
H	-3.781477	2.857282	-0.228991
C	-2.713234	4.722391	-0.007088
H	-3.471344	5.314427	-0.529121
H	-1.728191	5.141385	-0.234322
H	-2.882709	4.806943	1.070170
C	-2.686753	-1.051443	0.841705
H	-3.287959	-1.352467	-0.017508
C	-2.111934	-1.989509	1.591861
H	-1.487602	-1.736671	2.446406
H	-2.241365	-3.046575	1.374570
C	0.321330	1.204839	-1.426667
C	1.493410	2.093709	-3.820290
C	0.554457	2.567348	-1.646227
C	0.689404	0.300397	-2.424560
C	1.269926	0.736420	-3.612012
C	1.131923	3.007092	-2.832938
H	1.947403	2.438381	-4.744735
H	0.268148	3.283353	-0.882926
H	0.518359	-0.761449	-2.266669
H	1.550989	0.013784	-4.372745

H	1.302474	4.068971	-2.986907
C	1.946817	0.528106	1.037883
H	2.518759	1.027114	0.253564
H	2.447391	0.664202	2.000533
O	2.000038	-0.859600	0.672006
C	1.614383	-1.757487	1.591472
O	1.271438	-1.458970	2.713481
C	1.693258	-3.180440	1.041142
C	0.919903	-3.260574	-0.285798
H	0.929347	-4.292163	-0.655185
H	-0.124328	-2.955607	-0.153411
H	1.374748	-2.618232	-1.044415
C	3.175766	-3.519503	0.798629
H	3.748650	-3.470073	1.731099
H	3.260124	-4.537792	0.403254
H	3.625274	-2.830478	0.078081
C	1.092850	-4.145016	2.066115
H	0.035898	-3.925005	2.243579
H	1.174437	-5.173179	1.696941
H	1.611889	-4.075252	3.025647
H	-0.347239	-0.394082	-0.209464
H	0.563502	2.142988	1.224819

Substitution product ***syn*-40**

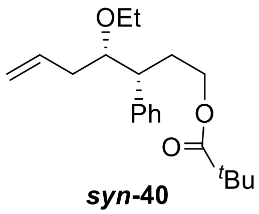

***syn*-40**

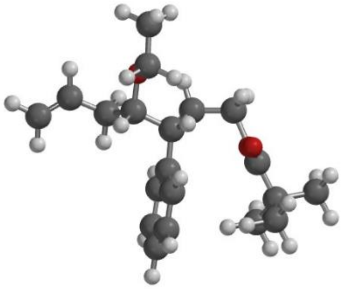

Spartan ’24: ωB97X-V/6-311+G(2df,2p)

Number of imaginary vibrations: 0

Number of basis functions: 405

Charge : 0

Number of electrons: 174

SCF total energy (ωB97X-V/gas): –1005.912006 hartrees

Table S29. Cartesian Coordinates (Angstroms) for ***syn*-40**

**Atom**	**X**	**Y**	**Z**
C	-0.343900	2.125128	1.007253
H	-0.259426	2.733612	0.092244
C	-0.306450	0.631795	0.583245
C	-0.462900	-0.302908	1.793266
O	-1.567561	2.421910	1.656432
C	0.771108	2.546951	1.966753
H	0.660282	1.976977	2.899406
H	1.740852	2.287385	1.531765
C	-2.659613	2.668468	0.796094
H	-2.887990	1.784480	0.181760
H	-2.411761	3.492331	0.108369
C	-3.857573	3.030915	1.652175
H	-4.113958	2.203503	2.320494
H	-3.635237	3.911056	2.262571
H	-4.724803	3.251078	1.021931
C	0.725125	4.017603	2.267975
H	-0.204350	4.379883	2.702928
C	1.718086	4.867600	2.022551
H	2.656289	4.536839	1.581391
H	1.633904	5.925664	2.253744
C	0.891890	0.360398	-0.309876
C	3.094328	0.058738	-2.037281
C	2.165291	0.074576	0.191226
C	0.743402	0.473791	-1.696145
C	1.829901	0.327139	-2.553293

C	3.255916	-0.071509	-0.660475
H	3.945005	-0.056432	-2.702174
H	2.318001	-0.032578	1.261044
H	-0.243908	0.667560	-2.107510
H	1.685917	0.419905	-3.625844
H	4.235250	-0.290971	-0.245340
C	-1.180472	-1.606429	1.477534
H	-1.270576	-2.218213	2.378803
H	-2.176501	-1.412356	1.071838
O	-0.451629	-2.420166	0.548240
C	-0.832536	-2.394172	-0.741131
O	-1.736649	-1.704213	-1.157824
C	-0.034465	-3.384379	-1.585361
C	1.456234	-3.343189	-1.223064
H	1.879407	-2.351737	-1.406302
H	1.620365	-3.597412	-0.173223
H	1.999033	-4.065806	-1.843107
C	-0.230036	-3.039241	-3.064135
H	0.293785	-3.773006	-3.686509
H	0.175277	-2.047291	-3.286611
H	-1.289020	-3.040994	-3.334328
C	-0.607882	-4.784595	-1.289473
H	-1.673639	-4.834649	-1.537815
H	-0.081839	-5.531691	-1.893930
H	-0.482949	-5.046614	-0.234287
H	-1.075010	0.213506	2.542292
H	-1.192703	0.484337	-0.043543
H	0.500688	-0.522577	2.265254

Table S30. Comparison between calculated and experimental chemical shifts of diastereomers of ester **40**

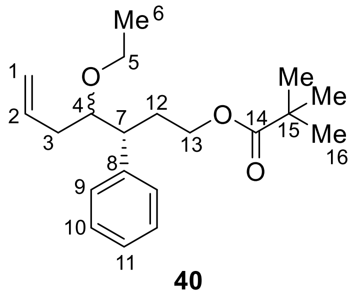

40

	$\Delta exp_{major–minor}$	$\Delta exp_{minor–major}$	$\Delta calc_{anti–syn}$
C4	0.1	-0.1	0.0
C7	0.8	-0.8	-1.7
C12	-0.3	0.3	-3.3
C8	1.2	-1.2	1.7
C5	-0.4	0.4	0.3
C3	0.1	-0.1	0.6
C6	0.0	0.0	-0.2
C2	-0.5	0.5	-1.1
C1	0.2	-0.2	2.2
C11	0.2	-0.2	0.0
C10	0.3	-0.3	-1.2
C9	-0.8	0.8	-1.8
C13	0.3	-0.3	-0.1
C14	0.0	0.0	0.0
C15	-0.1	0.1	0.1
C16	0.0	0.0	0.2
$MAE_{\Delta\Delta\delta}$ *syn*-major[a]	1.04		
$MAE_{\Delta\Delta\delta}$ *anti*-major	0.85		
CP3 *syn*-major[b]	–1.43		
CP3 *anti*-major	–0.13		

[a]A lower value indicates a better assignment

[b]A more positive value indicates a better assignment

Substitution product ***syn*-S33**

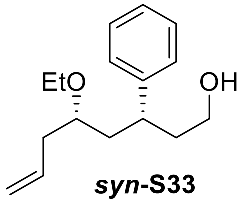

***syn*-S33**

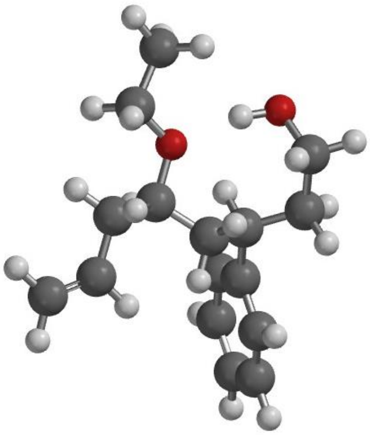

Spartan '24: ωB97X-V/6-311+G(2df,2p)

Number of imaginary vibrations: 0

Number of basis functions: 318

Charge : 0

Number of electrons: 136

SCF total energy (ωB97X-V/gas): –774.604578 hartrees

Table S31. Cartesian Coordinates (Angstroms) for ***syn*-S33**

Atom	X	Y	Z
C	-0.786840	-1.489001	-1.086733
H	0.262591	-1.654482	-0.815732
H	-0.937210	-1.936153	-2.076314
C	-1.028958	0.028121	-1.185496
H	-2.058134	0.189580	-1.541483
C	-0.857077	0.774350	0.135031
H	-1.118870	1.821494	-0.049532
H	-1.599855	0.394180	0.843623
O	-0.125204	0.616994	-2.116210
C	-0.515959	0.568475	-3.474472
H	-1.487671	1.071562	-3.595814
H	-0.638551	-0.473098	-3.804929
C	0.554414	1.262824	-4.294203
H	1.508441	0.734662	-4.206547

H	0.699487	2.288052	-3.941174
H	0.267733	1.290774	-5.349753
C	-1.689307	-2.157042	-0.087121
H	-1.431406	-2.018219	0.962495
C	-2.766344	-2.869374	-0.406984
H	-3.398164	-3.320298	0.352515
H	-3.052644	-3.036470	-1.443730
C	0.570547	0.665362	0.730826
H	1.223138	0.209433	-0.021589
C	1.194996	2.042092	1.025599
H	2.179188	1.879386	1.479871
H	0.586876	2.588575	1.760206
C	0.614381	-0.234510	1.950419
C	0.688027	-1.915689	4.202099
C	-0.171107	0.036657	3.075071
C	1.441660	-1.357940	1.979376
C	1.478816	-2.194564	3.092852
C	-0.137043	-0.793183	4.190377
H	0.713129	-2.567160	5.070462
H	-0.821662	0.908545	3.077164
H	2.063316	-1.583263	1.115697
H	2.126394	-3.066631	3.090185
H	-0.756088	-0.563564	5.052970
C	1.368017	2.910981	-0.226607
H	0.389320	3.270994	-0.581467
H	1.953715	3.798366	0.035380
O	2.061329	2.253602	-1.261978

Substitution product ***anti*-S33**

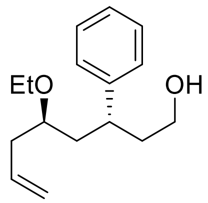

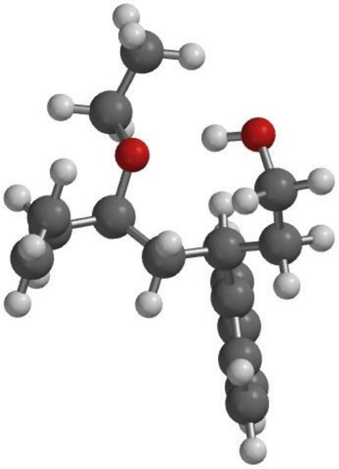

Spartan '24: ωB97X-V/6-311+G(2df,2p)

Number of imaginary vibrations: 0

Number of basis functions: 318

Charge : 0

Number of electrons: 136

SCF total energy (ωB97X-V/gas): –774.604681 hartrees

Table S32. Cartesian Coordinates (Angstroms) for ***anti*-S33**

**Atom**	**X**	**Y**	**Z**
C	-1.643238	-0.342495	-2.246999
H	-1.240671	-0.541852	-3.249626
H	-2.217688	-1.230277	-1.955815
C	-0.465684	-0.174367	-1.275706
H	0.146797	-1.090659	-1.298259
C	-0.894415	0.092717	0.161771
H	-1.634401	-0.657313	0.461932
H	-1.412702	1.057289	0.181789
O	0.351714	0.929658	-1.661897
C	1.333418	0.652659	-2.643185
H	1.921587	-0.226336	-2.336624
H	0.856997	0.414586	-3.605498
C	2.225315	1.871889	-2.775994
H	2.995644	1.698688	-3.533703

H	1.636756	2.745187	-3.072405
H	2.714634	2.094833	-1.823167
C	-2.533133	0.866320	-2.292809
H	-2.037962	1.803126	-2.544436
C	-3.838083	0.858794	-2.036238
H	-4.433797	1.765829	-2.082282
H	-4.362631	-0.057996	-1.774634
C	0.321342	0.080791	1.117970
H	1.212154	0.269401	0.508762
C	0.295094	1.221897	2.148802
H	1.191264	1.132327	2.773551
H	-0.572523	1.128943	2.816441
C	0.511525	-1.276103	1.765171
C	0.875184	-3.800482	2.945487
C	-0.427303	-1.788901	2.666615
C	1.632759	-2.051936	1.467599
C	1.816012	-3.304260	2.049930
C	-0.249126	-3.036599	3.253063
H	1.014996	-4.775584	3.402645
H	-1.310096	-1.202704	2.912499
H	2.374664	-1.666832	0.771483
H	2.696373	-3.890776	1.802816
H	-0.989039	-3.415117	3.952651
C	0.268122	2.613319	1.497350
H	-0.745954	2.843049	1.134730
H	0.506367	3.367776	2.253744
O	1.214296	2.765883	0.462890
H	0.895433	2.255626	-0.300414
H	1.432579	1.625582	-1.654352

Table S33. Comparison between calculated and experimental chemical shifts of diastereomers of alcohol **S33**

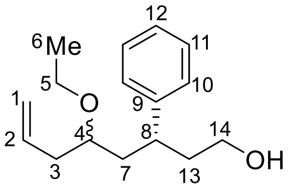

S33

	$\Delta exp_{major–minor}$	$\Delta exp_{minor–major}$	$\Delta calc_{anti–syn}$
C3	-0.6	0.6	1.5
C4	0.4	-0.4	-1.0
C7	-0.7	0.7	1.9
C5	-0.3	0.3	0.7
C6	-0.2	0.2	0.3
C2	0.2	-0.2	-0.1
C1	-0.1	0.1	0.5
C8	-0.1	0.1	1.2
C13	-0.9	0.9	0.9
C9	0.2	-0.2	-1.6
C12	0.0	0.0	0.1
C11	-0.2	0.2	0.4
C10	0.0	0.0	-0.2
C14	-0.2	0.2	0.1
$MAE_{\Delta\Delta\delta}$ *syn*-major[a]	0.49		
$MAE_{\Delta\Delta\delta}$ *anti*-major	1.04		
CP3 *syn*-major[b]	0.63		
CP3 *anti*-major	–2.03		

[a]A lower value indicates a better assignment

[b]A more positive value indicates a better assignment

X-ray crystallography

A colorless rod-like crystal of sulfonate ***anti*-S32** was obtained by slow evaporation in Et_2O. For other compounds such as ***anti*-S27** and ***anti*-44**, our original goal was to incorporate these molecules into a guanidinium sulfonate (GS) framework host crystal and determine its molecular structure by solving the crystal structure of the whole inclusion compound.[23] 300 μL of 0.01 M GS

host solution were mixed with 300 μL of methanol-ethanol mixtures (methanol:ethanol ratios 0:1, 2:1, 1:2, 1:0) and guest molecules in equimolar amounts with GS hosts. The following GS hosts were used: guanidinium cyclohexanemonosulfonate, guanidinium cyclohexylbenzenemonosulfonate, guanidinium biphenyldisulfonate, guanidinium 1,5-naphthalenedisulfonate, guanidinium 2,6-naphthalenedisulfonate and guanidinium 4,5-tetra(4-sulfonatophenyl)benzene. The solutions were set up for slow evaporation at room temperature for one-two weeks. All solutions with ***anti*-S27** produced plate-like crystals. X-ray diffraction experiment demonstrated that all crystals sufficiently large for determination of their crystal structure (all hosts except guanidinium biphenyldisulfonate and guanidinium 1,5-naphthalenedisulfonate) correspond to the crystals of ***anti*-S27**. For the structure determination a crystal from a guanidinium cyclohexylbenzenemonosulfonate solution was selected. All solutions containing ***anti*-44** formed fine fibers not suitable for single crystal X-ray diffraction analysis, however, solutions with guanidinium biphenyldisulfonate also produced large plates. They were found to be the GS inclusion compounds and were used to determine molecule structure of ***anti*-44**.

The crystals were mounted on MiTeGen MicroMount loops with Type B immersion oil (Cargille Labs) and X-ray diffraction data were collected on a Bruker SMART APEX II diffractometer equipped with a PHOTON-II-C14 detector. The X-ray beam generated from an INCOATEC micro-focused source was monochromated and collimated by a Montel multilayer optics (Mo*Kα* radiation, λ = 0.71073 Å). Full data sets were collected with omega and phi scan methods. The data sets were processed with the INTEGRATE program of the APEX4 software for reduction and cell refinement. Multi-scan absorption corrections were applied by the SCALE program for the area detector. The structures were solved by intrinsic phasing methods (SHELXT) and the structure models were completed and refined using the full-matrix least-square methods on F^2 (SHELXL). Non-hydrogen atoms in the structures were refined with anisotropic displacement parameters, and hydrogen atoms on carbons were placed in idealized positions (C-H = 0.95-1.00 Å) and included as riding with U_{iso}(H) = 1.2 or 1.5U_{eq}(non-H). The structure of ***anti*-S32** was refined as two component inversion twin. Graphical representation of crystallographic data was done using Mercury (Version 4.3.1). Crystallographic data of ***anti*-S32**, **anti-S27**, and **anti-44**, including cif, res, fcf, and hkl files, have been deposited with the Cambridge Crystallographic Data Centre with numbers 2388053, 2388054, and 2388055, respectively. These data can be obtained free of charge via www.ccdc.cam.ac.uk/data_request/cif, or by emailing data_request@ccdc.cam.ac.uk, or by contacting The Cambridge Crystallographic Data Centre, 12 Union Road, Cambridge CB2 1EZ, UK; fax: +44 1223 336033.

Figure S1: Molecular structure of **anti-S32**. The hydrogen atoms are omitted for clarity. The thermal ellipsoids are shown at 50% probability.

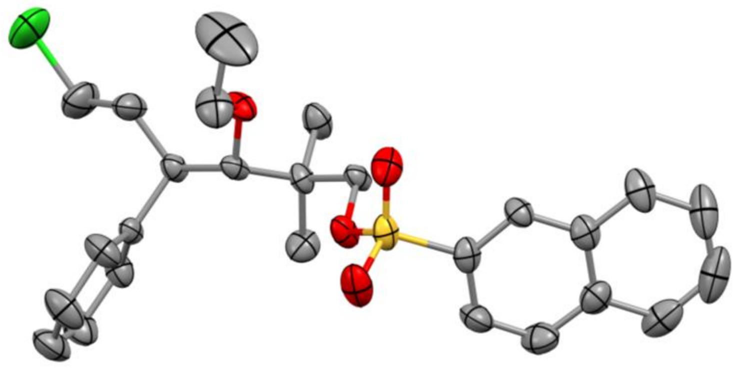

Figure S2: Molecular structure of ***anti*-S27**. The hydrogen atoms are omitted for clarity. The thermal ellipsoids are shown at 50% probability.

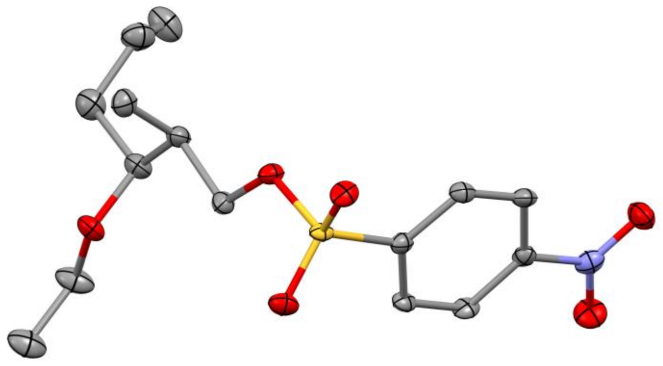

Figure S3: Molecular structure of ***anti*-44**. GS framework host molecules and hydrogen atoms are omitted for clarity. The thermal ellipsoids are shown at 50% probability.

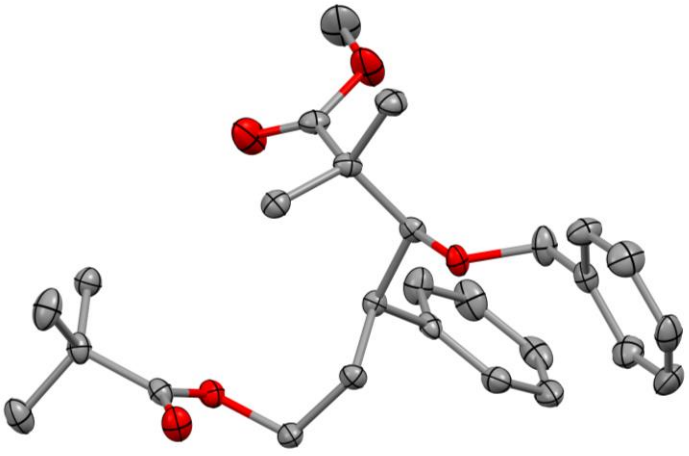

Table S34. Crystallographic data.

Compound	***anti*-S32**	***anti*-S27**	***anti*-44**
X-ray lab code	21kaw1h	24ags2s	24ags3s
CCDC numbers	2388053	2388054	2388055
Chemical formula	$C_{26}H_{31}ClO_4S$	$C_{15}H_{21}NO_6S$	$C_{41}H_{56}N_6O_{11}S_2$
Formula weight, g/mol	475.02	343.39	873.03
Temperature, K	200(2)	100(2)	100(2)
Crystal size, mm	0.12 x 0.19 x 0.38	0.01 x 0.19 x 0.26	0.05 x 0.32 x 0.36
Crystal habit	colorless rod	colorless plate	colorless plate
Crystal system	monoclinic	triclinic	monoclinic
Space group	*Pc*	*P*-1	*Cc*
a, Å	8.9237(10)	7.2005(15)	12.2384(5)
b, Å	26.600(3)	7.5723(16)	25.7202(12)
c, Å	11.5040(13)	16.222(4)	14.2122(6)
α, °	90	94.327(14)	90
β, °	110.660(4)	100.460(14)	93.543(3)
γ, °	90	107.846(13)	90
Volume, $Å^3$	2555.1(5)	819.8(3)	4465.1(3)
Z	4	2	4
Density (calc.), g/cm^3	1.235	1.391	1.299
Absorption coefficient, mm^{-1}	0.260	0.227	0.183
F(000)	1008	364	1856
Reflections collected	31473	11501	36413
Unique reflections	10368	3904	10388
Number of parameters	584	211	547
Number of restrains	2	0	2
R_{int}	0.0545	0.0968	0.0550
R_1 $[I > 2\sigma(I)]$	0.0738	0.0996	0.0432
wR_2 (all data)	0.1940	0.2414	0.0934

Weighting scheme[a], k_1, k_2	0.0671, 4.2742	0.0610, 5.1743	0.0412, 0
GOF (all data)	1.069	1.067	1.045
Largest diff. peak and hole, e/Å^3	0.628, -0.363	0.628, -0.363	0.298, -0.288

[a] $w = 1/[\sigma^2(F_o^2) + (k_1P)^2 + k_2P]$ with $P = (F_o^2 + 2F_c^2)/3$

References

(1) Babij, N. R.; McCusker, E. O.; Whiteker, G. T.; Canturk, B.; Choy, N.; Creemer, L. C.; Amicis, C. V. D.; Hewlett, N. M.; Johnson, P. L.; Knobelsdorf, J. A.; Li, F.; Lorsbach, B. A.; Nugent, B. M.; Ryan, S. J.; Smith, M. R.; Yang, Q. NMR Chemical Shifts of Trace Impurities: Industrially Preferred Solvents Used in Process and Green Chemistry. *Org. Process Res. Dev.* **2016**, *20*, 661–667.

(2) Otte, D. A. L.; Borchmann, D. E.; Lin, C.; Weck, M.; Woerpel, K. A. 13C NMR Spectroscopy for the Quantitative Determination of Compound Ratios and Polymer End Groups. *Org. Lett.* **2014**, *16*, 1566–1569.

(3) Arcadi, A.; Bernocchi, E.; Cacchi, S.; Marinelli, F. β-Vinyl-γ-butyrolactones via the palladium-catalysed reaction of vinyl triflates with Z-2-buten-1,4-diol. *Tetrahedron* **1991**, *47*, 1525–1540.

(4) Ramdular, A.; Woerpel, K. A. Diastereoselective Substitution Reactions of Acyclic β-Alkoxy Acetals via Electrostatically Stabilized Oxocarbenium Ion Intermediates. *Org. Lett.* **2022**, *24*, 3217–3222.

(5) Barrow, R. A.; Hemscheidt, T.; Liang, J.; Paik, S.; Moore, R. E.; Tius, M. A. Total Synthesis of Cryptophycins. Revision of the Structures of Cryptophycins A and C. *J. Am. Chem. Soc.* **1995**, *117*, 2479–2490.

(6) Fujita, S.; Abe, M.; Shibuya, M.; Yamamoto, Y. Intramolecular Hydroalkoxylation of Unactivated Alkenes Using Silane–Iodine Catalytic System. *Org. Lett.* **2015**, *17*, 3822–3825.

(7) Boal, B. W.; Schammel, A. W.; Garg, N. K. An Interrupted Fischer Indolization Approach toward Fused Indoline-Containing Natural Products. *Org. Lett.* **2009**, *11*, 3458–3461.

(8) Liang, S.; Kumon, T.; Angnes, R. A.; Sanchez, M.; Xu, B.; Hammond, G. B. Synthesis of Alkyl Halides from Aldehydes via Deformylative Halogenation. *Org. Lett.* **2019**, *21*, 3848–3854.

(9) Wermuth, C. G.; Bourguignon, J. J.; Schlewer, G.; Gies, J. P.; Schoenfelder, A.; Melikian, A.; Bouchet, M. J.; Chantreux, D.; Molimard, J. C. Synthesis and structure-activity relationships of a series of aminopyridazine derivatives of γ-aminobutyric acid acting as selective GABA-A antagonists. *J. Med. Chem.* **1987**, *30*, 239–249.

(10) Matsumoto, A.; Shiozaki, Y.; Sakurai, S.; Maruoka, K. Synthesis of Functionalized Aliphatic Acid Esters via the Generation of Alkyl Radicals from Silylperoxyacetals. *Chem. Asian. J.* **2021**, *16*, 2431–2434.

(11) Escher, S.; Niclass, Y. Structure and Synthesis of Novel C12 Terpenoids from Quince Fruit (Cydonia oblonga MILL.). *Helv. Chim. Acta* **1991**, *74*, 179–188.

(12) Kamat, V. P.; Hagiwara, H.; Katsumi, T.; Hoshi, T.; Suzuki, T.; Ando, M. Ring Closing Metathesis Directed Synthesis of (R)-(−)-Muscone from (+)-Citronellal. *Tetrahedron* **2000**, *56*, 4397–4403.

(13) Mori, K.; Akasaka, K. Pheromone synthesis. Part 256: Synthesis of the four stereoisomers of 5,11-dimethylpentacosane, a new sex pheromone component of the male Galleria mellonella (L.), with high stereochemical purities as determined by the derivatization-HPLC analysis of the eight stereoisomers of 5,11-dimethyl-8-pentacosanol. *Tetrahedron* **2015**, *71*, 4102–4115.

(14) Ren, J.; Wang, J.; Tong, R. Asymmetric Total Synthesis of (+)-Attenol B. *Org. Lett.* **2015**, *17*, 744–747.

(15) Witte, J.; Goldey, M.; Neaton, J. B.; Head-Gordon, M. Beyond Energies: Geometries of Nonbonded Molecular Complexes as Metrics for Assessing Electronic Structure Approaches. *J. Chem. Theory Comput.* **2015**, *11*, 1481–1492.
(16) Demkiw, K. M.; Remmerswaal, W. A.; Hansen, T.; van der Marel, G. A.; Codée, J. D. C.; Woerpel, K. A. Halogen Atom Participation in Guiding the Stereochemical Outcomes of Acetal Substitution Reactions. *Angew. Chem. Int. Ed.* **2022**, *61*, e202209401.
(17) Chai, J.-D.; Head-Gordon, M. Long-range corrected hybrid density functionals with damped atom–atom dispersion corrections. *Phys. Chem. Chem. Phys.* **2008**, *10*, 6615–6620.
(18) Csonka, G. I.; French, A. D.; Johnson, G. P.; Stortz, C. A. Evaluation of Density Functionals and Basis Sets for Carbohydrates. *J. Chem. Theory Comput.* **2009**, *5*, 679–692.
(19) Jabłoński, M.; Palusiak, M. Basis Set and Method Dependence in Quantum Theory of Atoms in Molecules Calculations for Covalent Bonds. *J. Phys. Chem. A* **2010**, *114*, 12498–12505.
(20) Hehre, W.; Klunzinger, P.; Deppmeier, B.; Driessen, A.; Uchida, N.; Hashimoto, M.; Fukushi, E.; Takata, Y. Efficient Protocol for Accurately Calculating 13C Chemical Shifts of Conformationally Flexible Natural Products: Scope, Assessment, and Limitations. *J. Nat. Prod.* **2019**, *82*, 2299–2306.
(21) Smith, S. G.; Goodman, J. M. Assigning the Stereochemistry of Pairs of Diastereoisomers Using GIAO NMR Shift Calculation. *J. Org. Chem.* **2009**, *74*, 4597–4607.
(22) Lauro, G.; Das, P.; Riccio, R.; Reddy, D. S.; Bifulco, G. DFT/NMR Approach for the Configuration Assignment of Groups of Stereoisomers by the Combination and Comparison of Experimental and Predicted Sets of Data. *J. Org. Chem.* **2020**, *85*, 3297–3306.
(23) Yusov, A.; Dillon, A. M.; Chaudhry, M. T.; Newman, J. A.; Lee, A. Y.; Ward, M. D. Benchmarking Guanidinium Organosulfonate Hydrogen-Bonded Frameworks for Structure Determination of Encapsulated Guests. *ACS Mater. Lett.* **2024**, *6*, 1906–1912.

^{1}H and ^{13}C NMR spectra

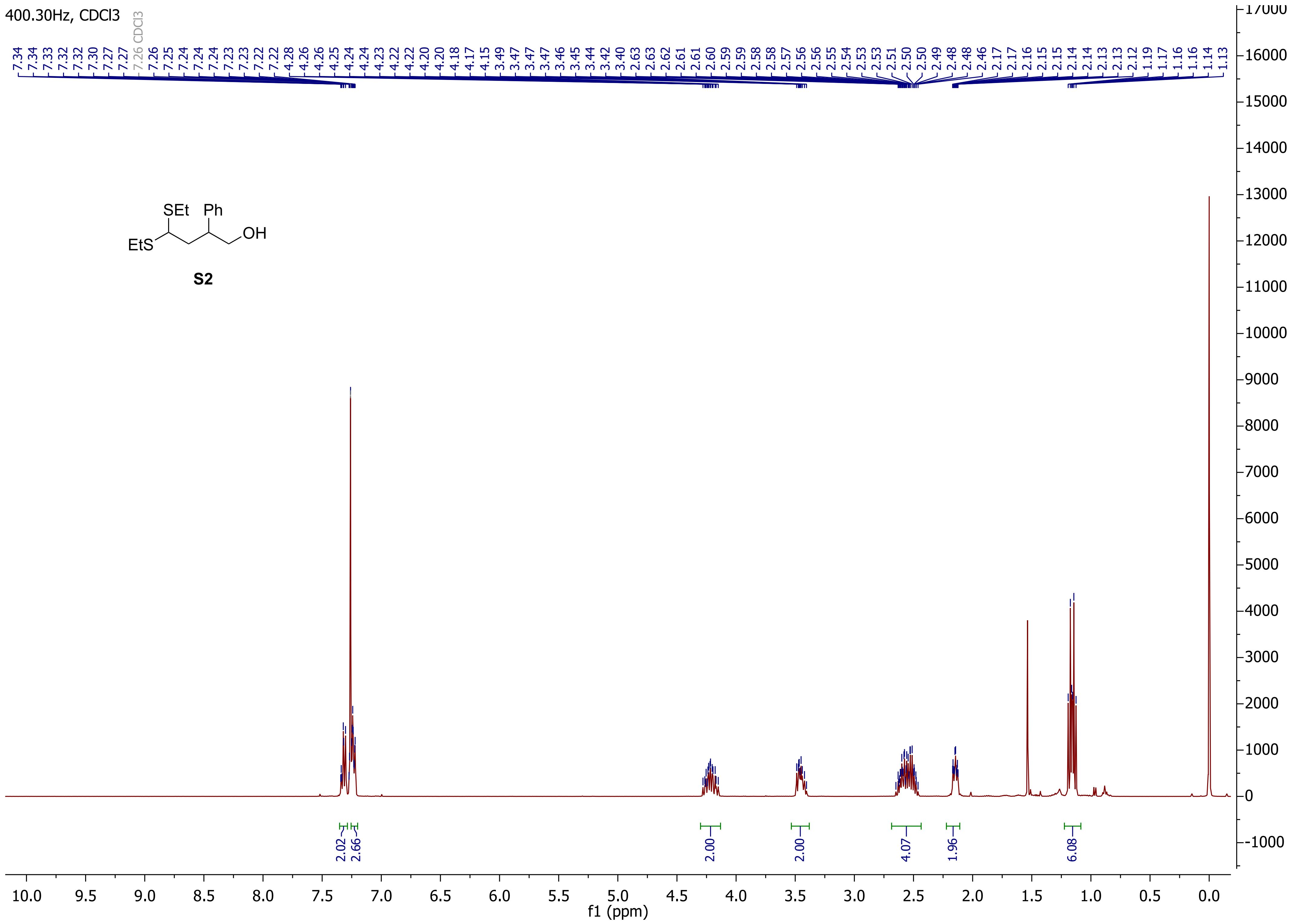
400.30Hz, CDCl3
7.34 7.34 7.33 7.32 7.32 7.30 7.27 7.27 7.26 CDCl3 7.26 7.25 7.24 7.24 7.24 7.23 7.23 7.22 7.22 4.28 4.26 4.26 4.25 4.24 4.24 4.23 4.22 4.22 4.20 4.20 4.18 4.17 4.15 3.49 3.47 3.47 3.46 3.45 3.44 3.42 3.40 2.63 2.63 2.62 2.61 2.61 2.60 2.59 2.59 2.58 2.58 2.57 2.56 2.56 2.55 2.54 2.53 2.53 2.51 2.50 2.50 2.49 2.48 2.48 2.46 2.17 2.17 2.16 2.15 2.15 2.14 2.14 2.13 2.13 2.12 1.19 1.17 1.16 1.16 1.14 1.13
SEt
Ph
EtS
OH
S2
2.02
2.66
2.00
2.00
4.07
1.96
6.08
f1 (ppm)

100.67Hz, CDCl3

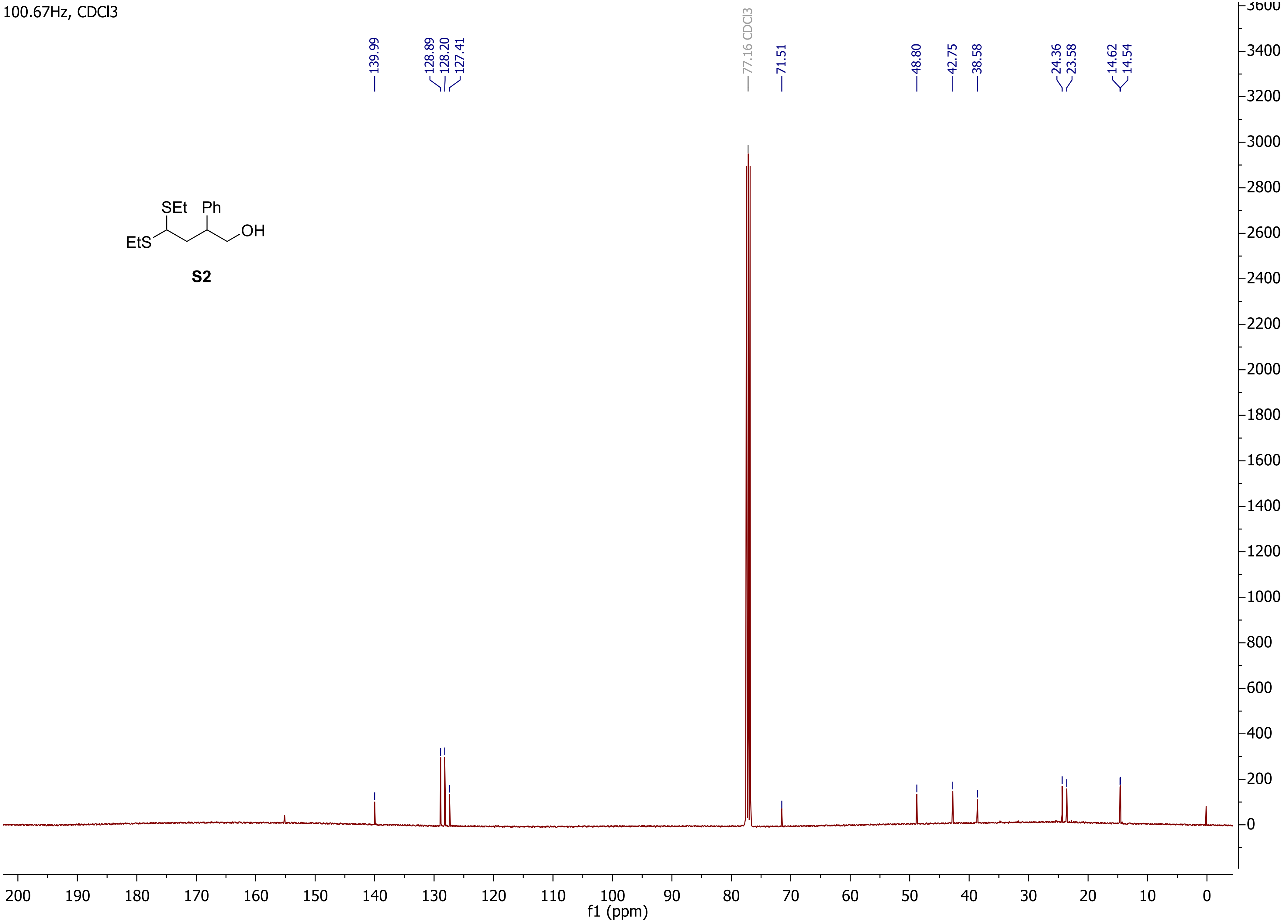

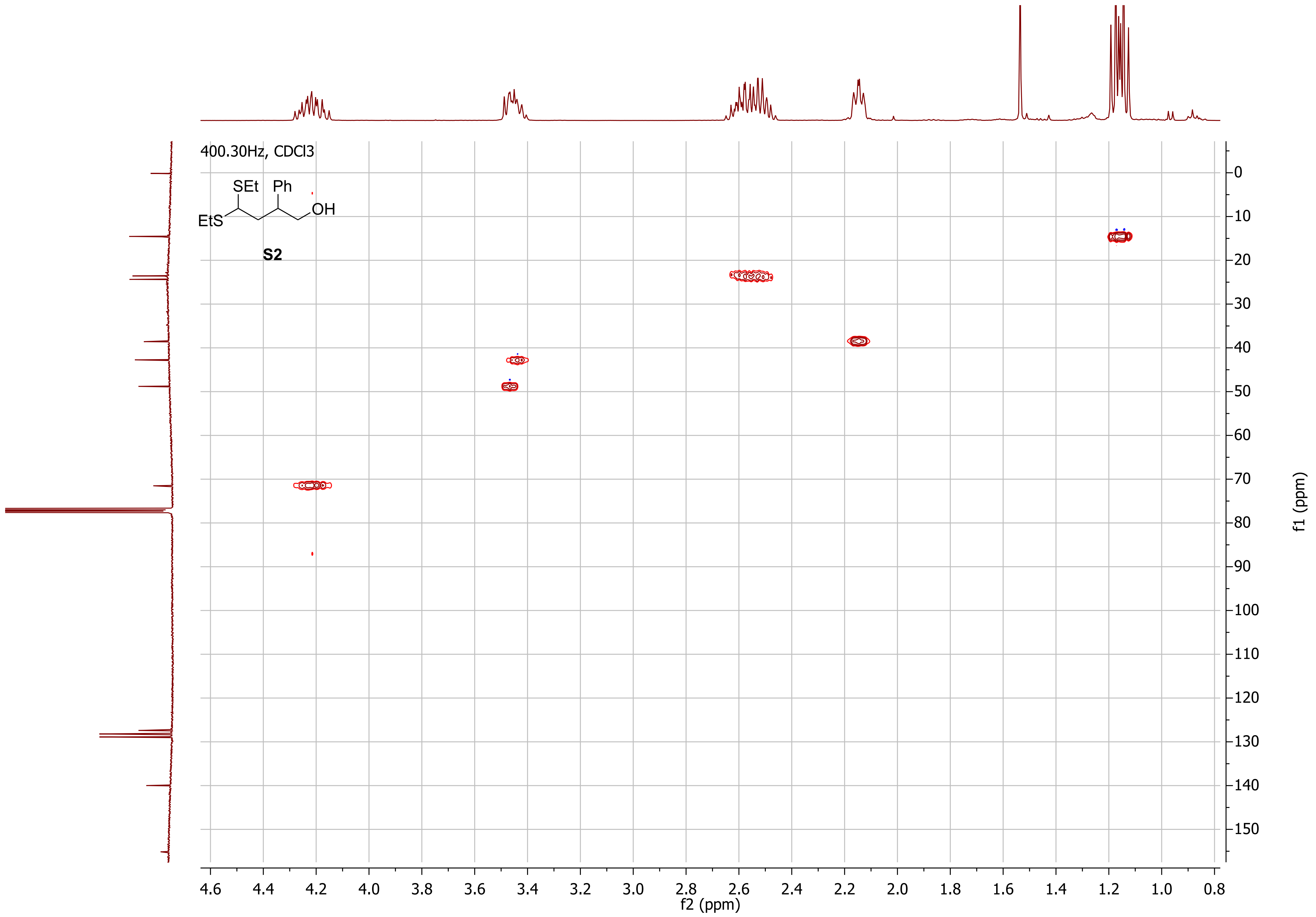

400.30Hz, CDCl3
SEt
Ph
EtS
OH
S2
f2 (ppm)
f1 (ppm)

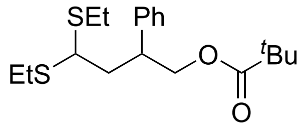

SEt
Ph
EtS
O
tBu
O

S3

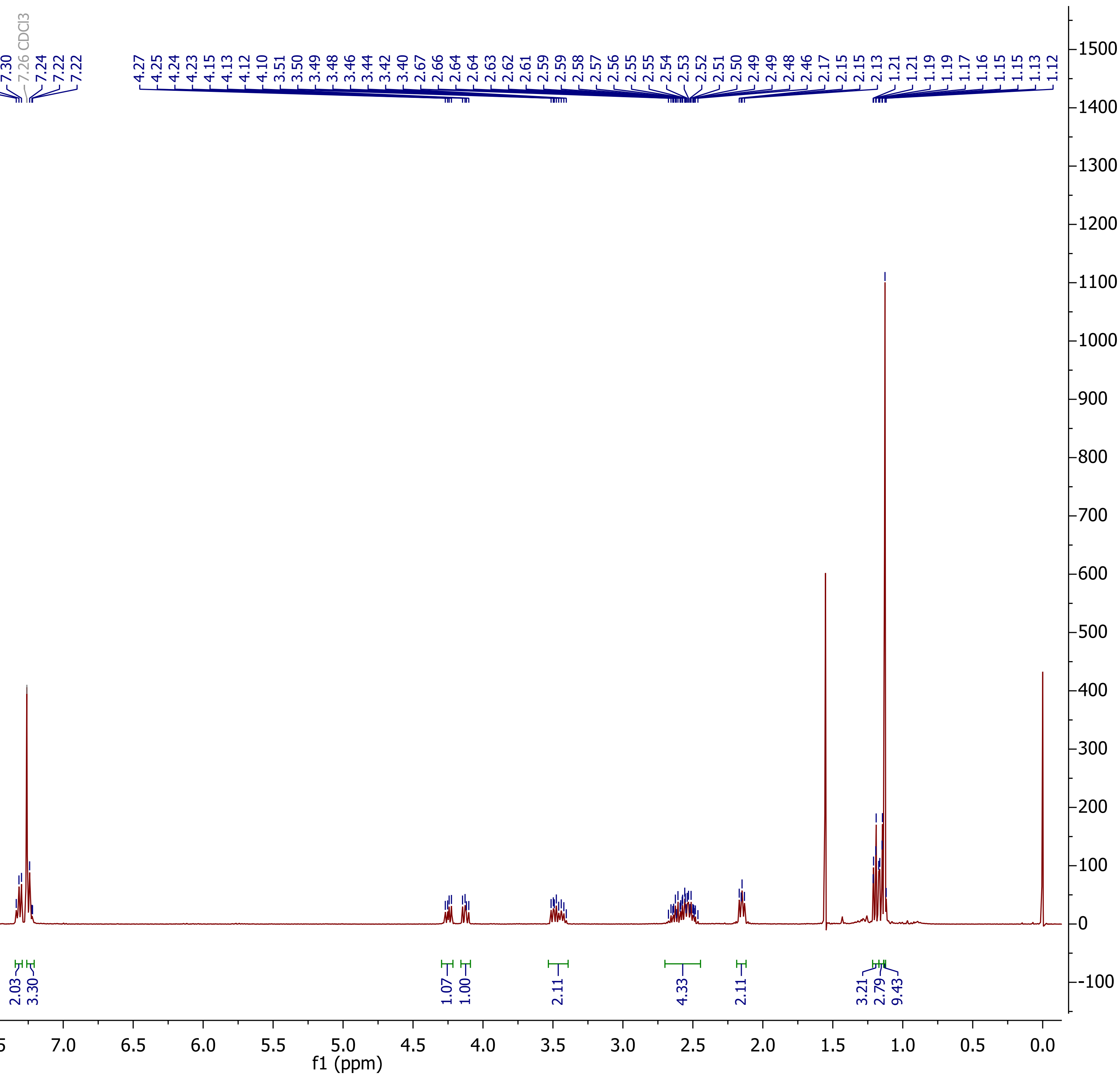

400.30Hz, CDCl3
7.34
7.32
7.30
7.26 CDCl3
7.24
7.22
7.22
4.27
4.25
4.24
4.23
4.15
4.13
4.12
4.10
3.51
3.50
3.49
3.48
3.46
3.44
3.42
3.40
2.67
2.66
2.64
2.64
2.63
2.62
2.61
2.59
2.59
2.58
2.57
2.56
2.55
2.55
2.54
2.53
2.53
2.52
2.51
2.50
2.49
2.49
2.48
2.46
2.17
2.15
2.15
2.13
1.21
1.21
1.19
1.19
1.17
1.16
1.15
1.15
1.13
1.12
2.03
3.30
1.07
1.00
2.11
4.33
2.11
3.21
2.79
9.43
f1 (ppm)

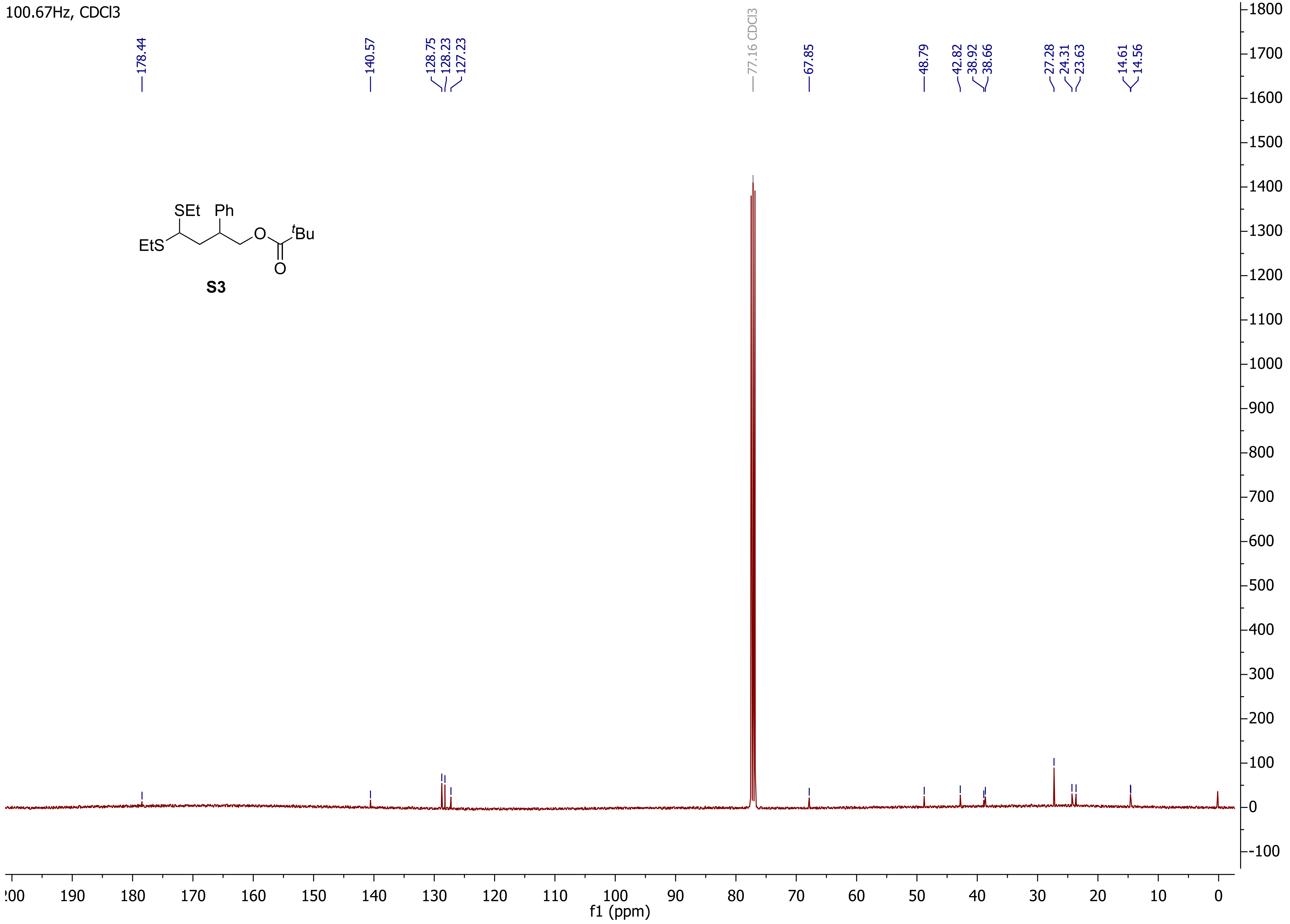
100.67Hz, CDCl3
178.44
140.57
128.75
128.23
127.23
77.16 CDCl3
67.85
48.79
42.82
38.92
38.66
27.28
24.31
23.63
14.61
14.56
SEt
Ph
EtS
O
tBu
O
S3
f1 (ppm)

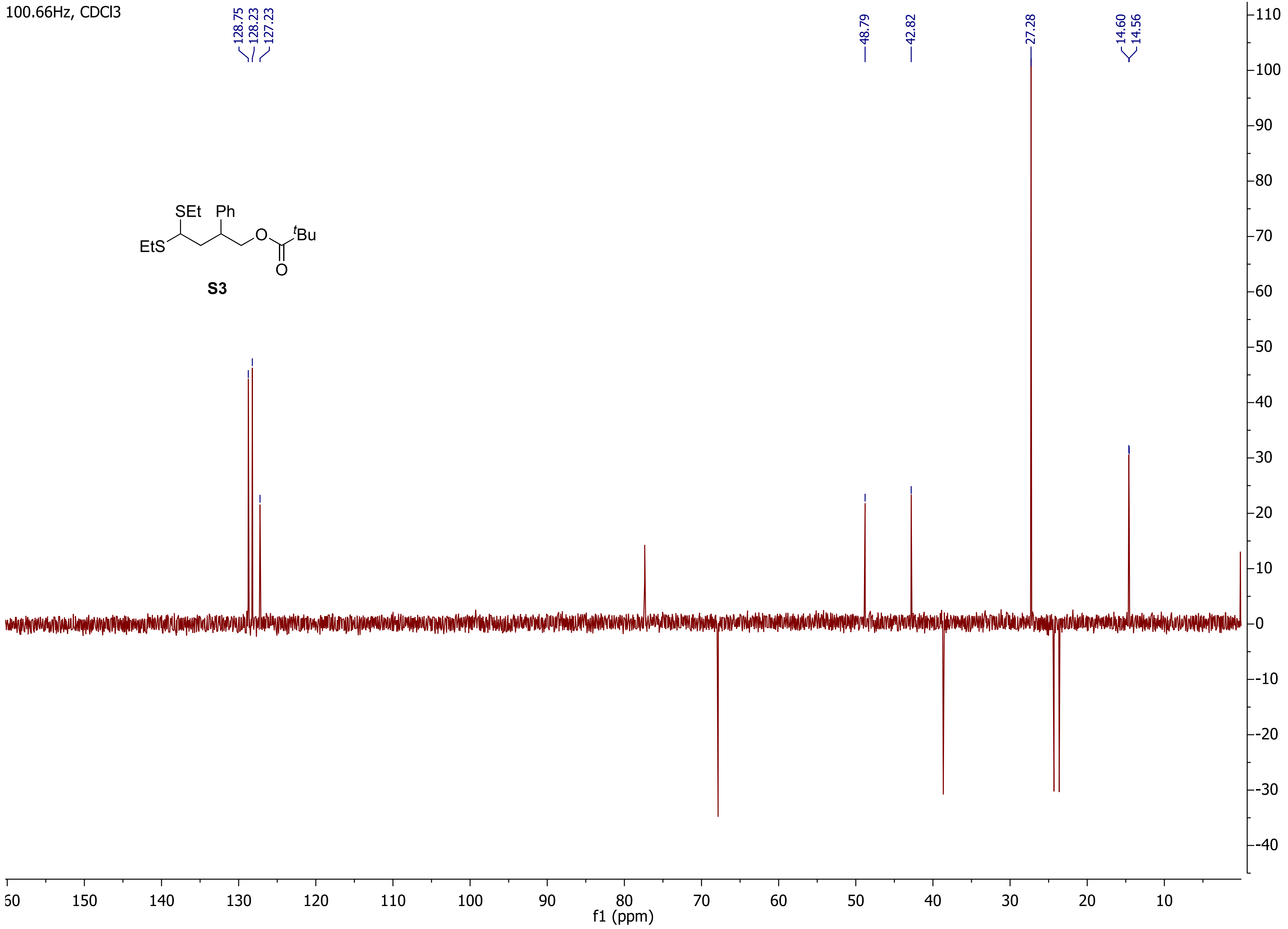
100.66Hz, CDCl3
128.75
128.23
127.23
48.79
42.82
27.28
14.60
14.56
SEt
Ph
EtS
O
tBu
O
S3
f1 (ppm)

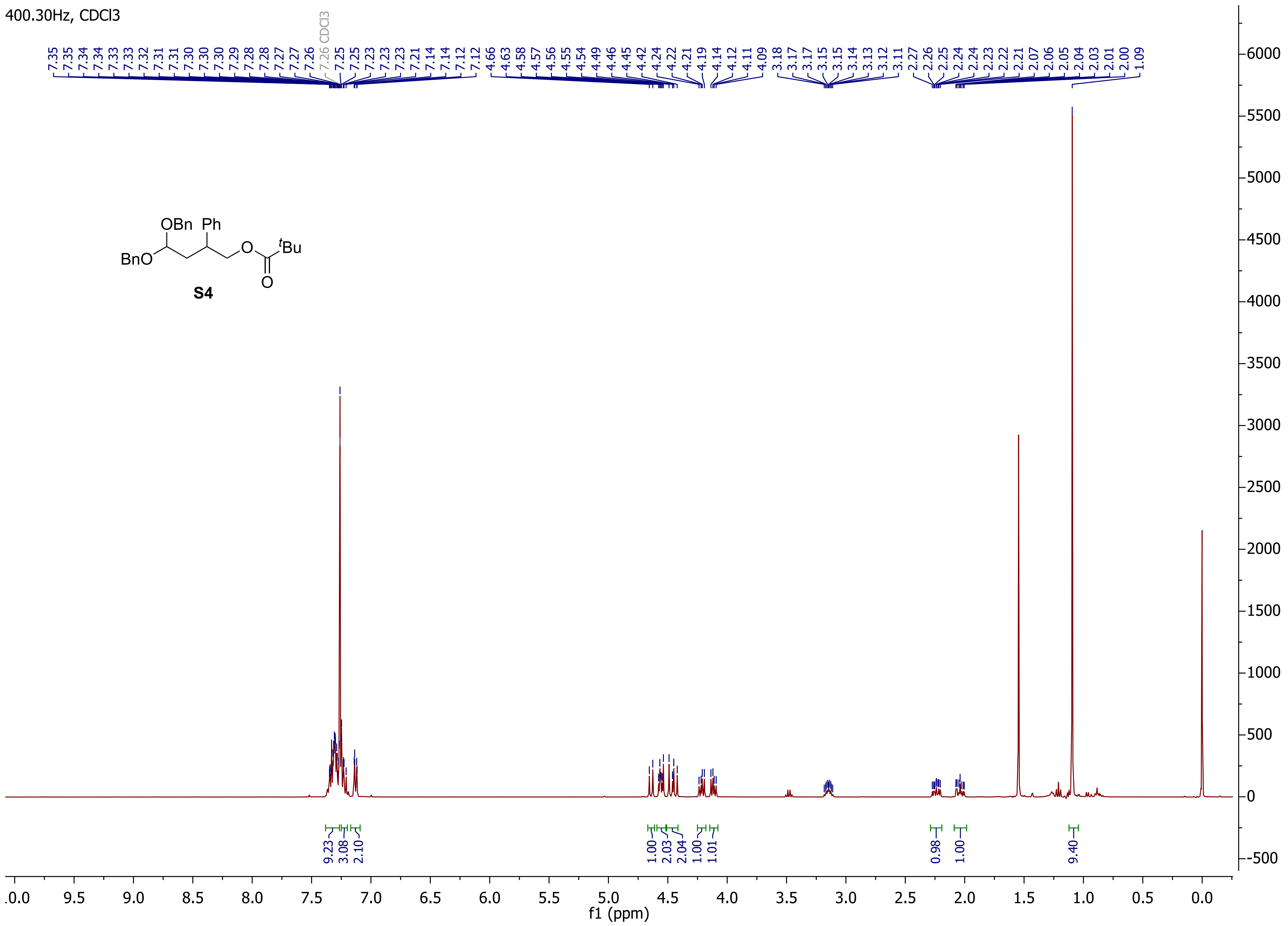
400.30Hz, CDCl3
7.35 7.35 7.34 7.34 7.33 7.33 7.32 7.31 7.31 7.30 7.30 7.30 7.29 7.28 7.28 7.27 7.27 7.26 7.26 CDCl3 7.25 7.25 7.23 7.23 7.23 7.21 7.14 7.14 7.12 7.12 4.66 4.63 4.58 4.57 4.56 4.55 4.54 4.49 4.46 4.45 4.42 4.24 4.22 4.21 4.19 4.14 4.12 4.11 4.09 3.18 3.17 3.17 3.15 3.15 3.14 3.13 3.12 3.11 2.27 2.26 2.25 2.24 2.24 2.23 2.22 2.21 2.07 2.06 2.05 2.04 2.03 2.01 2.00 1.09
OBn Ph
BnO
O
tBu
O
S4
9.23 3.08 2.10 1.00 2.03 2.04 1.00 1.01 0.98 1.00 9.40
f1 (ppm)

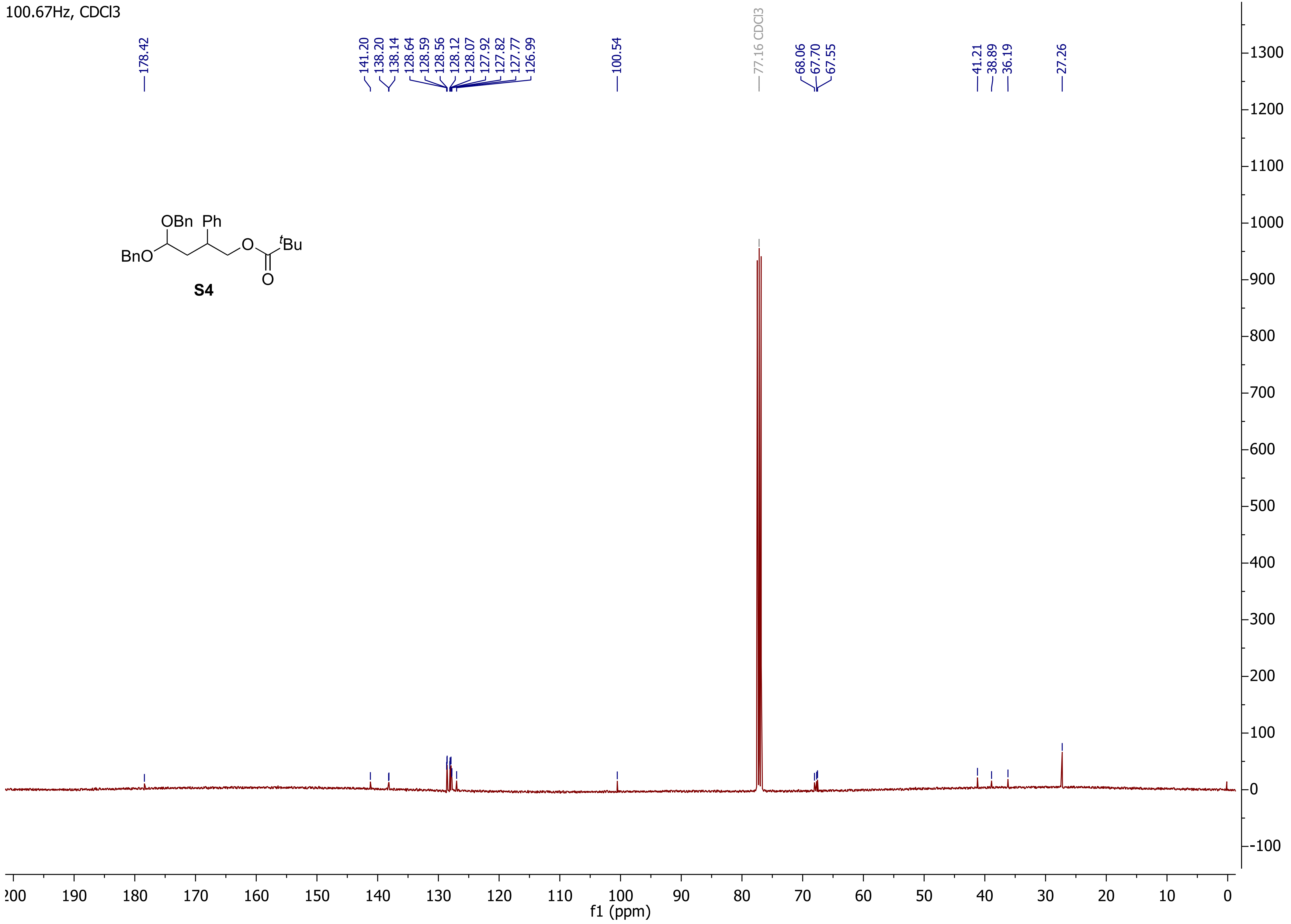
100.67Hz, CDCl3
178.42
141.20
138.20
138.14
128.64
128.59
128.56
128.12
128.07
127.92
127.82
127.77
126.99
100.54
77.16 CDCl3
68.06
67.70
67.55
41.21
38.89
36.19
27.26
OBn
Ph
BnO
O
tBu
O
S4
f1 (ppm)

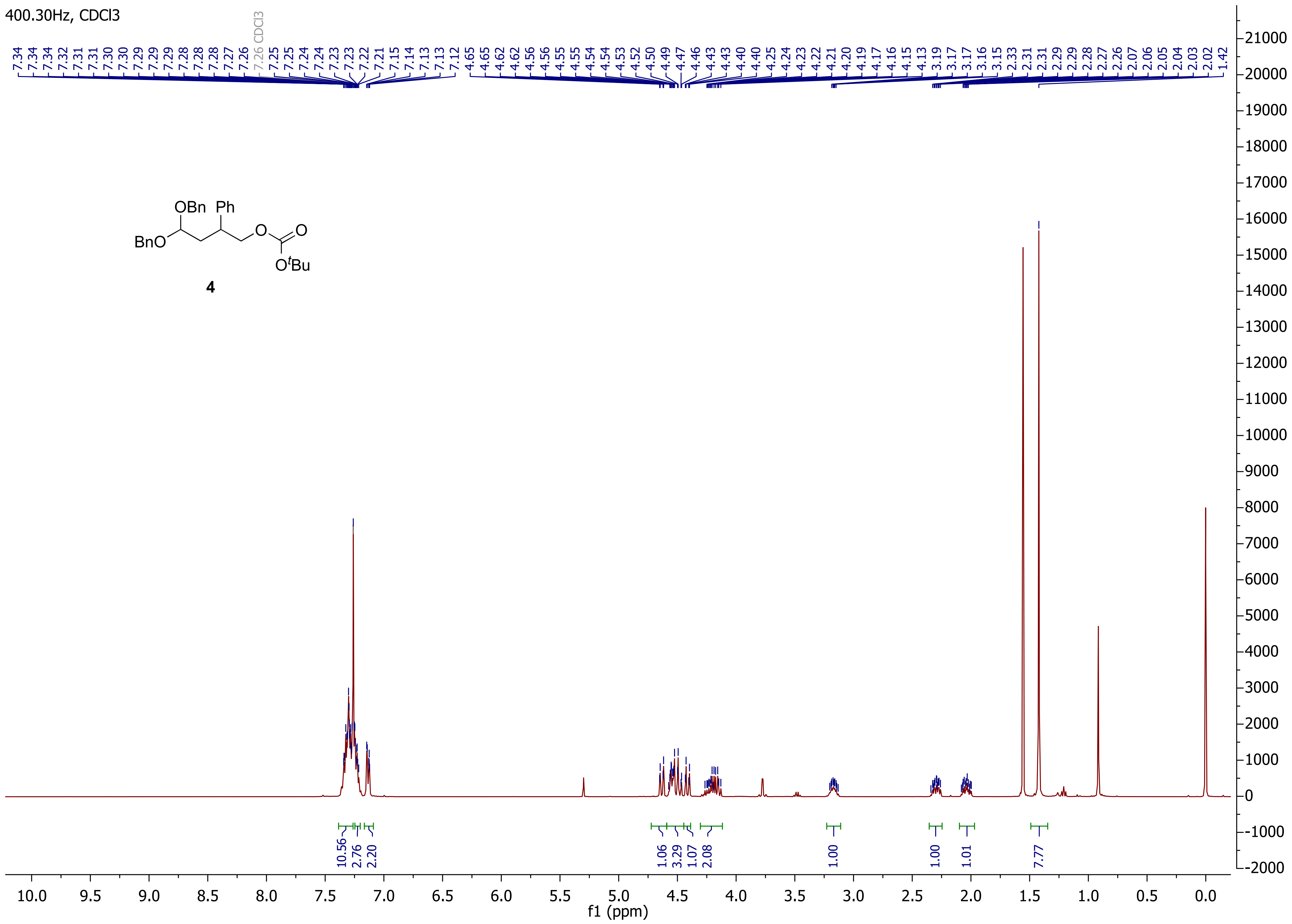
400.30Hz, CDCl3
7.34 7.34 7.34 7.32 7.31 7.31 7.30 7.30 7.29 7.29 7.29 7.28 7.28 7.28 7.27 7.26 7.26 CDCl3 7.25 7.25 7.24 7.24 7.23 7.23 7.22 7.21 7.15 7.14 7.13 7.13 7.12
4.65 4.65 4.62 4.62 4.56 4.56 4.55 4.55 4.54 4.54 4.53 4.52 4.50 4.49 4.47 4.46 4.43 4.43 4.40 4.40 4.25 4.24 4.23 4.22 4.21 4.20 4.19 4.17 4.16 4.15 4.13
3.19 3.17 3.17 3.16 3.15 2.33 2.31 2.31 2.29 2.29 2.28 2.27 2.26 2.07 2.06 2.05 2.04 2.03 2.02 1.42
OBn
Ph
BnO
O
O
O^{t}Bu
4
10.56
2.76
2.20
1.06
3.29
1.07
2.08
1.00
1.00
1.01
7.77
f1 (ppm)

100.67Hz, CDCl3

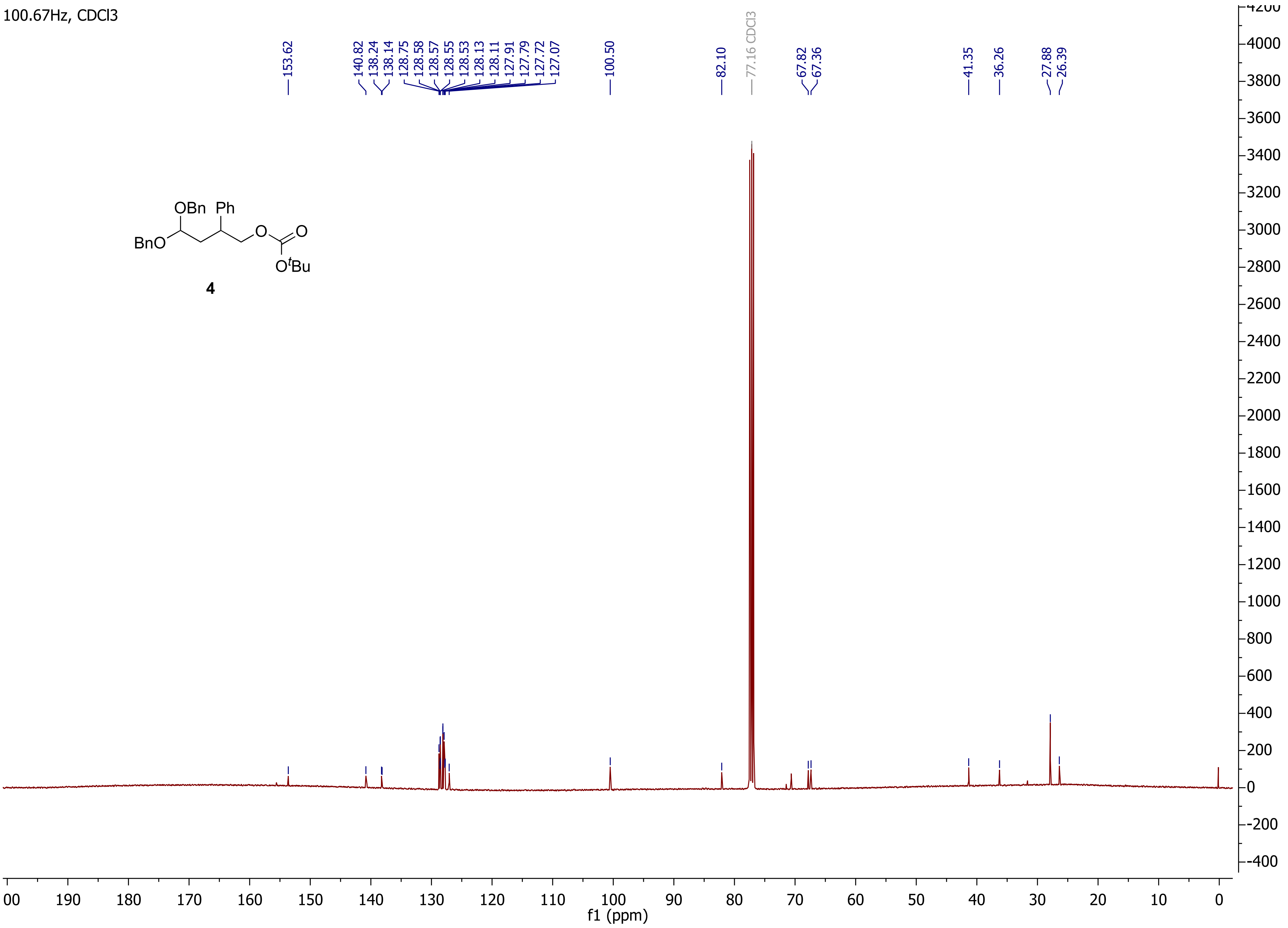

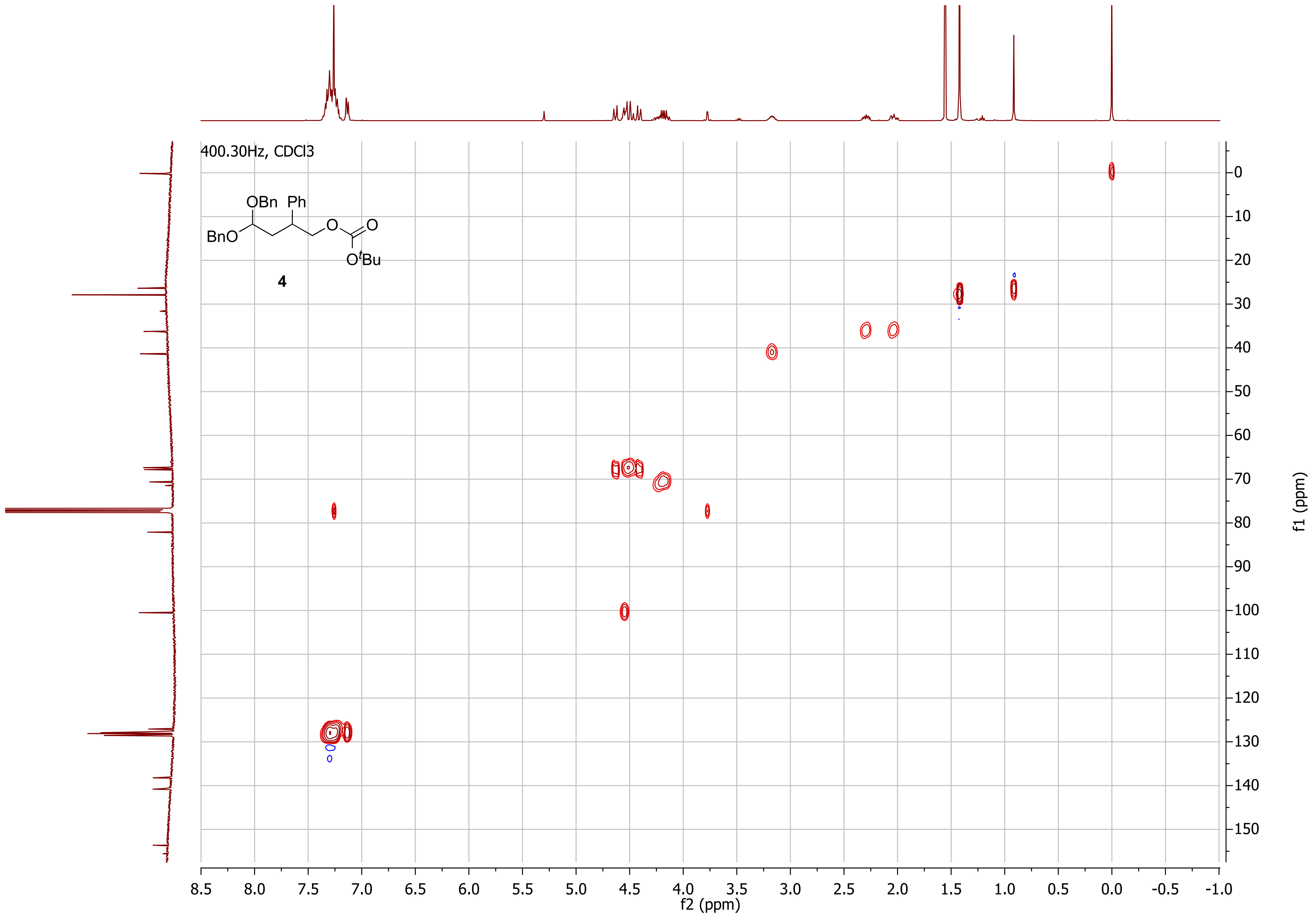

400.30Hz, CDCl3
OBn
Ph
BnO
O
O
O^{t}Bu
4
f2 (ppm)
f1 (ppm)

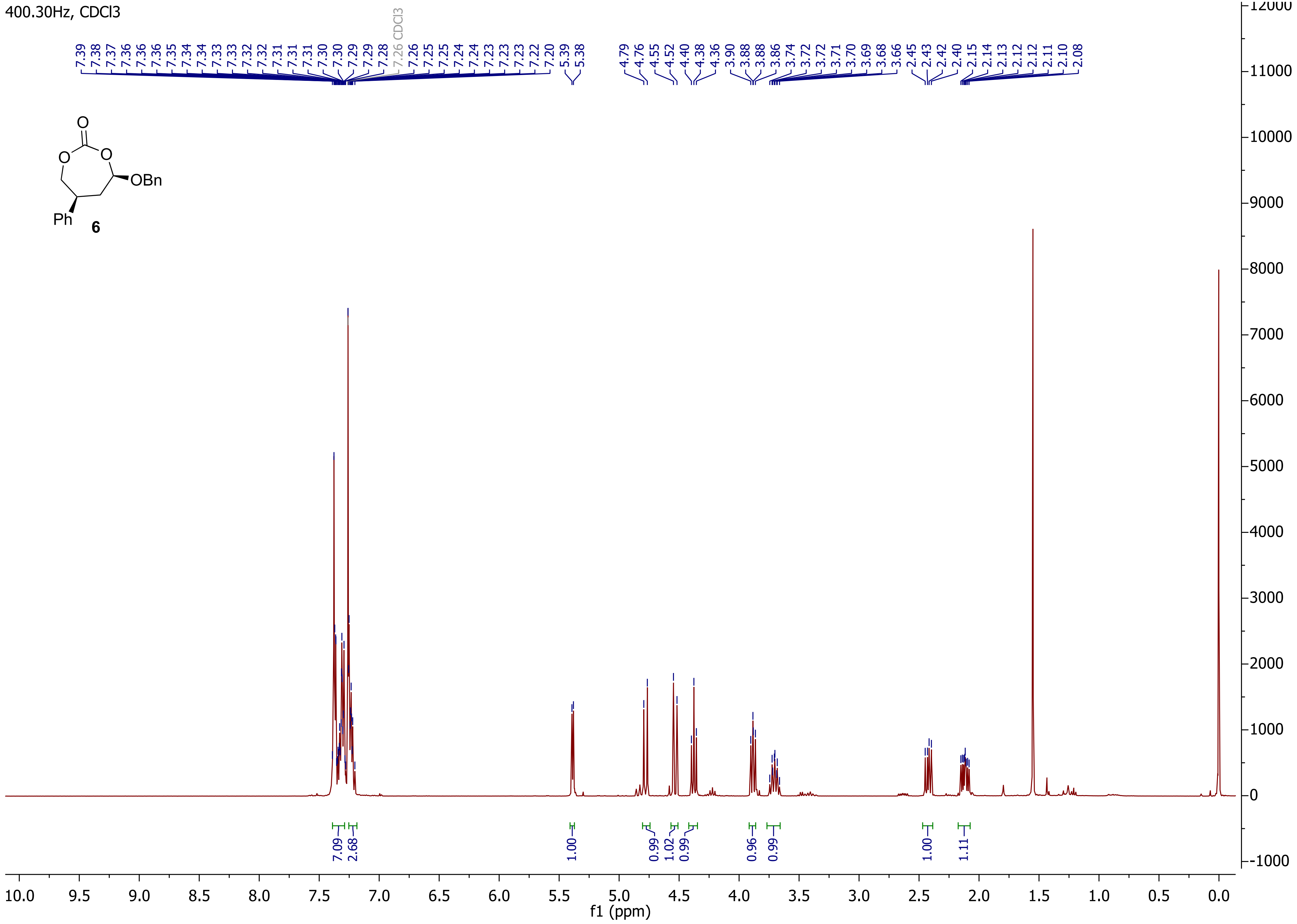

400.30Hz, CDCl3
7.39 7.38 7.37 7.36 7.36 7.36 7.35 7.34 7.34 7.33 7.33 7.32 7.32 7.31 7.31 7.31 7.30 7.30 7.29 7.29 7.28 7.26 CDCl3 7.26 7.25 7.25 7.24 7.24 7.23 7.23 7.23 7.22 7.20 5.39 5.38
4.79 4.76 4.55 4.52 4.40 4.38 4.36 3.90 3.88 3.88 3.86 3.74 3.72 3.72 3.71 3.70 3.69 3.68 3.66 2.45 2.43 2.42 2.40 2.15 2.14 2.13 2.12 2.12 2.11 2.10 2.08
O
O
O
OBn
Ph
6
7.09 2.68 1.00 0.99 1.02 0.99 0.96 0.99 1.00 1.11
f1 (ppm)

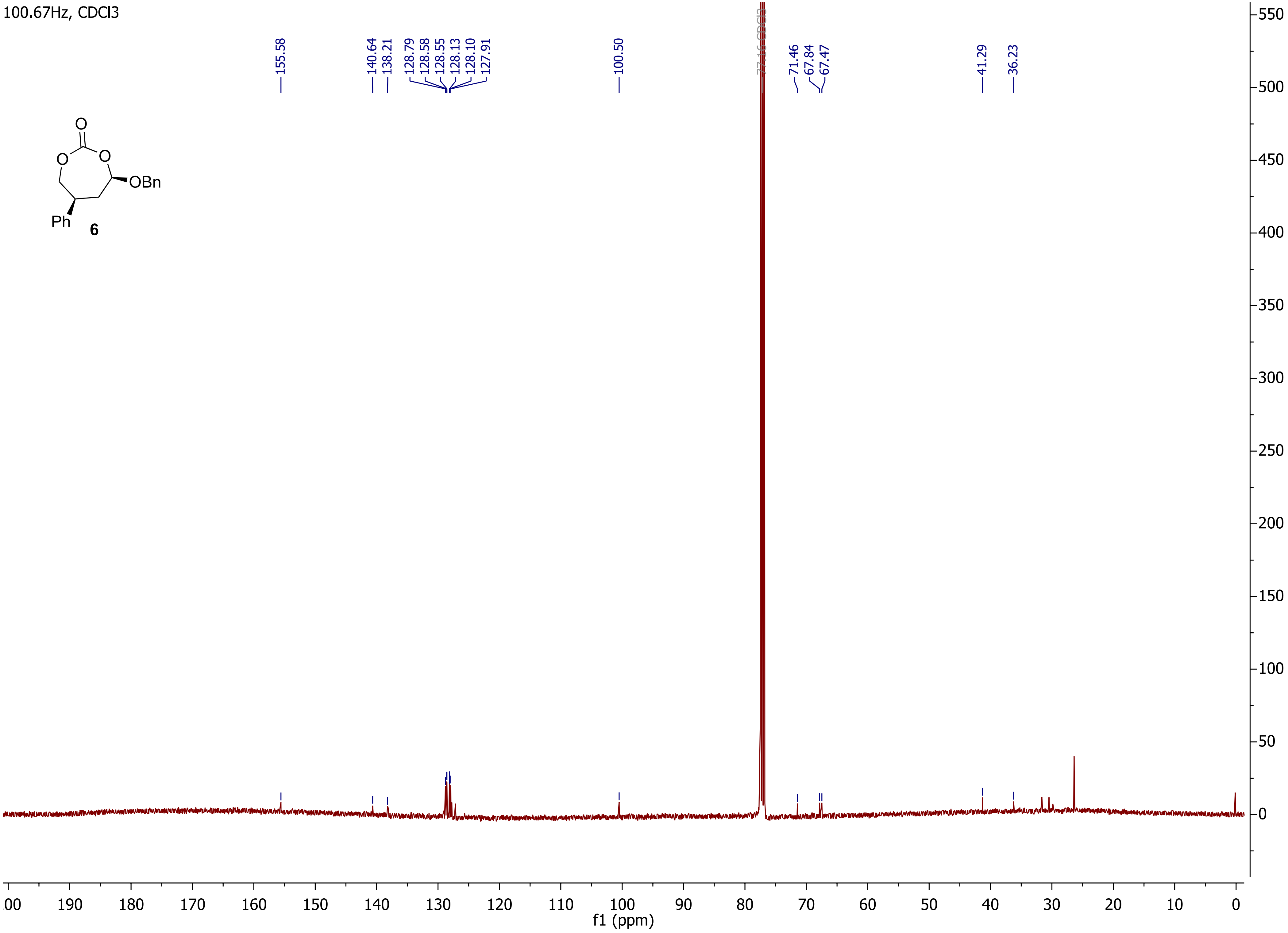
100.67Hz, CDCl3
155.58
140.64
138.21
128.79
128.58
128.55
128.13
128.10
127.91
100.50
77.16 CDCl3
71.46
67.84
67.47
41.29
36.23
OBn
Ph
6
f1 (ppm)

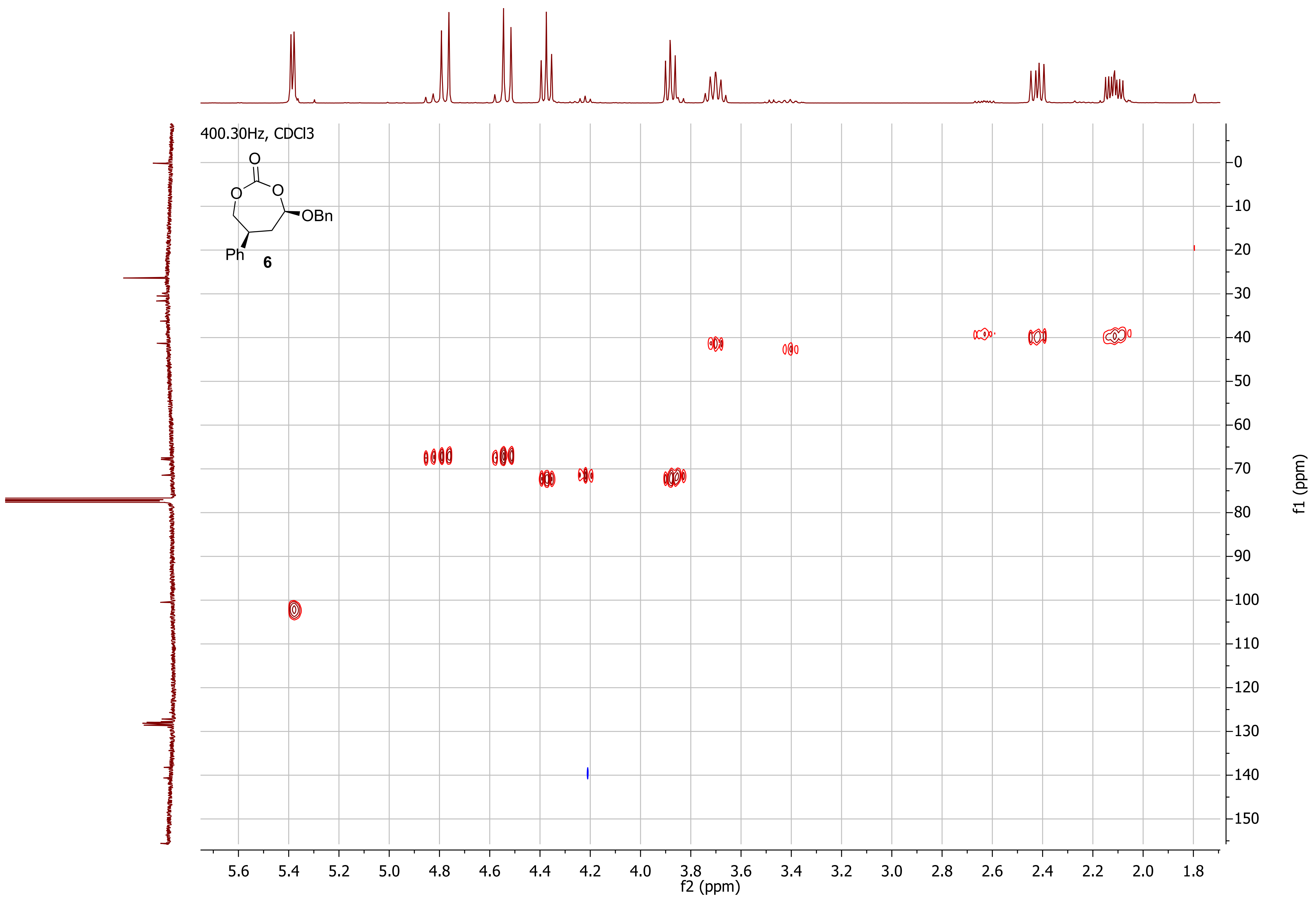

400.30Hz, CDCl3
O
O
O
OBn
Ph
6
f2 (ppm)
f1 (ppm)

400.30Hz, CDCl3

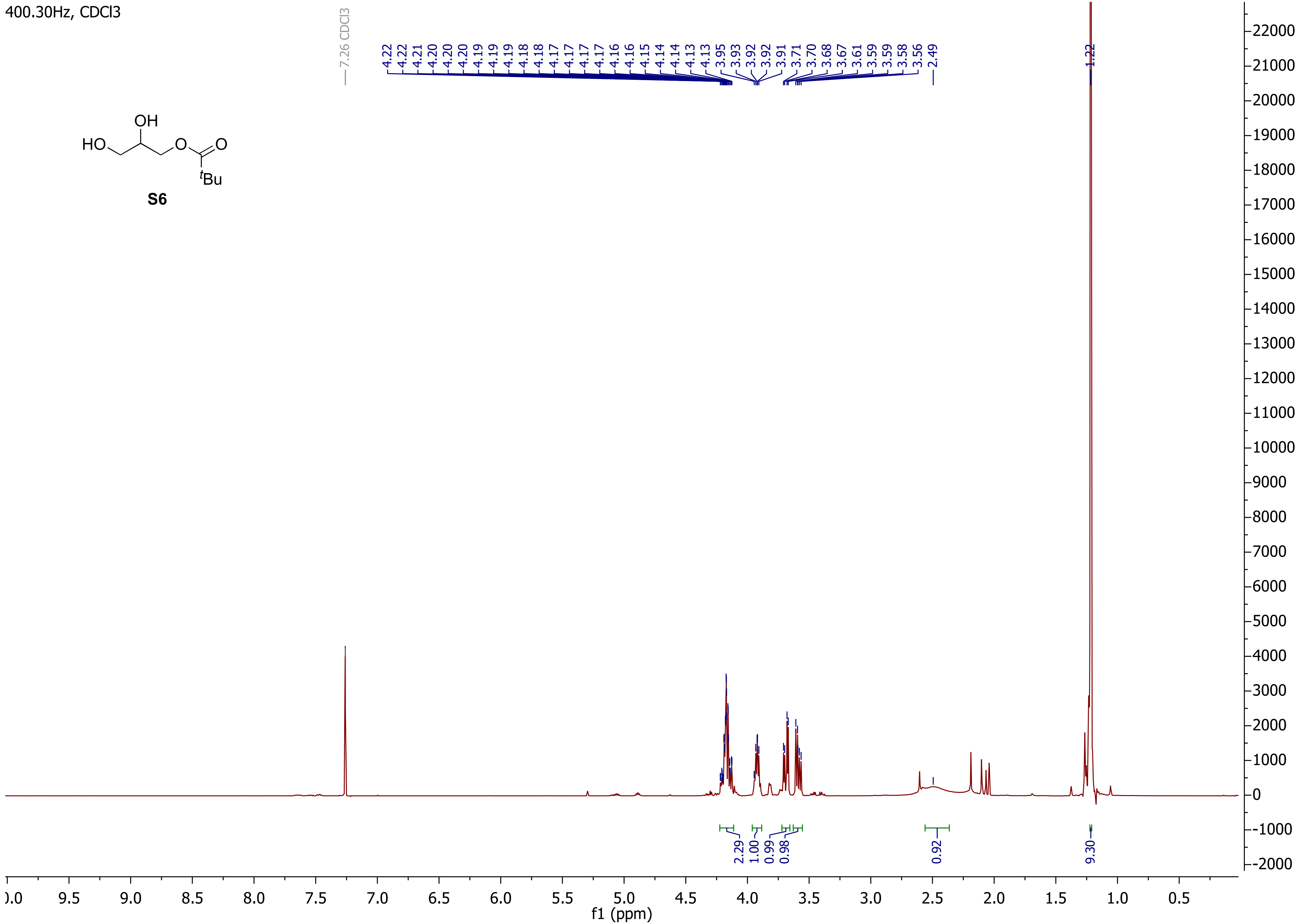

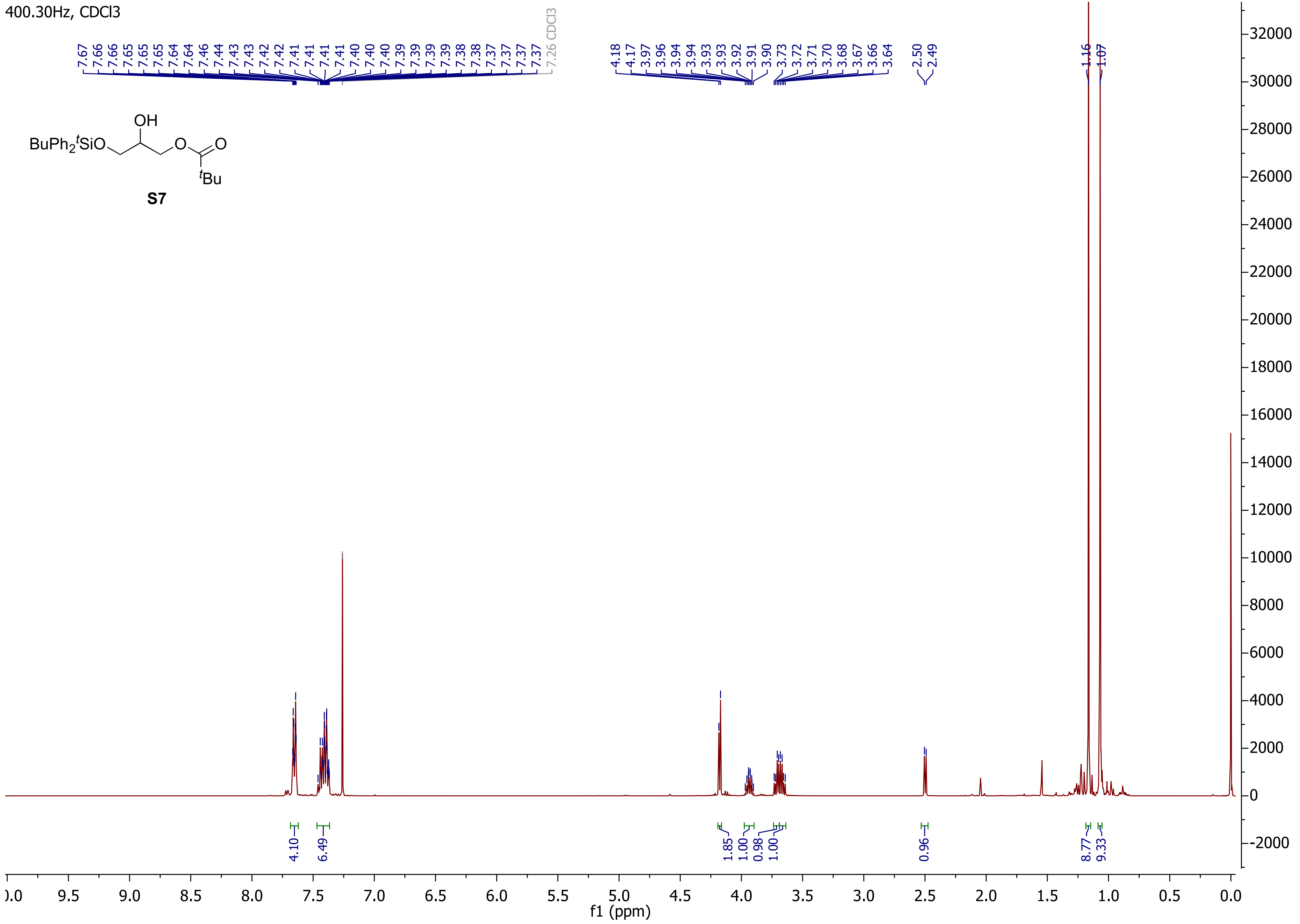
400.30Hz, CDCl3
7.67 7.66 7.66 7.65 7.65 7.64 7.64 7.46 7.44 7.43 7.43 7.42 7.42 7.41 7.41 7.41 7.41 7.40 7.40 7.40 7.39 7.39 7.39 7.39 7.38 7.38 7.37 7.37 7.37 7.37
7.26 CDCl3
4.18 4.17 3.97 3.96 3.94 3.94 3.93 3.93 3.92 3.91 3.90 3.73 3.72 3.71 3.70 3.68 3.67 3.66 3.64
2.50 2.49
1.16 1.07
OH
BuPh2tSiO
O
O
tBu
S7
4.10 6.49 1.85 1.00 0.98 1.00 0.96 8.77 9.33
f1 (ppm)

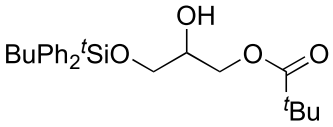

OH
BuPh2tSiO
O
O
tBu

S7

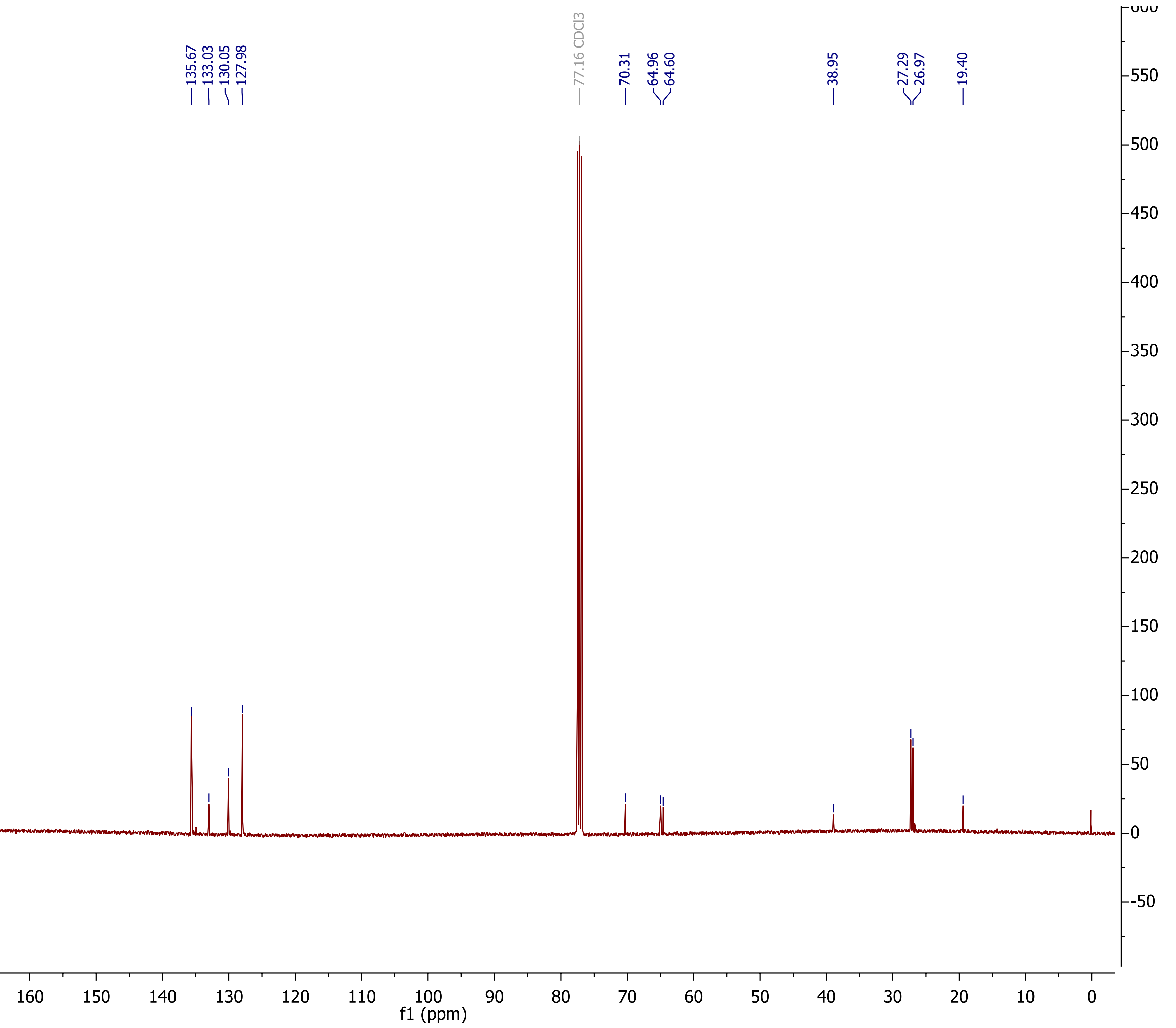

100.67Hz, CDCl3
178.74
135.67
133.03
130.05
127.98
77.16 CDCl3
70.31
64.96
64.60
38.95
27.29
26.97
19.40
f1 (ppm)

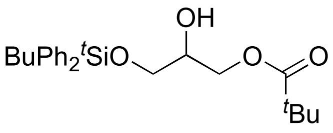

S7

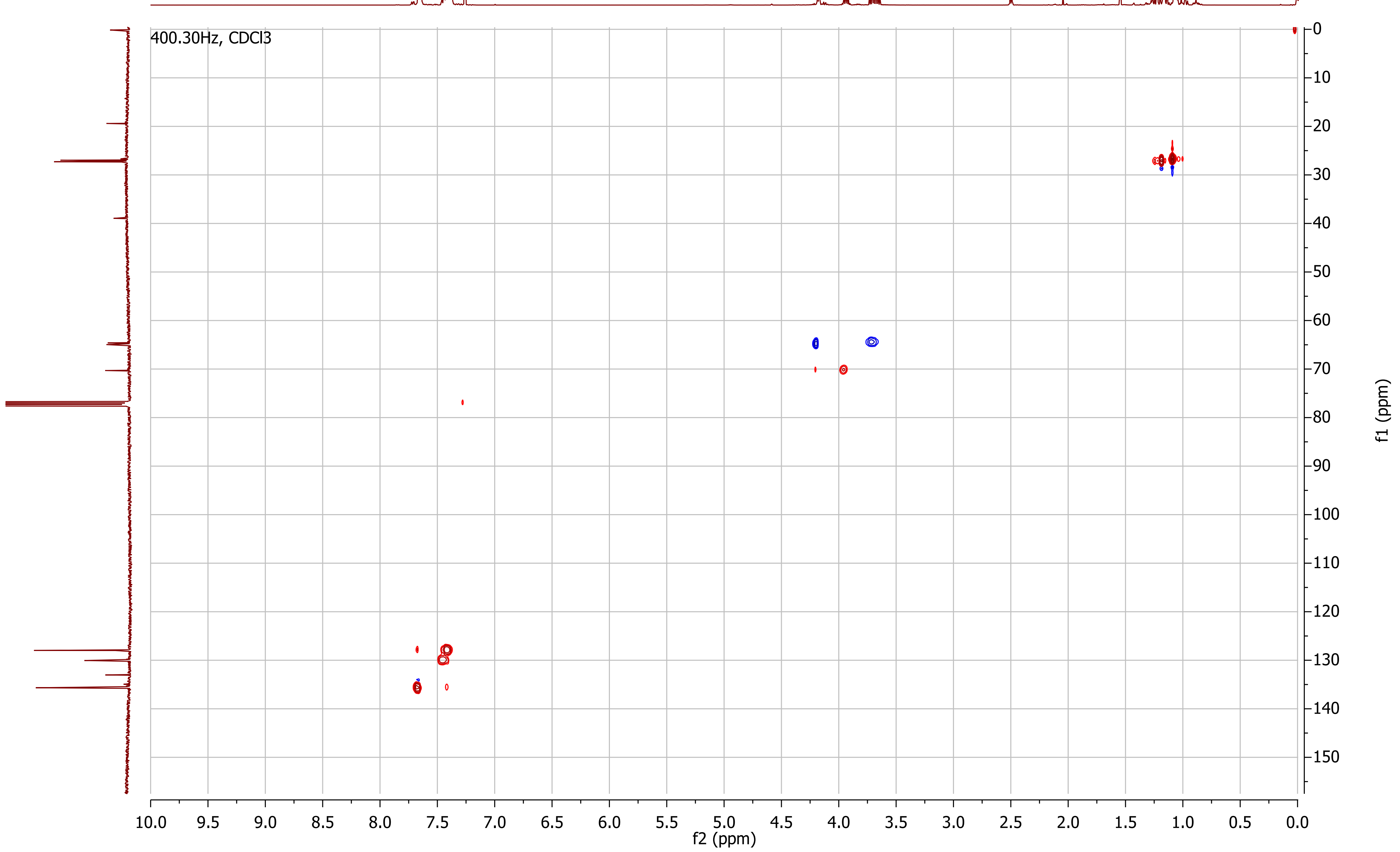

400.30Hz, CDCl3

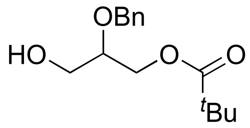

S8

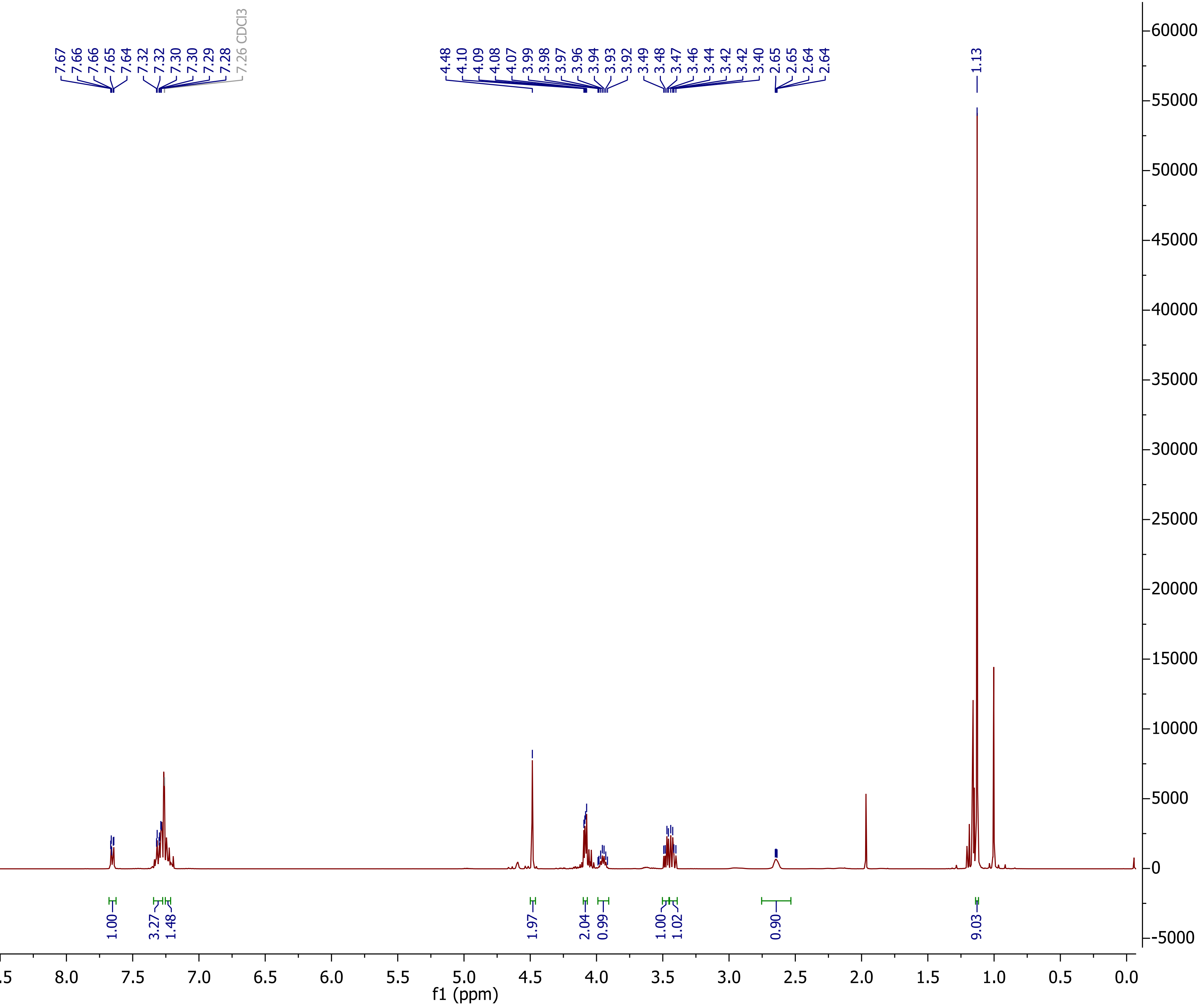

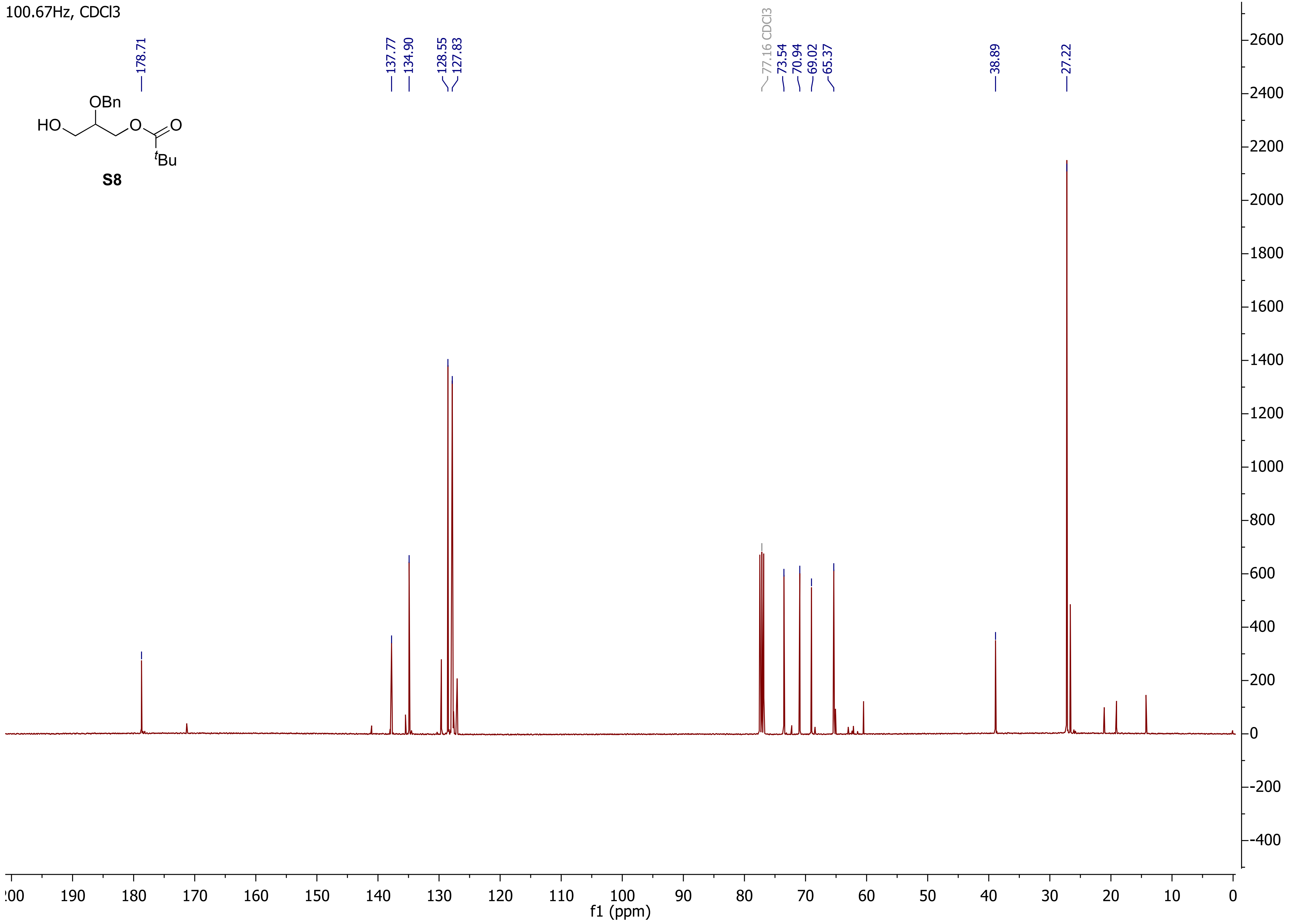

100.67Hz, CDCl3
178.71
137.77
134.90
128.55
127.83
77.16 CDCl3
73.54
70.94
69.02
65.37
38.89
27.22
OBn
HO
O
O
tBu
S8
f1 (ppm)

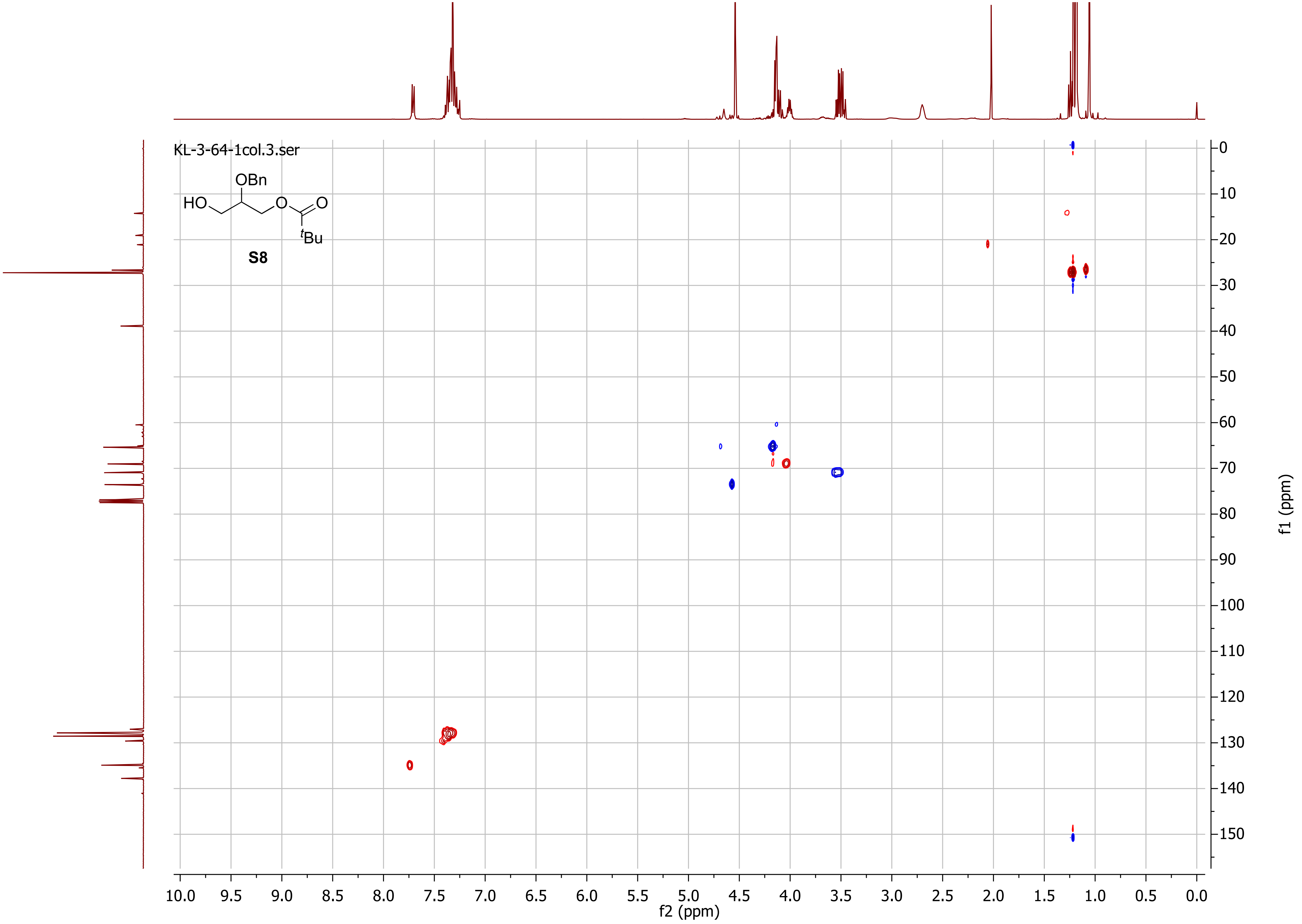
KL-3-64-1col.3.ser
OBn
HO
O
O
tBu
S8
f2 (ppm)
f1 (ppm)

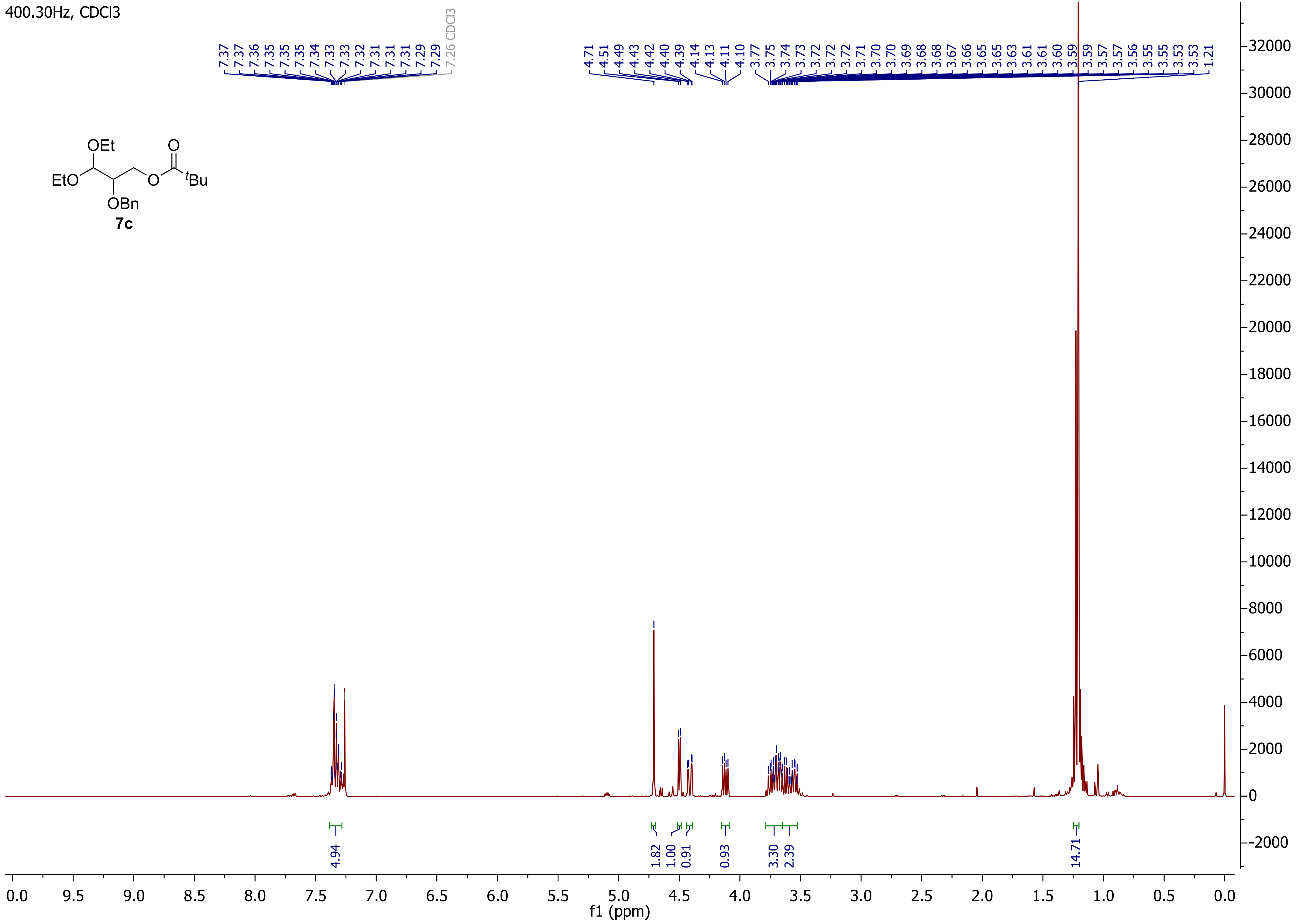
400.30Hz, CDCl3
7.37 7.37 7.36 7.35 7.35 7.35 7.34 7.33 7.33 7.32 7.31 7.31 7.31 7.29 7.29 7.26 CDCl3
4.71 4.51 4.49 4.43 4.42 4.40 4.39 4.14 4.13 4.11 4.10 3.77 3.75 3.74 3.73 3.72 3.72 3.72 3.71 3.70 3.70 3.69 3.68 3.68 3.67 3.66 3.65 3.65 3.63 3.61 3.61 3.60 3.59 3.59 3.57 3.57 3.56 3.55 3.55 3.53 3.53 1.21
OEt
EtO
O
O
tBu
OBn
7c
4.94
1.82
1.00
0.91
0.93
3.30
2.39
14.71
f1 (ppm)

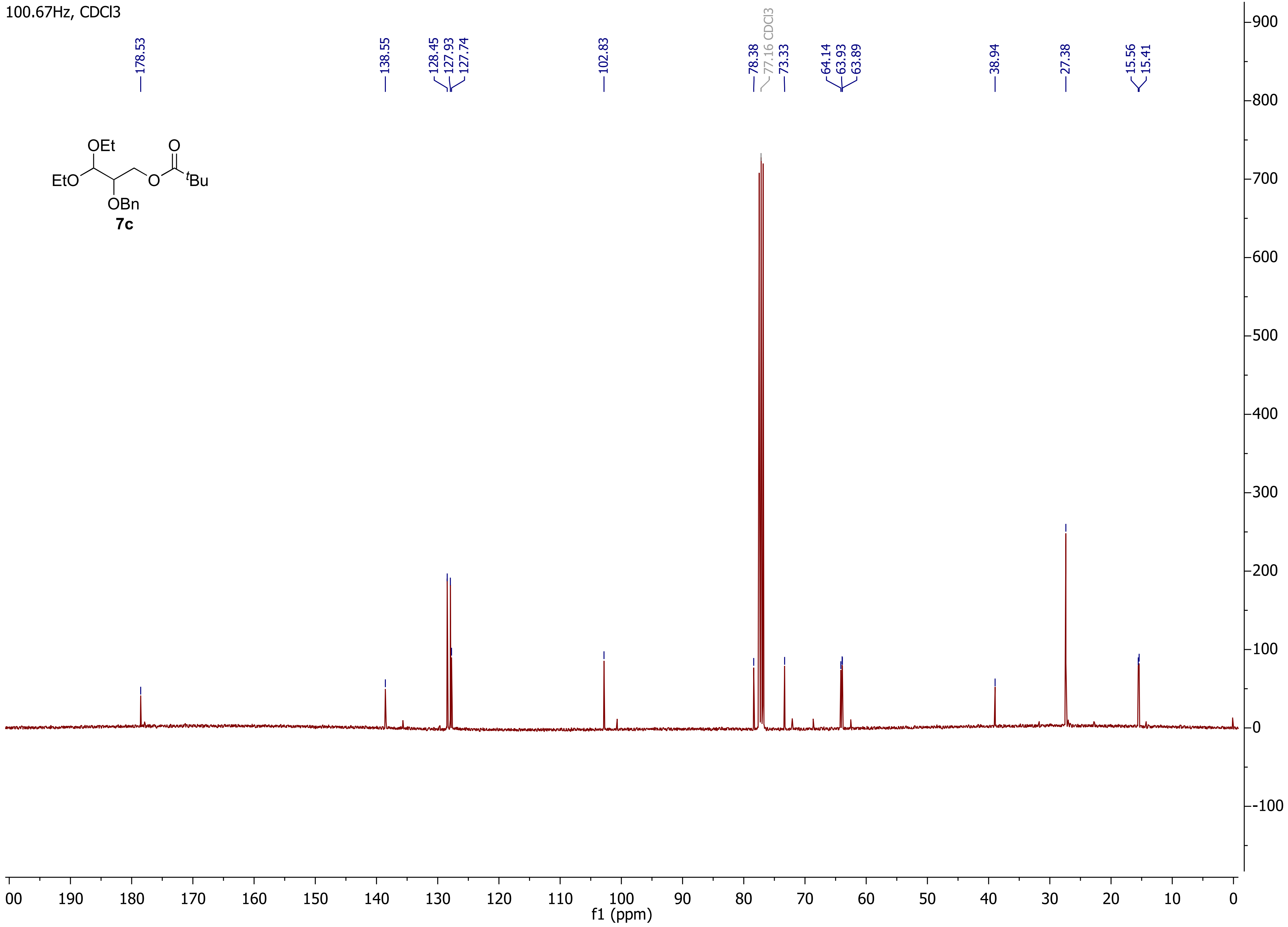

100.67Hz, CDCl3
178.53
138.55
128.45
127.93
127.74
102.83
78.38
77.16 CDCl3
73.33
64.14
63.93
63.89
38.94
27.38
15.56
15.41
OEt
EtO
O
O
tBu
OBn
7c
f1 (ppm)

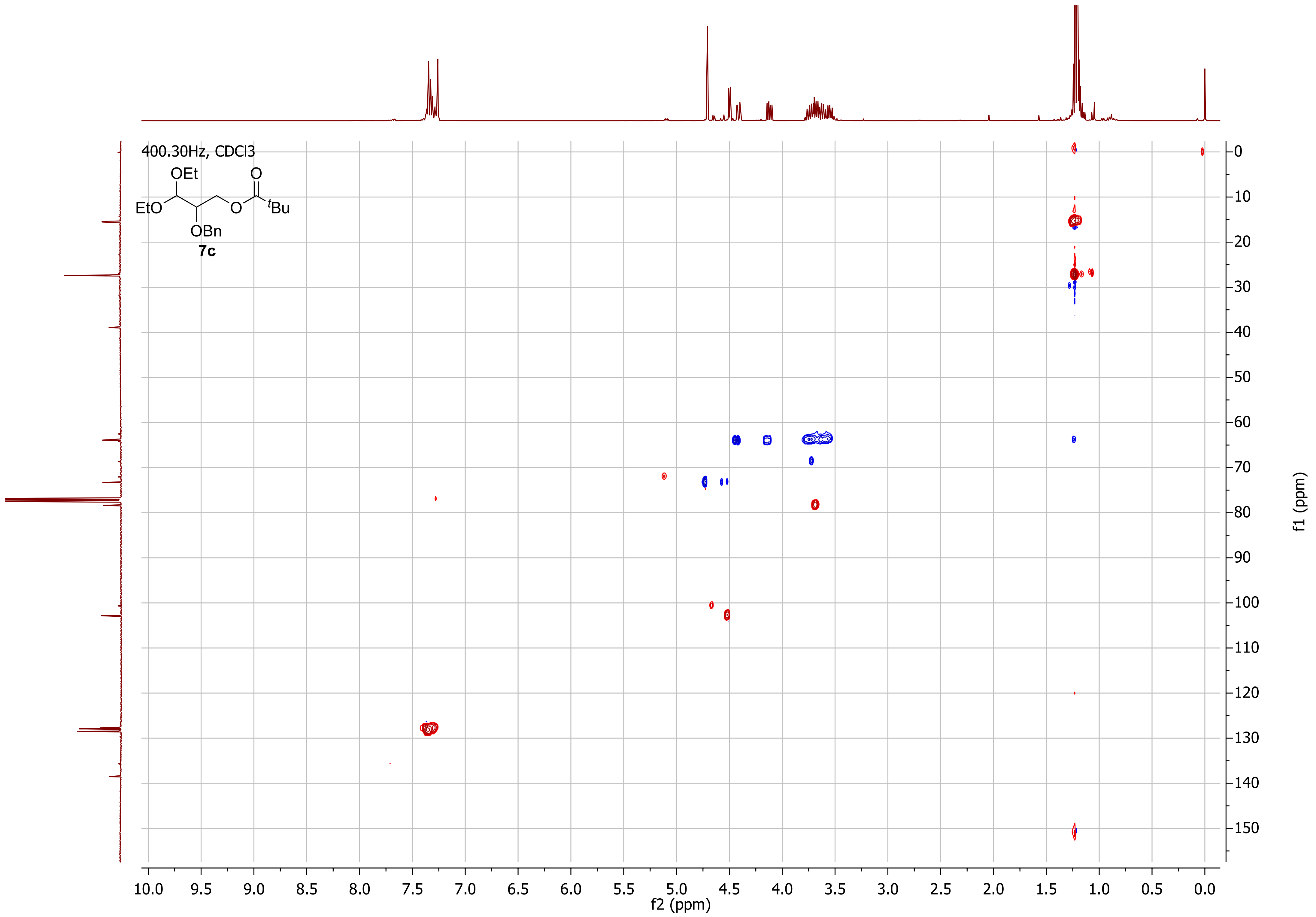

400.30Hz, CDCl3
OEt
O
EtO
O
tBu
OBn
7c
f2 (ppm)
f1 (ppm)

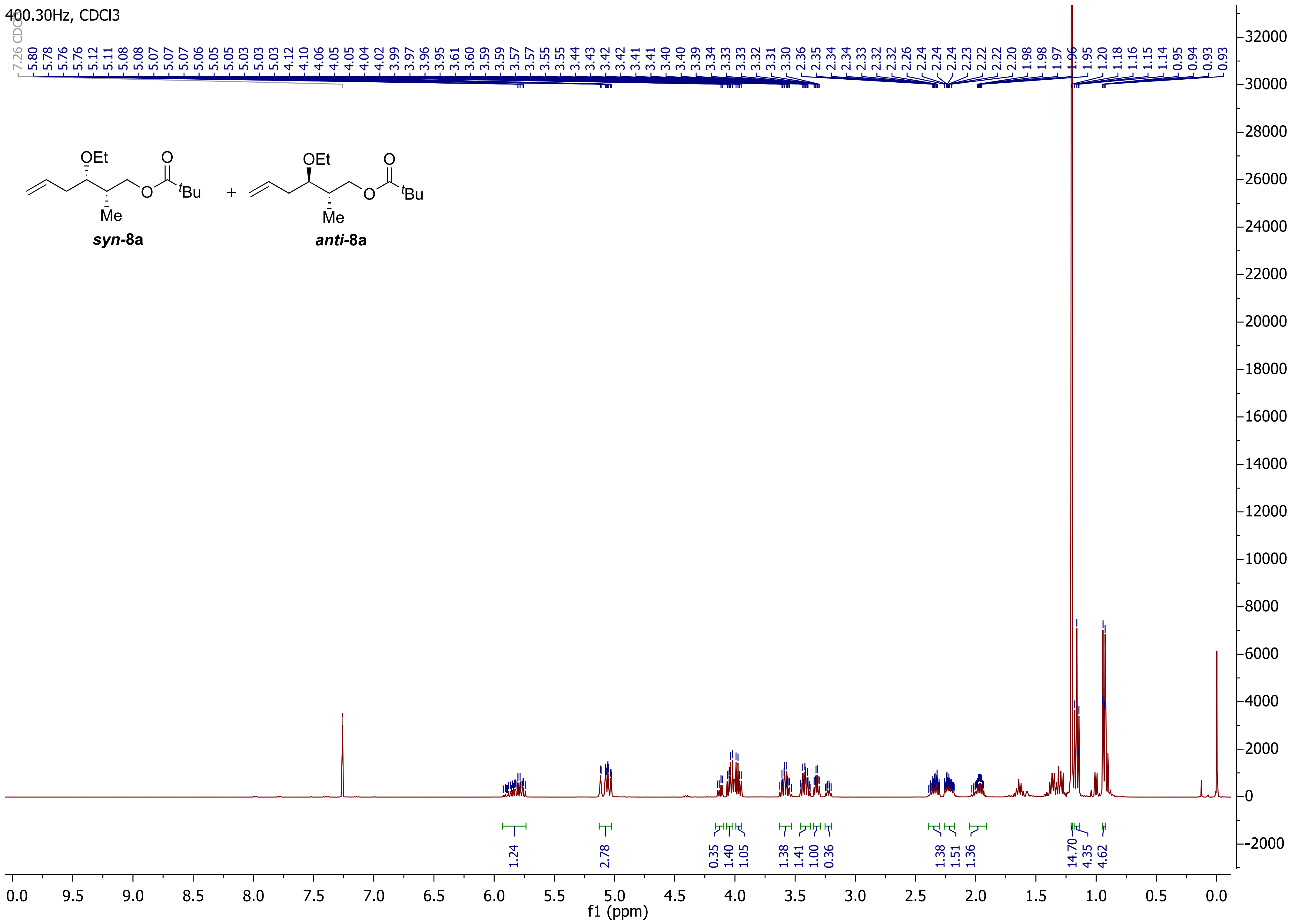

400.30Hz, CDCl3
7.26 CDCl3
5.80 5.78 5.76 5.76 5.12 5.11 5.08 5.08 5.07 5.07 5.07 5.06 5.05 5.05 5.03 5.03 5.03 4.12 4.10 4.06 4.05 4.05 4.04 4.02 3.99 3.97 3.96 3.95 3.61 3.60 3.59 3.59 3.57 3.57 3.55 3.55 3.44 3.43 3.42 3.42 3.41 3.41 3.40 3.40 3.39 3.34 3.33 3.33 3.32 3.31 3.30 2.36 2.35 2.34 2.34 2.33 2.32 2.32 2.26 2.24 2.24 2.24 2.23 2.22 2.22 2.20 1.98 1.98 1.97 1.96 1.95 1.20 1.18 1.16 1.15 1.14 0.95 0.94 0.93 0.93
OEt
O
Me
O
tBu
+
syn-8a
anti-8a
1.24
2.78
0.35
1.40
1.05
1.38
1.41
1.00
0.36
1.38
1.51
1.36
14.70
4.35
4.62
f1 (ppm)

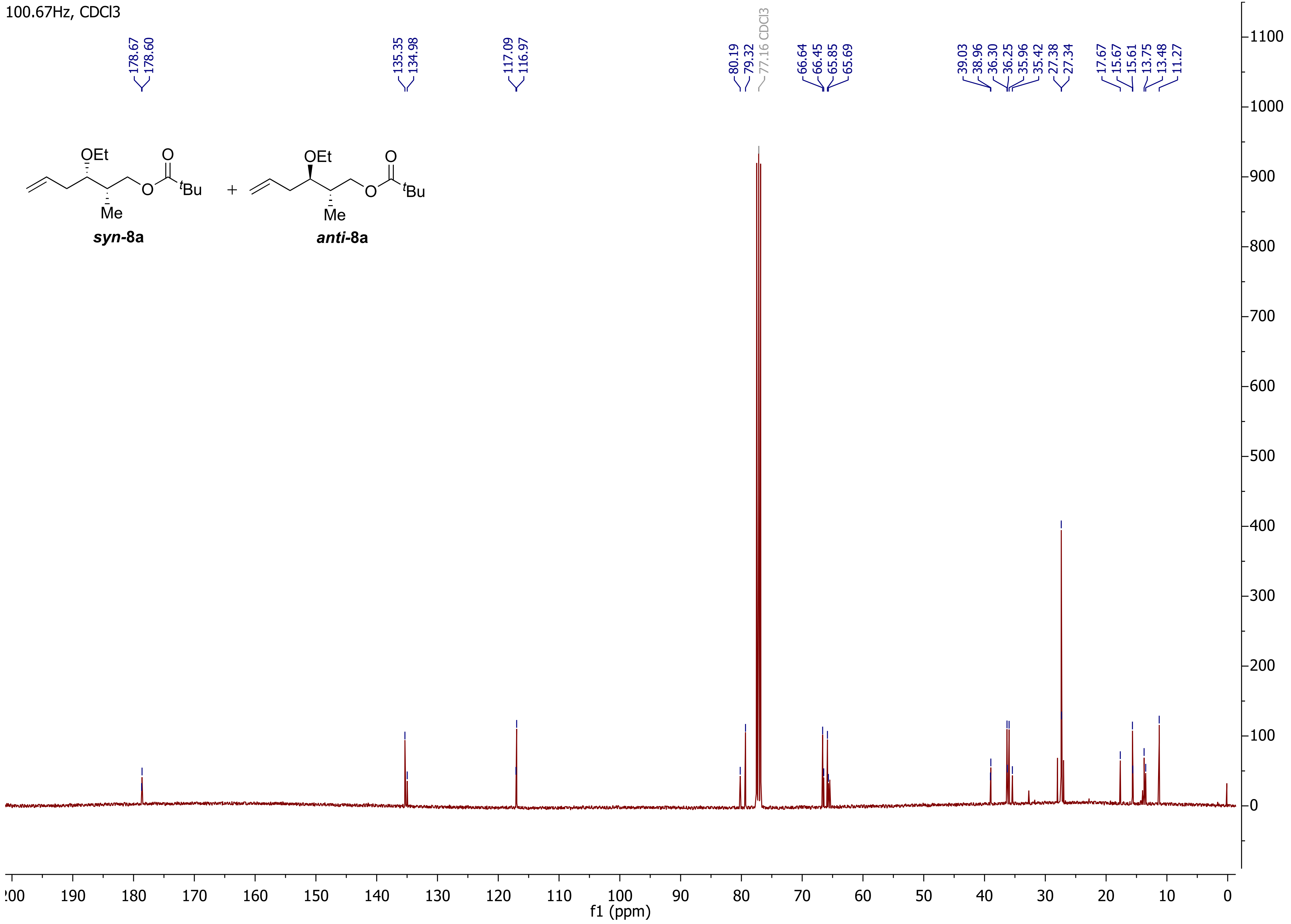
100.67Hz, CDCl3
178.67
178.60
135.35
134.98
117.09
116.97
80.19
79.32
77.16 CDCl3
66.64
66.45
65.85
65.69
39.03
38.96
36.30
36.25
35.96
35.42
27.38
27.34
17.67
15.67
15.61
13.75
13.48
11.27
OEt
O
O
tBu
Me
syn-8a
+
anti-8a
f1 (ppm)

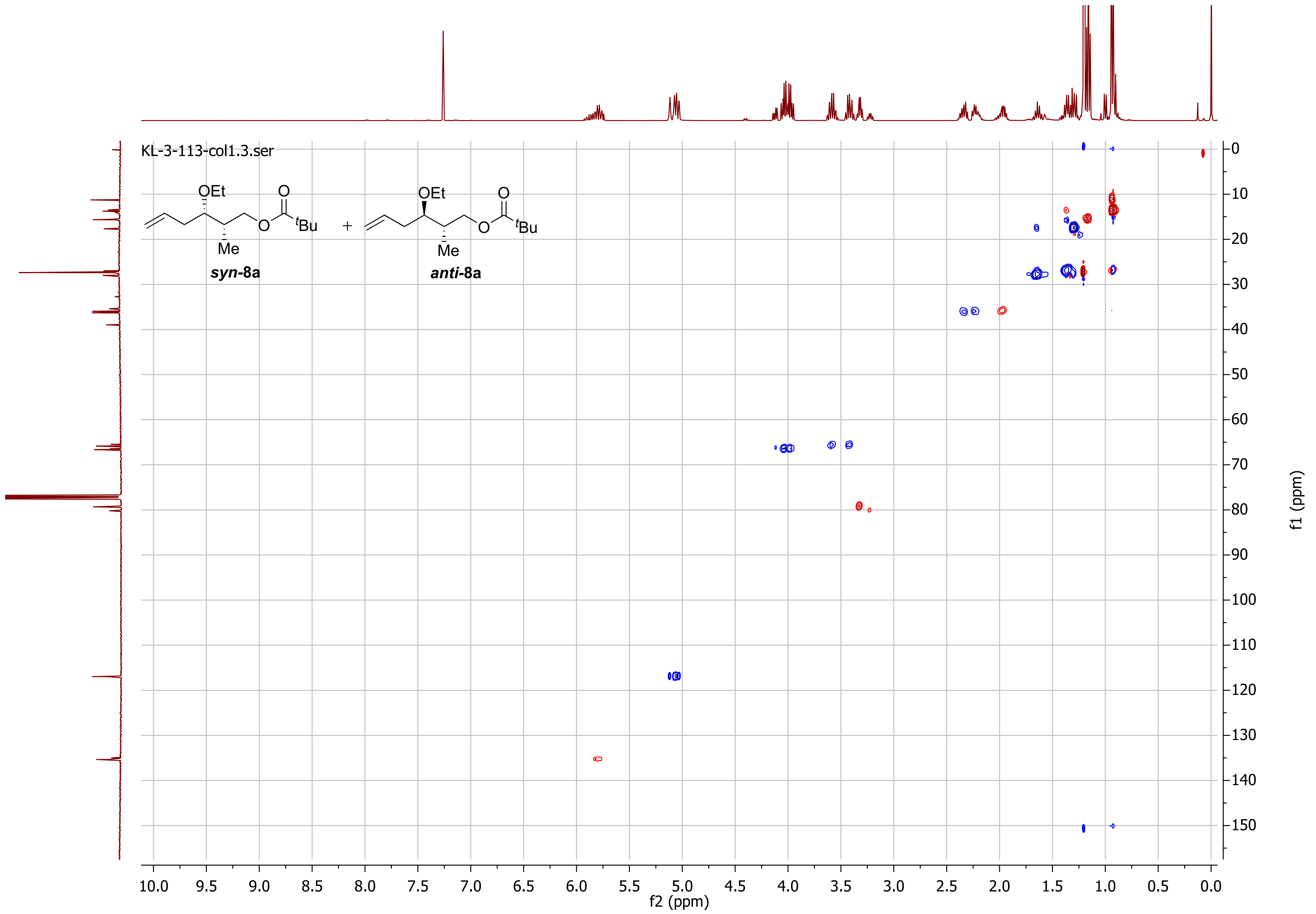
KL-3-113-col1.3.ser
OEt
O
tBu
Me
syn-8a
+
anti-8a
f2 (ppm)
f1 (ppm)

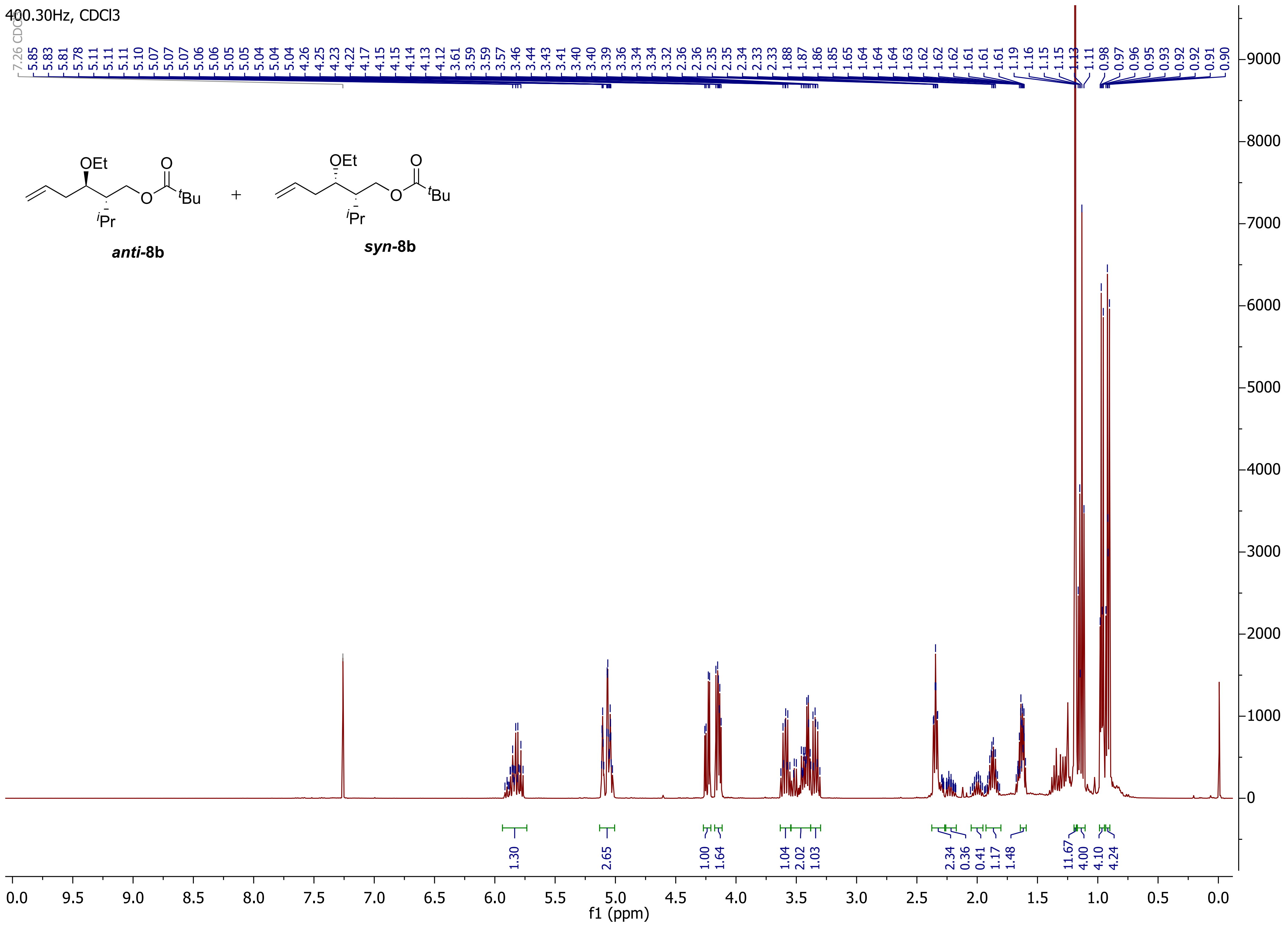
400.30Hz, CDCl3
7.26 CDCl3
5.85 5.83 5.81 5.78 5.11 5.11 5.11 5.10 5.07 5.07 5.07 5.06 5.05 5.05 5.04 5.04 5.04 4.26 4.25 4.23 4.22 4.17 4.15 4.15 4.14 4.13 4.12 3.61 3.59 3.59 3.57 3.46 3.44 3.43 3.41 3.40 3.40 3.39 3.36 3.34 3.34 3.32 2.36 2.36 2.35 2.35 2.34 2.33 2.33 1.88 1.87 1.86 1.85 1.65 1.64 1.64 1.63 1.62 1.62 1.62 1.61 1.61 1.61 1.19 1.16 1.15 1.15 1.13 1.11 0.98 0.97 0.96 0.95 0.93 0.92 0.91 0.90
OEt
O
tBu
iPr
anti-8b
syn-8b
1.30 2.65 1.00 1.64 1.04 2.02 1.03 2.34 0.36 0.41 1.17 1.48 11.67 4.00 4.10 4.24
f1 (ppm)

100.67Hz, CDCl3

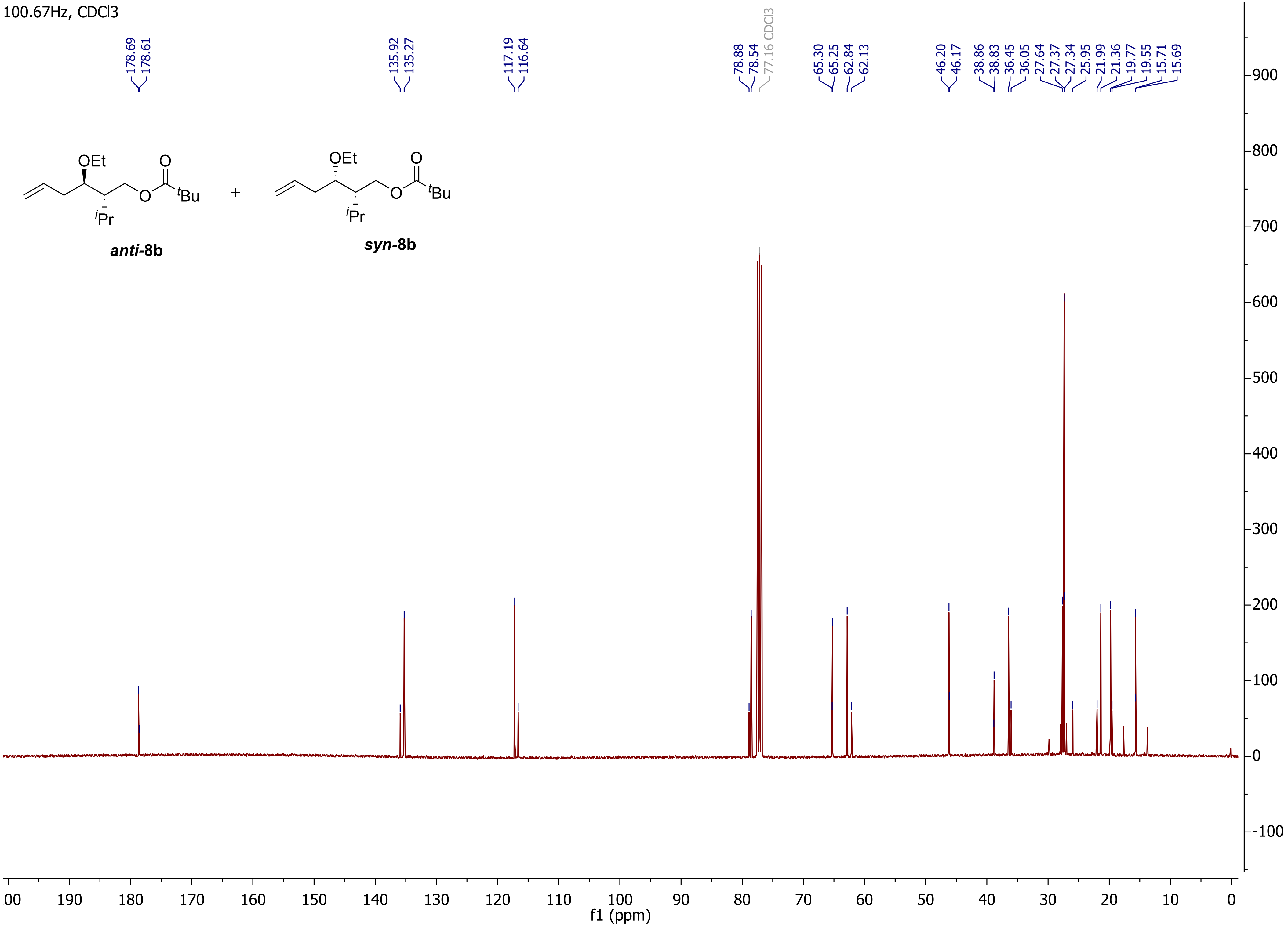

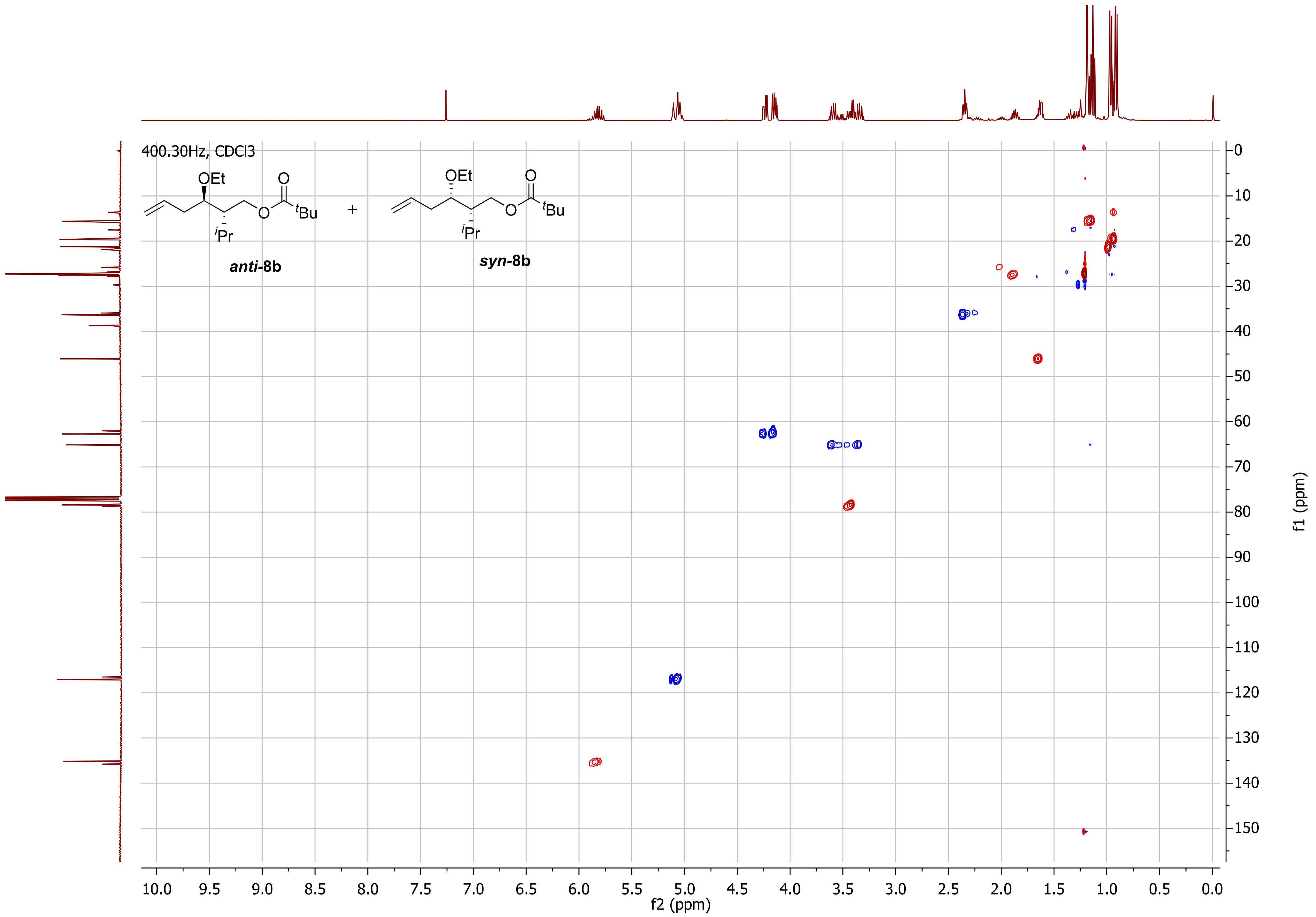

400.30Hz, CDCl3
OEt
O
iPr
O
tBu
+
anti-8b
syn-8b
f2 (ppm)
f1 (ppm)

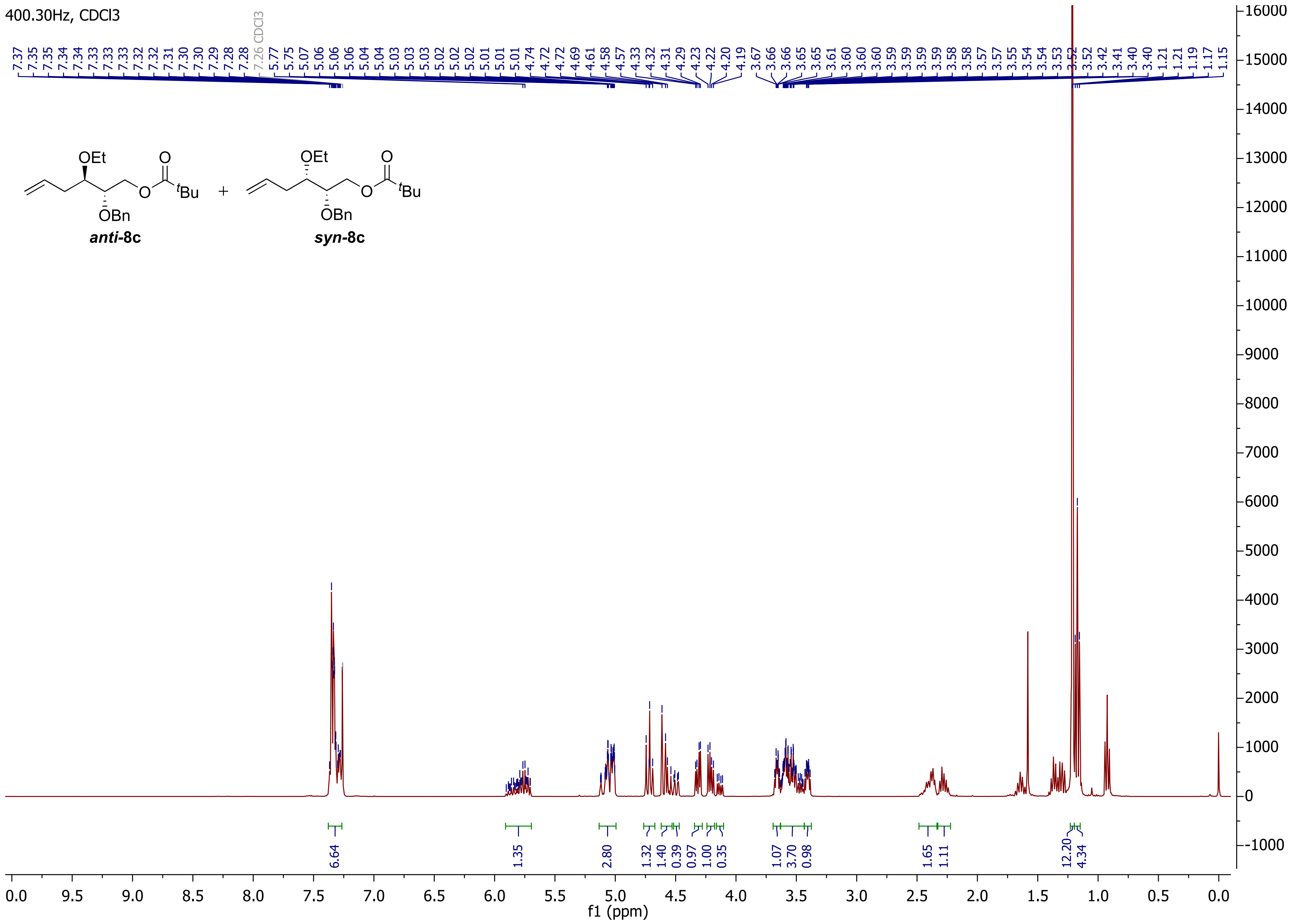
400.30Hz, CDCl3
anti-8c
syn-8c
OEt
OBn
tBu
f1 (ppm)

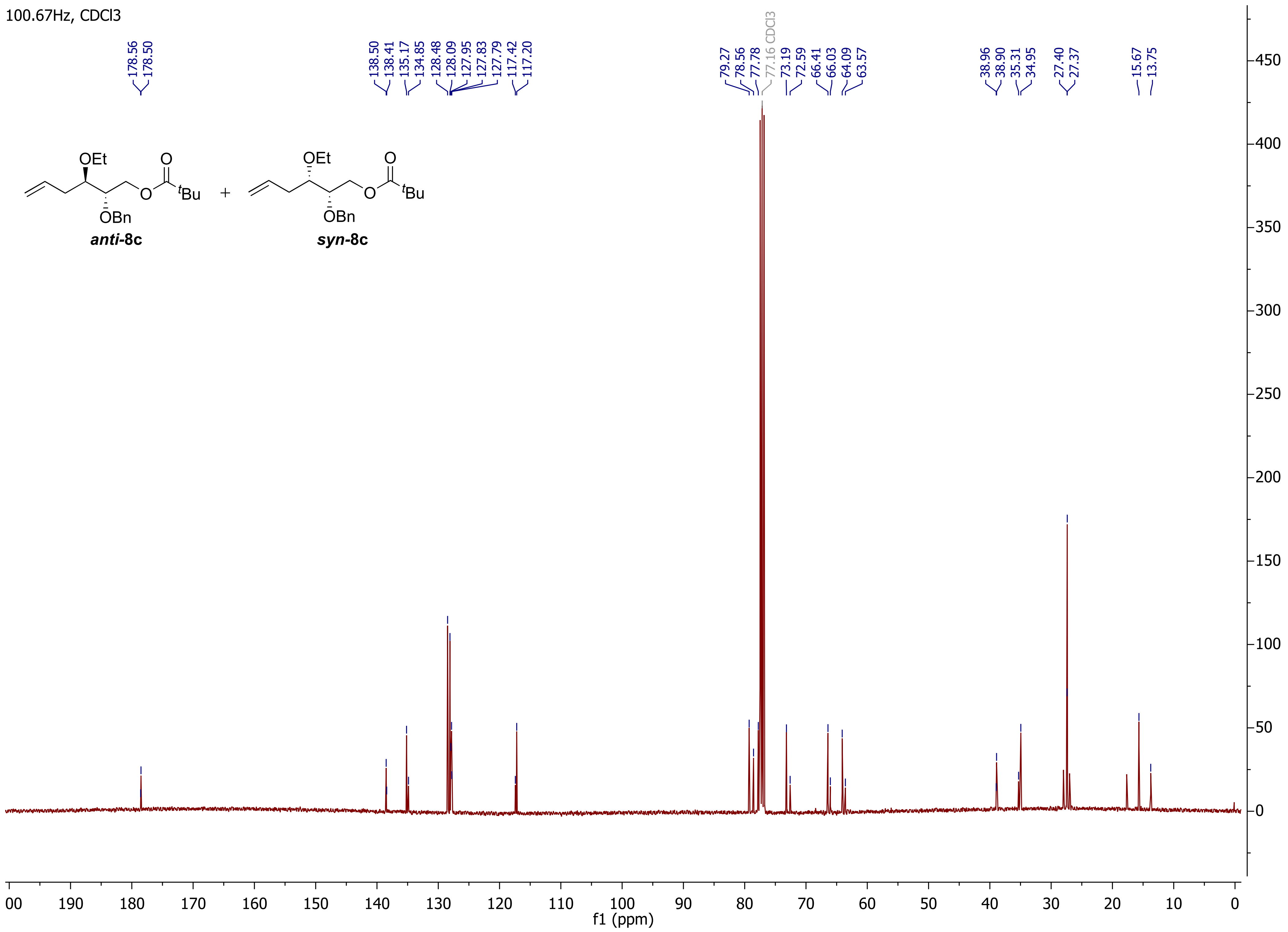

100.67Hz, CDCl3
178.56
178.50
138.50
138.41
135.17
134.85
128.48
128.09
127.95
127.83
127.79
117.42
117.20
79.27
78.56
77.78
77.16 CDCl3
73.19
72.59
66.41
66.03
64.09
63.57
38.96
38.90
35.31
34.95
27.40
27.37
15.67
13.75
OEt
O
O
tBu
OBn
anti-8c
+
syn-8c
f1 (ppm)

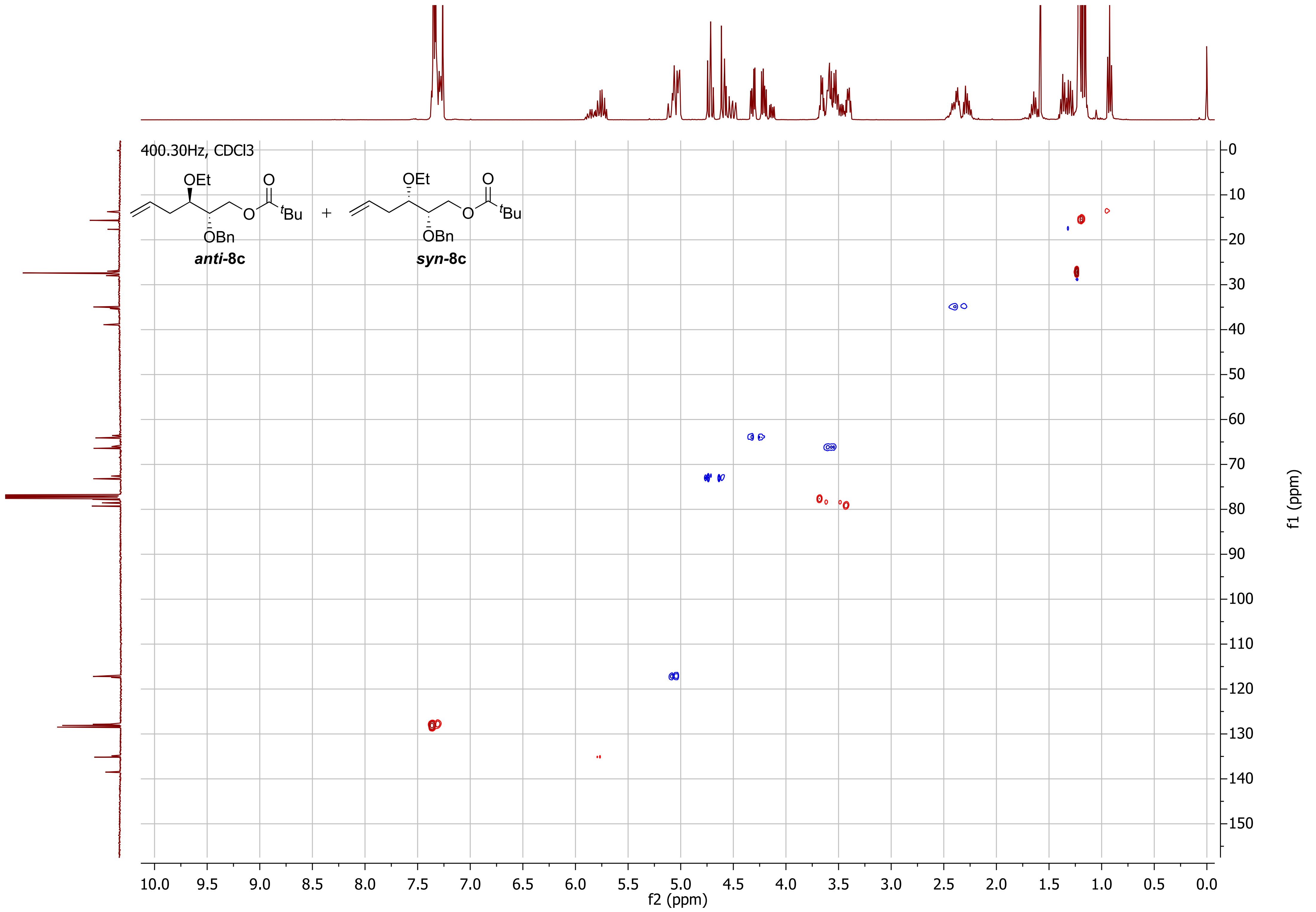
400.30Hz, CDCl3
OEt
O
^{t}Bu
OBn
anti-8c
+
syn-8c
f2 (ppm)
f1 (ppm)

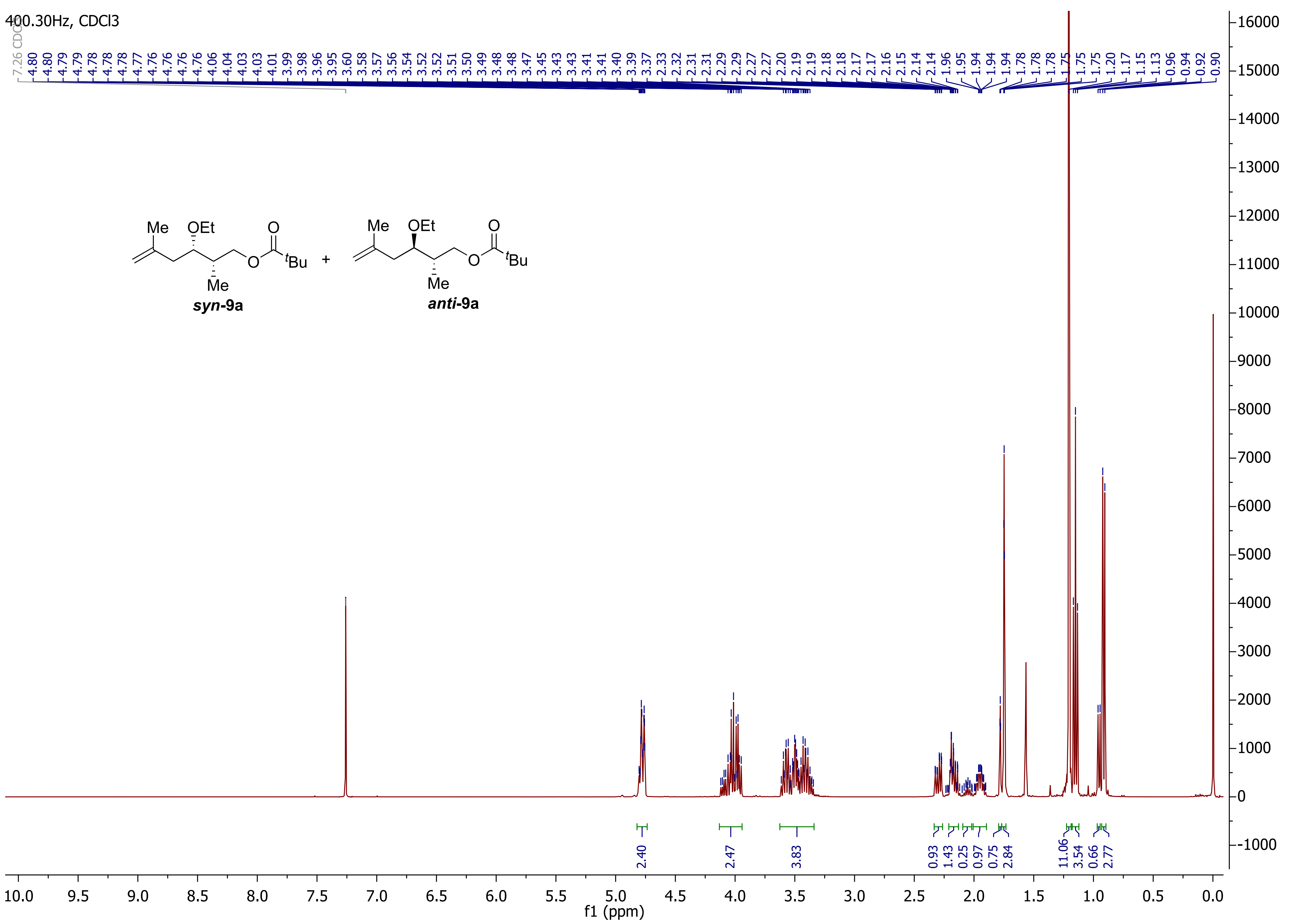
400.30Hz, CDCl3
Me
OEt
O
tBu
+
Me
syn-9a
anti-9a
f1 (ppm)
2.40
2.47
3.83
0.93
1.43
0.25
0.97
0.75
2.84
11.06
3.54
0.66
2.77

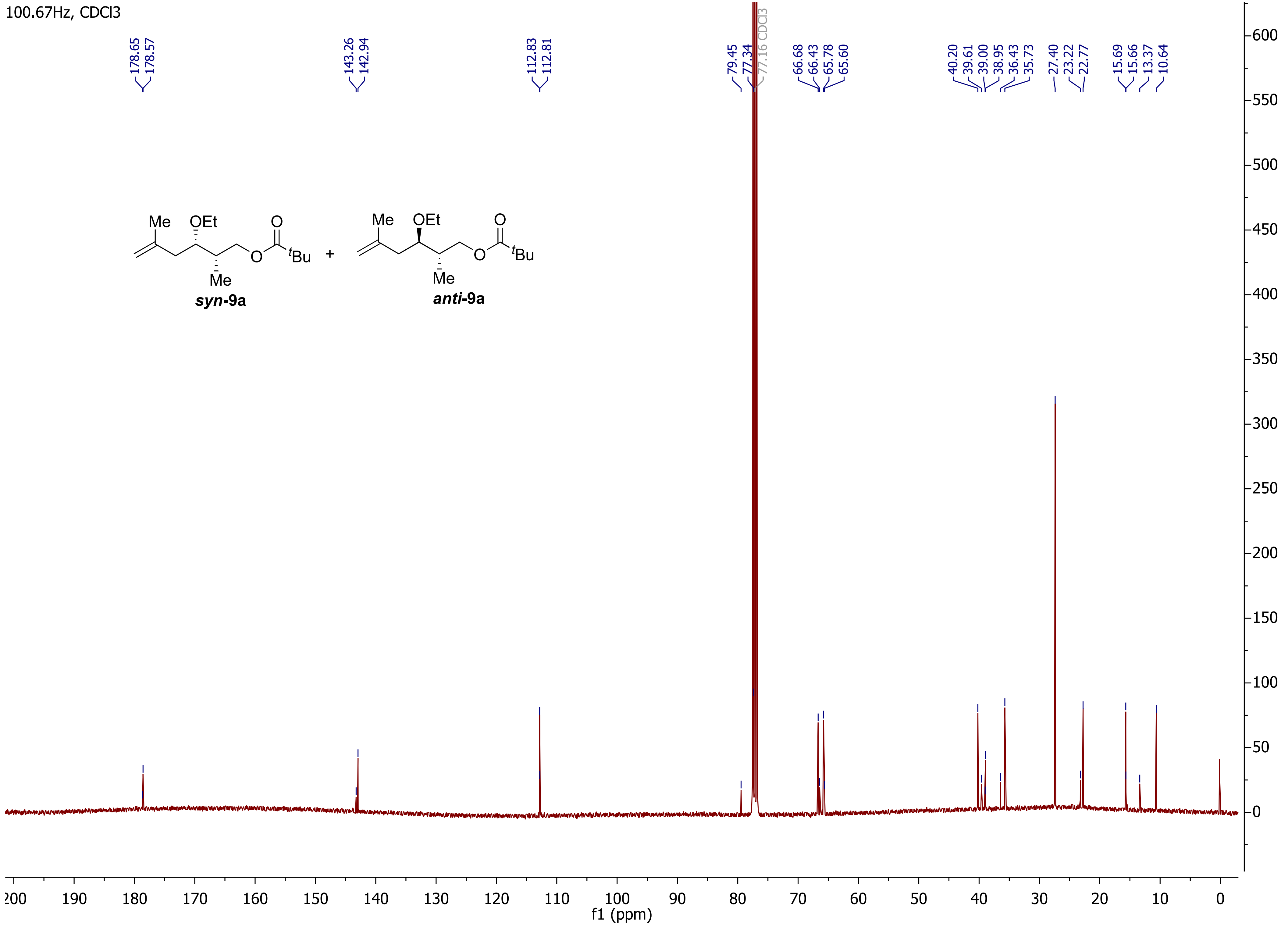
100.67Hz, CDCl3
178.65
178.57
143.26
142.94
112.83
112.81
79.45
77.34
77.16 CDCl3
66.68
66.43
65.78
65.60
40.20
39.61
39.00
38.95
36.43
35.73
27.40
23.22
22.77
15.69
15.66
13.37
10.64
Me
OEt
O
O
tBu
Me
syn-9a
+
anti-9a
f1 (ppm)

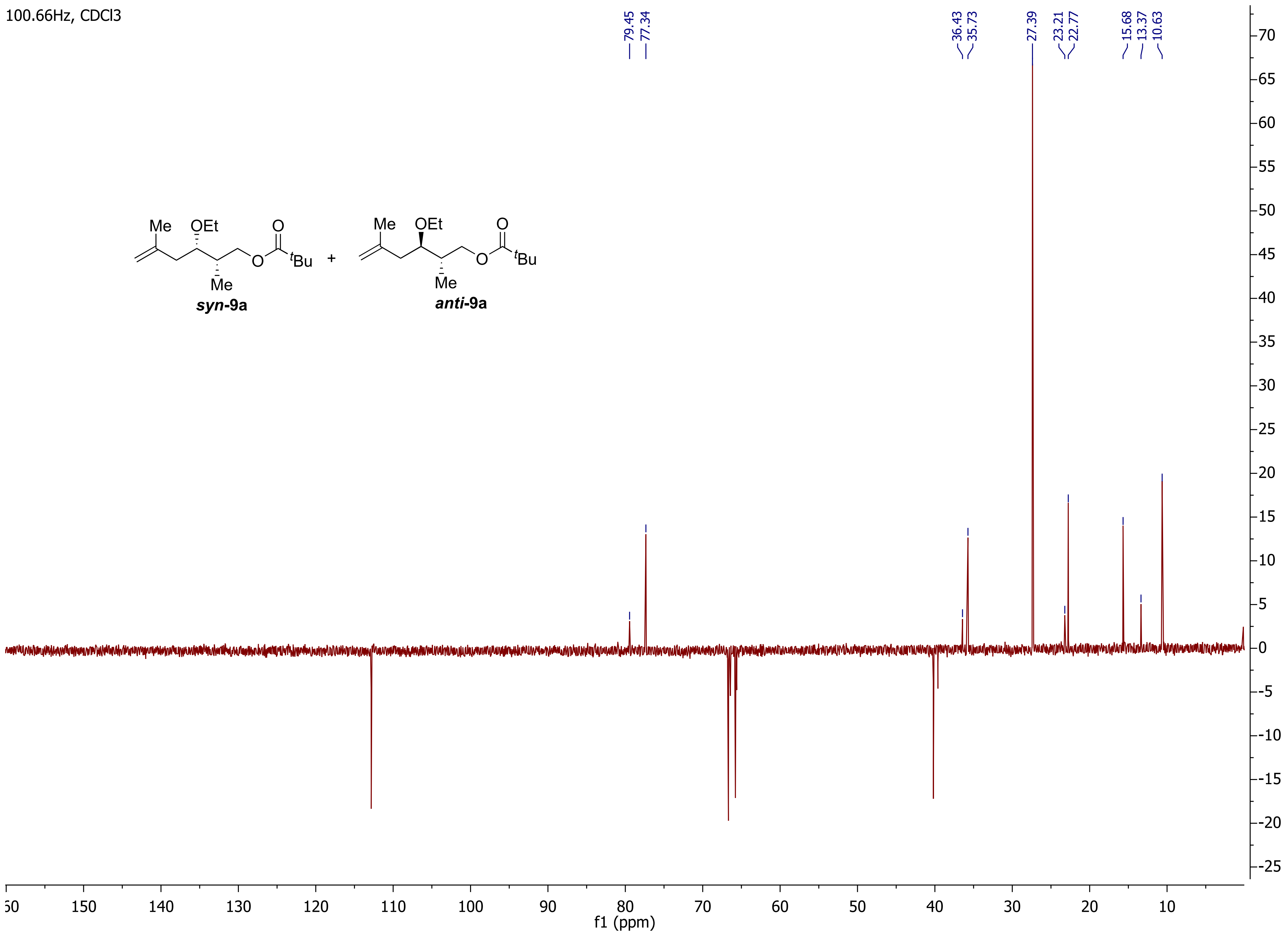
100.66Hz, CDCl3
79.45
77.34
36.43
35.73
27.39
23.21
22.77
15.68
13.37
10.63
Me
OEt
O
tBu
Me
syn-9a
+
Me
OEt
O
tBu
Me
anti-9a
f1 (ppm)

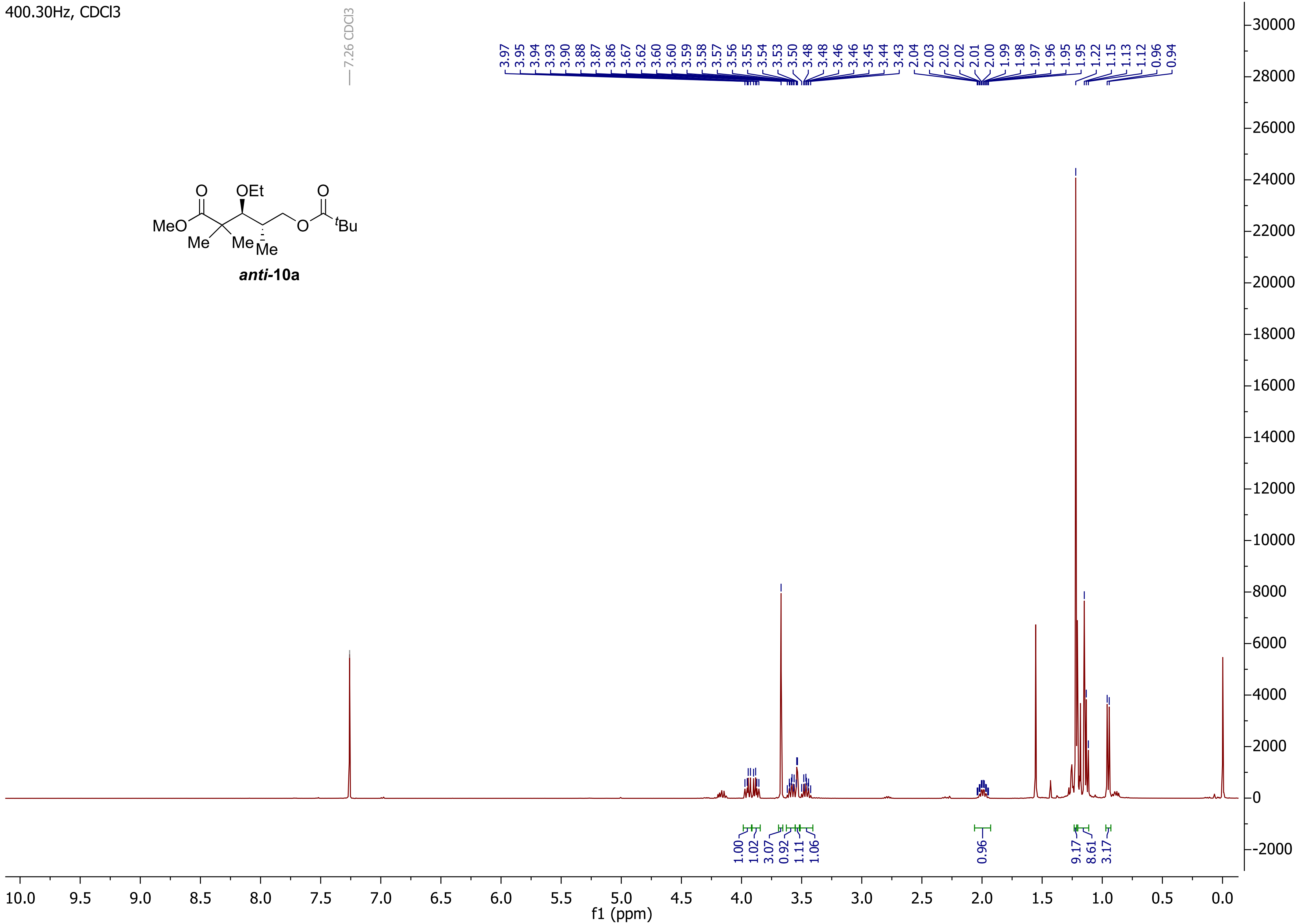
400.30Hz, CDCl3
7.26 CDCl3
3.97 3.95 3.94 3.93 3.90 3.88 3.87 3.86 3.67 3.62 3.60 3.60 3.59 3.58 3.57 3.56 3.55 3.54 3.53 3.50 3.48 3.48 3.46 3.46 3.45 3.44 3.43 2.04 2.03 2.02 2.02 2.01 2.00 1.99 1.98 1.97 1.96 1.95 1.95 1.22 1.15 1.13 1.12 0.96 0.94
O
OEt
O
MeO
O
tBu
Me
Me
Me
anti-10a
1.00 1.02 3.07 0.92 1.11 1.06
0.96
9.17 8.61 3.17
f1 (ppm)

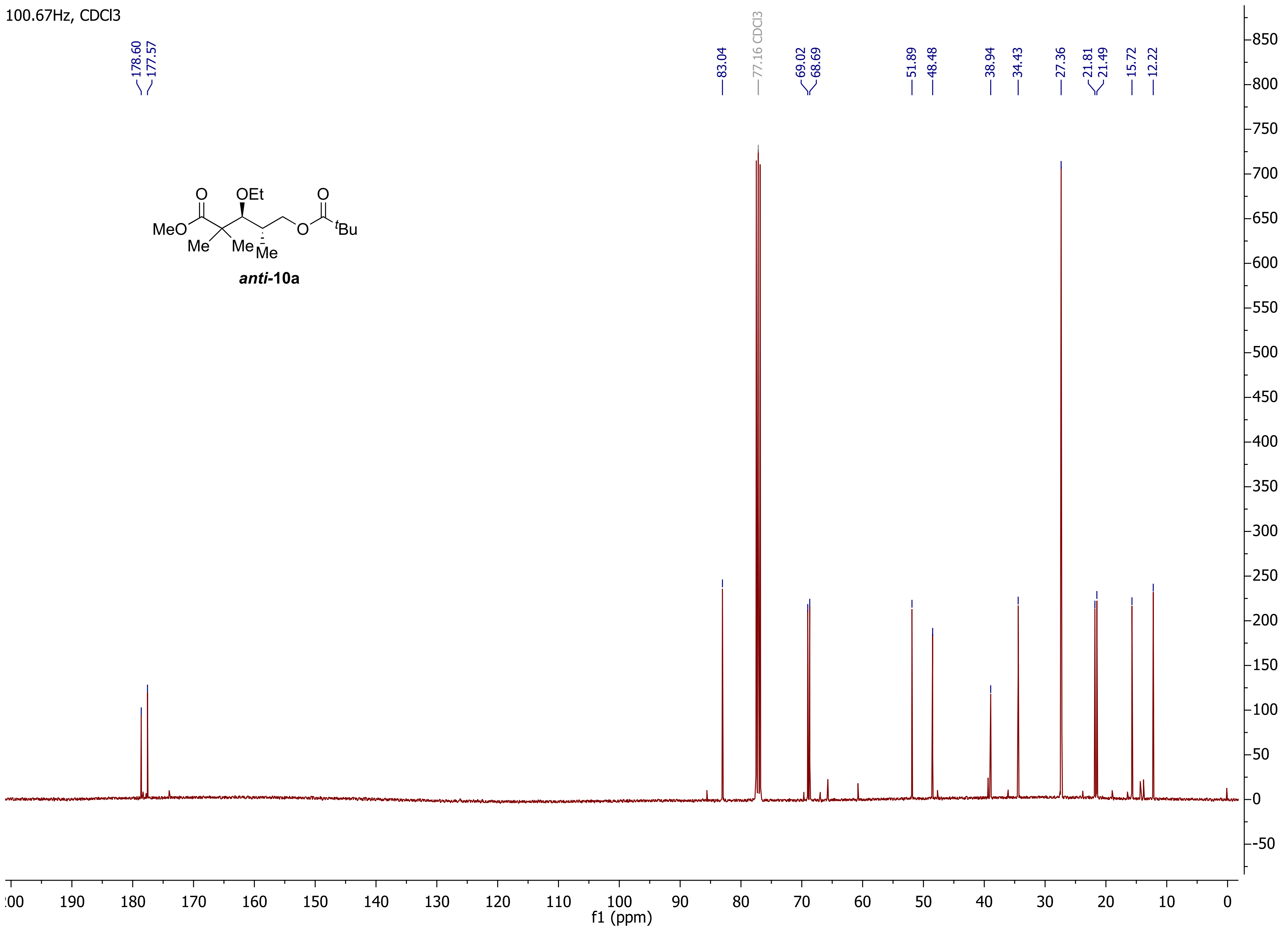
100.67Hz, CDCl3
178.60
177.57
83.04
77.16 CDCl3
69.02
68.69
51.89
48.48
38.94
34.43
27.36
21.81
21.49
15.72
12.22
MeO
O
OEt
O
Me
Me
Me
O
tBu
anti-10a
f1 (ppm)

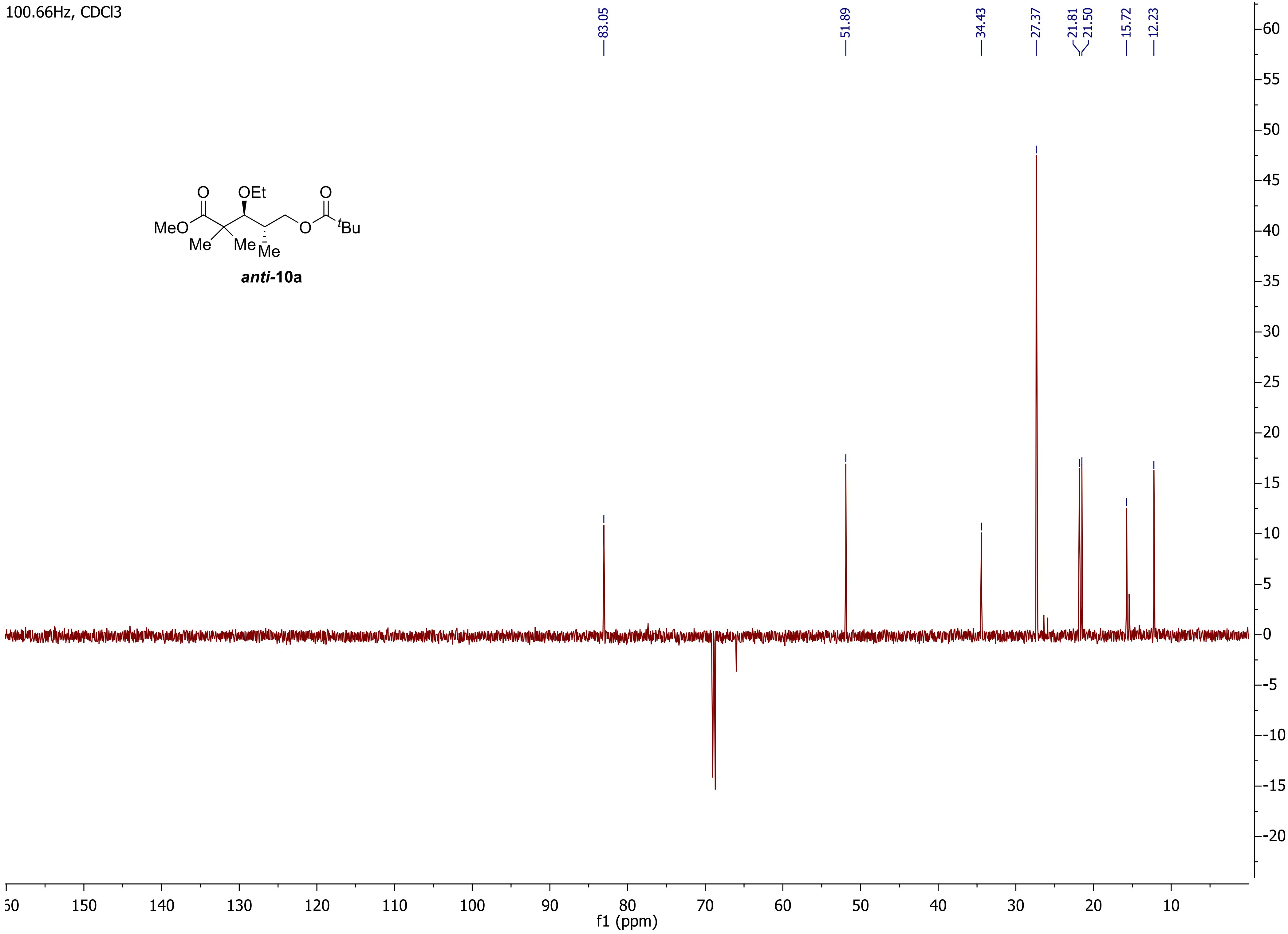
100.66Hz, CDCl3
83.05
51.89
34.43
27.37
21.81
21.50
15.72
12.23
MeO
O
OEt
O
tBu
Me
Me
Me
anti-10a
f1 (ppm)

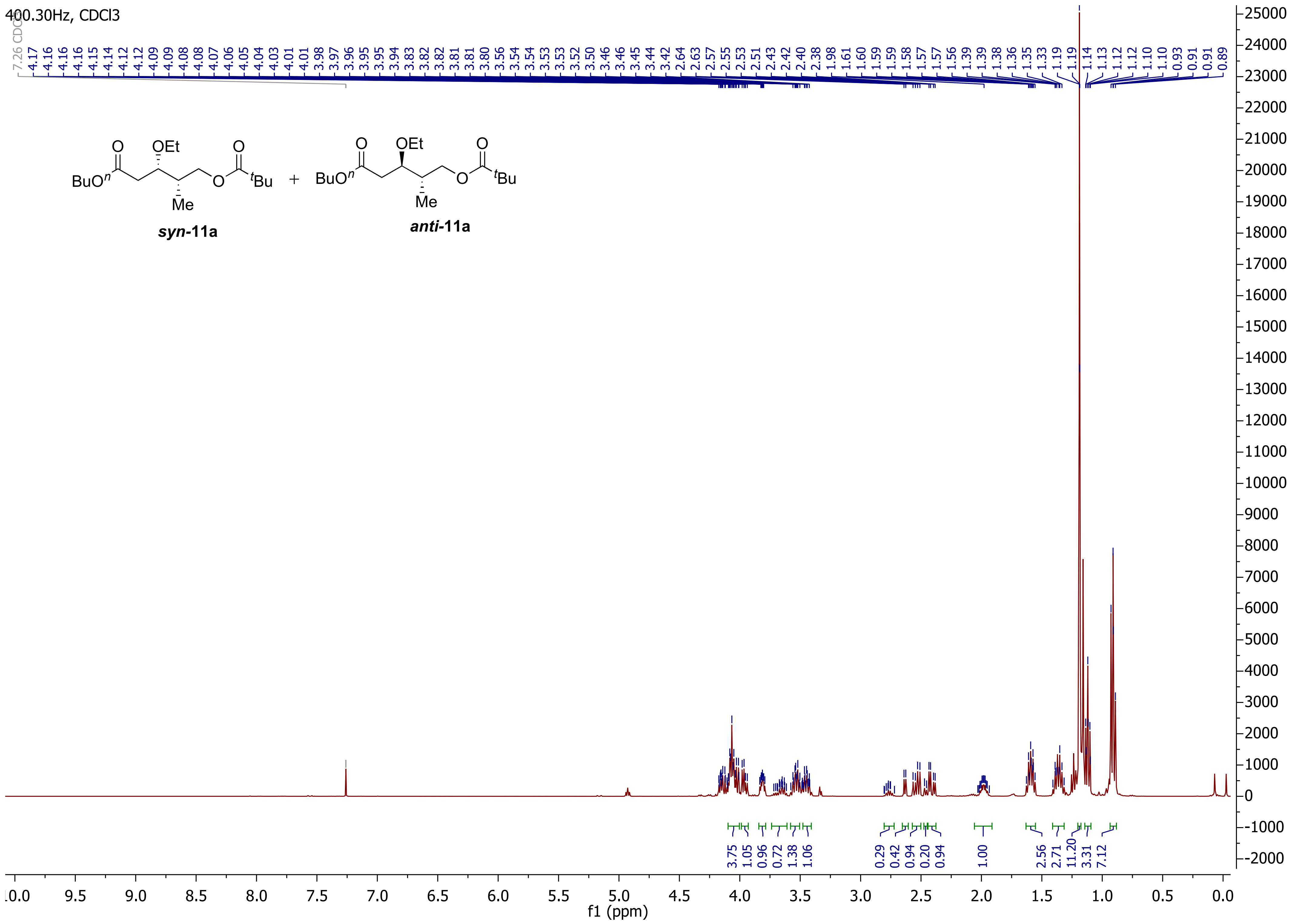

400.30Hz, CDCl3
7.26 CDCl3
4.17 4.16 4.16 4.15 4.14 4.12 4.12 4.09 4.09 4.08 4.08 4.07 4.06 4.05 4.04 4.03 4.01 4.01 3.98 3.97 3.96 3.95 3.95 3.94 3.83 3.82 3.82 3.81 3.81 3.80 3.56 3.54 3.54 3.53 3.53 3.52 3.50 3.46 3.46 3.45 3.44 3.42 2.64 2.63 2.57 2.55 2.53 2.51 2.43 2.42 2.40 2.38 1.98 1.61 1.60 1.59 1.59 1.58 1.57 1.57 1.56 1.39 1.39 1.38 1.36 1.35 1.33 1.19 1.19 1.14 1.13 1.12 1.12 1.10 1.10 0.93 0.91 0.89
BuO^n
OEt
Me
^{t}Bu
syn-**11a**
anti-**11a**
3.75 1.05 0.96 0.72 1.38 1.06 0.29 0.42 0.94 0.20 0.94 1.00 2.56 2.71 11.20 3.31 7.12
f1 (ppm)

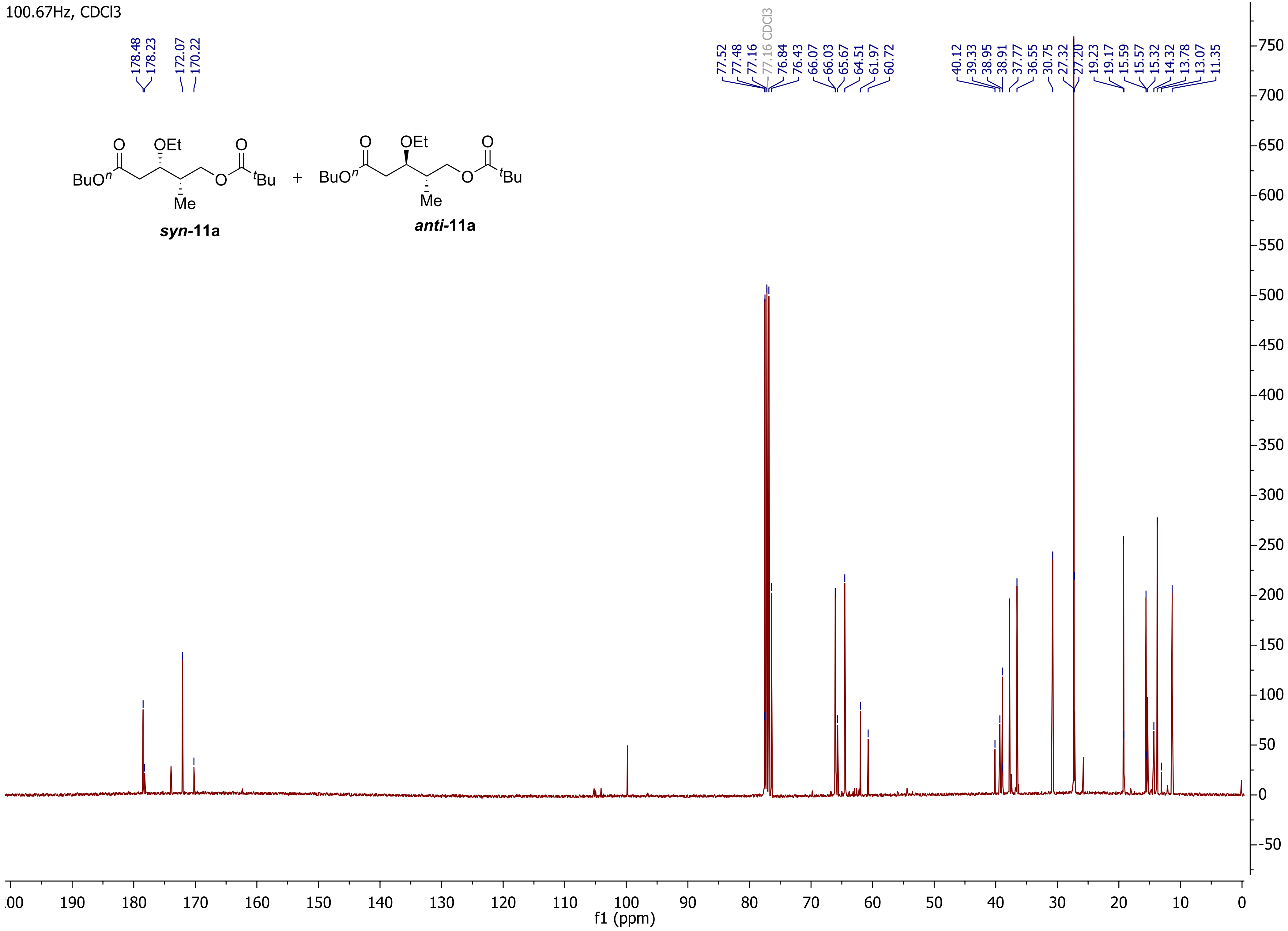

100.67Hz, CDCl3
178.48
178.23
172.07
170.22
77.52
77.48
77.16
77.16 CDCl3
76.84
76.43
66.07
66.03
65.67
64.51
61.97
60.72
40.12
39.33
38.95
38.91
37.77
36.55
30.75
27.32
27.20
19.23
19.17
15.59
15.57
15.32
14.32
13.78
13.07
11.35
OEt
Me
BuO^n
^{t}Bu
syn-11a
anti-11a
f1 (ppm)

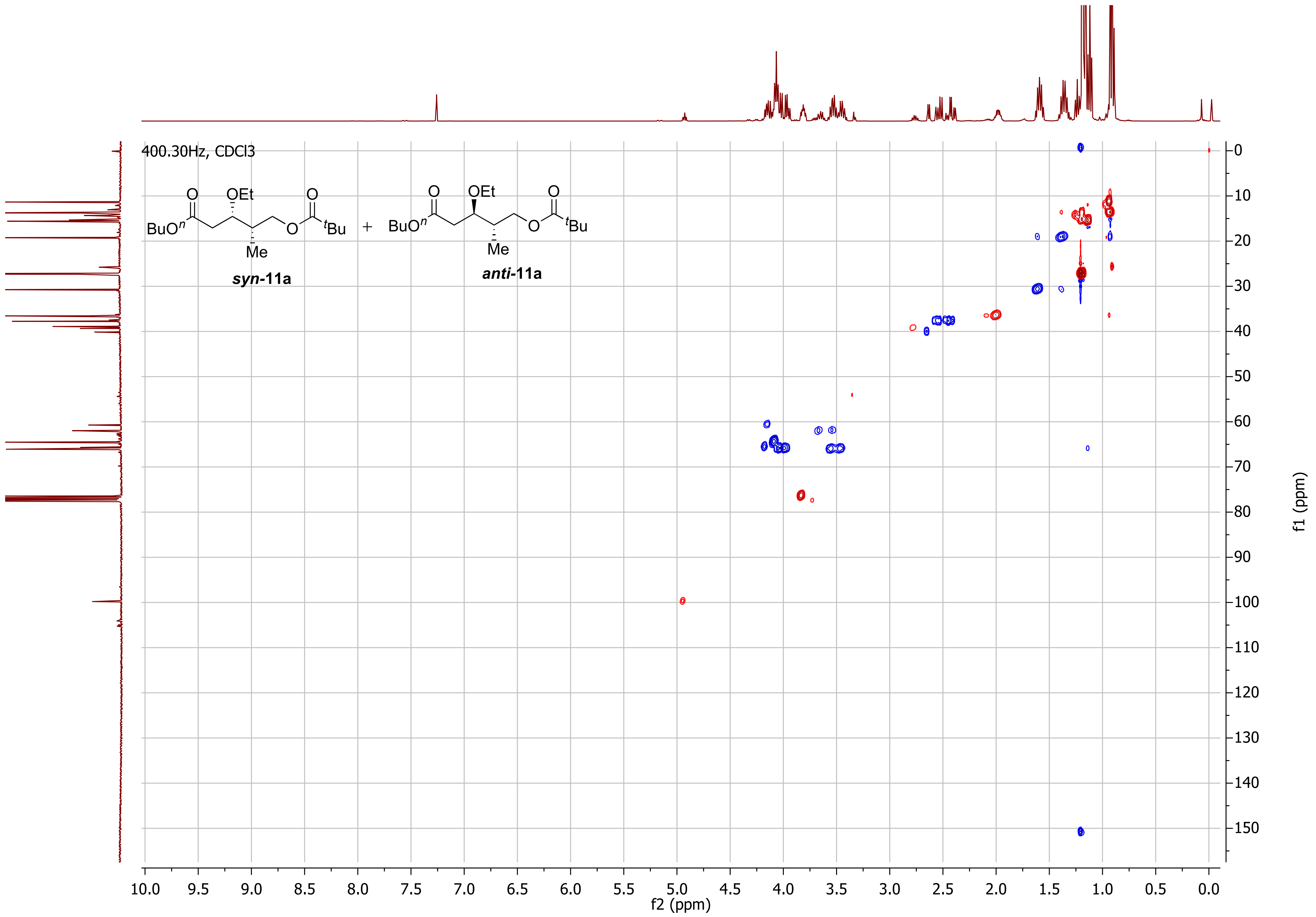
400.30Hz, CDCl3
BuO^n
OEt
Me
O
^{t}Bu
syn-11a
anti-11a
f2 (ppm)
f1 (ppm)

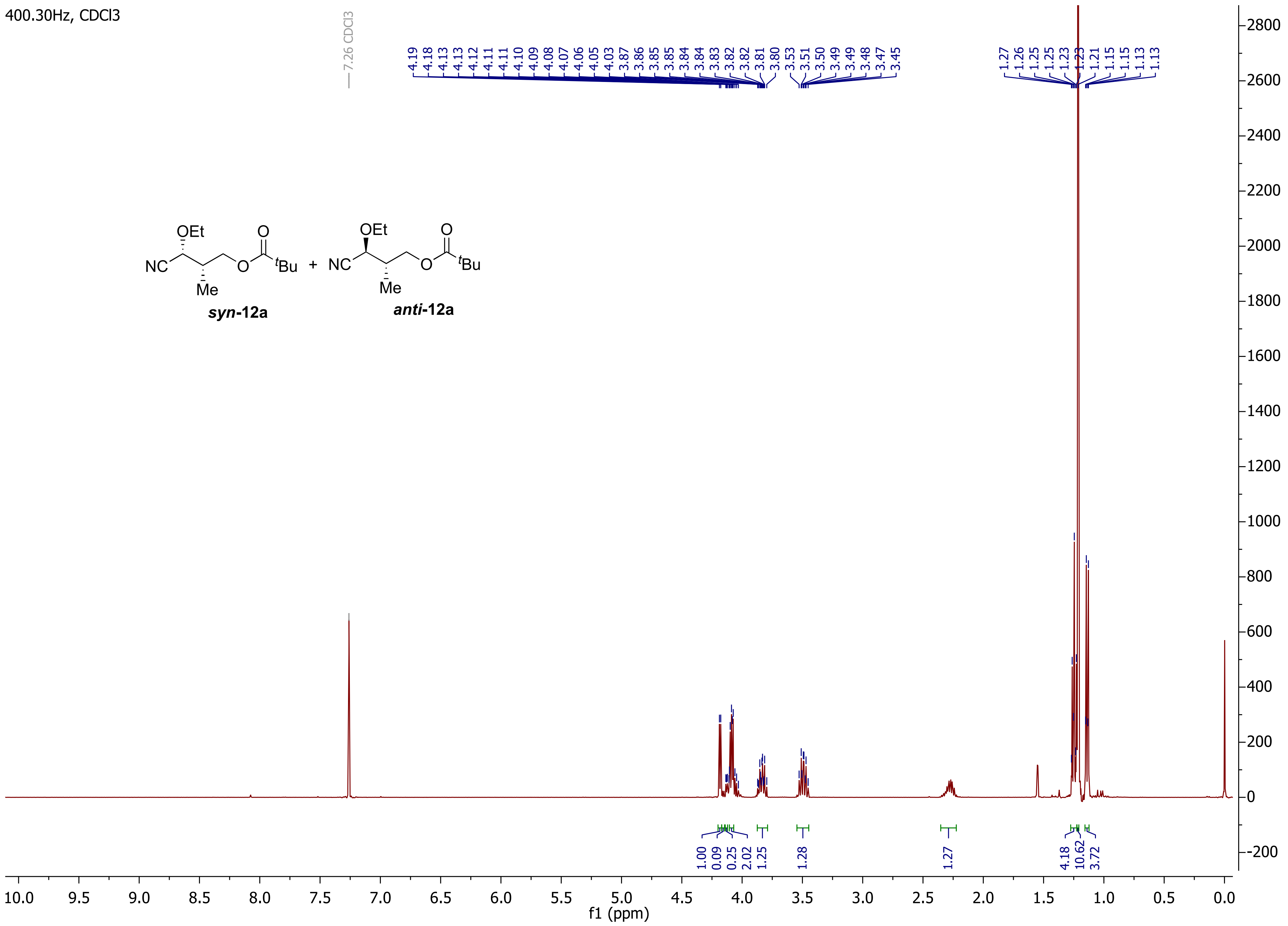

400.30Hz, CDCl3
7.26 CDCl3
4.19
4.18
4.13
4.13
4.12
4.11
4.11
4.10
4.09
4.08
4.07
4.06
4.05
4.03
3.87
3.86
3.85
3.85
3.84
3.84
3.83
3.82
3.82
3.81
3.80
3.53
3.51
3.50
3.49
3.49
3.48
3.47
3.45
1.27
1.26
1.25
1.25
1.23
1.23
1.21
1.15
1.15
1.13
1.13
OEt
NC
O
O
tBu
Me
syn-12a
anti-12a
1.00
0.09
0.25
2.02
1.25
1.28
1.27
4.18
10.62
3.72
f1 (ppm)

100.67Hz, CDCl3

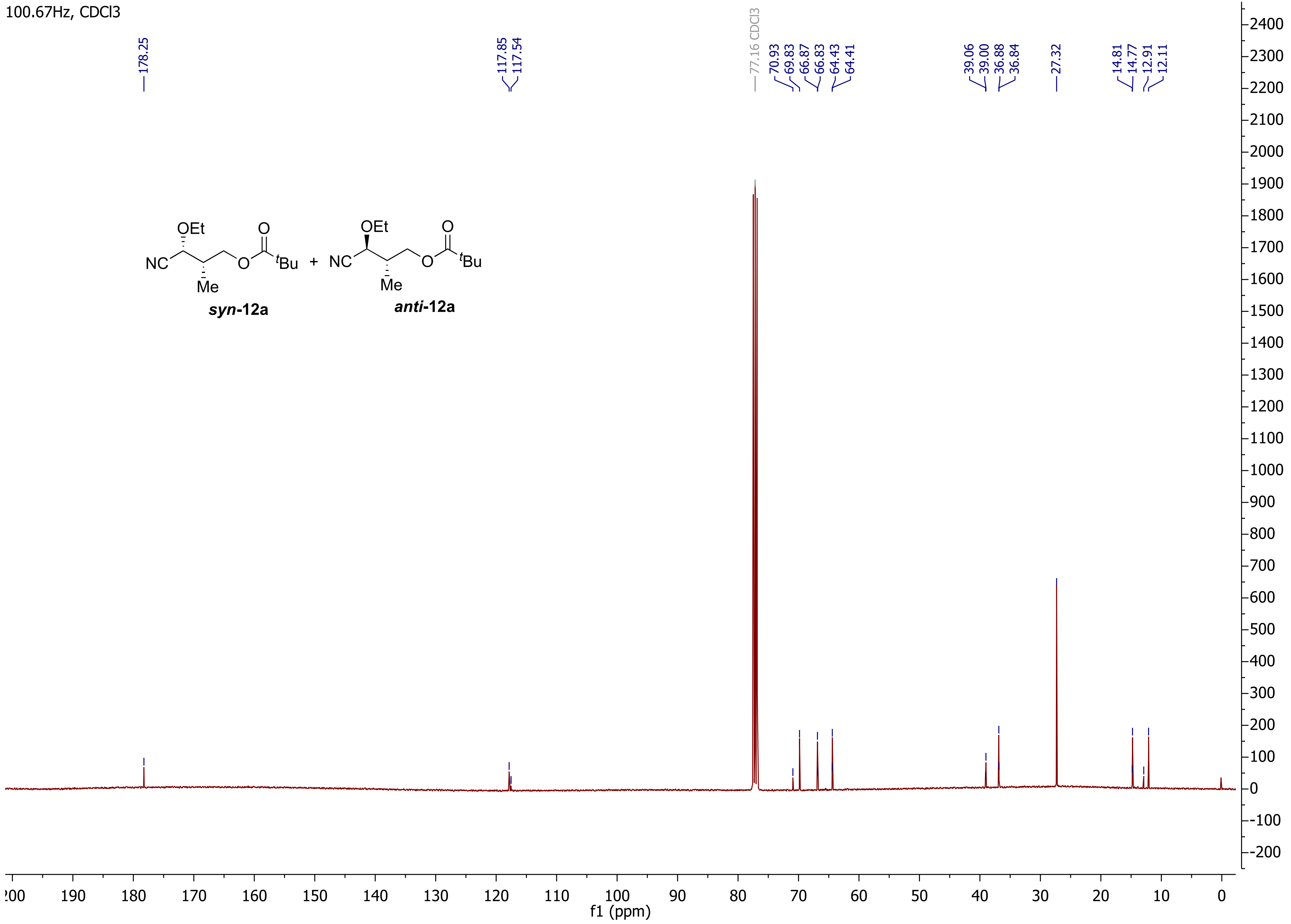

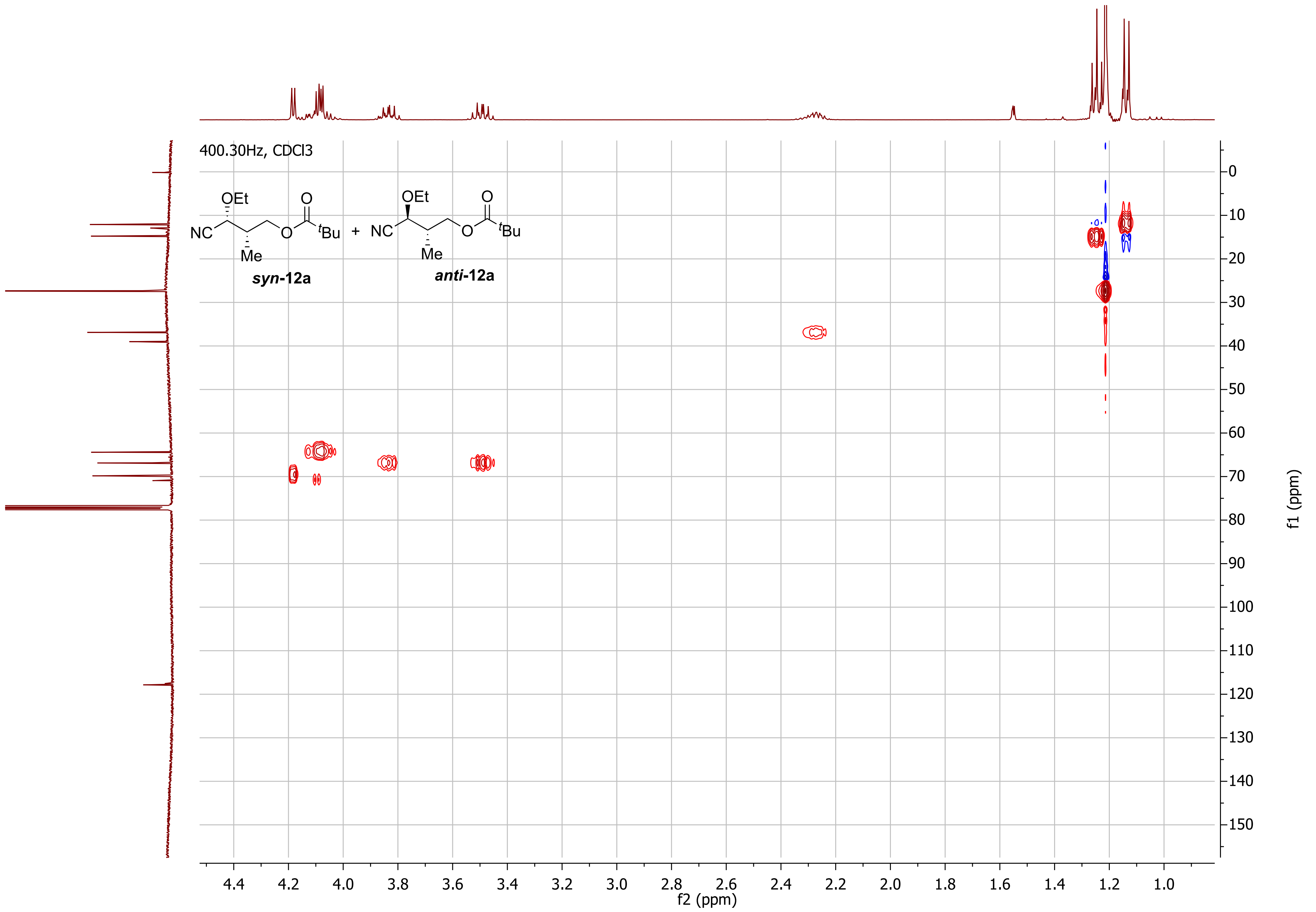
400.30Hz, CDCl3
OEt
O
NC
O
tBu
Me
syn-12a
+
anti-12a
f2 (ppm)
f1 (ppm)

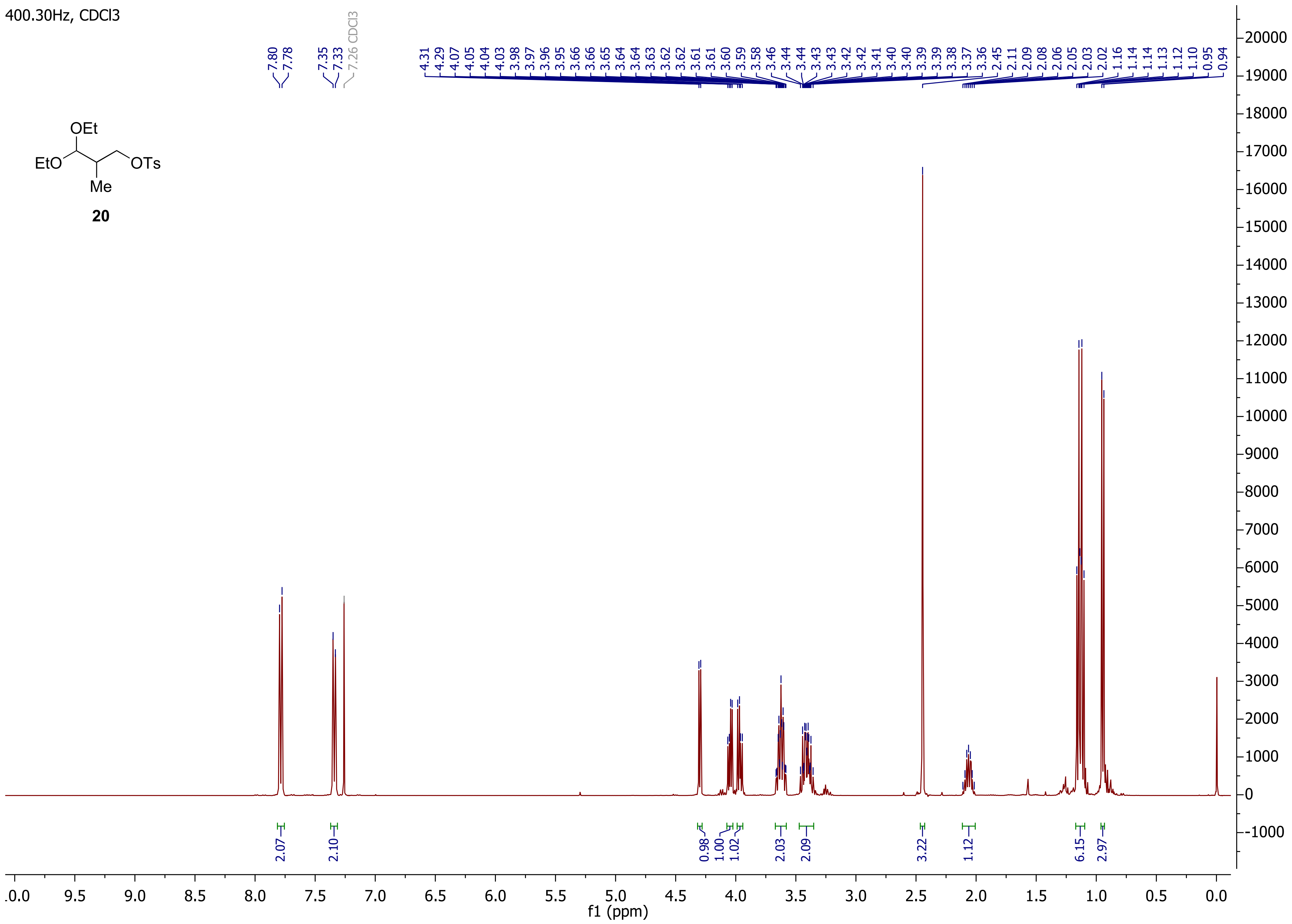
400.30Hz, CDCl3
OEt
EtO
OTs
Me
20
7.80
7.78
7.35
7.33
7.26 CDCl3
4.31
4.29
4.07
4.05
4.04
4.03
3.98
3.97
3.96
3.95
3.66
3.66
3.65
3.64
3.64
3.63
3.62
3.62
3.61
3.61
3.60
3.59
3.58
3.46
3.44
3.44
3.43
3.43
3.42
3.42
3.41
3.40
3.40
3.39
3.39
3.38
3.37
3.36
2.45
2.11
2.09
2.08
2.06
2.05
2.03
2.02
1.16
1.14
1.14
1.13
1.12
1.10
0.95
0.94
2.07
2.10
0.98
1.00
1.02
2.03
2.09
3.22
1.12
6.15
2.97
f1 (ppm)

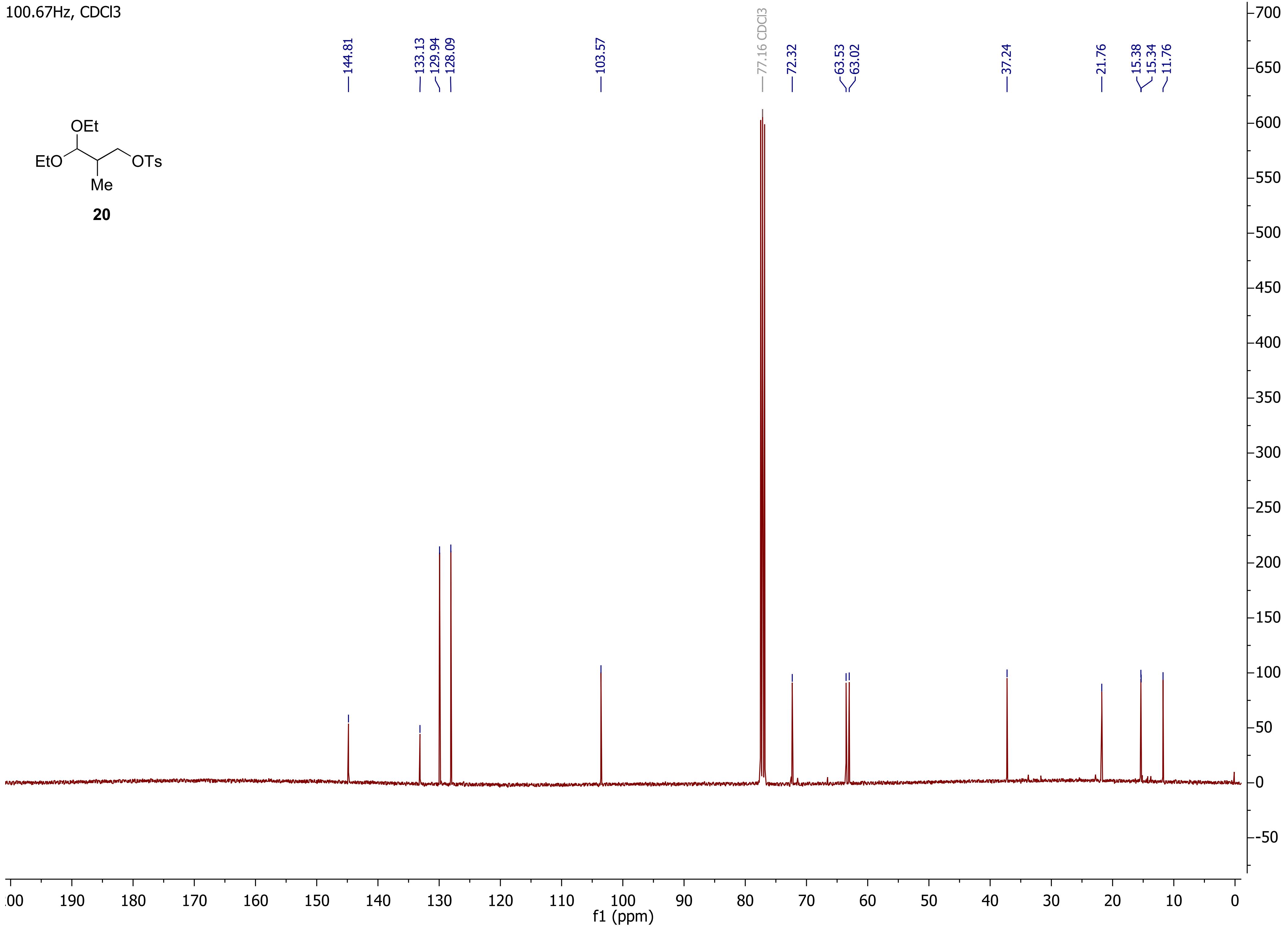

100.67Hz, CDCl3
144.81
133.13
129.94
128.09
103.57
77.16 CDCl3
72.32
63.53
63.02
37.24
21.76
15.38
15.34
11.76
OEt
EtO
OTs
Me
20
f1 (ppm)

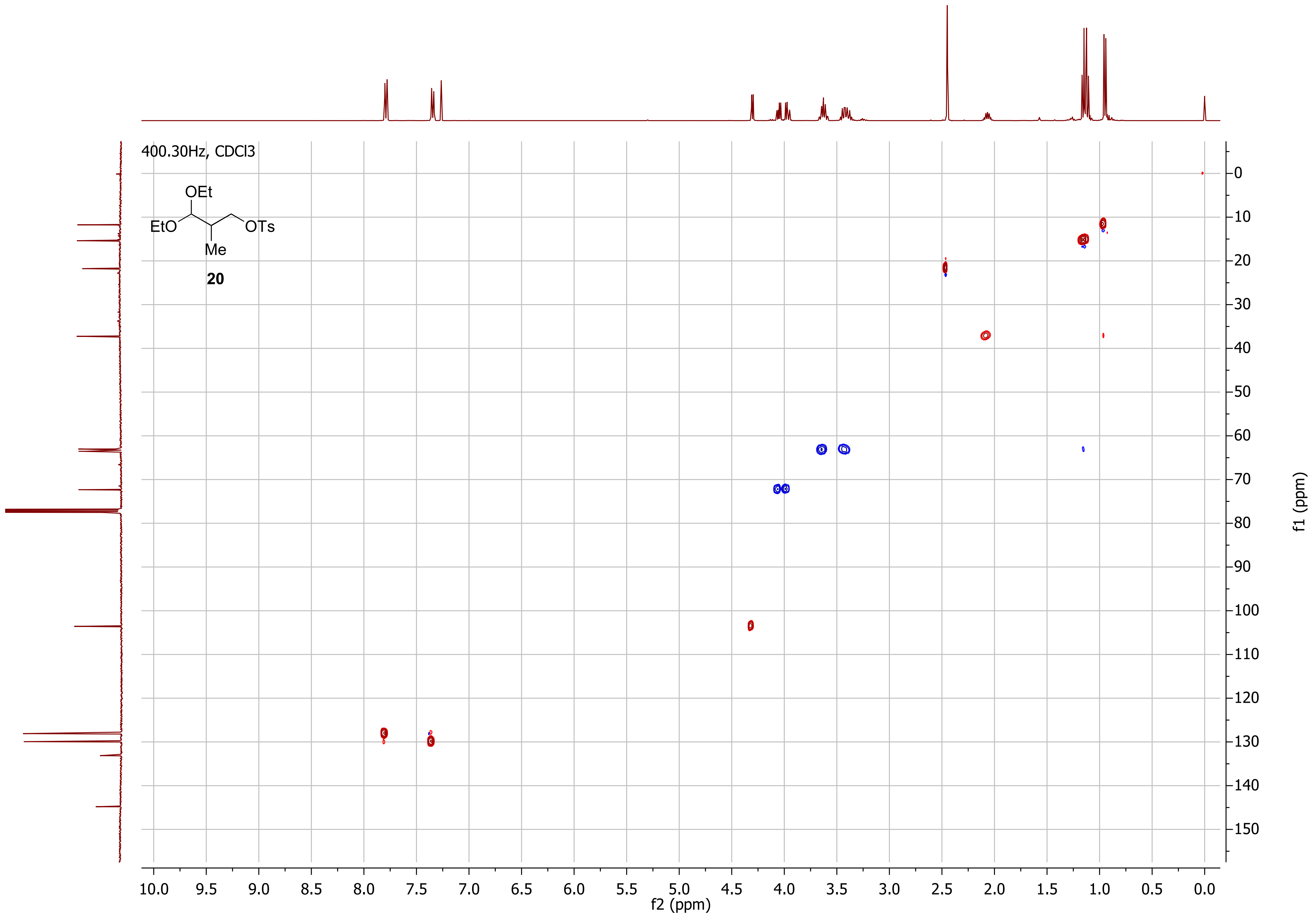
400.30Hz, CDCl3
OEt
EtO
OTs
Me
20
f2 (ppm)
f1 (ppm)

400.30Hz, CDCl3

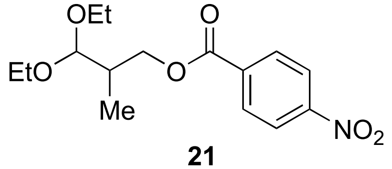

21

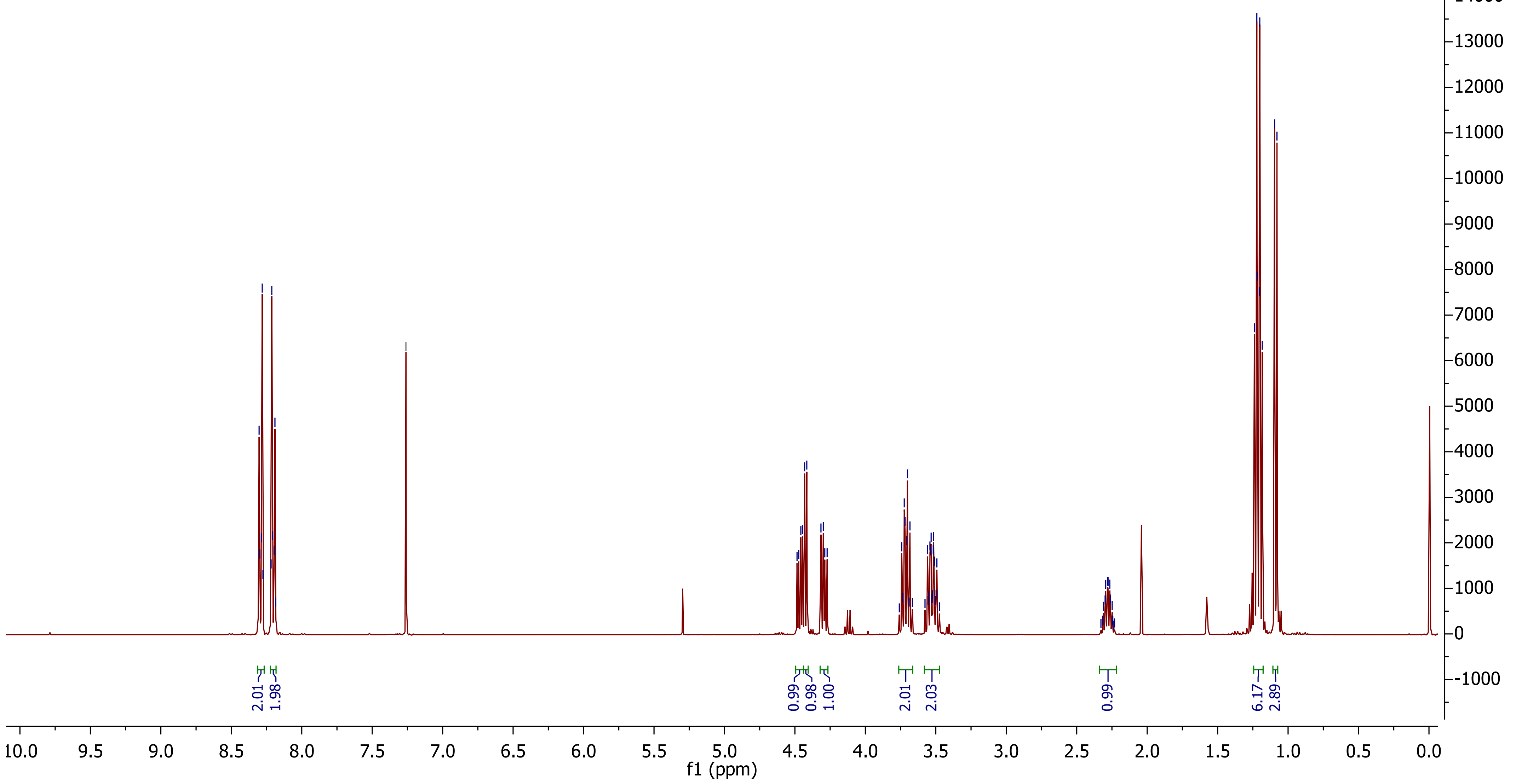

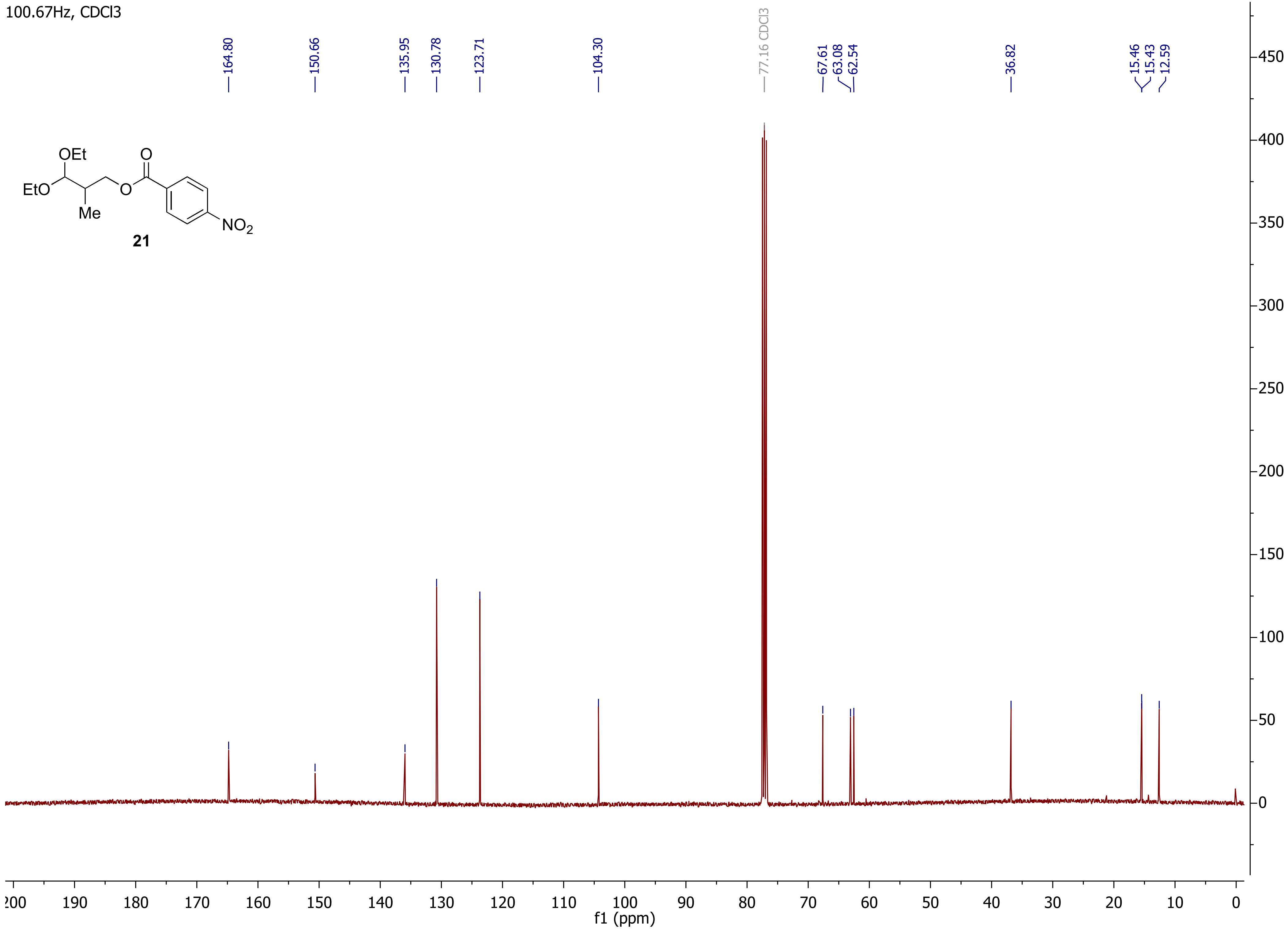
100.67Hz, CDCl3
164.80
150.66
135.95
130.78
123.71
104.30
77.16 CDCl3
67.61
63.08
62.54
36.82
15.46
15.43
12.59
OEt
EtO
O
Me
NO2
21
f1 (ppm)

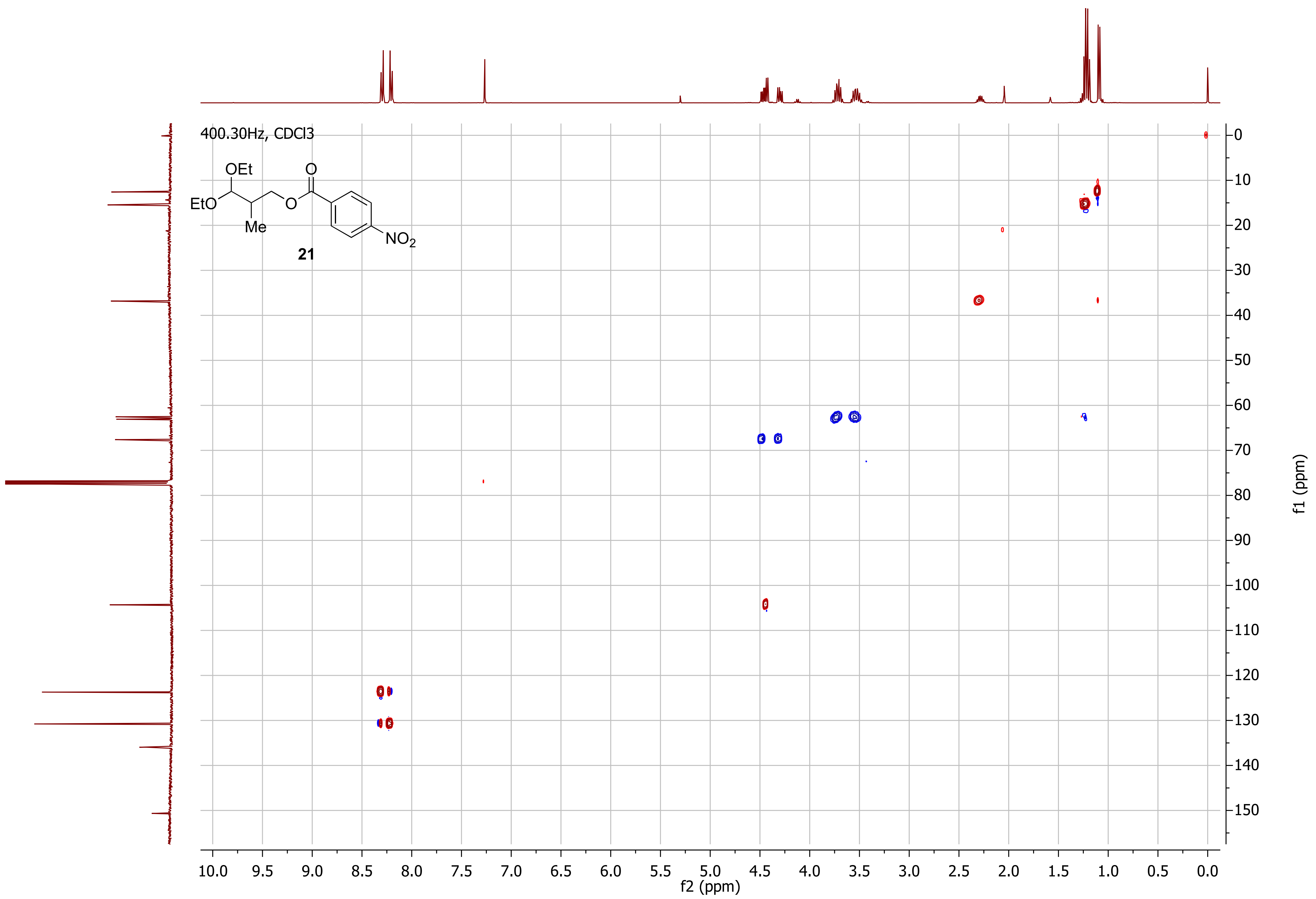
400.30Hz, CDCl3
OEt
O
EtO
O
Me
NO_2
21
f2 (ppm)
f1 (ppm)

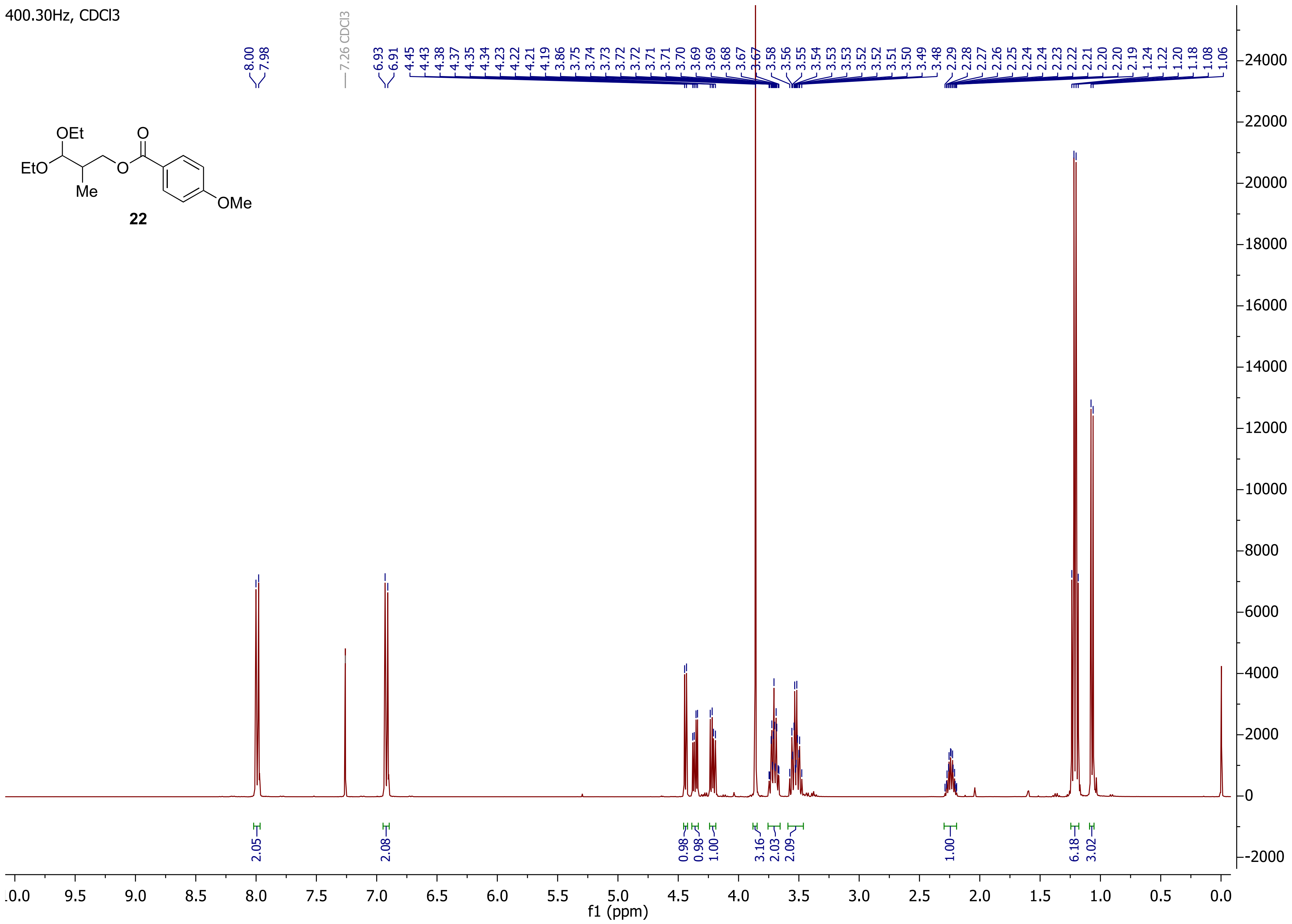
400.30Hz, CDCl3
8.00
7.98
7.26 CDCl3
6.93
6.91
4.45
4.43
4.38
4.37
4.35
4.34
4.23
4.22
4.21
4.19
3.86
3.75
3.74
3.73
3.72
3.72
3.71
3.71
3.70
3.69
3.69
3.68
3.67
3.67
3.58
3.56
3.55
3.54
3.53
3.53
3.52
3.52
3.51
3.50
3.49
3.48
2.29
2.28
2.27
2.26
2.25
2.24
2.24
2.23
2.22
2.21
2.20
2.20
2.19
1.24
1.22
1.20
1.18
1.08
1.06
OEt
EtO
O
O
Me
OMe
22
2.05
2.08
0.98
0.98
1.00
3.16
2.03
2.09
1.00
6.18
3.02
f1 (ppm)

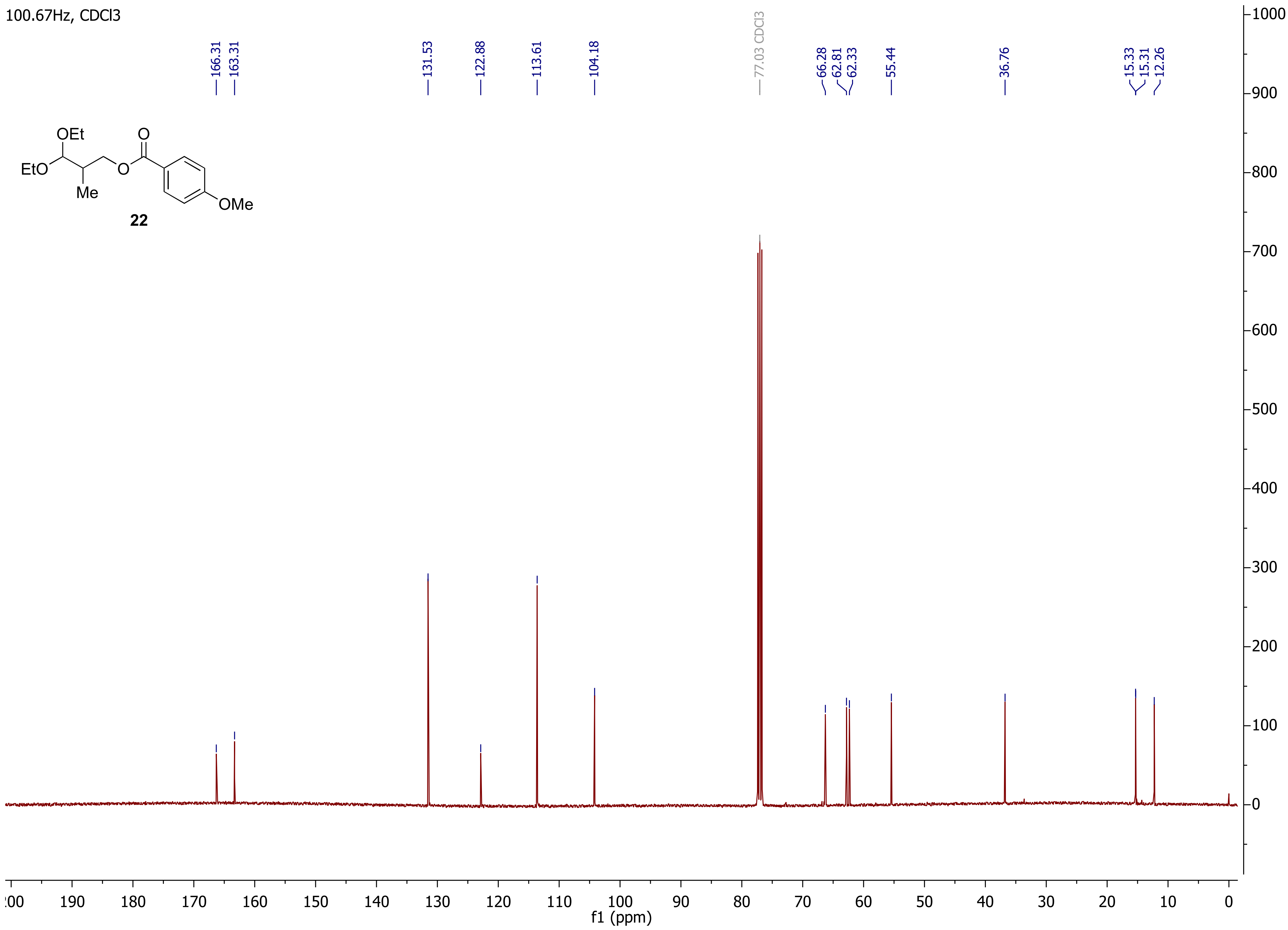
100.67Hz, CDCl3
166.31
163.31
131.53
122.88
113.61
104.18
77.03 CDCl3
66.28
62.81
62.33
55.44
36.76
15.33
15.31
12.26
OEt
EtO
O
O
Me
OMe
22
f1 (ppm)

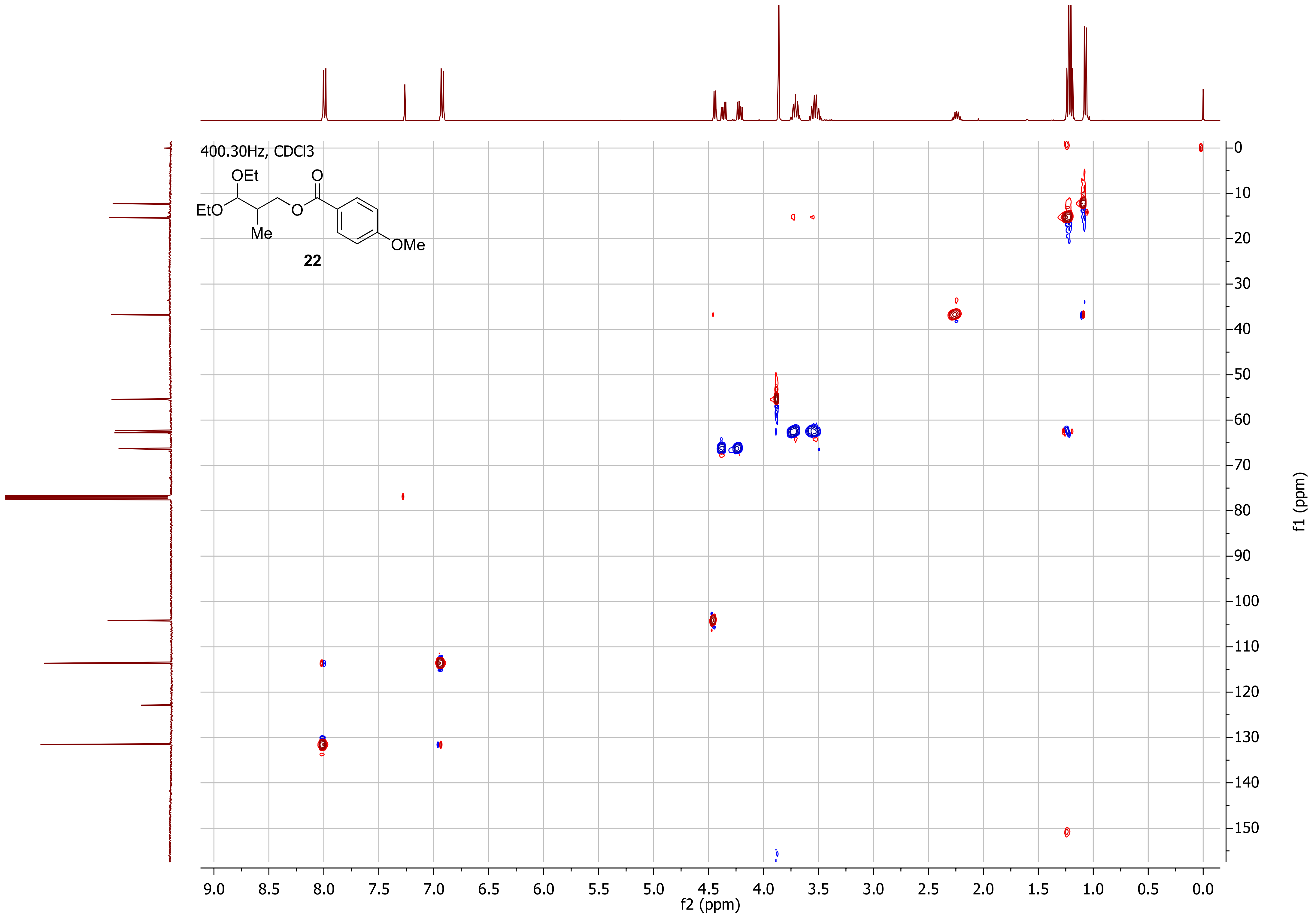
400.30Hz, CDCl3
OEt
EtO
O
Me
O
OMe
22
f2 (ppm)
f1 (ppm)

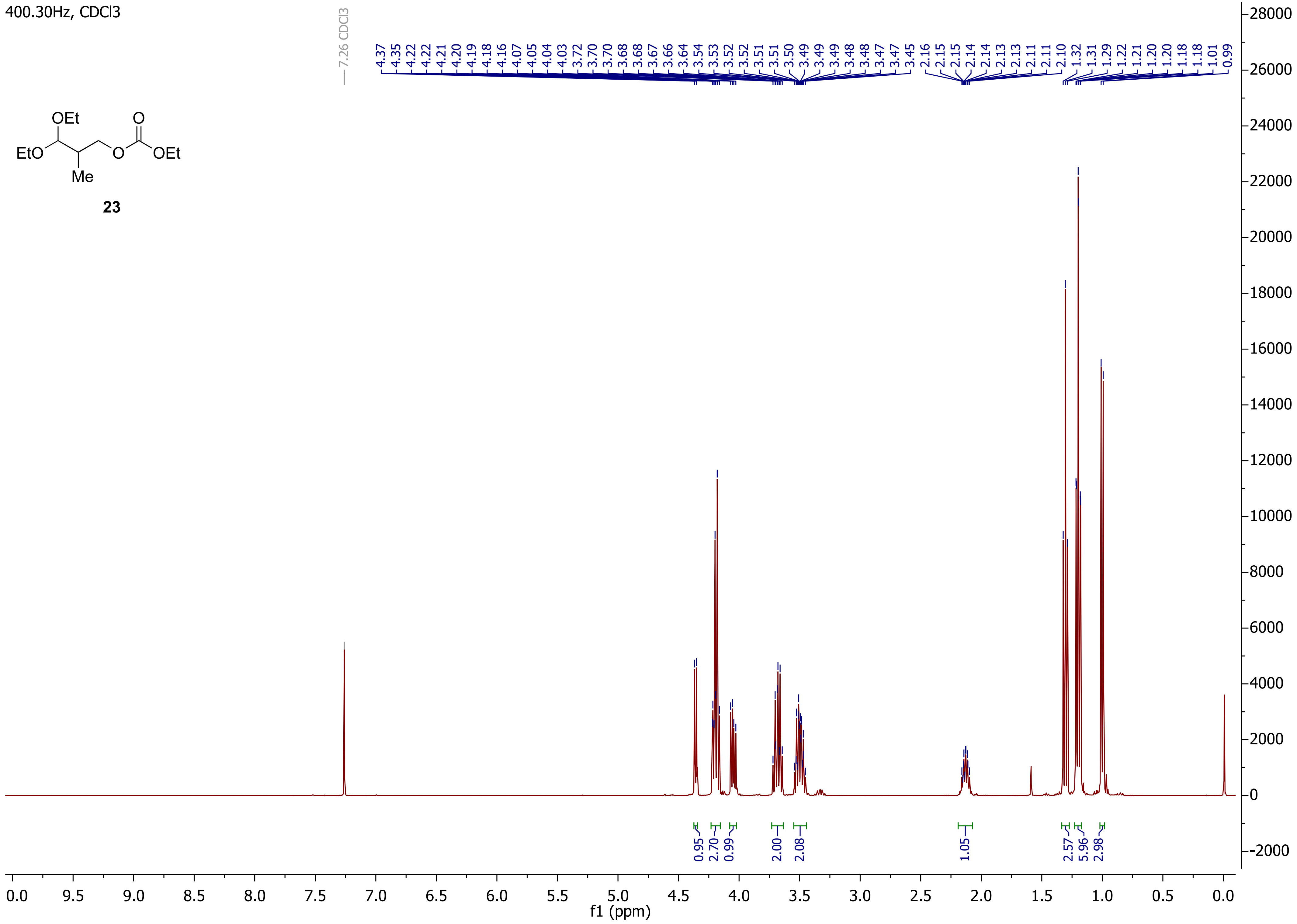
400.30Hz, CDCl3
7.26 CDCl3
4.37
4.35
4.22
4.22
4.21
4.20
4.19
4.18
4.16
4.07
4.05
4.04
4.03
3.72
3.70
3.70
3.68
3.68
3.67
3.66
3.64
3.54
3.53
3.52
3.52
3.51
3.51
3.50
3.49
3.49
3.49
3.48
3.48
3.47
3.47
3.45
2.16
2.15
2.15
2.14
2.14
2.13
2.13
2.11
2.11
2.10
1.32
1.31
1.29
1.22
1.21
1.20
1.20
1.18
1.18
1.01
0.99
OEt
O
EtO
O
OEt
Me
23
0.95
2.70
0.99
2.00
2.08
1.05
2.57
5.96
2.98
f1 (ppm)

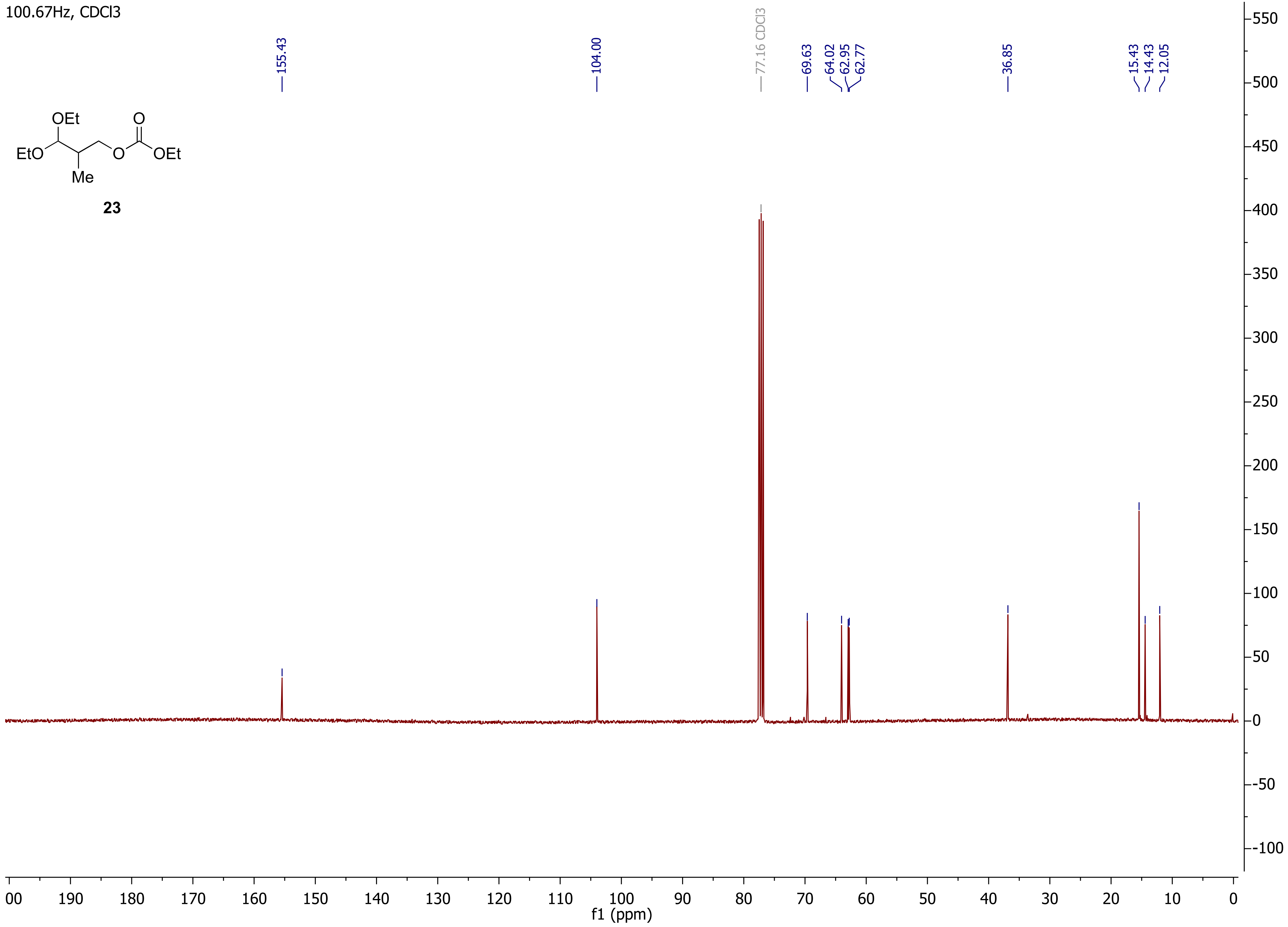
100.67Hz, CDCl3
155.43
104.00
77.16 CDCl3
69.63
64.02
62.95
62.77
36.85
15.43
14.43
12.05
OEt
EtO
O
OEt
Me
23
f1 (ppm)

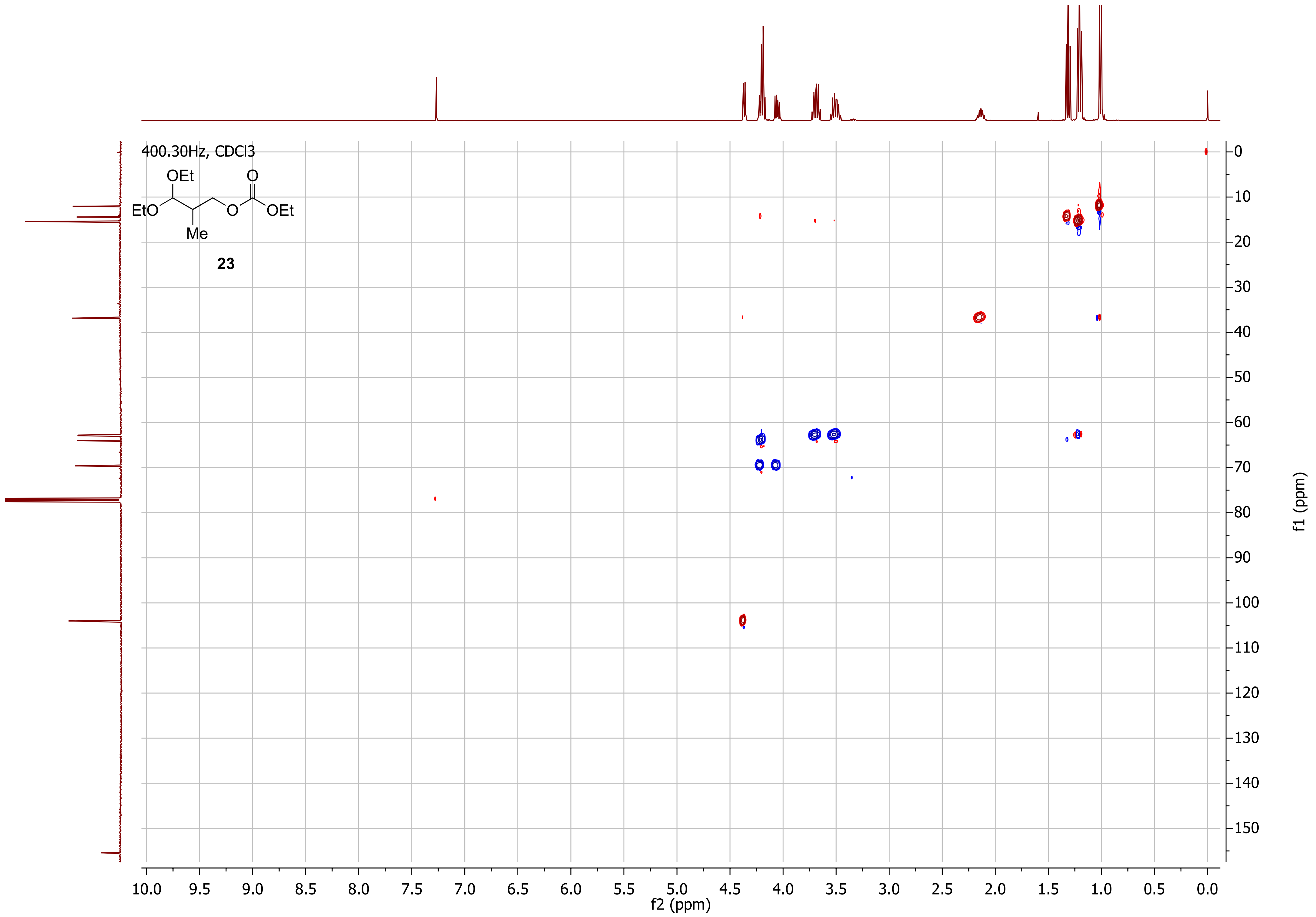
400.30Hz, CDCl3
OEt
O
EtO
O
OEt
Me
23
f2 (ppm)
f1 (ppm)

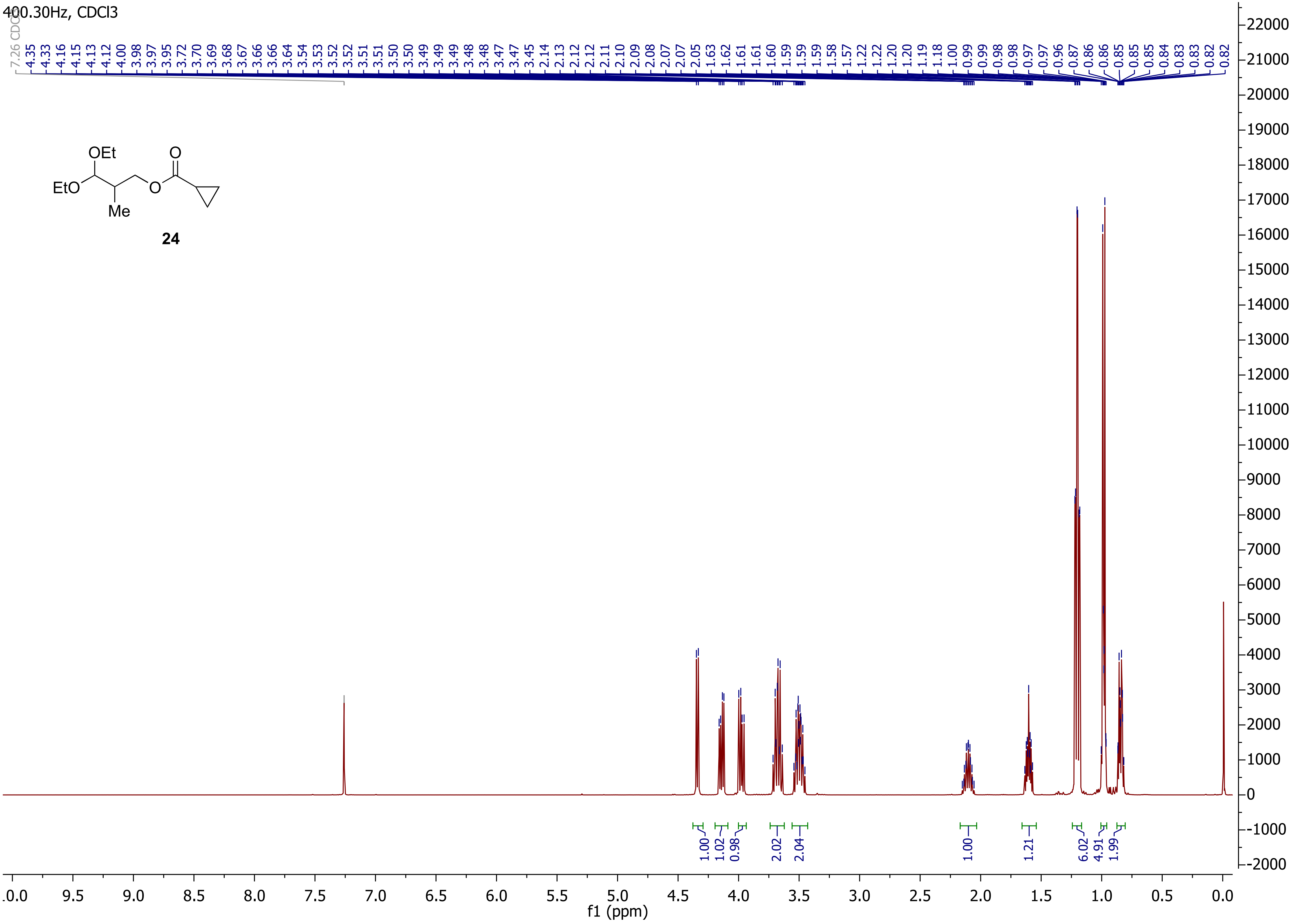
400.30Hz, CDCl3
7.26 CDCl3
4.35 4.33 4.16 4.15 4.13 4.12 4.00 3.98 3.97 3.95 3.72 3.70 3.69 3.68 3.67 3.66 3.66 3.64 3.54 3.53 3.52 3.52 3.51 3.51 3.50 3.50 3.49 3.49 3.49 3.48 3.48 3.47 3.47 3.45 2.14 2.13 2.12 2.12 2.11 2.10 2.09 2.08 2.07 2.07 2.05 1.63 1.62 1.61 1.61 1.60 1.59 1.59 1.59 1.58 1.57 1.22 1.22 1.20 1.20 1.19 1.18 1.00 0.99 0.99 0.98 0.98 0.97 0.97 0.96 0.87 0.86 0.86 0.85 0.85 0.85 0.84 0.83 0.83 0.82 0.82
OEt
EtO
O
O
Me
24
1.00 1.02 0.98 2.02 2.04 1.00 1.21 6.02 4.91 1.99
f1 (ppm)

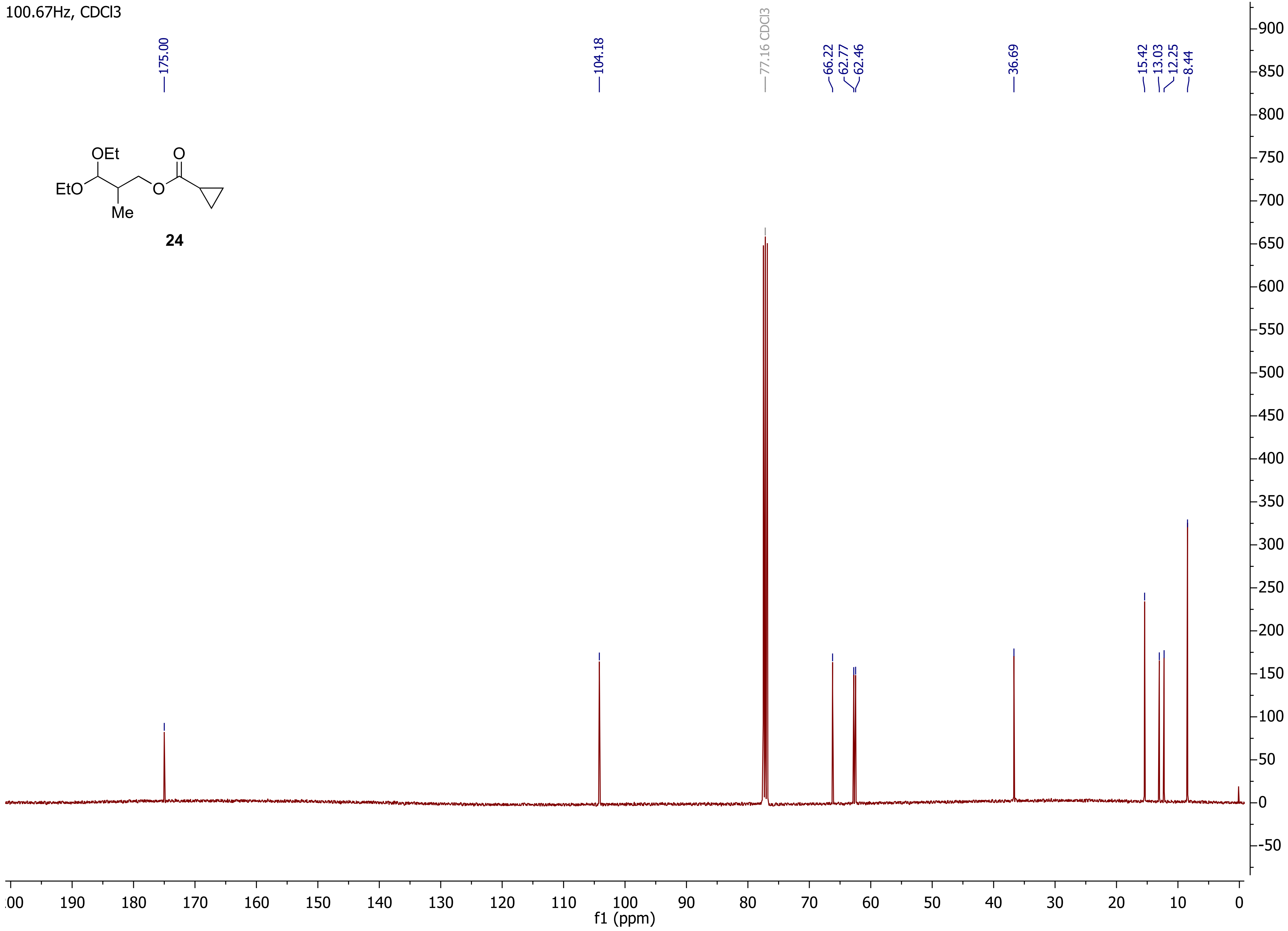
100.67Hz, CDCl3
175.00
104.18
77.16 CDCl3
66.22
62.77
62.46
36.69
15.42
13.03
12.25
8.44
OEt
O
EtO
O
Me
24
900
850
800
750
700
650
600
550
500
450
400
350
300
250
200
150
100
50
0
-50
200
190
180
170
160
150
140
130
120
110
100
90
80
70
60
50
40
30
20
10
0
f1 (ppm)

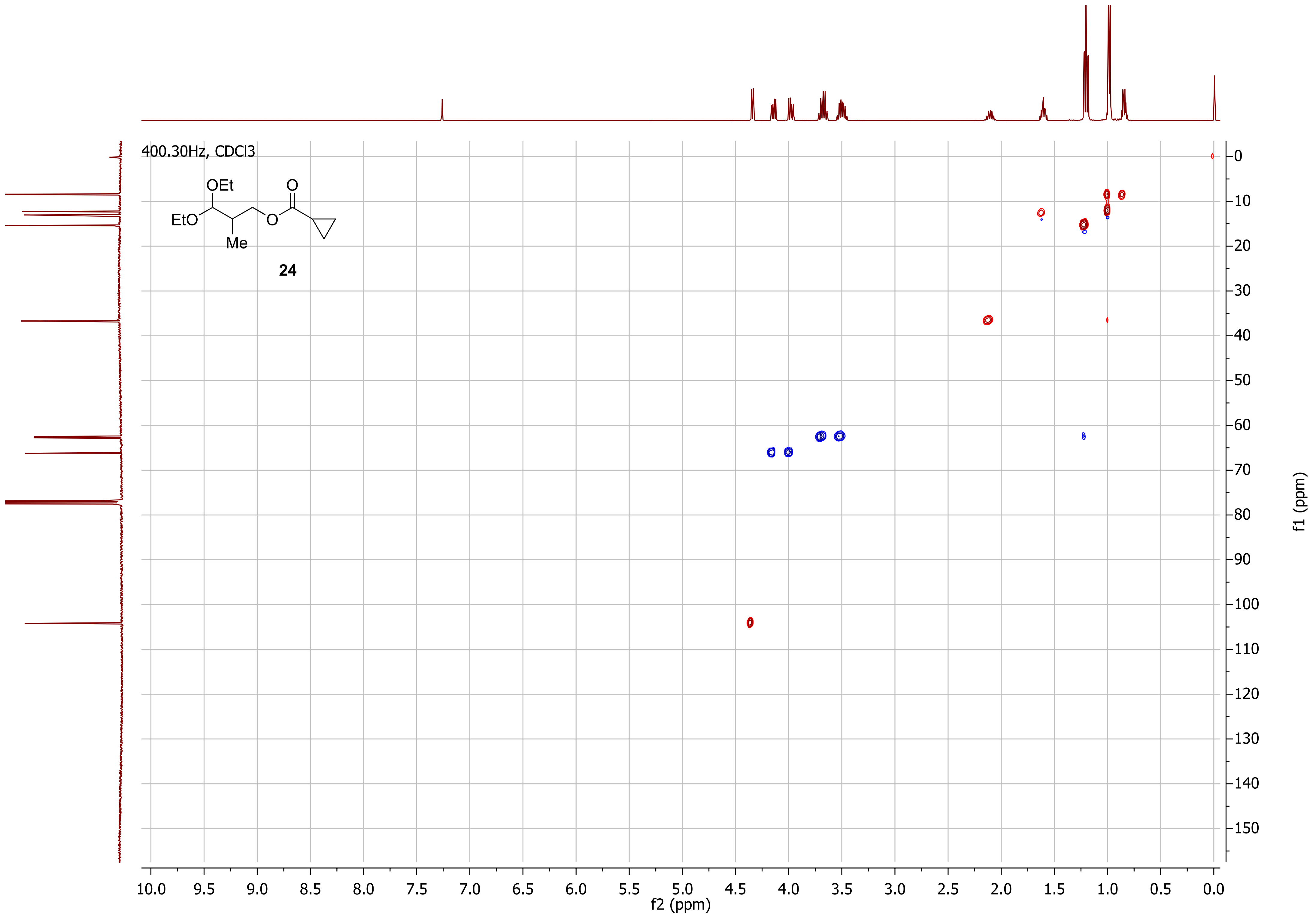

400.30Hz, CDCl3
OEt
O
EtO
O
Me
24
f1 (ppm)
f2 (ppm)

400.30Hz, CDCl3

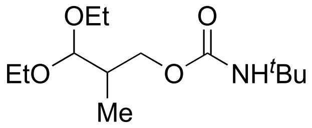

25

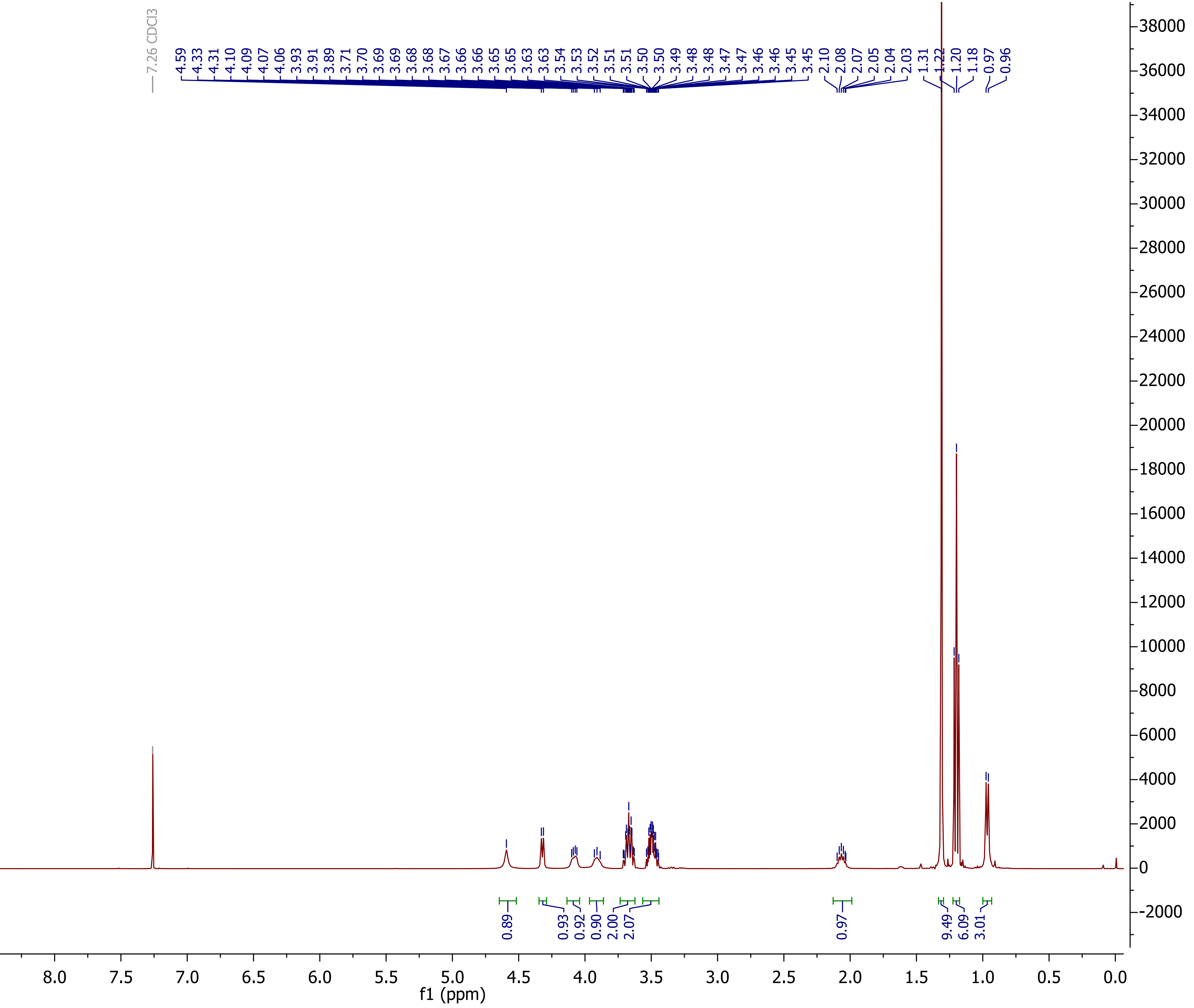

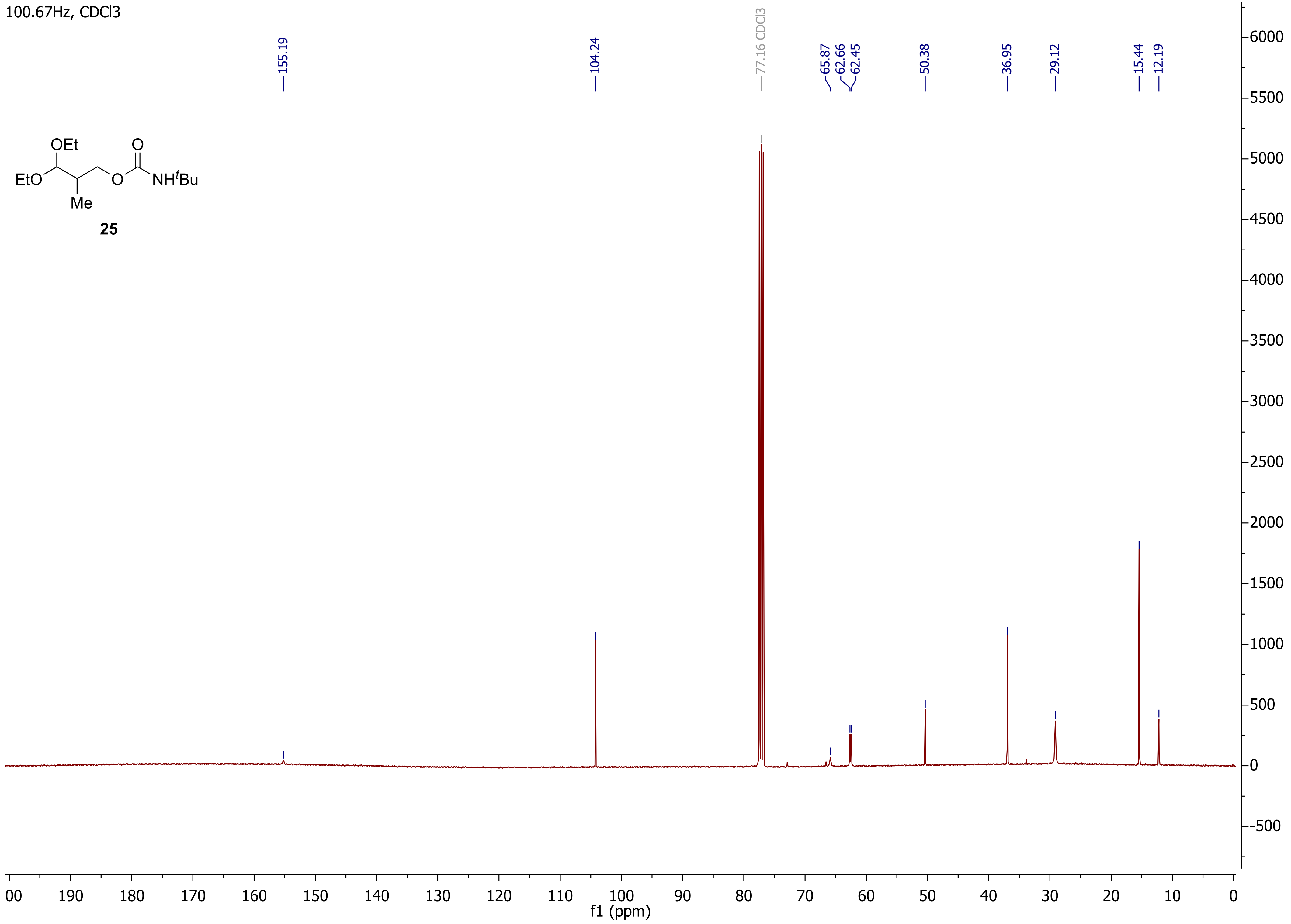
100.67Hz, CDCl3
155.19
104.24
77.16 CDCl3
65.87
62.66
62.45
50.38
36.95
29.12
15.44
12.19
OEt
O
EtO
O
NH^{t}Bu
Me
25
f1 (ppm)
6000
5500
5000
4500
4000
3500
3000
2500
2000
1500
1000
500
0
-500
00
190
180
170
160
150
140
130
120
110
100
90
80
70
60
50
40
30
20
10
0

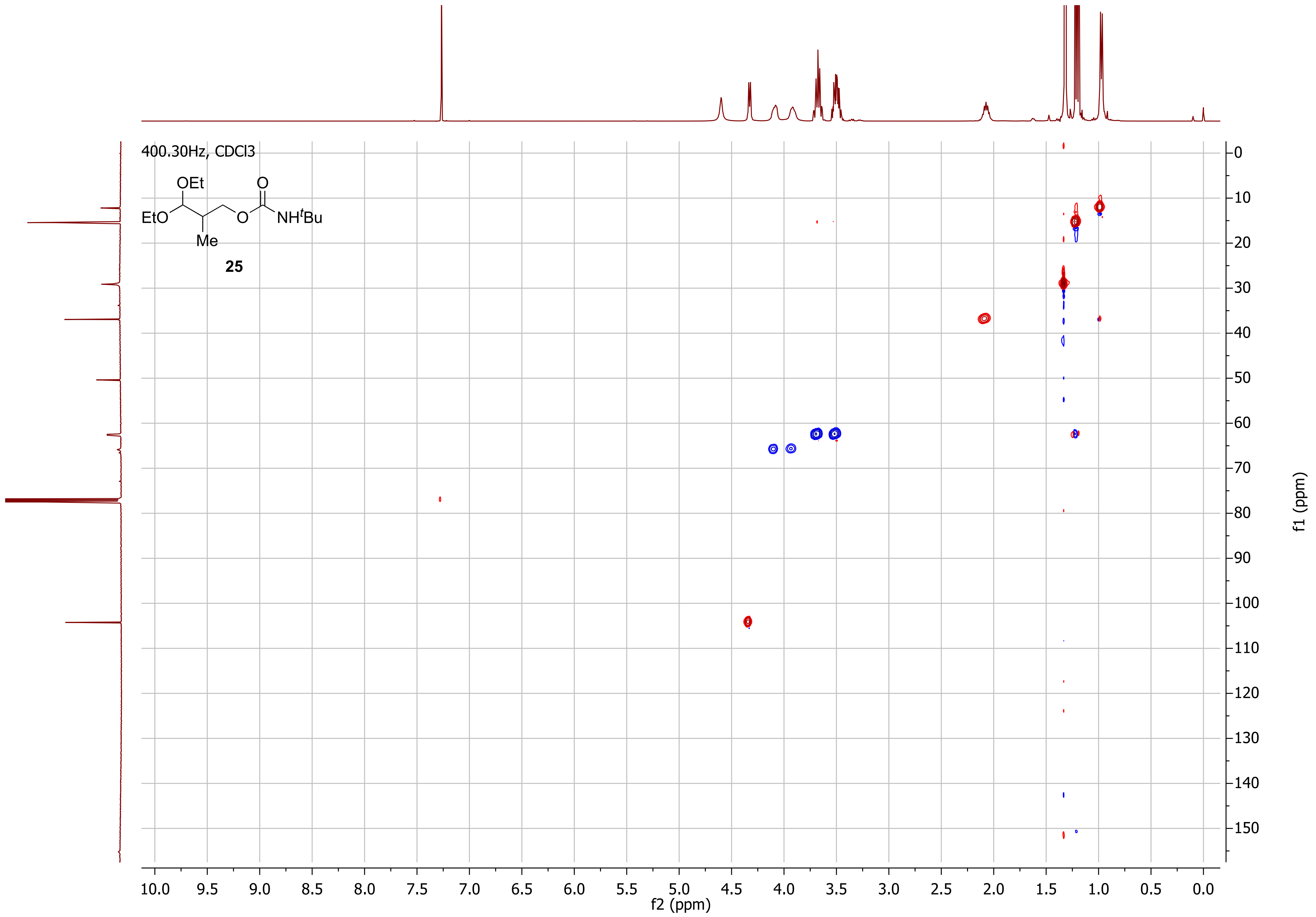
400.30Hz, CDCl3
OEt
O
EtO
O
NH^{t}Bu
Me
25
f2 (ppm)
f1 (ppm)

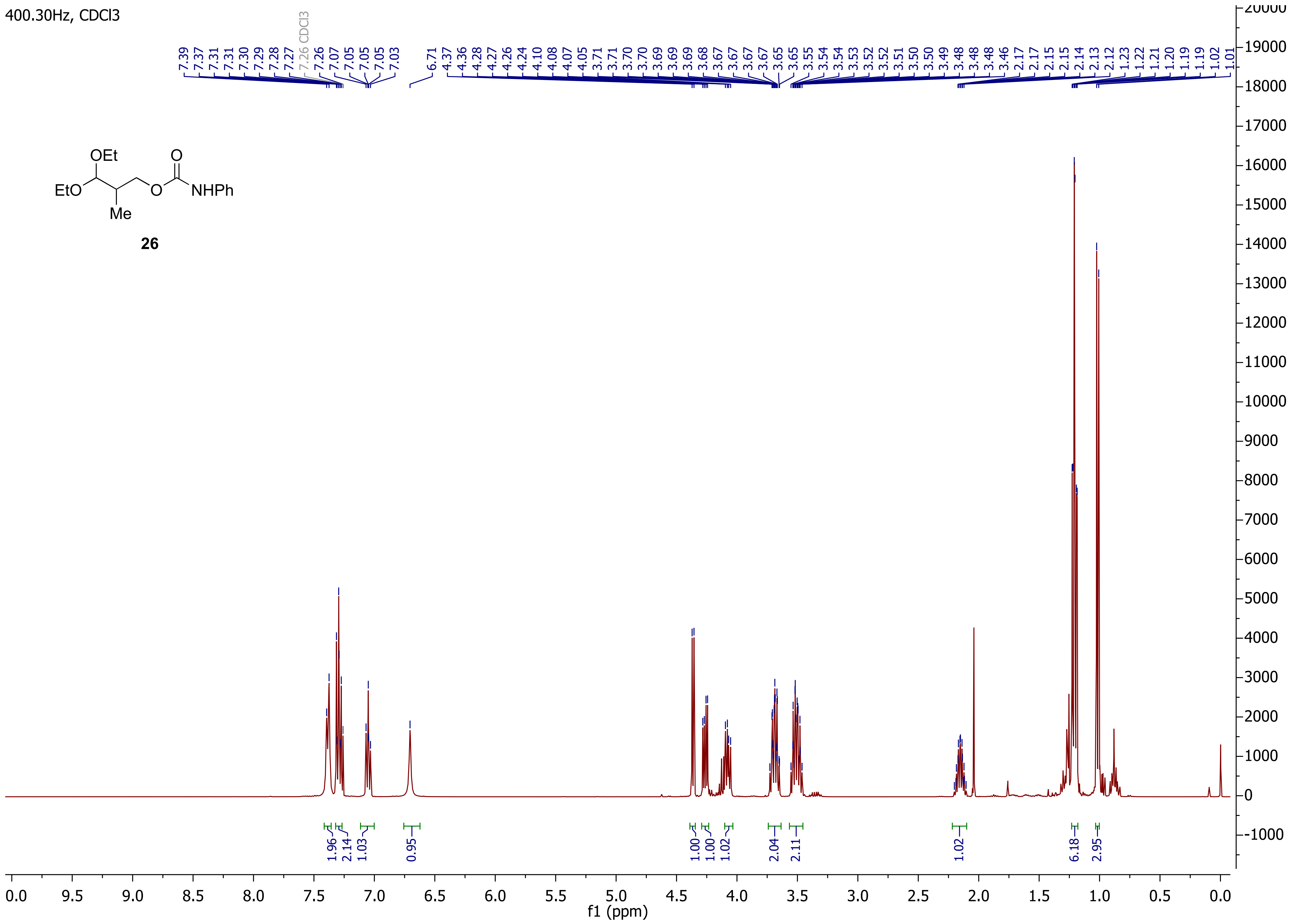

400.30Hz, CDCl3
7.39 7.37 7.31 7.31 7.30 7.29 7.28 7.27 7.26 CDCl3 7.26 7.07 7.05 7.05 7.05 7.03 6.71 4.37 4.36 4.28 4.27 4.26 4.24 4.10 4.08 4.07 4.05 3.71 3.71 3.70 3.70 3.69 3.69 3.69 3.68 3.67 3.67 3.67 3.67 3.65 3.65 3.55 3.54 3.54 3.53 3.52 3.52 3.51 3.50 3.50 3.49 3.48 3.48 3.48 3.46 2.17 2.17 2.15 2.15 2.14 2.13 2.12 1.23 1.22 1.21 1.20 1.19 1.19 1.02 1.01
OEt
O
EtO
O
NHPh
Me
26
1.96
2.14
1.03
0.95
1.00
1.00
1.02
2.04
2.11
1.02
6.18
2.95
f1 (ppm)
0.0 9.5 9.0 8.5 8.0 7.5 7.0 6.5 6.0 5.5 5.0 4.5 4.0 3.5 3.0 2.5 2.0 1.5 1.0 0.5 0.0
20000 19000 18000 17000 16000 15000 14000 13000 12000 11000 10000 9000 8000 7000 6000 5000 4000 3000 2000 1000 0 -1000

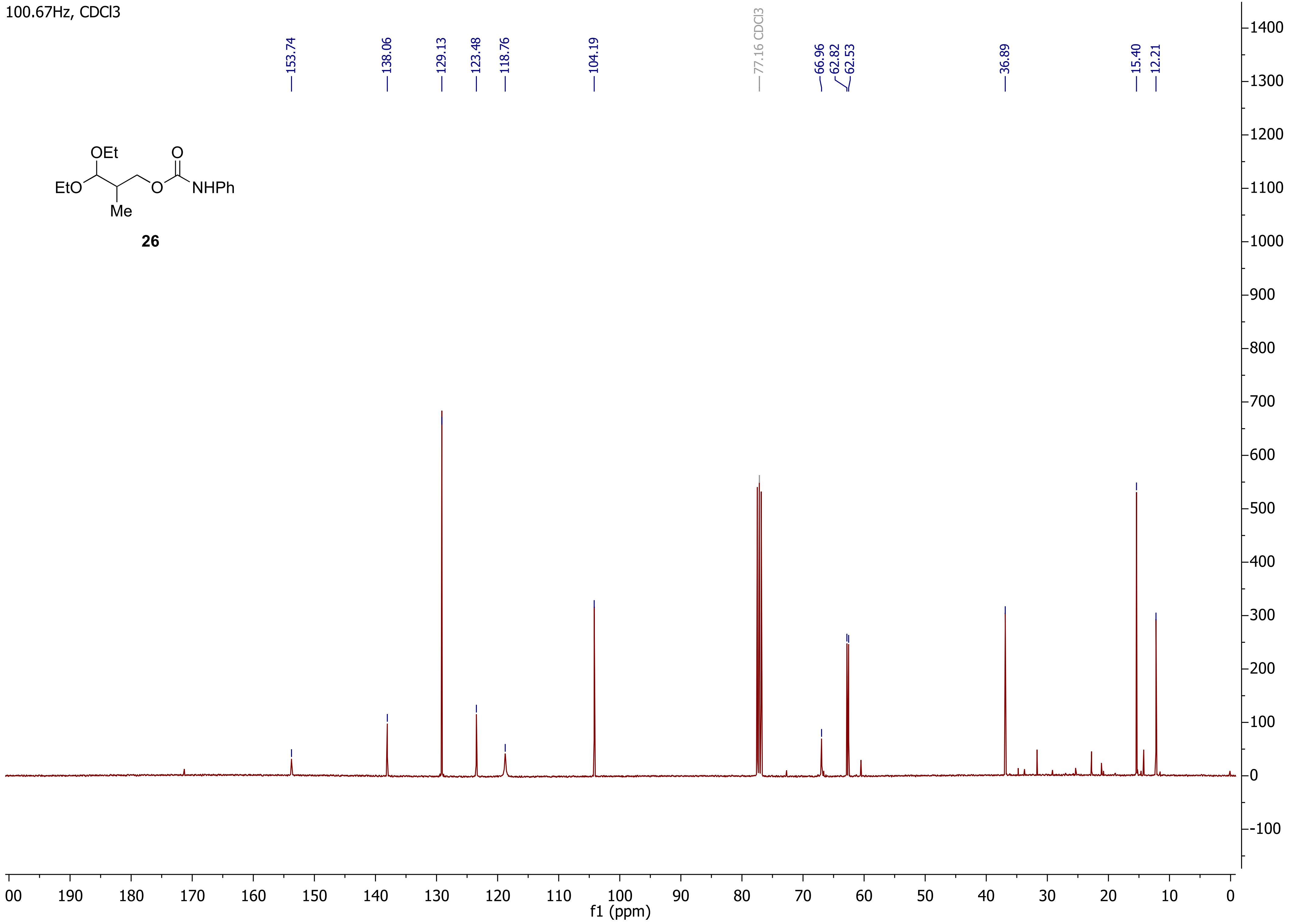

100.67Hz, CDCl3
153.74
138.06
129.13
123.48
118.76
104.19
77.16 CDCl3
66.96
62.82
62.53
36.89
15.40
12.21
OEt
EtO
O
Me
O
NHPh
26
f1 (ppm)

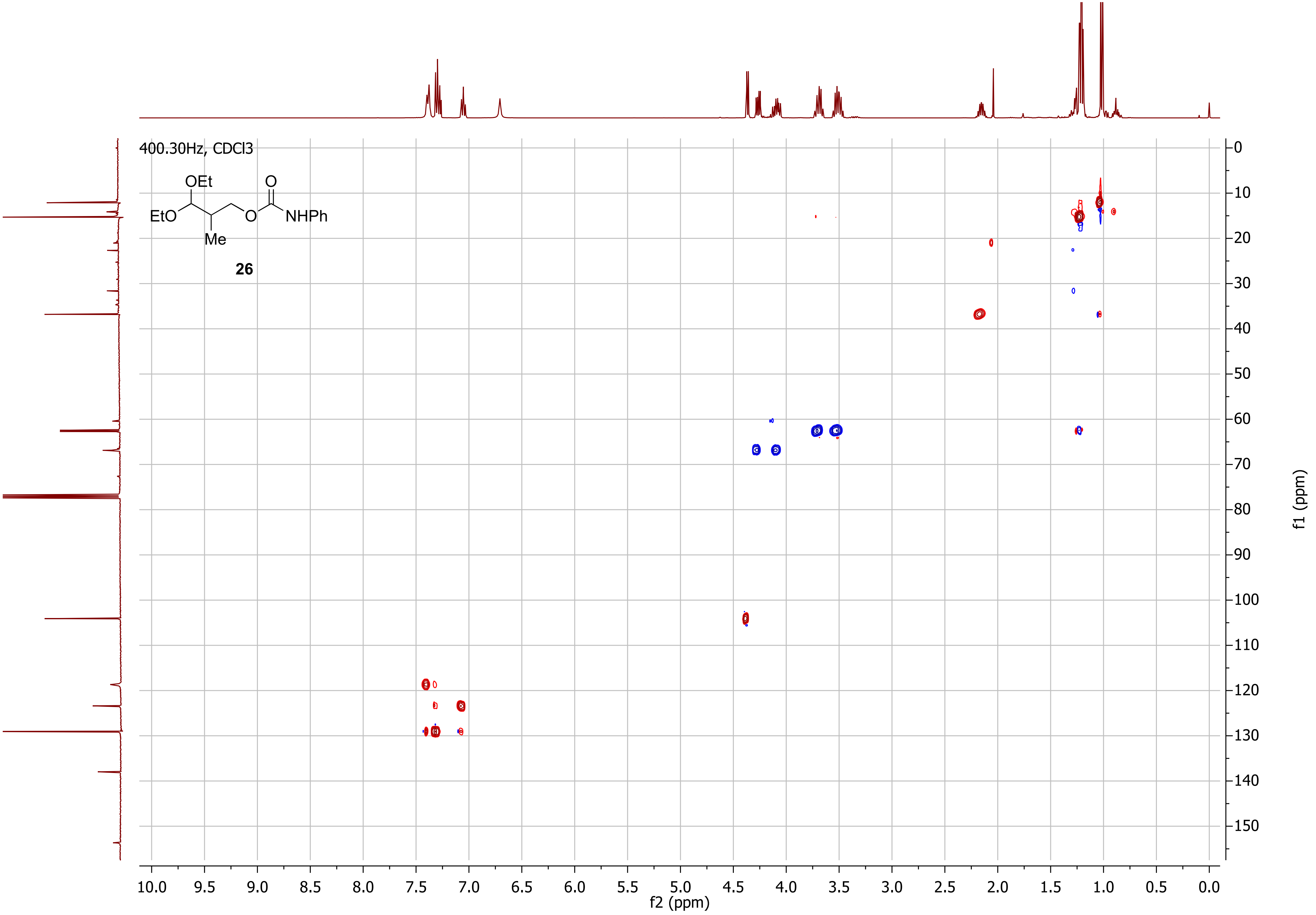

400.30Hz, CDCl3
OEt
O
EtO
O
NHPh
Me
26
f2 (ppm)
f1 (ppm)

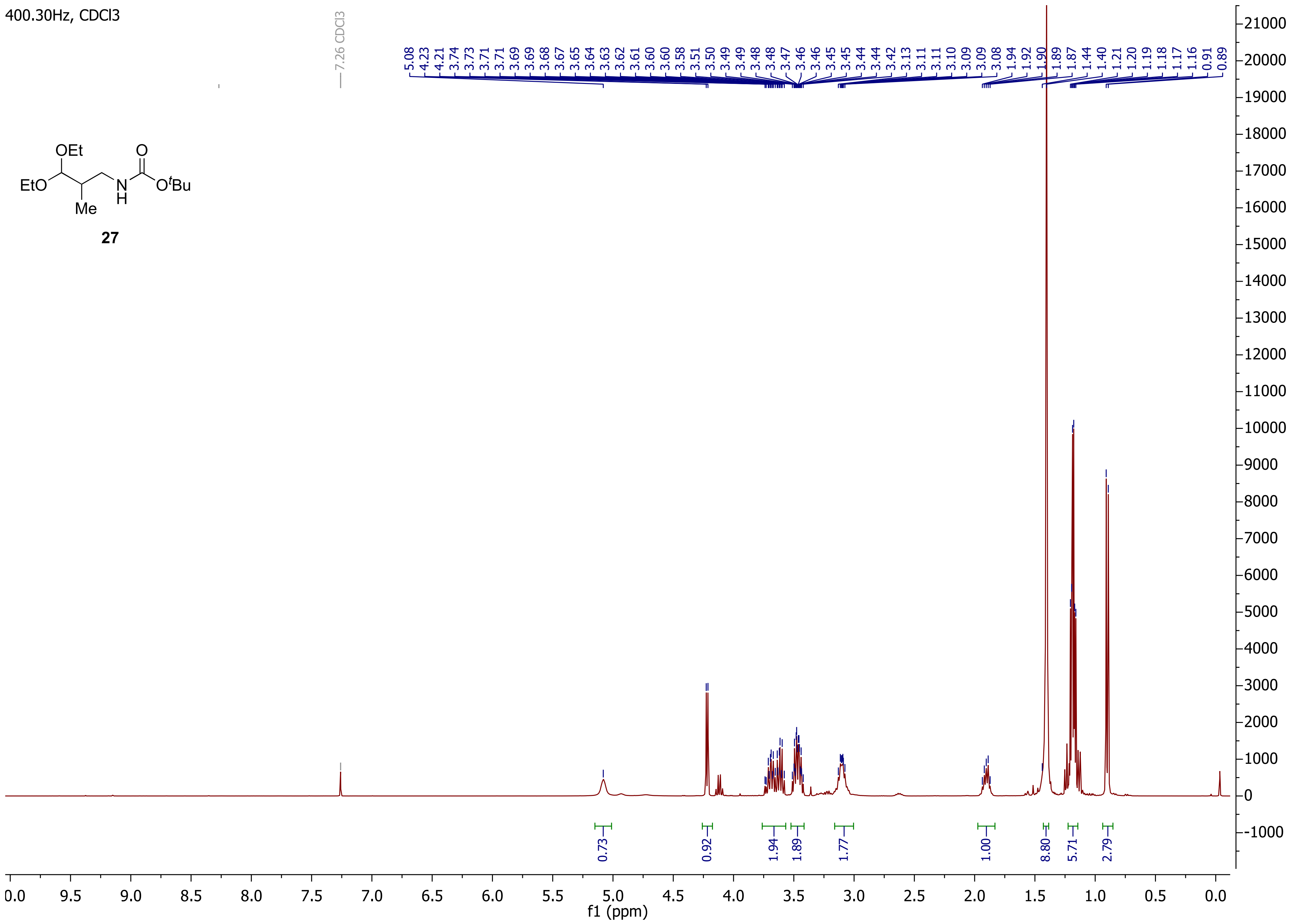
400.30Hz, CDCl3
7.26 CDCl3
5.08 4.23 4.21 3.74 3.73 3.71 3.71 3.69 3.69 3.68 3.67 3.65 3.64 3.63 3.62 3.61 3.60 3.60 3.58 3.51 3.50 3.49 3.49 3.48 3.48 3.47 3.46 3.46 3.45 3.45 3.44 3.44 3.42 3.13 3.11 3.11 3.10 3.09 3.09 3.08 1.94 1.92 1.90 1.89 1.87 1.44 1.40 1.21 1.20 1.19 1.18 1.17 1.16 0.91 0.89
OEt
EtO
O
N
H
O^{t}Bu
Me
27
0.73 0.92 1.94 1.89 1.77 1.00 8.80 5.71 2.79
f1 (ppm)

100.67Hz, CDCl3

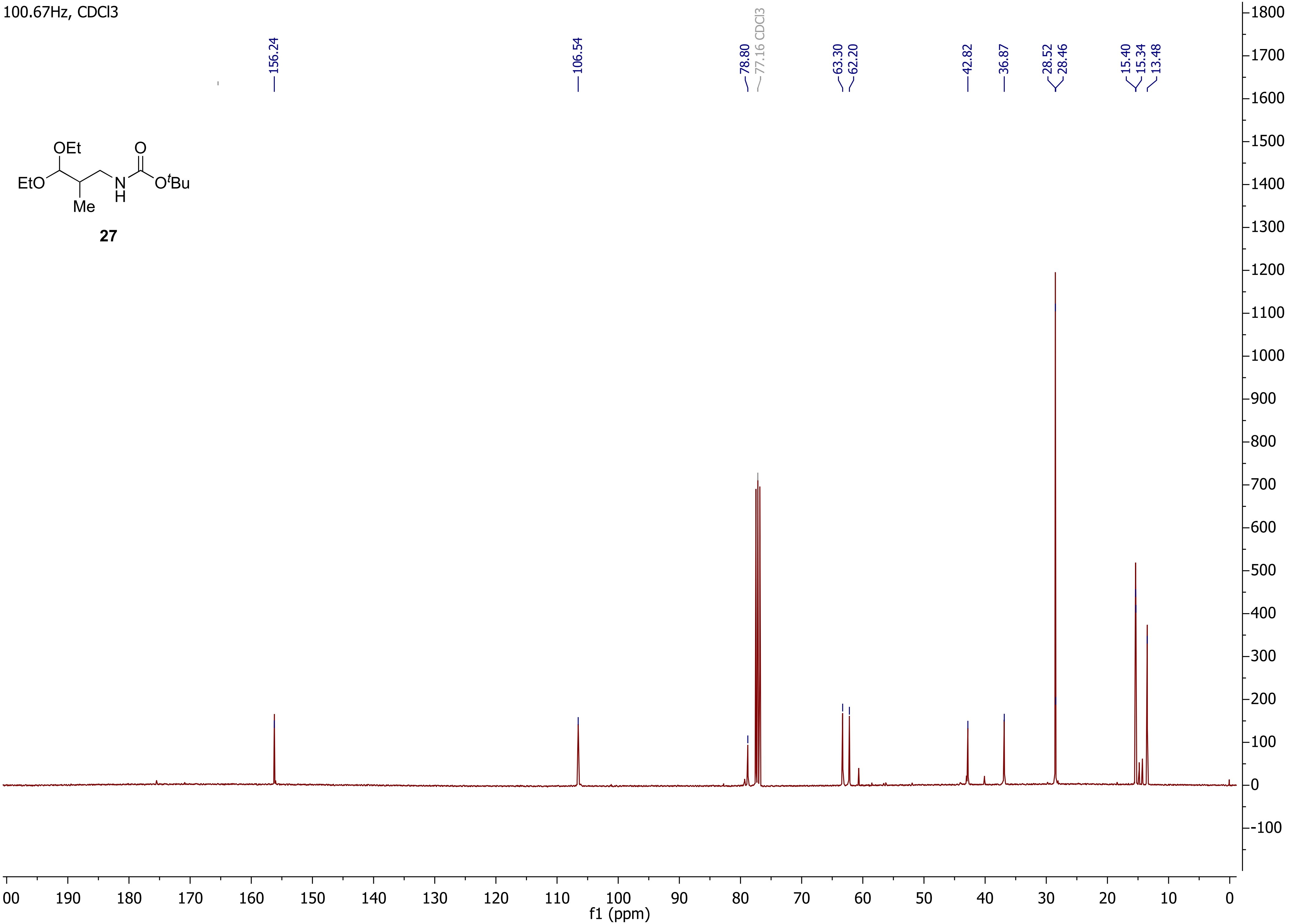

27

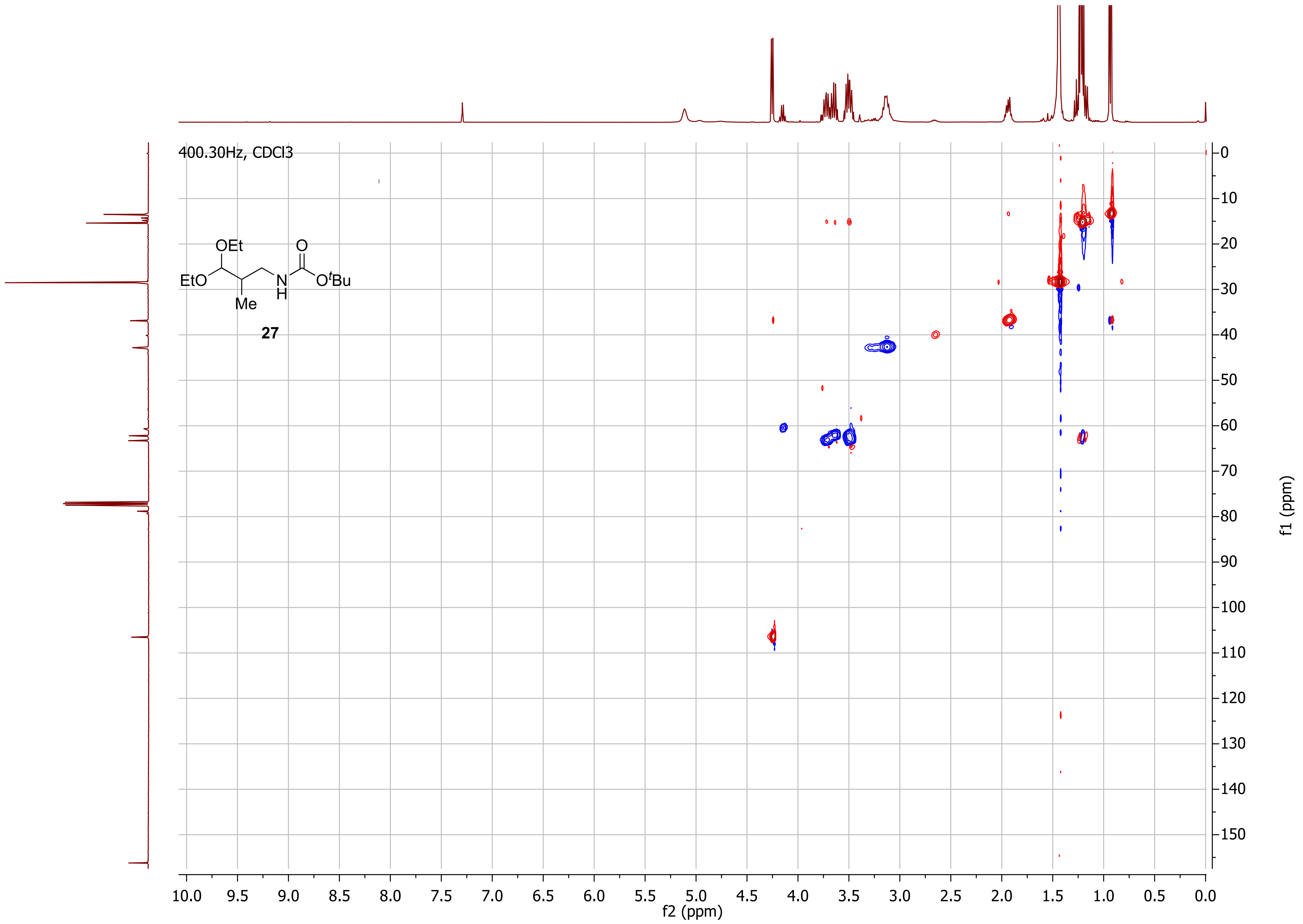
400.30Hz, CDCl3
OEt
O
EtO
N
H
O^tBu
Me
27
f2 (ppm)
f1 (ppm)

400.30Hz, CDCl3

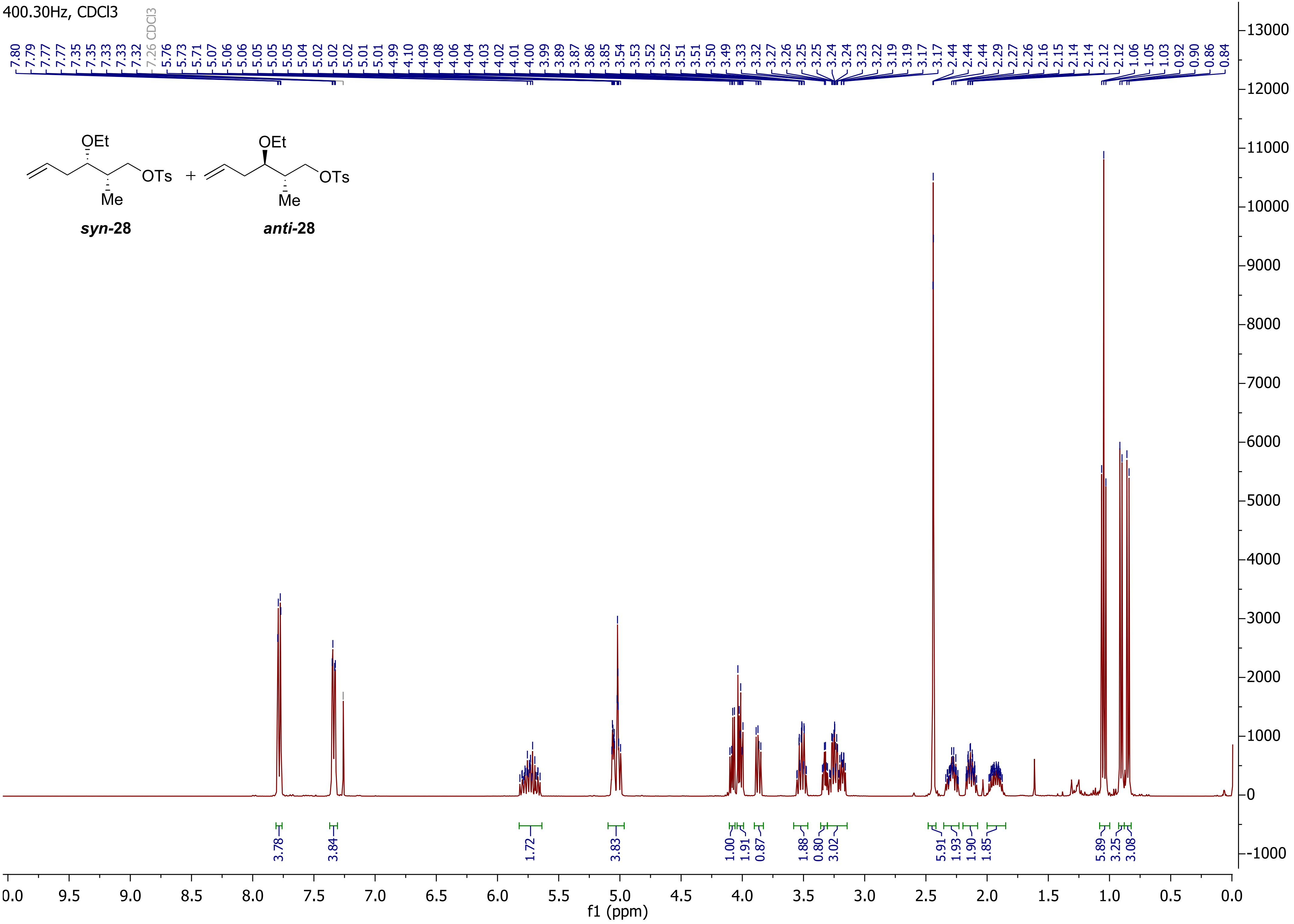

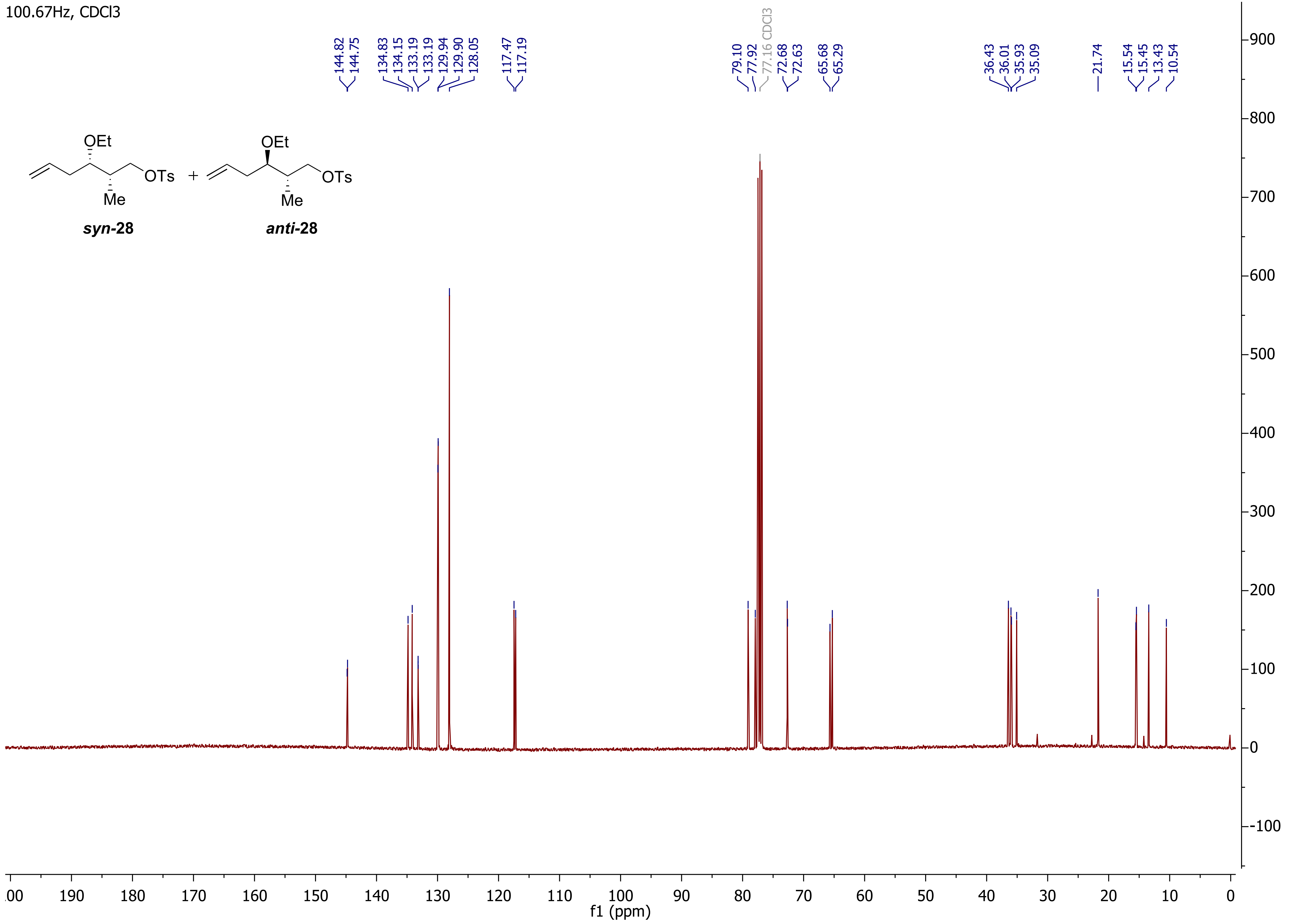

100.67Hz, CDCl3
144.82
144.75
134.83
134.15
133.19
133.19
129.94
129.90
128.05
117.47
117.19
79.10
77.92
77.16 CDCl3
72.68
72.63
65.68
65.29
36.43
36.01
35.93
35.09
21.74
15.54
15.45
13.43
10.54
OEt
OTs
Me
syn-28
anti-28
f1 (ppm)

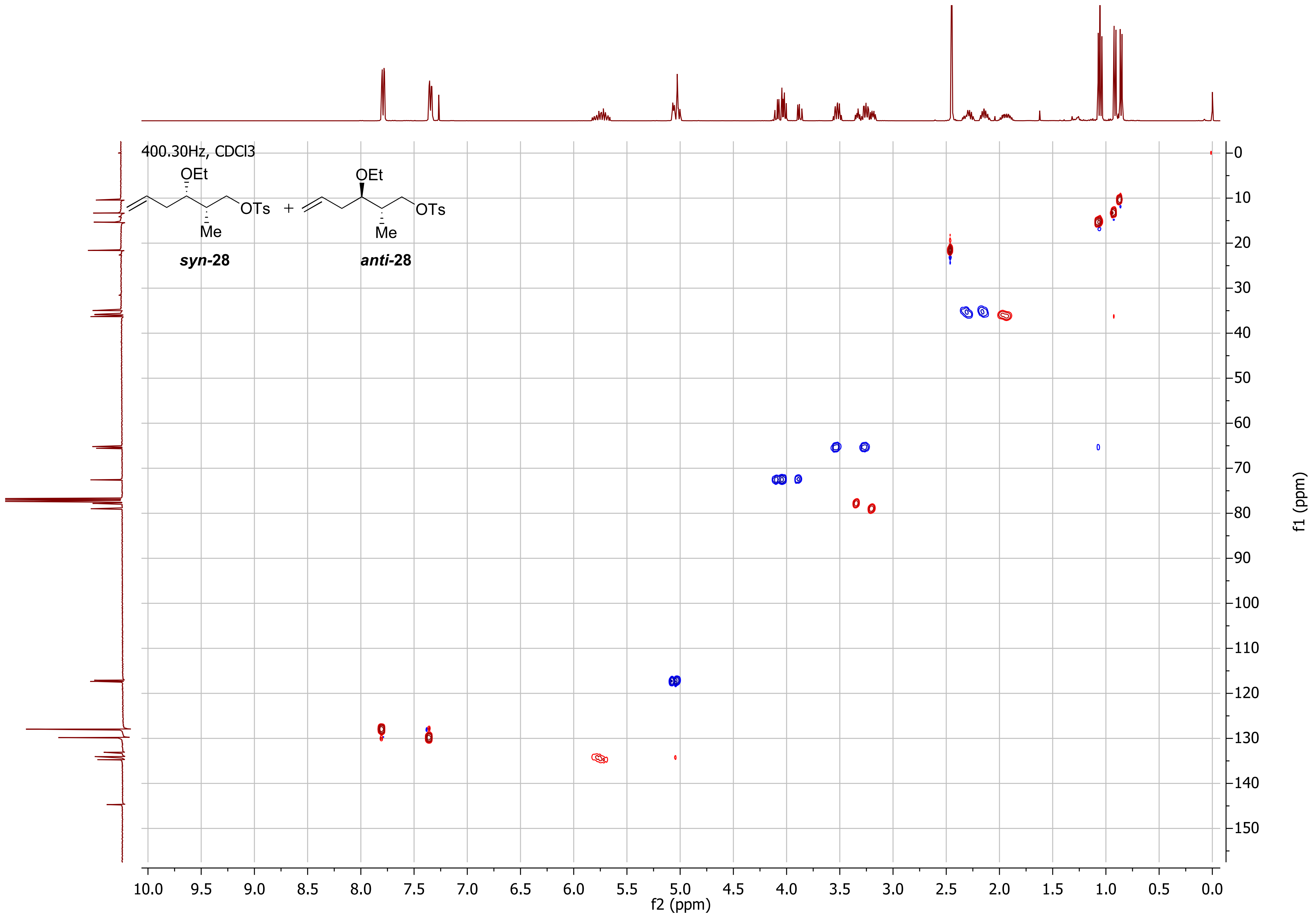
400.30Hz, CDCl3
OEt
OTs
Me
+
OEt
OTs
Me
syn-28
anti-28
f1 (ppm)
f2 (ppm)

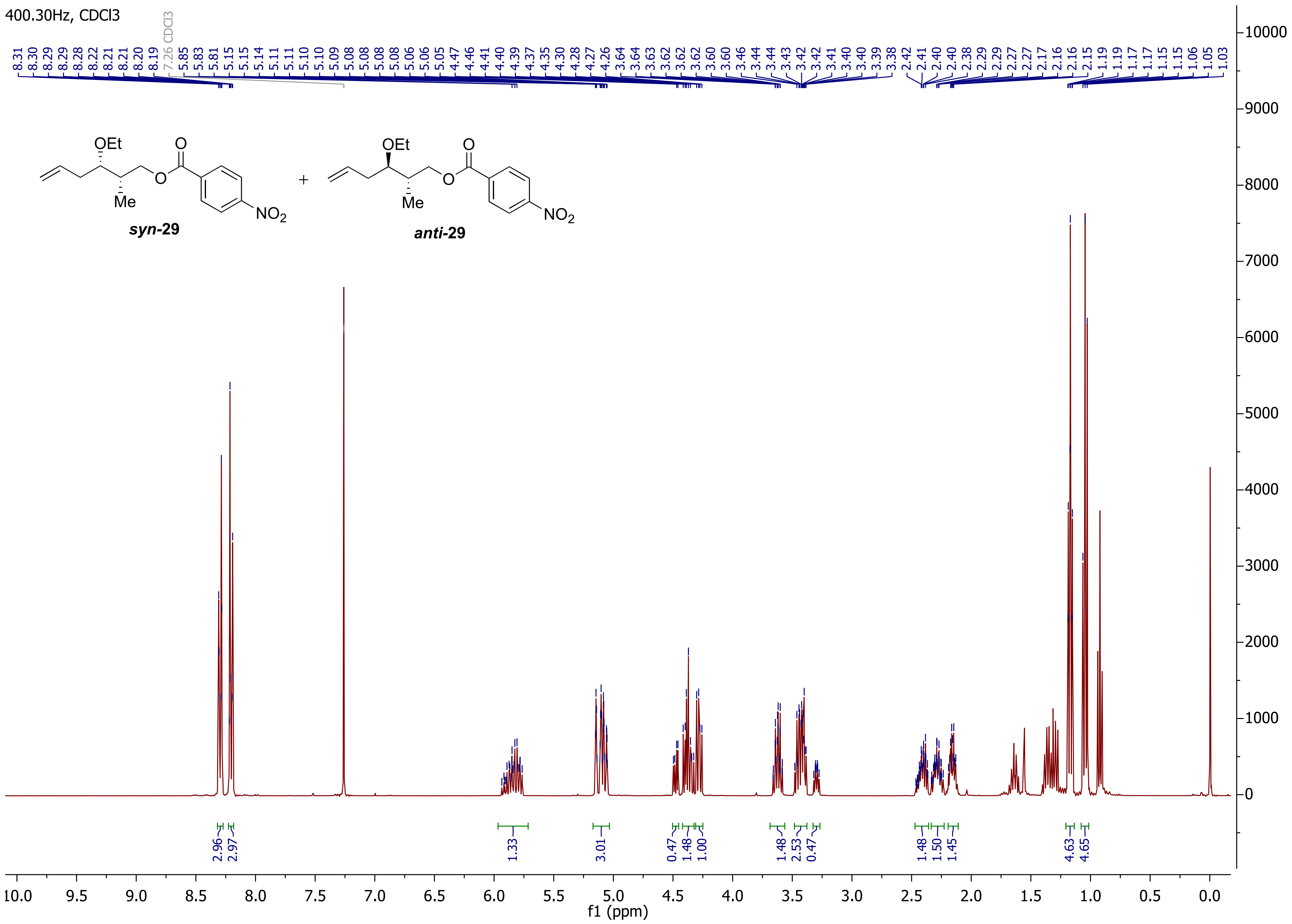

400.30Hz, CDCl3
syn-29
anti-29
OEt
Me
NO2
f1 (ppm)

100.67Hz, CDCl3

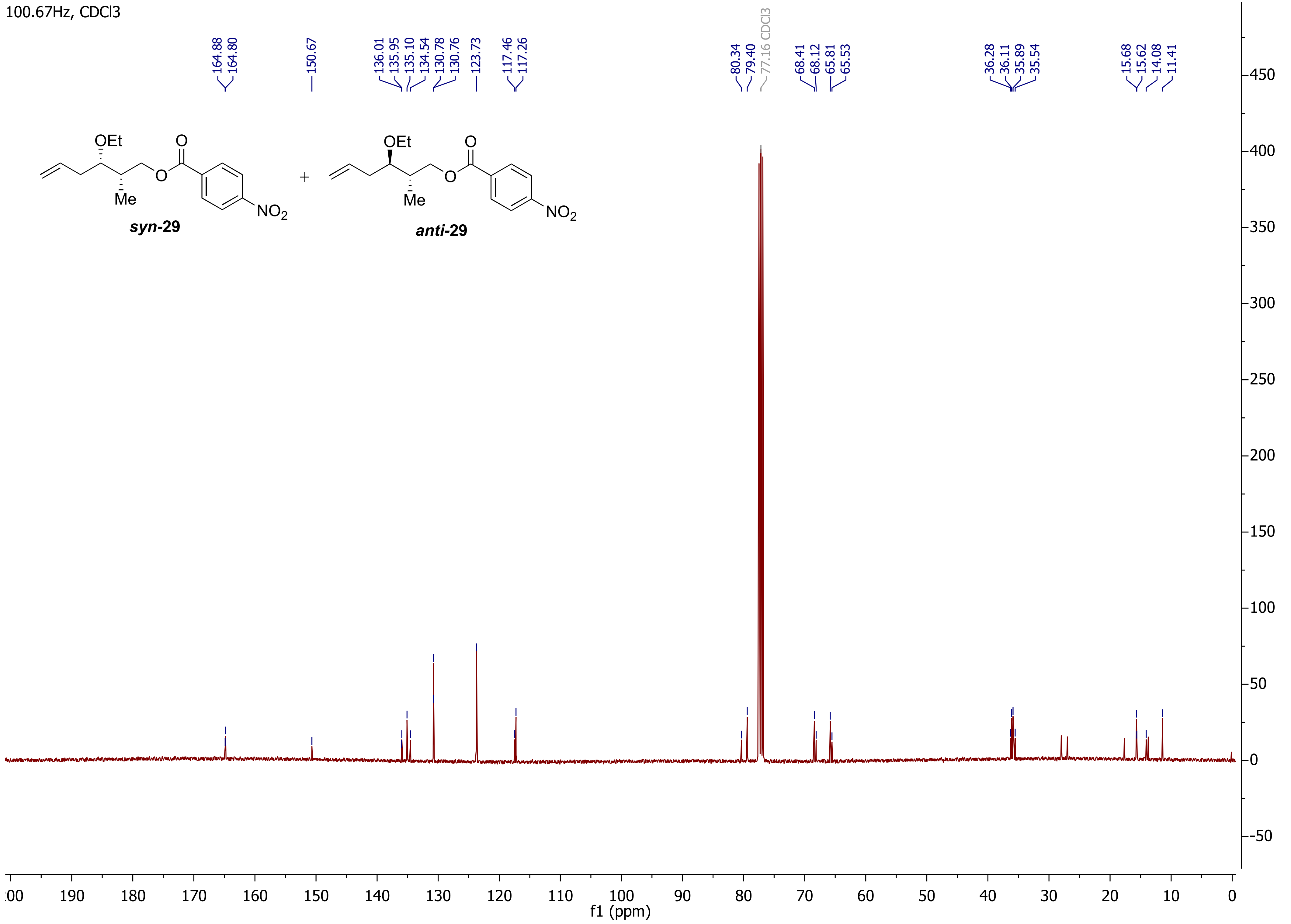

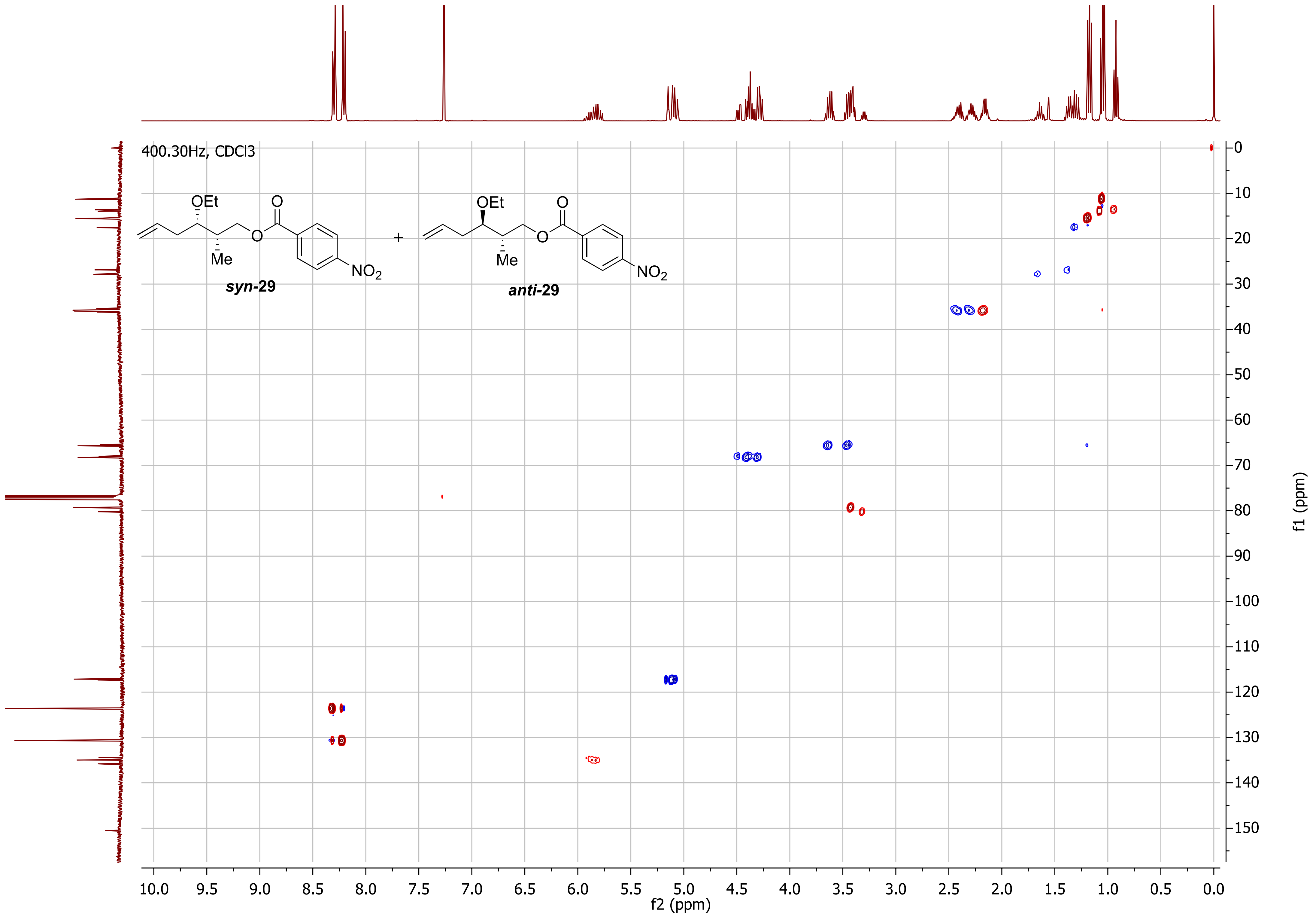

400.30Hz, CDCl3
OEt
O
O
Me
NO_2
syn-29
+
OEt
O
O
Me
NO_2
anti-29
f2 (ppm)
f1 (ppm)

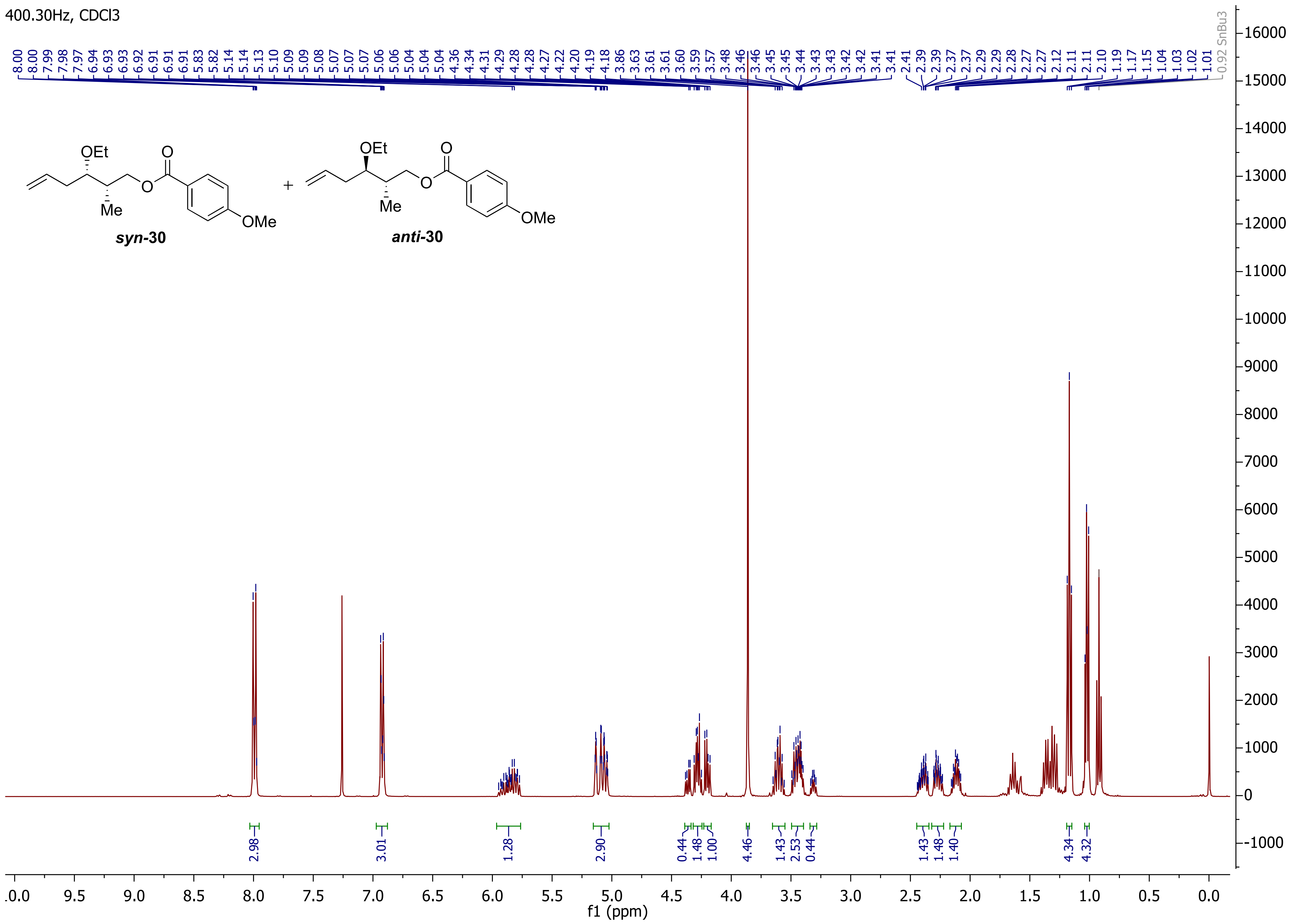
400.30Hz, CDCl3
OEt
O
Me
OMe
syn-30
+
anti-30
f1 (ppm)

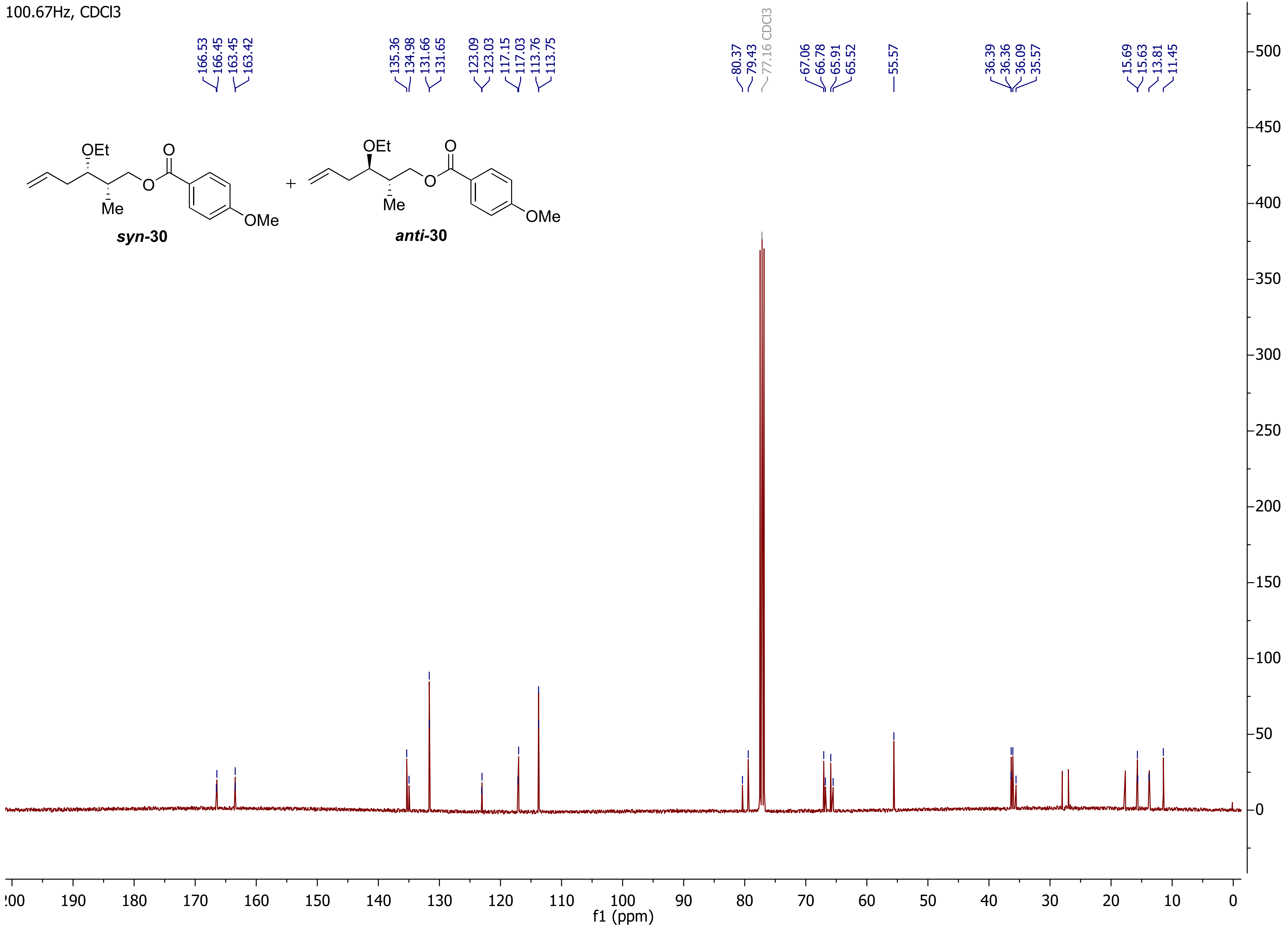
100.67Hz, CDCl3
166.53
166.45
163.45
163.42
135.36
134.98
131.66
131.65
123.09
123.03
117.15
117.03
113.76
113.75
80.37
79.43
77.16 CDCl3
67.06
66.78
65.91
65.52
55.57
36.39
36.36
36.09
35.57
15.69
15.63
13.81
11.45
OEt
O
O
Me
OMe
syn-30
+
anti-30
f1 (ppm)

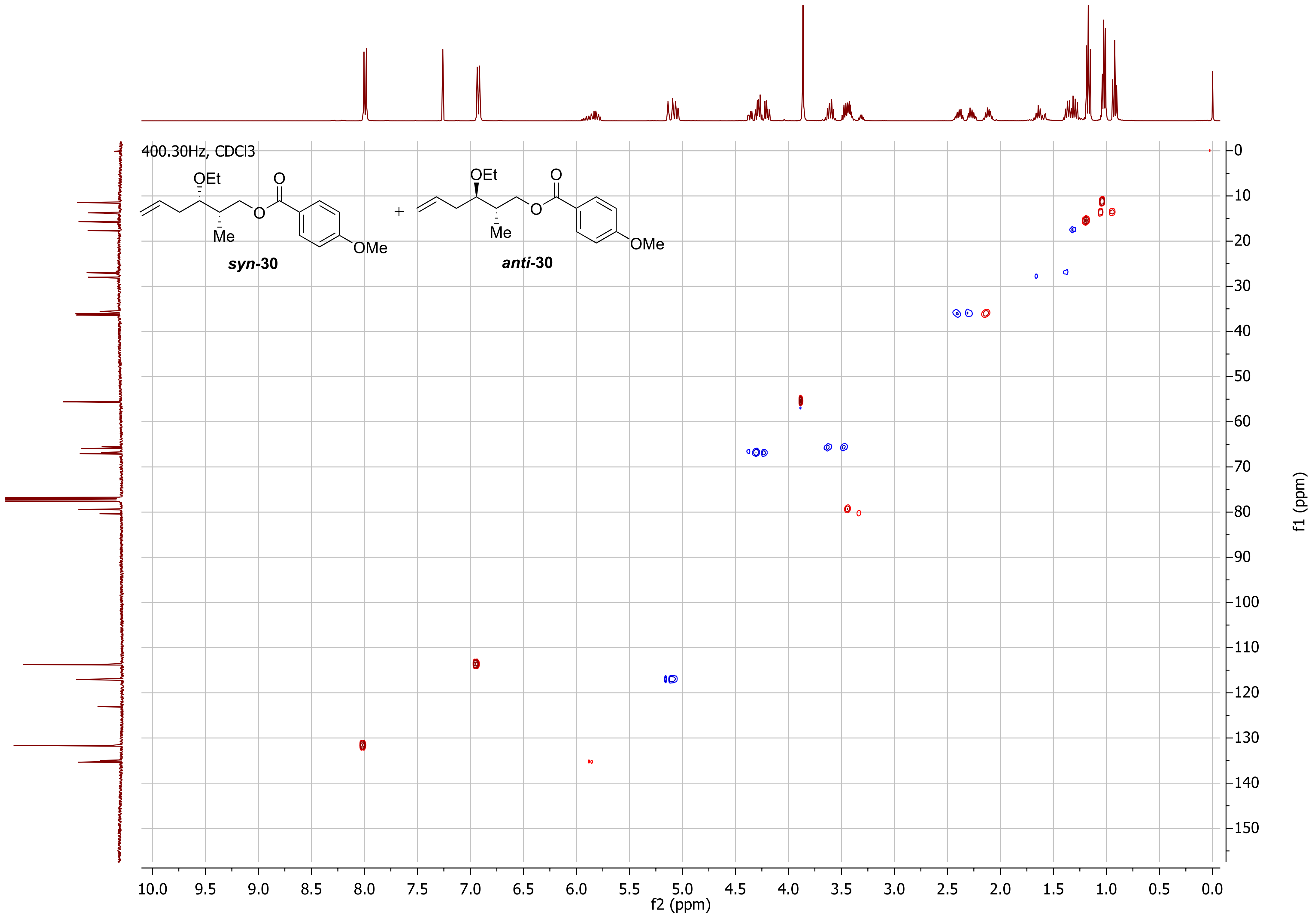
400.30Hz, CDCl3
OEt
O
O
Me
OMe
syn-30
+
anti-30
f2 (ppm)
f1 (ppm)

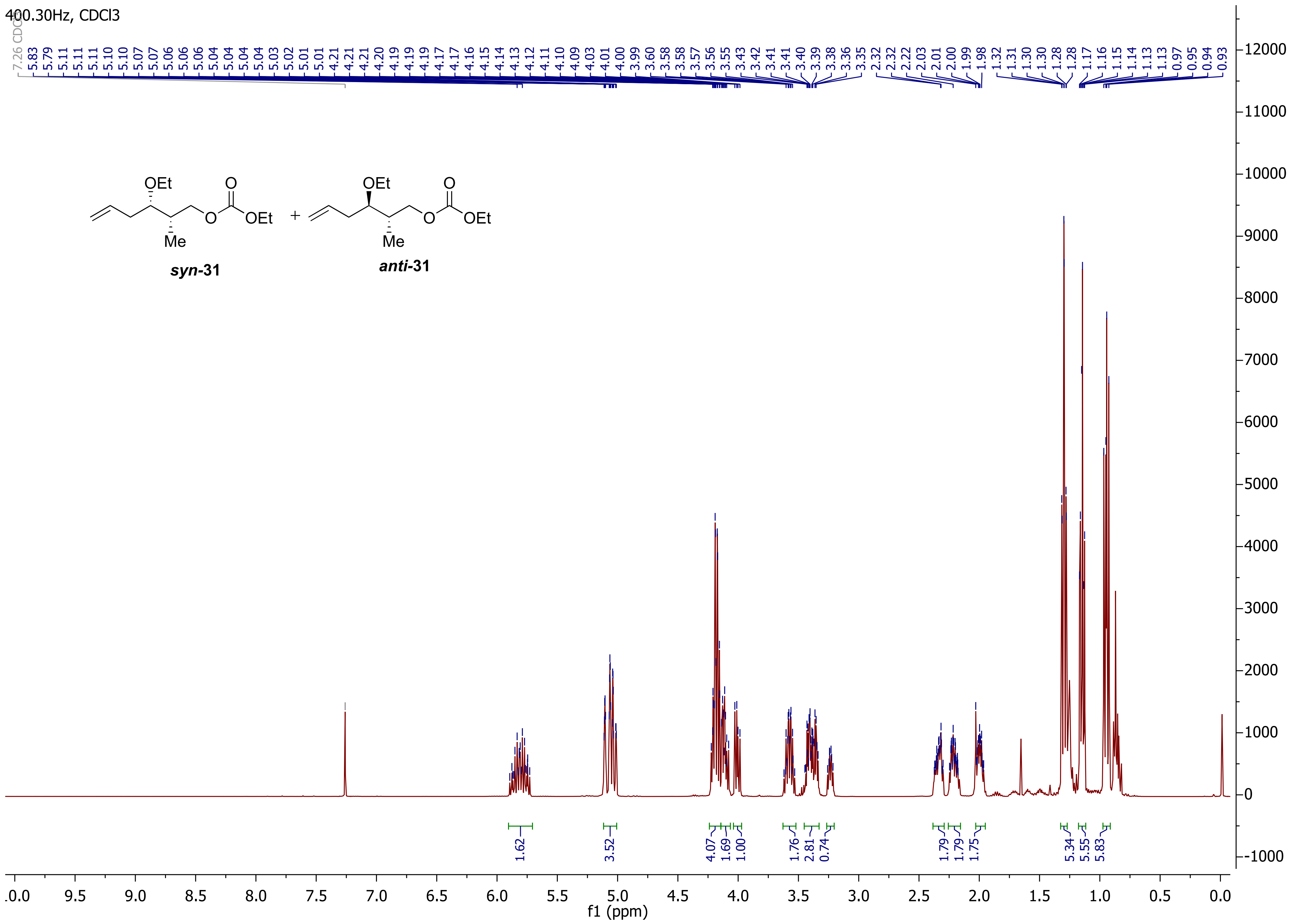
400.30Hz, CDCl3
7.26 CDCl3
5.83 5.79 5.11 5.11 5.11 5.10 5.10 5.07 5.07 5.06 5.06 5.06 5.04 5.04 5.04 5.03 5.02 5.01 5.01 4.21 4.21 4.21 4.20 4.19 4.19 4.19 4.17 4.17 4.16 4.15 4.14 4.13 4.12 4.11 4.10 4.09 4.03 4.01 4.00 3.99 3.60 3.58 3.58 3.57 3.56 3.55 3.43 3.42 3.41 3.41 3.40 3.39 3.38 3.36 3.35 2.32 2.32 2.22 2.03 2.01 2.00 1.99 1.98 1.32 1.31 1.30 1.30 1.28 1.28 1.17 1.16 1.15 1.14 1.13 1.13 0.97 0.95 0.94 0.93
OEt
O
O
OEt
Me
+
OEt
O
O
OEt
Me
syn-31
anti-31
1.62
3.52
4.07
1.69
1.00
1.76
2.81
0.74
1.79
1.79
1.75
5.34
5.55
5.83
f1 (ppm)

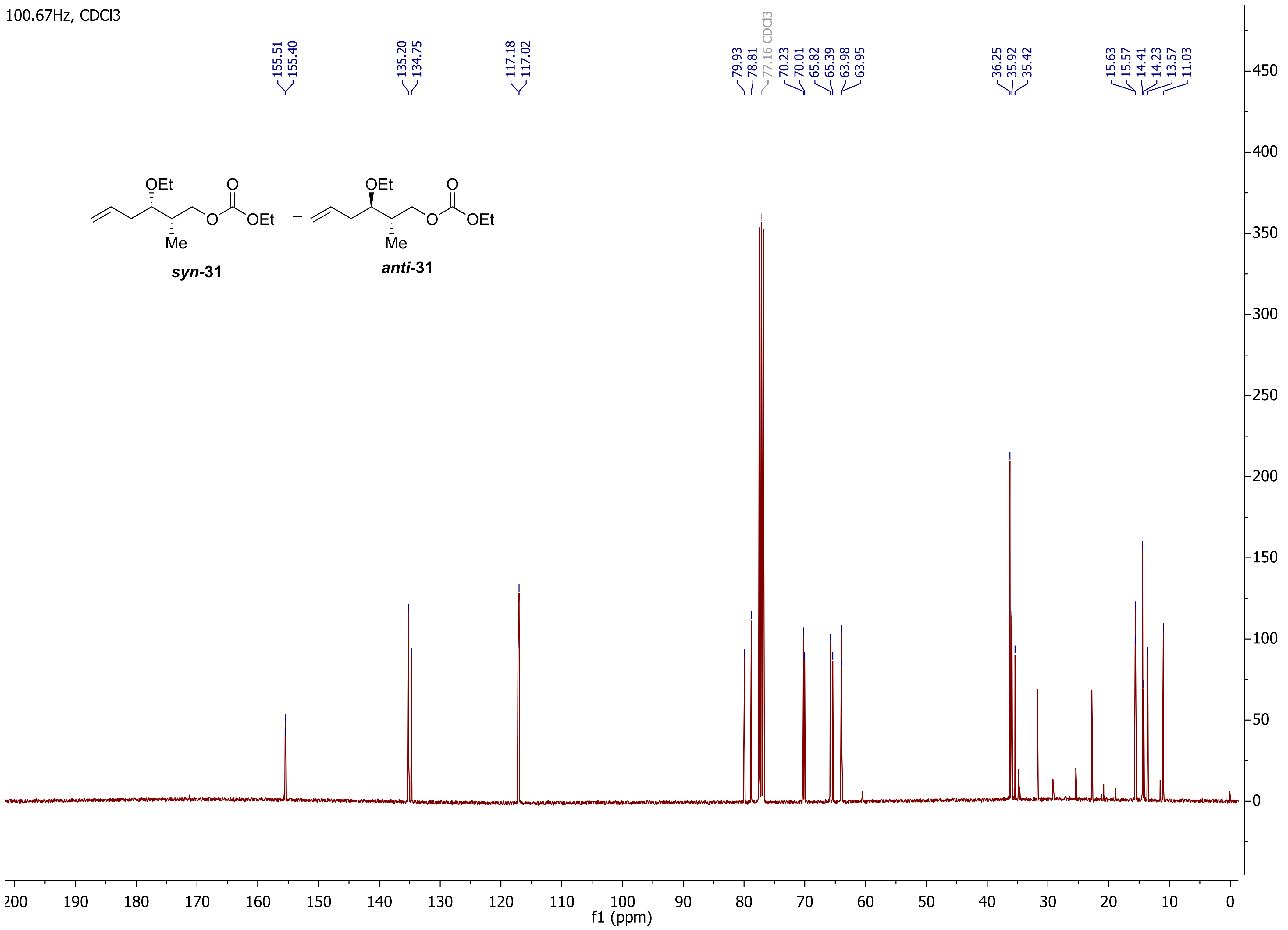

100.67Hz, CDCl3
155.51
155.40
135.20
134.75
117.18
117.02
79.93
78.81
77.16 CDCl3
70.23
70.01
65.82
65.39
63.98
63.95
36.25
35.92
35.42
15.63
15.57
14.41
14.23
13.57
11.03
OEt
O
OEt
Me
syn-31
+
anti-31
f1 (ppm)

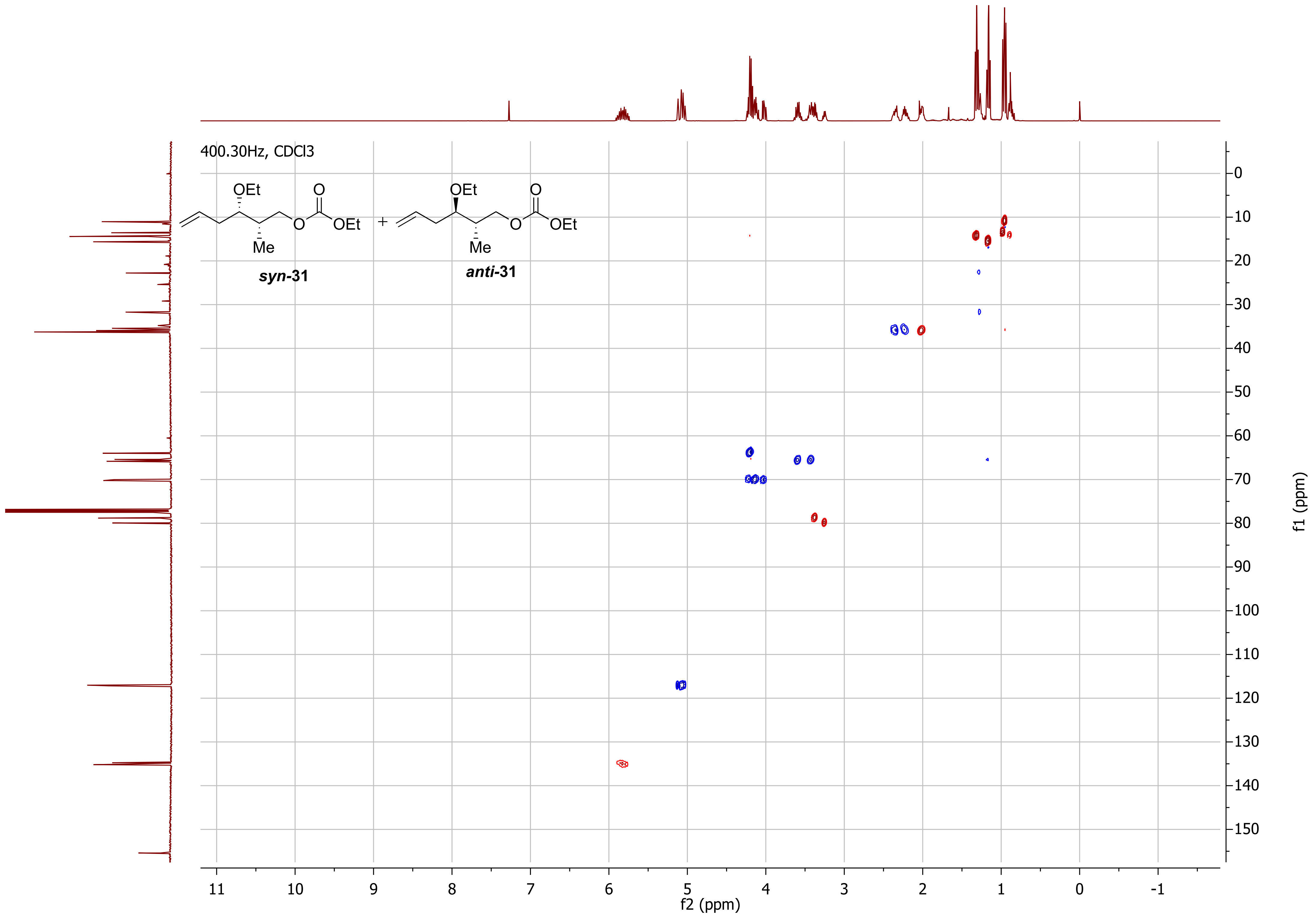

400.30Hz, CDCl3
OEt
O
OEt
Me
syn-31
+
anti-31
f2 (ppm)
f1 (ppm)

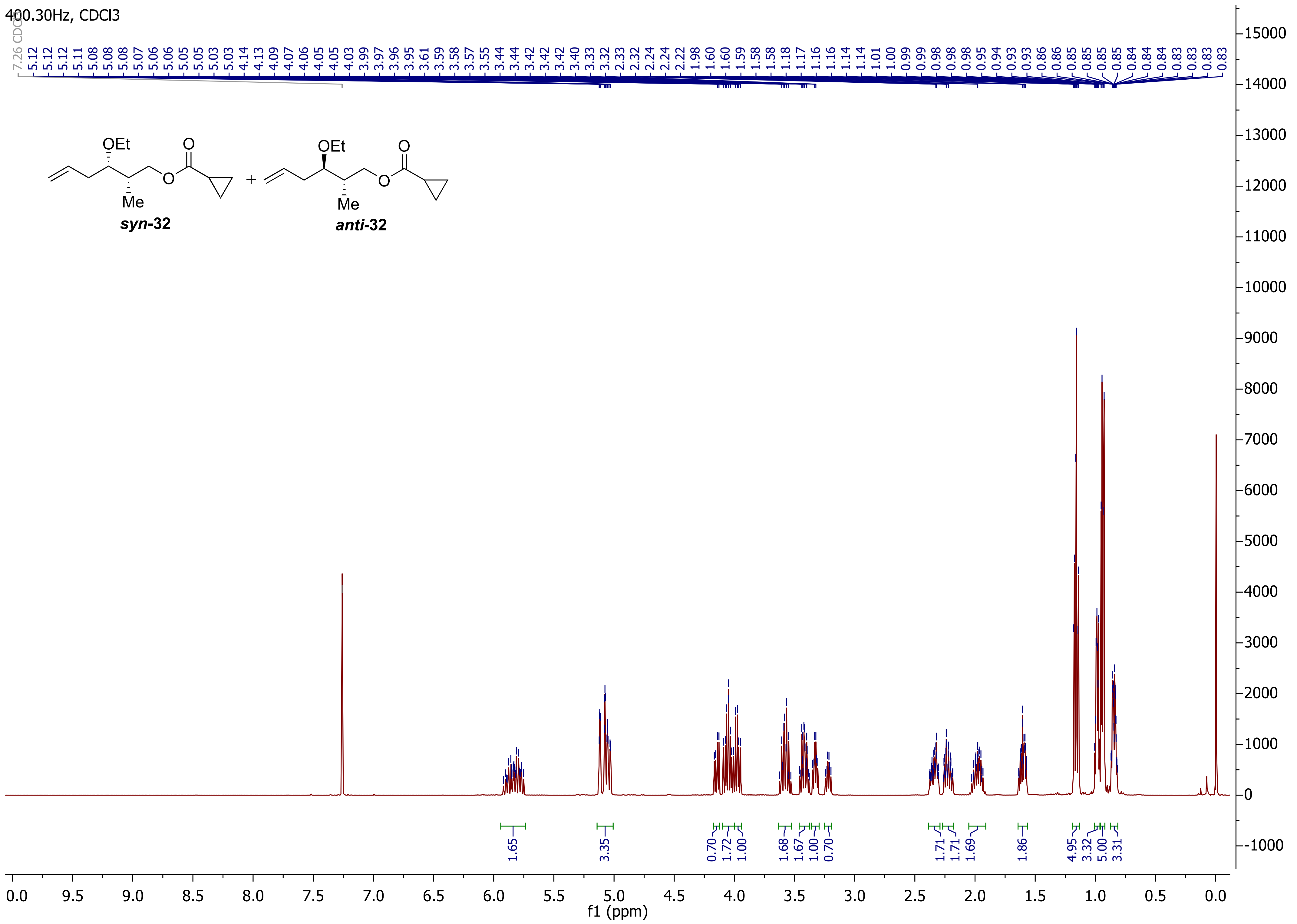

400.30Hz, CDCl3
7.26 CDCl3
5.12 5.12 5.12 5.11 5.08 5.08 5.08 5.07 5.06 5.06 5.05 5.05 5.03 5.03 4.14 4.13 4.09 4.07 4.06 4.05 4.05 4.03 3.99 3.97 3.96 3.95 3.61 3.59 3.58 3.57 3.55 3.44 3.44 3.42 3.42 3.42 3.40 3.33 3.32 2.33 2.32 2.24 2.24 2.22 1.98 1.60 1.60 1.59 1.58 1.58 1.18 1.17 1.16 1.16 1.14 1.14 1.01 1.00 0.99 0.99 0.98 0.98 0.98 0.95 0.94 0.93 0.93 0.86 0.86 0.85 0.85 0.85 0.85 0.84 0.84 0.84 0.83 0.83 0.83 0.83
OEt
O
Me
syn-32
+
OEt
O
Me
anti-32
1.65
3.35
0.70
1.72
1.00
1.68
1.67
1.00
0.70
1.71
1.71
1.69
1.86
4.95
3.32
5.00
3.31
f1 (ppm)

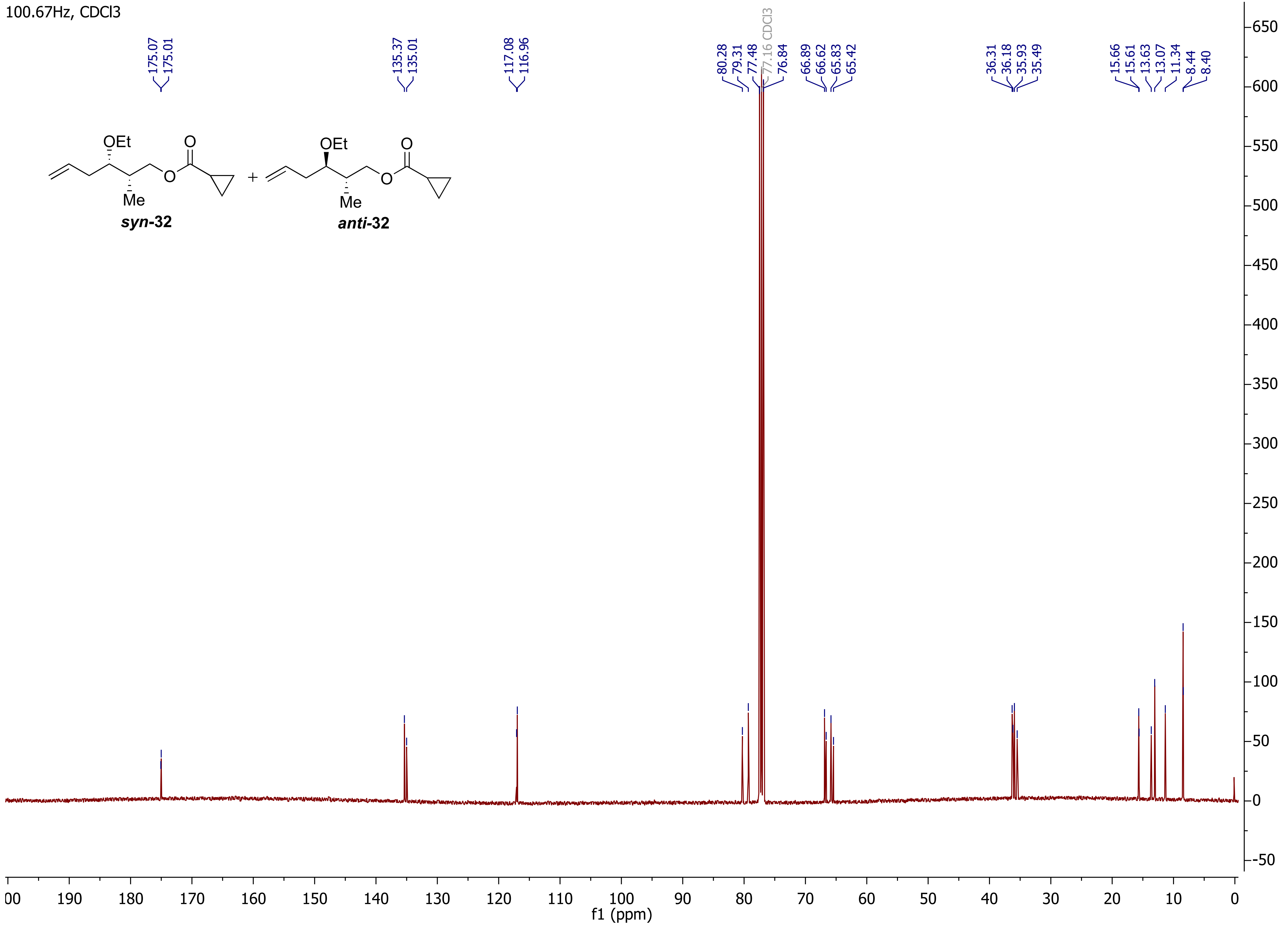
100.67Hz, CDCl3
175.07
175.01
135.37
135.01
117.08
116.96
80.28
79.31
77.48
77.16 CDCl3
76.84
66.89
66.62
65.83
65.42
36.31
36.18
35.93
35.49
15.66
15.61
13.63
13.07
11.34
8.44
8.40
OEt
O
Me
syn-32
+
OEt
O
Me
anti-32
f1 (ppm)

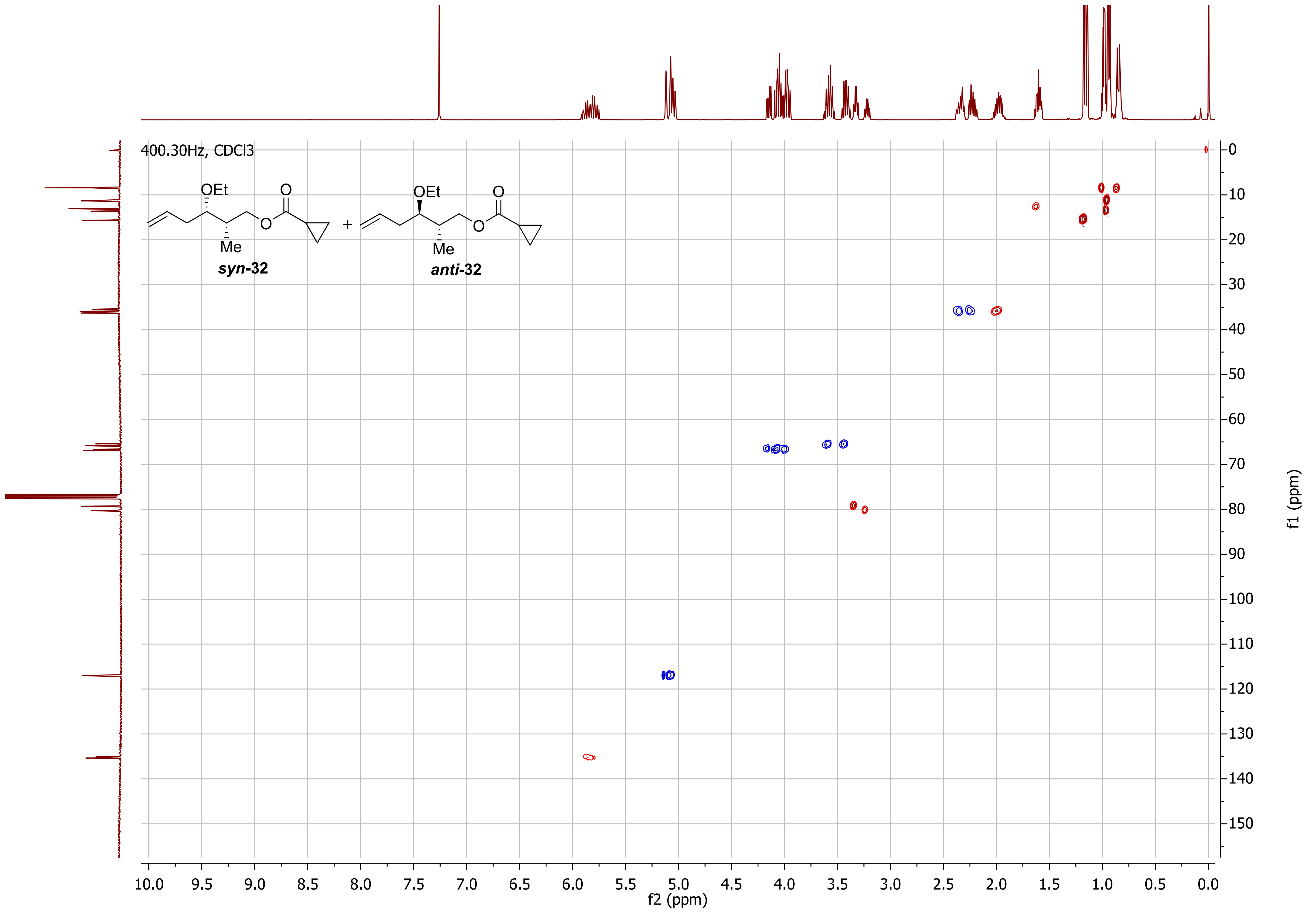
400.30Hz, CDCl3
OEt
O
Me
syn-32
+
anti-32
f2 (ppm)
f1 (ppm)

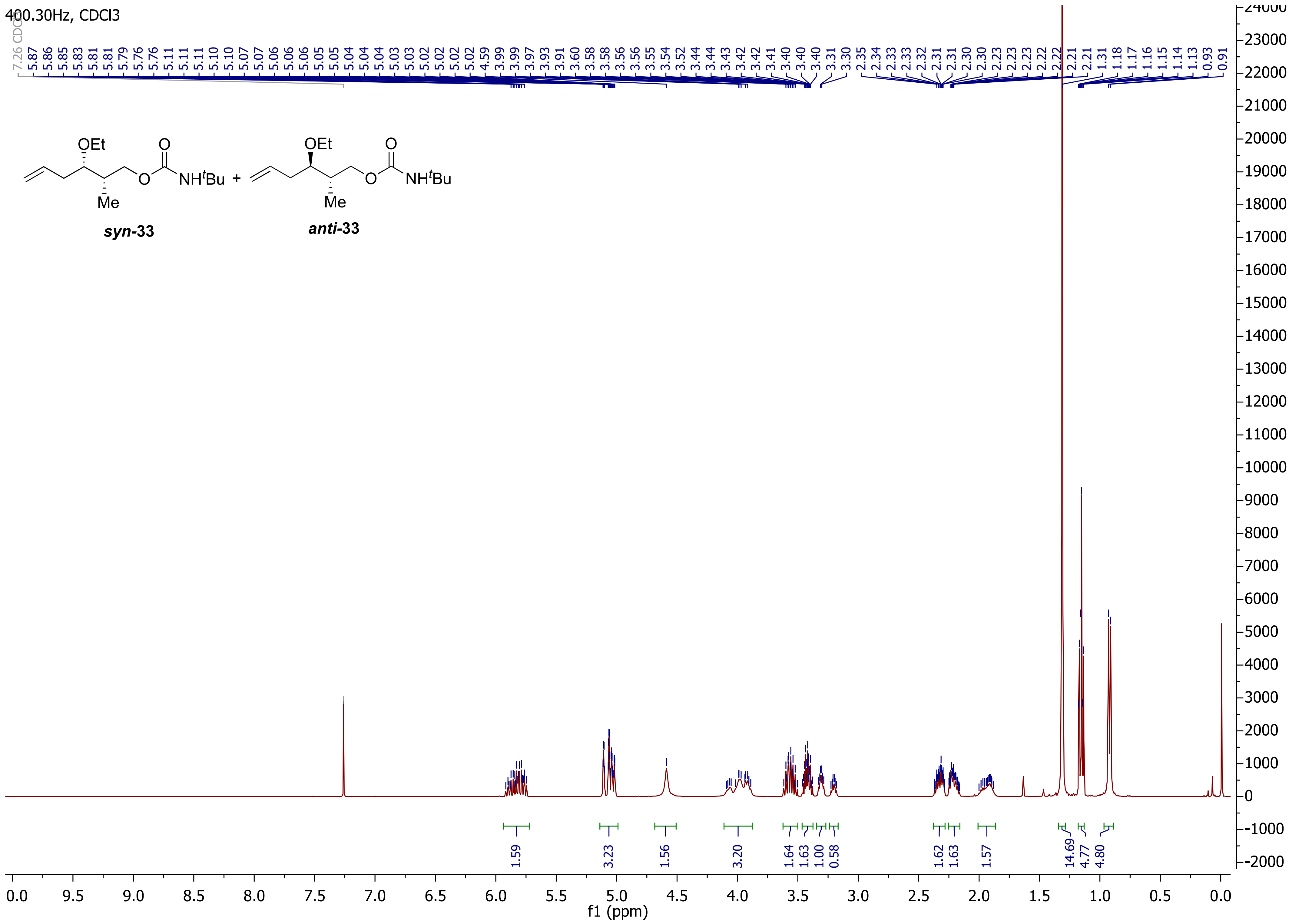
400.30Hz, CDCl3
syn-33
anti-33
f1 (ppm)

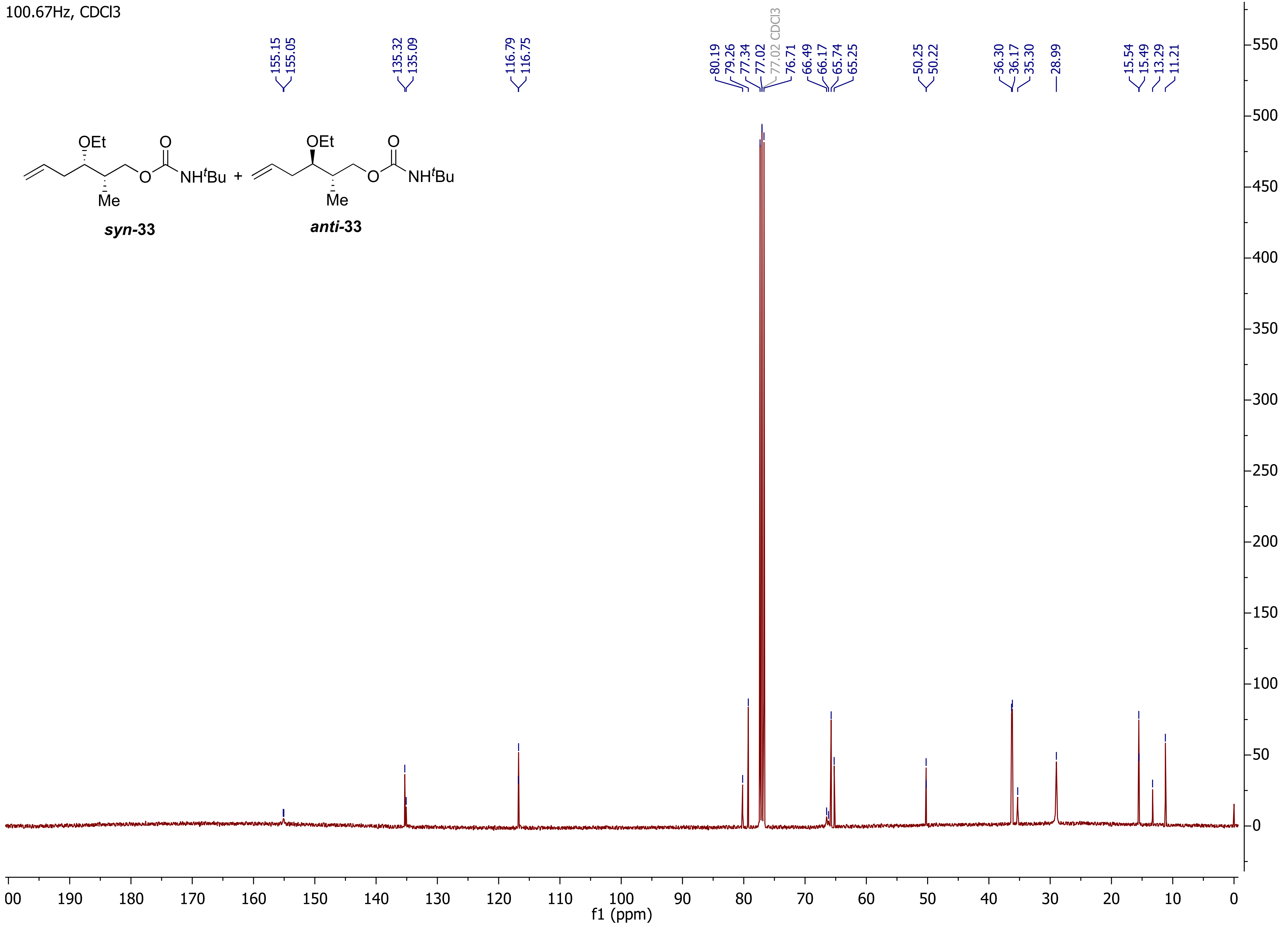
100.67Hz, CDCl3
155.15
155.05
135.32
135.09
116.79
116.75
80.19
79.26
77.34
77.02
77.02 CDCl3
76.71
66.49
66.17
65.74
65.25
50.25
50.22
36.30
36.17
35.30
28.99
15.54
15.49
13.29
11.21
OEt
O
O
NH^{t}Bu
Me
syn-33
anti-33
f1 (ppm)

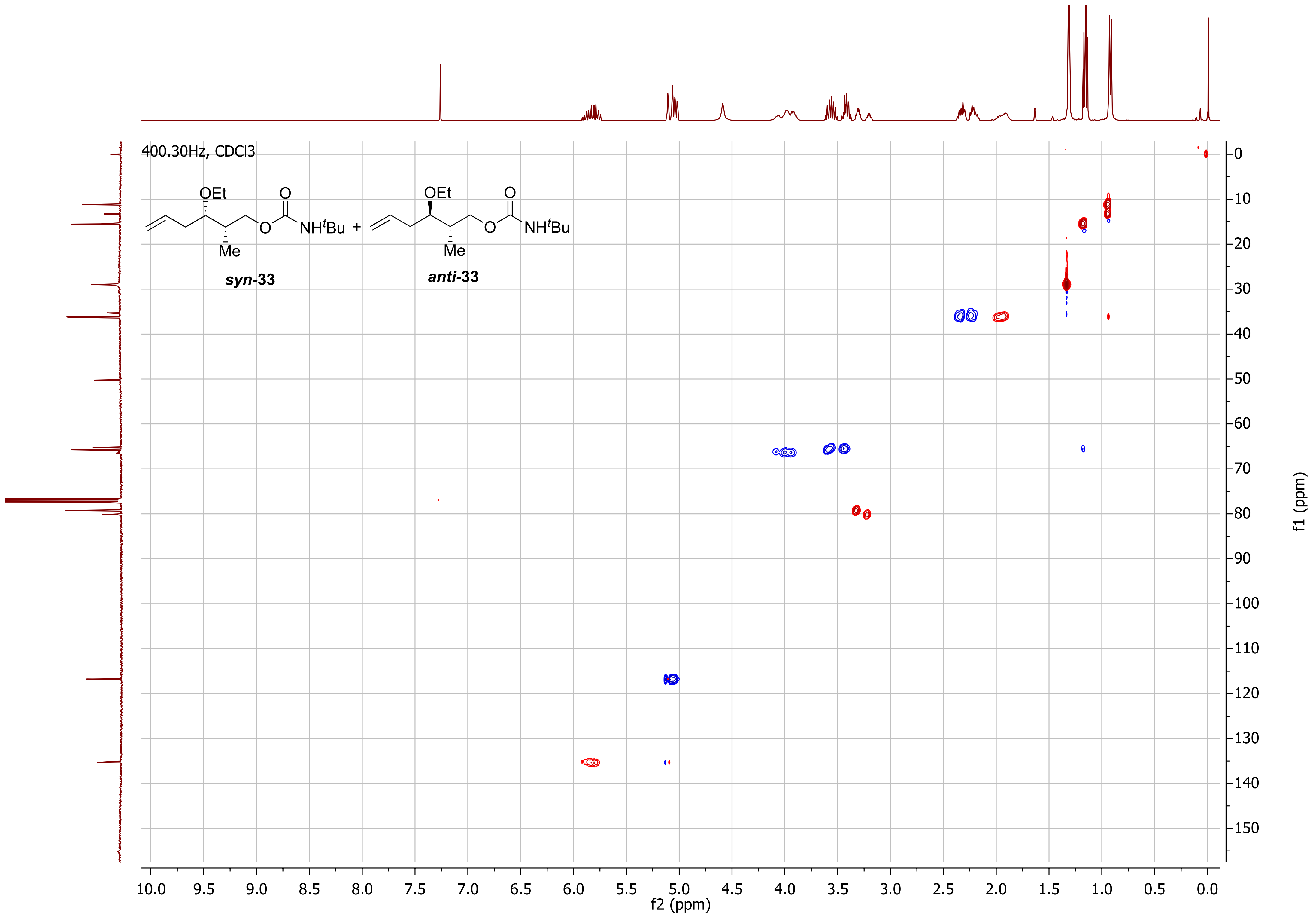
400.30Hz, CDCl3
OEt
O
NH^{t}Bu
+
Me
syn-33
anti-33
f2 (ppm)
f1 (ppm)
10.0
9.5
9.0
8.5
8.0
7.5
7.0
6.5
6.0
5.5
5.0
4.5
4.0
3.5
3.0
2.5
2.0
1.5
1.0
0.5
0.0
0
10
20
30
40
50
60
70
80
90
100
110
120
130
140
150

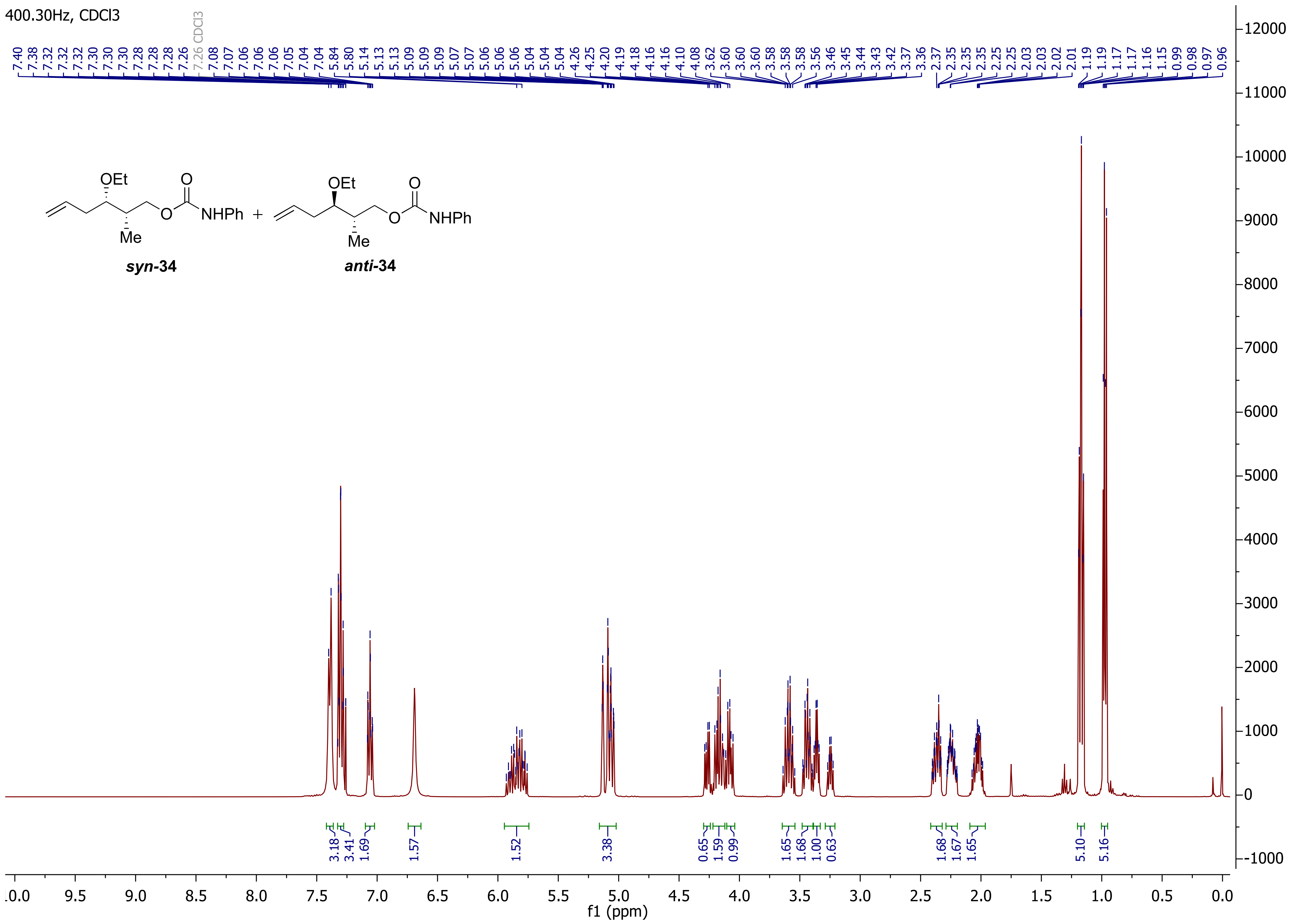
400.30Hz, CDCl3
7.40 7.38 7.32 7.32 7.30 7.30 7.30 7.28 7.28 7.28 7.26 7.26 CDCl3 7.08 7.07 7.06 7.06 7.06 7.05 7.04 7.04 5.84 5.80 5.14 5.13 5.09 5.09 5.09 5.07 5.07 5.06 5.06 5.06 5.04 5.04 5.04 4.26 4.25 4.20 4.19 4.18 4.16 4.16 4.10 4.08 3.62 3.60 3.60 3.58 3.58 3.58 3.56 3.46 3.45 3.44 3.43 3.42 3.37 3.36 2.37 2.35 2.35 2.35 2.25 2.25 2.03 2.03 2.02 2.01 1.19 1.19 1.17 1.17 1.16 1.15 0.99 0.98 0.97 0.96
OEt
O
NHPh
Me
syn-34
+
anti-34
3.18 3.41 1.69 1.57 1.52 3.38 0.65 1.59 0.99 1.65 1.68 1.00 0.63 1.68 1.67 1.65 5.10 5.16
f1 (ppm)

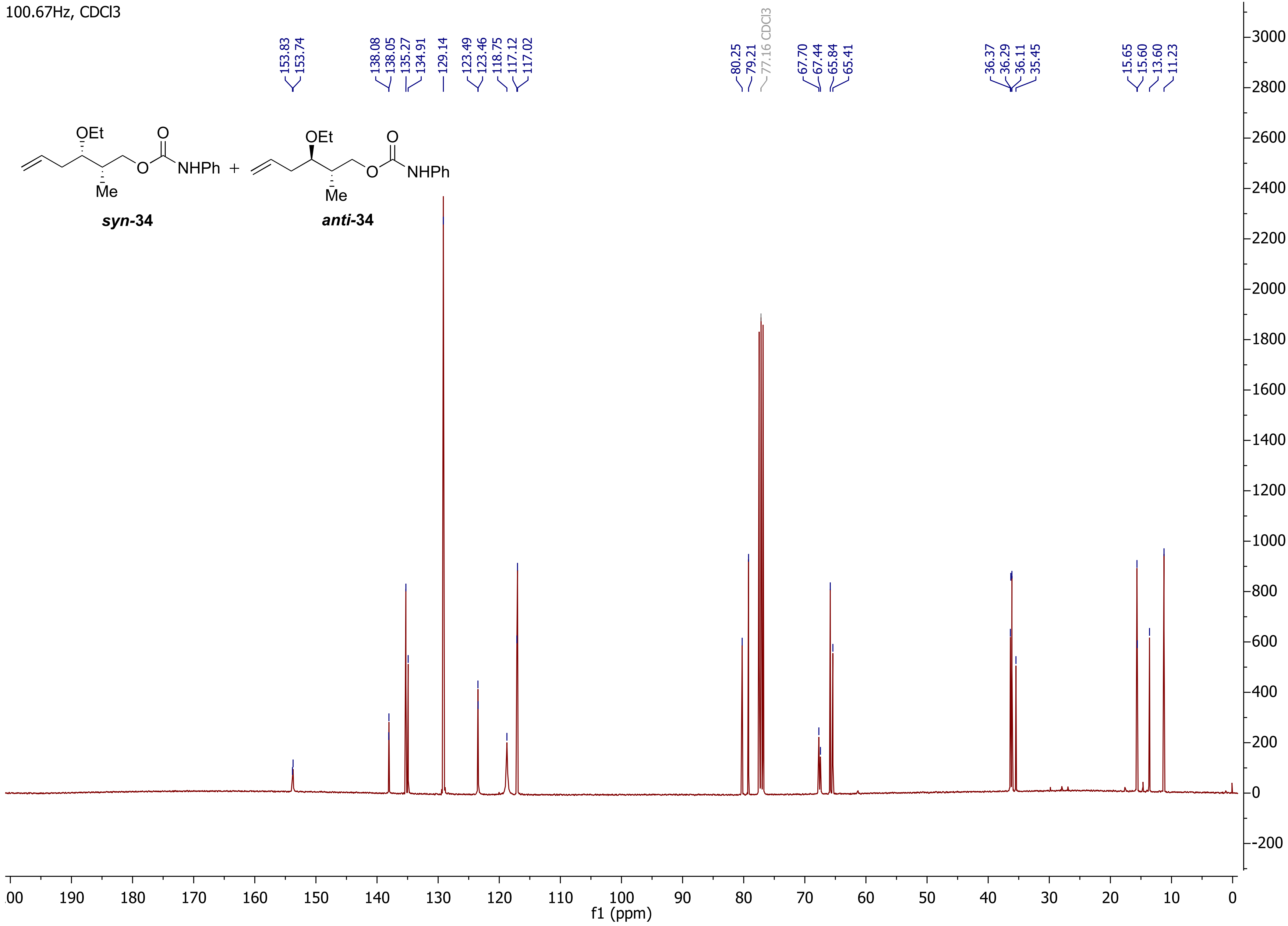

100.67Hz, CDCl3
153.83
153.74
138.08
138.05
135.27
134.91
129.14
123.49
123.46
118.75
117.12
117.02
80.25
79.21
77.16 CDCl3
67.70
67.44
65.84
65.41
36.37
36.29
36.11
35.45
15.65
15.60
13.60
11.23
OEt
O
O
NHPh
Me
+
syn-34
anti-34
f1 (ppm)

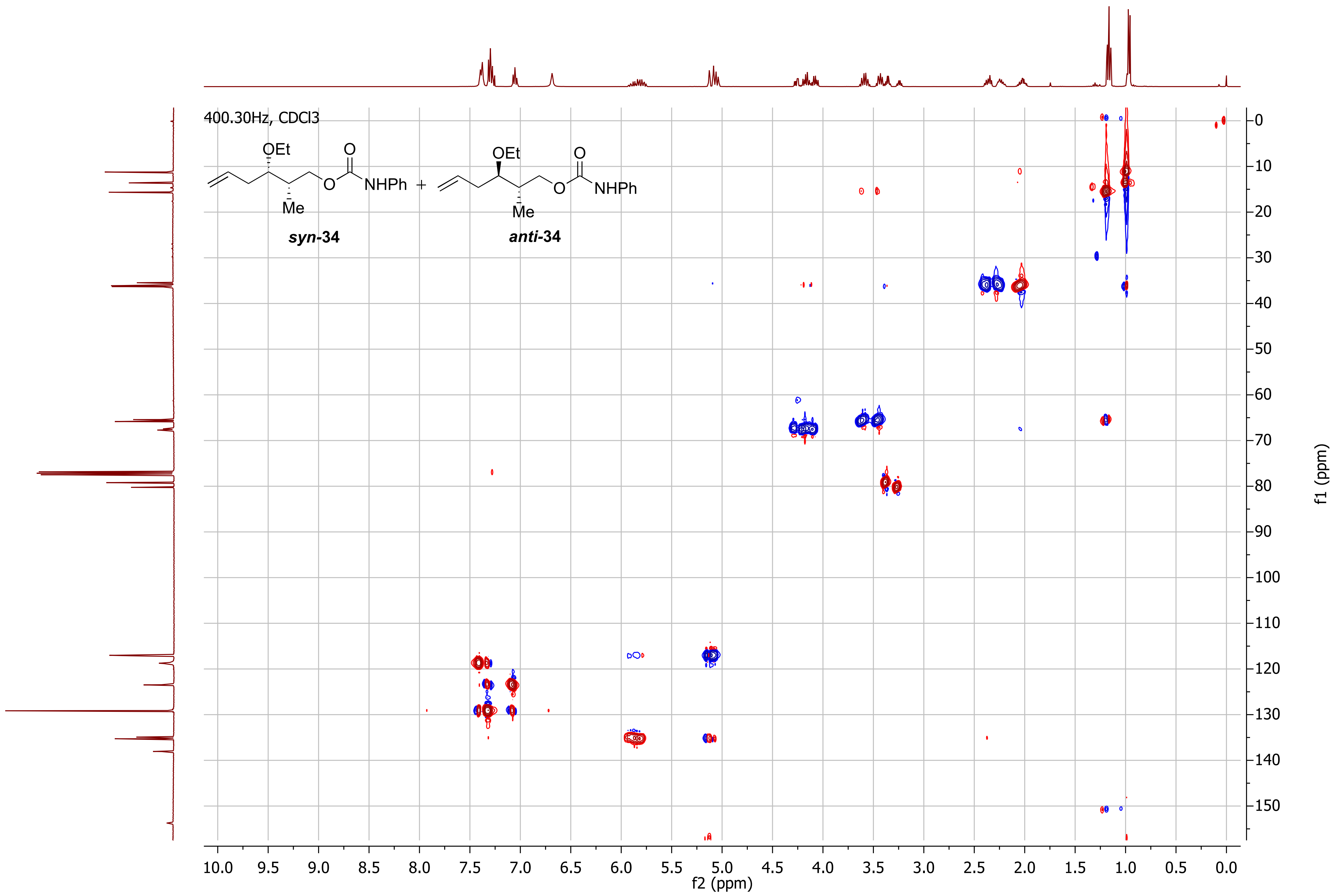
400.30Hz, CDCl3
OEt
O
NHPh
Me
syn-34
anti-34
f1 (ppm)
f2 (ppm)

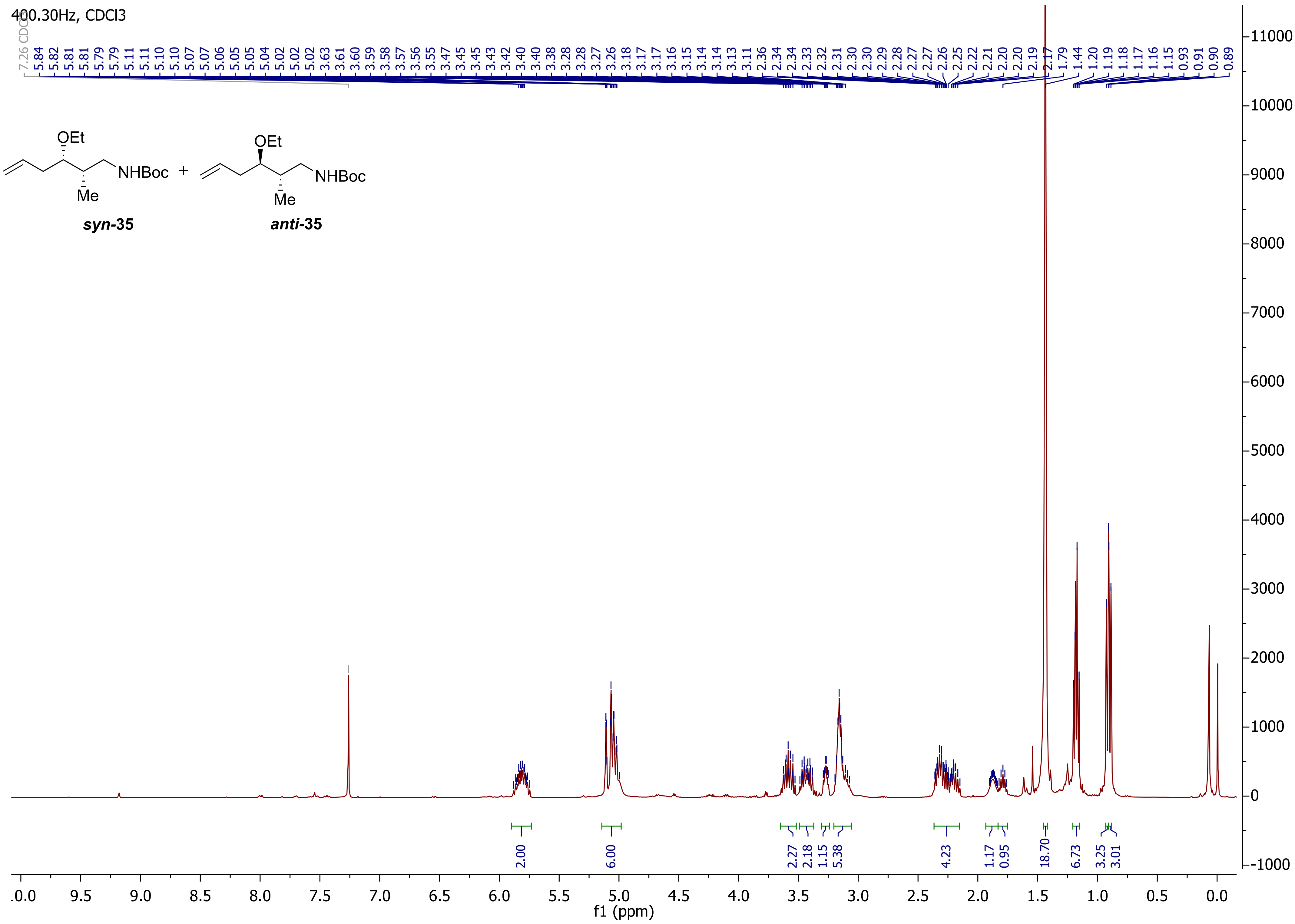
400.30Hz, CDCl3
OEt
NHBoc
Me
syn-35
anti-35
f1 (ppm)

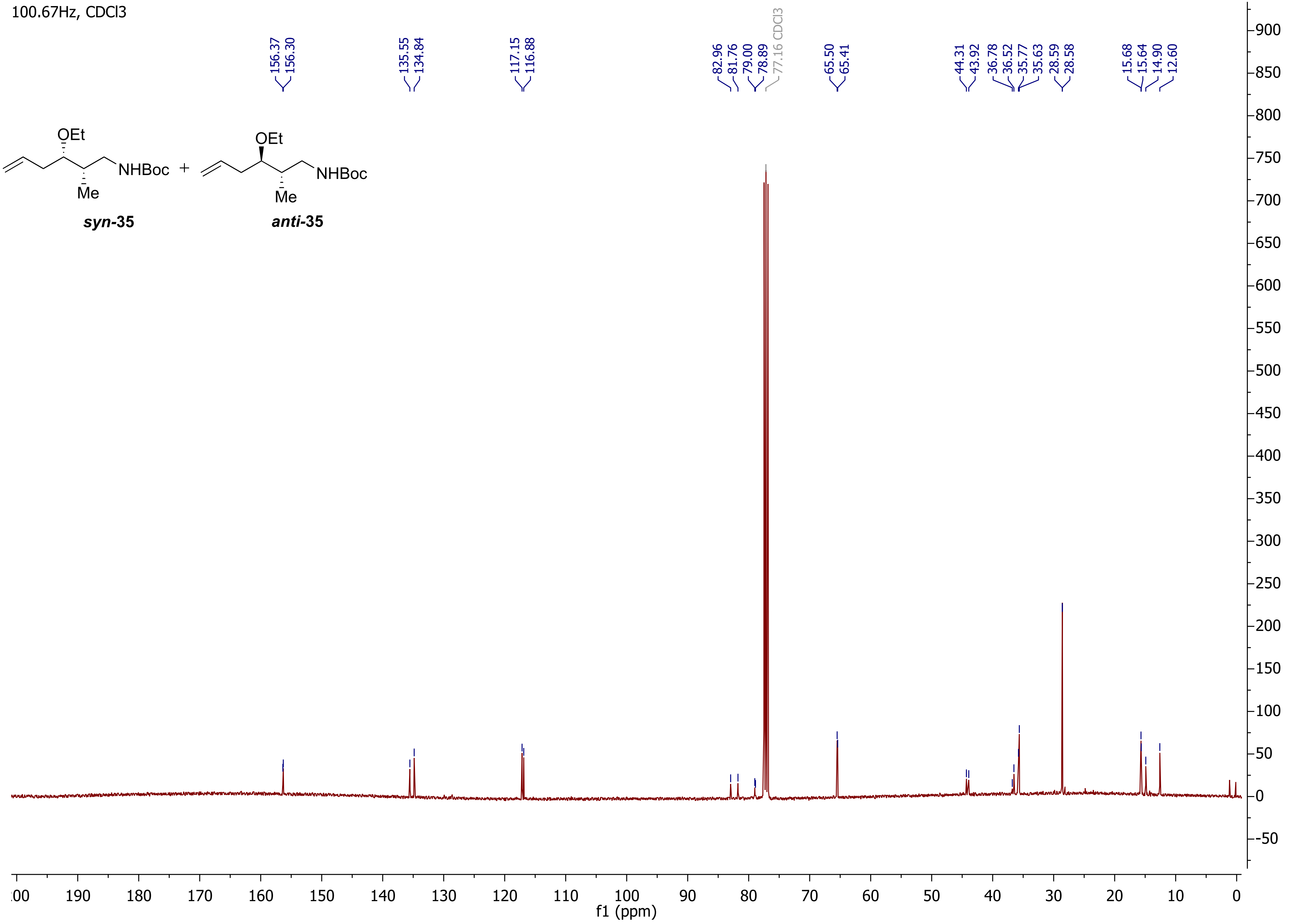
100.67Hz, CDCl3
OEt
NHBoc
Me
+
OEt
NHBoc
Me
syn-35
anti-35
156.37
156.30
135.55
134.84
117.15
116.88
82.96
81.76
79.00
78.89
77.16 CDCl3
65.50
65.41
44.31
43.92
36.78
36.52
35.77
35.63
28.59
28.58
15.68
15.64
14.90
12.60
f1 (ppm)

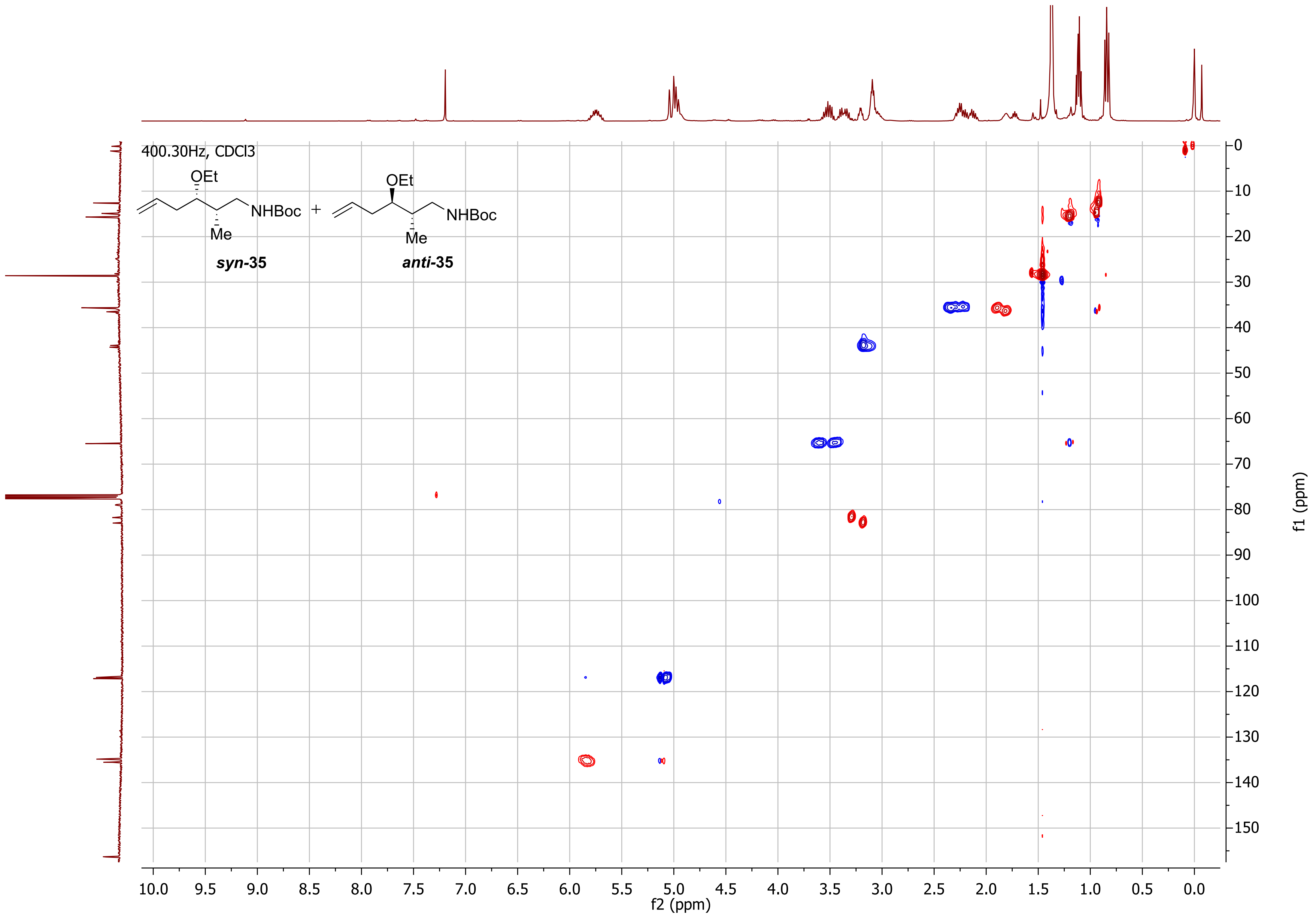
400.30Hz, CDCl3
OEt
NHBoc
Me
syn-35
OEt
NHBoc
Me
anti-35
f2 (ppm)
f1 (ppm)

400.30Hz, CDCl3

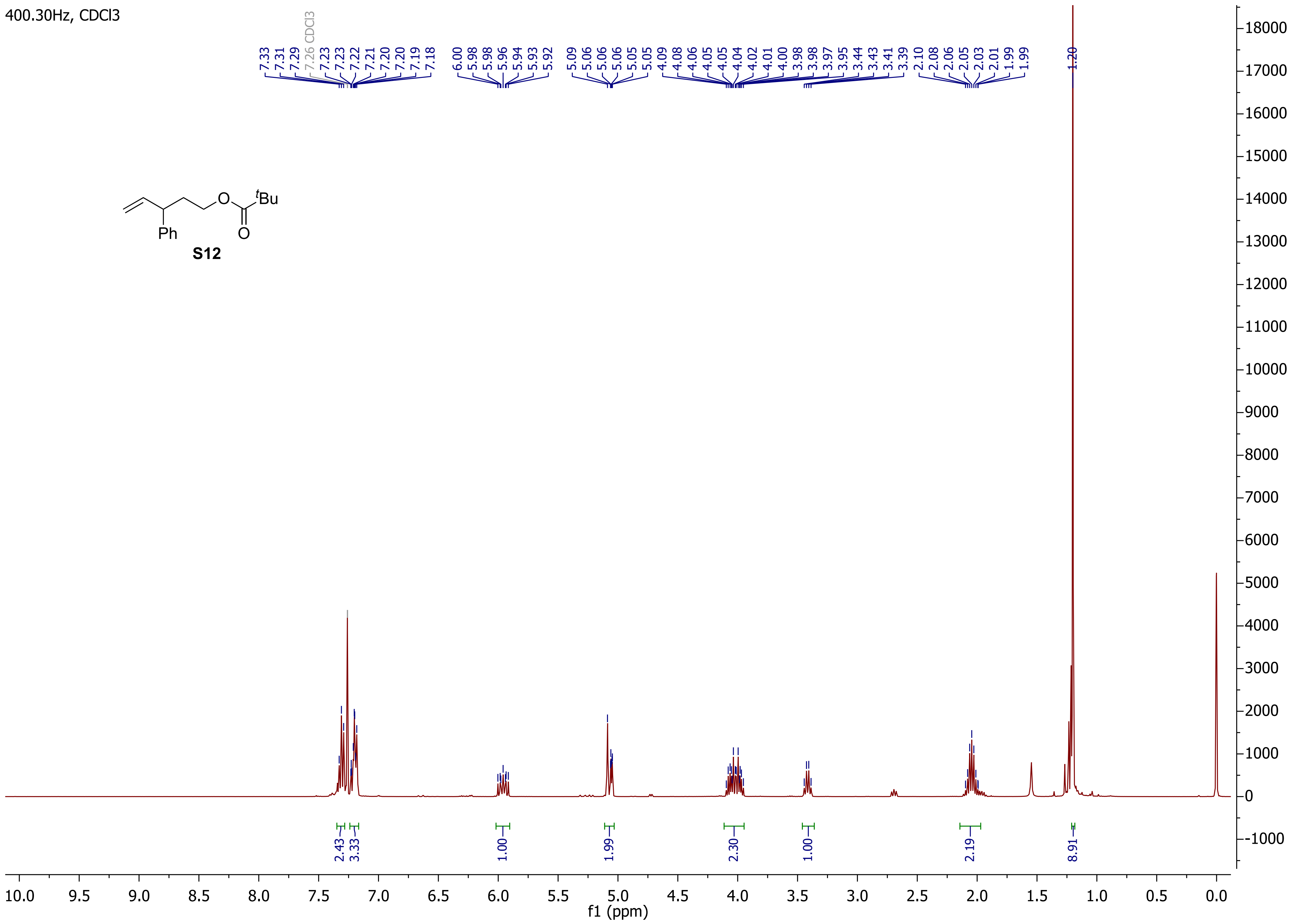

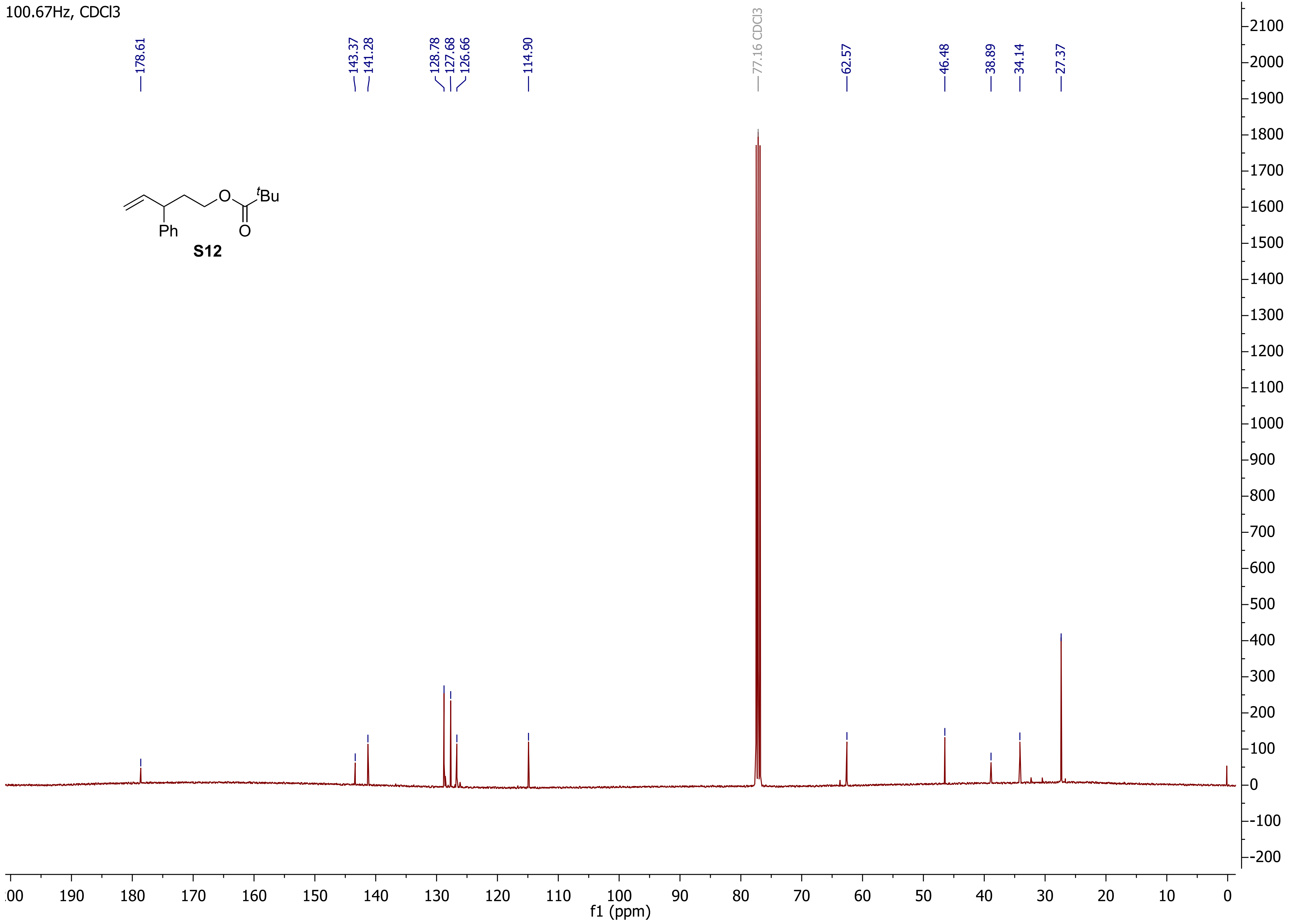

100.67Hz, CDCl3
178.61
143.37
141.28
128.78
127.68
126.66
114.90
77.16 CDCl3
62.57
46.48
38.89
34.14
27.37
tBu
O
O
Ph
S12
f1 (ppm)

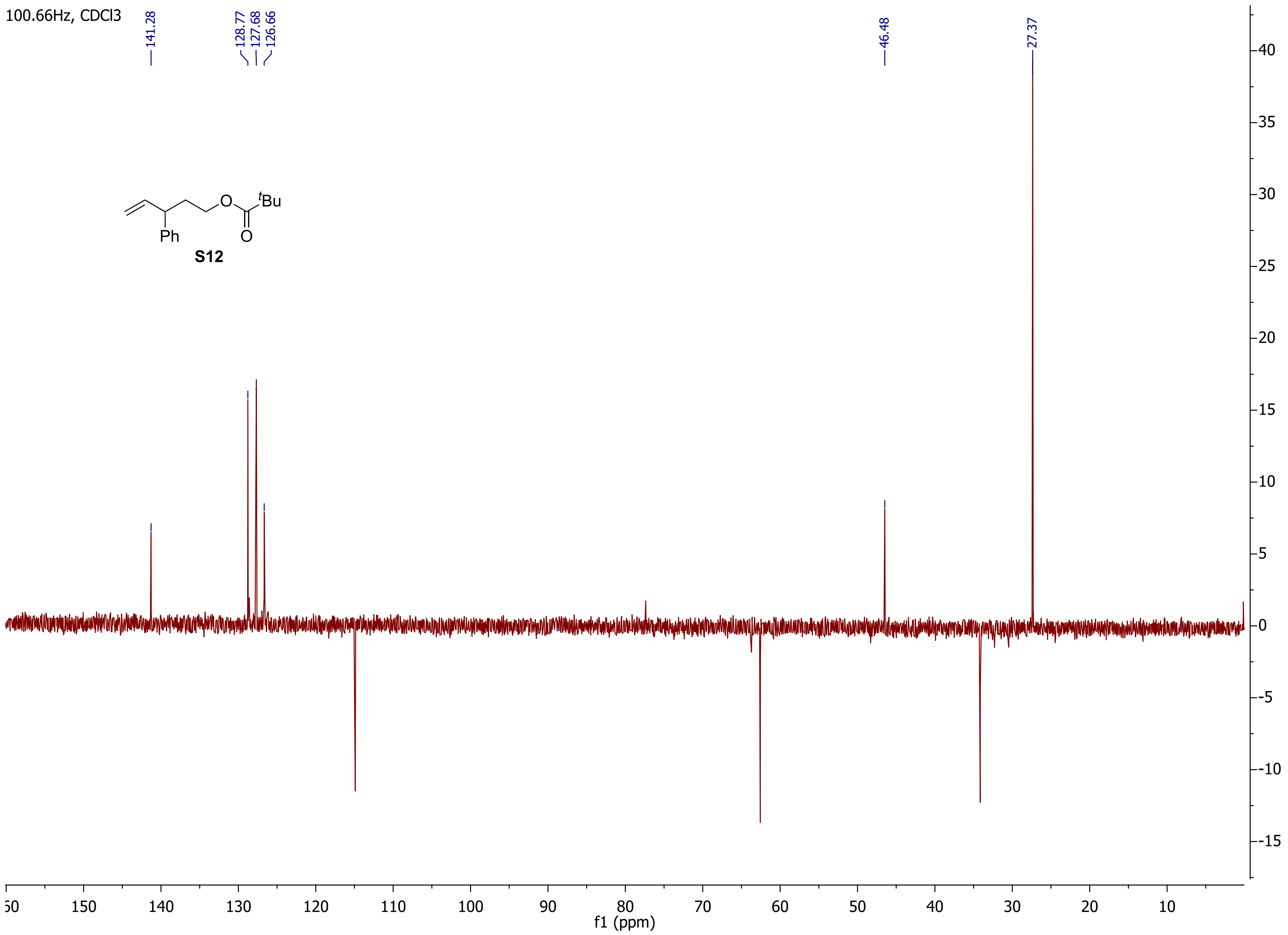

100.66Hz, CDCl3
141.28
128.77
127.68
126.66
46.48
27.37
O
tBu
Ph
O
S12
f1 (ppm)

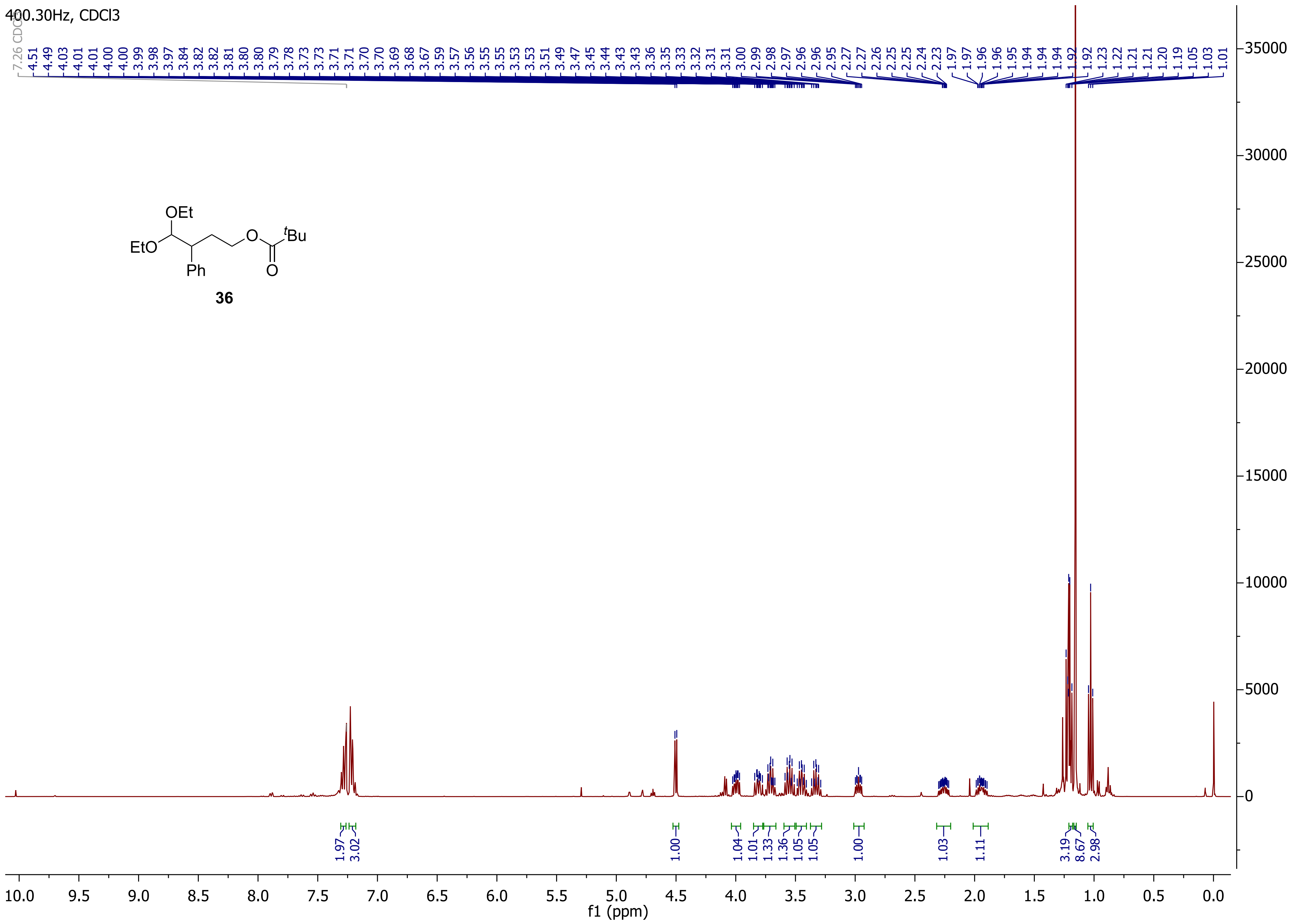

400.30Hz, CDCl3
7.26 CDCl3
4.51 4.49 4.03 4.01 4.01 4.00 4.00 3.99 3.98 3.97 3.84 3.82 3.82 3.81 3.80 3.80 3.79 3.78 3.73 3.73 3.71 3.71 3.70 3.69 3.68 3.67 3.59 3.57 3.56 3.55 3.55 3.53 3.53 3.51 3.49 3.47 3.45 3.44 3.43 3.43 3.36 3.35 3.33 3.32 3.31 3.31 3.00 2.99 2.98 2.97 2.96 2.96 2.95 2.27 2.27 2.26 2.25 2.25 2.24 2.23 1.97 1.97 1.96 1.96 1.95 1.94 1.94 1.94 1.92 1.92 1.23 1.22 1.21 1.21 1.20 1.19 1.05 1.03 1.01
OEt
EtO
O
tBu
O
Ph
36
1.97 3.02 1.00 1.04 1.01 1.33 1.36 1.05 1.05 1.00 1.03 1.11 3.19 8.67 2.98
f1 (ppm)

100.67Hz, CDCl3

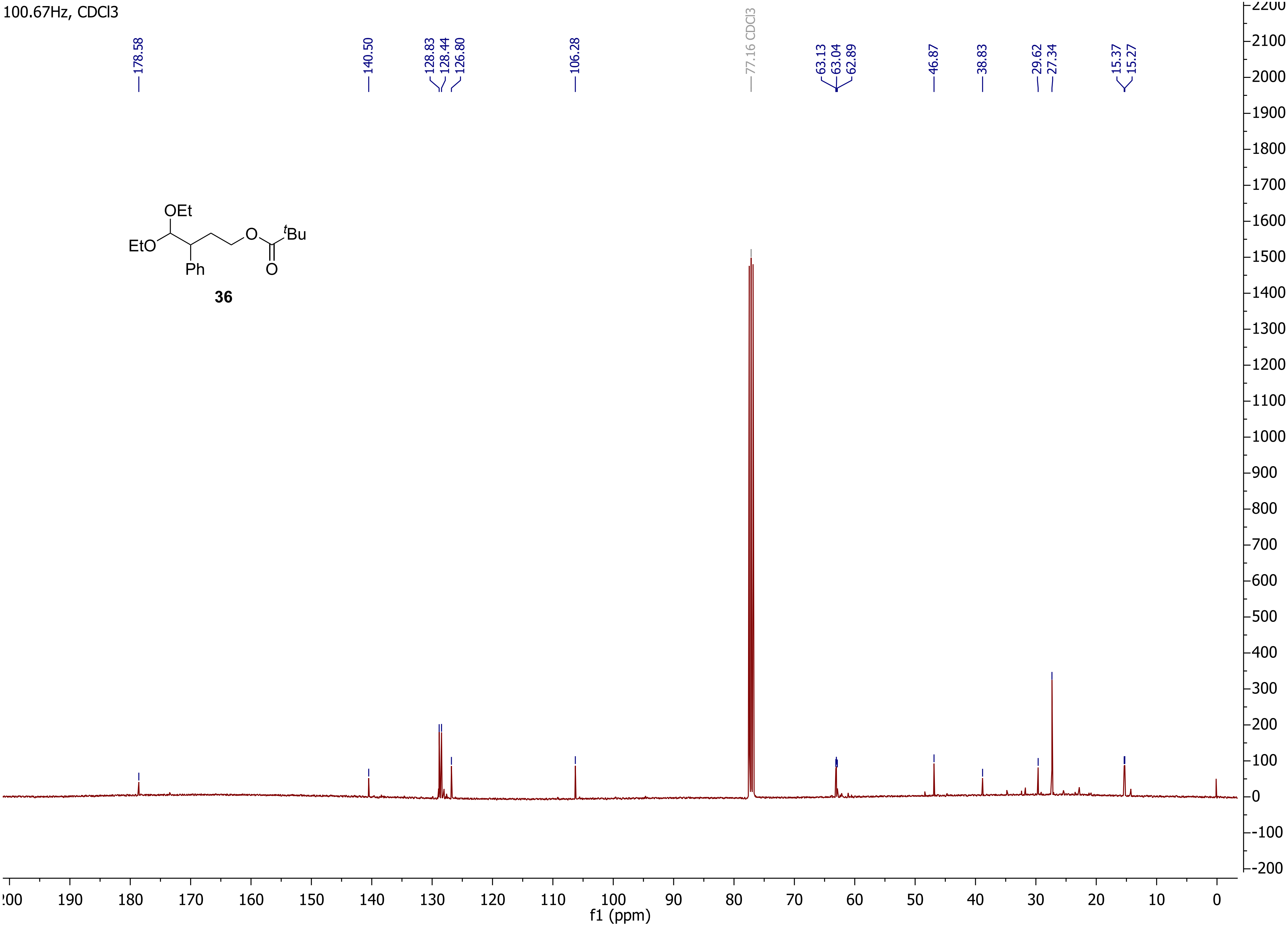

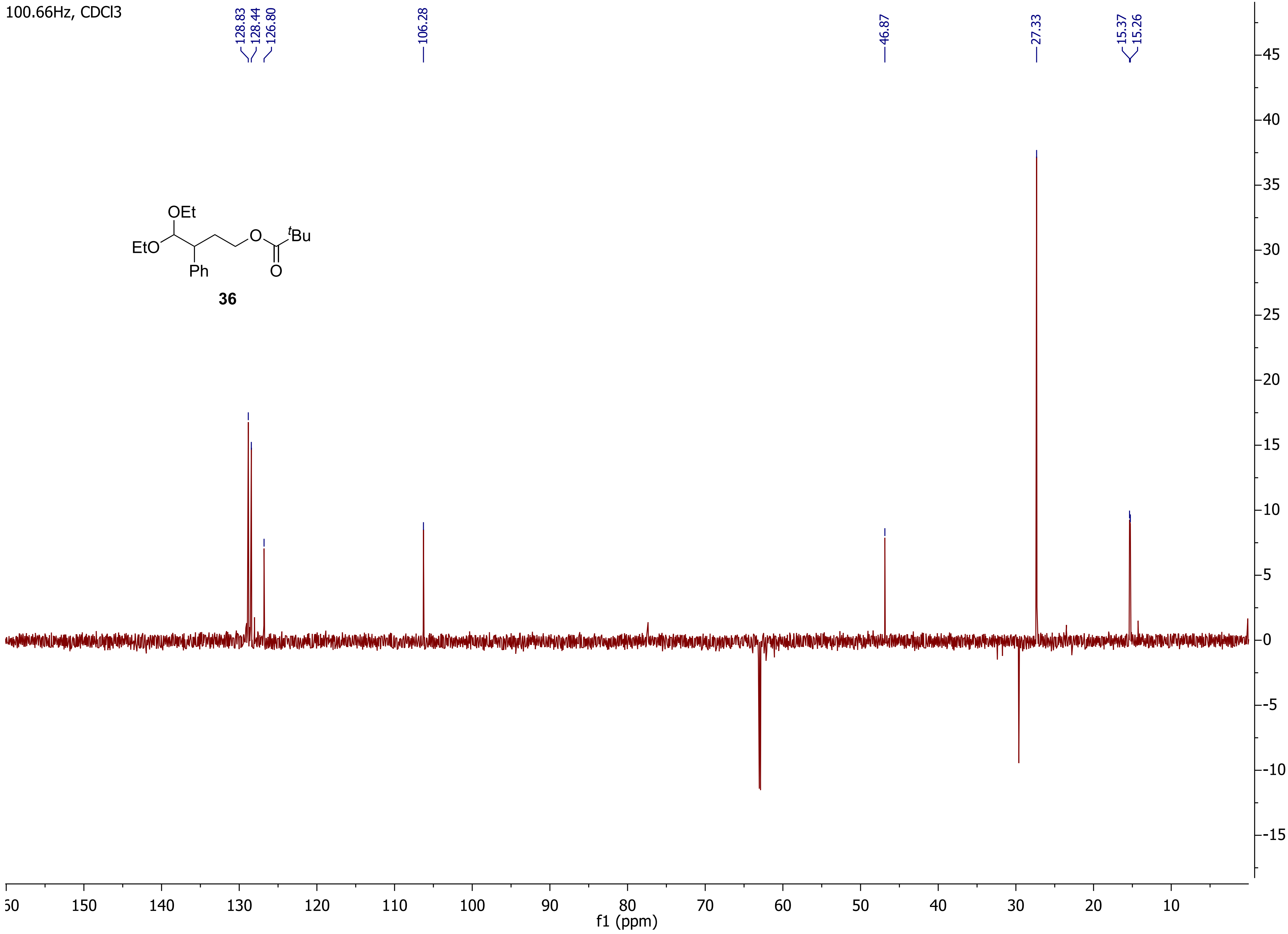
100.66Hz, CDCl3
128.83
128.44
126.80
106.28
46.87
27.33
15.37
15.26
OEt
EtO
O
tBu
Ph
O
36
f1 (ppm)

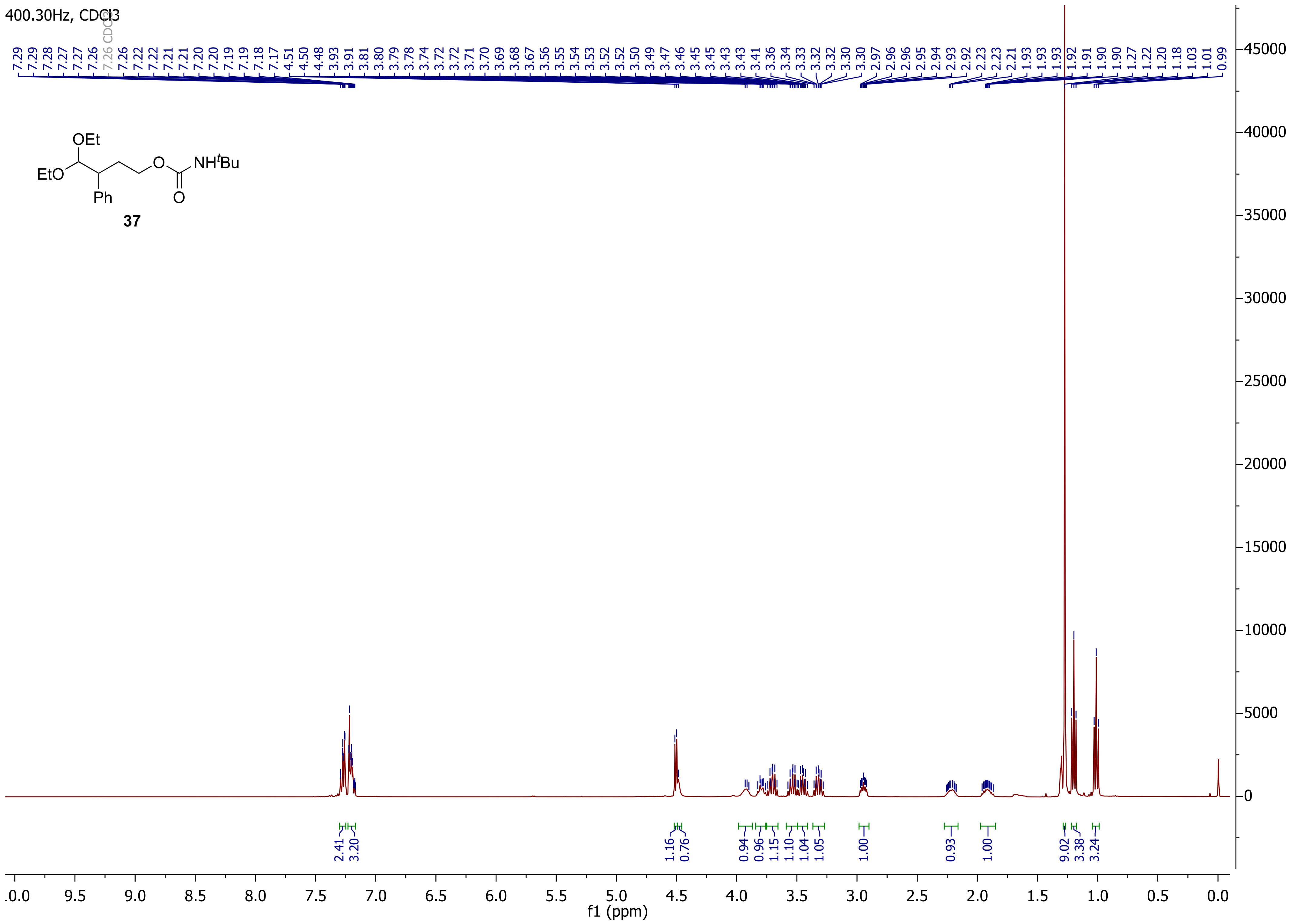
400.30Hz, CDCl3
7.29 7.29 7.28 7.27 7.27 7.26 7.26 CDCl3 7.26 7.22 7.22 7.21 7.21 7.20 7.20 7.19 7.19 7.18 7.17 4.51 4.50 4.48 3.93 3.91 3.81 3.80 3.79 3.78 3.74 3.72 3.72 3.71 3.70 3.69 3.68 3.67 3.56 3.55 3.54 3.53 3.52 3.52 3.50 3.49 3.47 3.46 3.45 3.45 3.43 3.43 3.41 3.36 3.34 3.33 3.32 3.32 3.30 3.30 2.97 2.96 2.96 2.95 2.94 2.93 2.92 2.23 2.23 2.21 1.93 1.93 1.93 1.92 1.91 1.90 1.90 1.27 1.22 1.20 1.18 1.03 1.01 0.99
OEt
EtO
Ph
O
NH^{t}Bu
O
37
2.41 3.20 1.16 0.76 0.94 0.96 1.15 1.10 1.04 1.05 1.00 0.93 1.00 9.02 3.38 3.24
45000 40000 35000 30000 25000 20000 15000 10000 5000 0
.0.0 9.5 9.0 8.5 8.0 7.5 7.0 6.5 6.0 5.5 5.0 4.5 4.0 3.5 3.0 2.5 2.0 1.5 1.0 0.5 0.0
f1 (ppm)

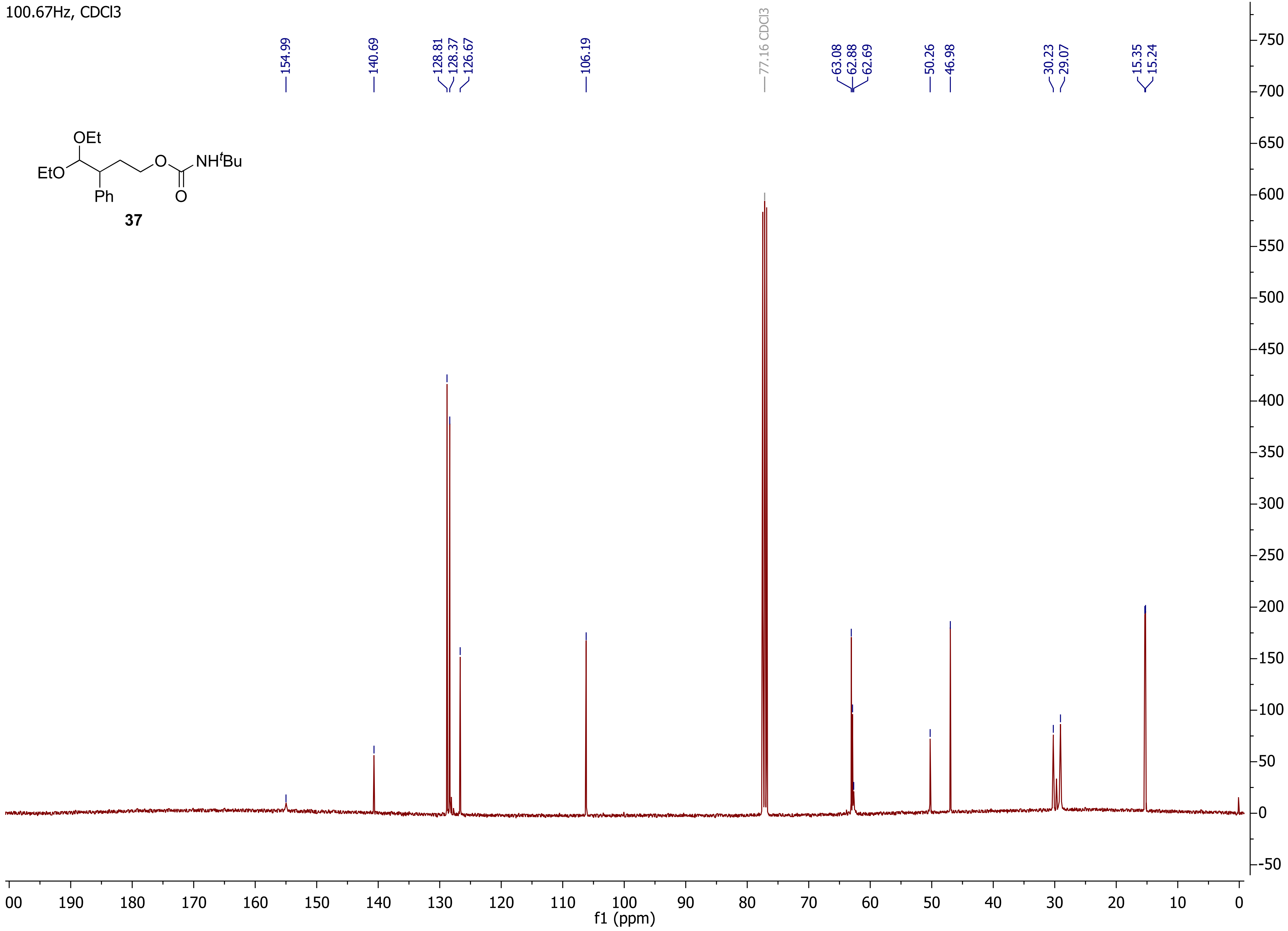
100.67Hz, CDCl3
154.99
140.69
128.81
128.37
126.67
106.19
77.16 CDCl3
63.08
62.88
62.69
50.26
46.98
30.23
29.07
15.35
15.24
OEt
EtO
Ph
O
NH^{t}Bu
O
37
f1 (ppm)

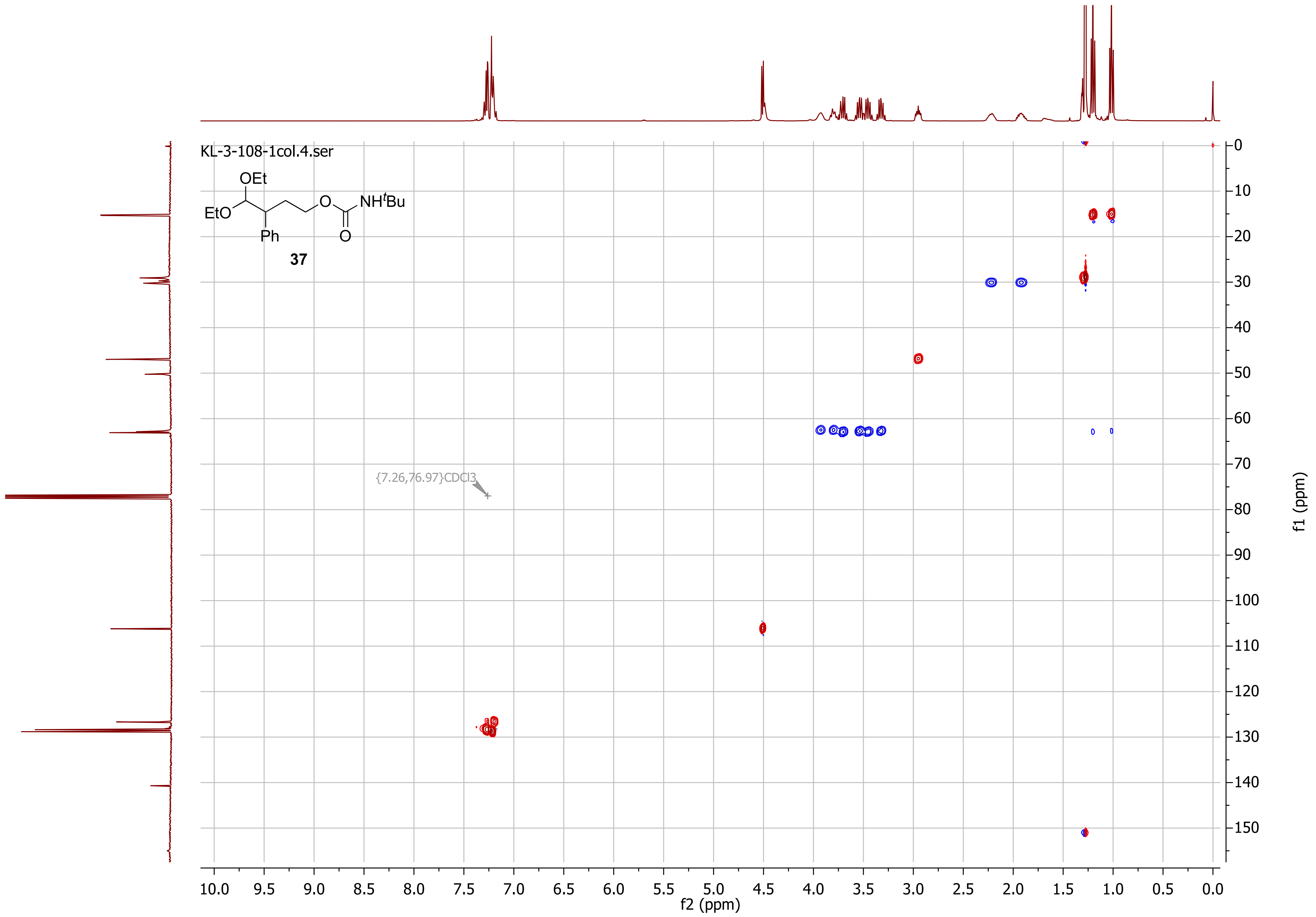
KL-3-108-1col.4.ser
OEt
EtO
O
NH^{t}Bu
Ph
O
37
{7.26,76.97}CDCl3
f2 (ppm)
f1 (ppm)

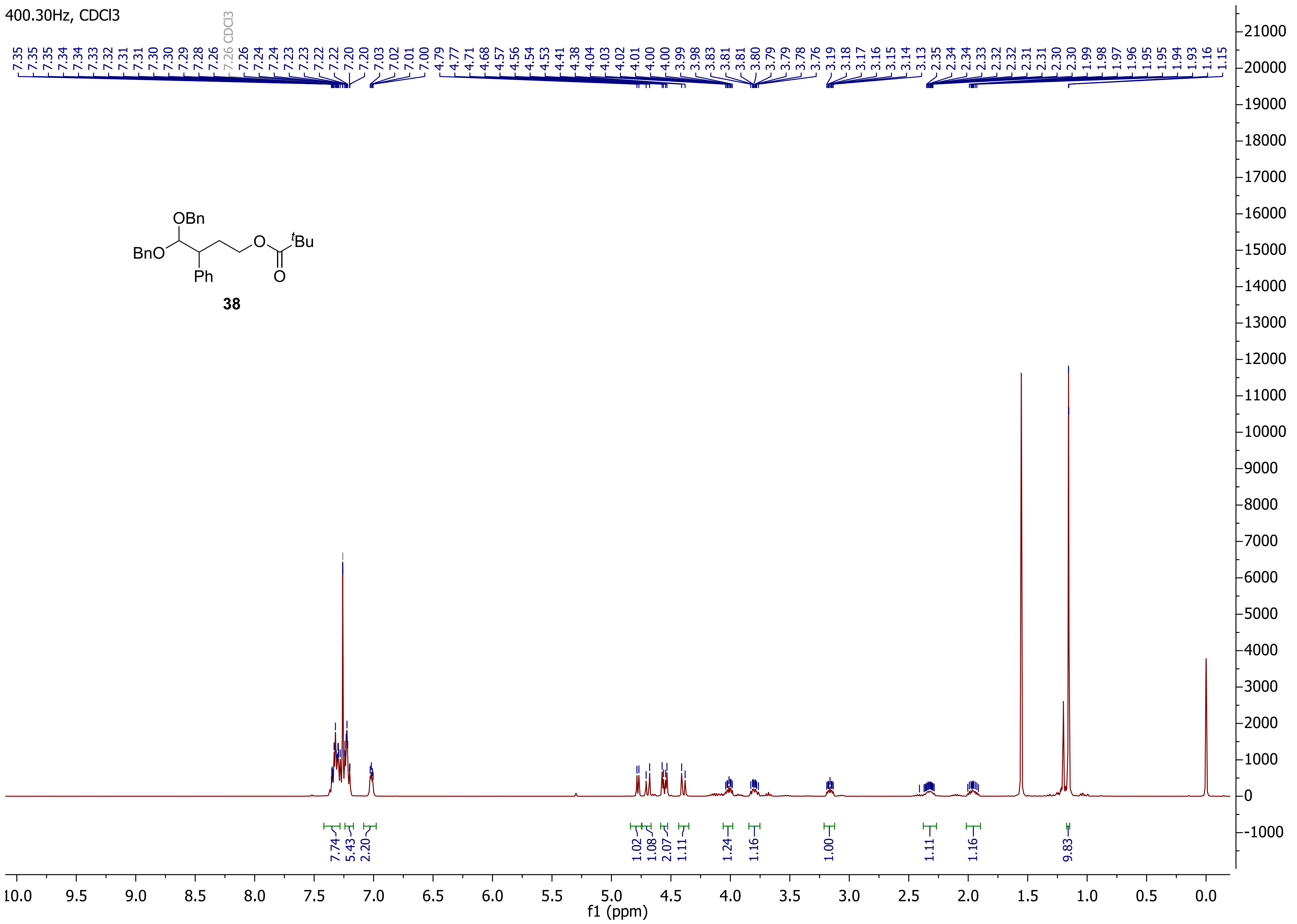
400.30Hz, CDCl3
OBn
BnO
Ph
O
tBu
O
38
7.35 7.35 7.35 7.34 7.34 7.33 7.32 7.31 7.31 7.30 7.30 7.29 7.28 7.26 7.26 CDCl3 7.26 7.24 7.24 7.23 7.23 7.22 7.22 7.20 7.20 7.03 7.02 7.01 7.00 4.79 4.77 4.71 4.68 4.57 4.56 4.54 4.53 4.41 4.38 4.04 4.03 4.02 4.01 4.00 3.99 3.98 3.83 3.81 3.81 3.80 3.79 3.79 3.78 3.76 3.19 3.18 3.17 3.16 3.15 3.14 3.13 2.35 2.34 2.34 2.33 2.32 2.32 2.31 2.31 2.30 2.30 1.99 1.98 1.97 1.96 1.95 1.95 1.94 1.93 1.16 1.15
7.74 5.43 2.20 1.02 1.08 2.07 1.11 1.24 1.16 1.00 1.11 1.16 9.83
f1 (ppm)

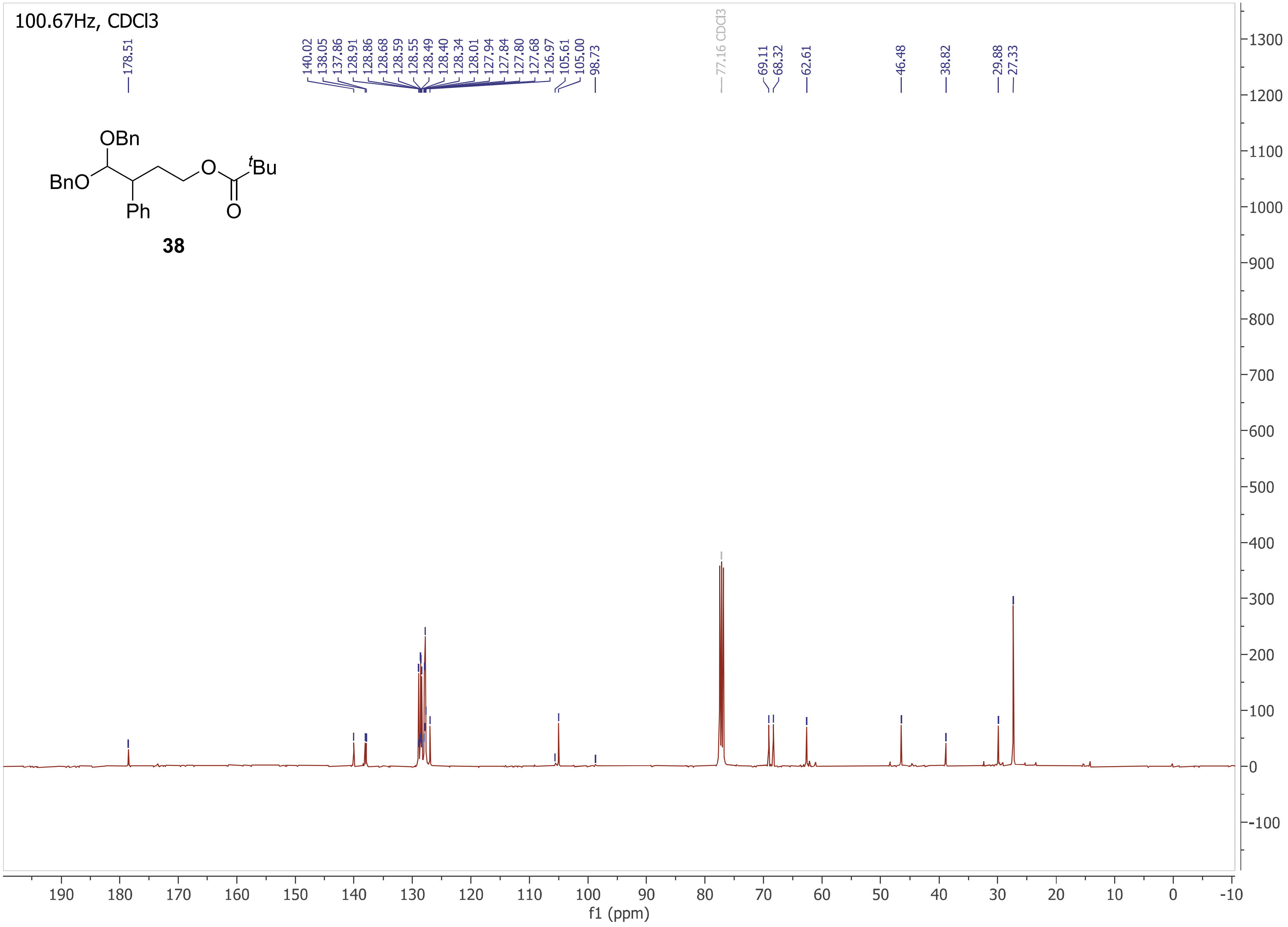
100.67Hz, CDCl3
178.51
140.02
138.05
137.86
128.91
128.86
128.68
128.59
128.55
128.49
128.40
128.34
128.01
127.94
127.84
127.80
127.68
126.97
105.61
105.00
98.73
77.16 CDCl3
69.11
68.32
62.61
46.48
38.82
29.88
27.33
OBn
BnO
O
^{t}Bu
Ph
O
38
f1 (ppm)

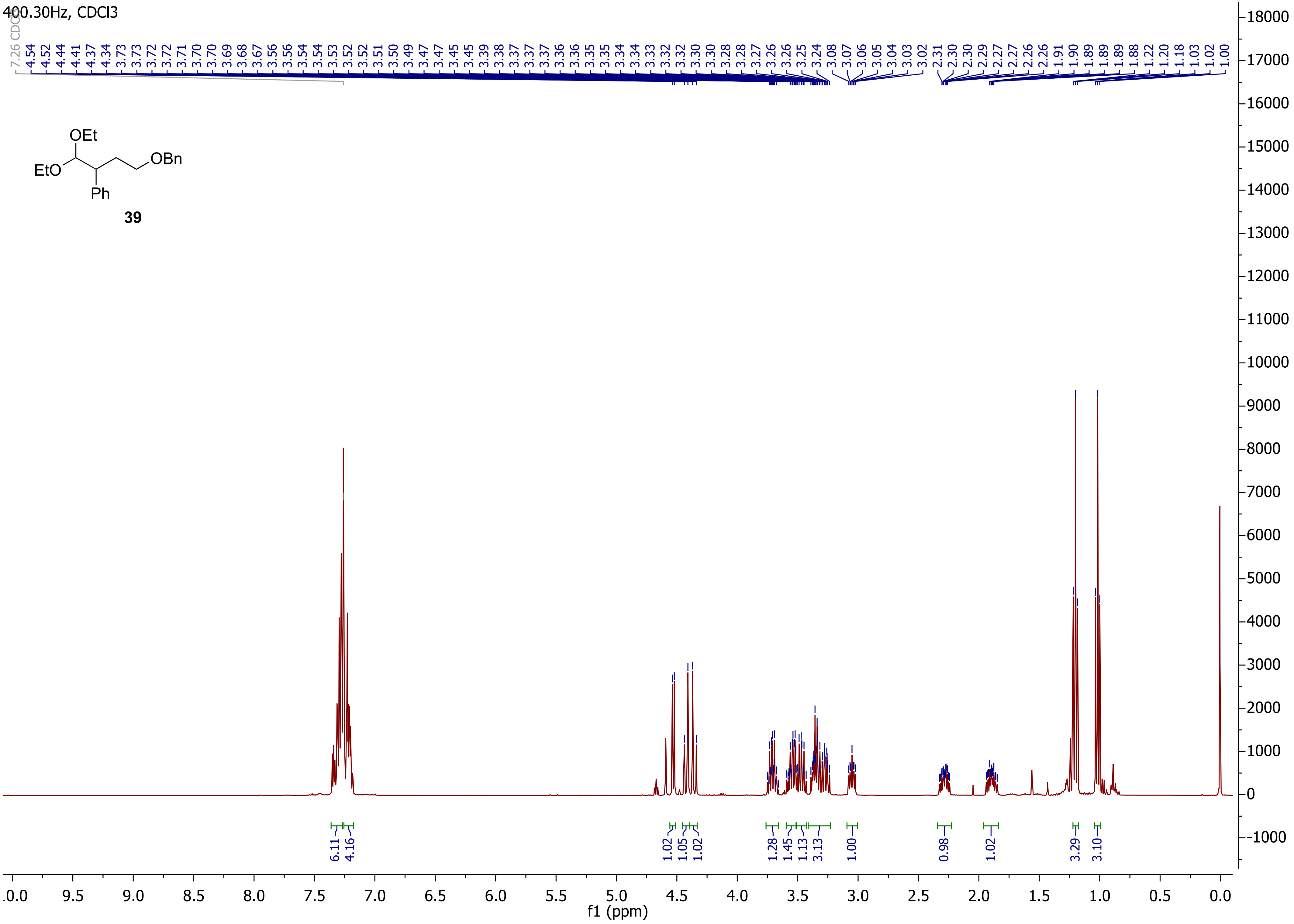
400.30Hz, CDCl3
7.26 CDCl3
4.54 4.52 4.44 4.41 4.37 4.34 3.73 3.73 3.72 3.72 3.71 3.70 3.70 3.69 3.68 3.67 3.56 3.56 3.54 3.54 3.53 3.52 3.52 3.51 3.50 3.49 3.47 3.47 3.45 3.45 3.39 3.38 3.37 3.37 3.37 3.36 3.36 3.35 3.35 3.34 3.34 3.33 3.32 3.32 3.30 3.30 3.28 3.28 3.27 3.26 3.26 3.25 3.24 3.08 3.07 3.06 3.05 3.04 3.03 3.02 2.31 2.30 2.30 2.29 2.27 2.27 2.26 1.91 1.90 1.89 1.89 1.89 1.88 1.22 1.20 1.18 1.03 1.02 1.00
OEt
EtO
OBn
Ph
39
6.11 4.16 1.02 1.05 1.02 1.28 1.45 1.13 3.13 1.00 0.98 1.02 3.29 3.10
f1 (ppm)

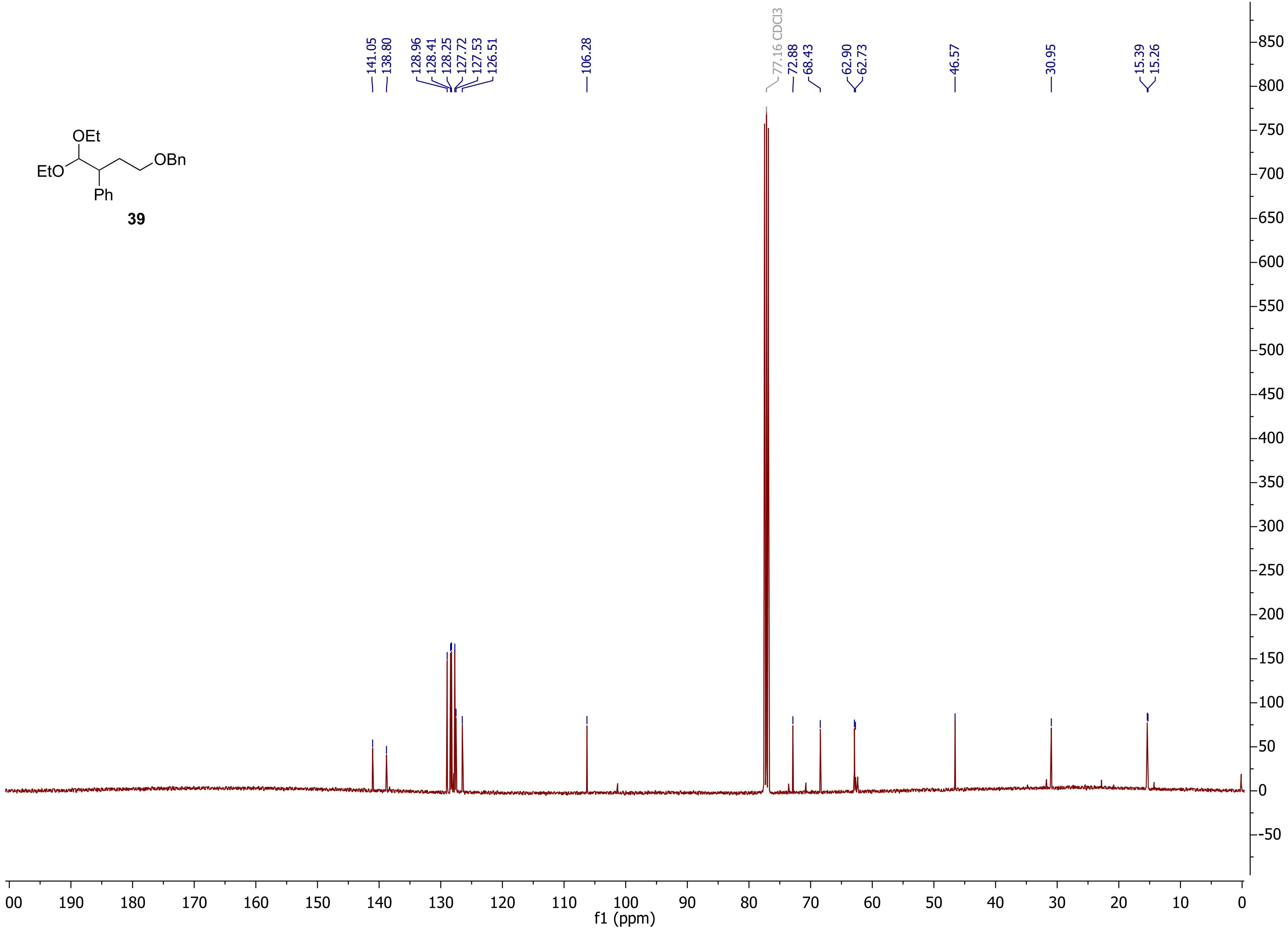

OEt
EtO
OBn
Ph
39
141.05
138.80
128.96
128.41
128.25
127.72
127.53
126.51
106.28
77.16 CDCl3
72.88
68.43
62.90
62.73
46.57
30.95
15.39
15.26
f1 (ppm)

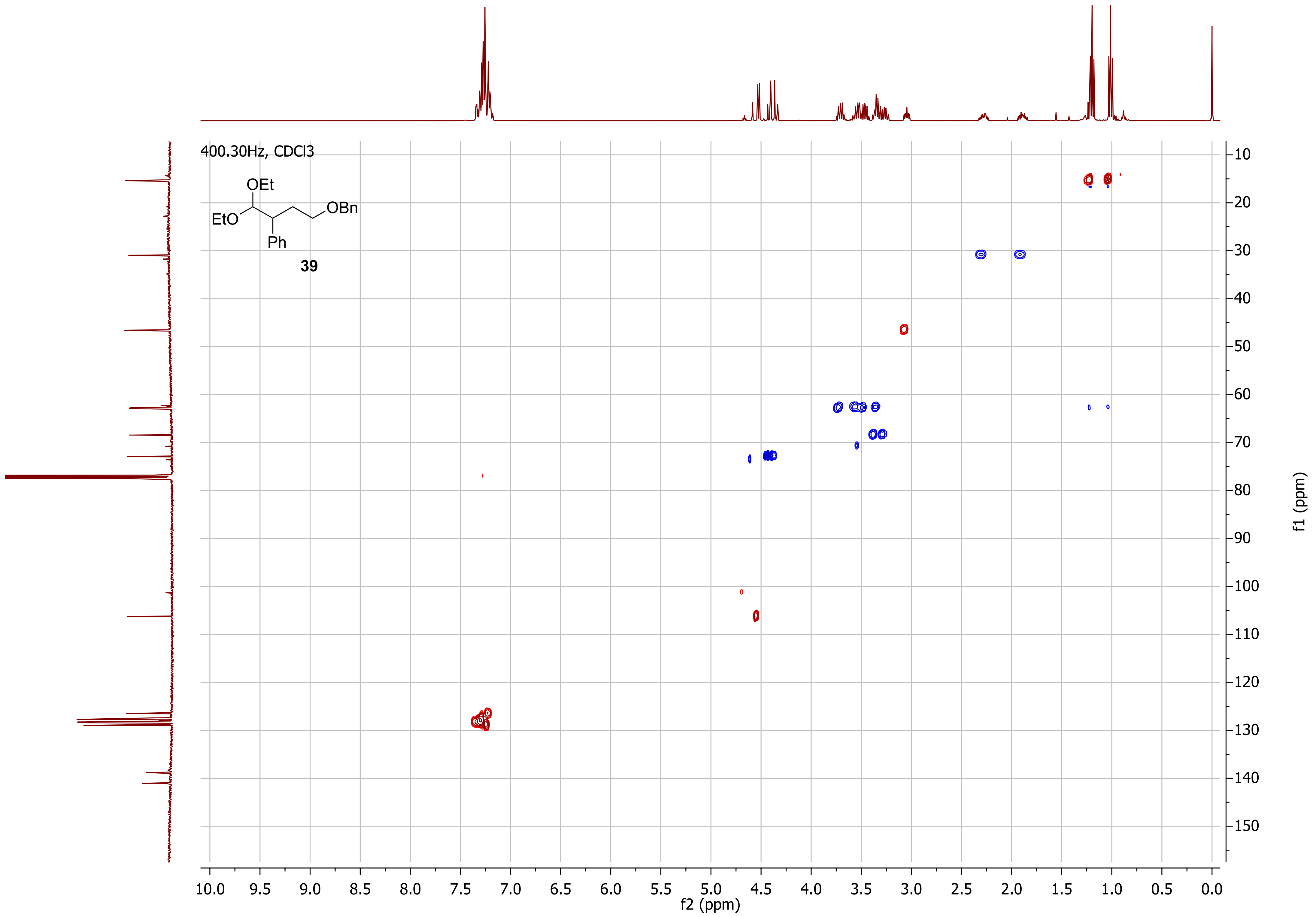
400.30Hz, CDCl3
OEt
EtO
OBn
Ph
39
f2 (ppm)
f1 (ppm)

400.30Hz, CDCl3

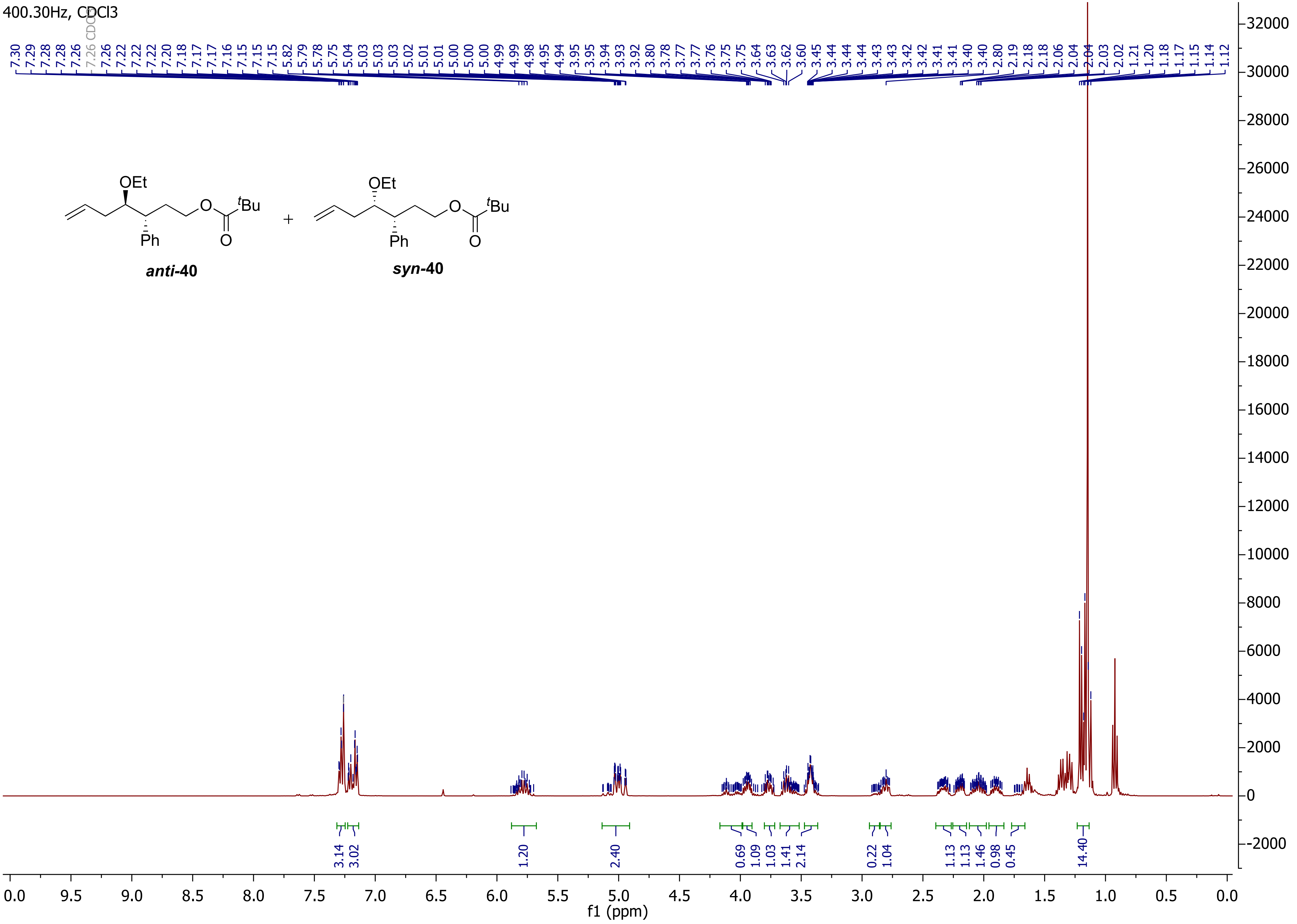

100.67Hz, CDCl3

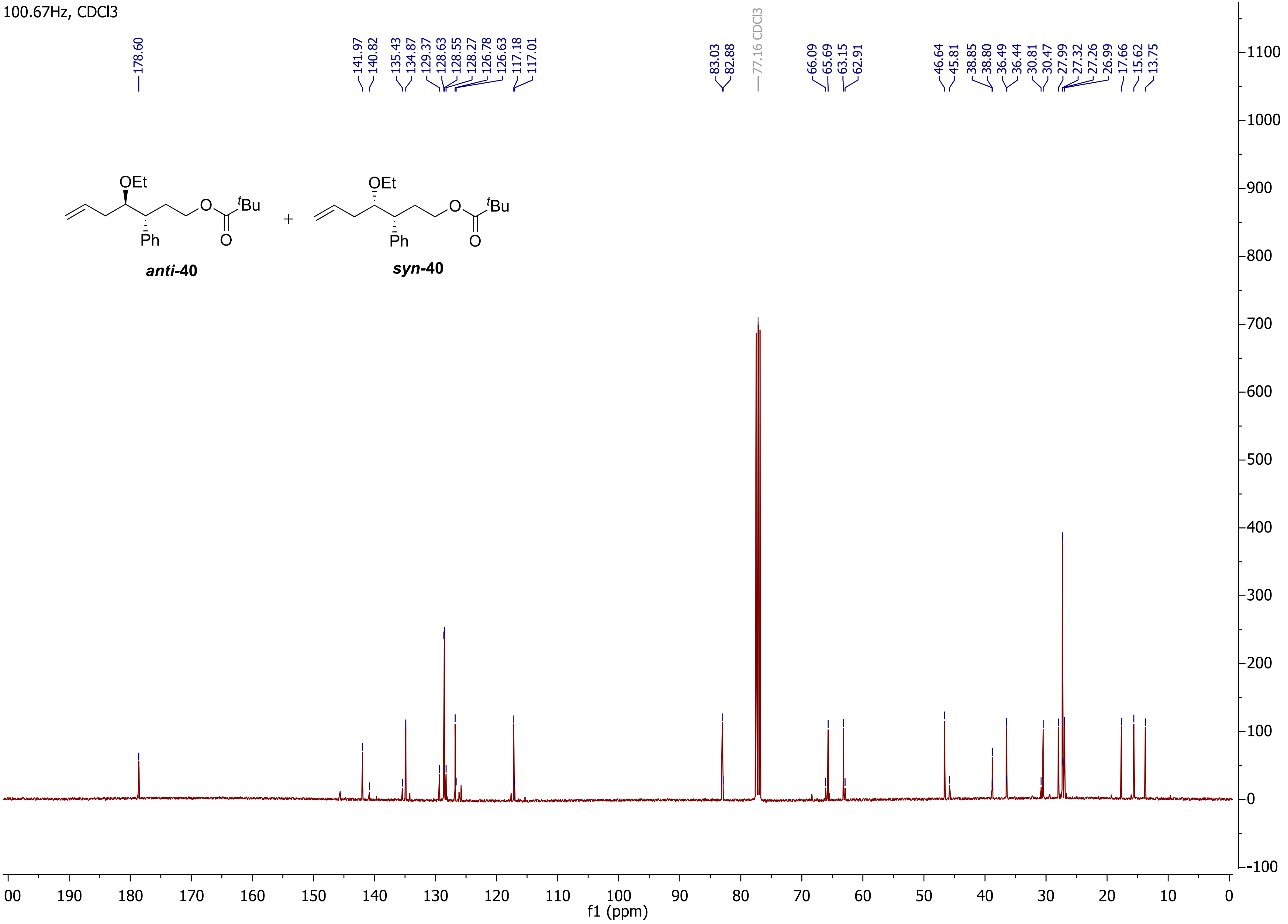

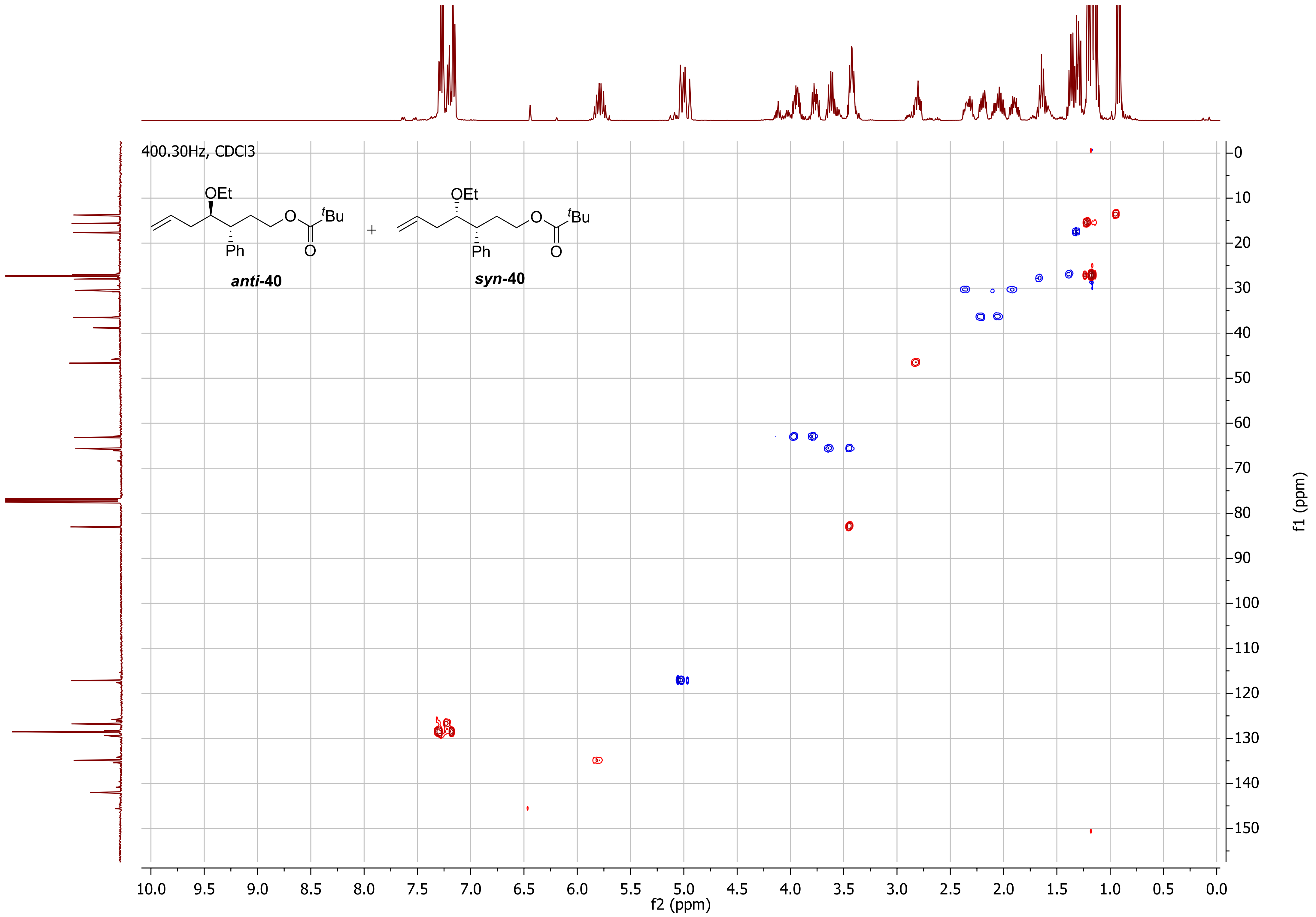

400.30Hz, CDCl3
OEt
tBu
Ph
O
anti-40
+
syn-40
f2 (ppm)
f1 (ppm)

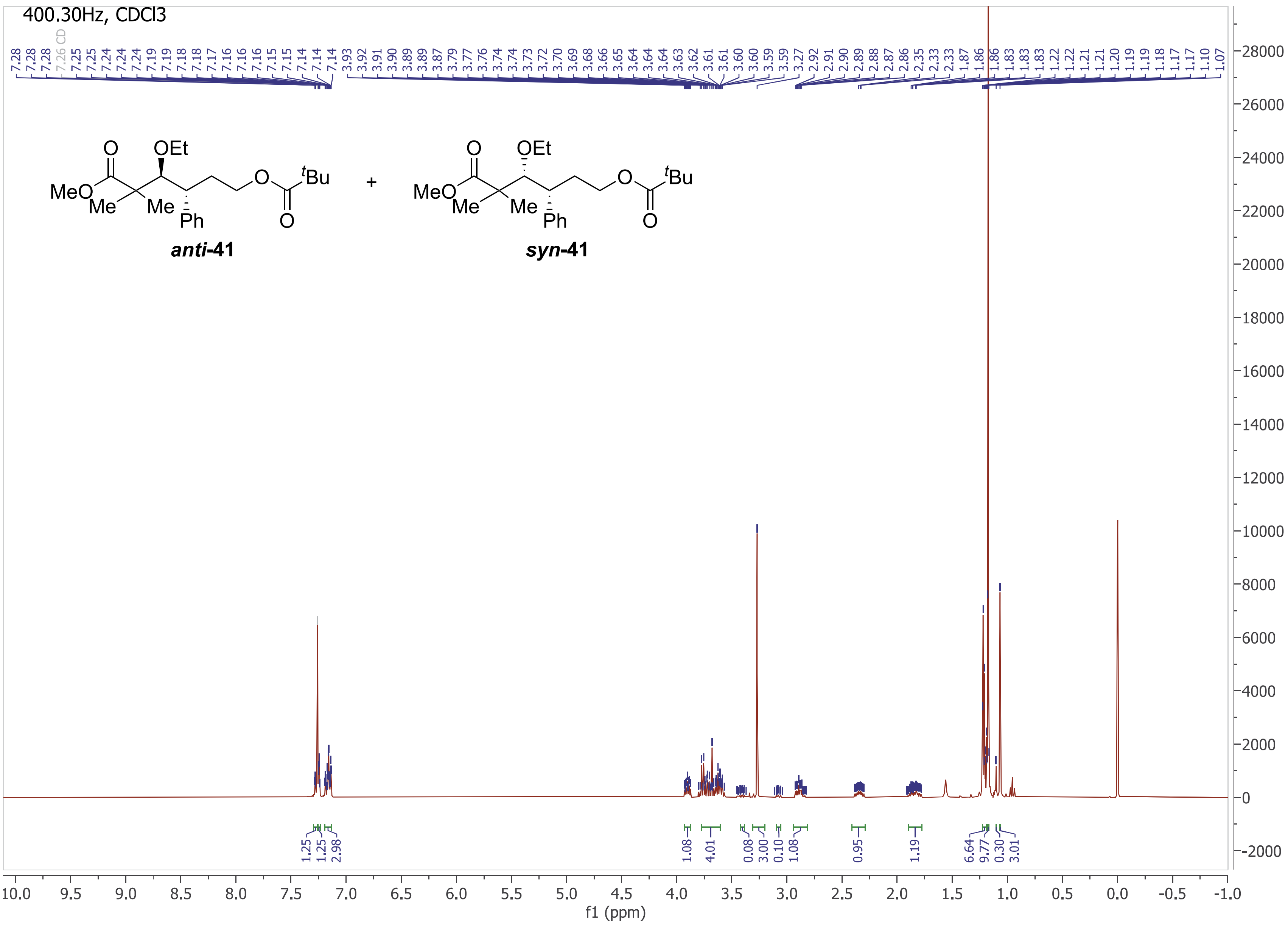
400.30Hz, CDCl3
anti-41
syn-41
f1 (ppm)

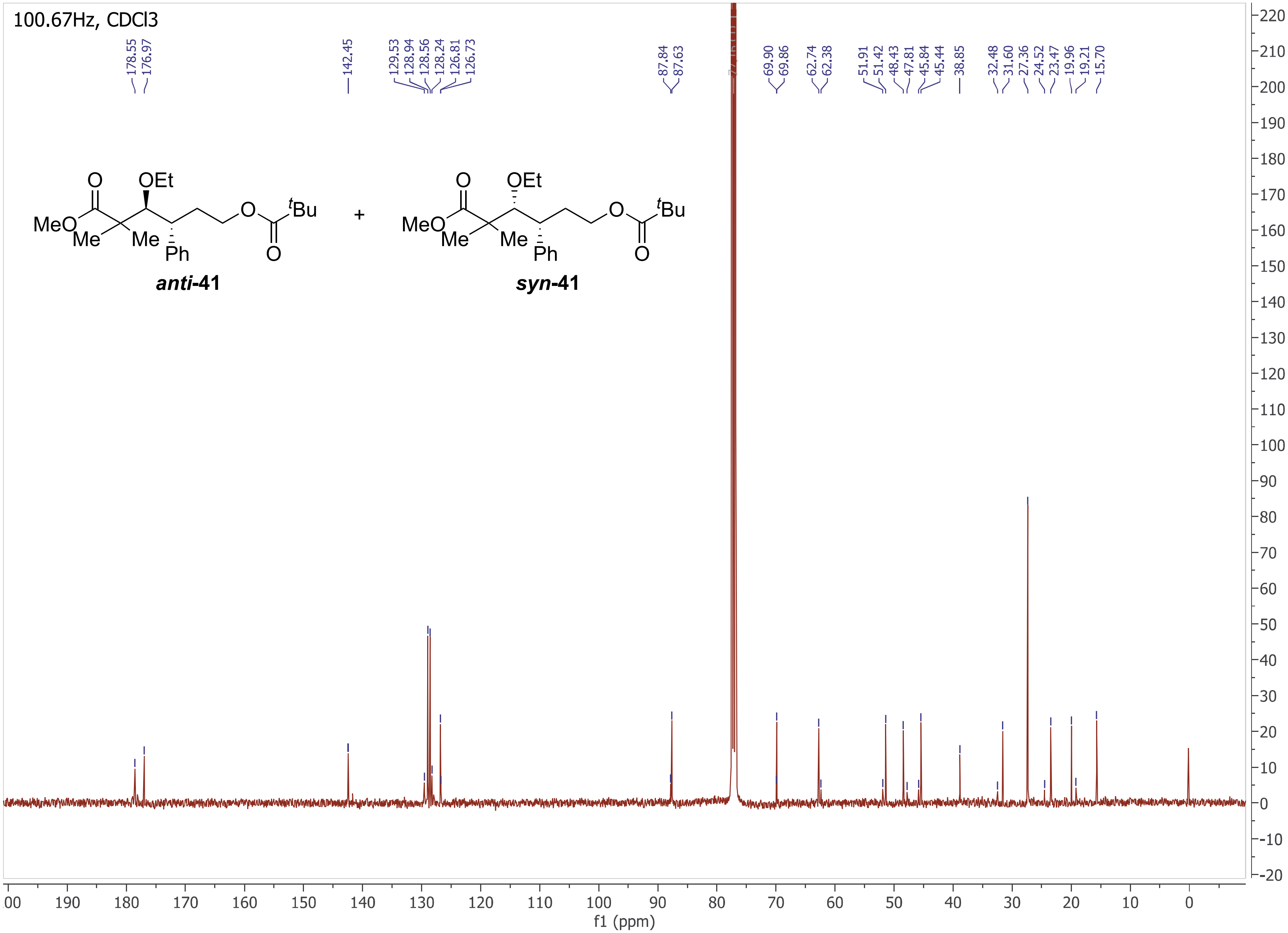

100.67Hz, CDCl3
178.55
176.97
142.45
129.53
128.94
128.56
128.24
126.81
126.73
87.84
87.63
69.90
69.86
62.74
62.38
51.91
51.42
48.43
47.81
45.84
45.44
38.85
32.48
31.60
27.36
24.52
23.47
19.96
19.21
15.70
MeO
Me
Me
OEt
O
O
tBu
Ph
anti-41
+
syn-41
f1 (ppm)

400.30Hz, CDCl3

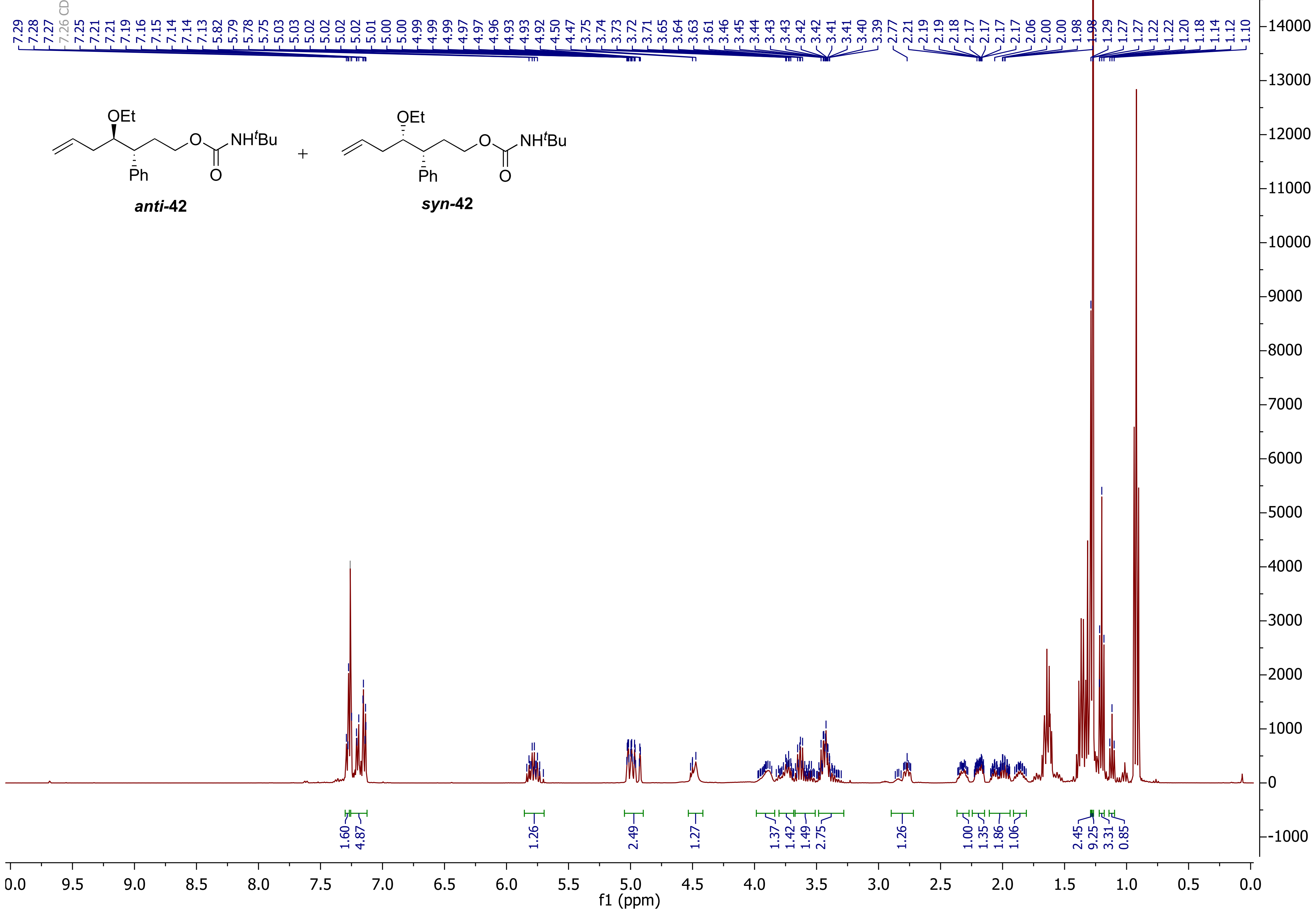

anti-**42** + *syn*-**42**

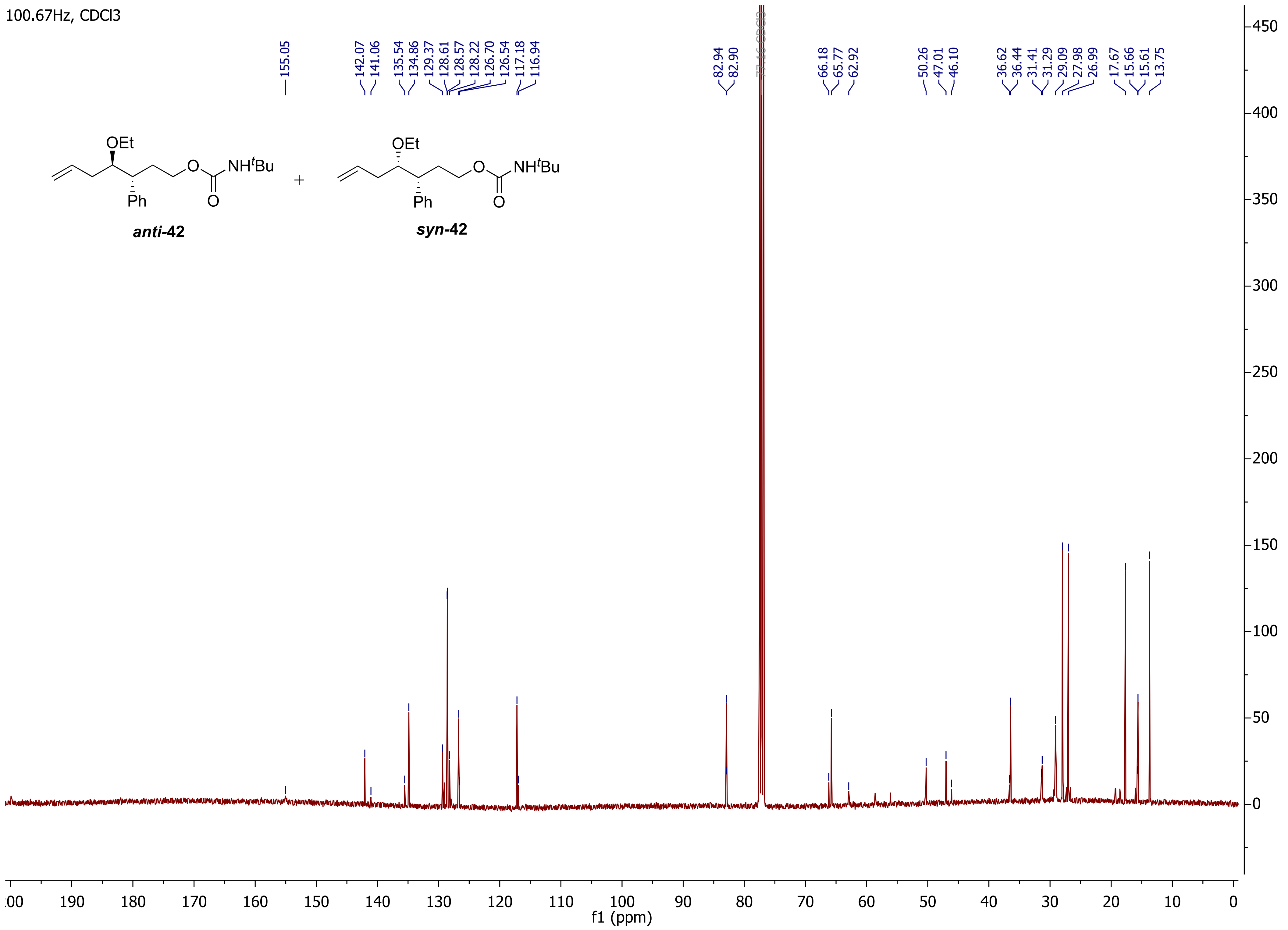

100.67Hz, CDCl3
155.05
142.07
141.06
135.54
134.86
129.37
128.61
128.57
128.22
126.70
126.54
117.18
116.94
82.94
82.90
77.16 CDCl3
66.18
65.77
62.92
50.26
47.01
46.10
36.62
36.44
31.41
31.29
29.09
27.98
26.99
17.67
15.66
15.61
13.75
OEt
NH^{t}Bu
Ph
O
anti-42
+
syn-42
f1 (ppm)

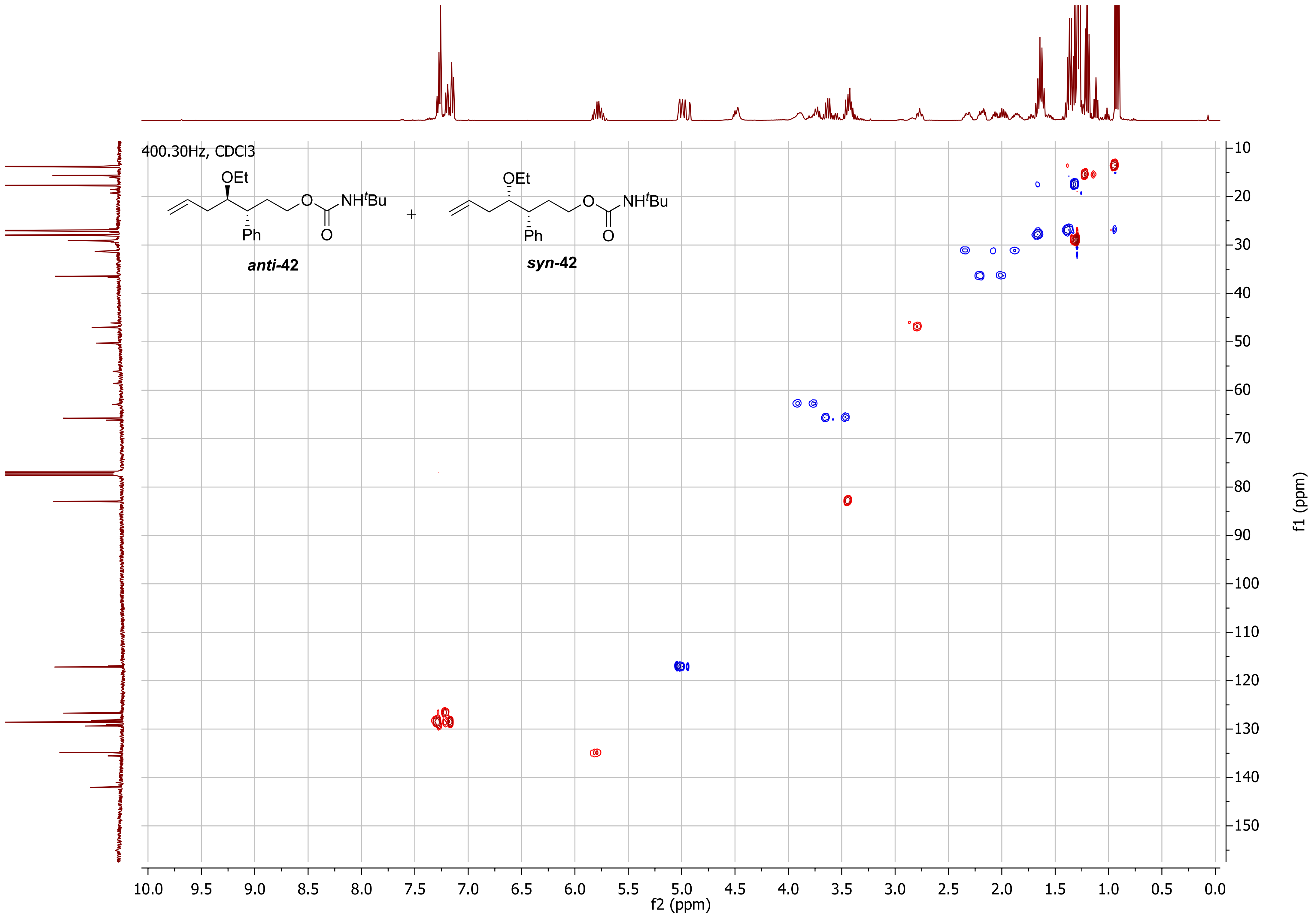

400.30Hz, CDCl3
OEt
NH^{t}Bu
Ph
O
anti-42
syn-42
f2 (ppm)
f1 (ppm)

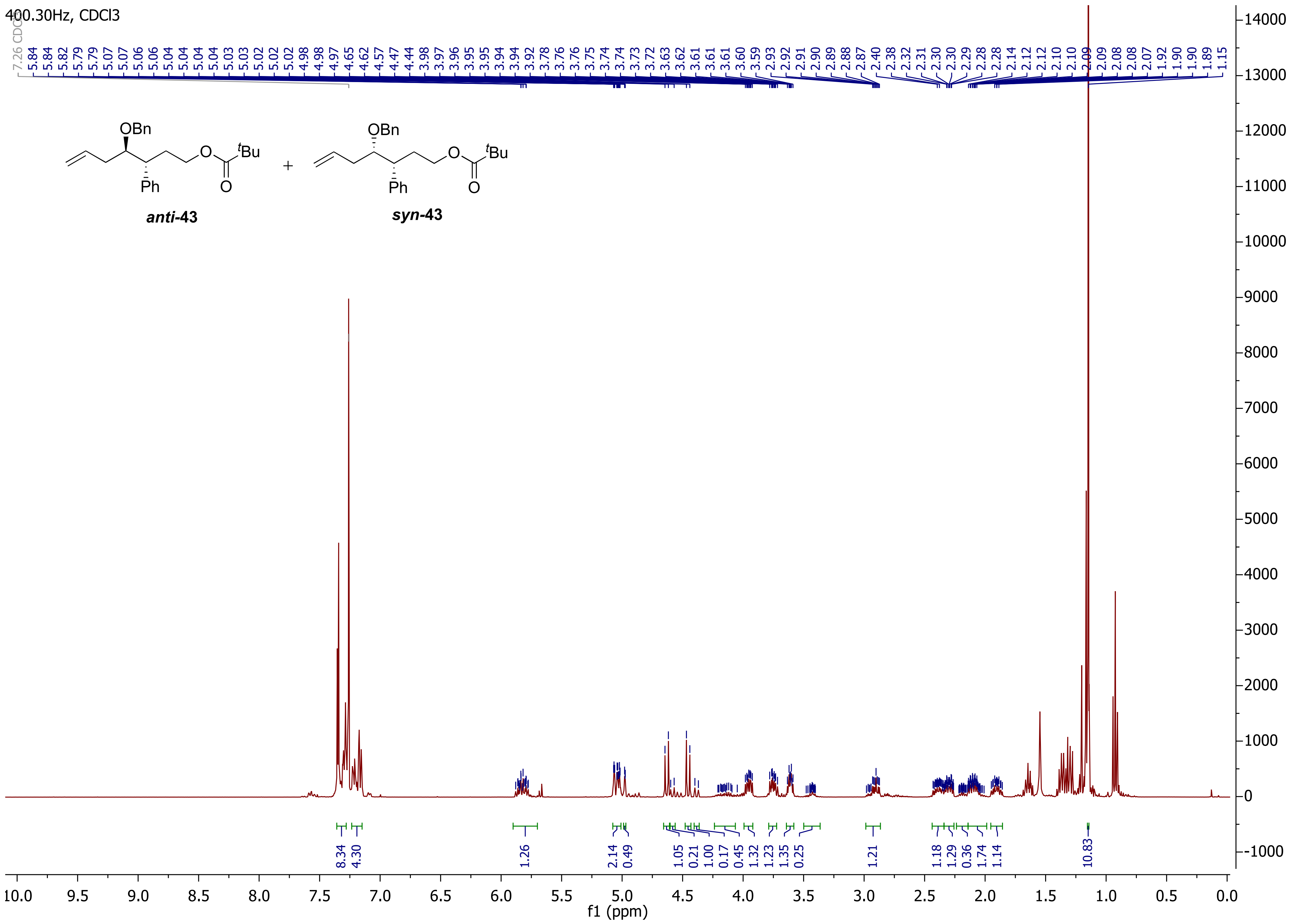
400.30Hz, CDCl3
anti-43
syn-43
f1 (ppm)

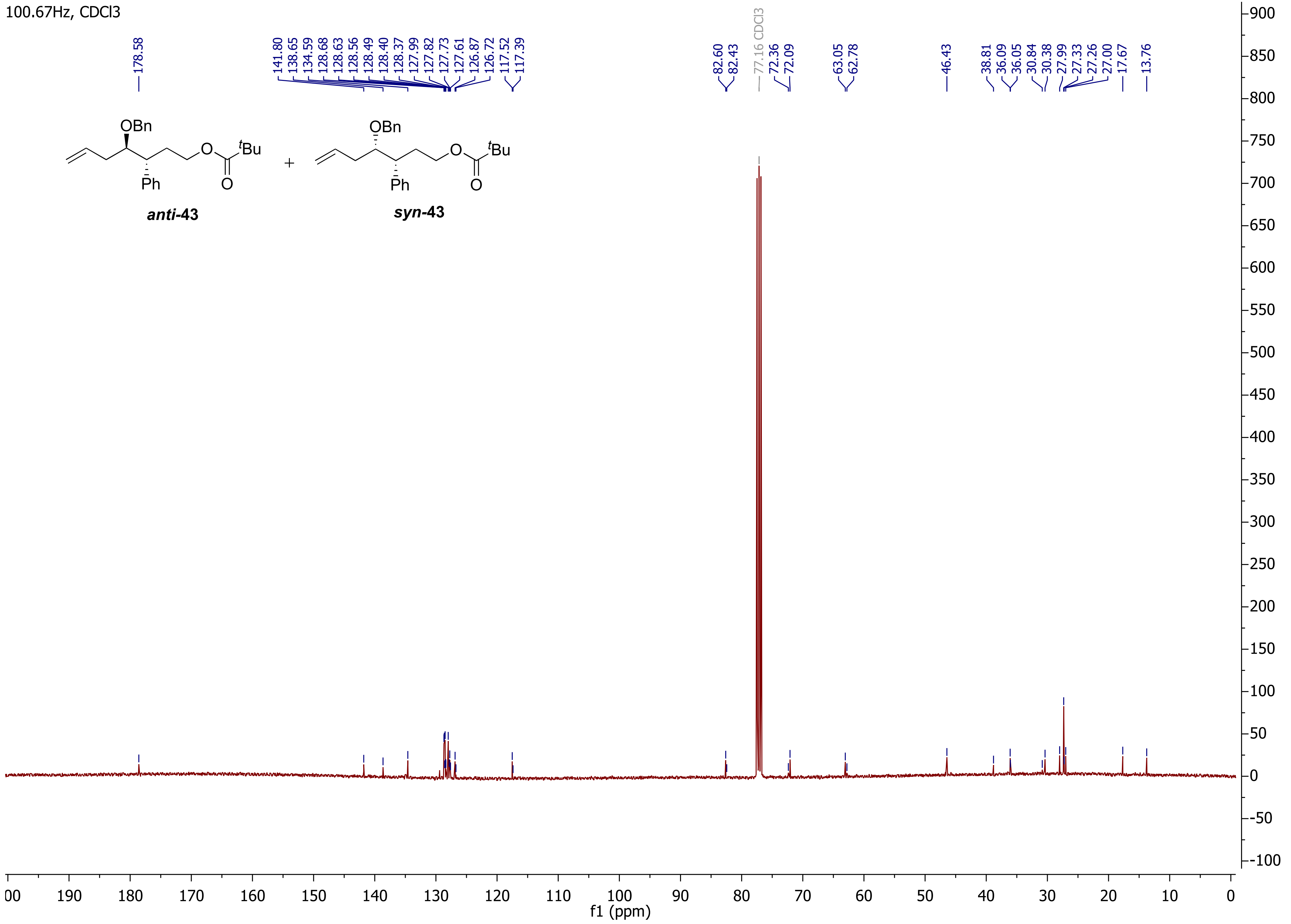
100.67Hz, CDCl3
178.58
141.80
138.65
134.59
128.68
128.63
128.56
128.49
128.40
128.37
127.99
127.82
127.73
127.61
126.87
126.72
117.52
117.39
82.60
82.43
77.16 CDCl3
72.36
72.09
63.05
62.78
46.43
38.81
36.09
36.05
30.84
30.38
27.99
27.33
27.26
27.00
17.67
13.76
OBn
tBu
Ph
O
+
anti-43
syn-43
f1 (ppm)

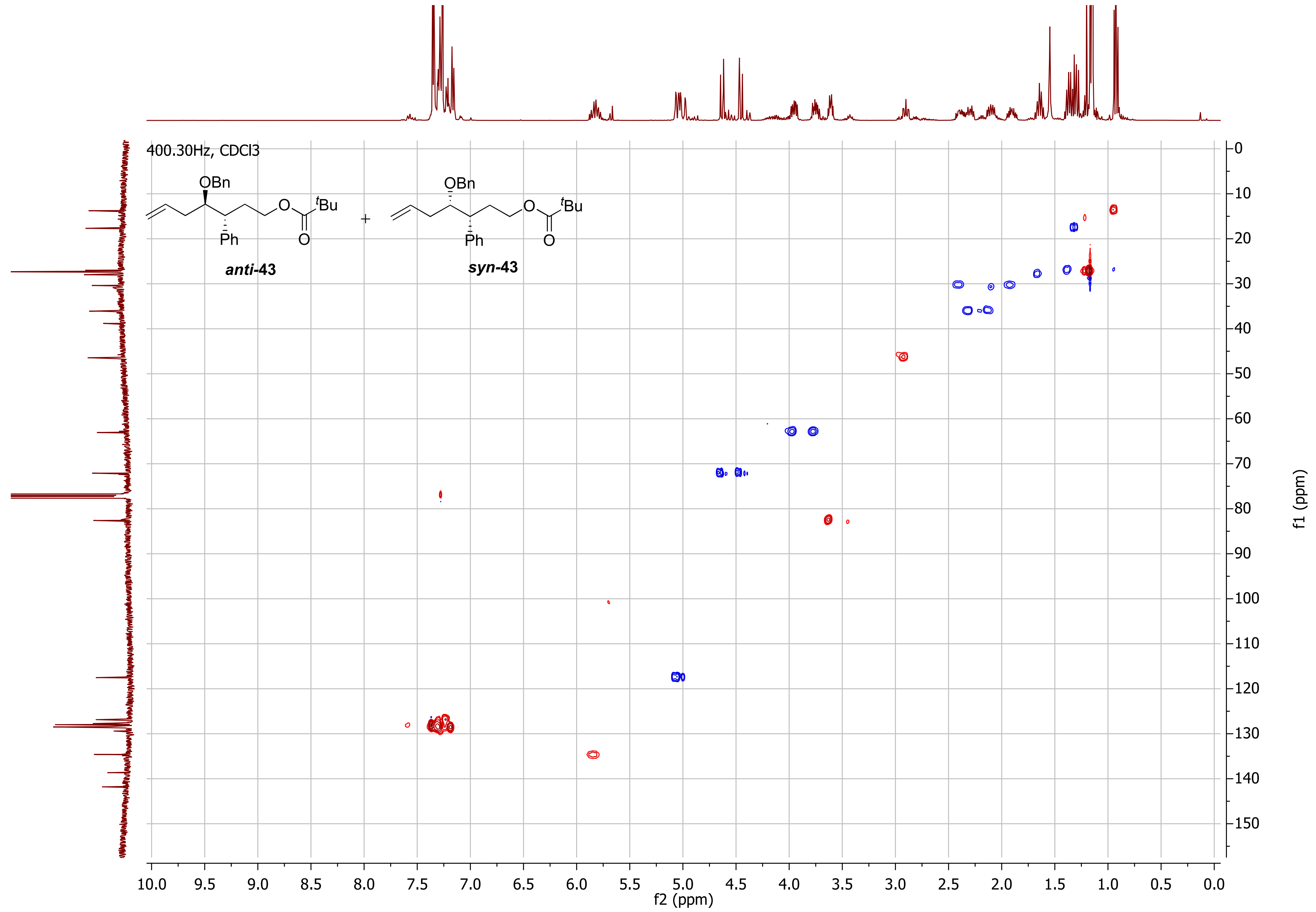
400.30Hz, CDCl3
OBn
tBu
Ph
O
anti-43
+
syn-43
f2 (ppm)
f1 (ppm)

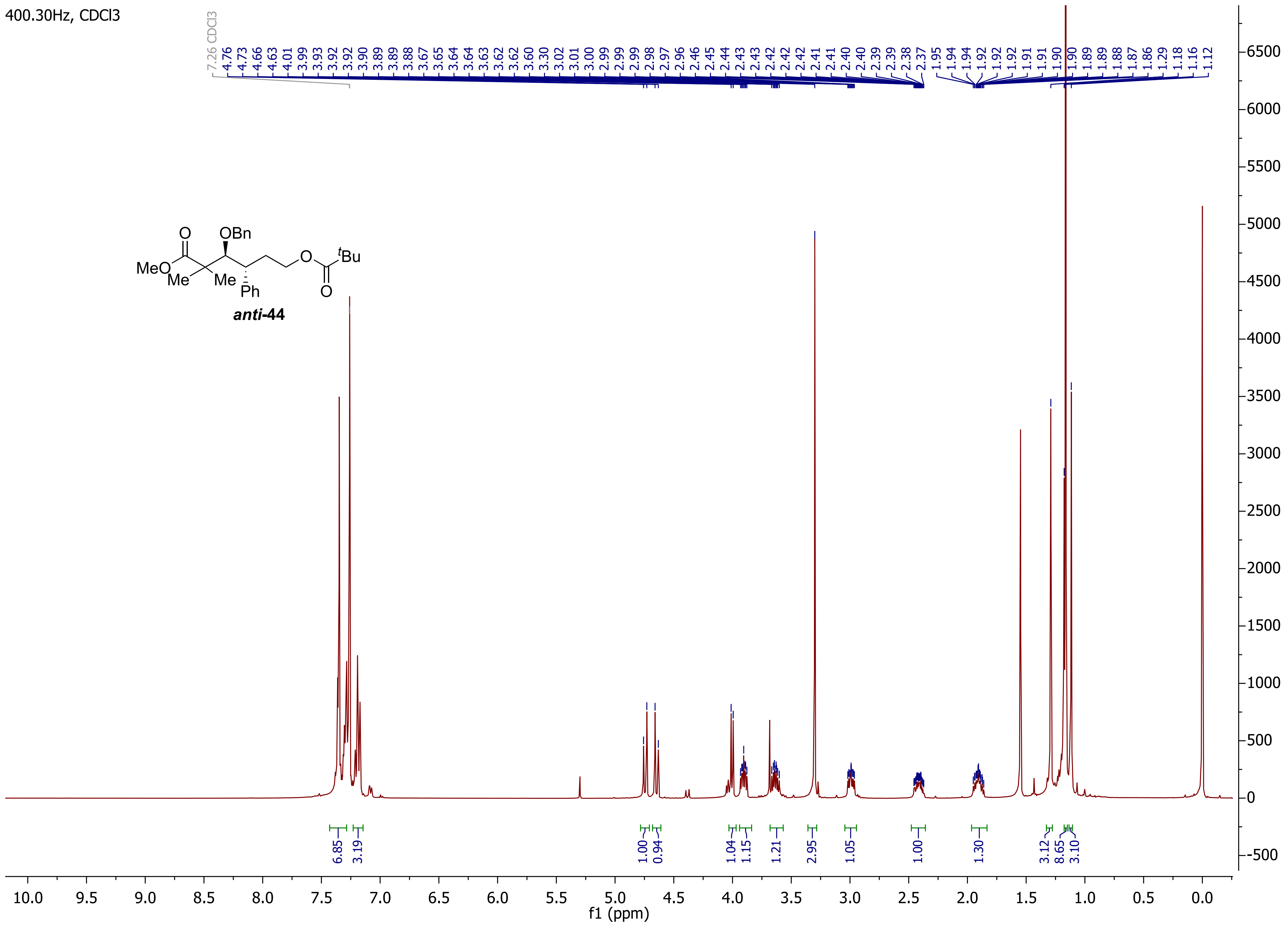
400.30Hz, CDCl3
7.26 CDCl3
4.76 4.73 4.66 4.63 4.01 3.99 3.93 3.92 3.92 3.90 3.89 3.89 3.88 3.67 3.65 3.64 3.63 3.62 3.62 3.60 3.30 3.02 3.01 3.00 2.99 2.99 2.99 2.98 2.97 2.96 2.46 2.45 2.44 2.43 2.43 2.42 2.42 2.42 2.41 2.41 2.40 2.40 2.39 2.39 2.38 2.37 1.95 1.94 1.94 1.92 1.92 1.92 1.91 1.91 1.90 1.90 1.89 1.89 1.88 1.87 1.86 1.29 1.18 1.16 1.12
MeO
Me
Me
OBn
Ph
O
O
^{t}Bu
O
anti-**44**
6.85 3.19 1.00 0.94 1.04 1.15 1.21 2.95 1.05 1.00 1.30 3.12 8.65 3.10
f1 (ppm)

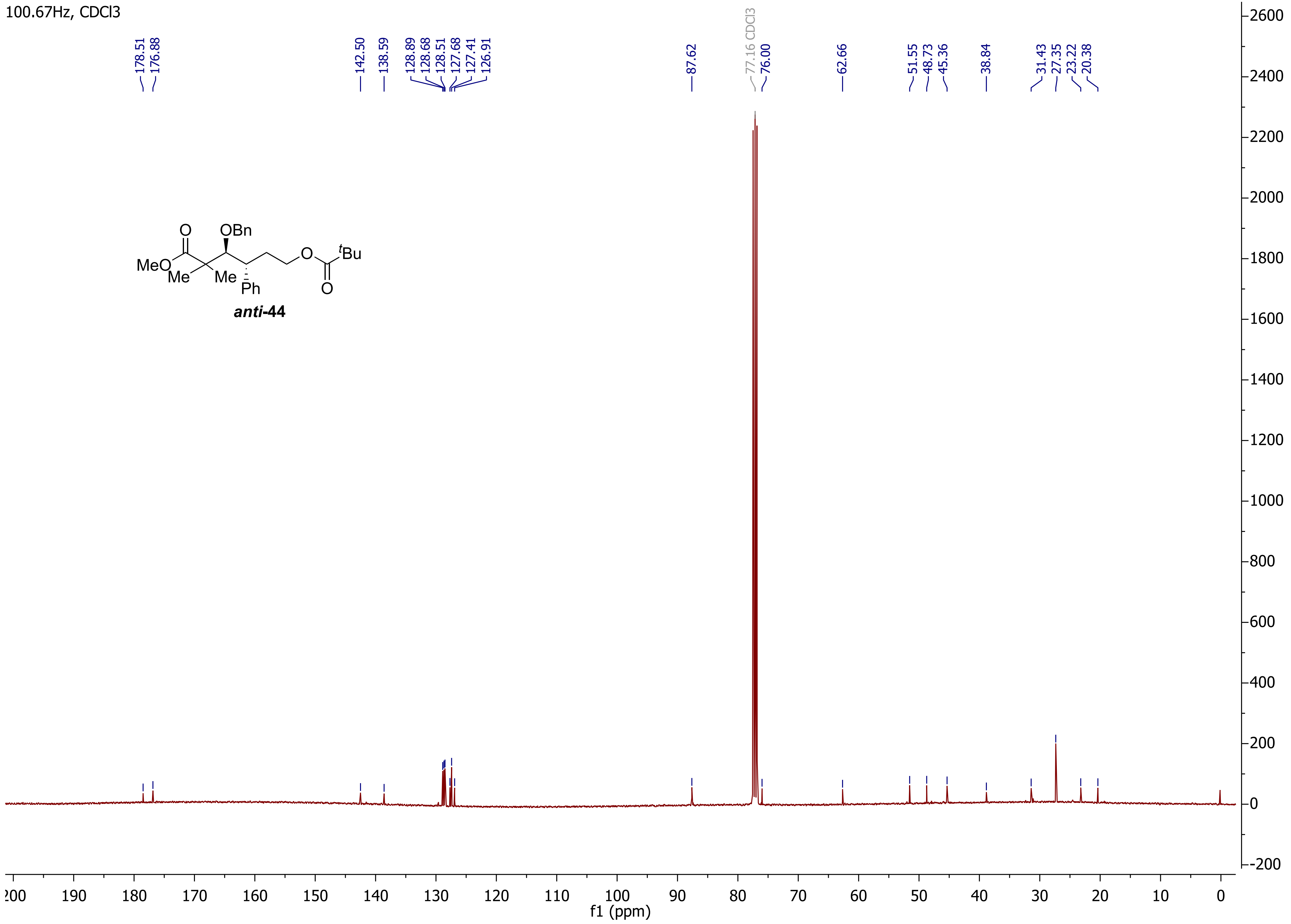
100.67Hz, CDCl3
178.51
176.88
142.50
138.59
128.89
128.68
128.51
127.68
127.41
126.91
87.62
77.16 CDCl3
76.00
62.66
51.55
48.73
45.36
38.84
31.43
27.35
23.22
20.38
O
OBn
MeO
Me
Me
Ph
O
tBu
O
anti-44
f1 (ppm)

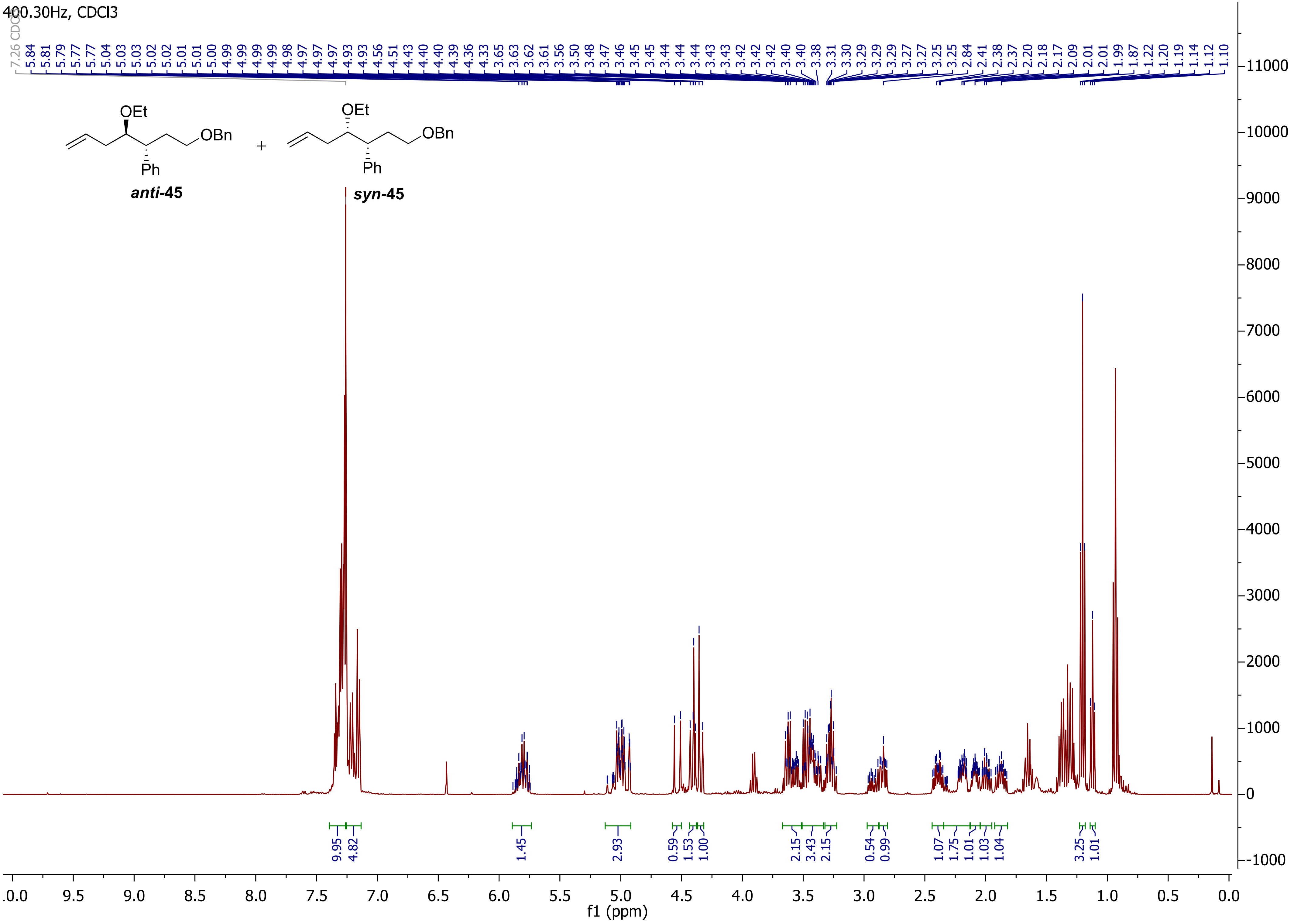

400.30Hz, CDCl3
7.26 CDCl3
5.84 5.81 5.79 5.77 5.77 5.04 5.03 5.03 5.02 5.02 5.01 5.01 5.00 4.99 4.99 4.99 4.99 4.98 4.97 4.97 4.97 4.93 4.93 4.56 4.51 4.43 4.40 4.40 4.39 4.36 4.33 3.65 3.63 3.62 3.61 3.56 3.50 3.48 3.47 3.46 3.45 3.45 3.44 3.44 3.44 3.43 3.43 3.42 3.42 3.42 3.40 3.40 3.38 3.31 3.30 3.29 3.29 3.29 3.27 3.27 3.25 3.25 2.84 2.41 2.38 2.37 2.20 2.18 2.17 2.09 2.01 2.01 1.99 1.87 1.22 1.20 1.19 1.14 1.12 1.10
OEt
OBn
Ph
anti-45
+
syn-45
9.95 4.82 1.45 2.93 0.59 1.53 1.00 2.15 3.43 2.15 0.54 0.99 1.07 1.75 1.01 1.03 1.04 3.25 1.01
f1 (ppm)

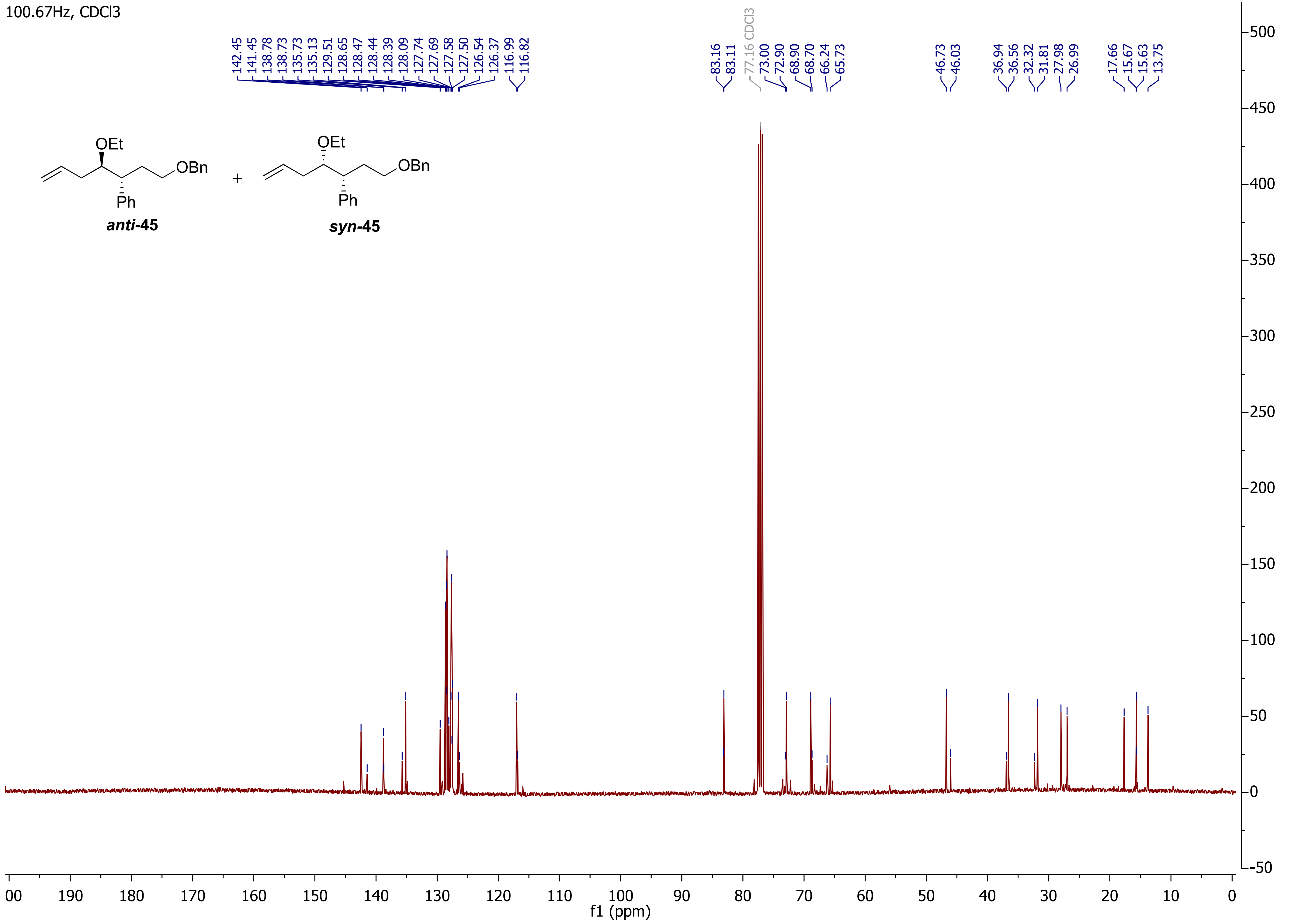

100.67Hz, CDCl3
142.45
141.45
138.78
138.73
135.73
135.13
129.51
128.65
128.47
128.44
128.39
128.09
127.74
127.69
127.58
127.50
126.54
126.37
116.99
116.82
83.16
83.11
77.16 CDCl3
73.00
72.90
68.90
68.70
66.24
65.73
46.73
46.03
36.94
36.56
32.32
31.81
27.98
26.99
17.66
15.67
15.63
13.75
OEt
OBn
Ph
+
anti-45
syn-45
f1 (ppm)

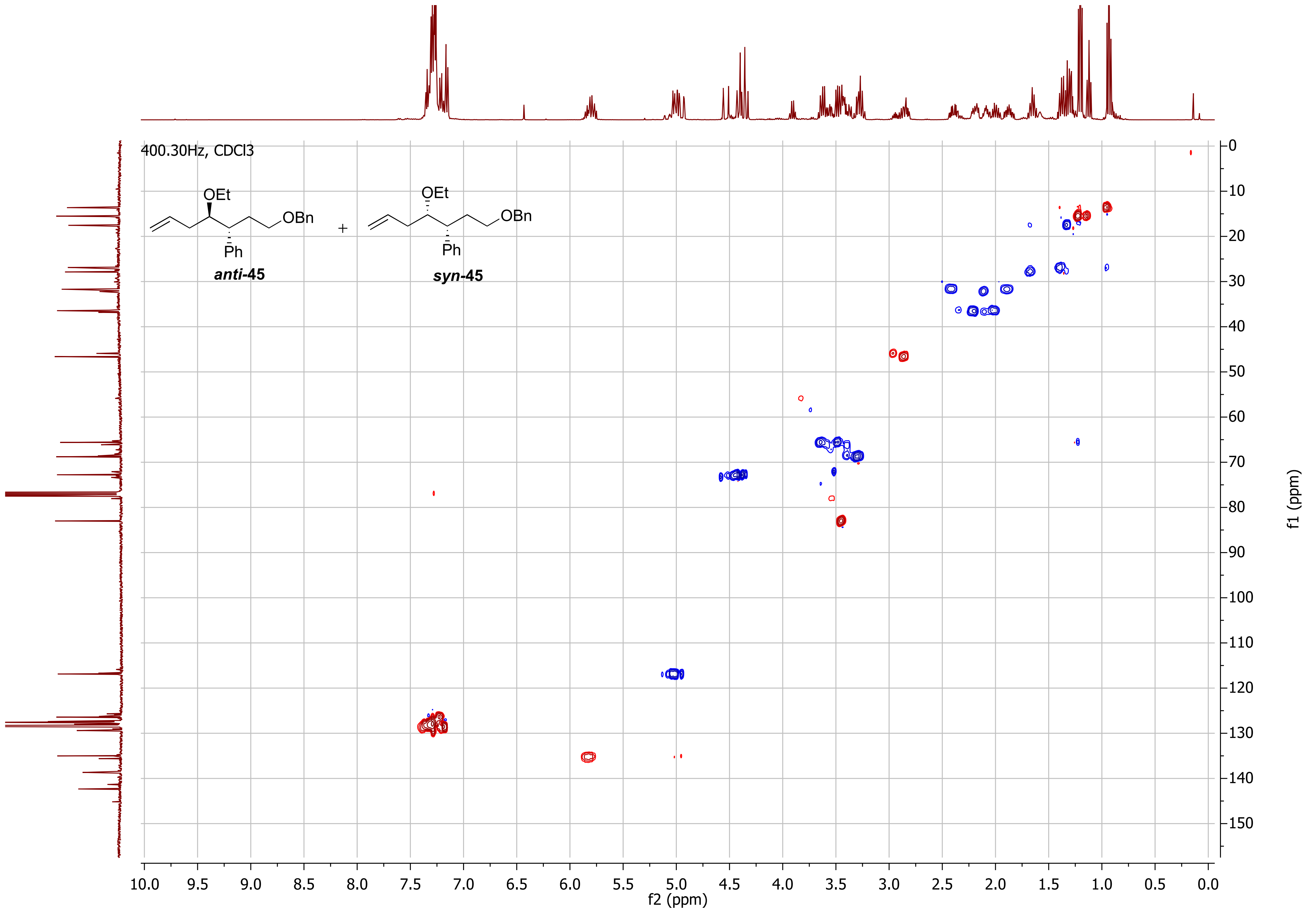
400.30Hz, CDCl3
OEt
OBn
Ph
anti-45
+
OEt
OBn
Ph
syn-45
f2 (ppm)
f1 (ppm)

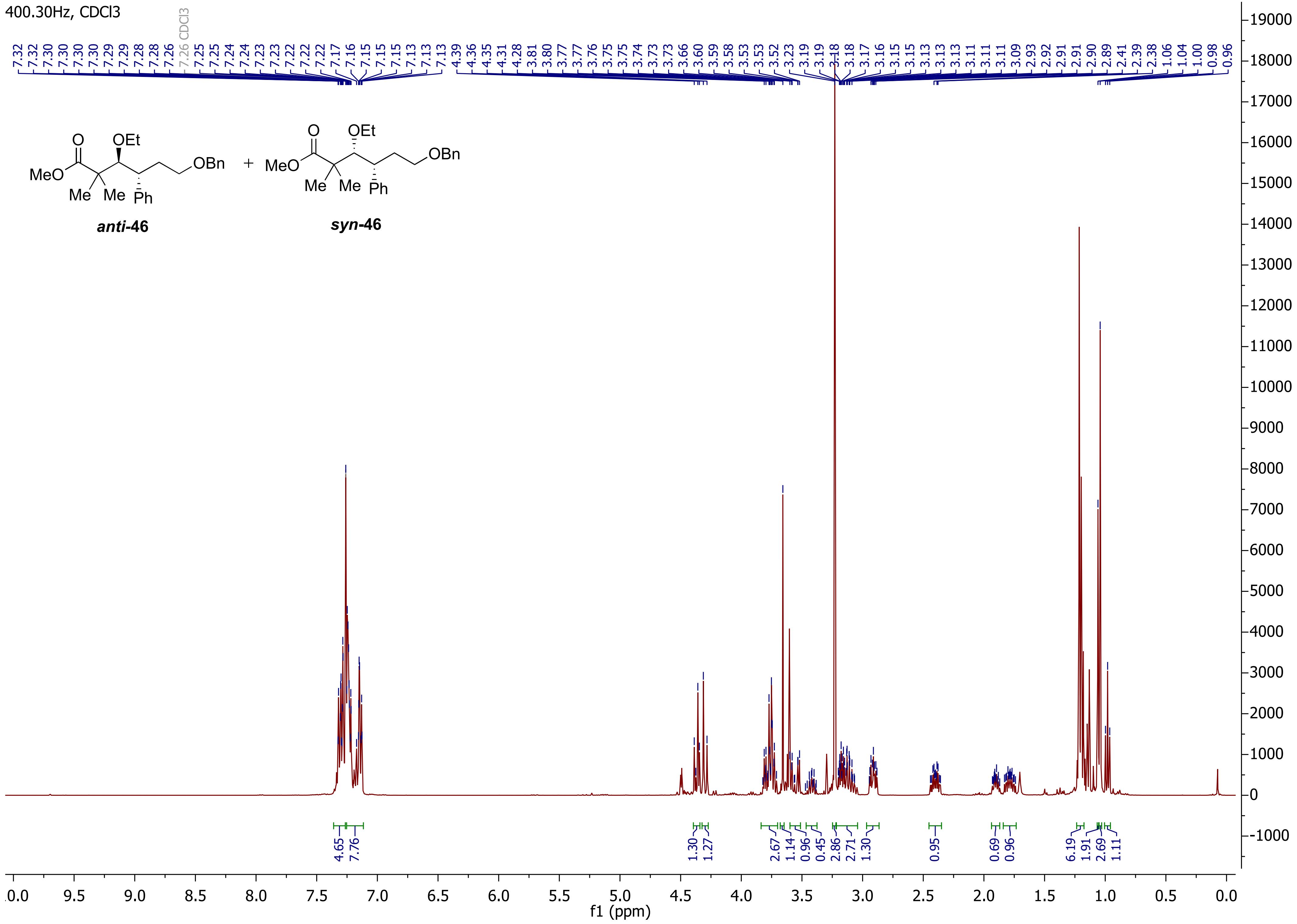

400.30Hz, CDCl3
anti-46
syn-46
MeO
OEt
OBn
Me
Ph
f1 (ppm)

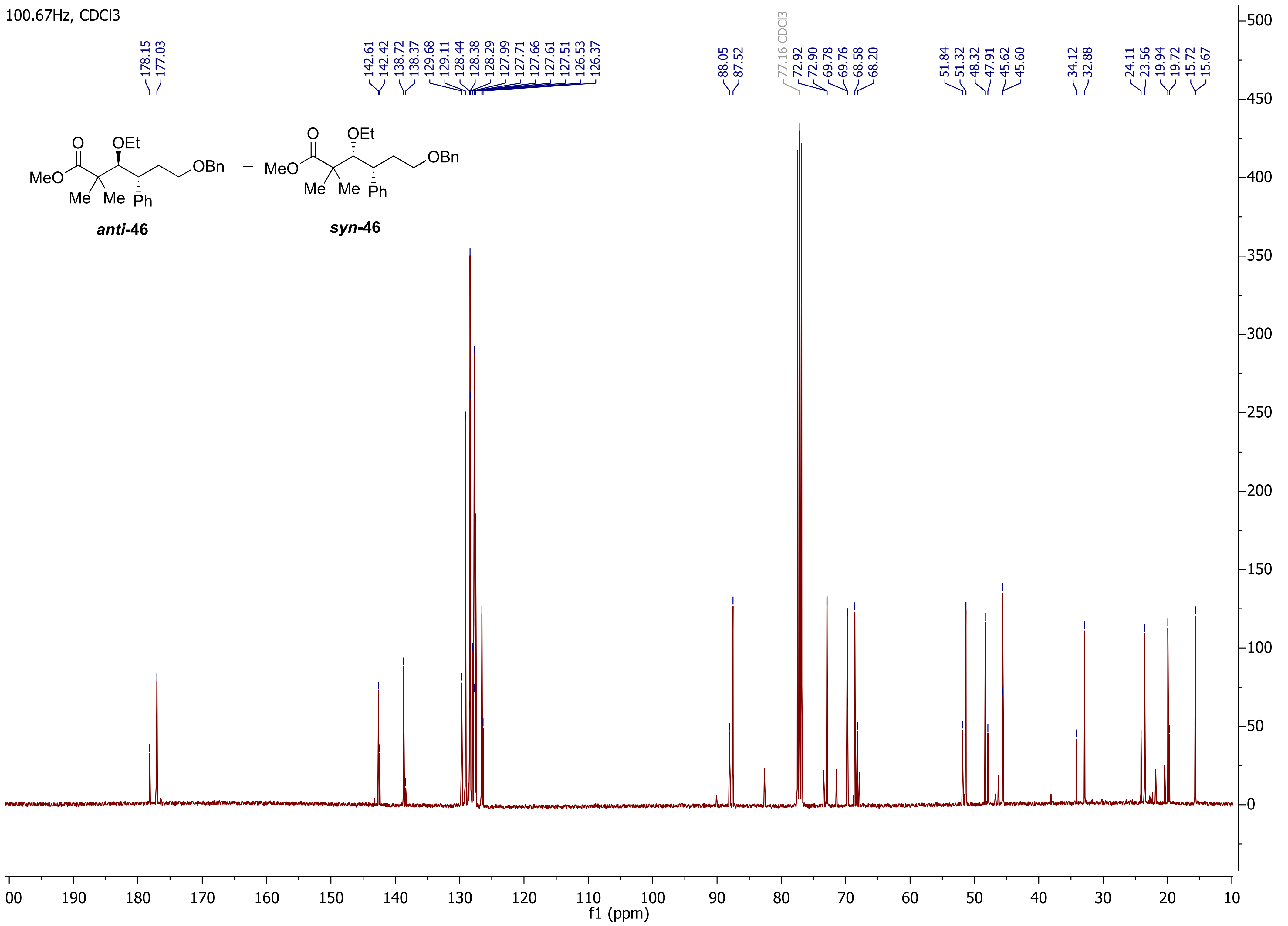

100.67Hz, CDCl3
178.15
177.03
142.61
142.42
138.72
138.37
129.68
129.11
128.44
128.38
128.29
127.99
127.71
127.66
127.61
127.51
126.53
126.37
88.05
87.52
77.16 CDCl3
72.92
72.90
69.78
69.76
68.58
68.20
51.84
51.32
48.32
47.91
45.62
45.60
34.12
32.88
24.11
23.56
19.94
19.72
15.72
15.67
MeO
O
OEt
OBn
Me
Me
Ph
anti-46
syn-46
f1 (ppm)

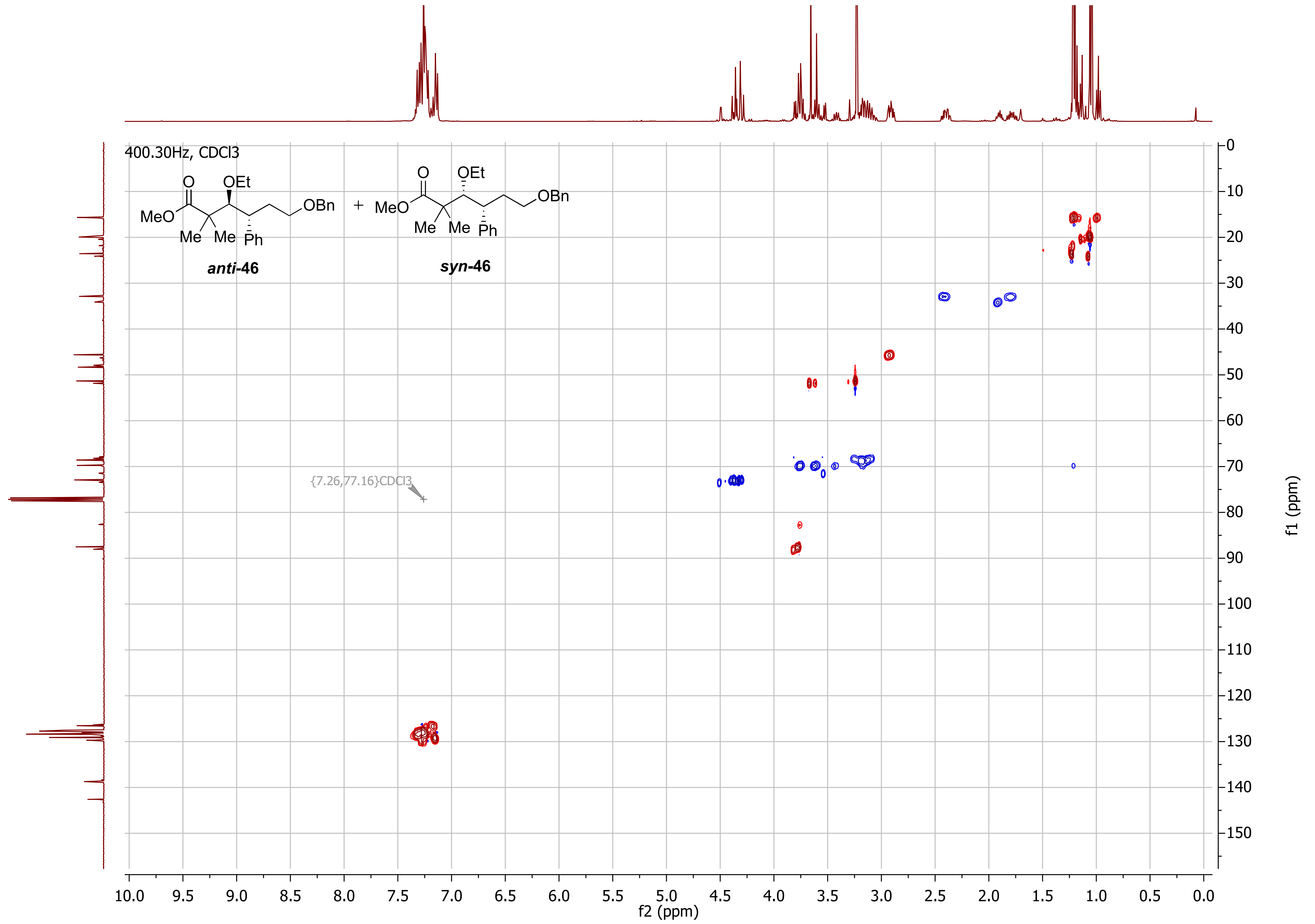
400.30Hz, CDCl3
MeO O Me Me OEt Ph OBn
anti-46
+
MeO O Me Me OEt Ph OBn
syn-46
{7.26,77.16}CDCl3
f2 (ppm)
f1 (ppm)

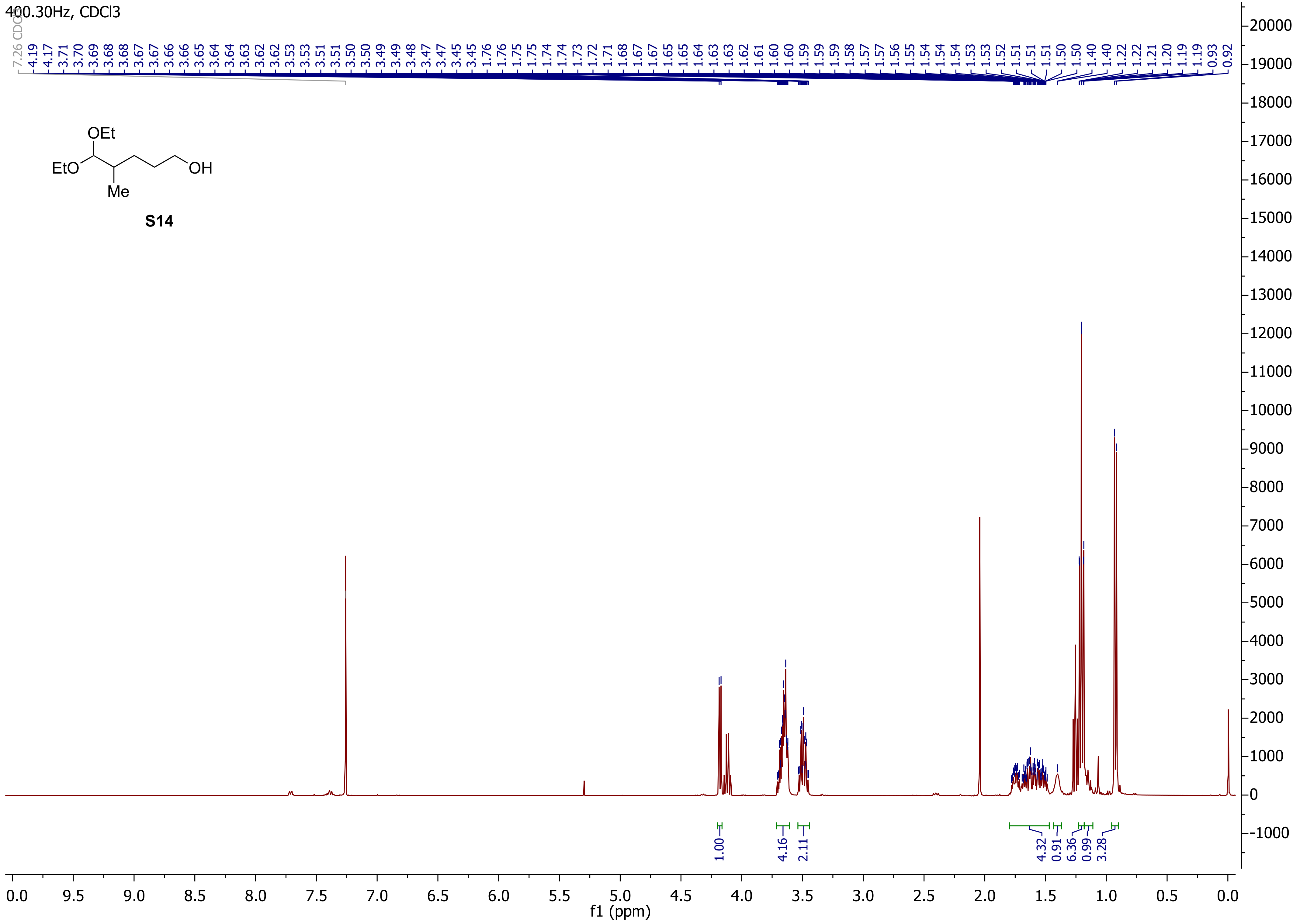

400.30Hz, CDCl3
7.26 CDCl3
4.19 4.17 3.71 3.70 3.69 3.68 3.68 3.67 3.67 3.66 3.66 3.65 3.64 3.64 3.63 3.62 3.62 3.53 3.53 3.51 3.51 3.50 3.50 3.49 3.49 3.48 3.47 3.47 3.45 3.45 1.76 1.76 1.75 1.75 1.74 1.74 1.73 1.72 1.71 1.68 1.67 1.67 1.65 1.65 1.64 1.63 1.63 1.62 1.61 1.60 1.60 1.59 1.59 1.59 1.58 1.57 1.56 1.55 1.54 1.54 1.54 1.53 1.53 1.52 1.51 1.51 1.51 1.50 1.50 1.40 1.40 1.22 1.22 1.21 1.20 1.19 1.19 0.93 0.92
OEt
EtO
OH
Me
1.00
4.16
2.11
4.32
0.91
6.36
0.99
3.28
f1 (ppm)

S14

100.67Hz, CDCl3

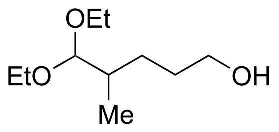

S14

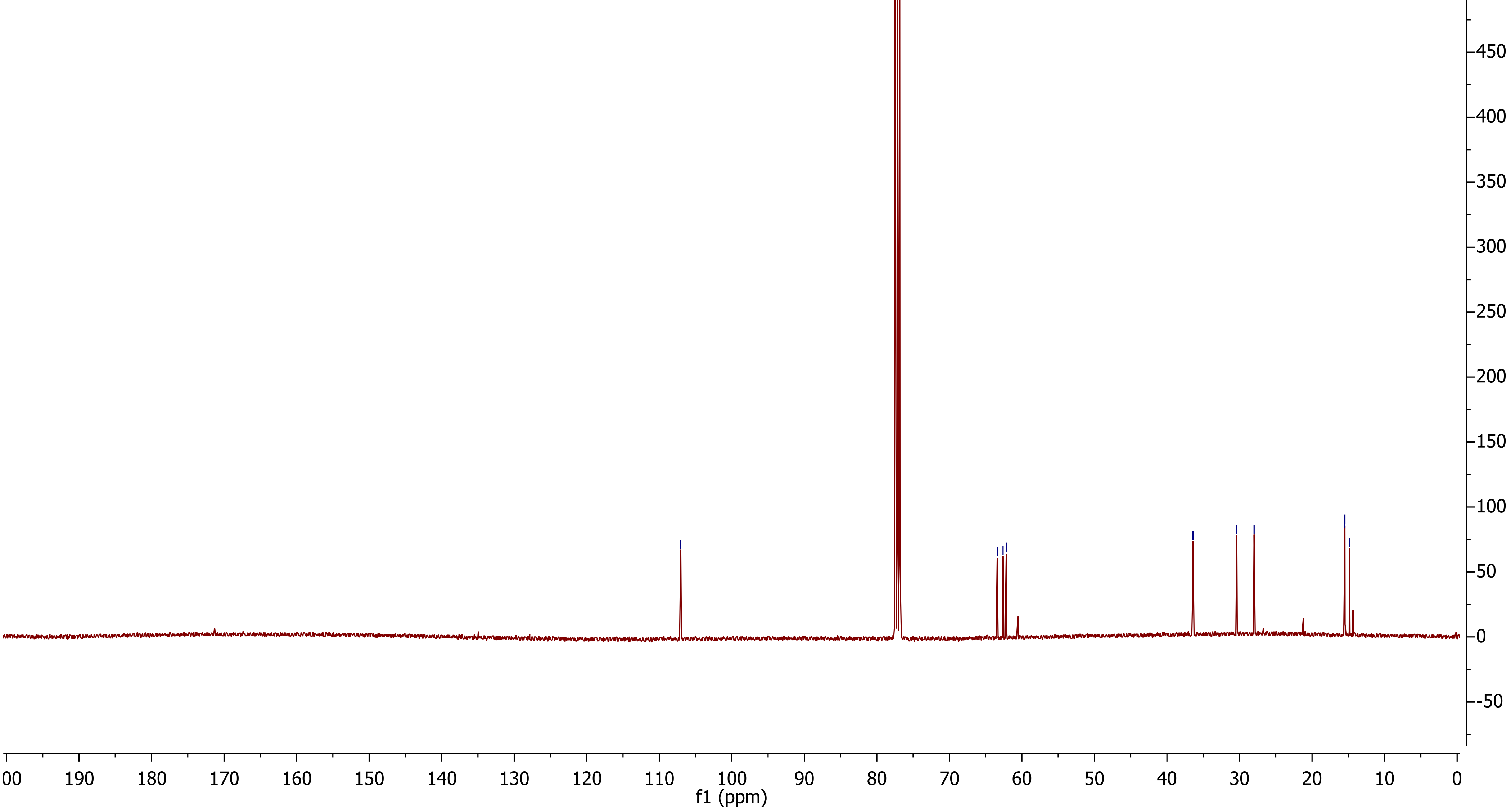

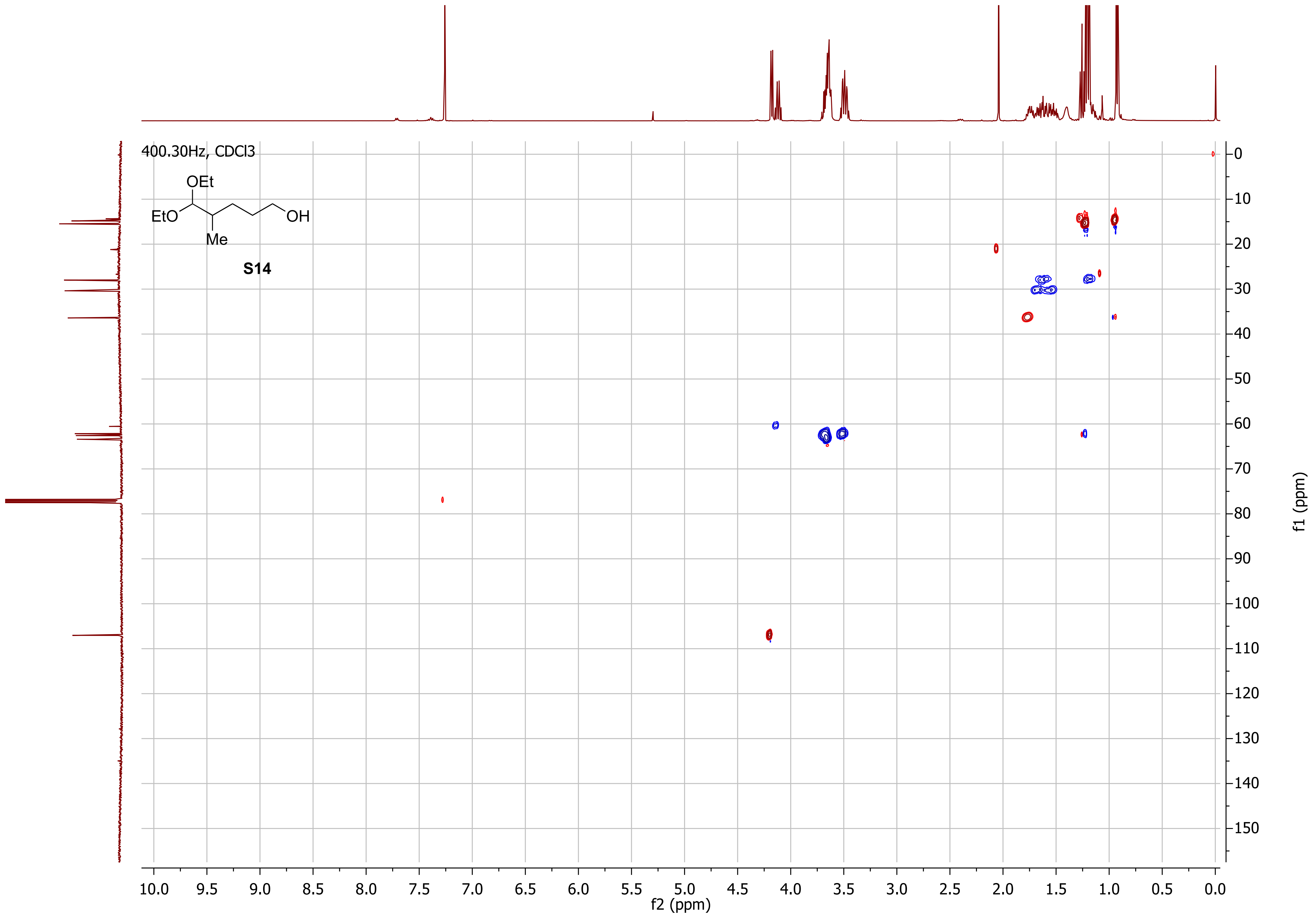
400.30Hz, CDCl3
OEt
EtO
OH
Me
S14
f2 (ppm)
f1 (ppm)

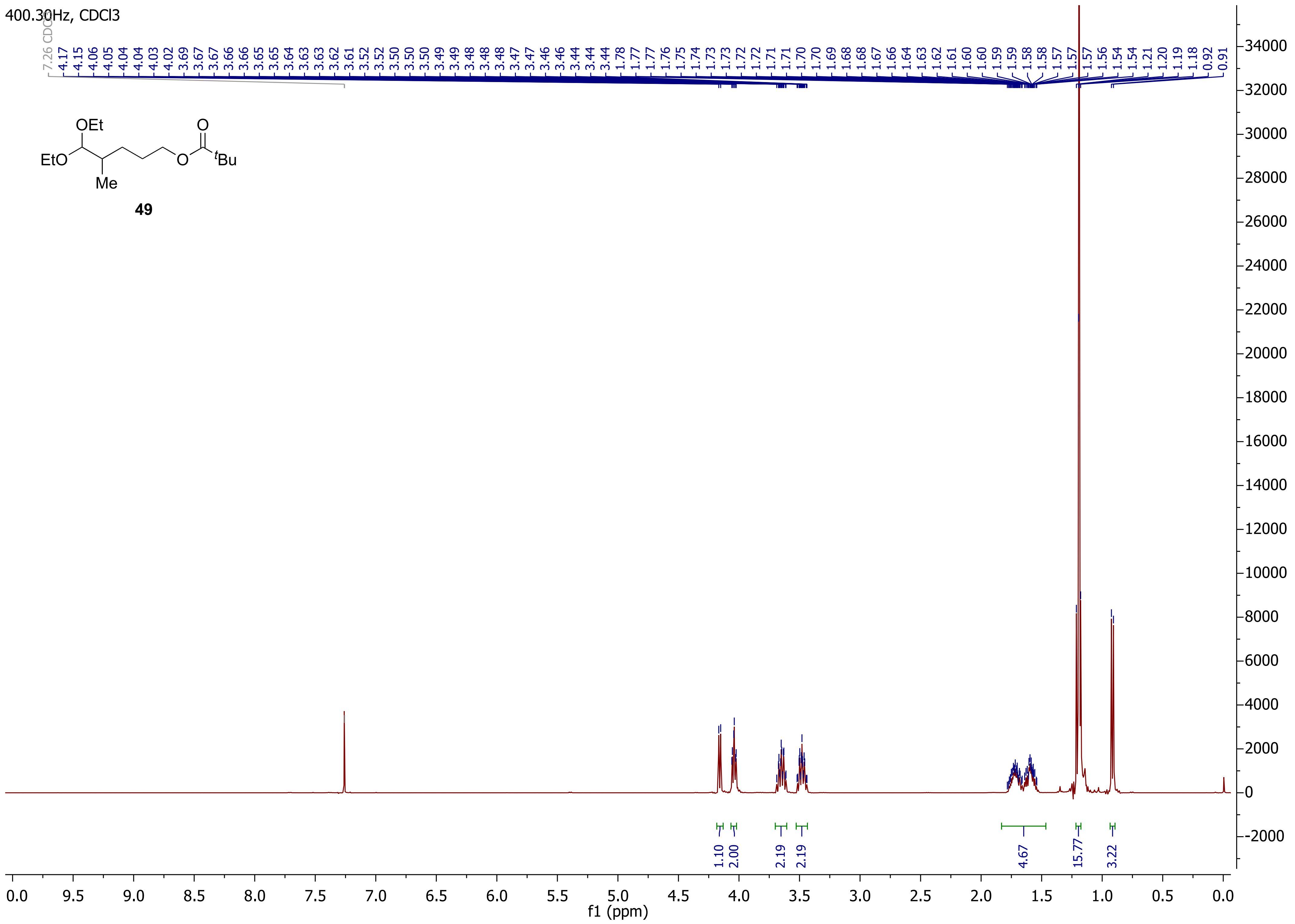
400.30Hz, CDCl3
7.26 CDCl3
4.17 4.15 4.06 4.05 4.04 4.04 4.03 4.02 3.69 3.67 3.67 3.66 3.66 3.65 3.65 3.64 3.63 3.63 3.62 3.61 3.52 3.52 3.50 3.50 3.50 3.49 3.49 3.48 3.48 3.48 3.47 3.47 3.46 3.46 3.44 3.44 3.44 1.78 1.77 1.77 1.76 1.75 1.74 1.73 1.73 1.72 1.72 1.71 1.71 1.70 1.70 1.69 1.68 1.68 1.67 1.66 1.64 1.63 1.62 1.61 1.60 1.60 1.59 1.59 1.58 1.58 1.57 1.57 1.57 1.56 1.54 1.54 1.21 1.20 1.19 1.18 0.92 0.91
OEt
EtO
Me
O
O
tBu
49
1.10
2.00
2.19
2.19
4.67
15.77
3.22
f1 (ppm)

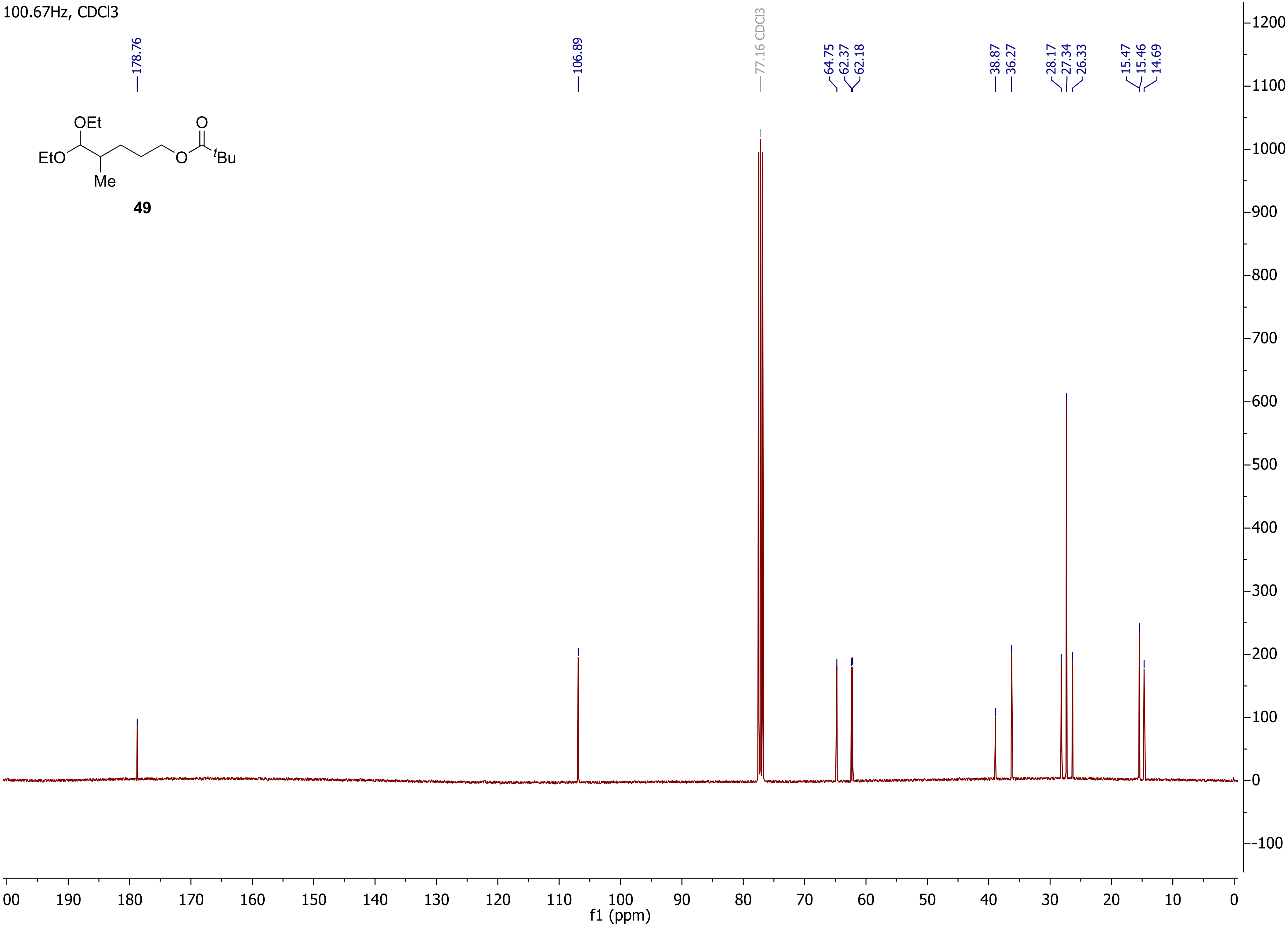
100.67Hz, CDCl3
178.76
106.89
77.16 CDCl3
64.75
62.37
62.18
38.87
36.27
28.17
27.34
26.33
15.47
15.46
14.69
OEt
EtO
Me
O
O
tBu
49
1200
1100
1000
900
800
700
600
500
400
300
200
100
0
-100
00
190
180
170
160
150
140
130
120
110
100
90
80
70
60
50
40
30
20
10
0
f1 (ppm)

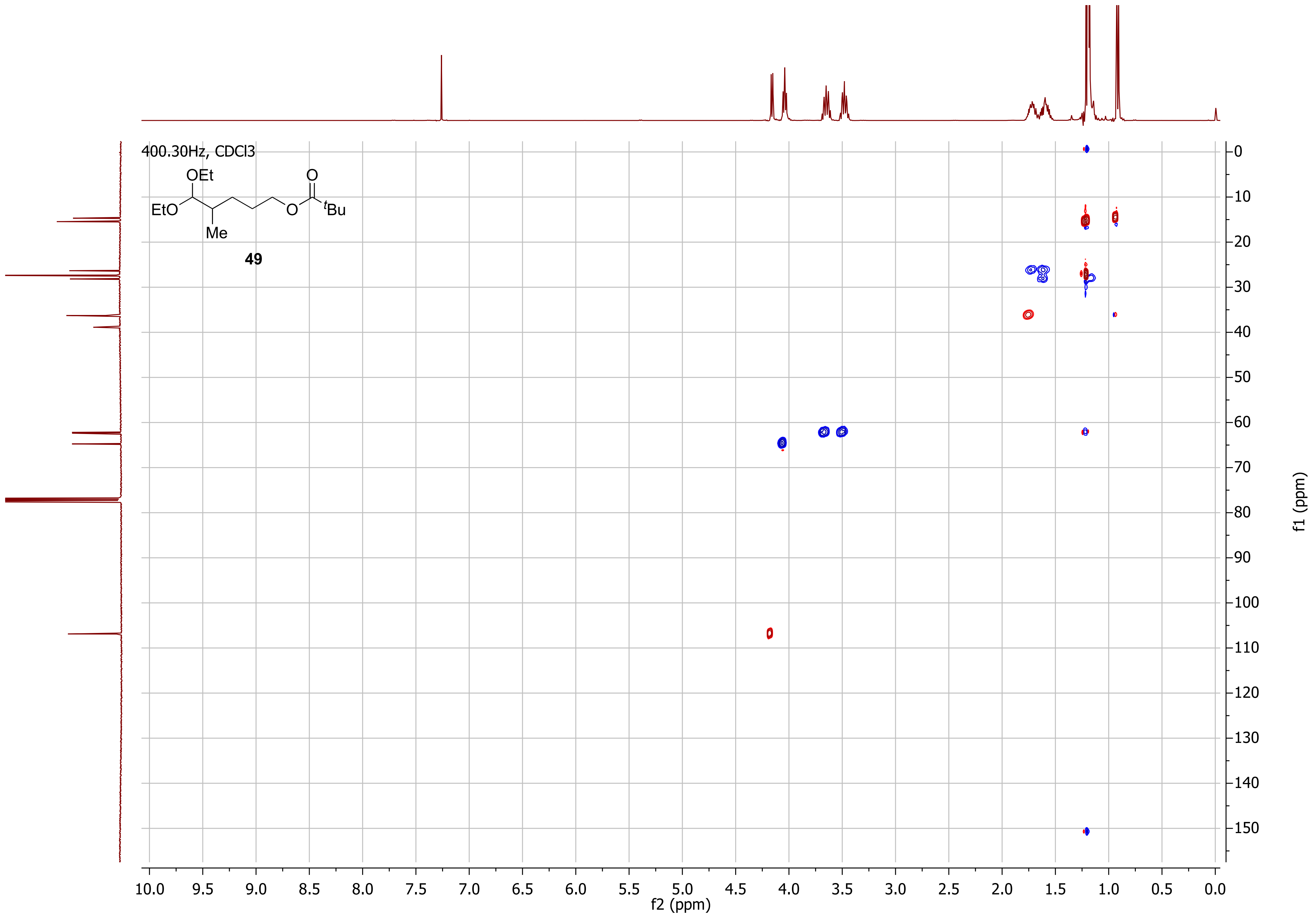

400.30Hz, CDCl3
OEt
EtO
Me
O
O
tBu
49
f2 (ppm)
f1 (ppm)

400.30Hz, CDCl3

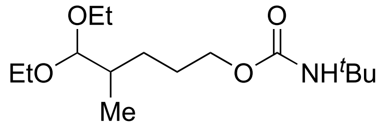

50

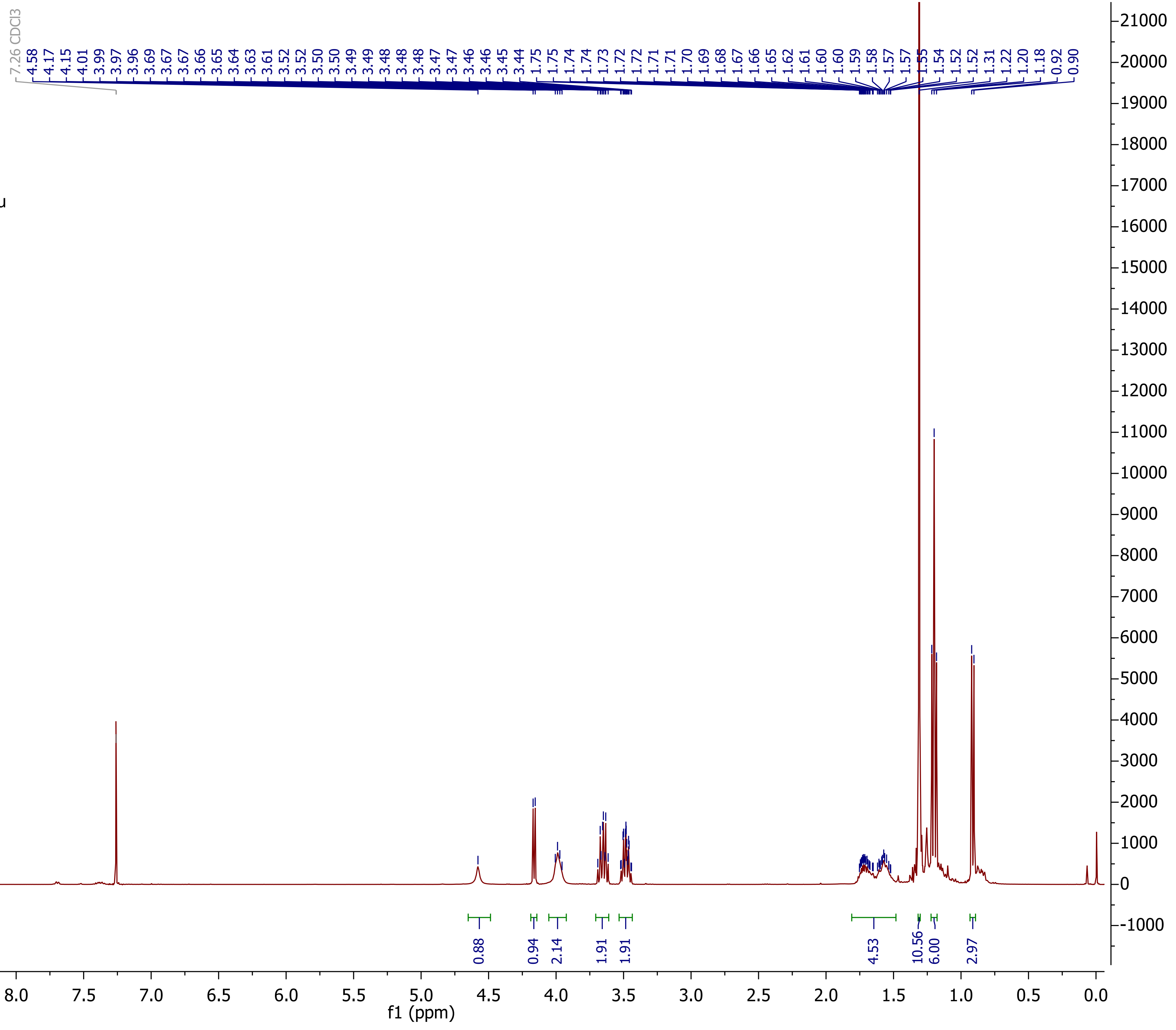

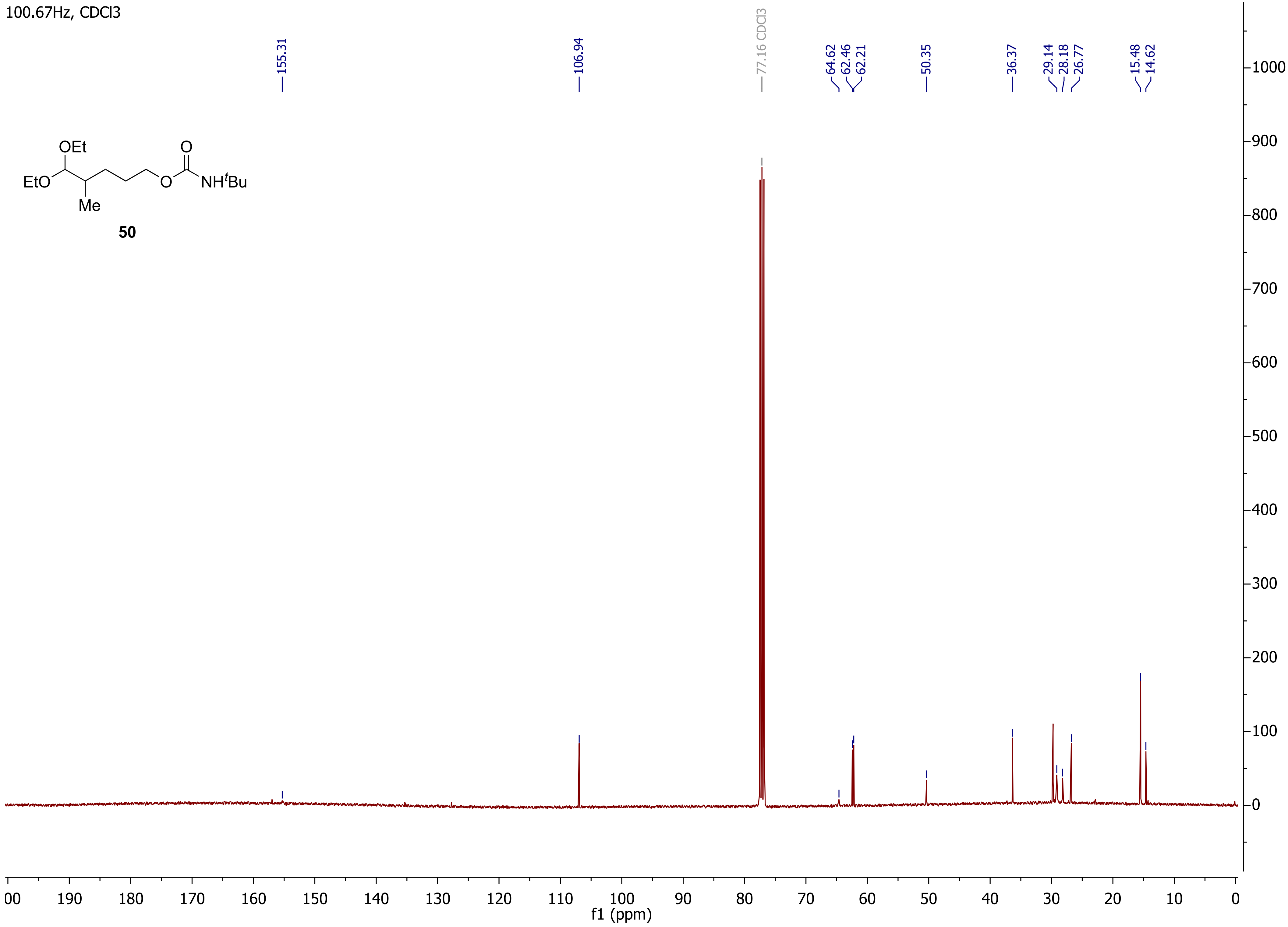
100.67Hz, CDCl3
155.31
106.94
77.16 CDCl3
64.62
62.46
62.21
50.35
36.37
29.14
28.18
26.77
15.48
14.62
OEt
EtO
Me
O
O
NH^{t}Bu
50
f1 (ppm)

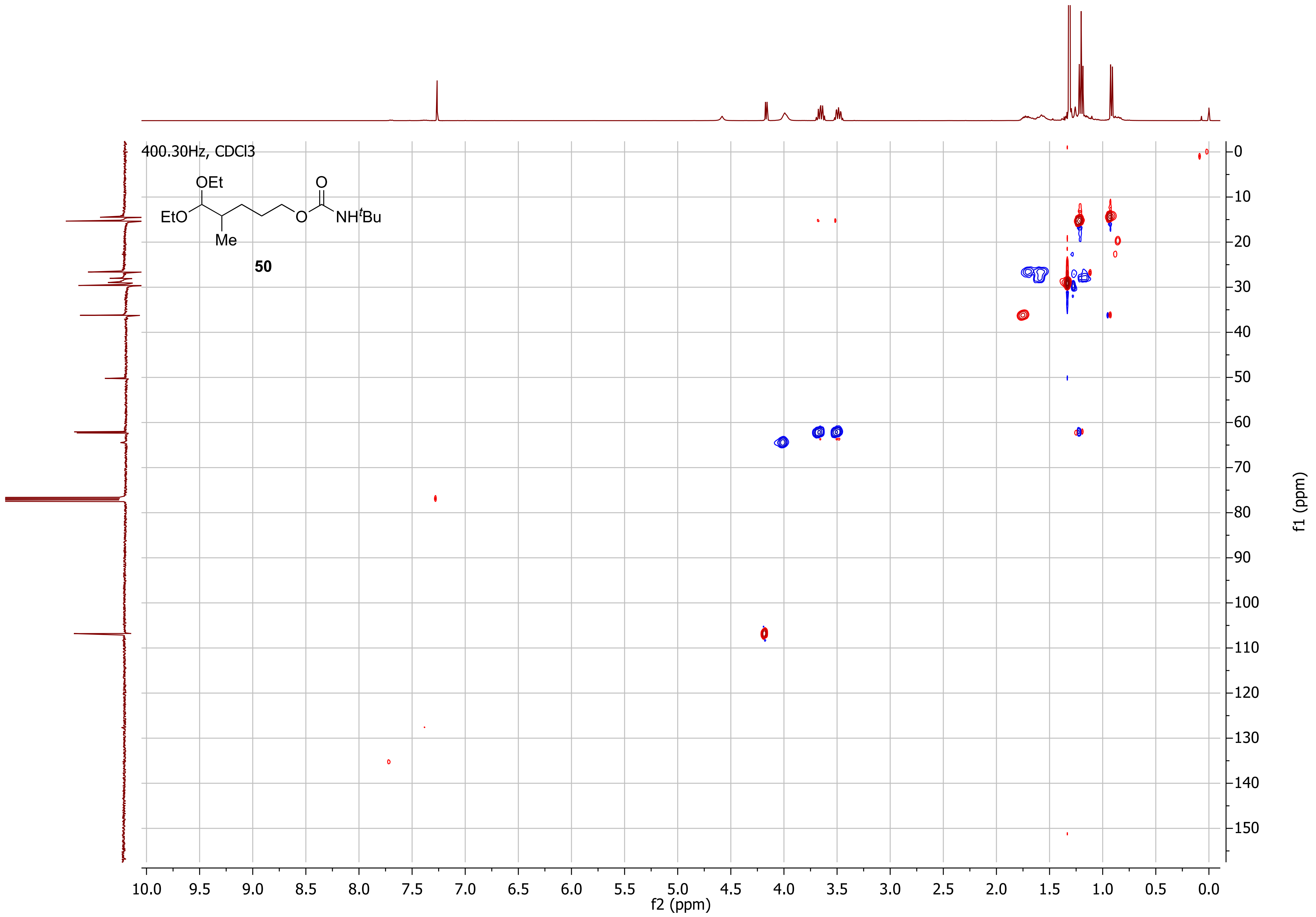
400.30Hz, CDCl3
OEt
EtO
Me
O
O
NH^{t}Bu
50
f2 (ppm)
f1 (ppm)

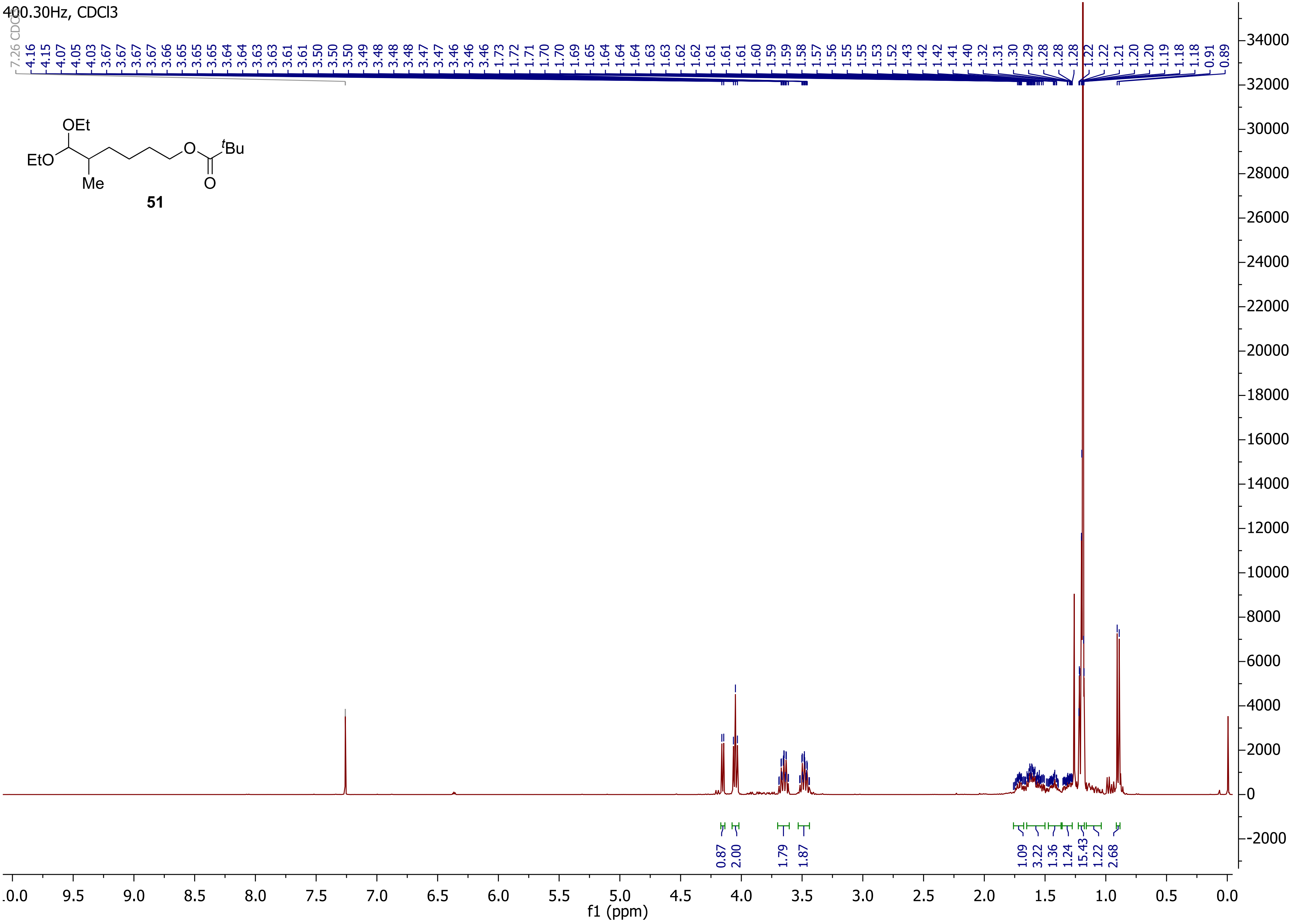

400.30Hz, CDCl3
7.26 CDCl3
4.16
4.15
4.07
4.05
4.03
3.67
3.67
3.67
3.67
3.66
3.65
3.65
3.65
3.64
3.64
3.63
3.63
3.61
3.61
3.50
3.50
3.50
3.49
3.48
3.48
3.48
3.47
3.47
3.46
3.46
3.46
1.73
1.72
1.71
1.70
1.70
1.69
1.65
1.64
1.64
1.64
1.63
1.63
1.62
1.62
1.61
1.61
1.61
1.60
1.59
1.59
1.58
1.57
1.56
1.55
1.55
1.53
1.52
1.43
1.42
1.42
1.41
1.40
1.32
1.31
1.30
1.29
1.28
1.28
1.28
1.22
1.22
1.21
1.20
1.20
1.19
1.18
1.18
0.91
0.89
OEt
EtO
Me
O
tBu
O
51
0.87
2.00
1.79
1.87
1.09
3.22
1.36
1.24
15.43
1.22
2.68
f1 (ppm)

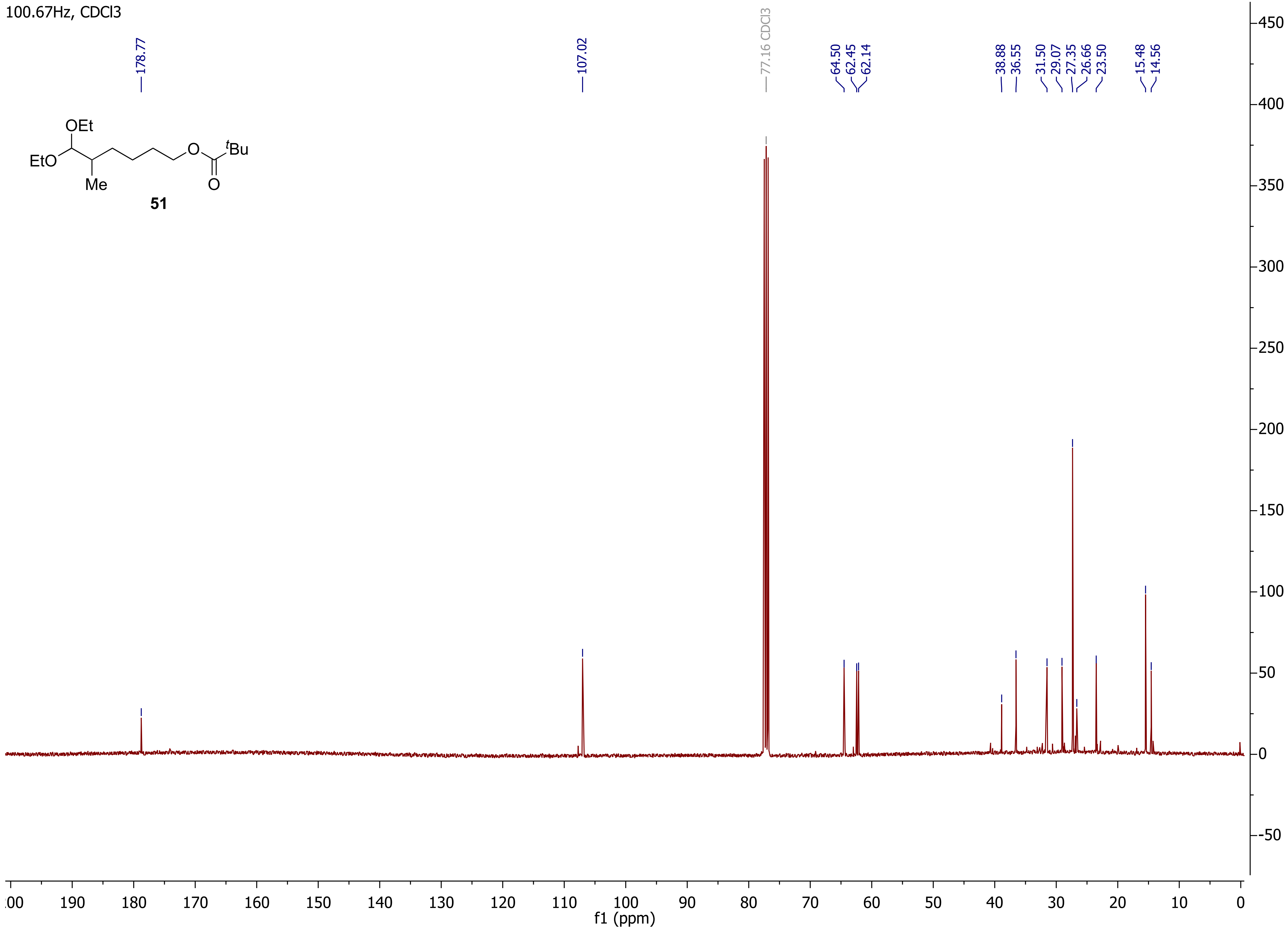

100.67Hz, CDCl3
178.77
107.02
77.16 CDCl3
64.50
62.45
62.14
38.88
36.55
31.50
29.07
27.35
26.66
23.50
15.48
14.56
OEt
EtO
Me
O
tBu
O
51
f1 (ppm)

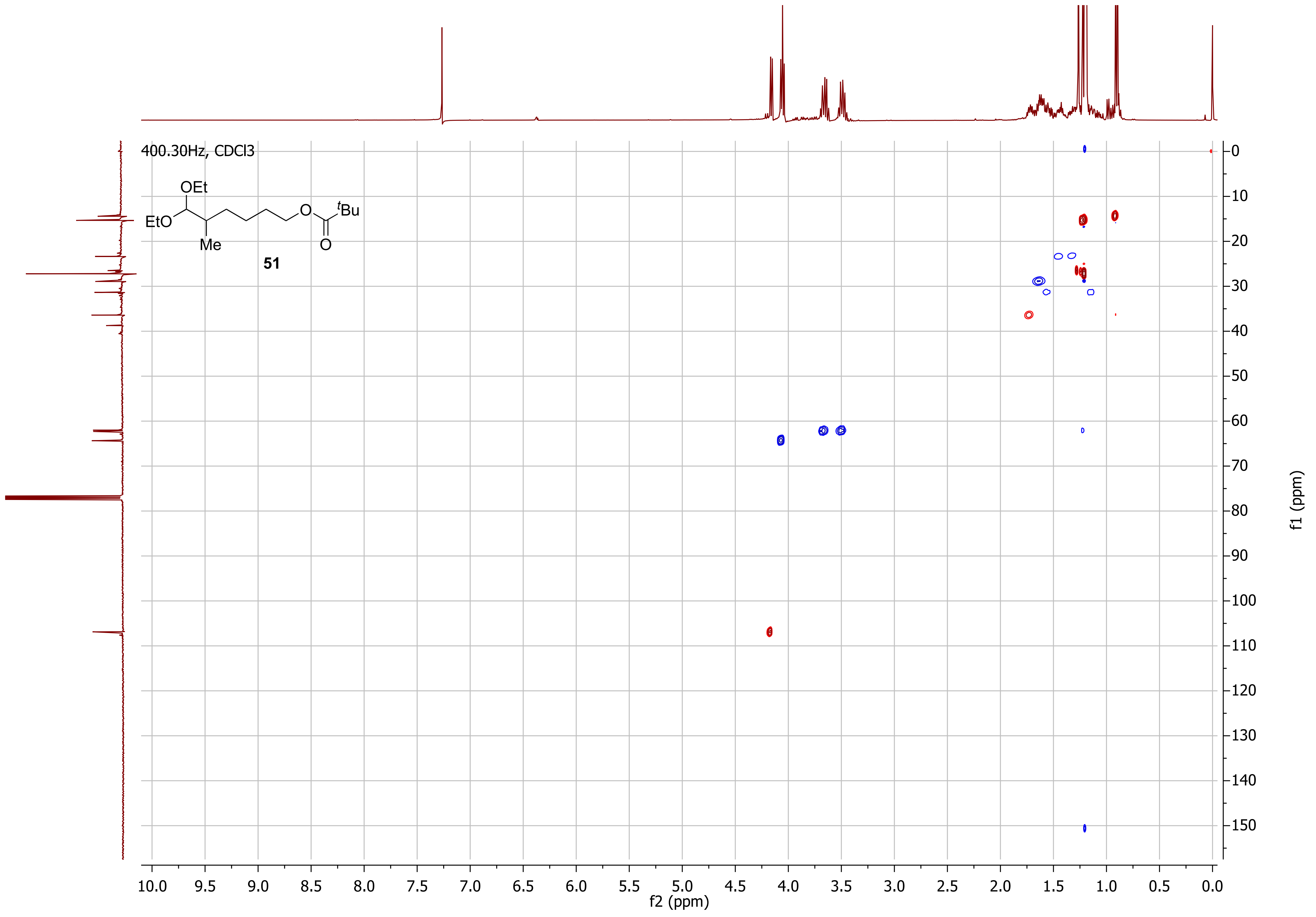
400.30Hz, CDCl3
OEt
EtO
Me
O
tBu
O
51
f2 (ppm)
f1 (ppm)

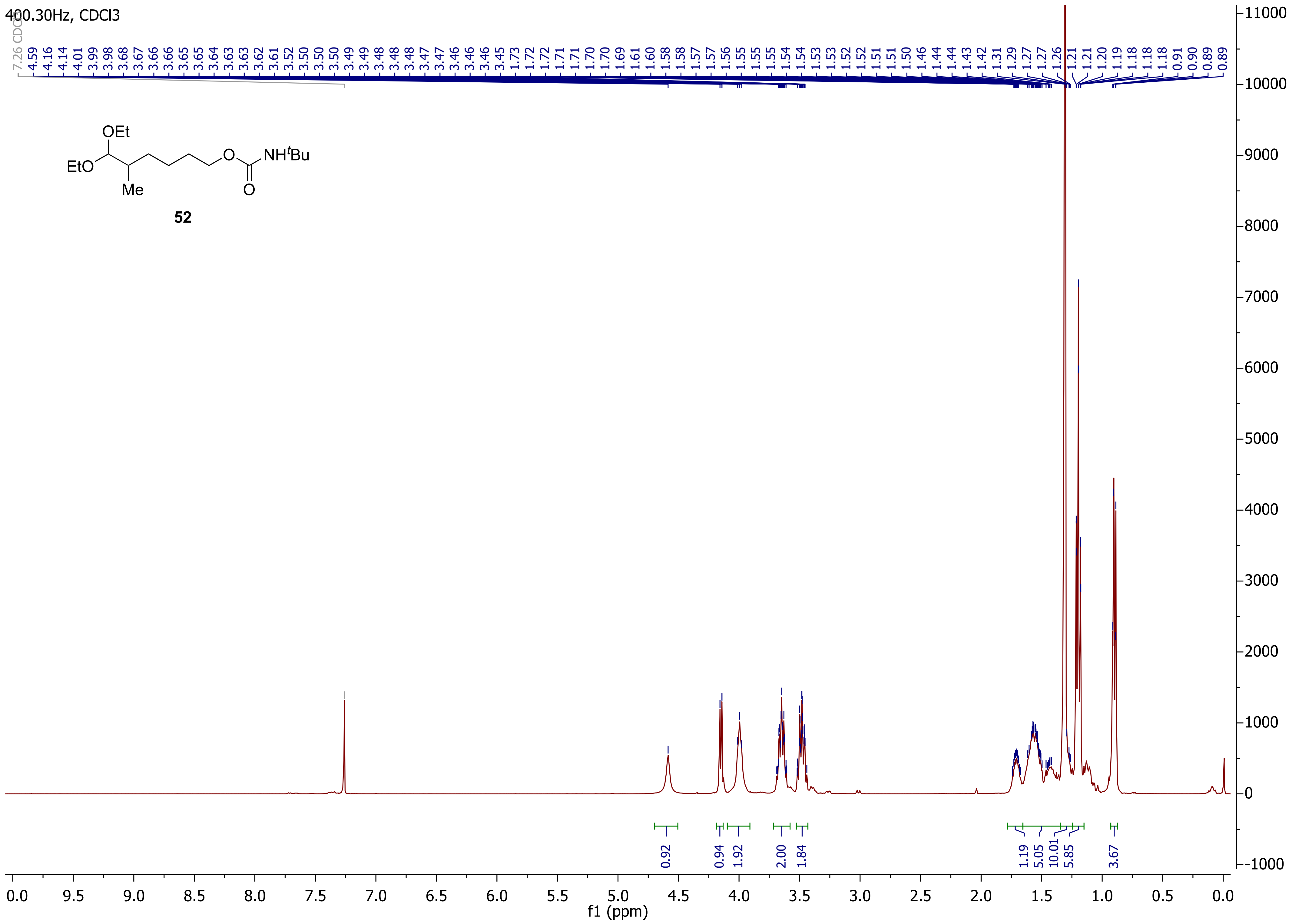

400.30Hz, CDCl3
7.26 CDCl3
4.59 4.16 4.14 4.01 3.99 3.98 3.68 3.67 3.66 3.66 3.65 3.65 3.64 3.63 3.63 3.62 3.61 3.52 3.50 3.50 3.50 3.49 3.49 3.48 3.48 3.48 3.47 3.47 3.46 3.46 3.46 3.45 1.73 1.72 1.72 1.71 1.71 1.70 1.70 1.69 1.61 1.60 1.58 1.58 1.57 1.57 1.56 1.55 1.55 1.55 1.54 1.54 1.53 1.53 1.52 1.52 1.51 1.51 1.50 1.46 1.44 1.44 1.43 1.42 1.31 1.29 1.27 1.27 1.26 1.21 1.21 1.20 1.19 1.18 1.18 1.18 0.91 0.90 0.89 0.89
OEt
EtO
Me
O
NH^{t}Bu
O
52
0.92
0.94
1.92
2.00
1.84
1.19
5.05
10.01
5.85
3.67
f1 (ppm)

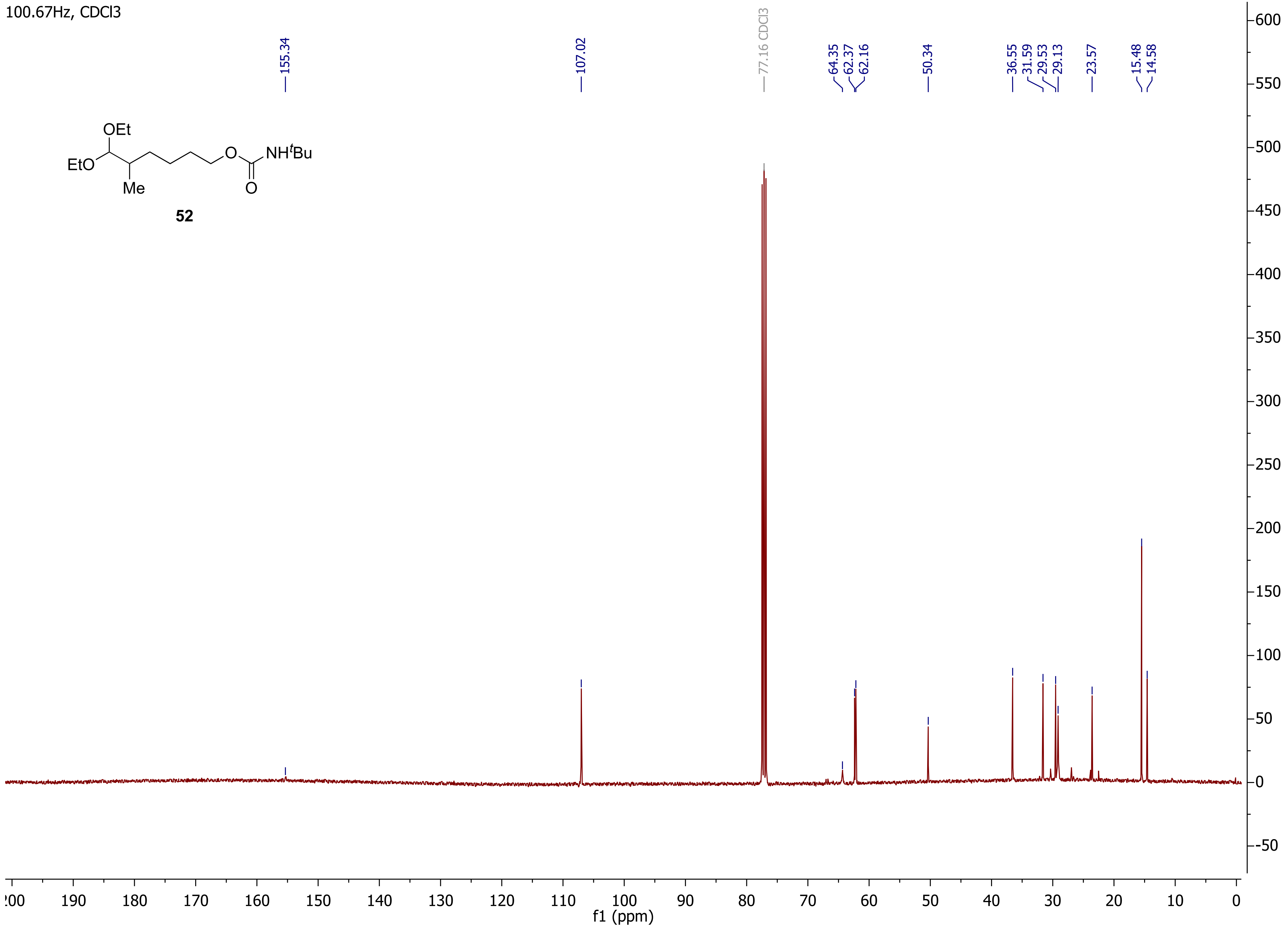
100.67Hz, CDCl3
155.34
107.02
77.16 CDCl3
64.35
62.37
62.16
50.34
36.55
31.59
29.53
29.13
23.57
15.48
14.58
OEt
EtO
NH^{t}Bu
O
O
Me
52
f1 (ppm)

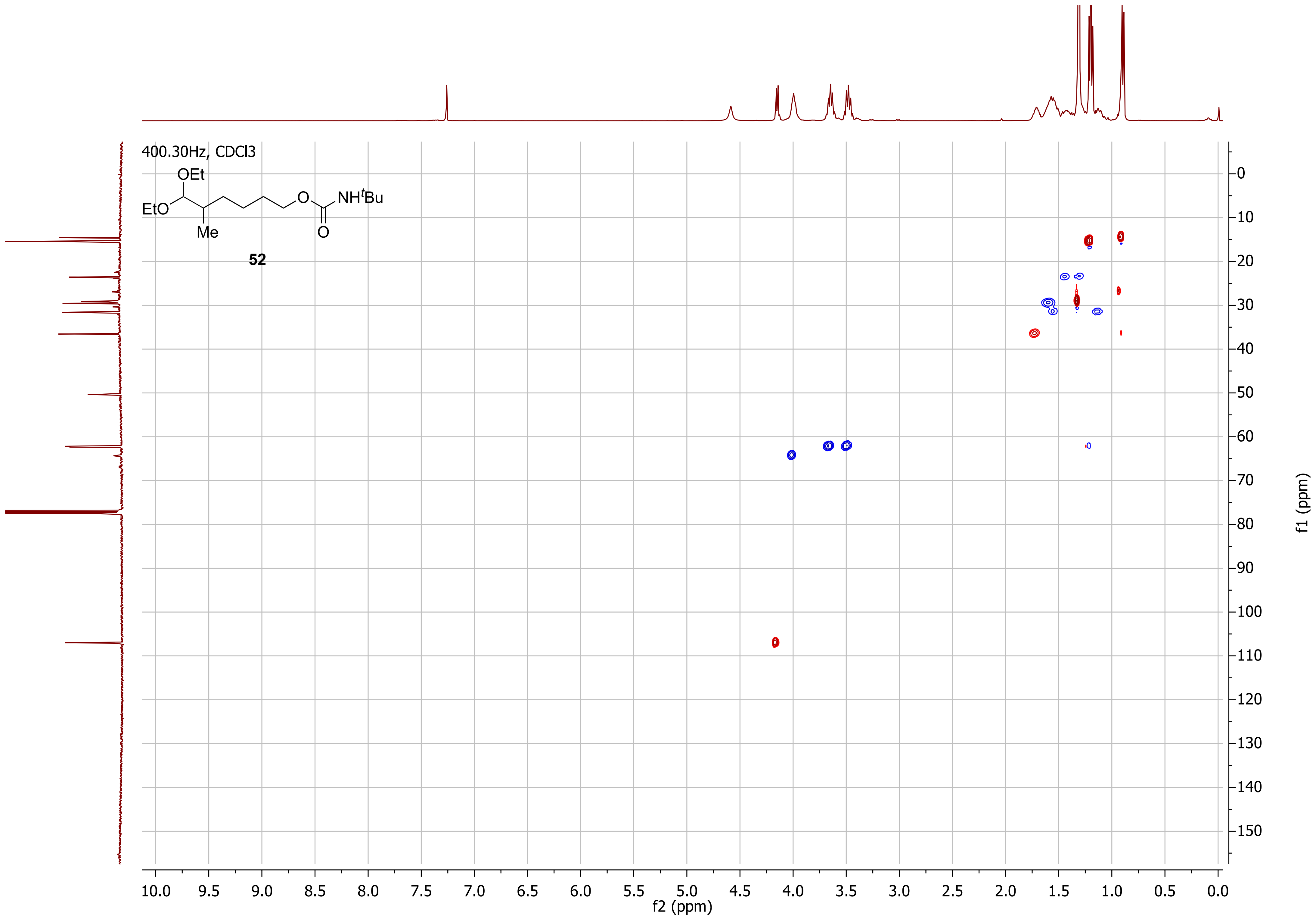
400.30Hz, CDCl3
OEt
EtO
NHtBu
Me
O
O
52
f2 (ppm)
f1 (ppm)

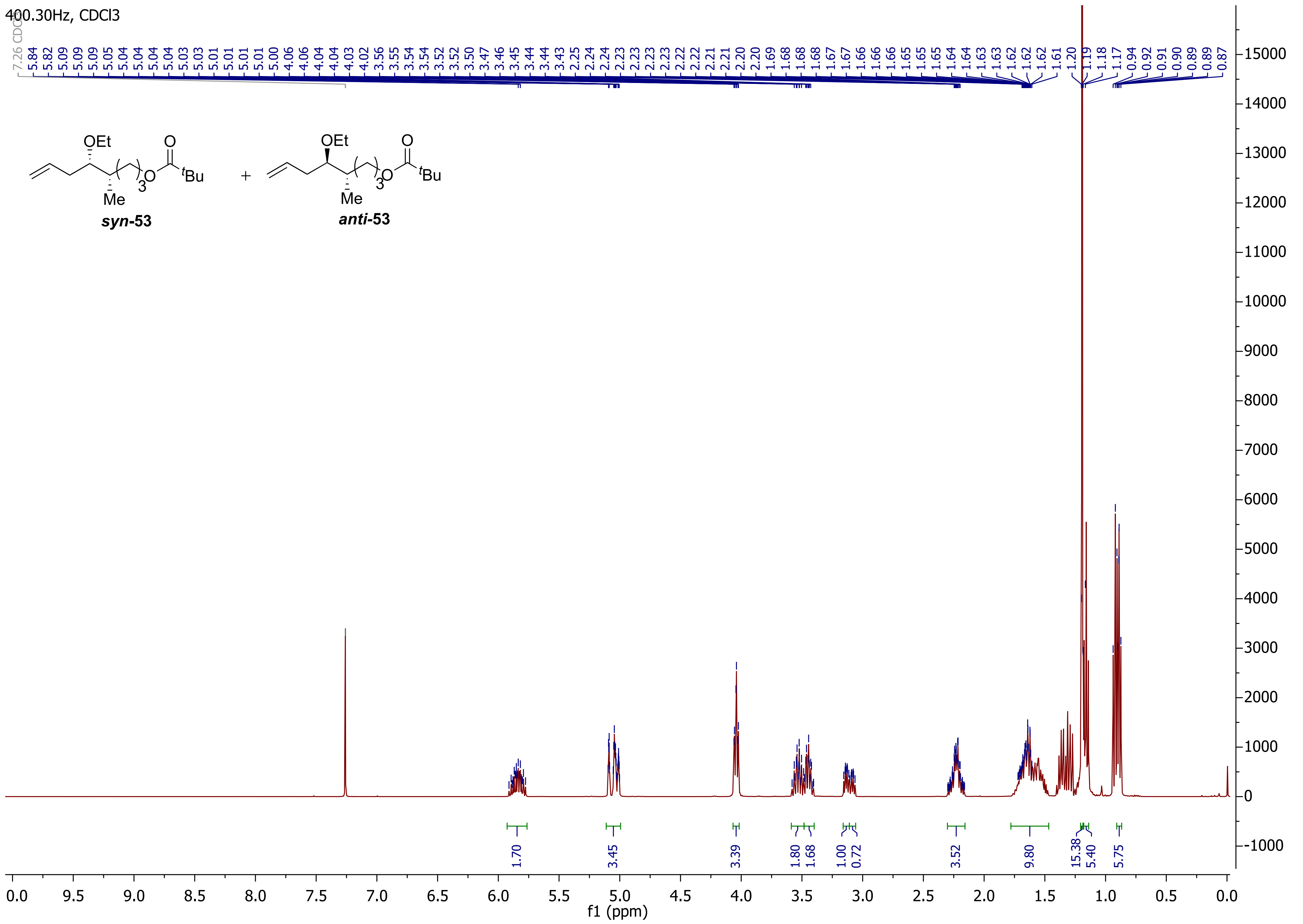
400.30Hz, CDCl3
OEt
O
Me
3
tBu
syn-53
anti-53
f1 (ppm)
1.70
3.45
3.39
1.80
1.68
1.00
0.72
3.52
9.80
15.38
5.40
5.75

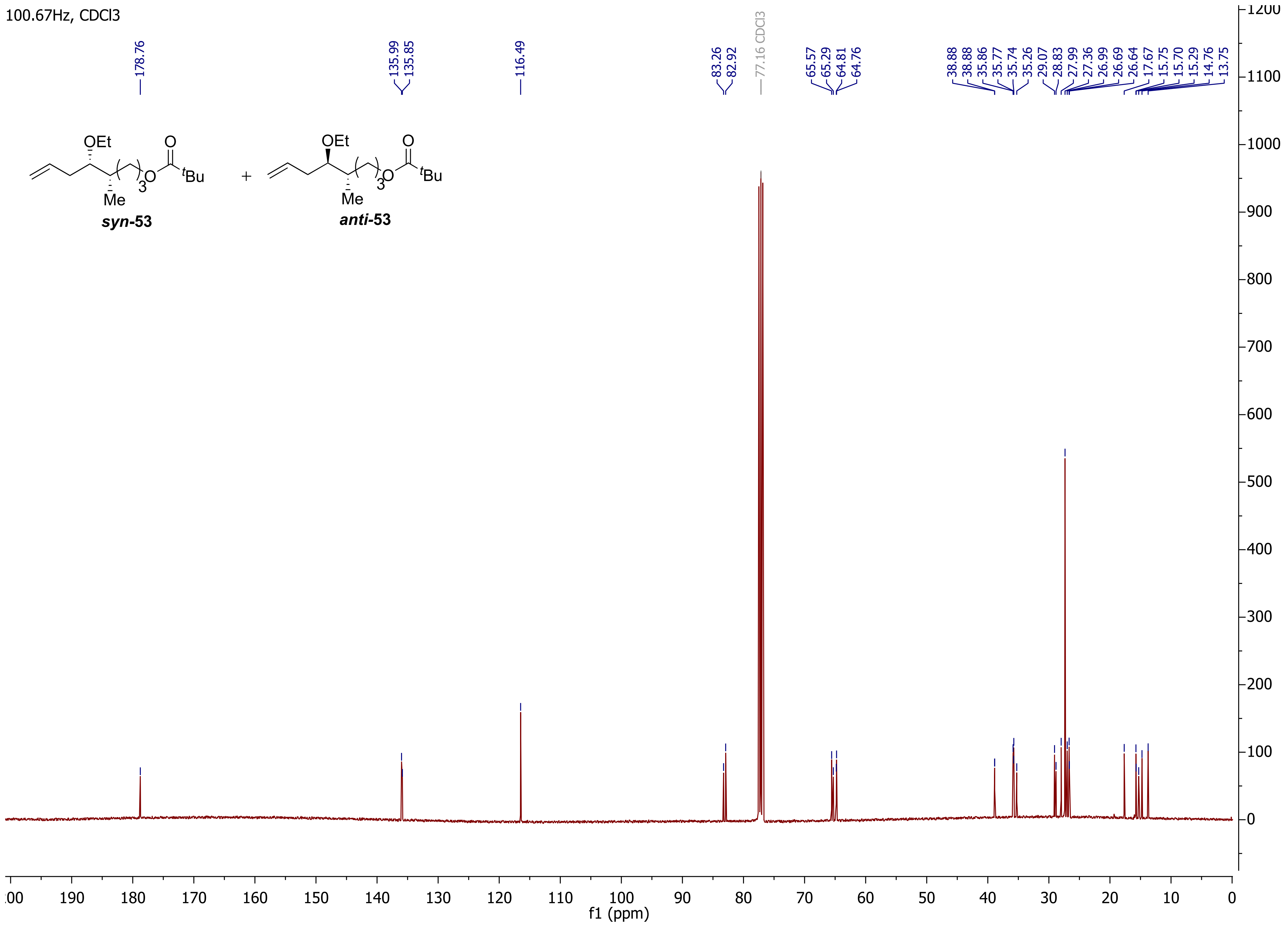
100.67Hz, CDCl3
178.76
135.99
135.85
116.49
83.26
82.92
77.16 CDCl3
65.57
65.29
64.81
64.76
38.88
38.88
35.86
35.77
35.74
35.26
29.07
28.83
27.99
27.36
26.99
26.69
26.64
17.67
15.75
15.70
15.29
14.76
13.75
OEt
O
O
tBu
3
Me
syn-53
anti-53
f1 (ppm)

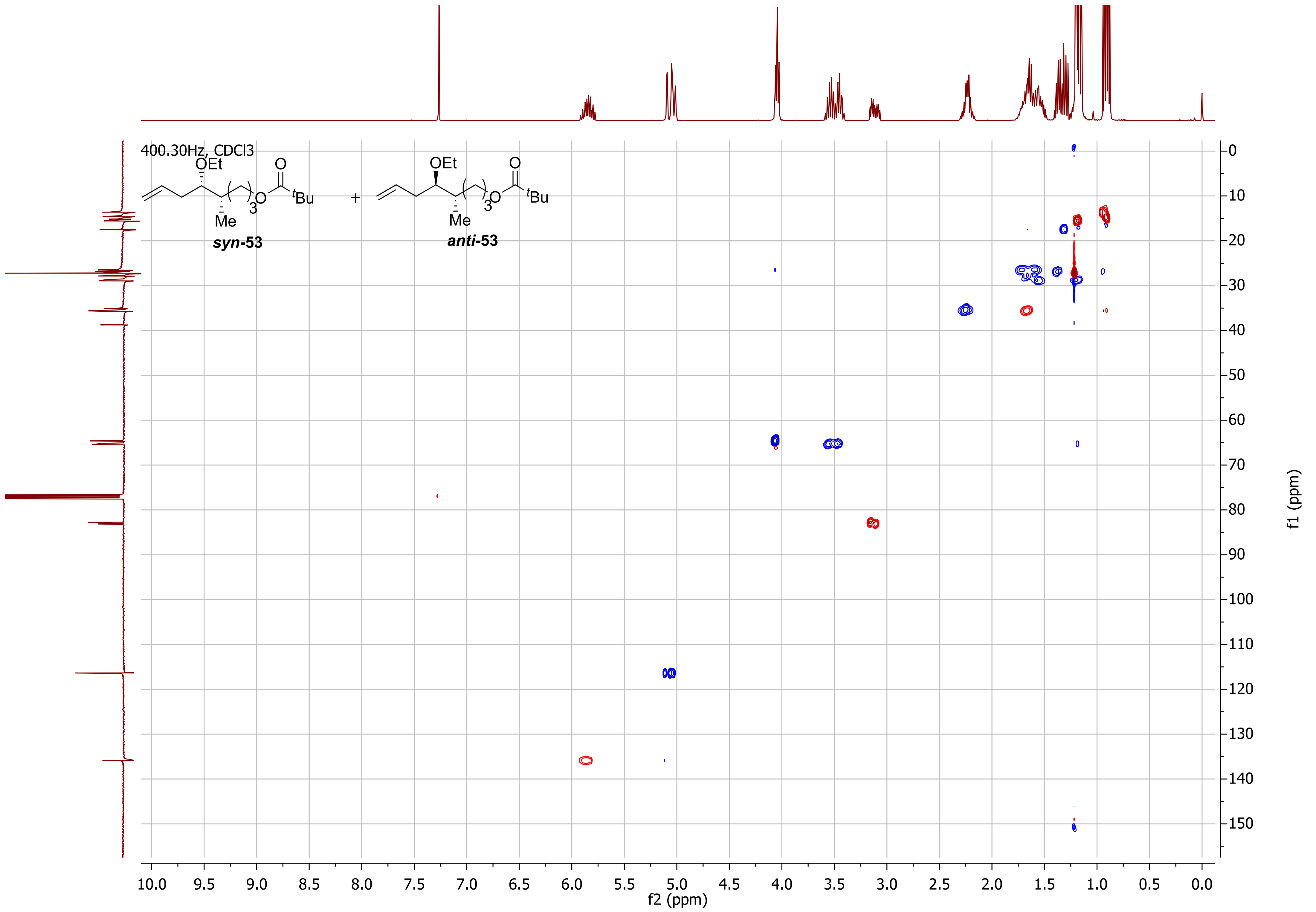
400.30Hz, CDCl3
syn-53
anti-53
f2 (ppm)
f1 (ppm)

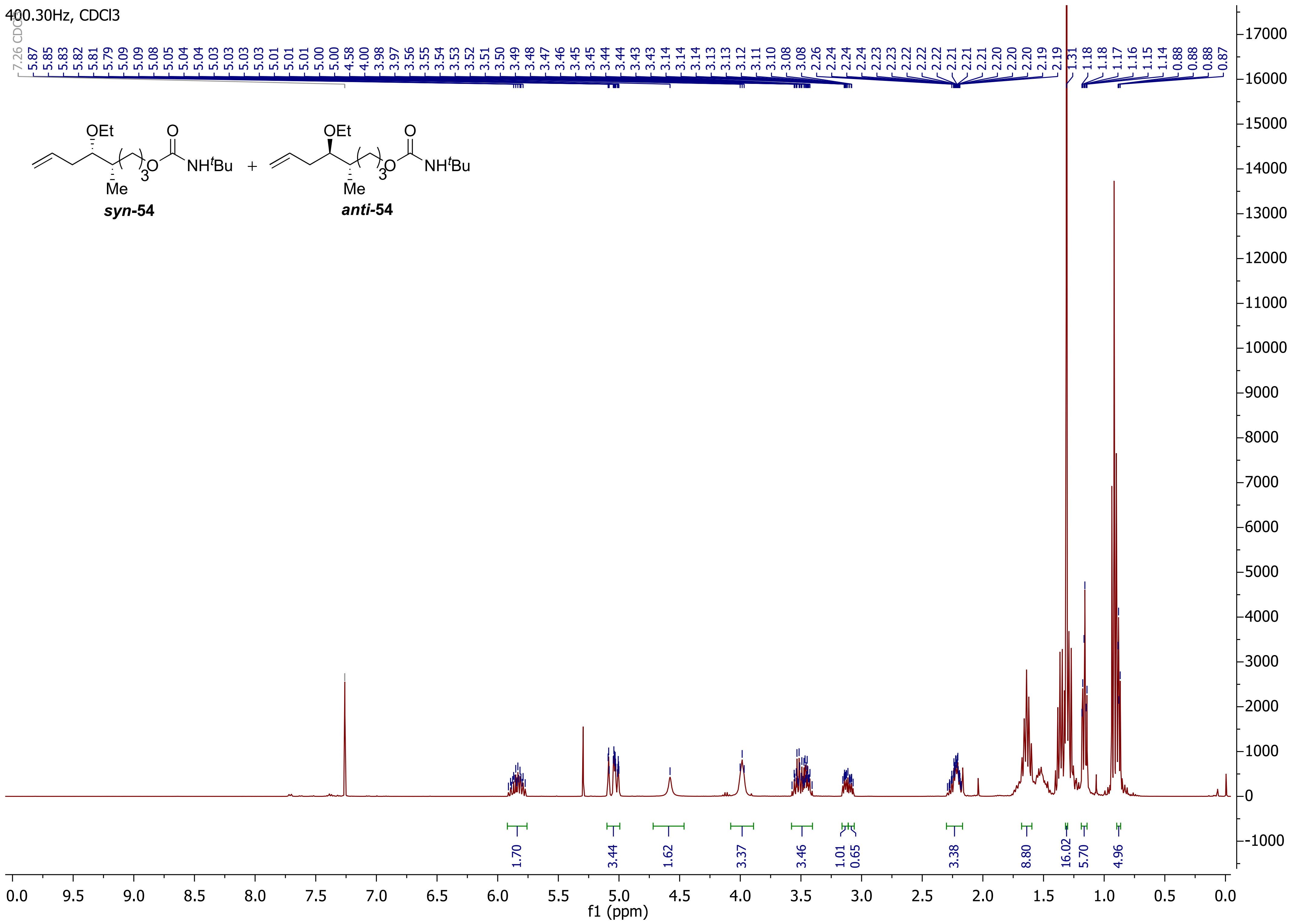
400.30Hz, CDCl3
syn-54
anti-54
OEt
Me
NH^{t}Bu
f1 (ppm)
1.70
3.44
1.62
3.37
3.46
1.01
0.65
3.38
8.80
16.02
5.70
4.96

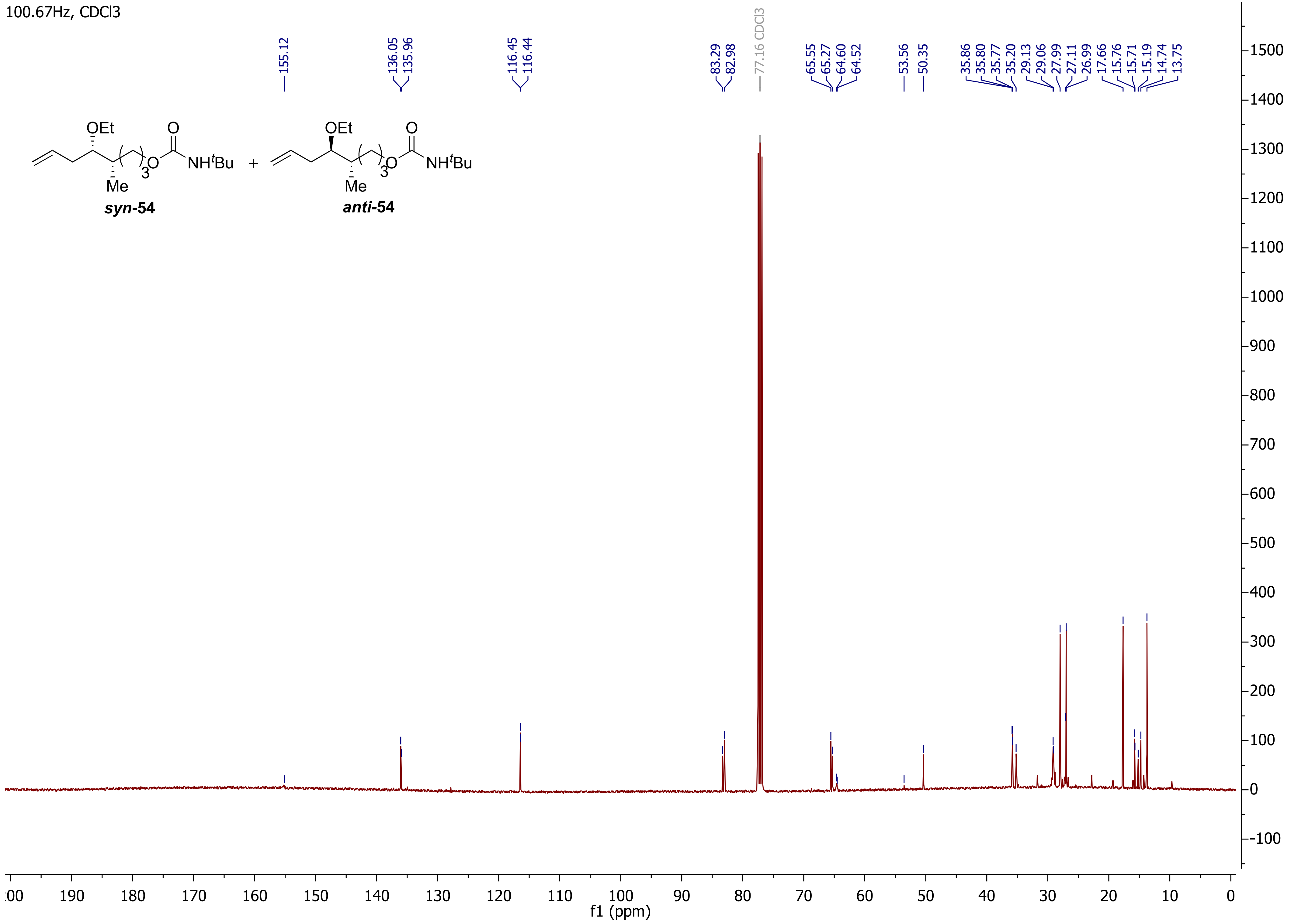
100.67Hz, CDCl3
155.12
136.05
135.96
116.45
116.44
83.29
82.98
77.16 CDCl3
65.55
65.27
64.60
64.52
53.56
50.35
35.86
35.80
35.77
35.20
29.13
29.06
27.99
27.11
26.99
17.66
15.76
15.71
15.19
14.74
13.75
OEt
O
NH^{t}Bu
Me
syn-54
anti-54
f1 (ppm)

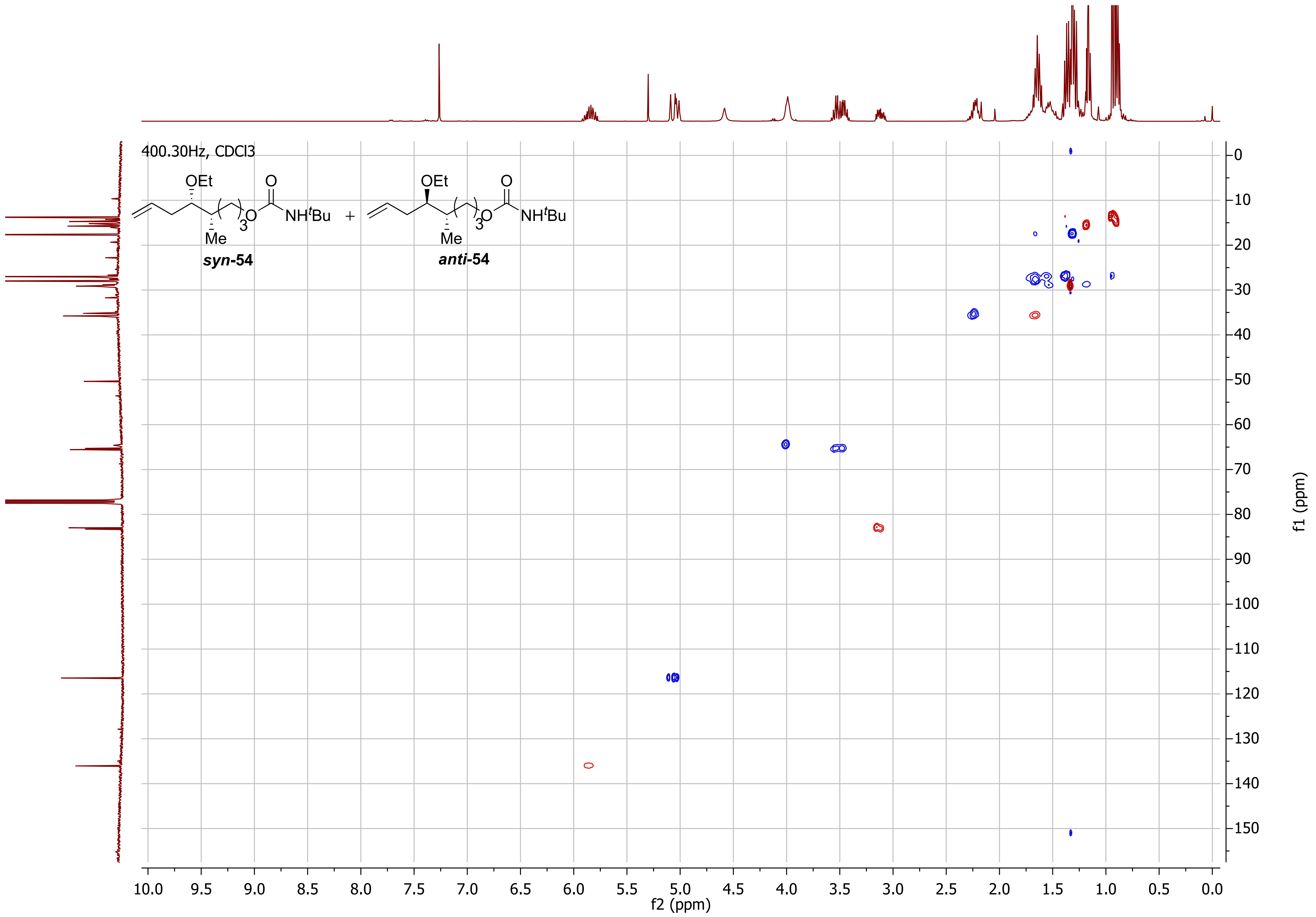
400.30Hz, CDCl3
OEt
O
NH^{t}Bu
Me
3
syn-54
anti-54
f2 (ppm)
f1 (ppm)

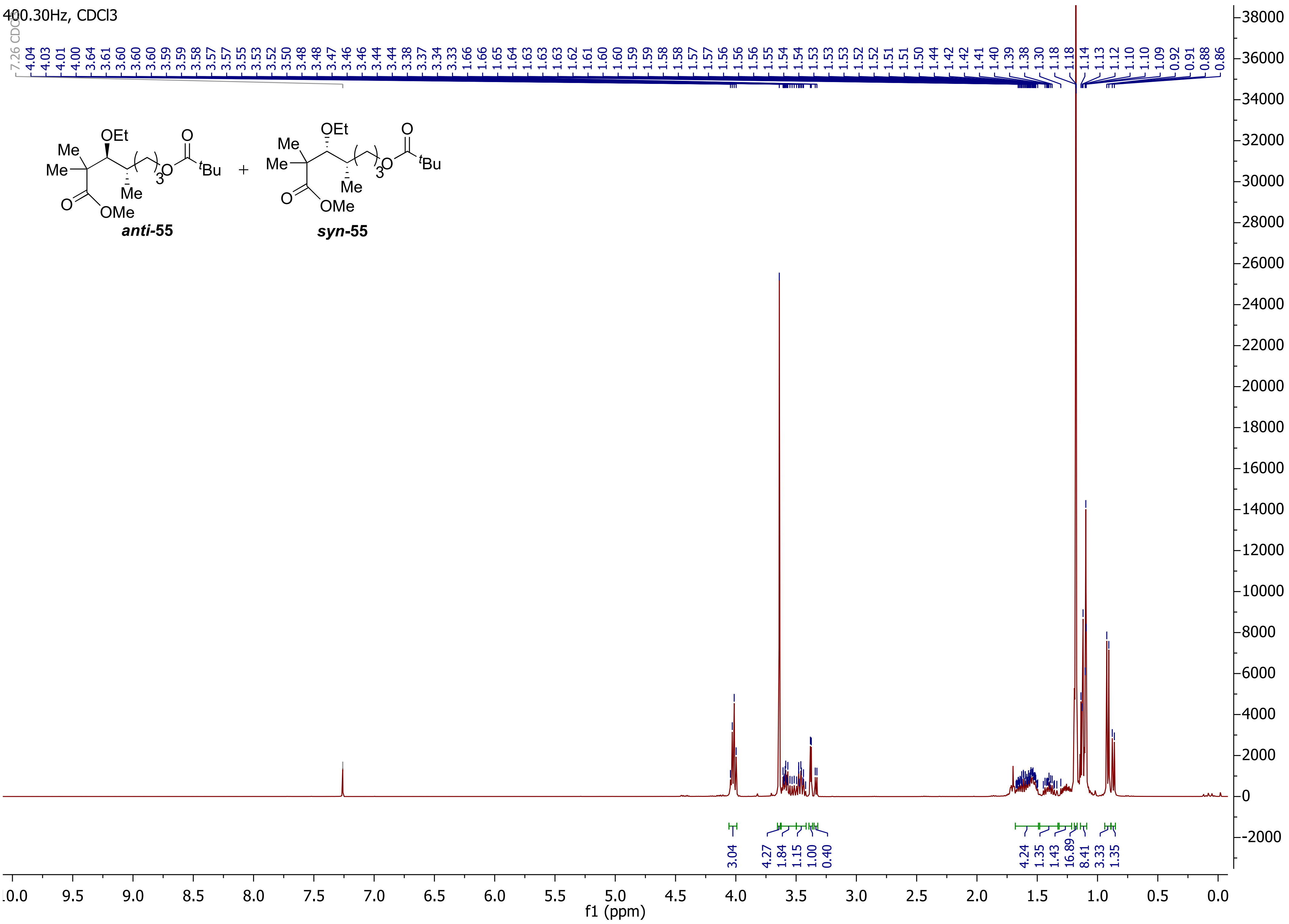

400.30Hz, CDCl3
7.26 CDCl3
4.04 4.03 4.01 4.00 3.64 3.61 3.60 3.60 3.60 3.59 3.59 3.58 3.57 3.57 3.55 3.53 3.52 3.50 3.48 3.48 3.47 3.46 3.46 3.44 3.44 3.38 3.37 3.34 3.33 1.66 1.66 1.65 1.64 1.63 1.63 1.63 1.62 1.61 1.60 1.60 1.59 1.59 1.58 1.58 1.57 1.57 1.56 1.56 1.56 1.55 1.54 1.54 1.53 1.53 1.53 1.52 1.52 1.51 1.51 1.50 1.44 1.42 1.42 1.41 1.40 1.39 1.38 1.30 1.18 1.18 1.14 1.13 1.12 1.10 1.10 1.09 0.92 0.91 0.88 0.86
OEt
Me
Me
O
tBu
Me
O
OMe
anti-55
+
syn-55
3.04
4.27
1.84
1.15
1.00
0.40
4.24
1.35
1.43
16.89
8.41
3.33
1.35
f1 (ppm)

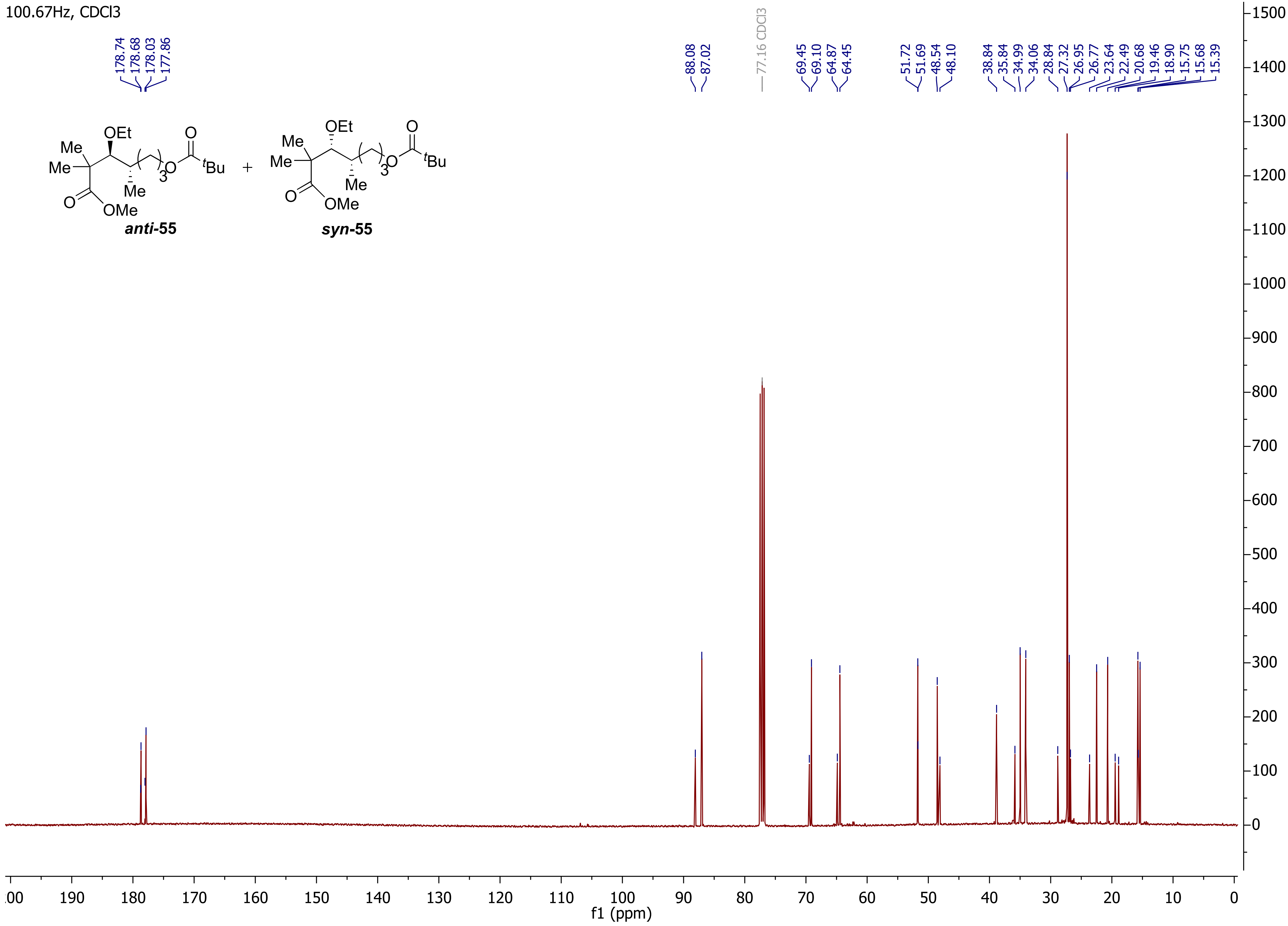

100.67Hz, CDCl3
178.74
178.68
178.03
177.86
88.08
87.02
77.16 CDCl3
69.45
69.10
64.87
64.45
51.72
51.69
48.54
48.10
38.84
35.84
34.99
34.06
28.84
27.32
26.95
26.77
23.64
22.49
20.68
19.46
18.90
15.75
15.68
15.39
OEt
Me
Me
O
OMe
Me
O
tBu
3
anti-55
syn-55
f1 (ppm)

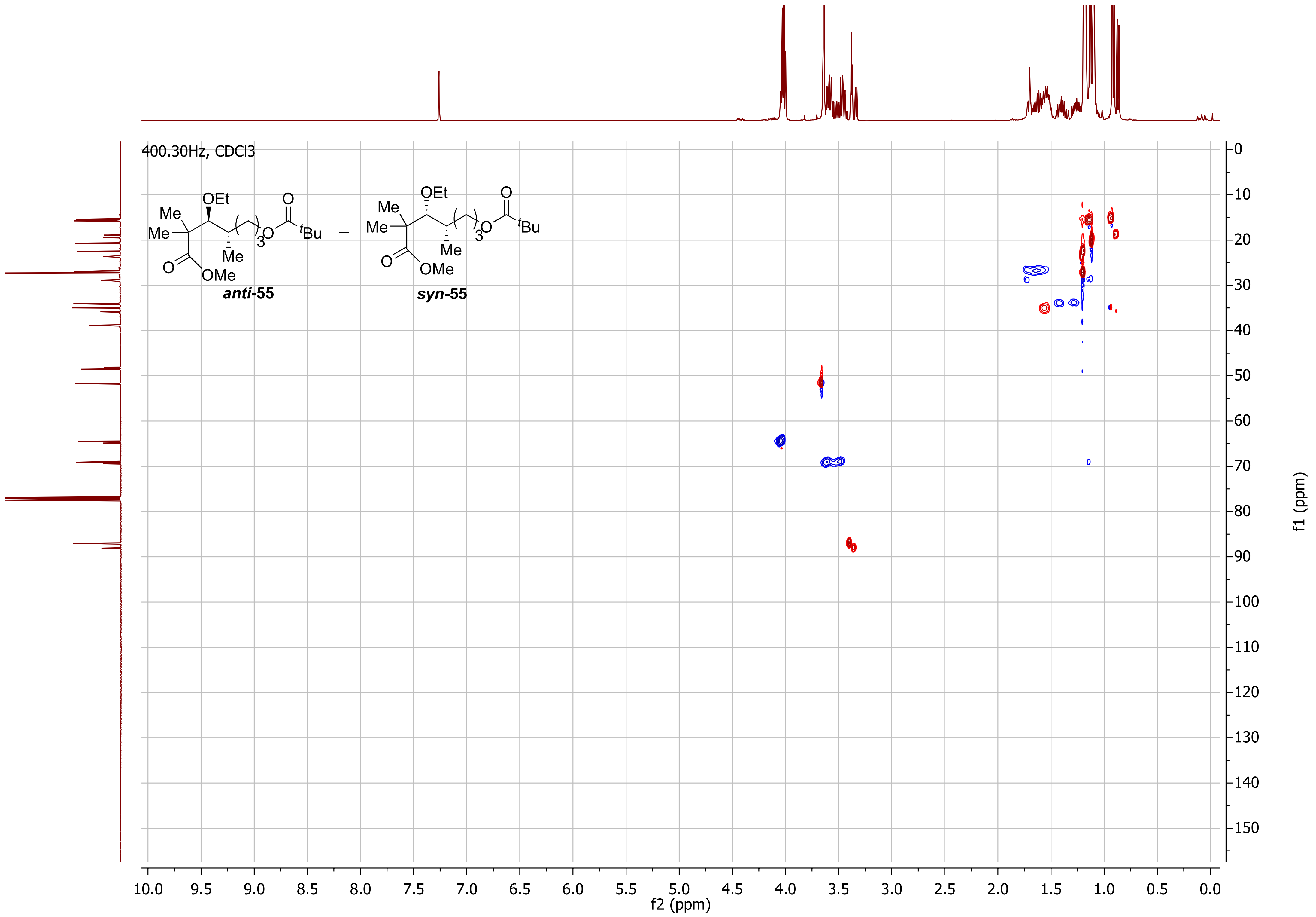

400.30Hz, CDCl3
anti-55
syn-55
f2 (ppm)
f1 (ppm)

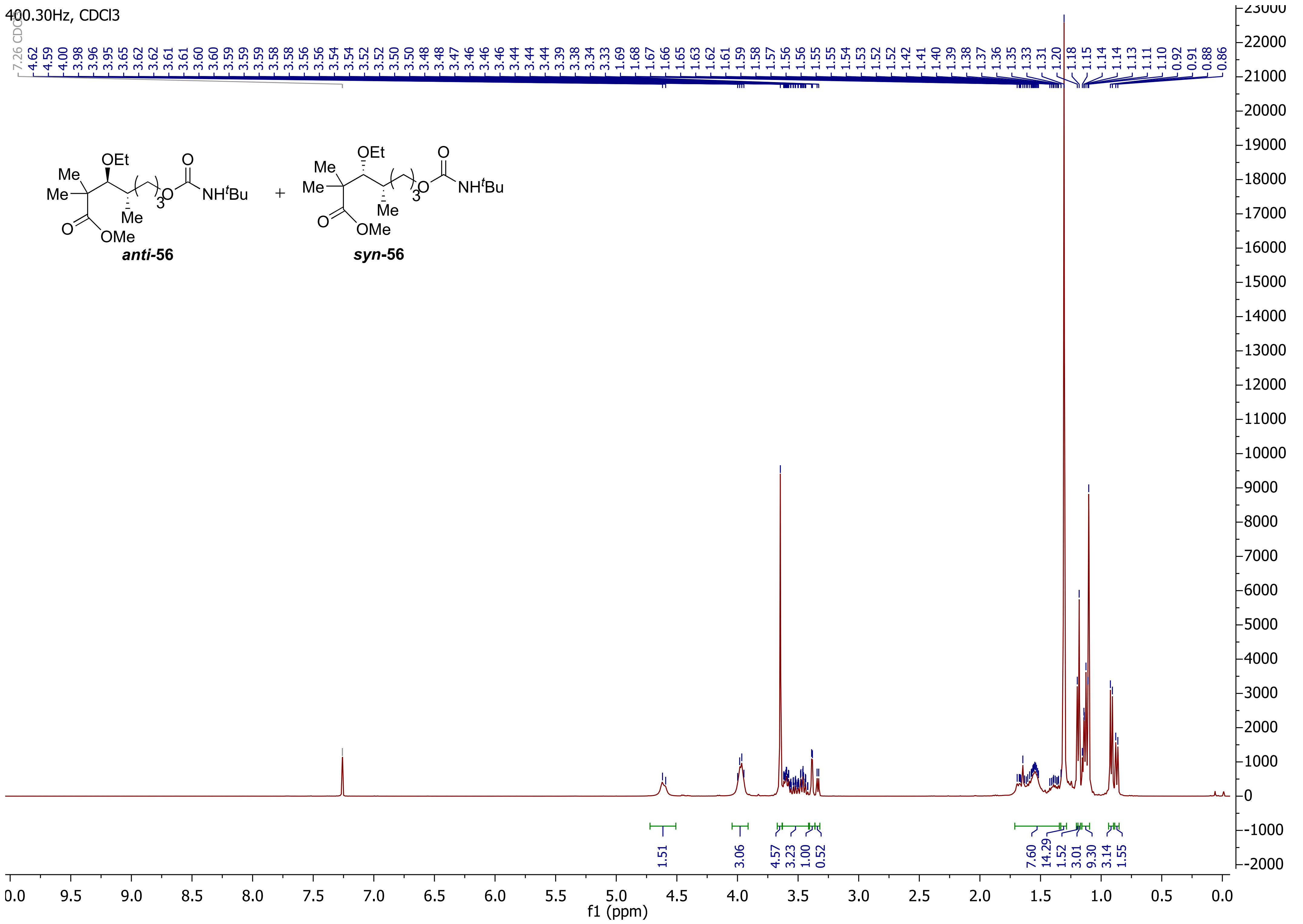
400.30Hz, CDCl3
anti-56
syn-56
f1 (ppm)

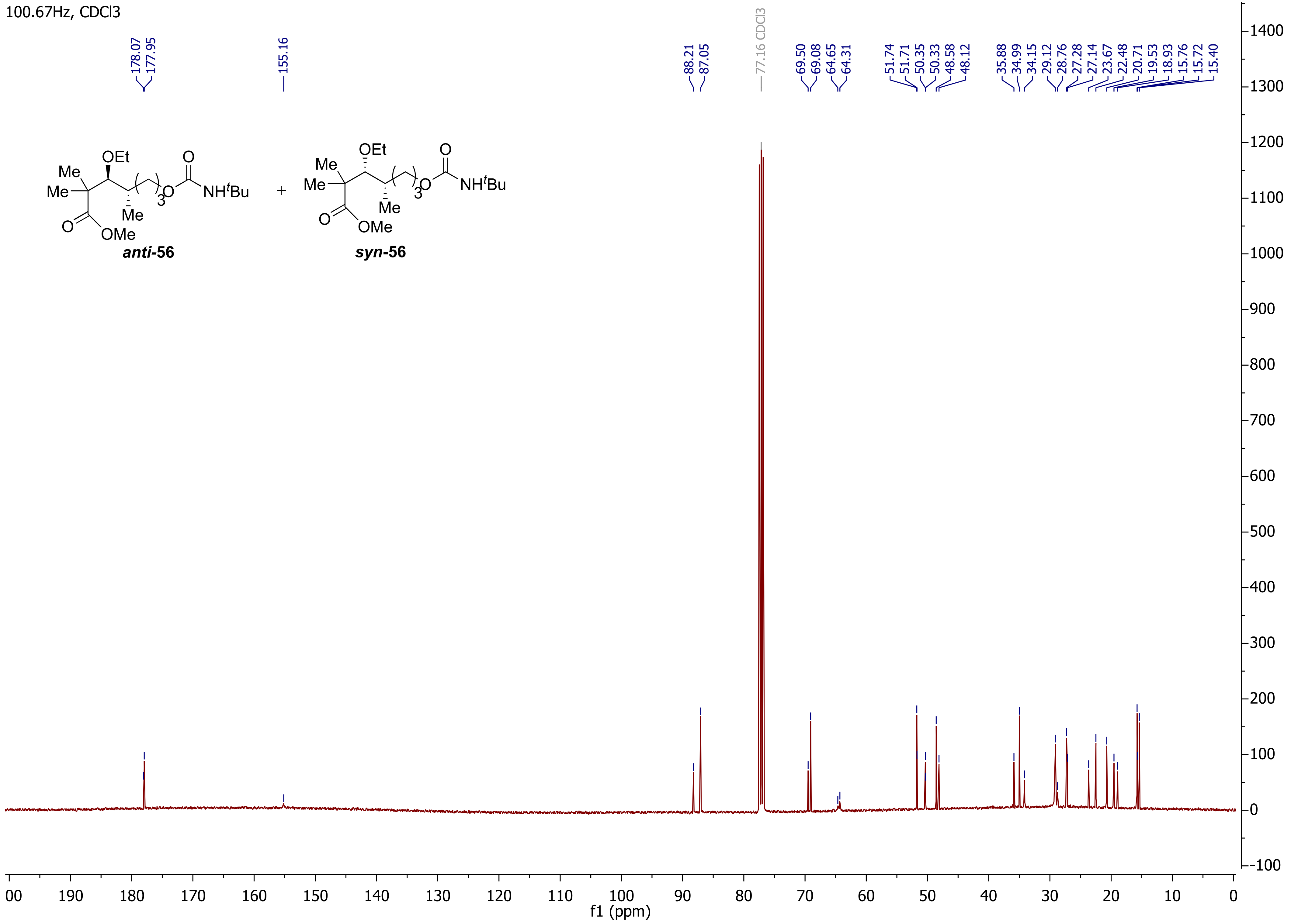
100.67Hz, CDCl3
178.07
177.95
155.16
88.21
87.05
77.16 CDCl3
69.50
69.08
64.65
64.31
51.74
51.71
50.35
50.33
48.58
48.12
35.88
34.99
34.15
29.12
28.76
27.28
27.14
23.67
22.48
20.71
19.53
18.93
15.76
15.72
15.40
anti-56
syn-56
f1 (ppm)

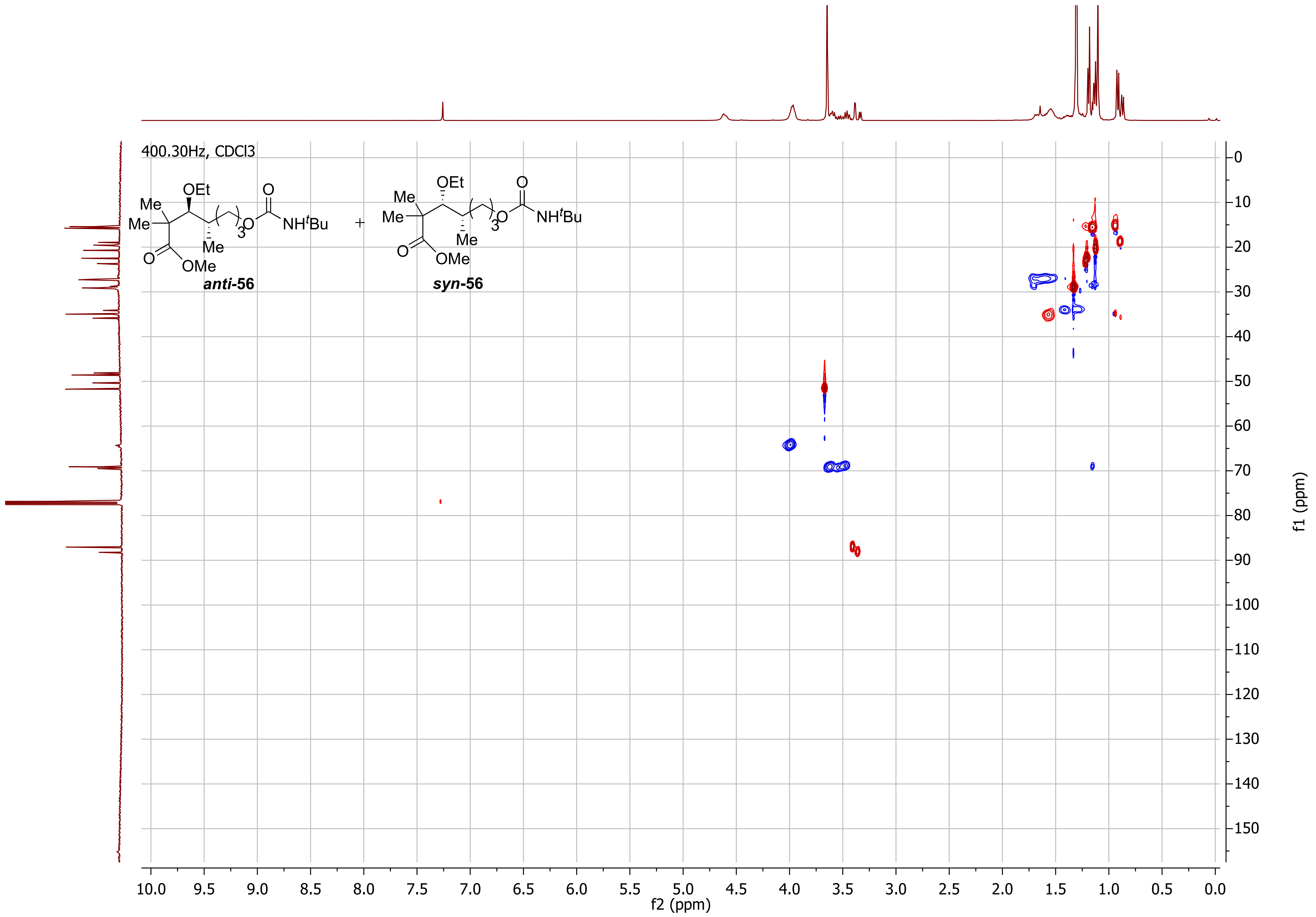

400.30Hz, CDCl3
anti-56
syn-56
f2 (ppm)
f1 (ppm)

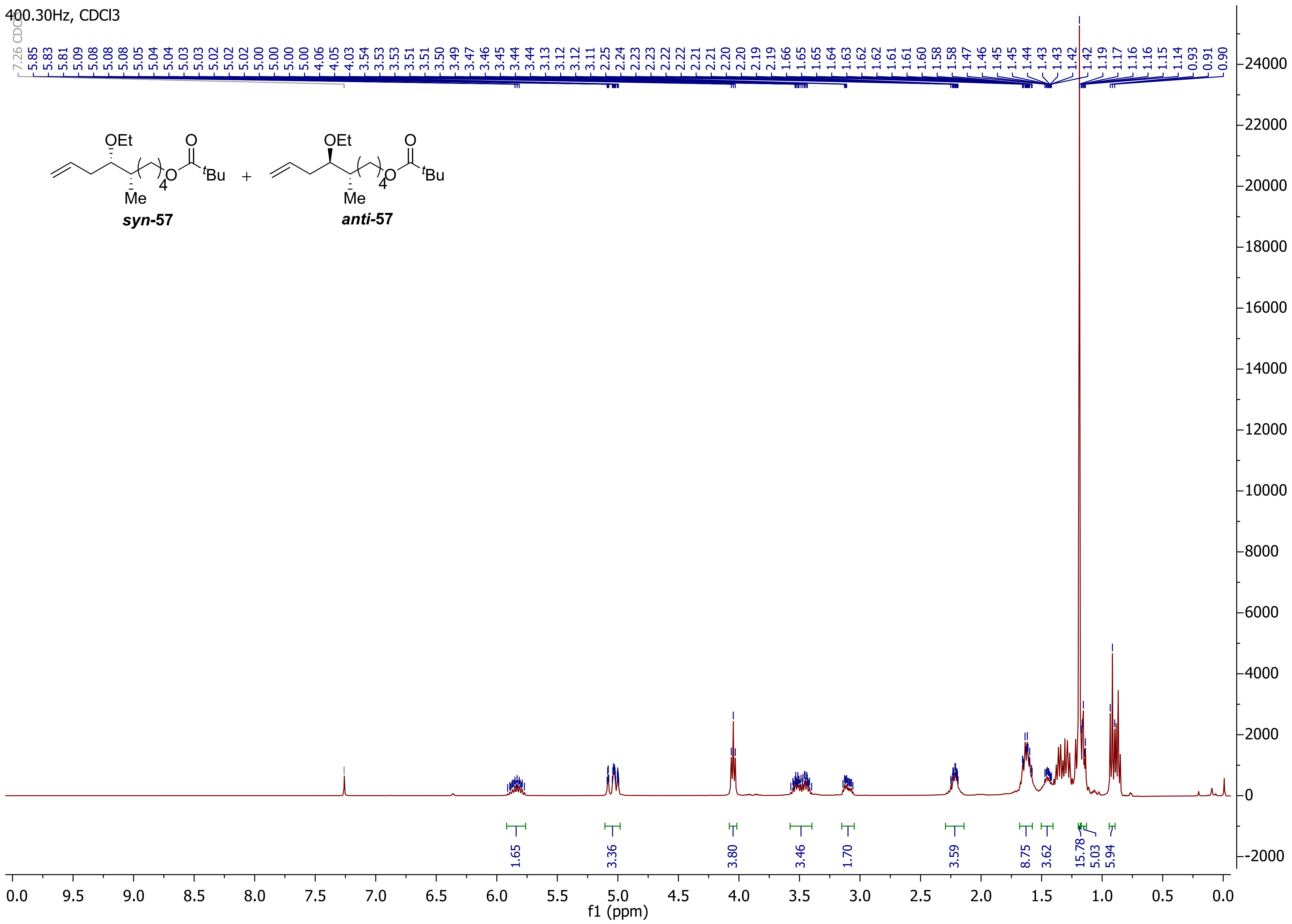
400.30Hz, CDCl3
7.26 CDCl3
5.85 5.83 5.81 5.09 5.08 5.08 5.08 5.05 5.04 5.04 5.03 5.03 5.02 5.02 5.02 5.00 5.00 5.00 5.00 4.06 4.05 4.03 3.54 3.53 3.53 3.51 3.51 3.50 3.49 3.47 3.46 3.45 3.44 3.44 3.13 3.12 3.12 3.11 2.25 2.24 2.23 2.23 2.22 2.22 2.21 2.21 2.20 2.20 2.19 2.19 1.66 1.65 1.65 1.64 1.63 1.62 1.61 1.61 1.60 1.58 1.58 1.47 1.46 1.45 1.45 1.44 1.43 1.43 1.42 1.42 1.19 1.17 1.16 1.16 1.15 1.14 0.93 0.91 0.90
OEt
O
Me
O
tBu
4
+
syn-57
anti-57
1.65
3.36
3.80
3.46
1.70
3.59
8.75
3.62
15.78
5.03
5.94
f1 (ppm)
0.0 9.5 9.0 8.5 8.0 7.5 7.0 6.5 6.0 5.5 5.0 4.5 4.0 3.5 3.0 2.5 2.0 1.5 1.0 0.5 0.0
24000 22000 20000 18000 16000 14000 12000 10000 8000 6000 4000 2000 0 -2000

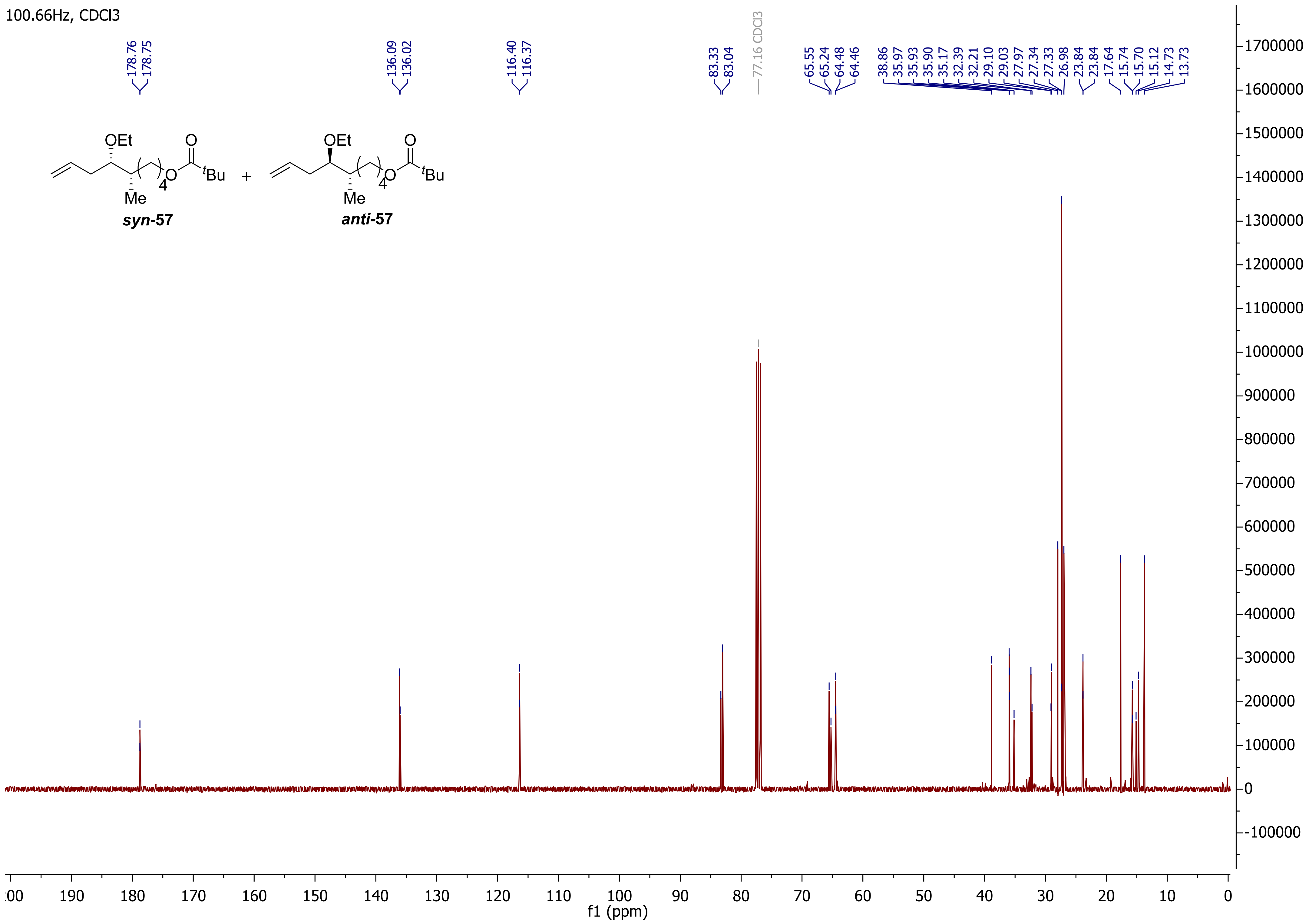

100.66Hz, CDCl3
178.76
178.75
136.09
136.02
116.40
116.37
83.33
83.04
77.16 CDCl3
65.55
65.24
64.48
64.46
38.86
35.97
35.93
35.90
35.17
32.39
32.21
29.10
29.03
27.97
27.34
27.33
26.98
23.84
23.84
17.64
15.74
15.70
15.12
14.73
13.73
OEt
O
tBu
Me
4
syn-57
anti-57
f1 (ppm)

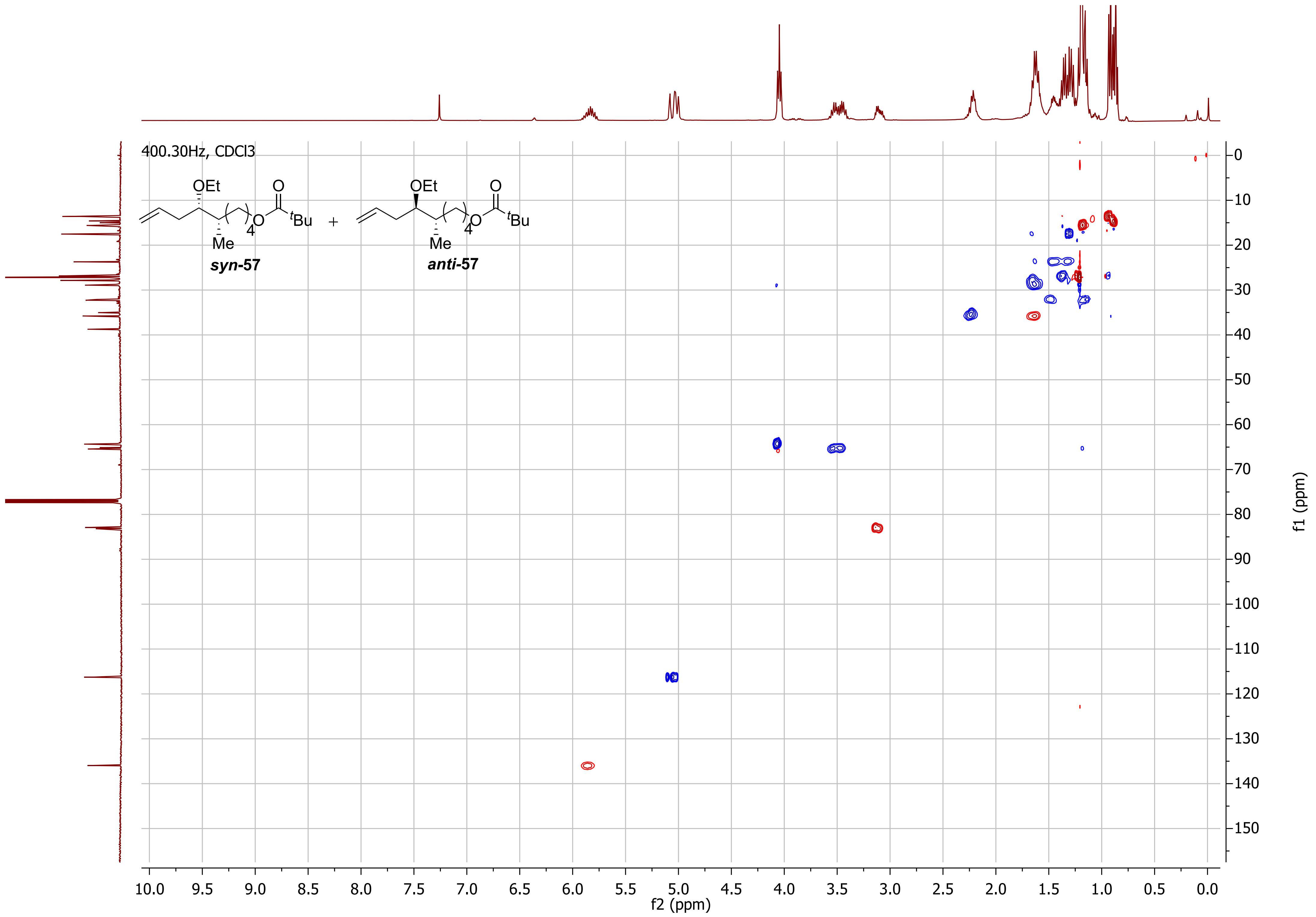

400.30Hz, CDCl3
OEt
O
O
tBu
4
Me
syn-57
+
anti-57
f2 (ppm)
f1 (ppm)

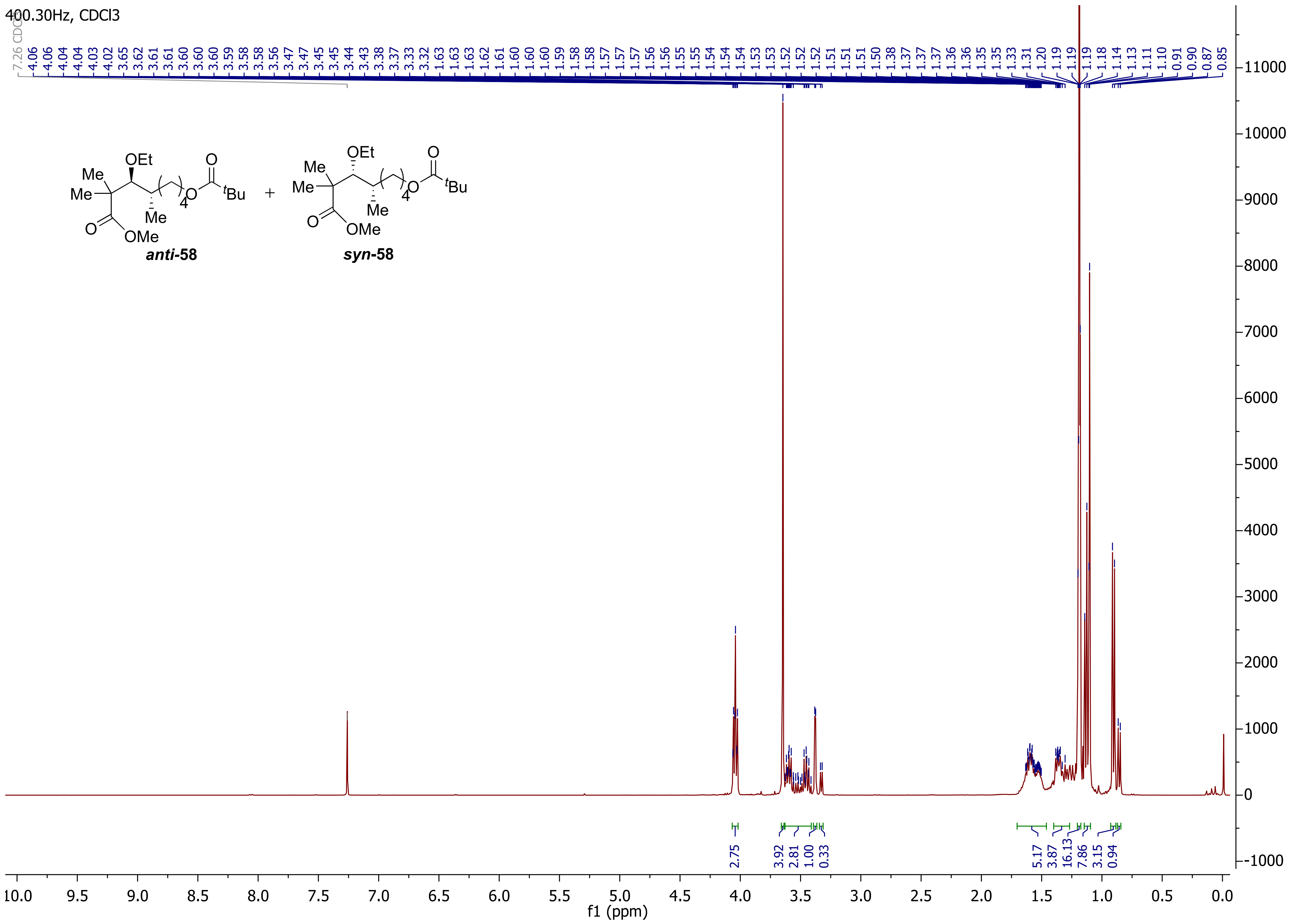

400.30Hz, CDCl3
7.26 CDCl3
anti-58
syn-58
2.75
3.92
2.81
1.00
0.33
5.17
3.87
16.13
7.86
3.15
0.94
f1 (ppm)

100.67Hz, CDCl3

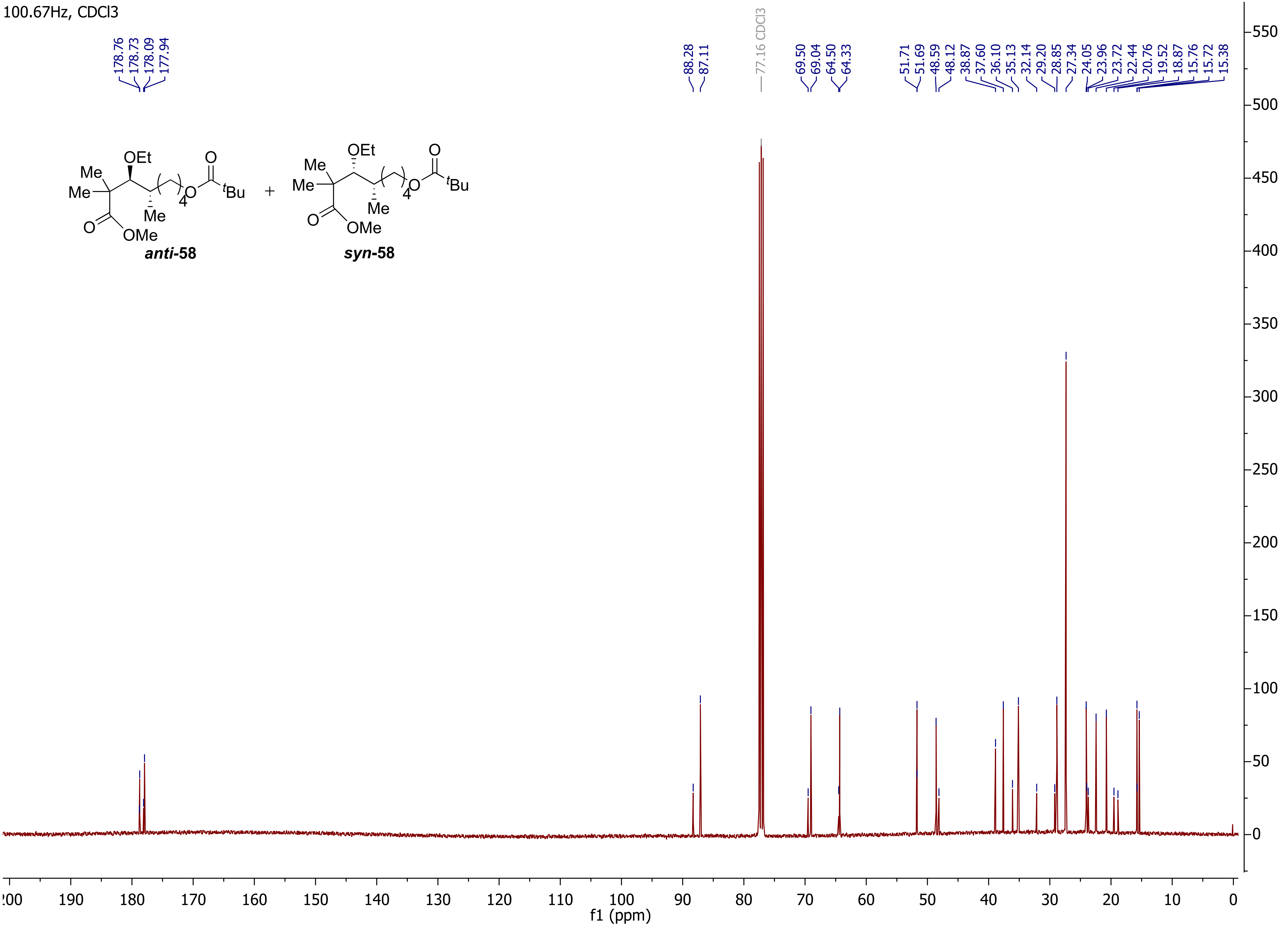

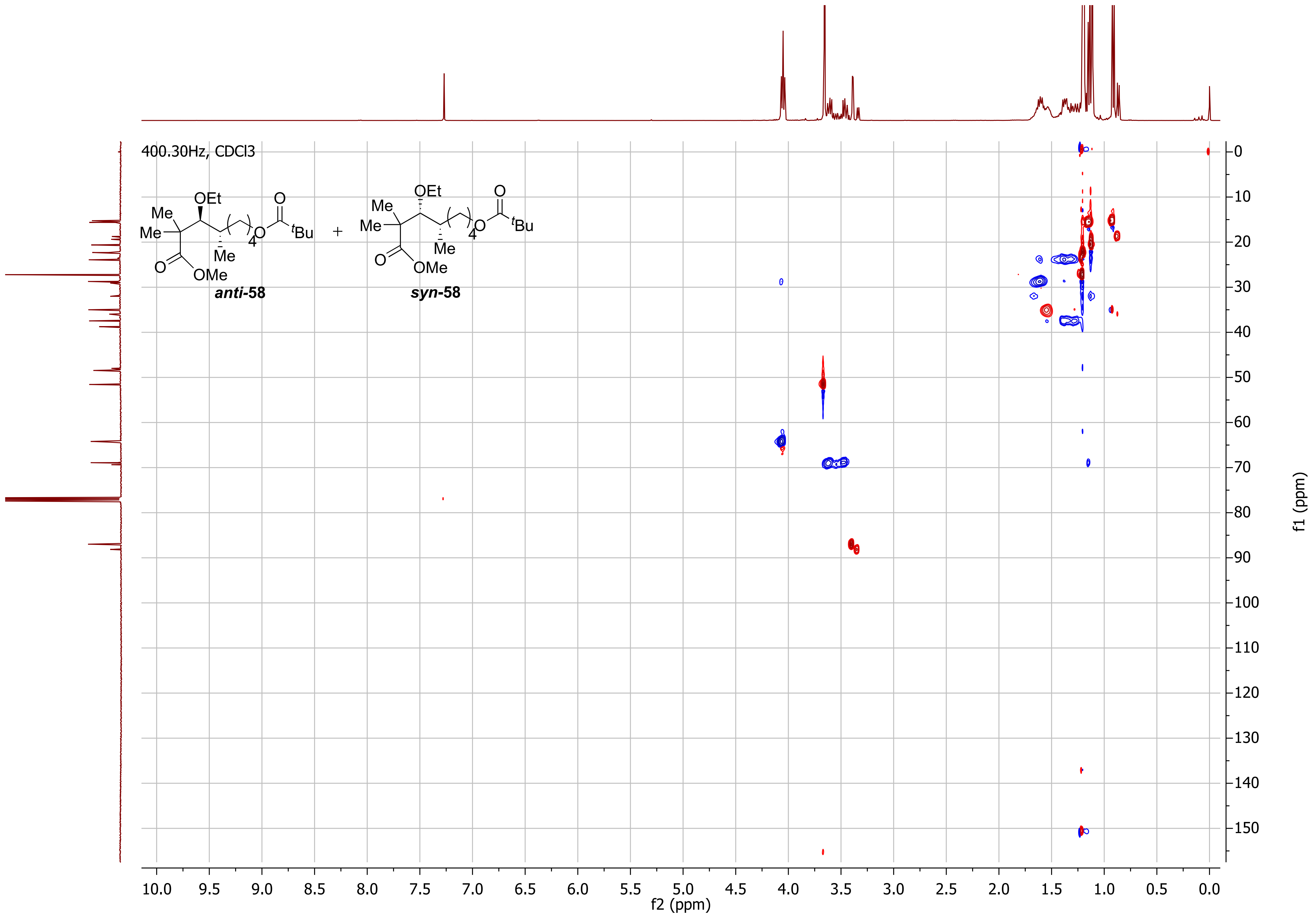
400.30Hz, CDCl3
OEt
Me
Me
O
O
tBu
Me
4
OMe
anti-58
+
OEt
Me
Me
O
O
tBu
Me
4
OMe
syn-58
f2 (ppm)
f1 (ppm)

400.30Hz, CDCl3

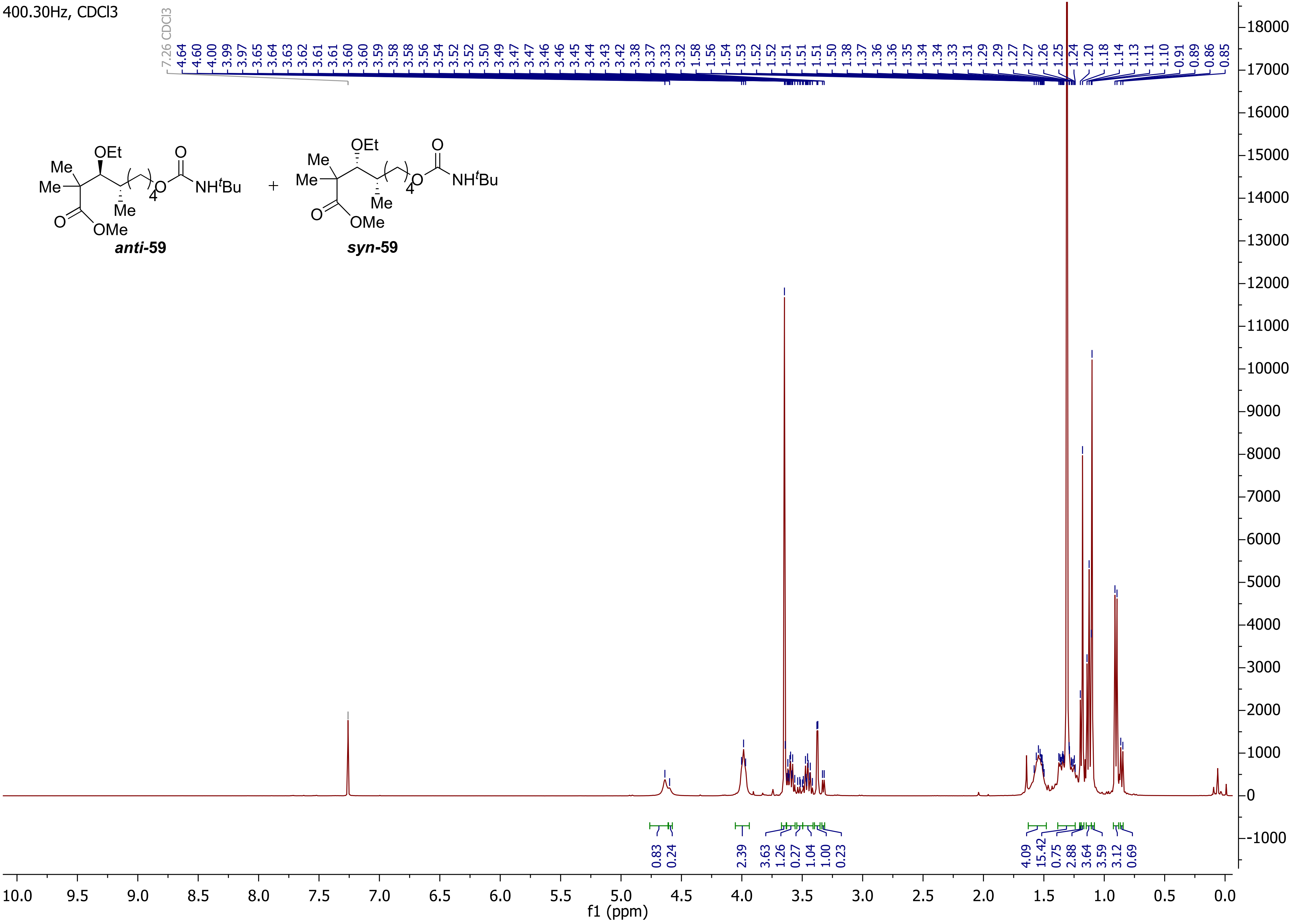

100.67Hz, CDCl3

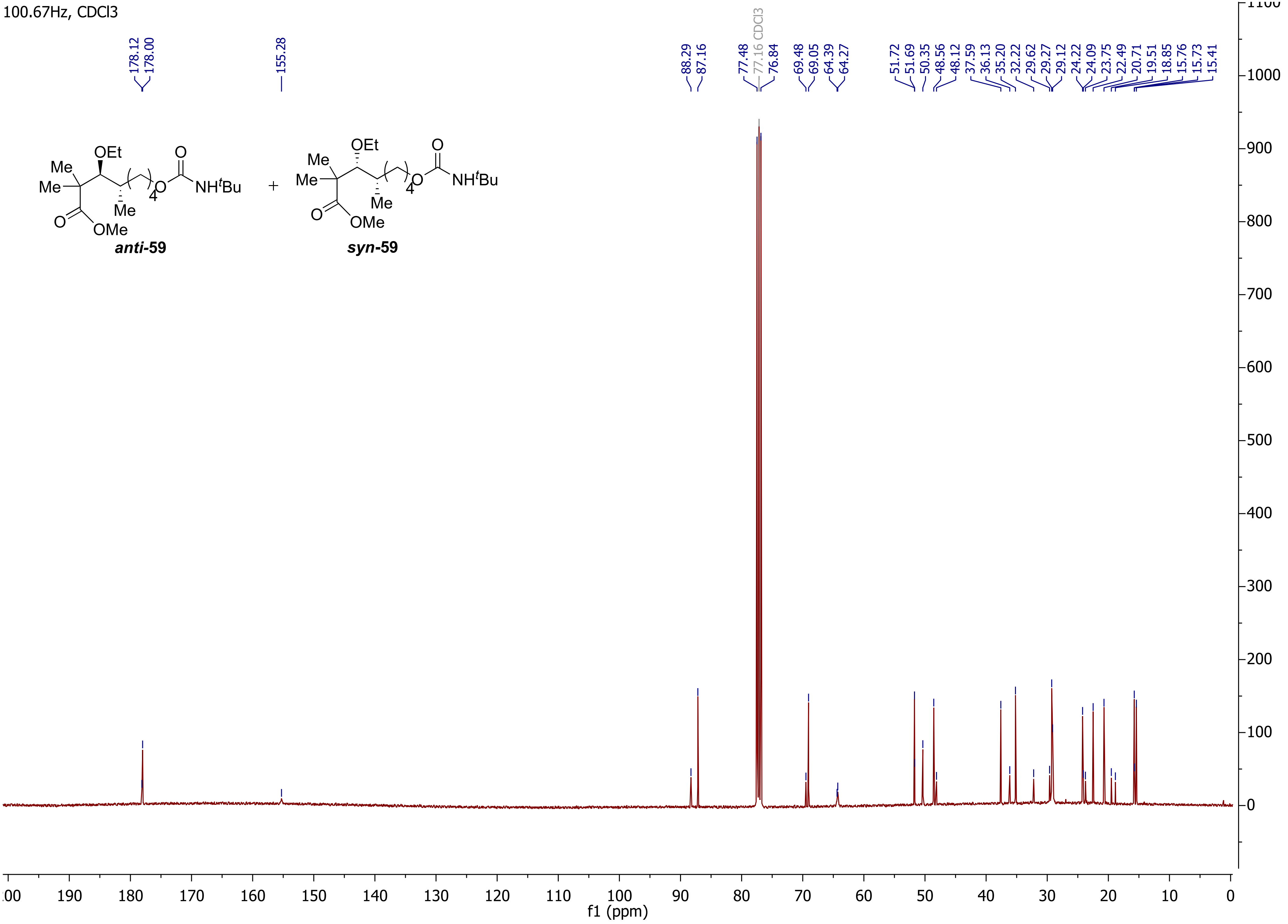

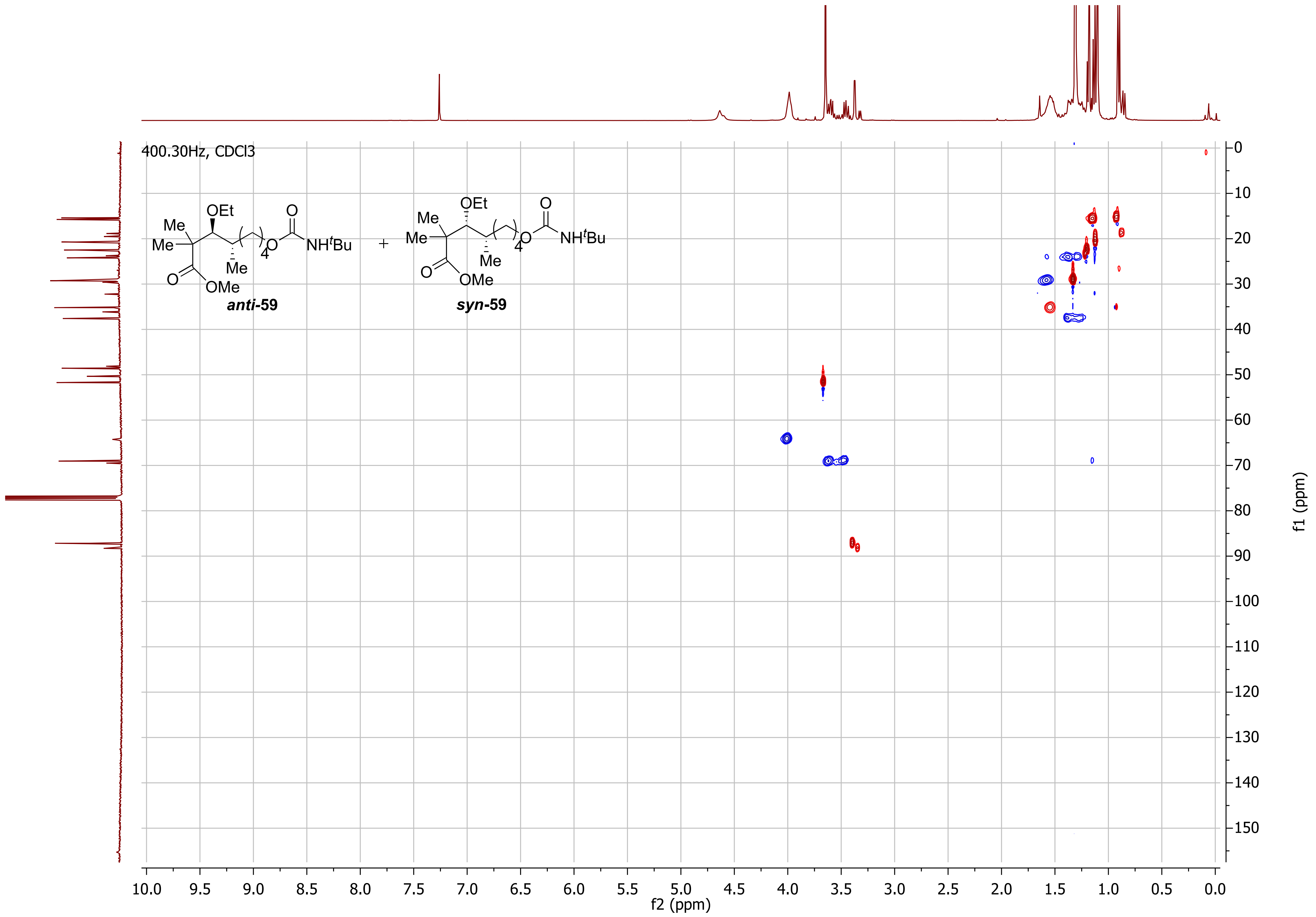
400.30Hz, CDCl3
anti-59
syn-59
f2 (ppm)
f1 (ppm)

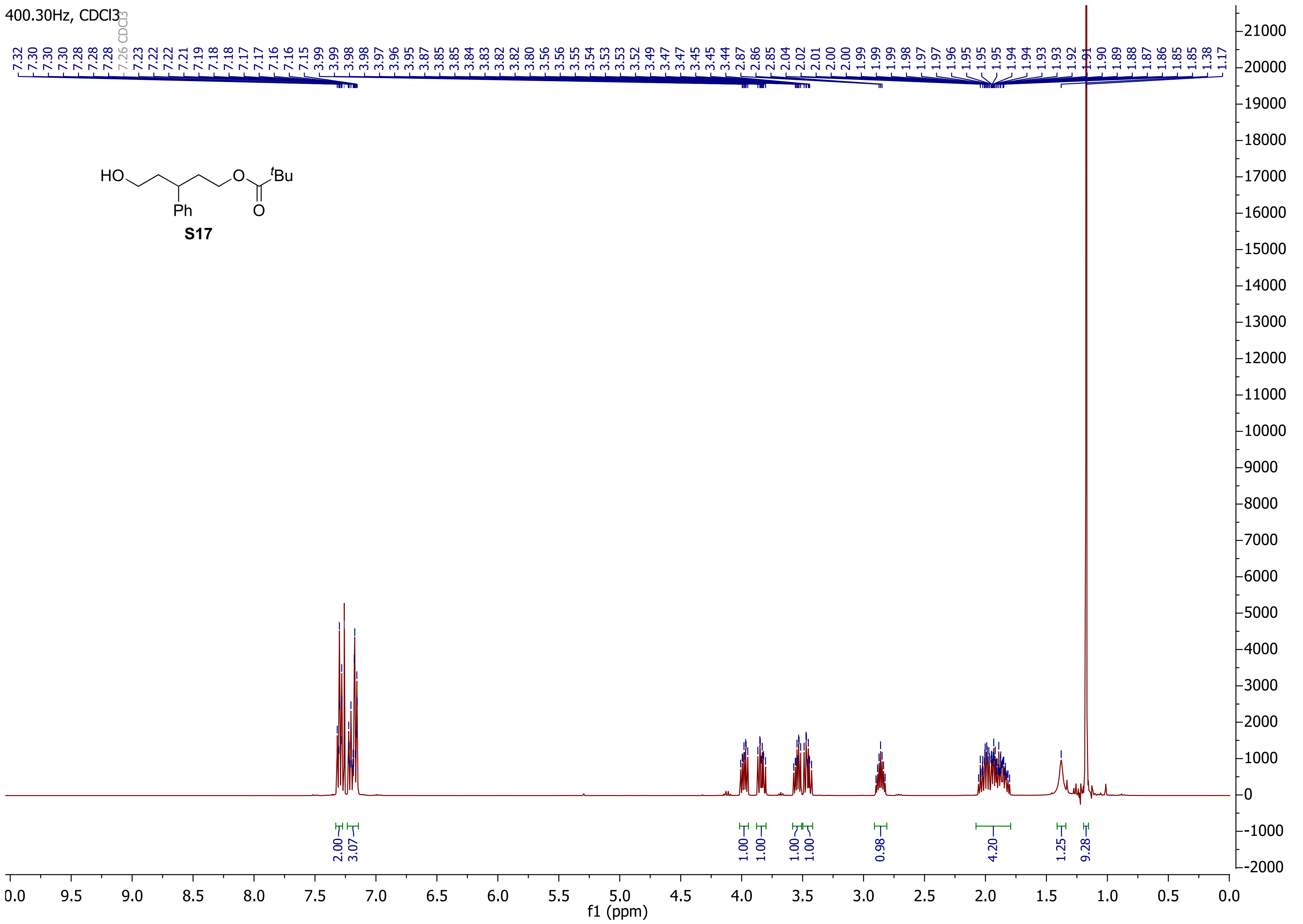
400.30Hz, CDCl3
7.32 7.30 7.30 7.30 7.28 7.28 7.28 7.26 CDCl3 7.23 7.22 7.22 7.21 7.19 7.18 7.17 7.17 7.16 7.16 7.15 3.99 3.99 3.98 3.98 3.97 3.96 3.95 3.87 3.85 3.85 3.84 3.83 3.82 3.82 3.80 3.56 3.56 3.55 3.54 3.53 3.53 3.52 3.49 3.47 3.47 3.45 3.45 3.44 2.87 2.86 2.85 2.04 2.02 2.01 2.00 2.00 1.99 1.99 1.99 1.98 1.97 1.97 1.96 1.95 1.95 1.95 1.94 1.94 1.93 1.93 1.92 1.91 1.90 1.89 1.88 1.87 1.86 1.85 1.85 1.38 1.17
HO
O
tBu
Ph
O
S17
2.00 3.07 1.00 1.00 1.00 1.00 0.98 4.20 1.25 9.28
f1 (ppm)

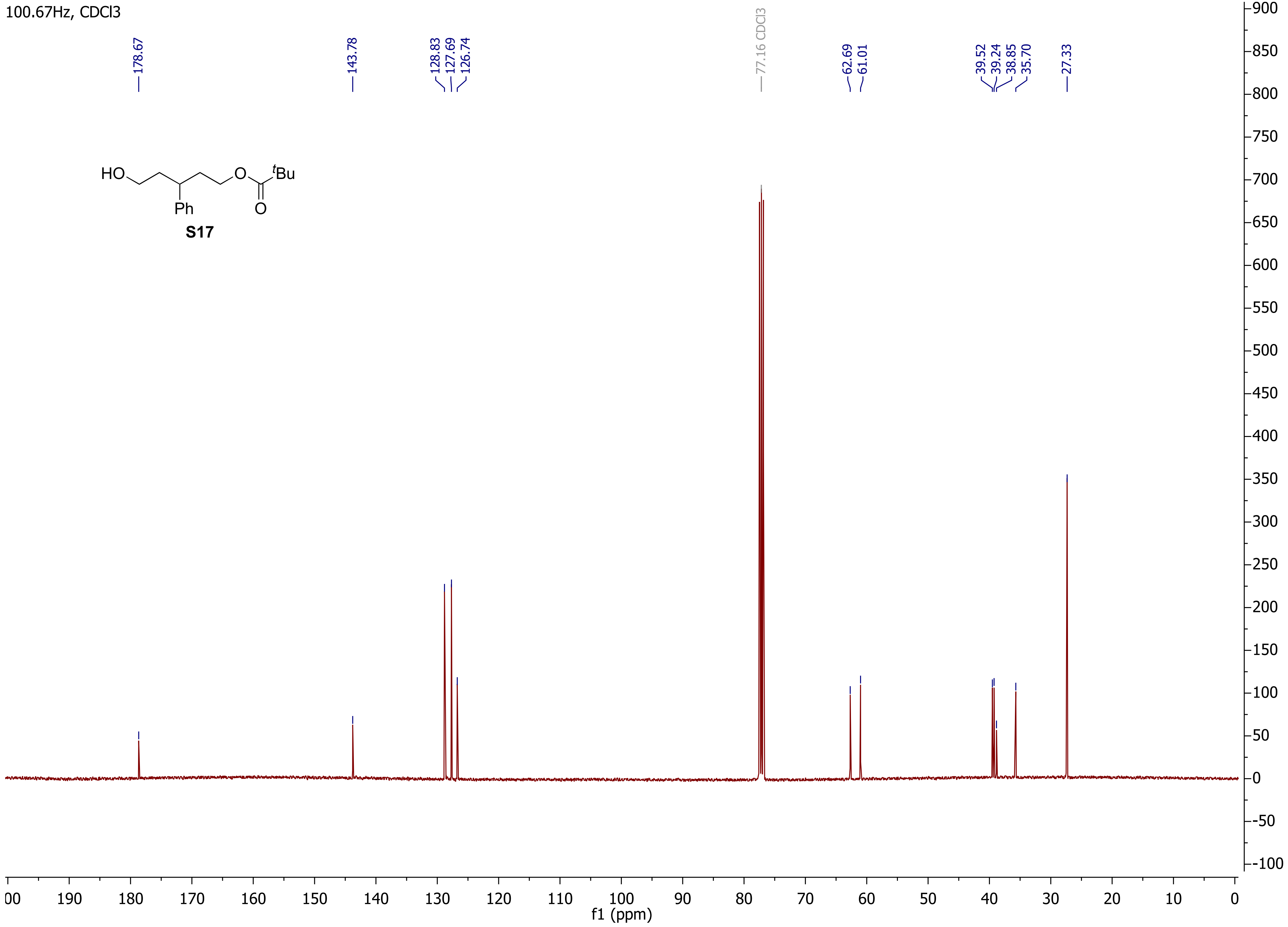
100.67Hz, CDCl3
178.67
143.78
128.83
127.69
126.74
77.16 CDCl3
62.69
61.01
39.52
39.24
38.85
35.70
27.33
HO
O
tBu
Ph
O
S17
f1 (ppm)

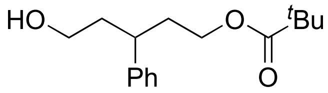

S17

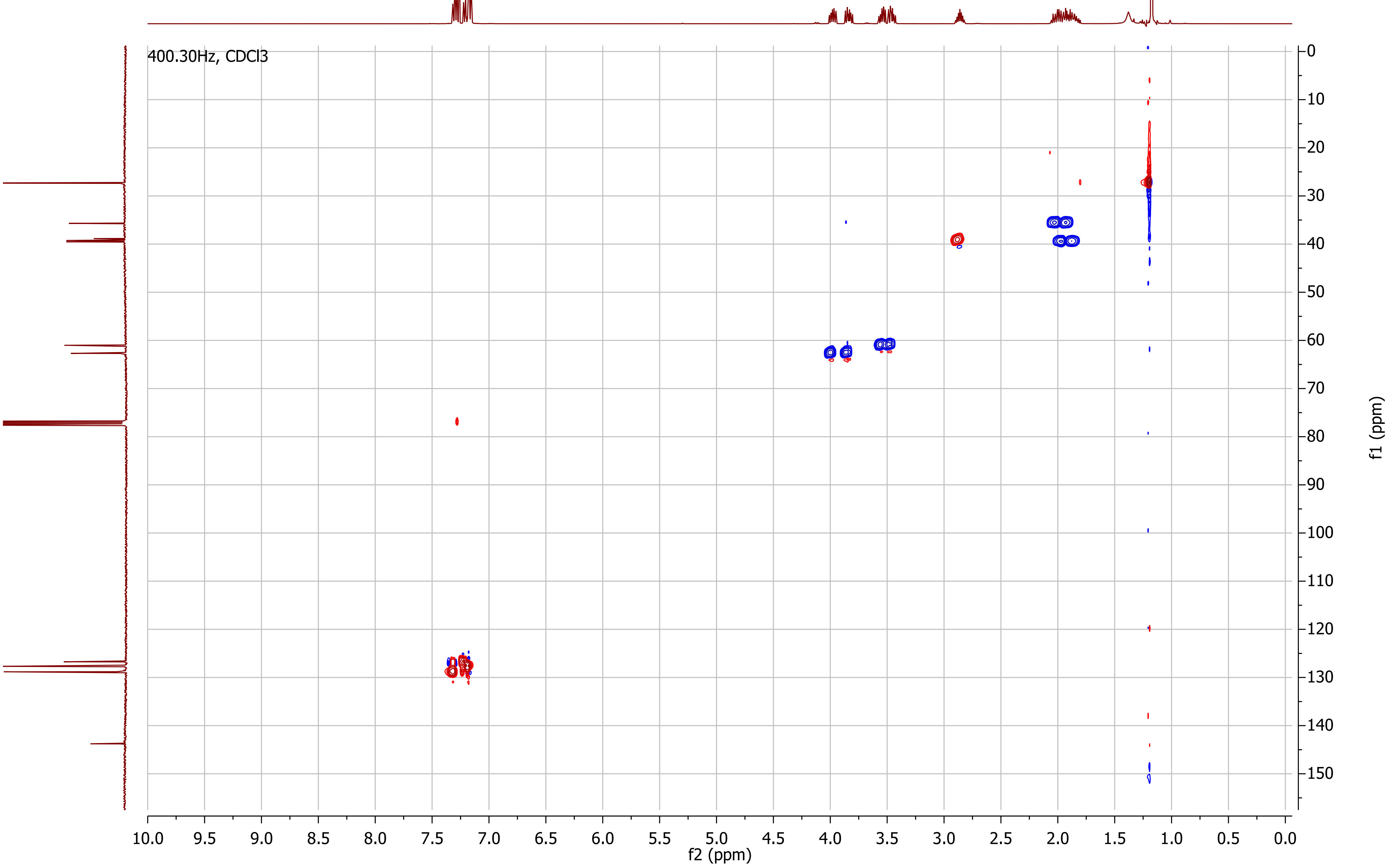

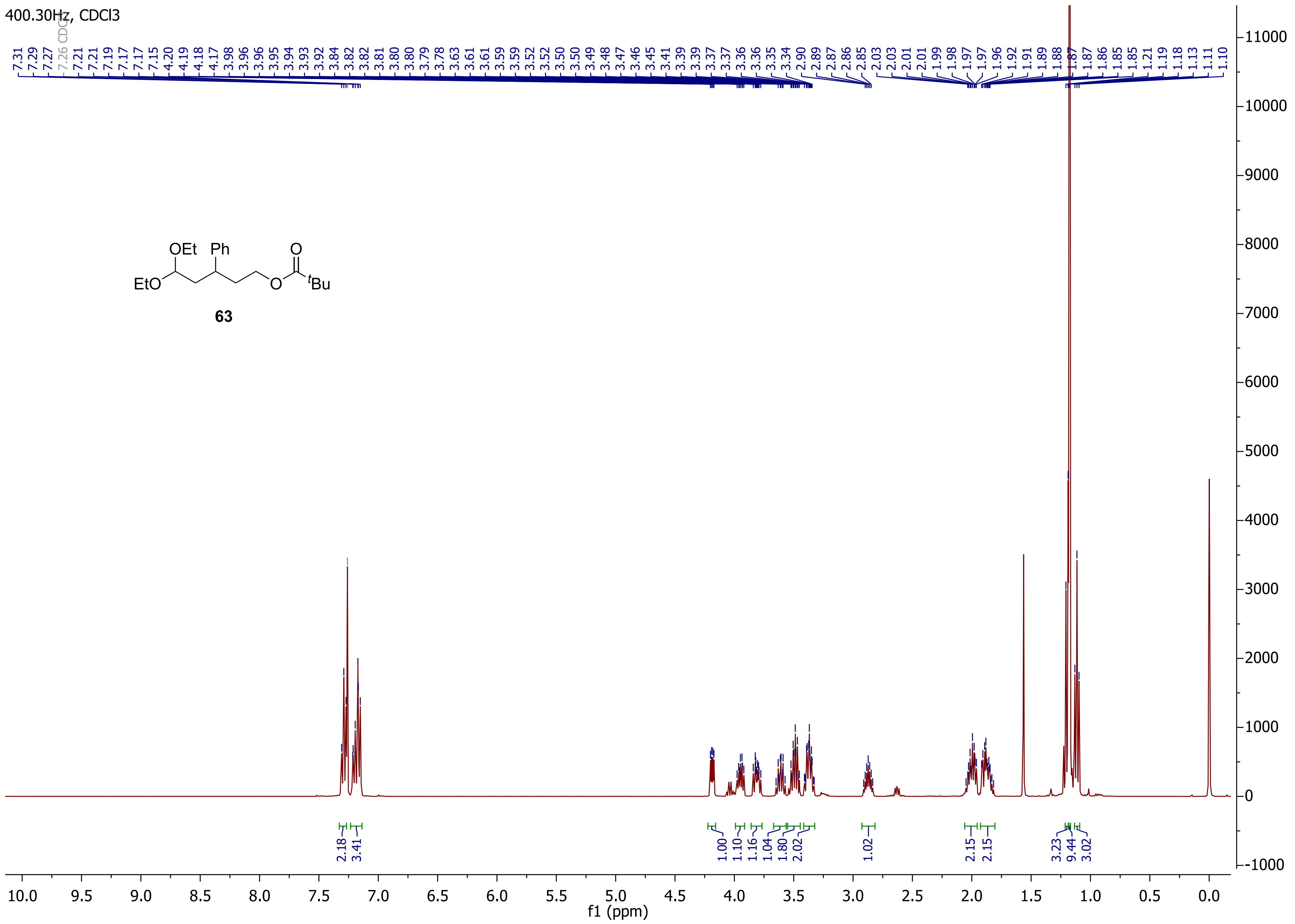
400.30Hz, CDCl3
7.31 7.29 7.27 7.26 CDCl3 7.21 7.21 7.19 7.17 7.17 7.15 4.20 4.19 4.18 4.17 3.98 3.96 3.96 3.95 3.94 3.93 3.92 3.84 3.82 3.82 3.81 3.80 3.80 3.79 3.78 3.63 3.61 3.61 3.59 3.59 3.52 3.52 3.50 3.50 3.49 3.48 3.47 3.46 3.45 3.41 3.39 3.39 3.37 3.37 3.36 3.36 3.35 3.34 2.90 2.89 2.87 2.86 2.85 2.03 2.01 2.01 1.99 1.98 1.97 1.97 1.96 1.92 1.91 1.89 1.88 1.87 1.87 1.86 1.85 1.85 1.21 1.19 1.18 1.13 1.11 1.11 1.10
OEt
Ph
O
EtO
O
tBu
63
2.18 3.41 1.00 1.10 1.16 1.04 1.80 2.02 1.02 2.15 2.15 3.23 9.44 3.02
f1 (ppm)

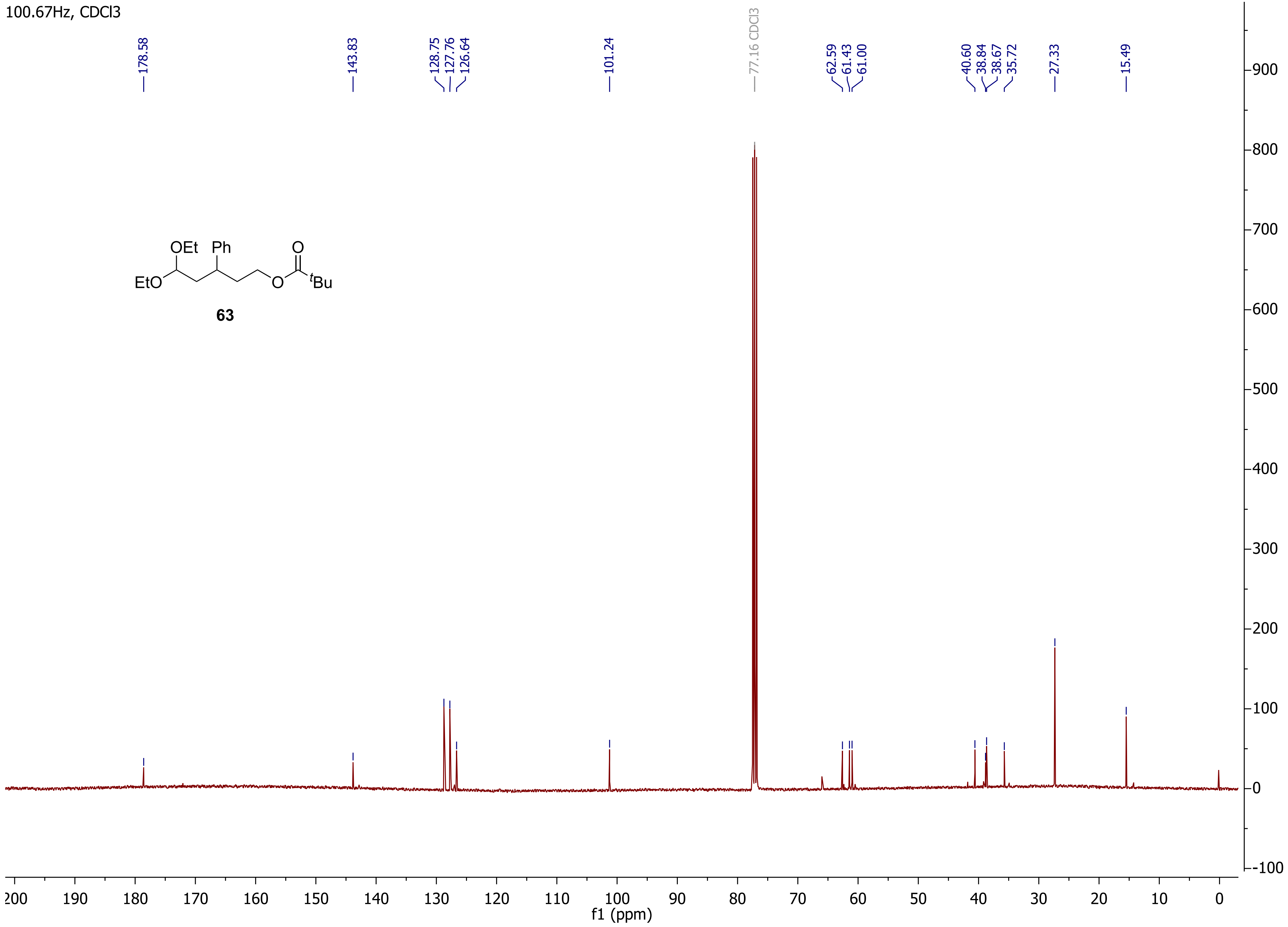

100.67Hz, CDCl3
178.58
143.83
128.75
127.76
126.64
101.24
77.16 CDCl3
62.59
61.43
61.00
40.60
38.84
38.67
35.72
27.33
15.49
OEt
Ph
O
EtO
O
tBu
63
f1 (ppm)

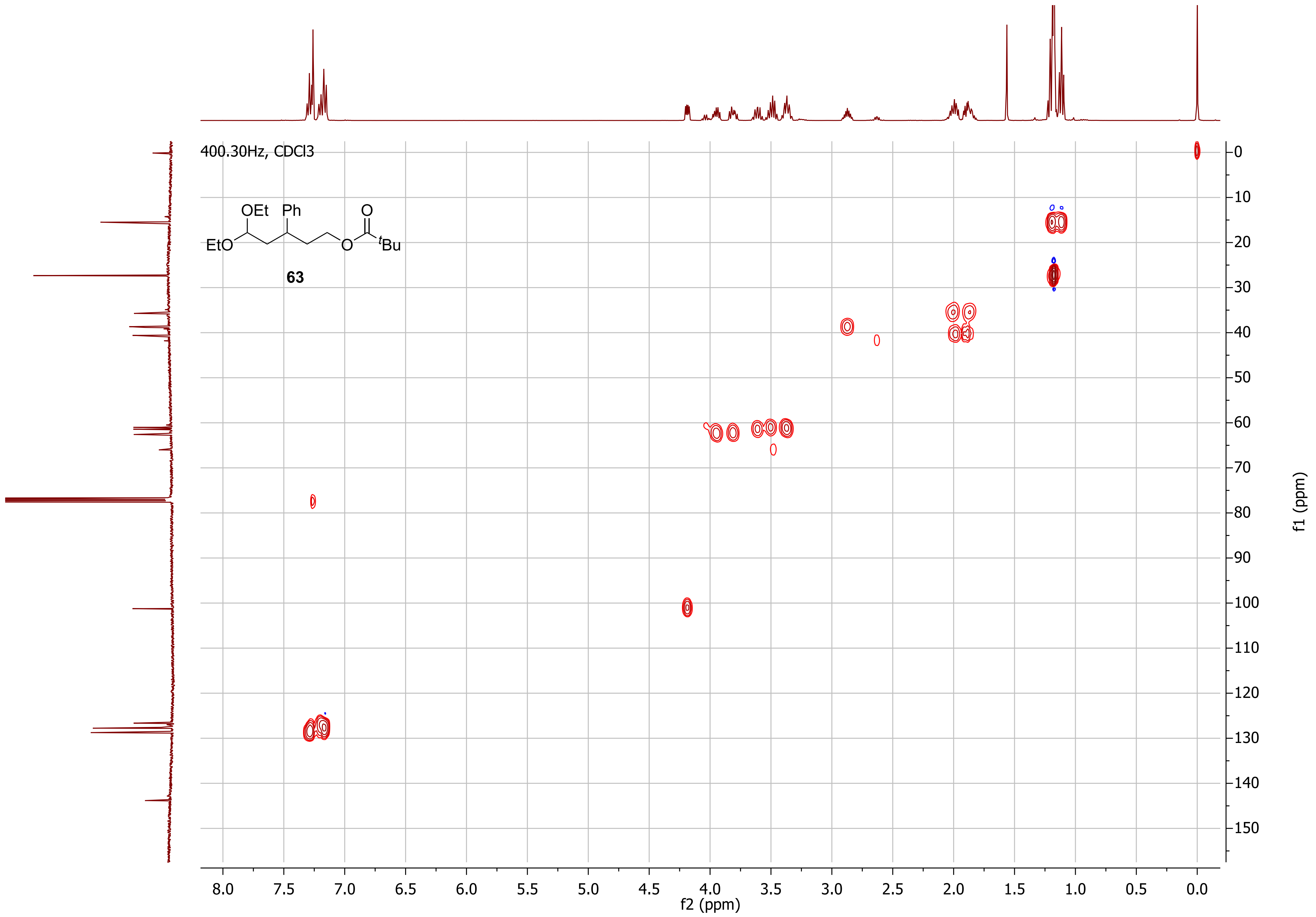

400.30Hz, CDCl3
OEt
Ph
O
EtO
O
^{t}Bu
63
f1 (ppm)
f2 (ppm)

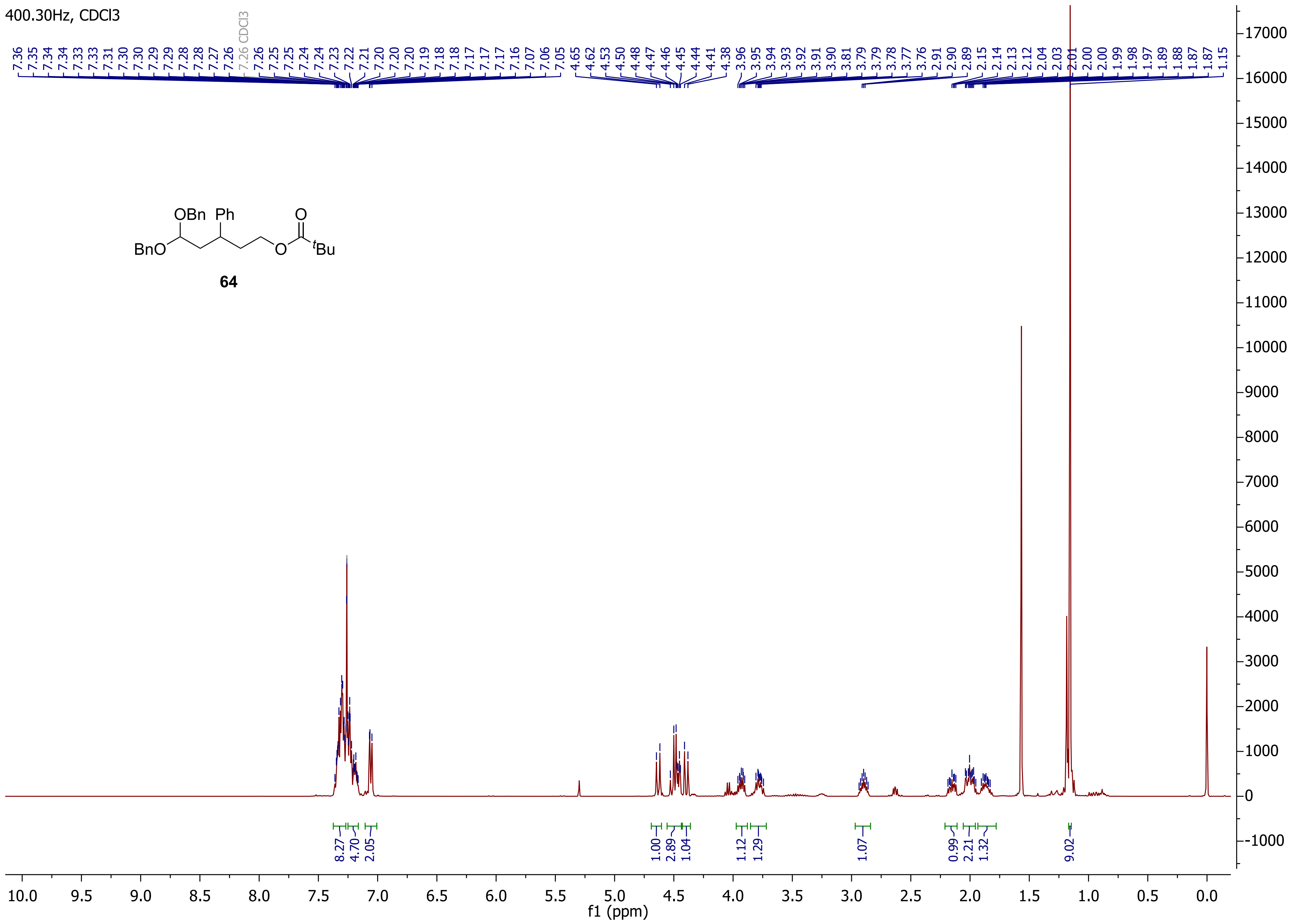

400.30Hz, CDCl3
7.36 7.35 7.34 7.34 7.33 7.33 7.31 7.30 7.30 7.29 7.29 7.28 7.28 7.27 7.26 7.26 CDCl3 7.26 7.25 7.25 7.24 7.24 7.23 7.22 7.21 7.21 7.20 7.20 7.19 7.18 7.18 7.17 7.17 7.17 7.16 7.07 7.06 7.05 4.65 4.62 4.53 4.50 4.48 4.47 4.46 4.45 4.44 4.41 4.38 3.96 3.95 3.94 3.93 3.92 3.91 3.90 3.81 3.79 3.79 3.78 3.77 3.76 2.91 2.90 2.89 2.15 2.14 2.13 2.12 2.04 2.03 2.01 2.00 2.00 1.99 1.98 1.97 1.89 1.88 1.87 1.87 1.15
OBn
Ph
O
BnO
O
tBu
64
8.27 4.70 2.05 1.00 2.89 1.04 1.12 1.29 1.07 0.99 2.21 1.32 9.02
f1 (ppm)

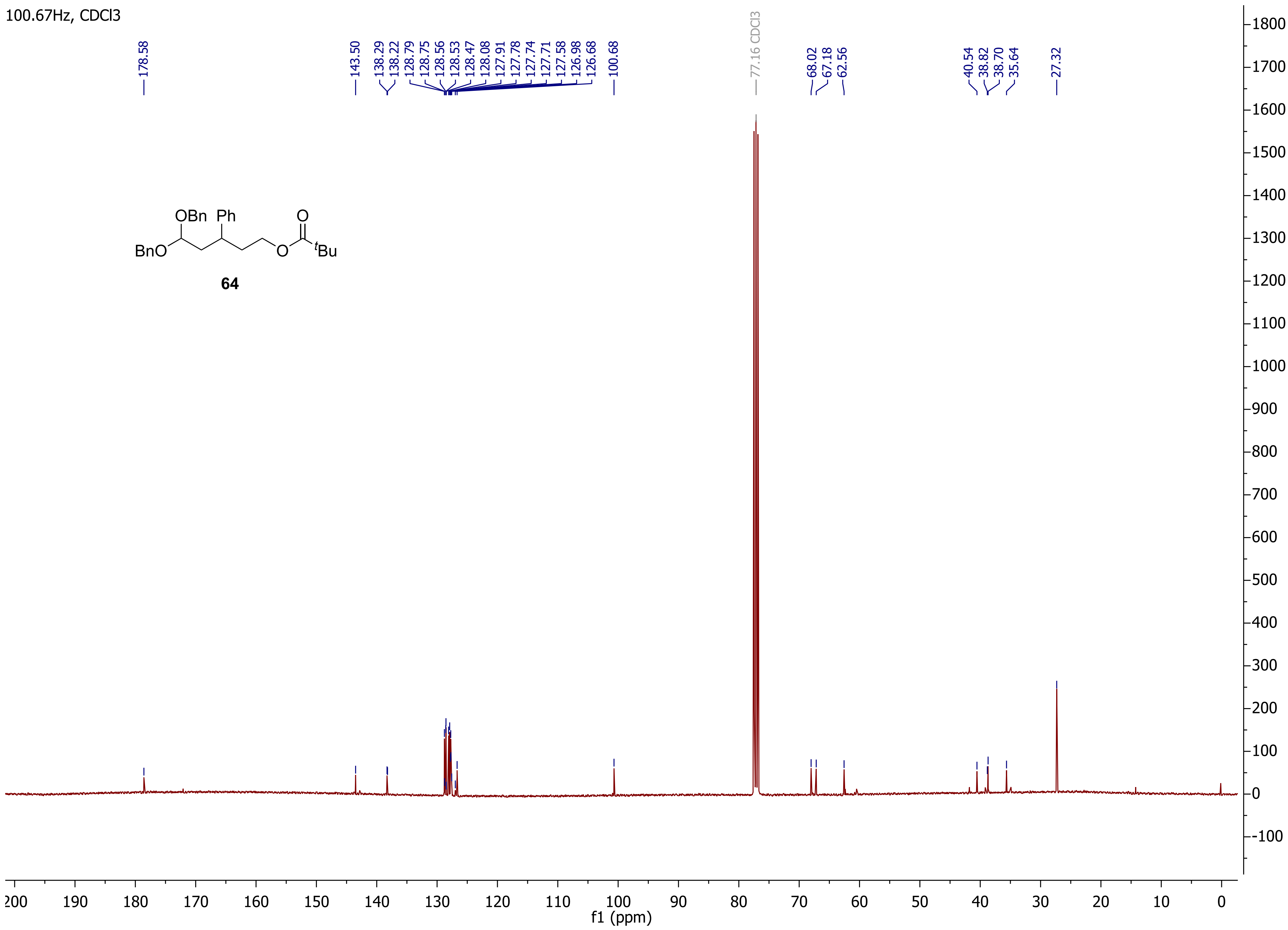
100.67Hz, CDCl3
178.58
143.50
138.29
138.22
128.79
128.75
128.56
128.53
128.47
128.08
127.91
127.78
127.74
127.71
127.58
126.98
126.68
100.68
77.16 CDCl3
68.02
67.18
62.56
40.54
38.82
38.70
35.64
27.32
OBn
Ph
O
BnO
O
tBu
64
f1 (ppm)

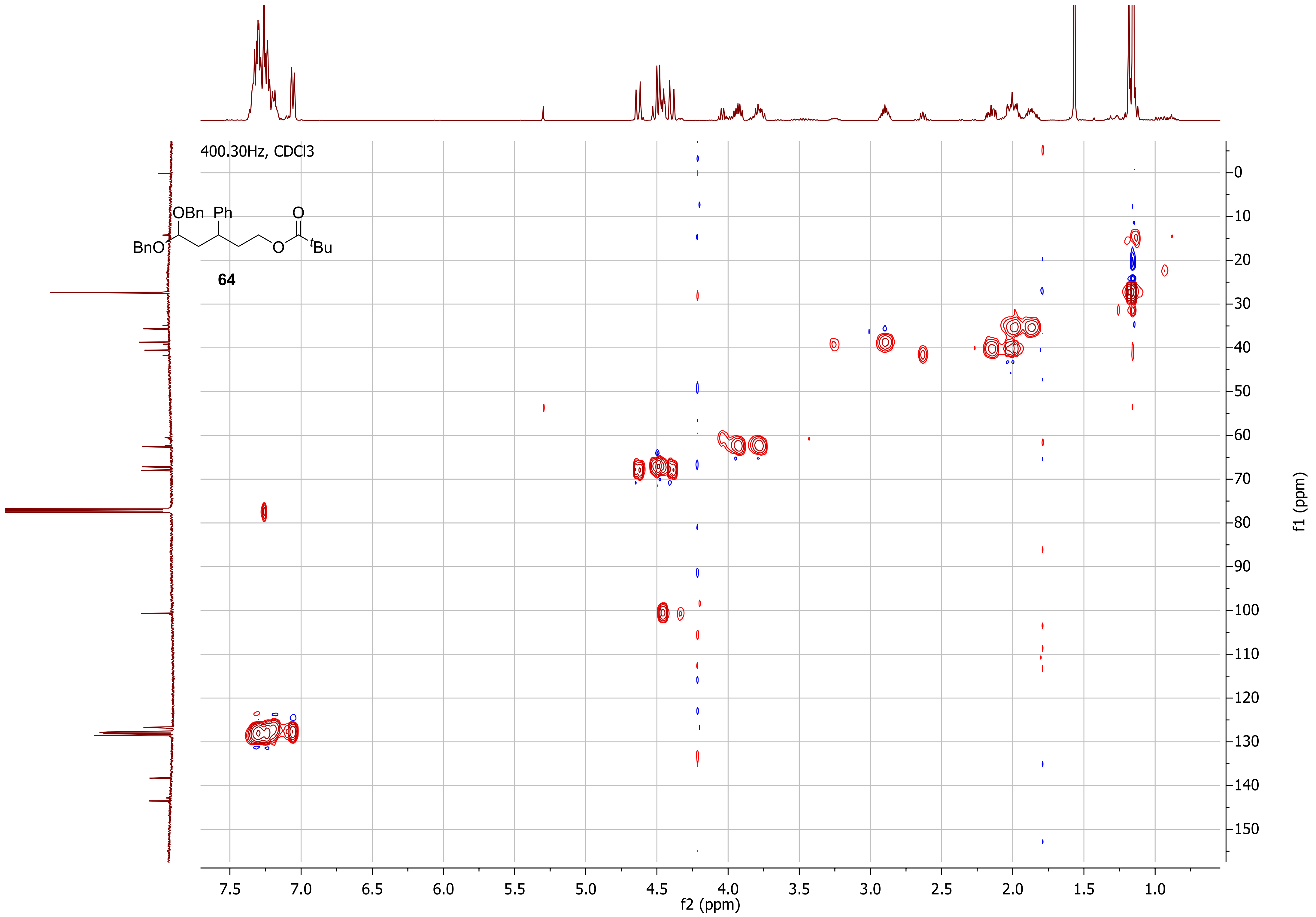
400.30Hz, CDCl3
OBn Ph O
BnO O tBu
64
f2 (ppm)
f1 (ppm)

400.30Hz, CDCl3

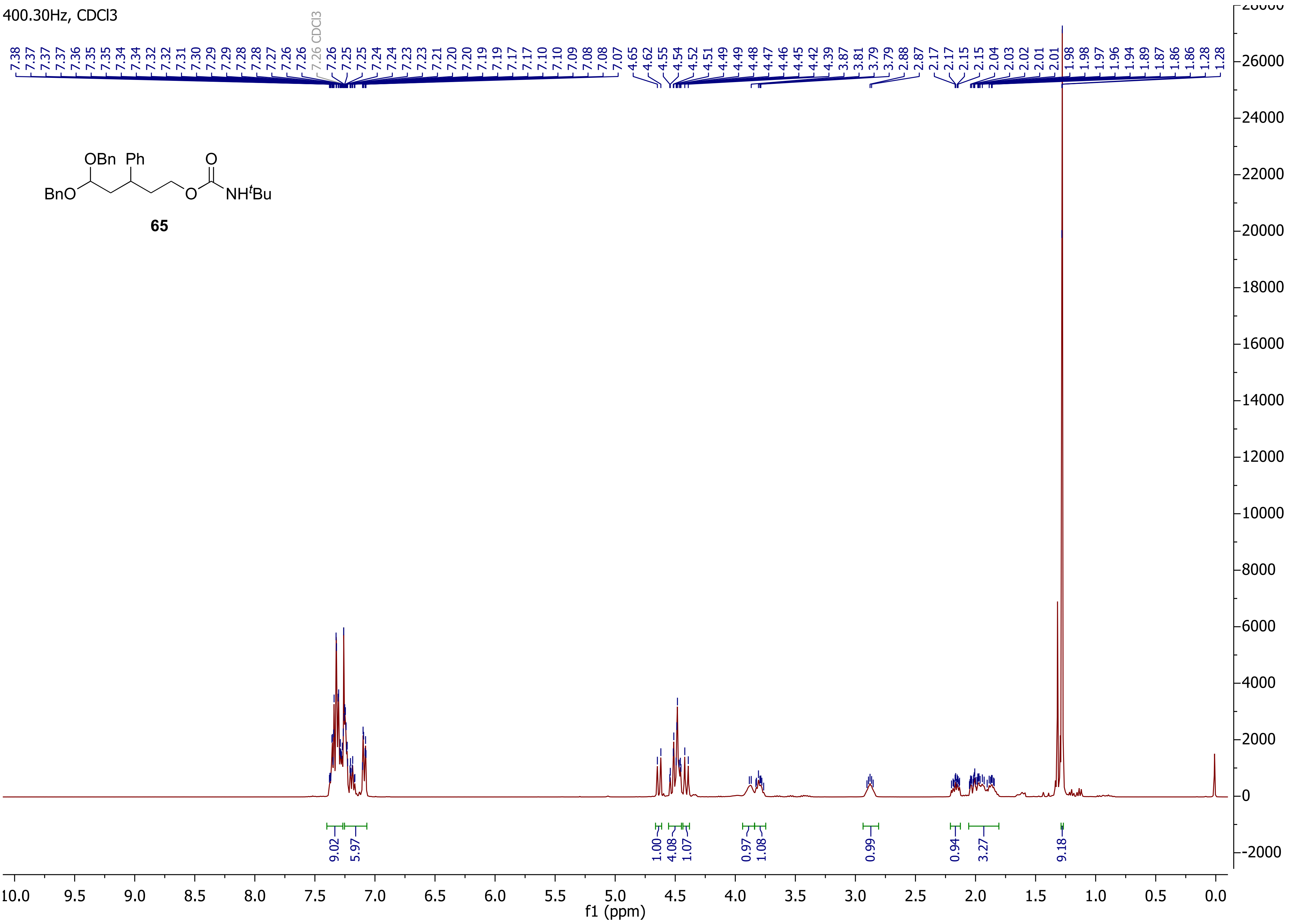

65

100.67Hz, CDCl3

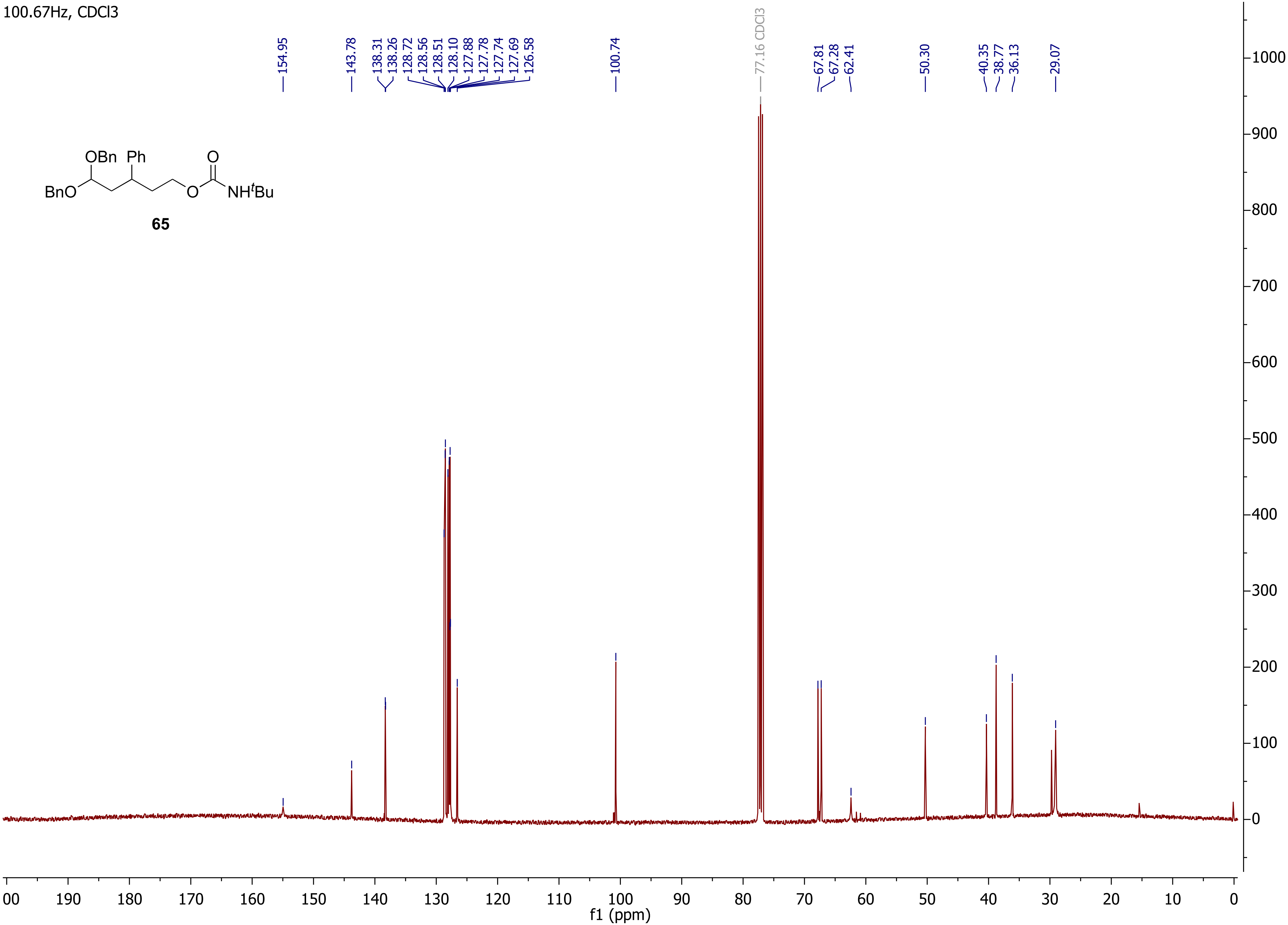

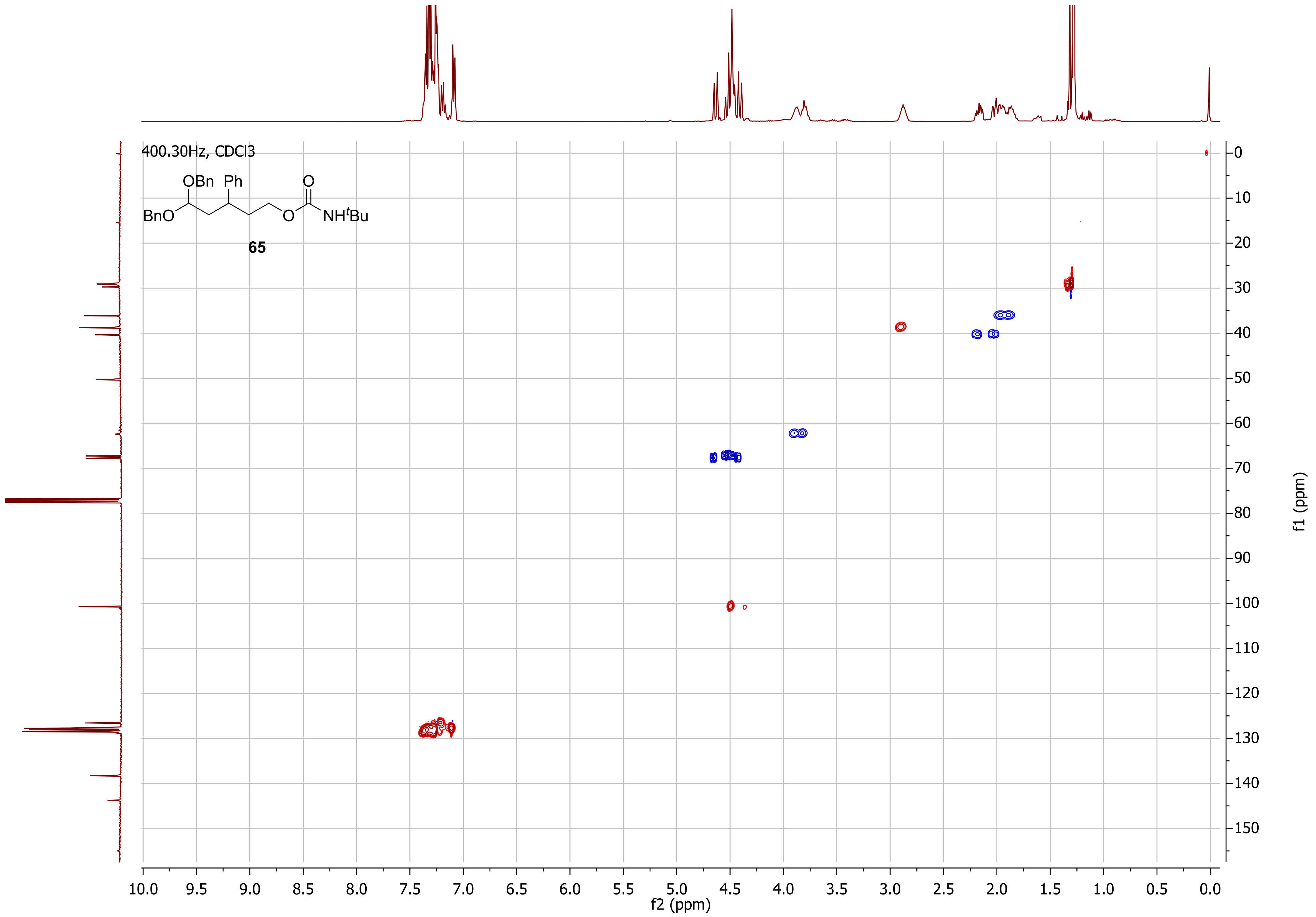

400.30Hz, CDCl3
OBn
Ph
O
BnO
O
NH^{t}Bu
65
f2 (ppm)
f1 (ppm)

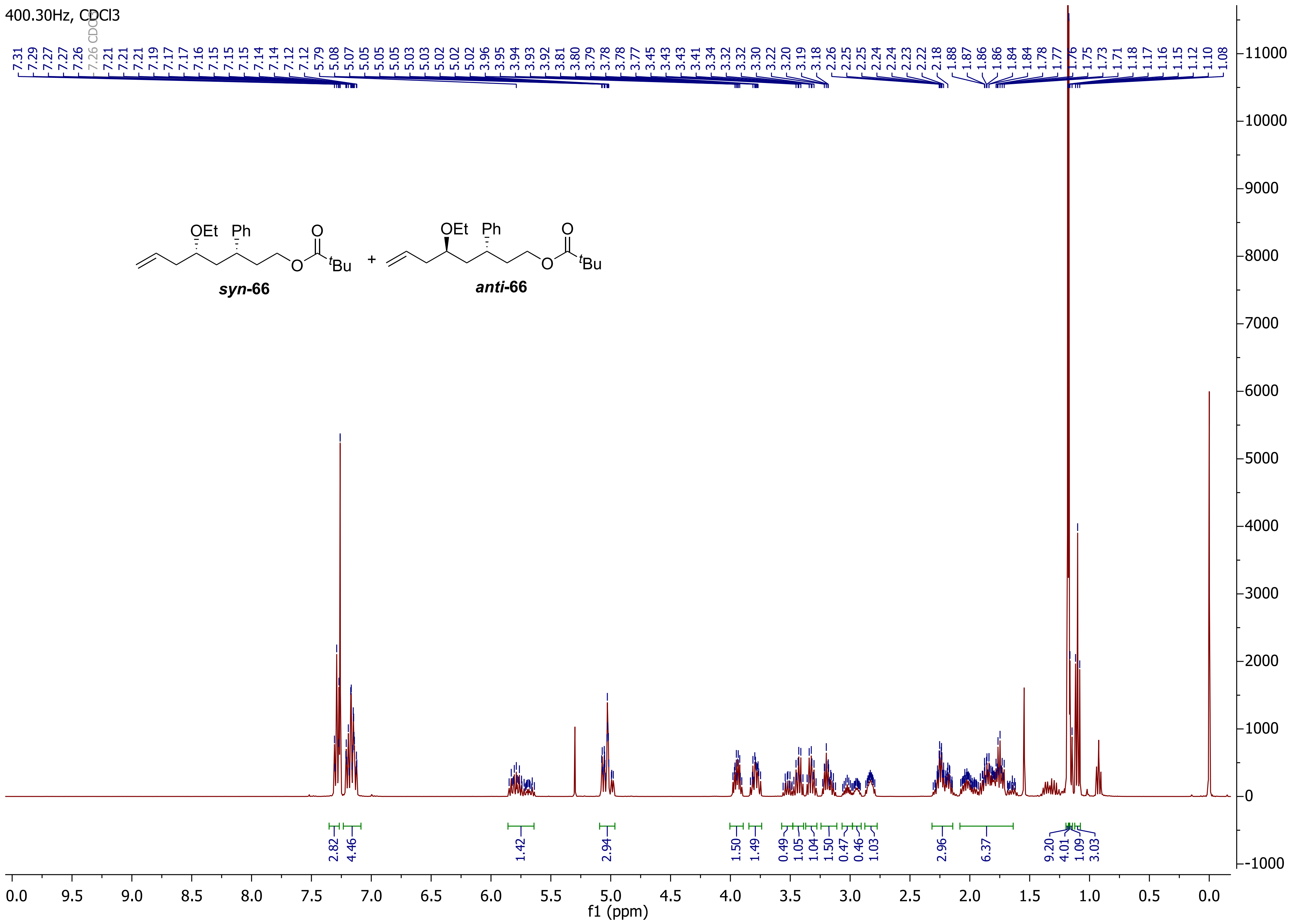
400.30Hz, CDCl3
7.31 7.29 7.27 7.27 7.26 7.26 CDCl3 7.21 7.21 7.21 7.19 7.17 7.17 7.16 7.15 7.15 7.15 7.14 7.14 7.12 7.12 5.79 5.08 5.07 5.05 5.05 5.05 5.03 5.03 5.02 5.02 5.02 3.96 3.95 3.94 3.93 3.92 3.81 3.80 3.79 3.78 3.78 3.77 3.45 3.43 3.43 3.41 3.34 3.32 3.32 3.30 3.22 3.20 3.19 3.18 2.26 2.25 2.25 2.24 2.24 2.23 2.22 2.18 1.88 1.87 1.86 1.86 1.84 1.84 1.78 1.77 1.76 1.75 1.73 1.71 1.18 1.17 1.16 1.15 1.12 1.10 1.08
OEt
Ph
O
O
tBu
syn-66
+
OEt
Ph
O
O
tBu
anti-66
2.82 4.46 1.42 2.94 1.50 1.49 0.49 1.05 1.04 1.50 0.47 0.46 1.03 2.96 6.37 9.20 4.01 1.09 3.03
f1 (ppm)

100.67Hz, CDCl3

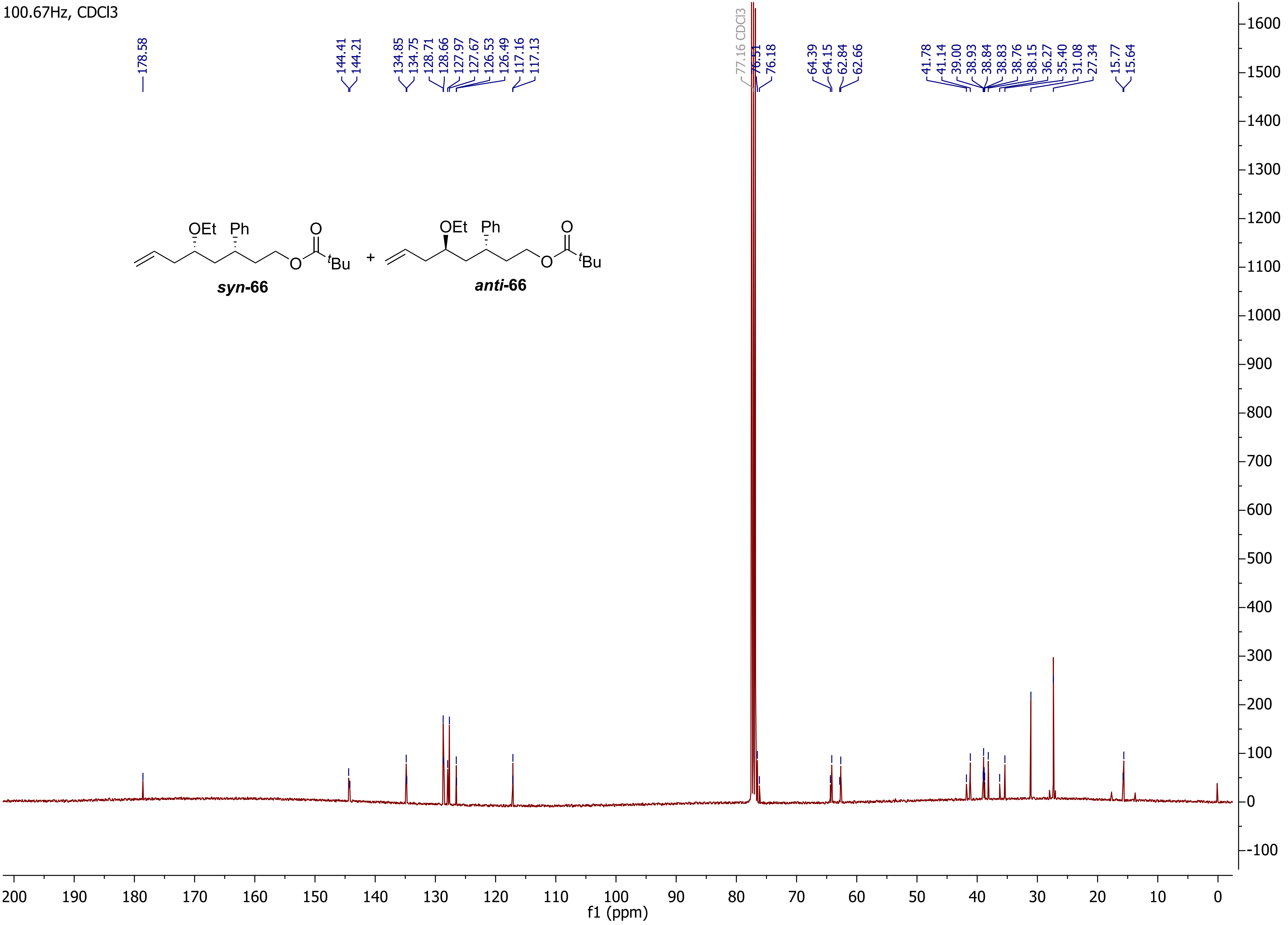

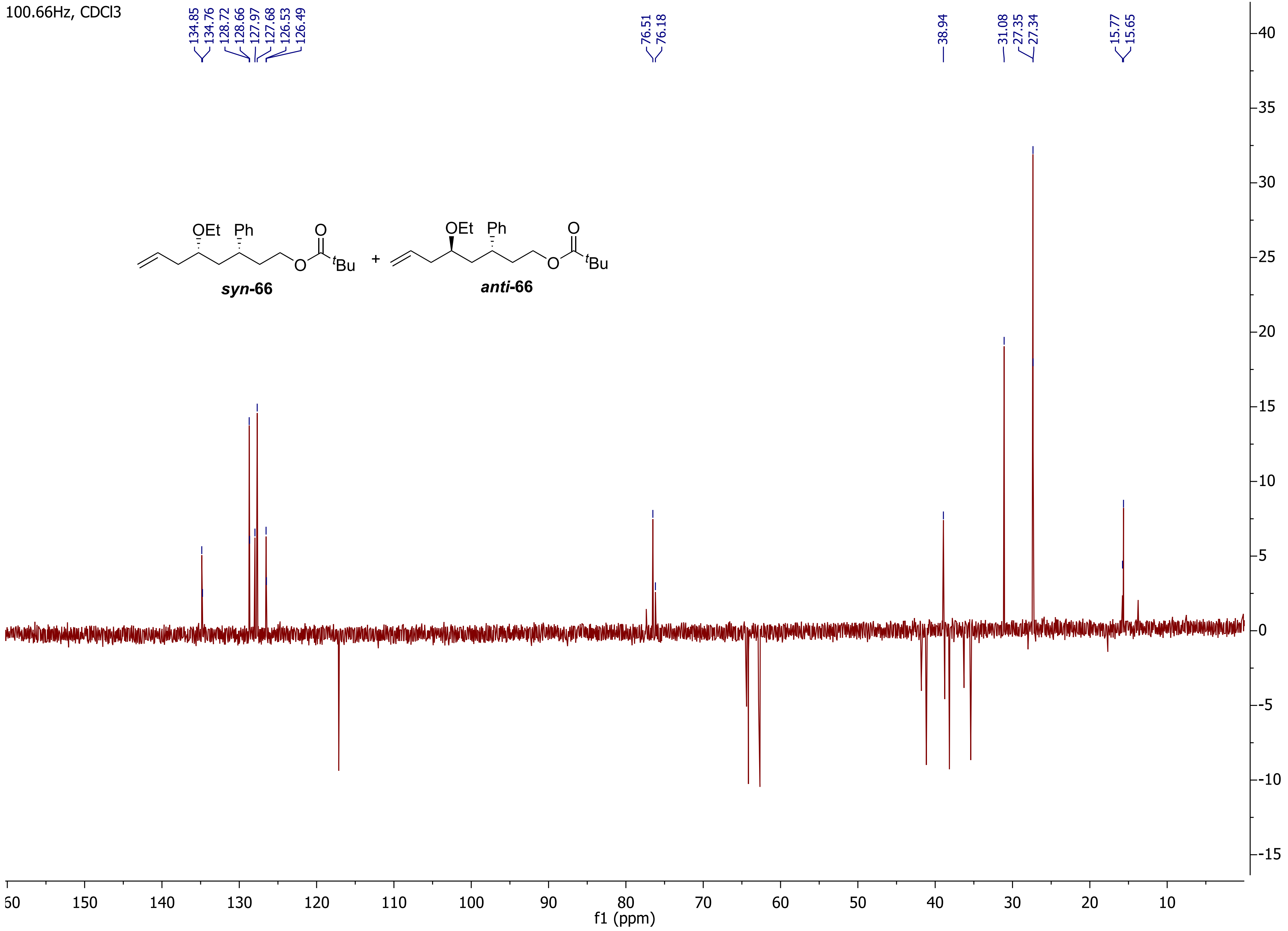
100.66Hz, CDCl3
134.85
134.76
128.72
128.66
127.97
127.68
126.53
126.49
76.51
76.18
38.94
31.08
27.35
27.34
15.77
15.65
OEt Ph O O tBu
syn-66
+
anti-66
f1 (ppm)

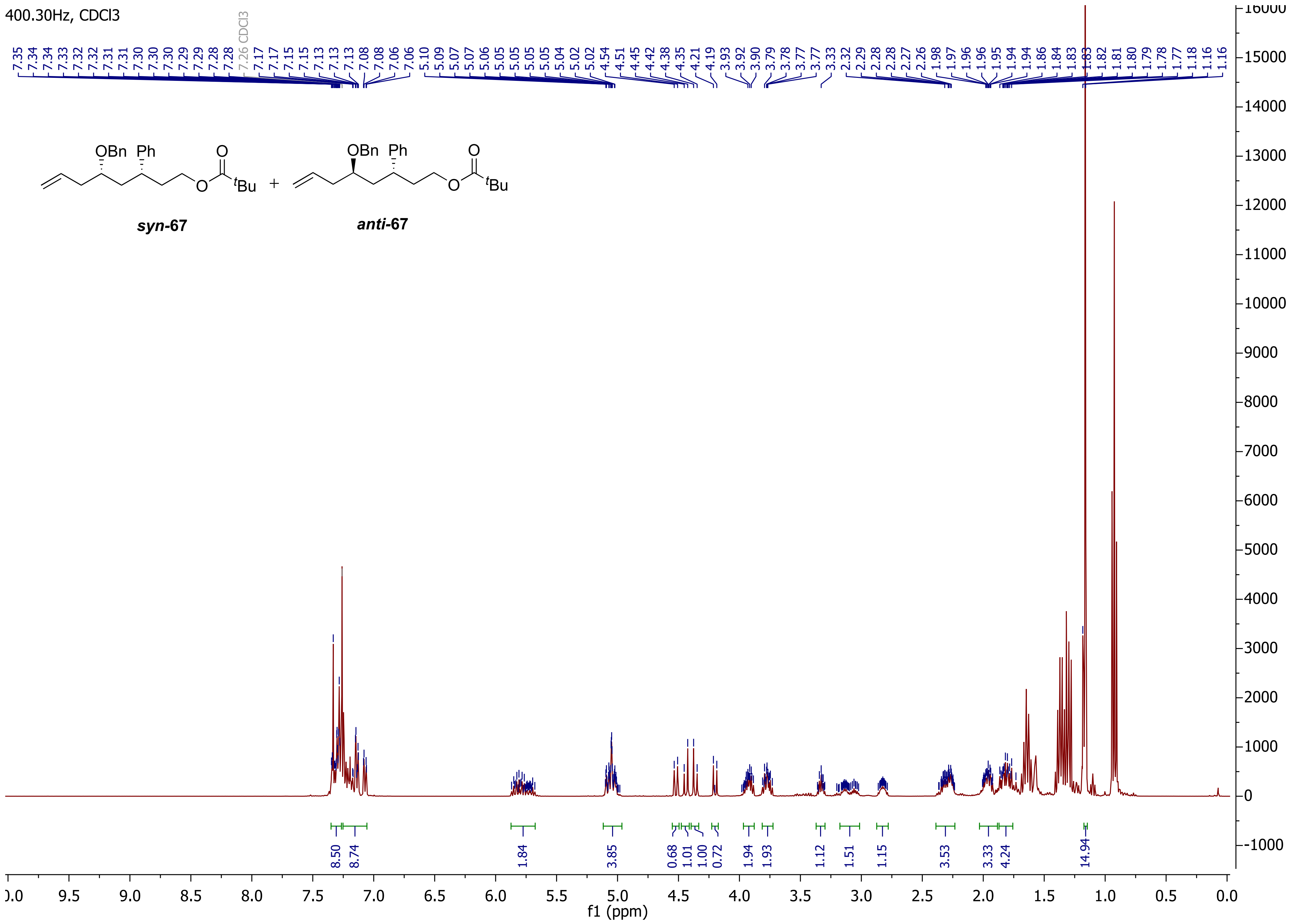
400.30Hz, CDCl3
OBn
Ph
O
tBu
+
syn-67
anti-67
8.50
8.74
1.84
3.85
0.68
1.01
1.00
0.72
1.94
1.93
1.12
1.51
1.15
3.53
3.33
4.24
14.94
f1 (ppm)

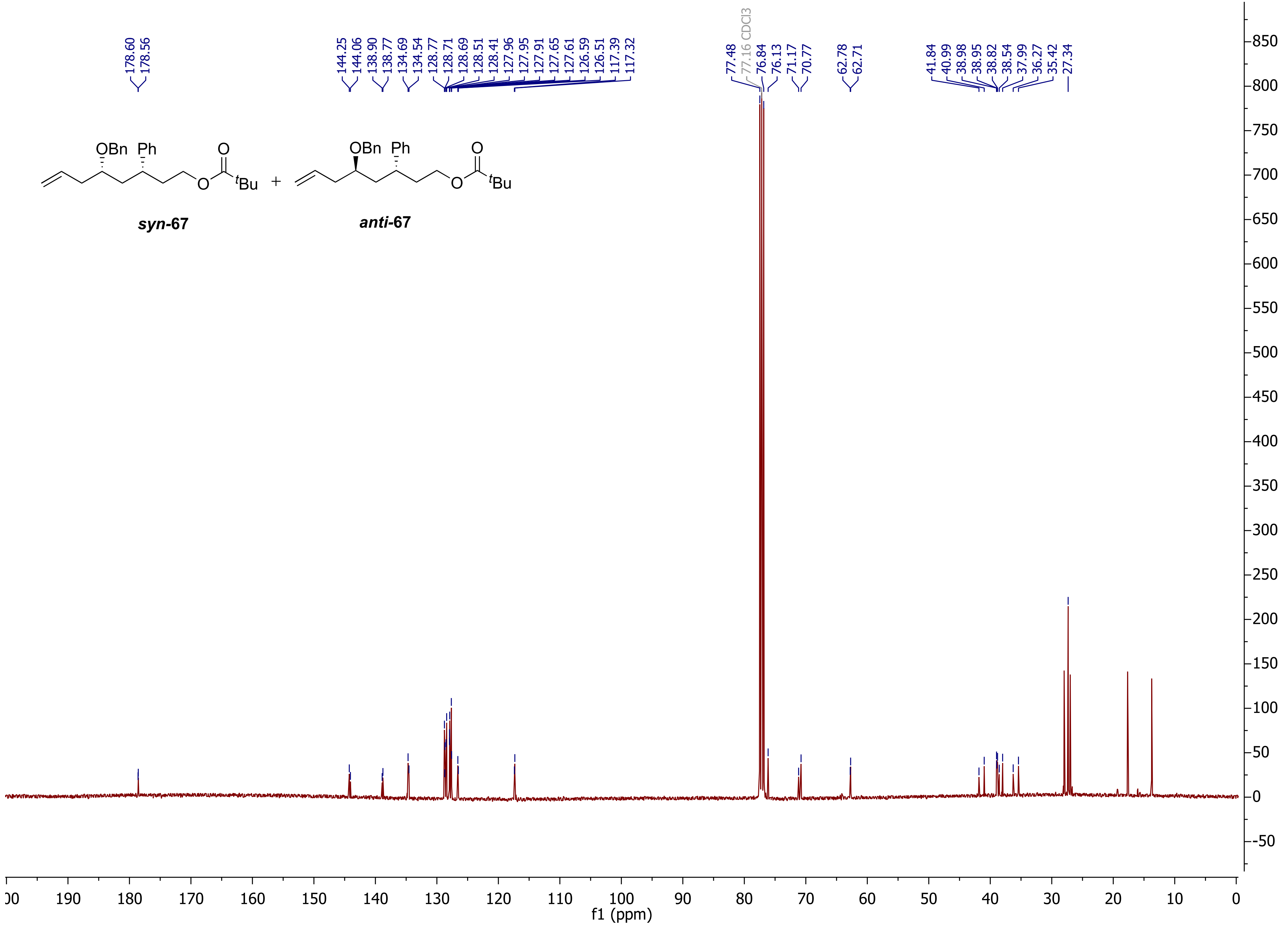
178.60
178.56
144.25
144.06
138.90
138.77
134.69
134.54
128.77
128.71
128.69
128.51
128.41
127.96
127.95
127.91
127.65
127.61
126.59
126.51
117.39
117.32
77.48
77.16 CDCl3
76.84
76.13
71.17
70.77
62.78
62.71
41.84
40.99
38.98
38.95
38.82
38.54
37.99
36.27
35.42
27.34
OBn
Ph
O
O
tBu
+
syn-67
anti-67
f1 (ppm)

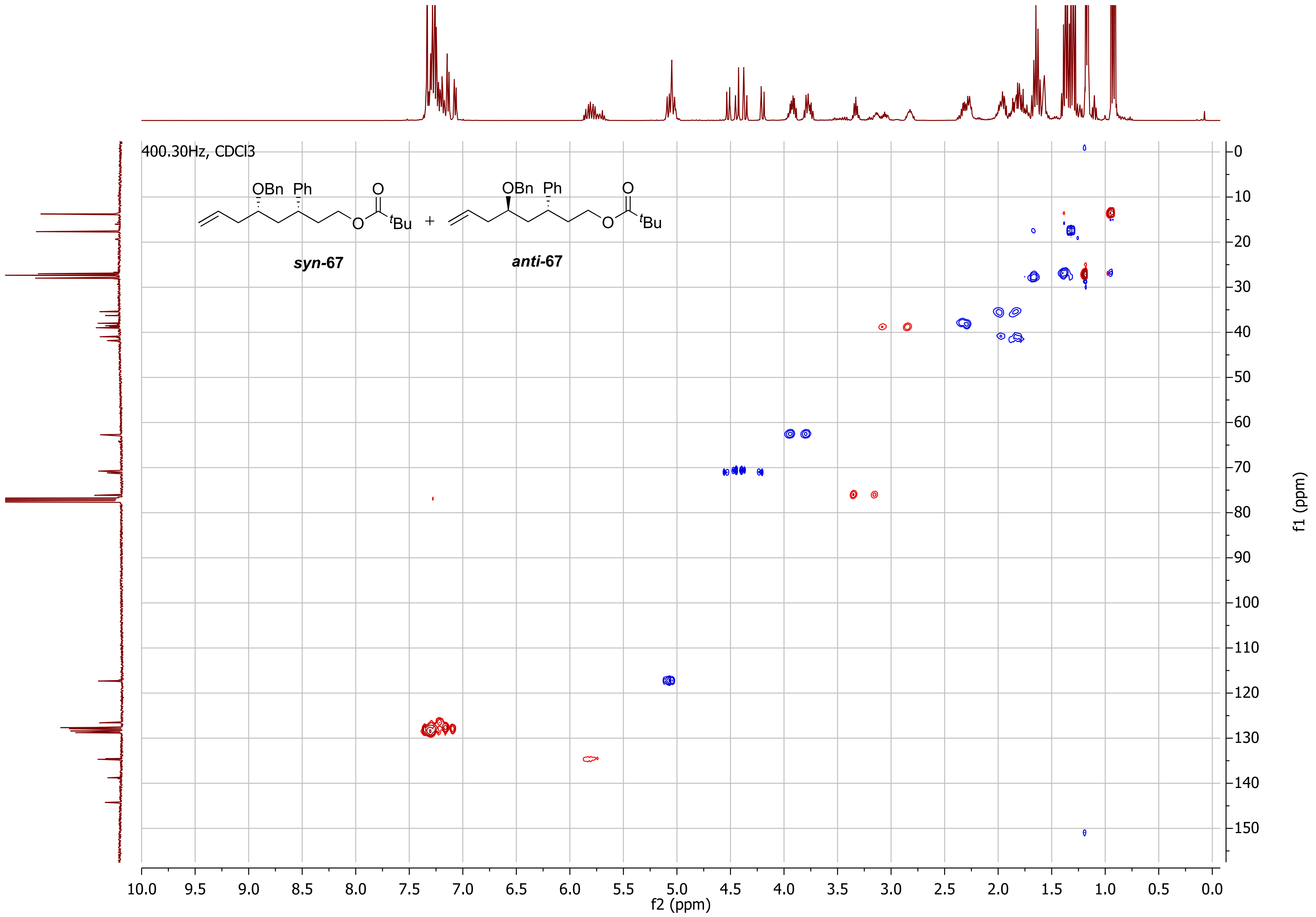
400.30Hz, CDCl3
OBn Ph O
tBu
syn-67
anti-67
f2 (ppm)
f1 (ppm)

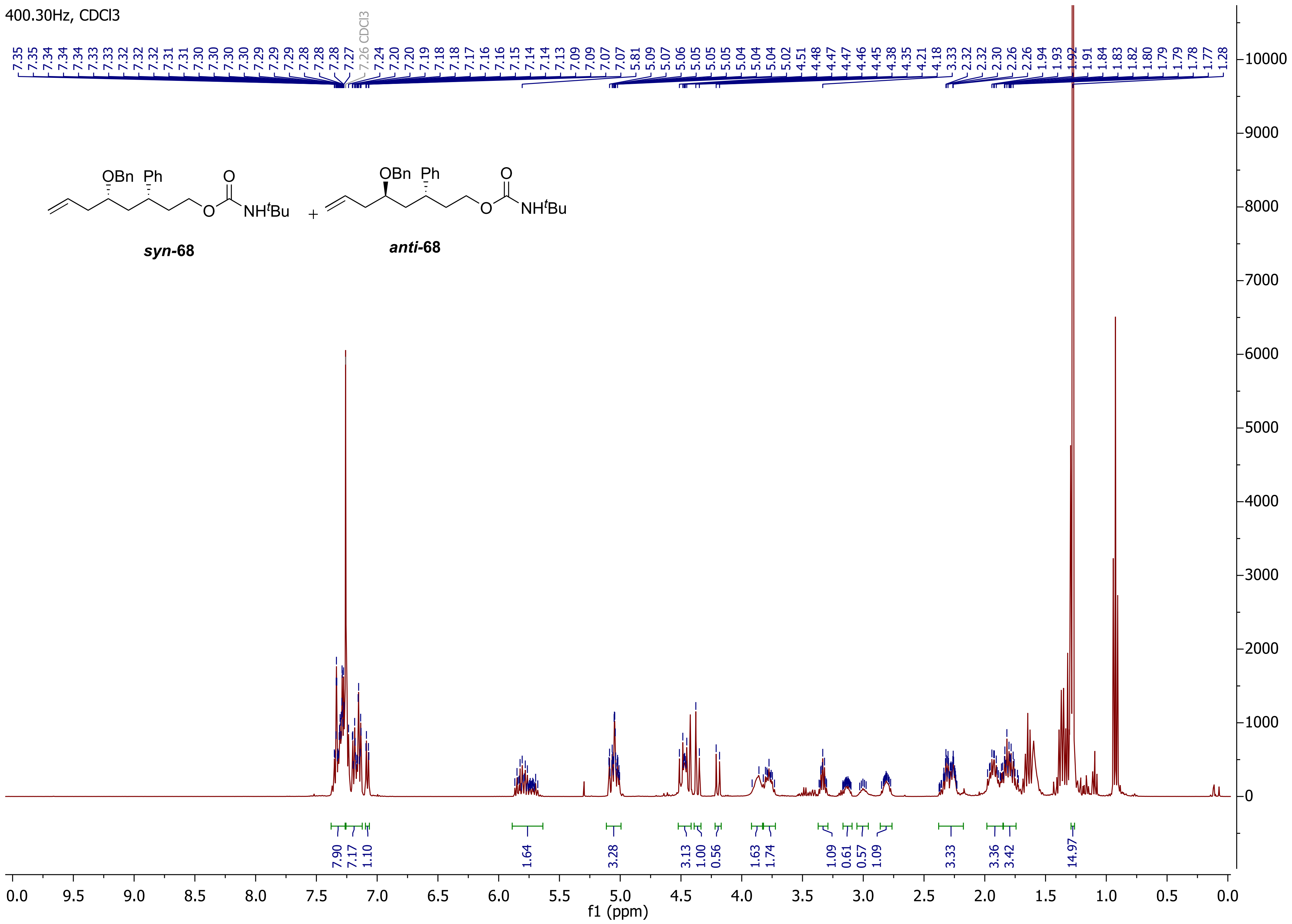
400.30Hz, CDCl3
7.35 7.35 7.34 7.34 7.33 7.33 7.32 7.32 7.31 7.31 7.30 7.30 7.30 7.29 7.29 7.29 7.28 7.28 7.28 7.27 7.26 CDCl3 7.24 7.20 7.20 7.19 7.18 7.18 7.17 7.16 7.16 7.15 7.14 7.14 7.13 7.09 7.09 7.07 7.07 5.81 5.09 5.07 5.06 5.05 5.05 5.05 5.04 5.04 5.04 5.02 4.51 4.48 4.47 4.47 4.46 4.45 4.38 4.35 4.21 4.18 3.33 2.32 2.32 2.30 2.26 2.26 1.94 1.93 1.92 1.91 1.84 1.83 1.82 1.80 1.79 1.79 1.78 1.77 1.28
OBn Ph O O NH^{t}Bu
+
OBn Ph O O NH^{t}Bu
syn-68
anti-68
7.90 7.17 1.10 1.64 3.28 3.13 1.00 0.56 1.63 1.74 1.09 0.61 0.57 1.09 3.33 3.36 3.42 14.97
f1 (ppm)

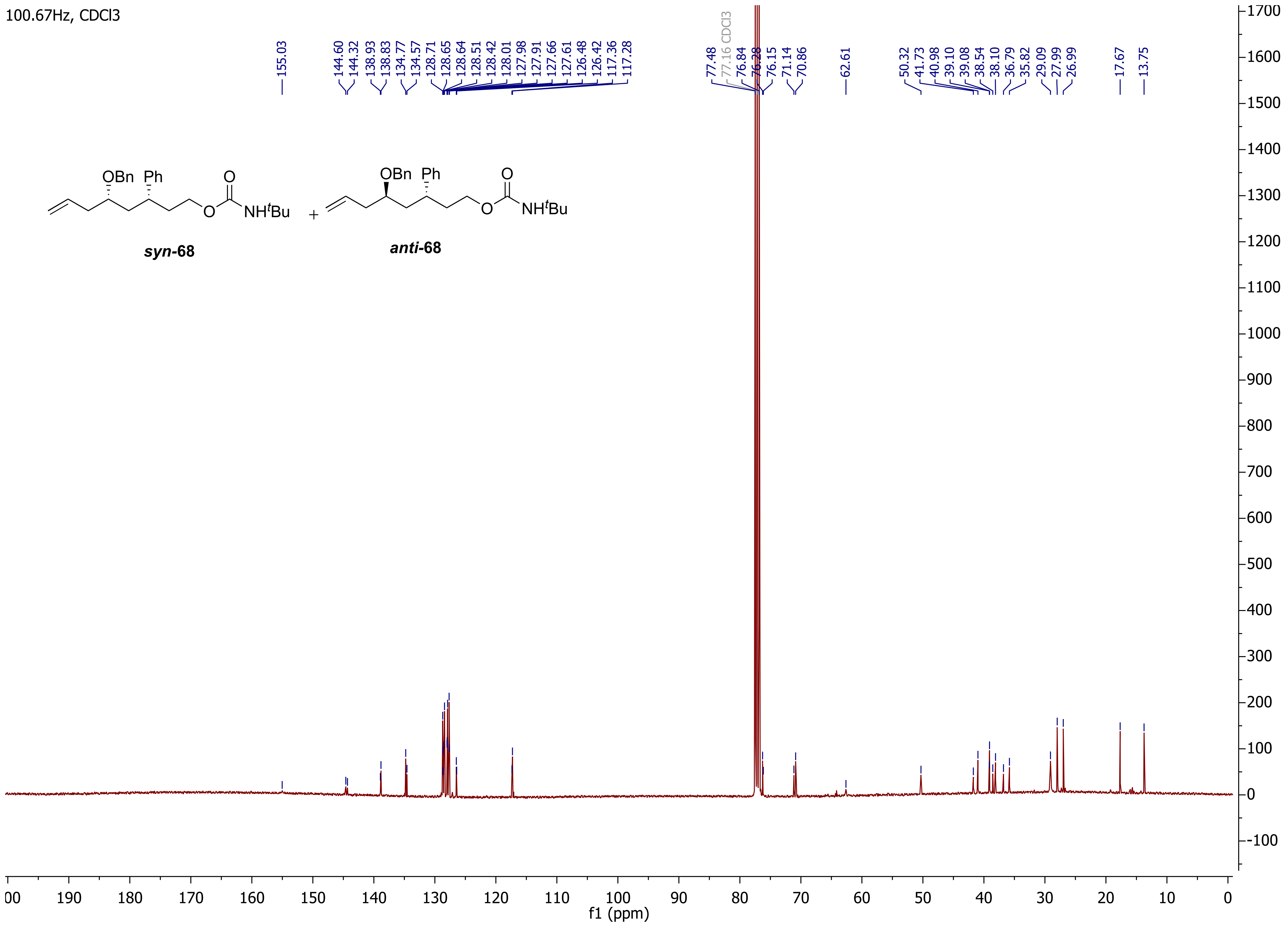
100.67Hz, CDCl3
155.03
144.60
144.32
138.93
138.83
134.77
134.57
128.71
128.65
128.64
128.51
128.42
128.01
127.98
127.91
127.66
127.61
126.48
126.42
117.36
117.28
77.48
77.16 CDCl3
76.84
76.28
76.15
71.14
70.86
62.61
50.32
41.73
40.98
39.10
39.08
38.54
38.10
36.79
35.82
29.09
27.99
26.99
17.67
13.75
OBn
Ph
O
O
NH^{t}Bu
+
syn-68
anti-68
f1 (ppm)

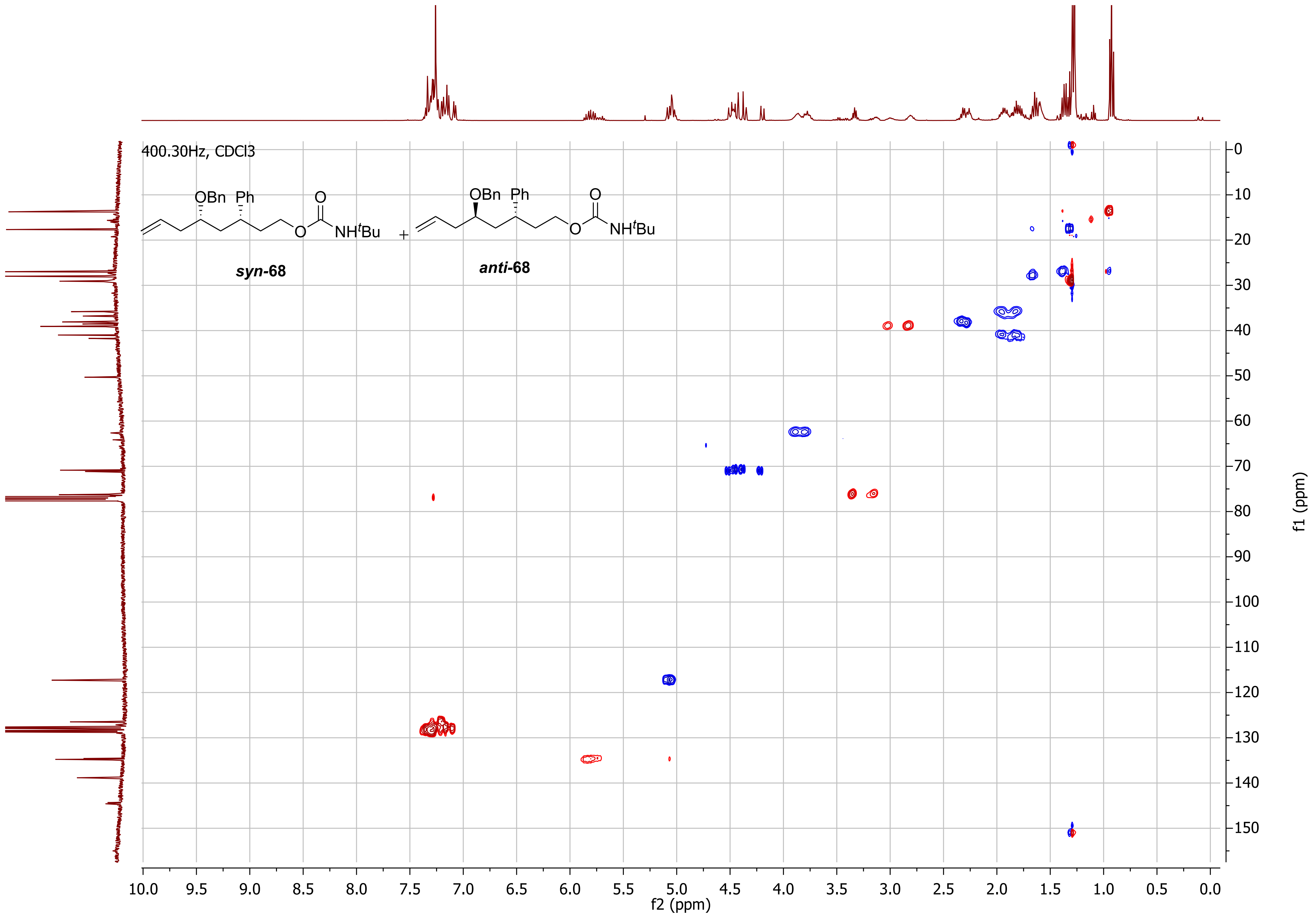

400.30Hz, CDCl3
syn-68
anti-68
f2 (ppm)
f1 (ppm)

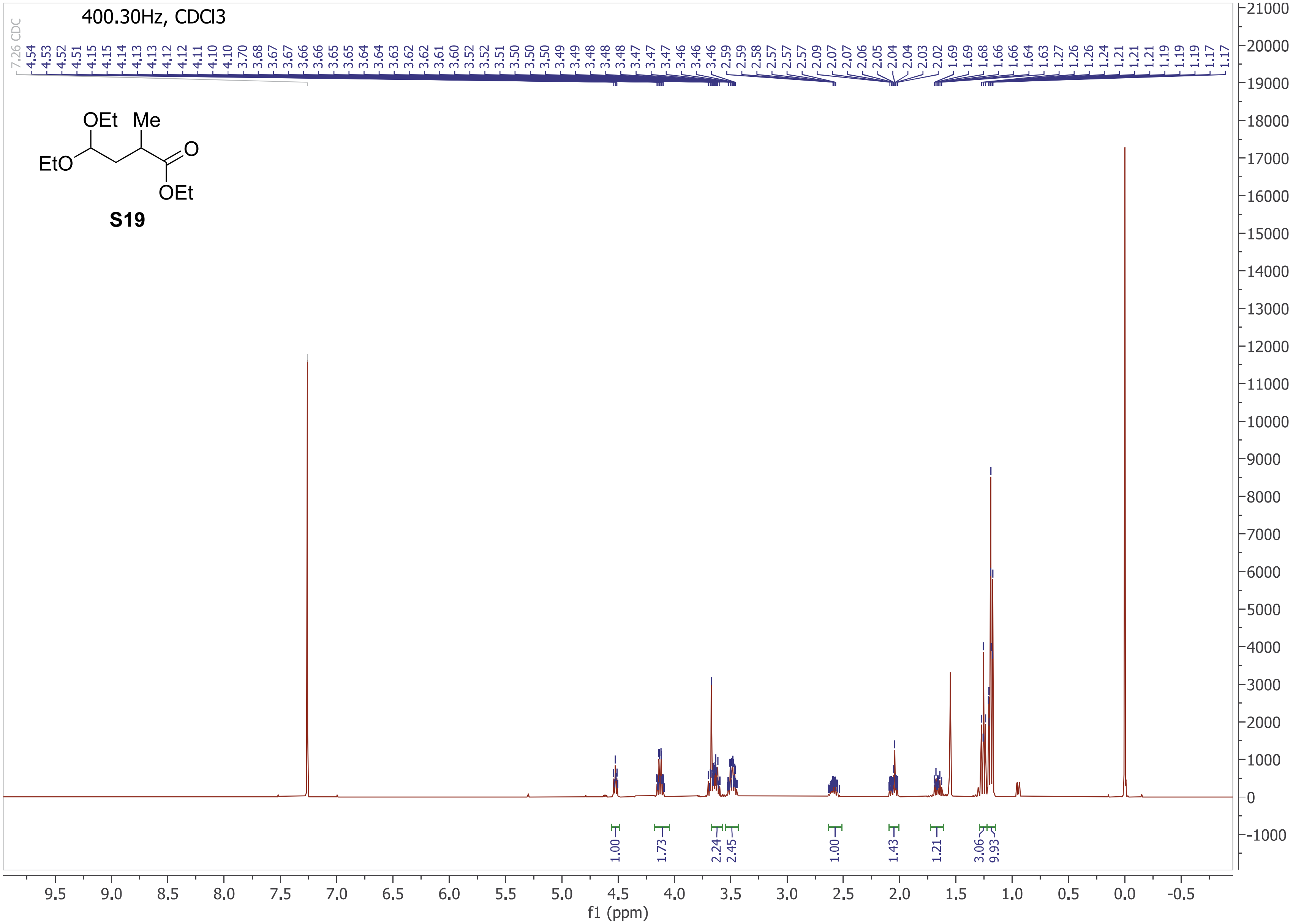
400.30Hz, CDCl3
OEt
Me
EtO
O
OEt
S19
f1 (ppm)

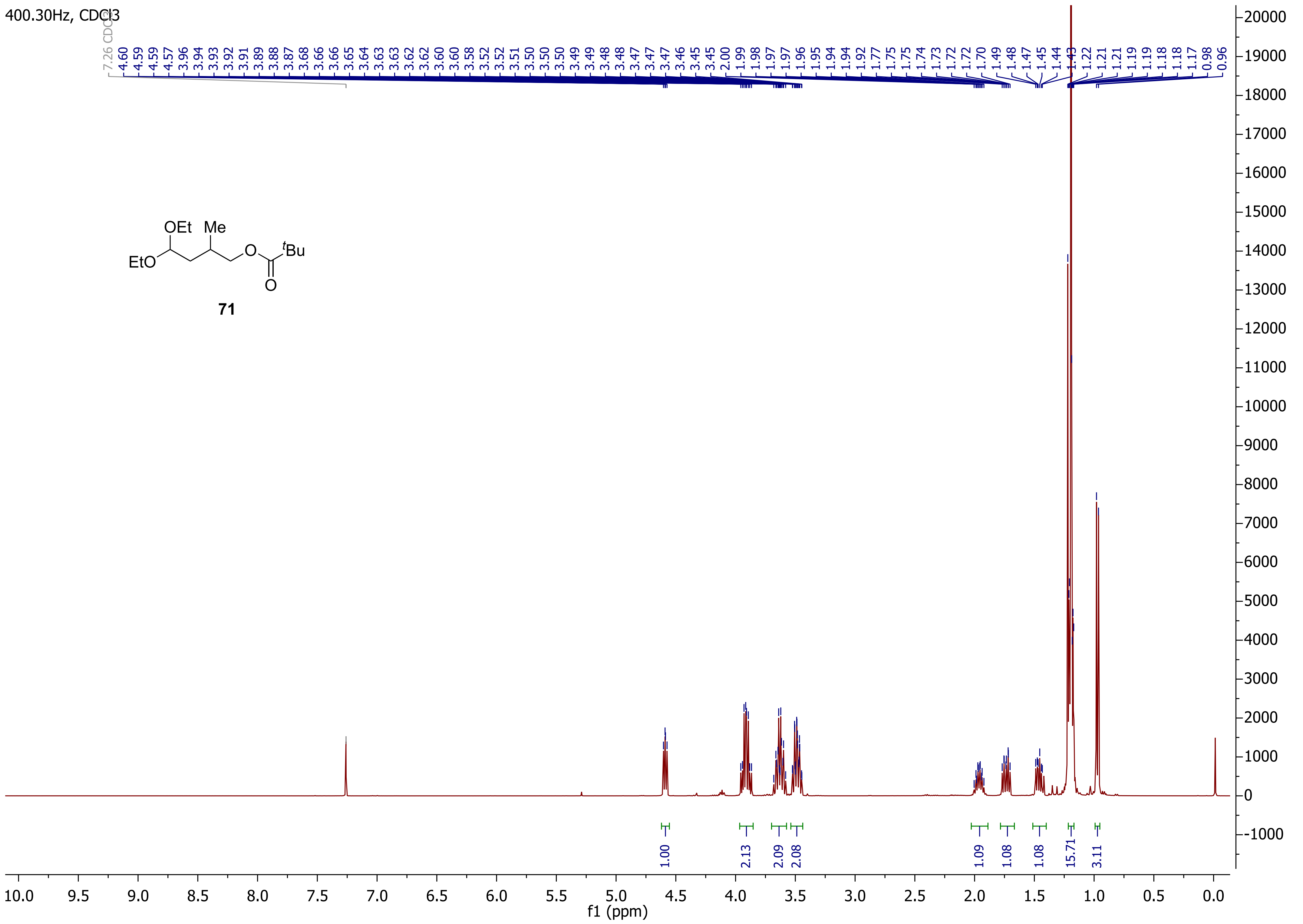
400.30Hz, CDCl3
OEt
Me
EtO
O
tBu
O
71
f1 (ppm)

100.67Hz, CDCl3

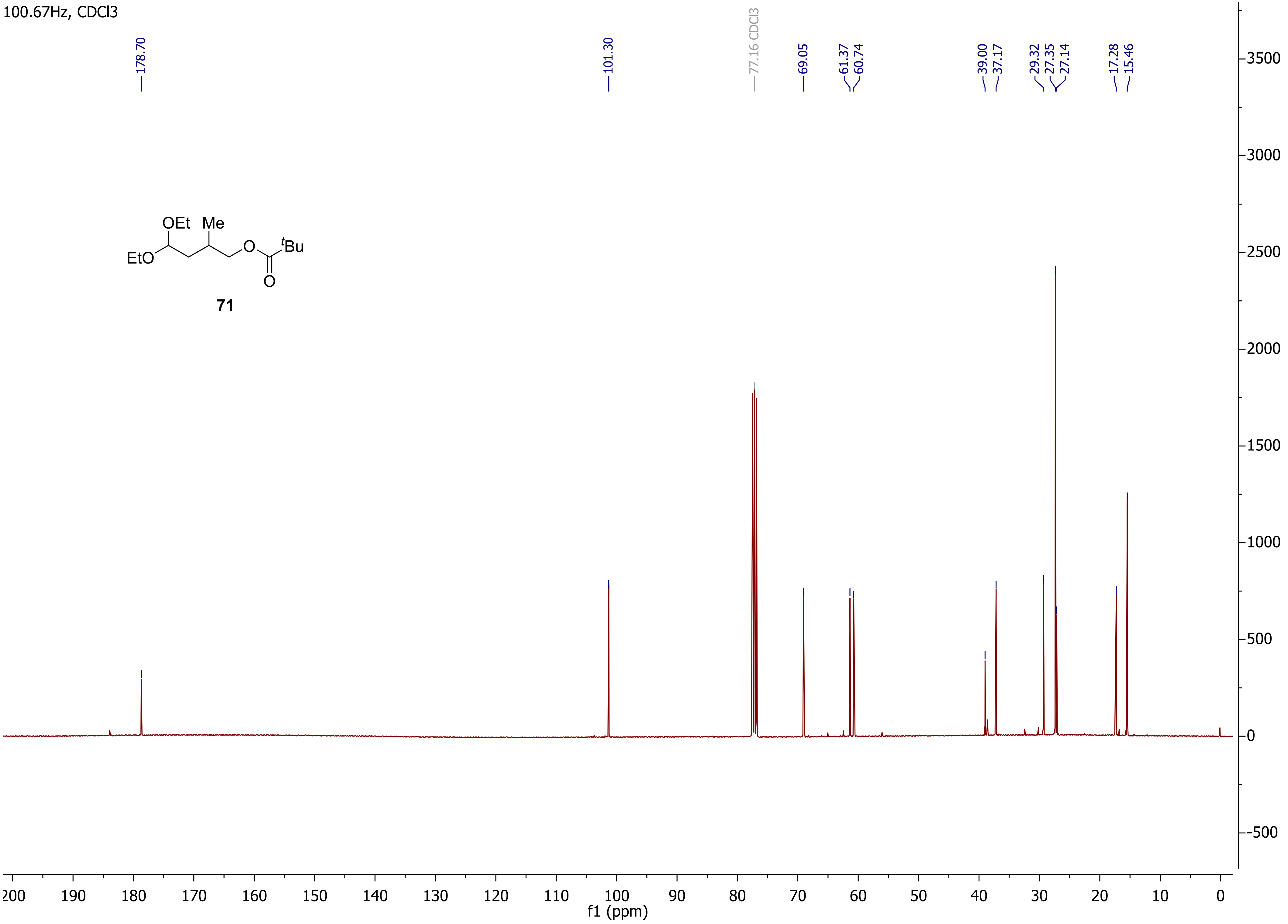

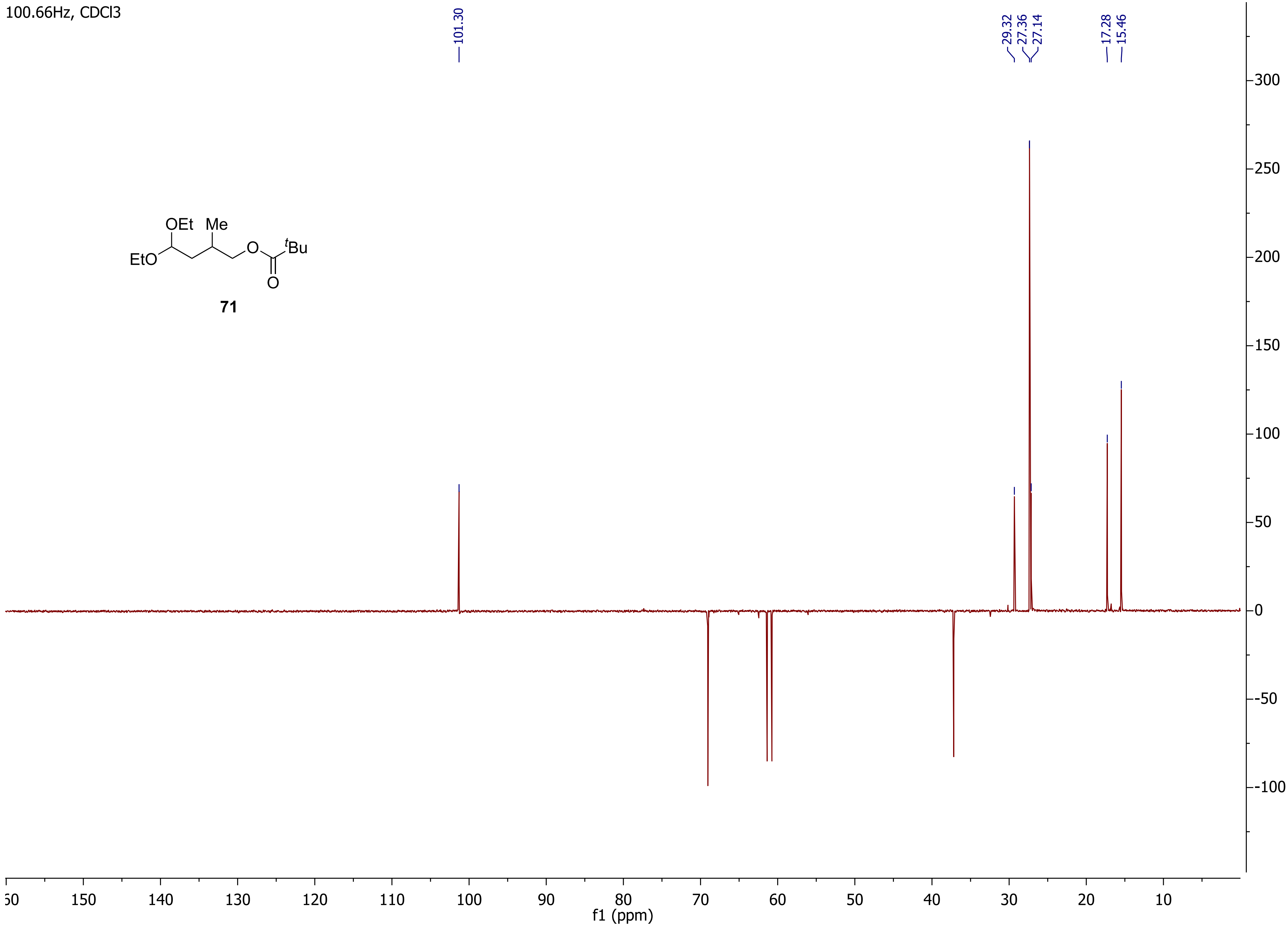
100.66Hz, CDCl3
101.30
29.32
27.36
27.14
17.28
15.46
OEt
Me
EtO
O
^{t}Bu
O
71
f1 (ppm)

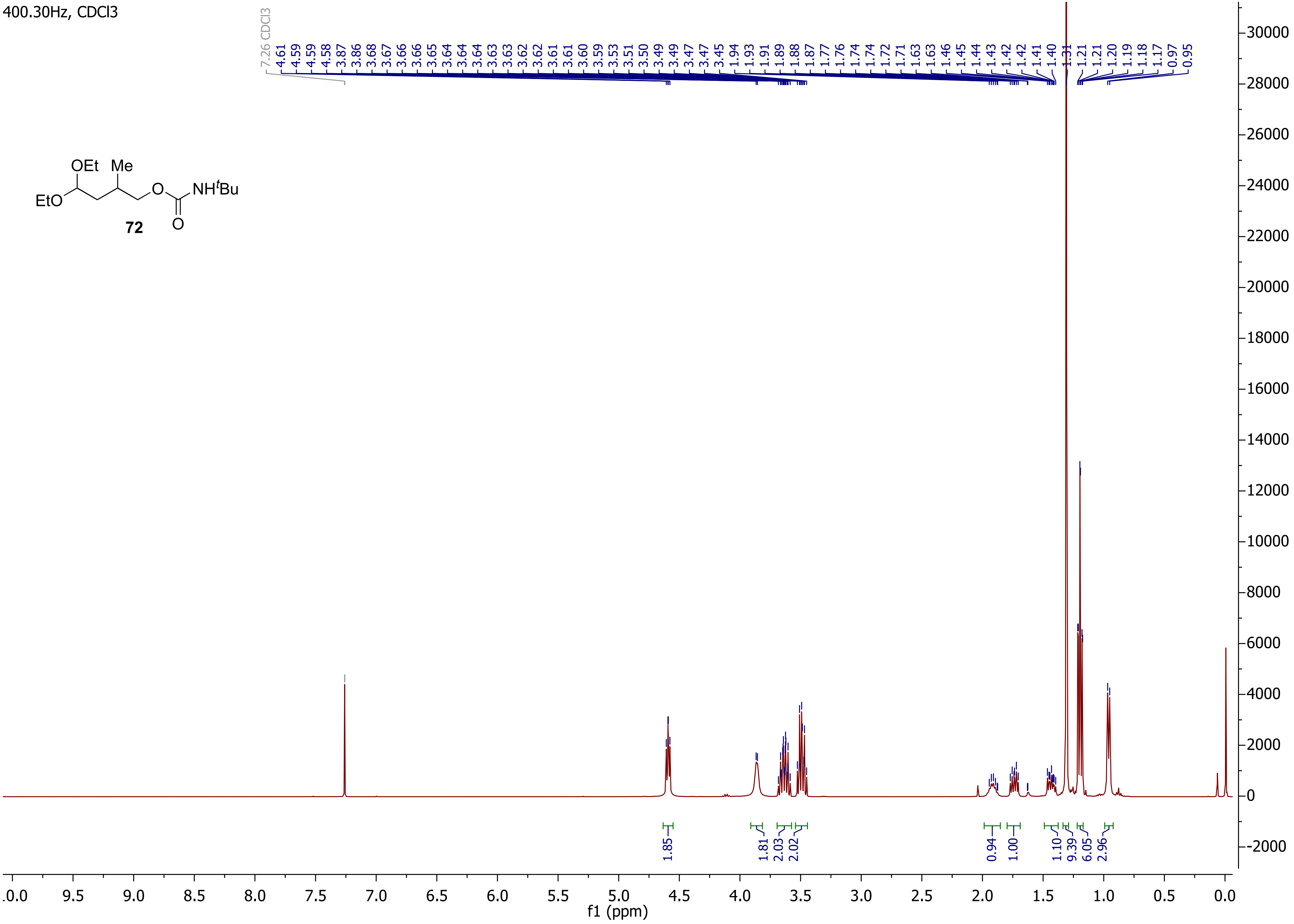

400.30Hz, CDCl3
OEt Me
EtO
O
NH^{t}Bu
O
72
7.26 CDCl3
4.61 4.59 4.59 4.58 3.87 3.86 3.68 3.67 3.66 3.66 3.65 3.64 3.64 3.63 3.63 3.62 3.62 3.61 3.61 3.60 3.59 3.53 3.51 3.50 3.49 3.49 3.47 3.47 3.45 1.94 1.93 1.91 1.89 1.88 1.87 1.77 1.76 1.74 1.74 1.72 1.71 1.63 1.63 1.46 1.45 1.44 1.43 1.42 1.42 1.41 1.40 1.31 1.21 1.21 1.20 1.19 1.18 1.17 0.97 0.95
1.85 1.81 2.03 2.02 0.94 1.00 1.10 9.39 6.05 2.96
f1 (ppm)

100.67Hz, CDCl3

72

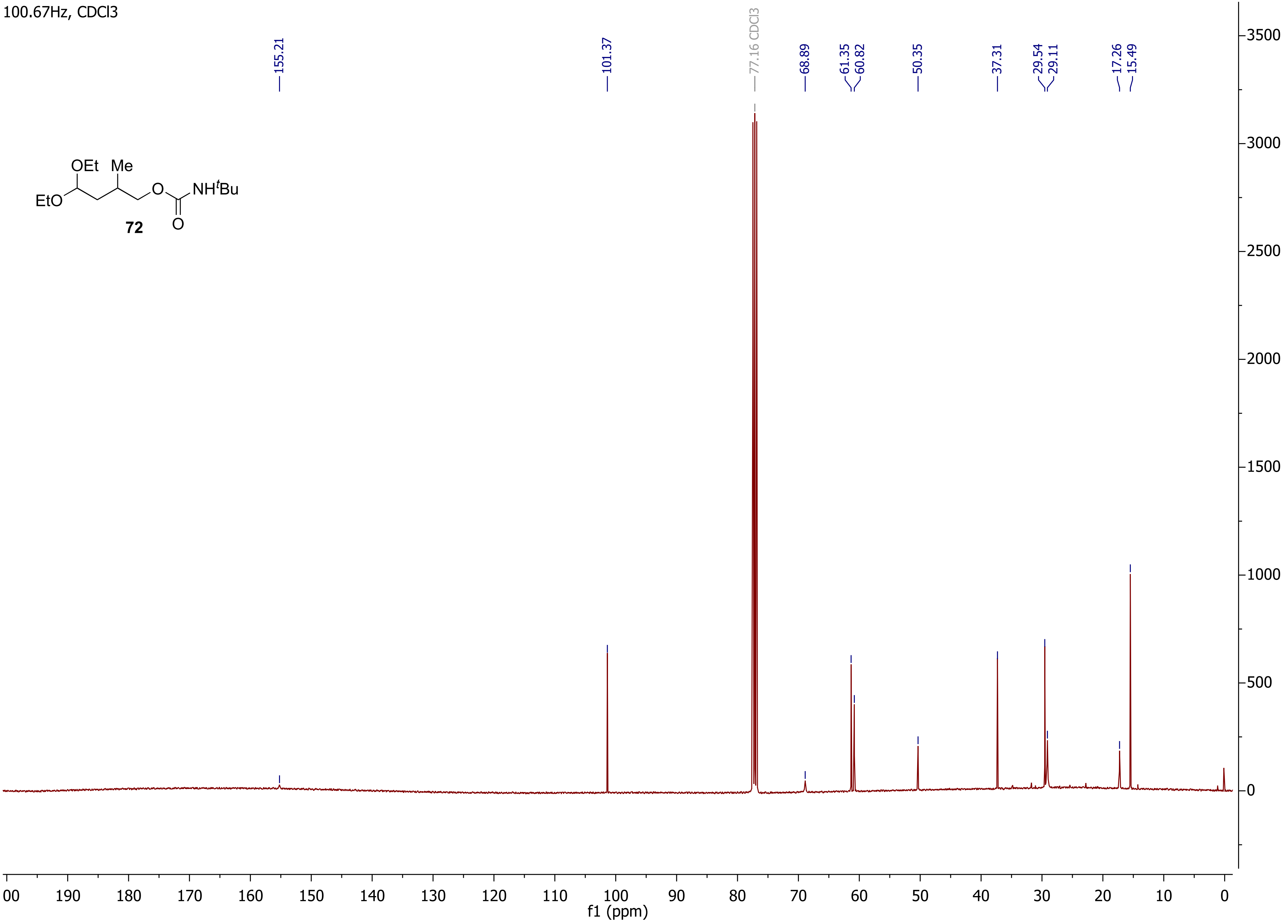

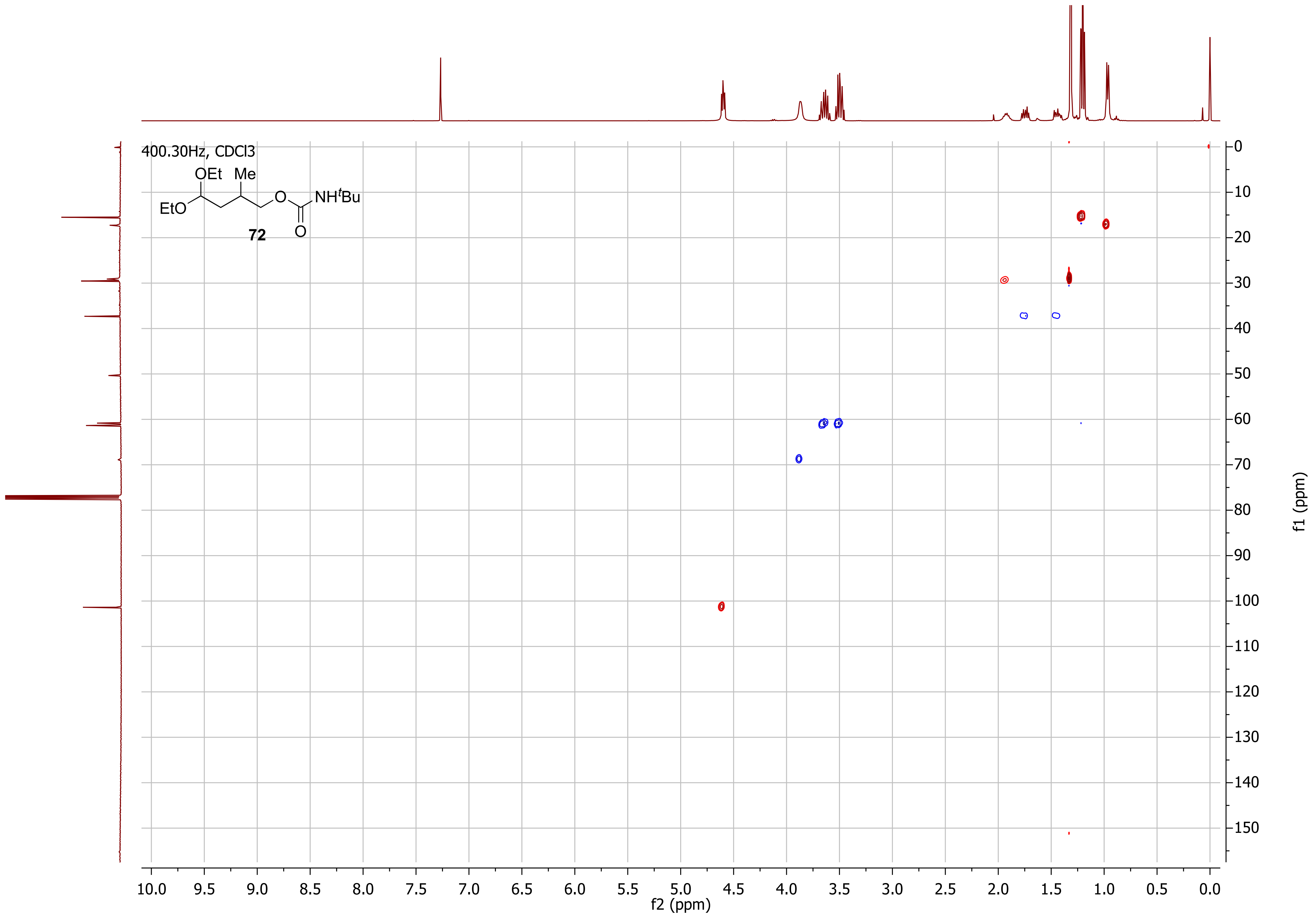
400.30Hz, CDCl3
OEt
Me
EtO
O
NH^{t}Bu
O
72
f2 (ppm)
f1 (ppm)

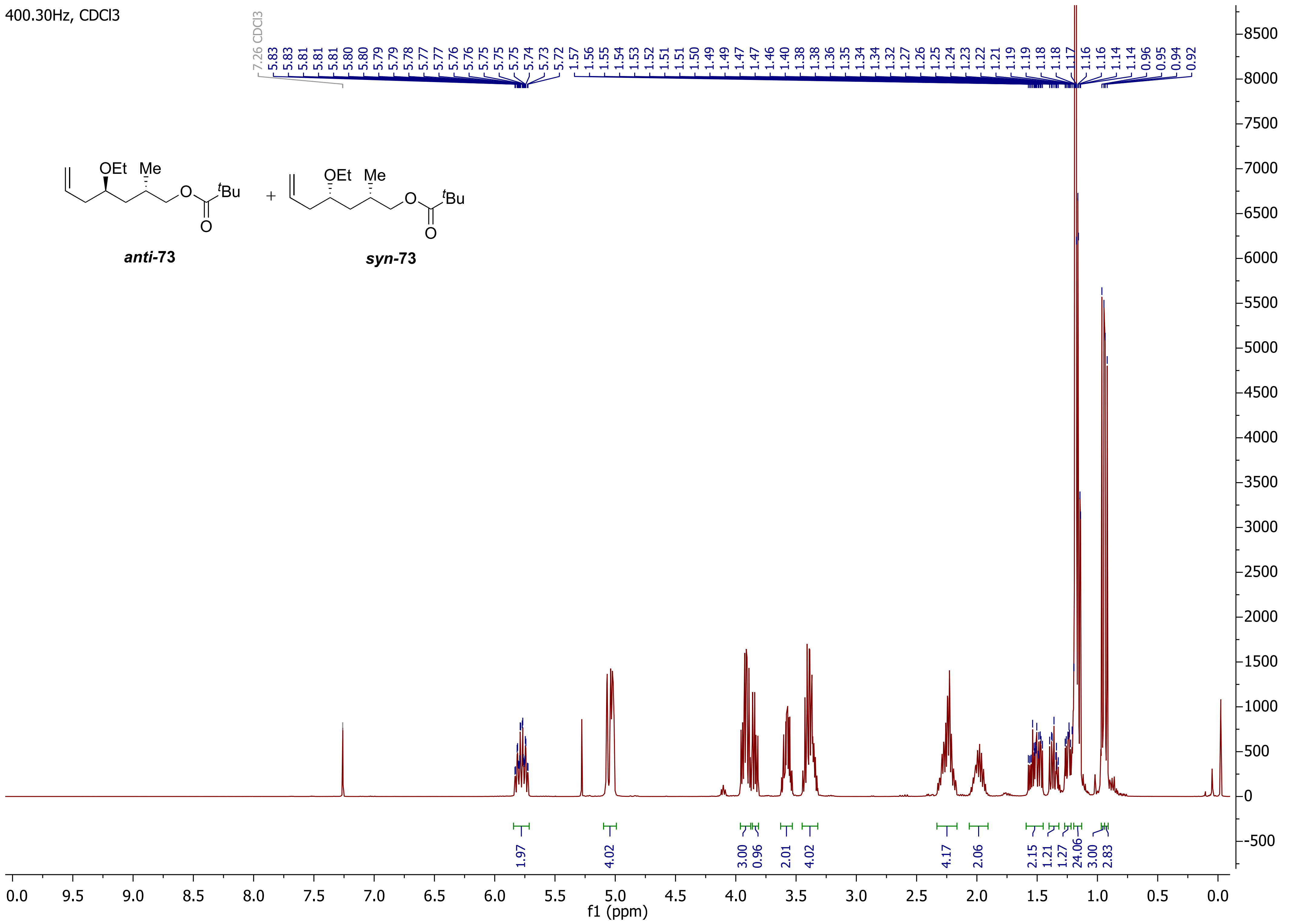

400.30Hz, CDCl3
7.26 CDCl3
5.83 5.83 5.81 5.81 5.81 5.80 5.80 5.79 5.79 5.78 5.77 5.77 5.76 5.76 5.75 5.75 5.75 5.74 5.74 5.73 5.72
1.57 1.56 1.55 1.54 1.53 1.52 1.51 1.51 1.50 1.49 1.49 1.47 1.47 1.46 1.40 1.38 1.38 1.36 1.35 1.34 1.32 1.27 1.26 1.25 1.24 1.23 1.22 1.21 1.19 1.19 1.18 1.18 1.17 1.16 1.16 1.14 1.14 0.96 0.95 0.94 0.92
OEt
Me
tBu
O
anti-73
syn-73
1.97
4.02
3.00
0.96
2.01
4.02
4.17
2.06
2.15
1.21
1.27
24.06
3.00
2.83
f1 (ppm)

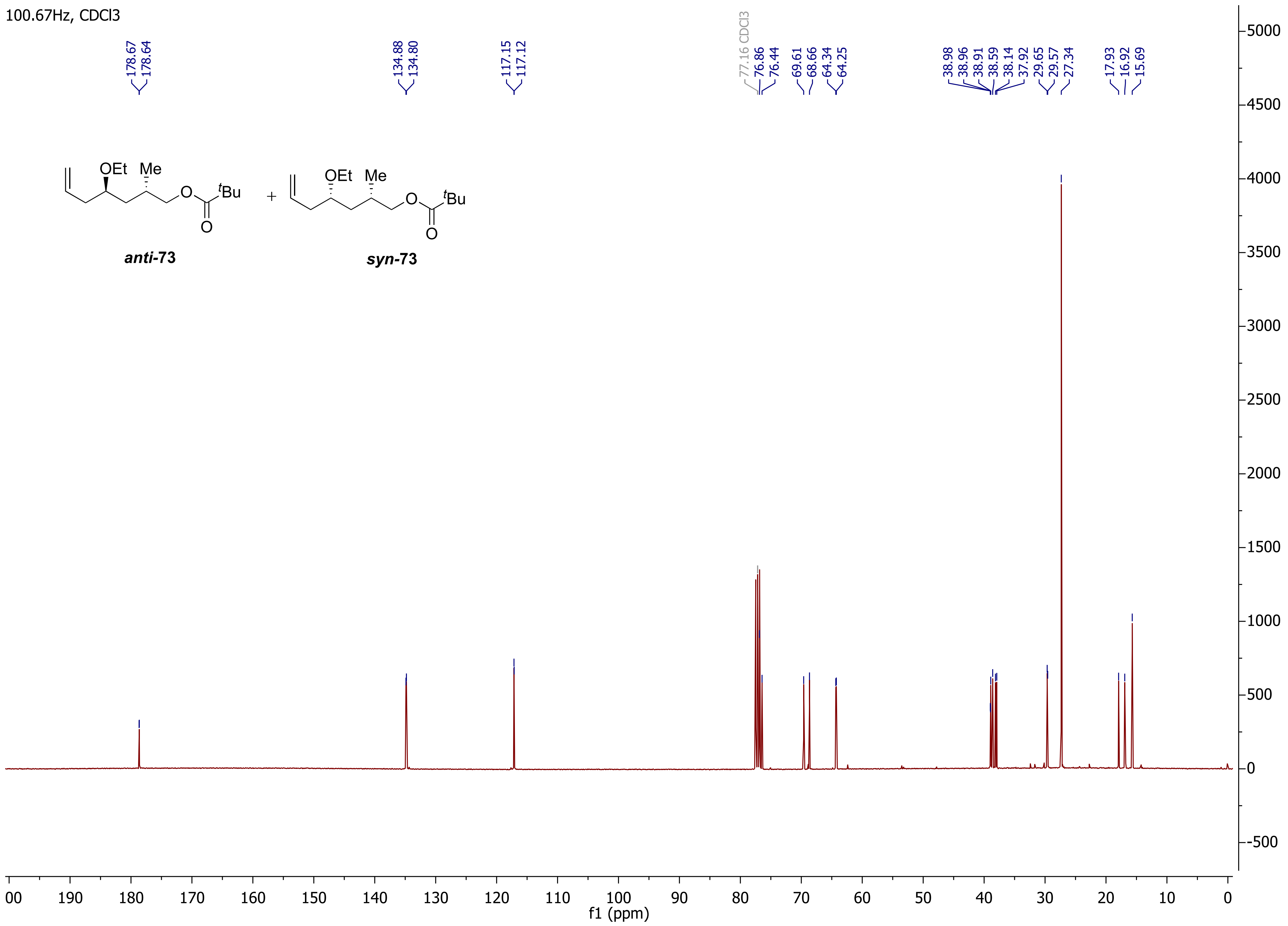
100.67Hz, CDCl3
178.67
178.64
134.88
134.80
117.15
117.12
77.16 CDCl3
76.86
76.44
69.61
68.66
64.34
64.25
38.98
38.96
38.91
38.59
38.14
37.92
29.65
29.57
27.34
17.93
16.92
15.69
OEt
Me
tBu
O
anti-73
syn-73
f1 (ppm)

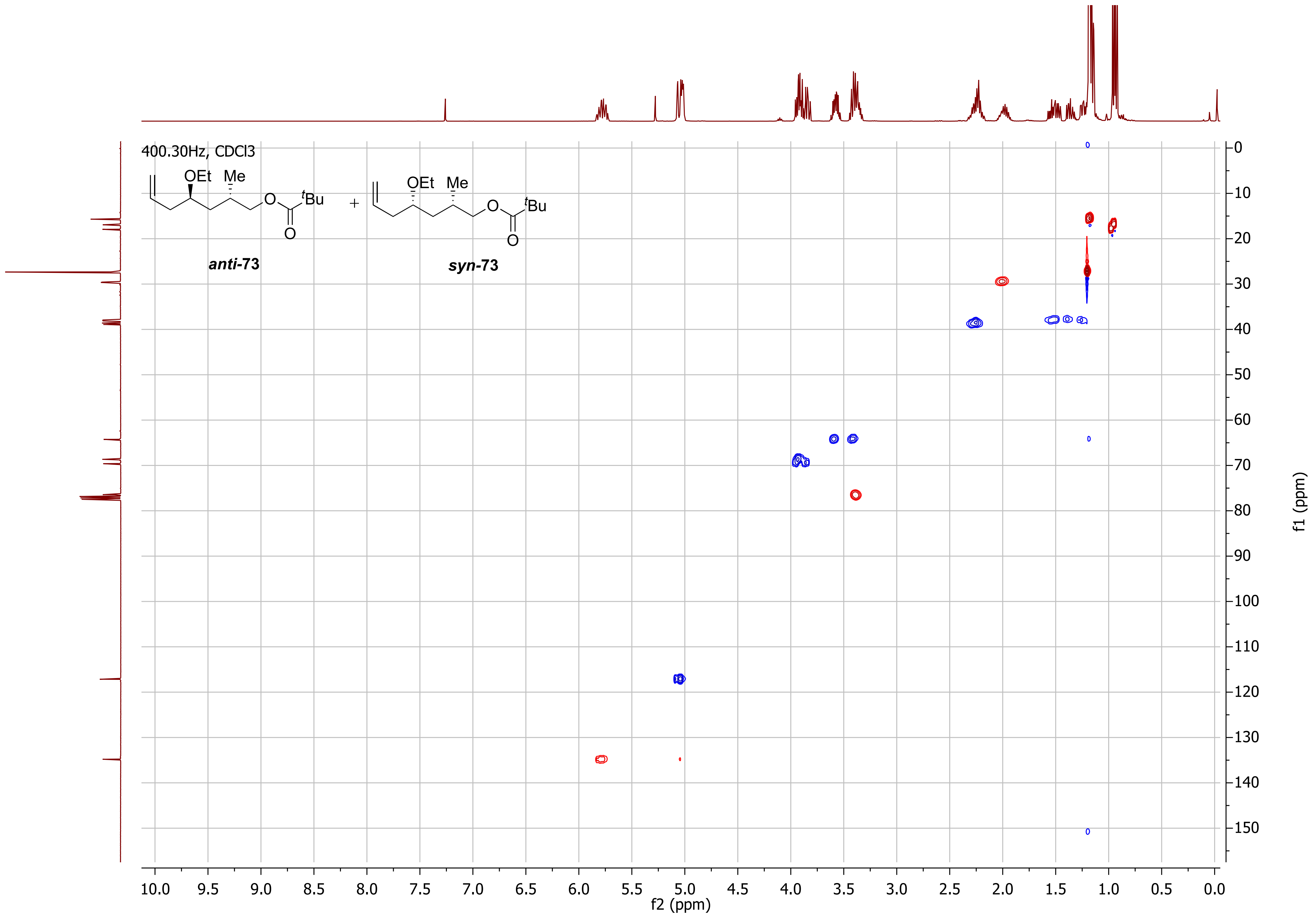

400.30Hz, CDCl3
OEt Me
O
tBu
O
anti-73
+
OEt Me
O
tBu
O
syn-73
f2 (ppm)
f1 (ppm)

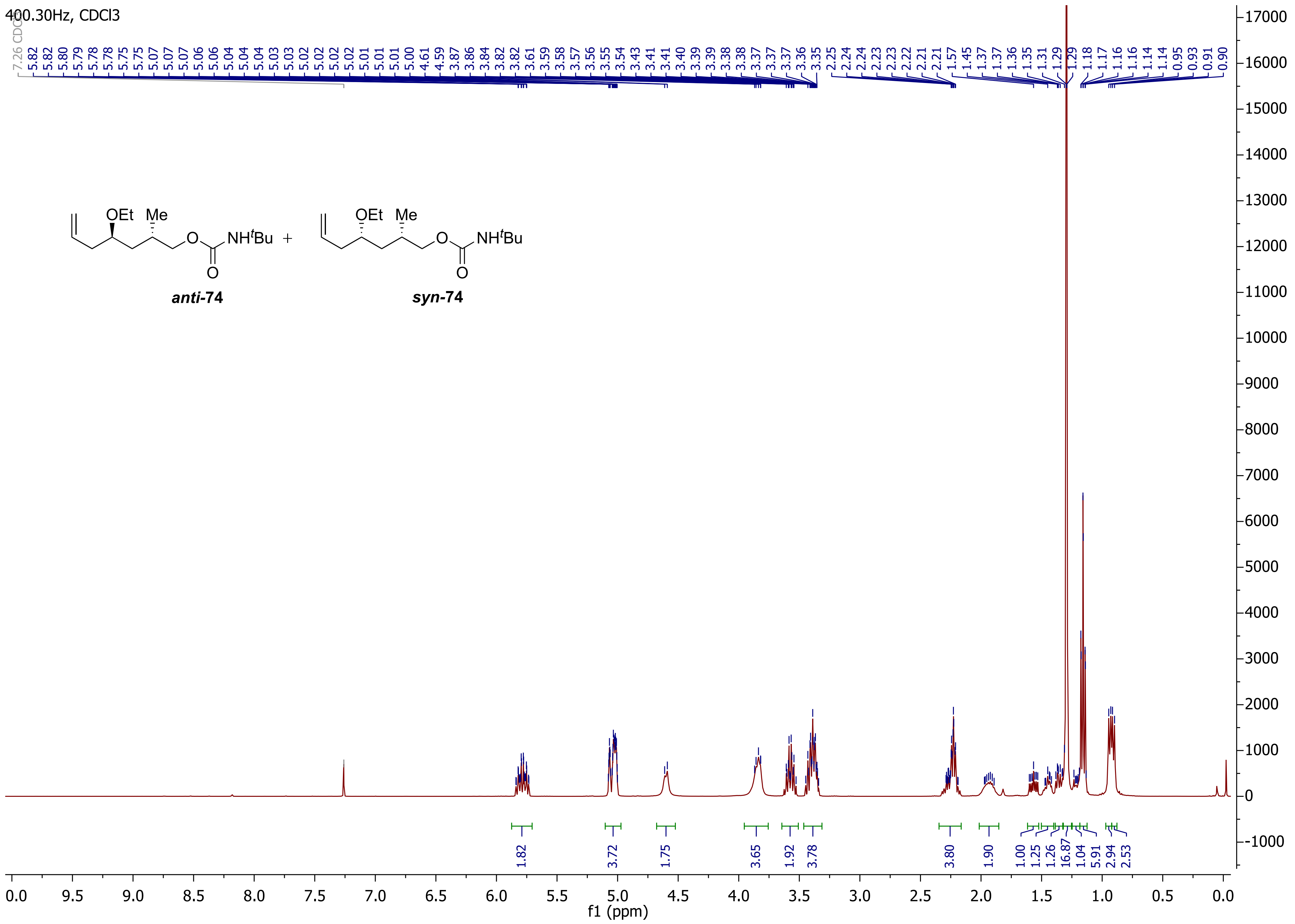
400.30Hz, CDCl3
7.26 CDCl3
5.82 5.82 5.80 5.79 5.78 5.78 5.75 5.75 5.07 5.07 5.07 5.06 5.06 5.04 5.04 5.04 5.03 5.03 5.02 5.02 5.02 5.02 5.01 5.01 5.01 5.00 4.61 4.59 3.87 3.86 3.84 3.82 3.82 3.61 3.59 3.58 3.57 3.56 3.55 3.54 3.43 3.41 3.41 3.40 3.39 3.39 3.38 3.38 3.37 3.37 3.37 3.36 3.35 2.25 2.24 2.24 2.23 2.23 2.22 2.21 2.21 1.57 1.45 1.37 1.37 1.36 1.35 1.31 1.29 1.29 1.18 1.17 1.16 1.16 1.14 1.14 0.95 0.93 0.91 0.90
OEt
Me
NH^{t}Bu
O
anti-74
syn-74
1.82
3.72
1.75
3.65
1.92
3.78
3.80
1.90
1.00
1.25
1.26
16.87
1.04
5.91
2.94
2.53
f1 (ppm)

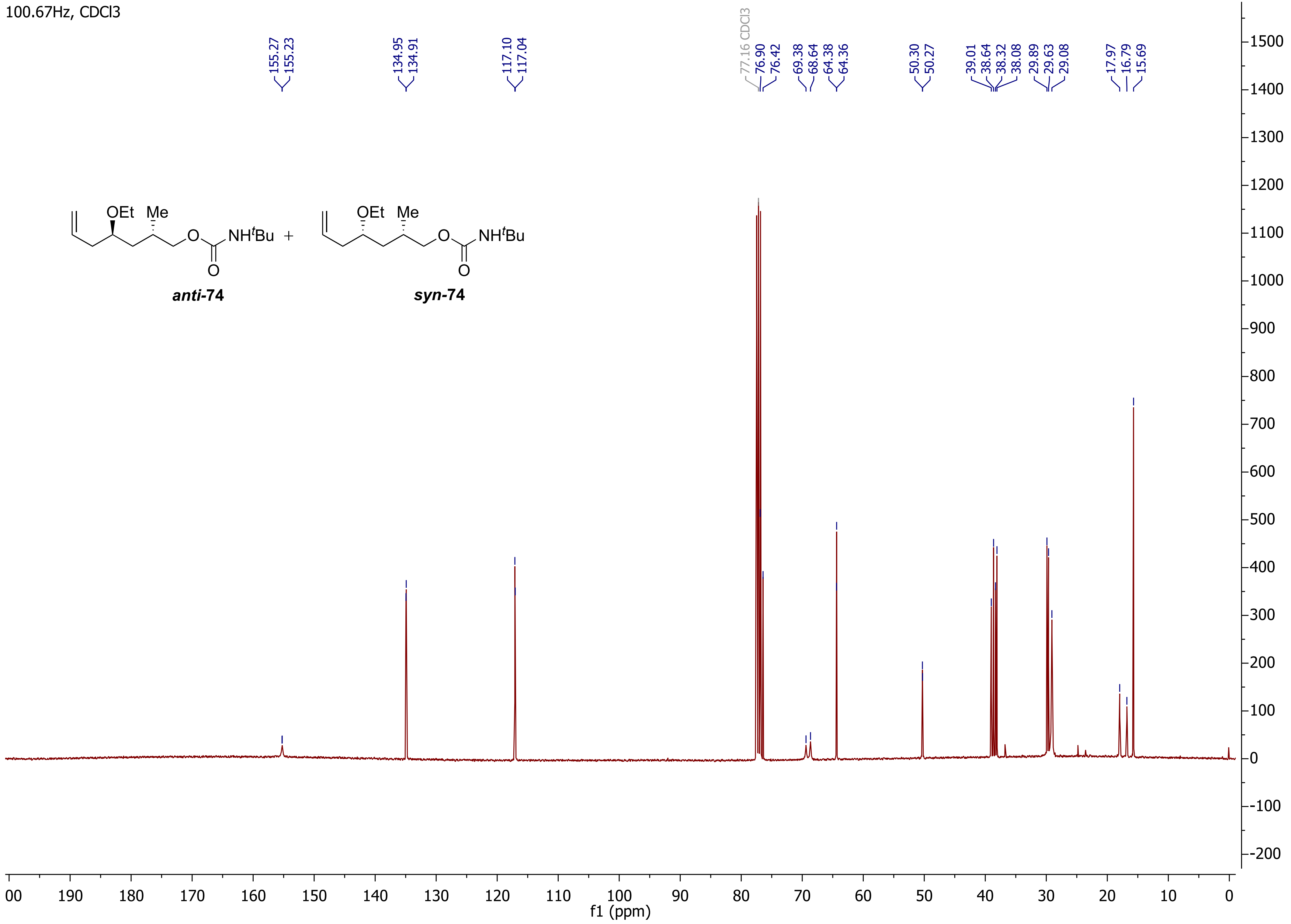

100.67Hz, CDCl3
155.27
155.23
134.95
134.91
117.10
117.04
77.16 CDCl3
76.90
76.42
69.38
68.64
64.38
64.36
50.30
50.27
39.01
38.64
38.32
38.08
29.89
29.63
29.08
17.97
16.79
15.69
OEt
Me
O
NH^{t}Bu
+
anti-74
syn-74
f1 (ppm)

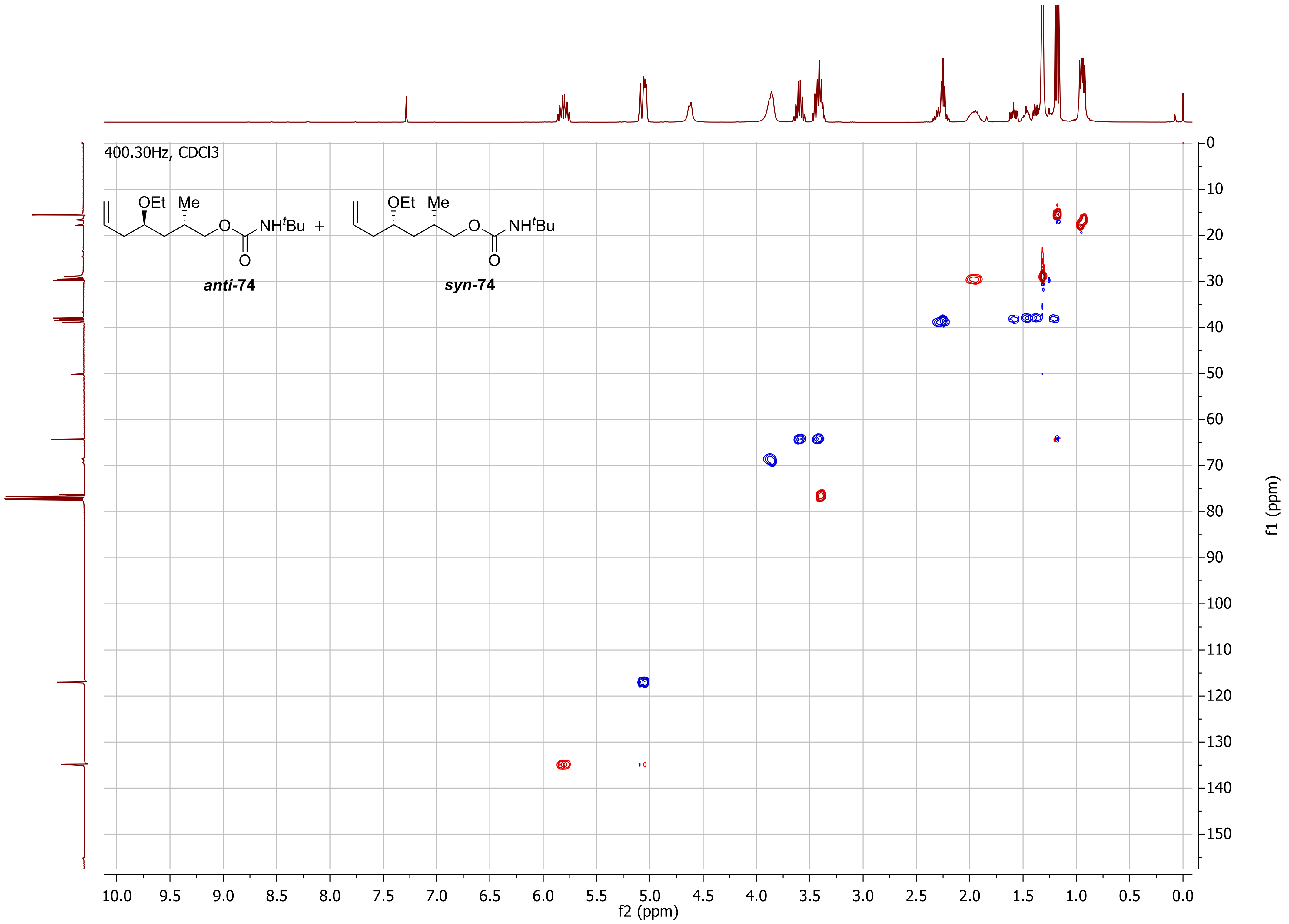

400.30Hz, CDCl3
OEt
Me
O
NH^{t}Bu
+
anti-74
syn-74
f2 (ppm)
f1 (ppm)

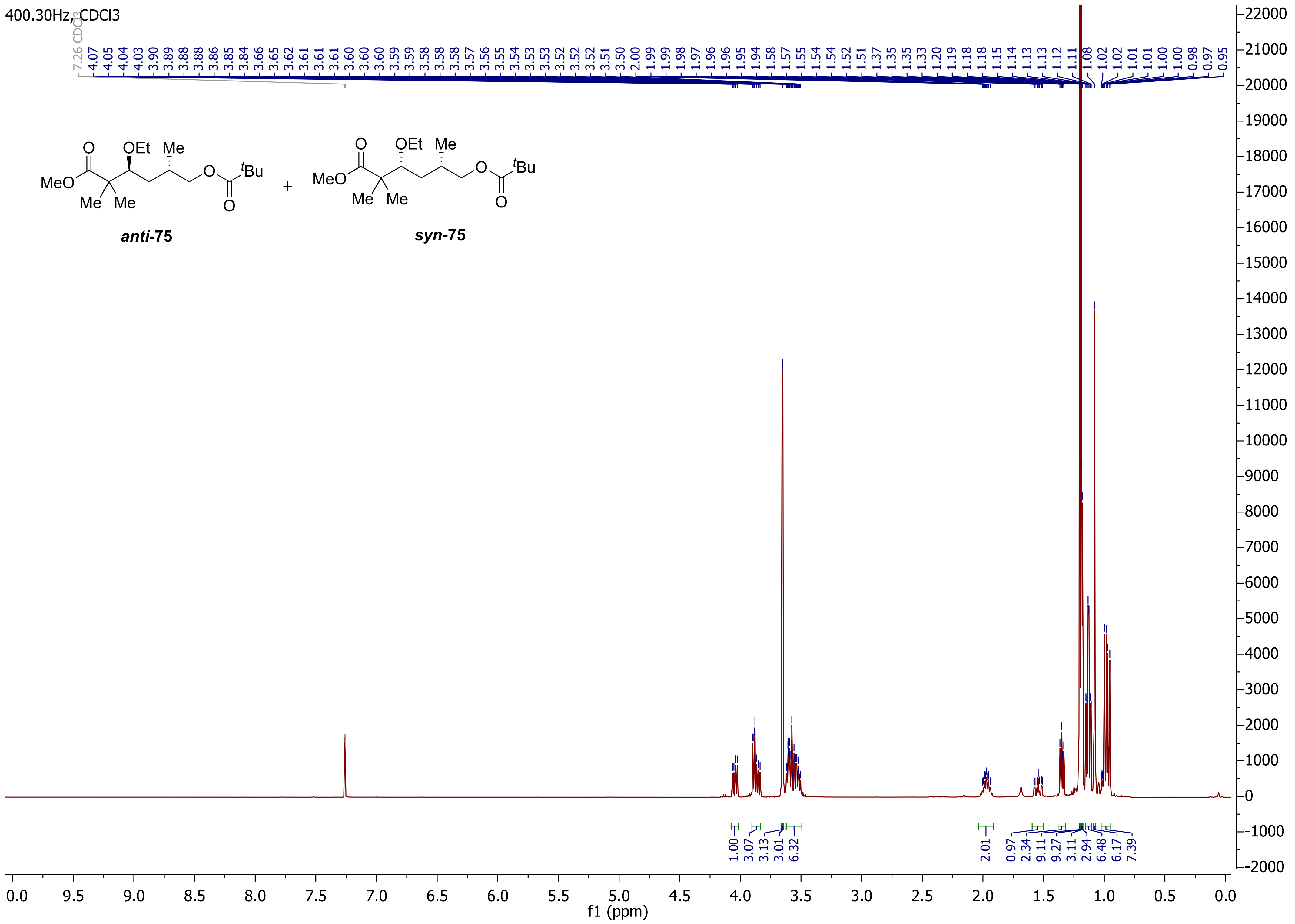
400.30Hz, CDCl3
anti-75
syn-75
f1 (ppm)

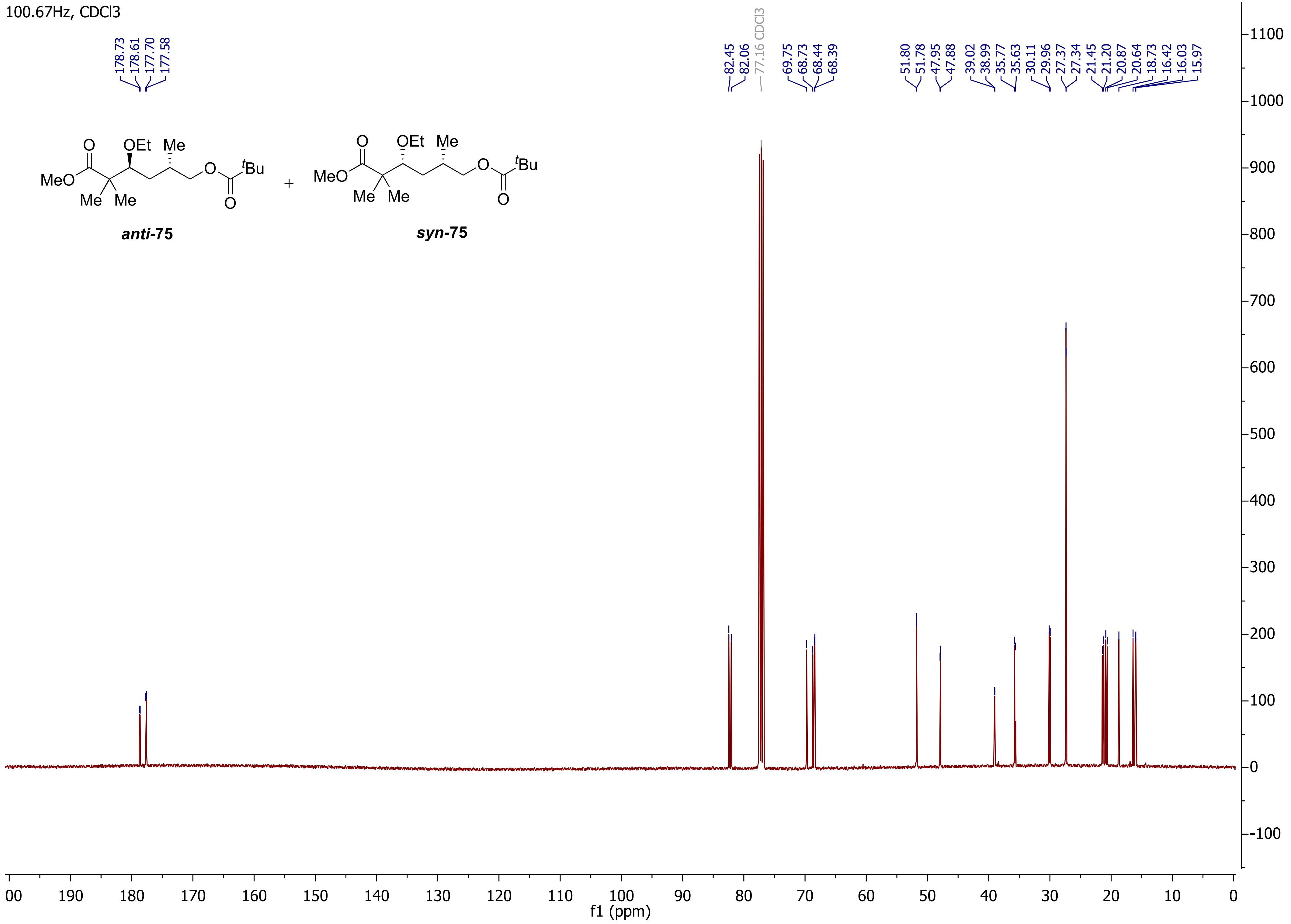
100.67Hz, CDCl3
178.73
178.61
177.70
177.58
82.45
82.06
77.16 CDCl3
69.75
68.73
68.44
68.39
51.80
51.78
47.95
47.88
39.02
38.99
35.77
35.63
30.11
29.96
27.37
27.34
21.45
21.20
20.87
20.64
18.73
16.42
16.03
15.97
anti-75
syn-75
f1 (ppm)

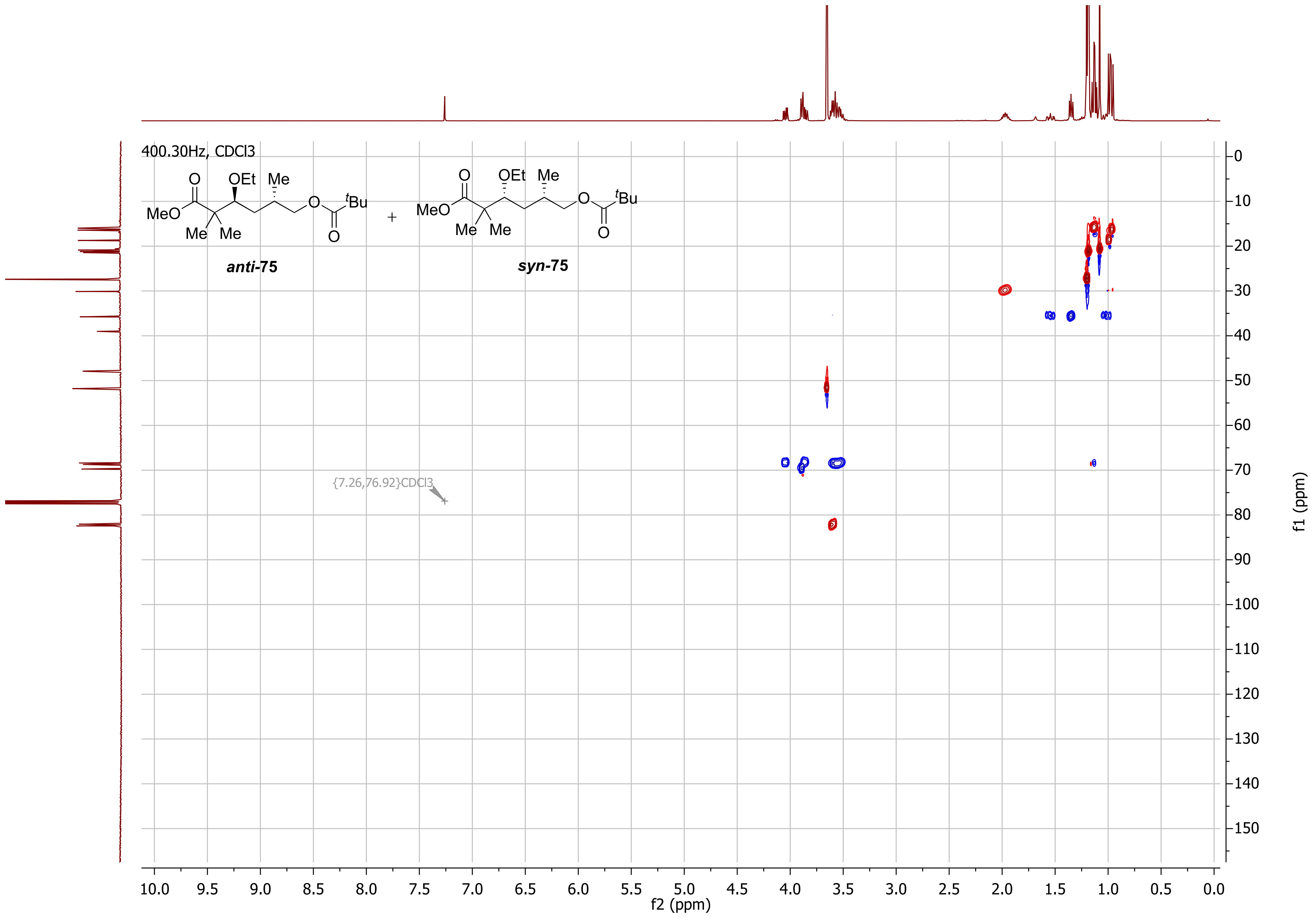

400.30Hz, CDCl3
MeO
OEt
Me
tBu
Me
O
anti-75
syn-75
{7.26,76.92}CDCl3
f2 (ppm)
f1 (ppm)

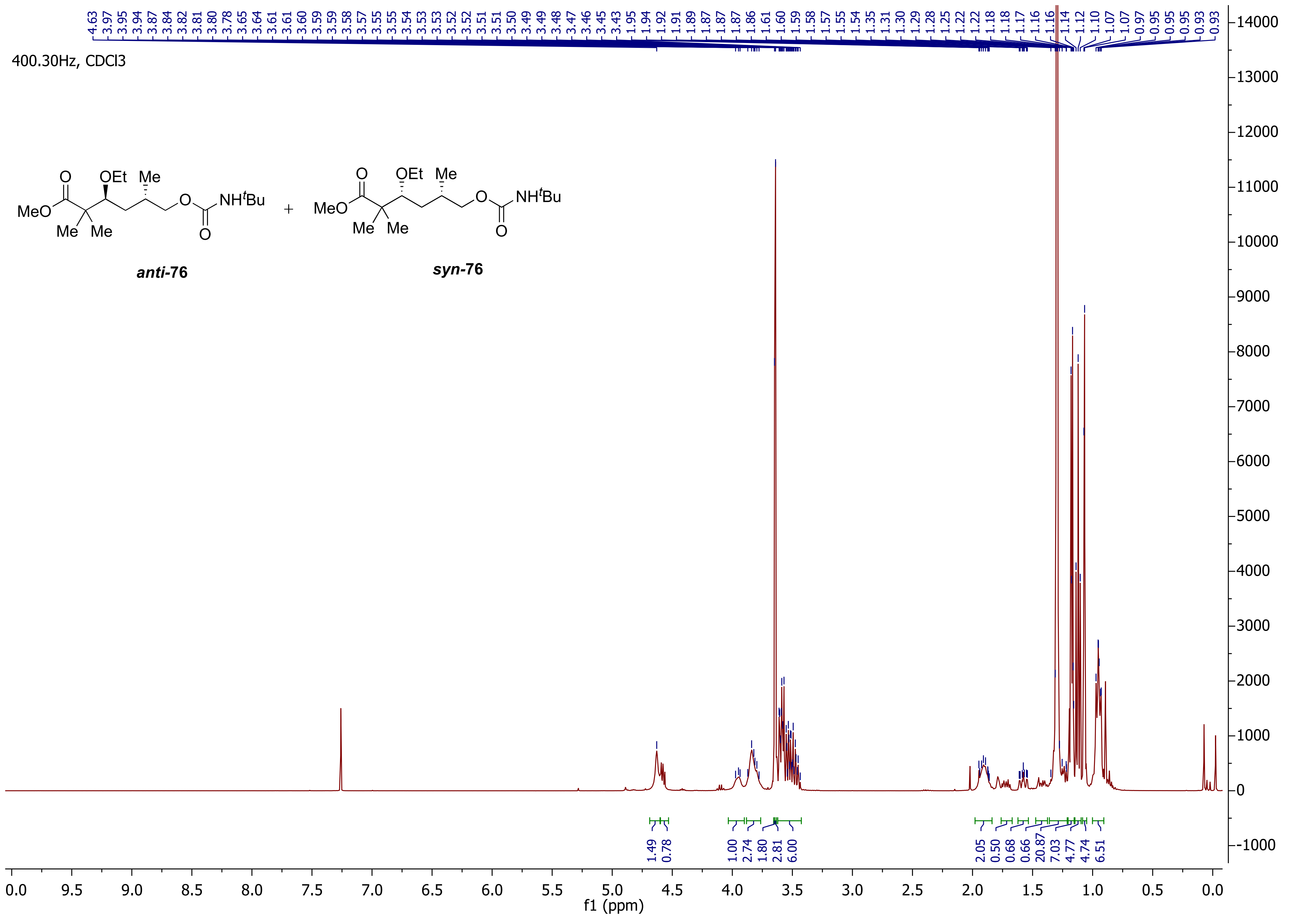

400.30Hz, CDCl3
anti-76
syn-76
OEt
Me
MeO
O
NH^{t}Bu
4.63 3.97 3.95 3.94 3.87 3.84 3.82 3.81 3.80 3.78 3.65 3.64 3.61 3.61 3.60 3.59 3.59 3.58 3.57 3.55 3.55 3.54 3.53 3.53 3.52 3.52 3.51 3.51 3.50 3.49 3.49 3.48 3.47 3.46 3.45 3.43 1.95 1.94 1.92 1.91 1.89 1.87 1.87 1.87 1.86 1.61 1.60 1.59 1.58 1.57 1.55 1.54 1.35 1.31 1.30 1.29 1.28 1.25 1.22 1.22 1.18 1.18 1.17 1.16 1.16 1.14 1.12 1.10 1.07 1.07 0.97 0.95 0.95 0.93 0.93
1.49 0.78 1.00 2.74 1.80 2.81 6.00 2.05 0.50 0.68 0.66 20.87 7.03 4.77 4.74 6.51
f1 (ppm)

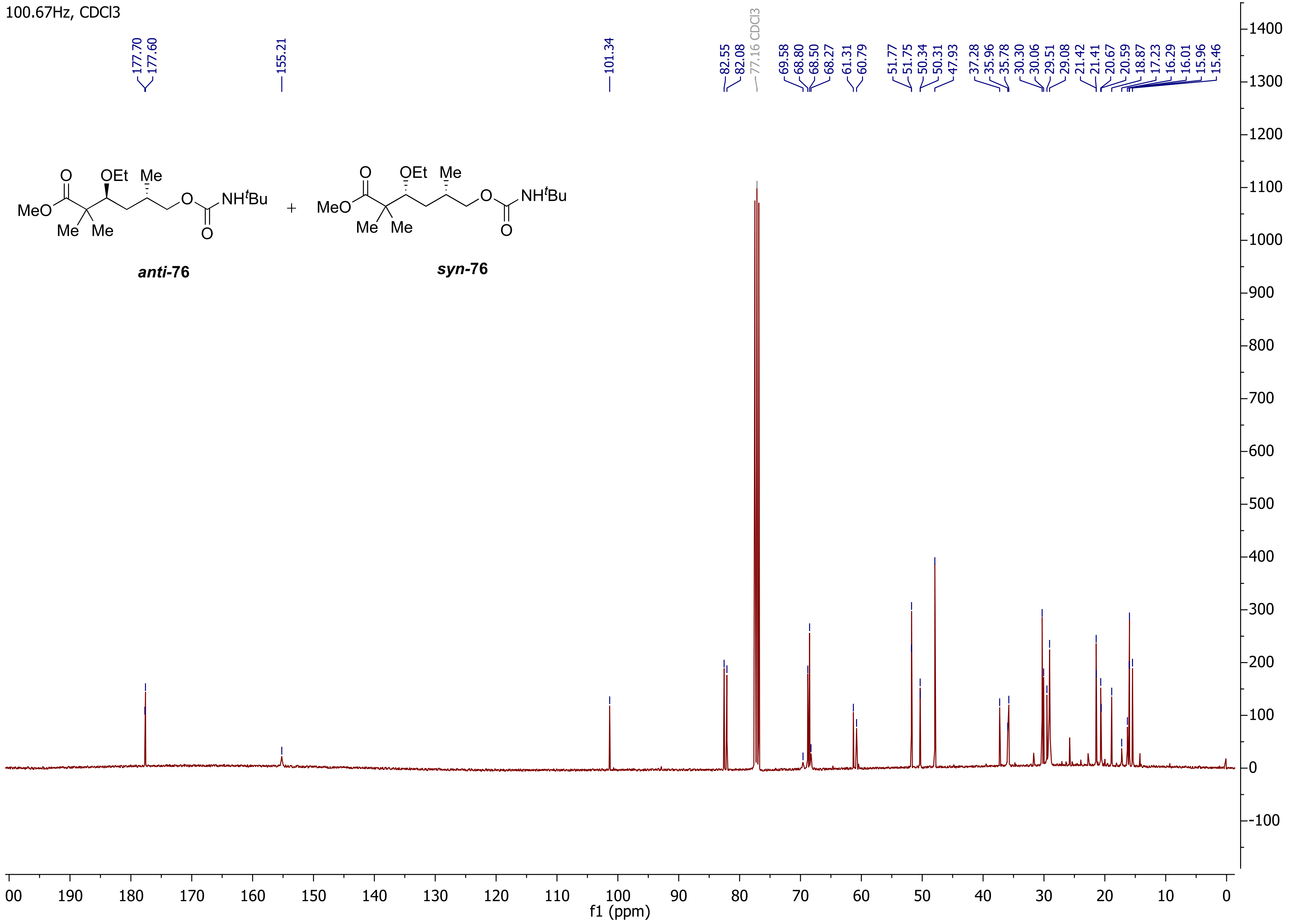

100.67Hz, CDCl3
177.70
177.60
155.21
101.34
82.55
82.08
77.16 CDCl3
69.58
68.80
68.50
68.27
61.31
60.79
51.77
51.75
50.34
50.31
47.93
37.28
35.96
35.78
30.30
30.06
29.51
29.08
21.42
21.41
20.67
20.59
18.87
17.23
16.29
16.01
15.96
15.46
anti-76
syn-76
f1 (ppm)

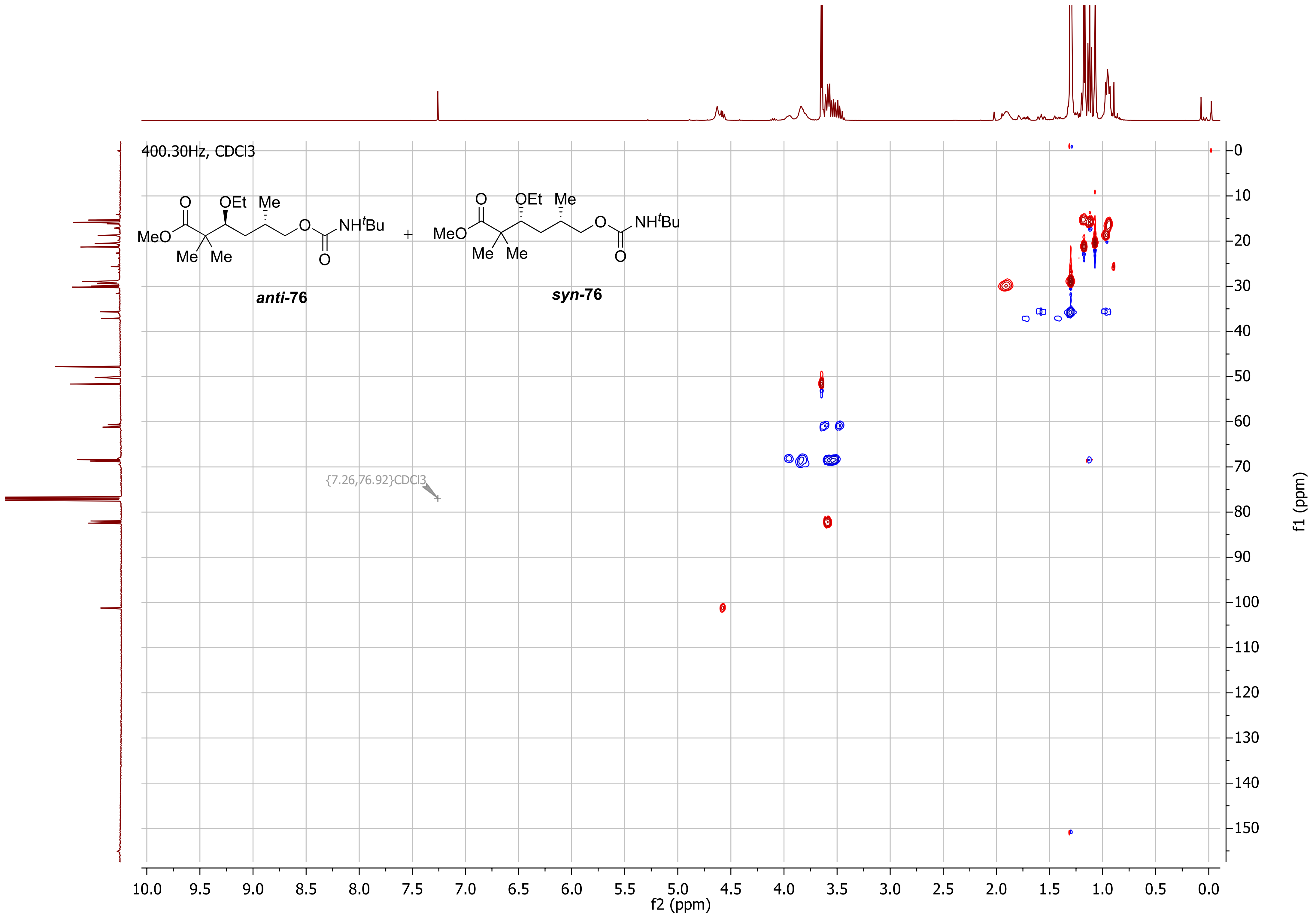
400.30Hz, CDCl3
MeO
O
OEt
Me
O
NH^{t}Bu
O
Me
Me
+
anti-76
syn-76
{7.26,76.92}CDCl3
f2 (ppm)
f1 (ppm)

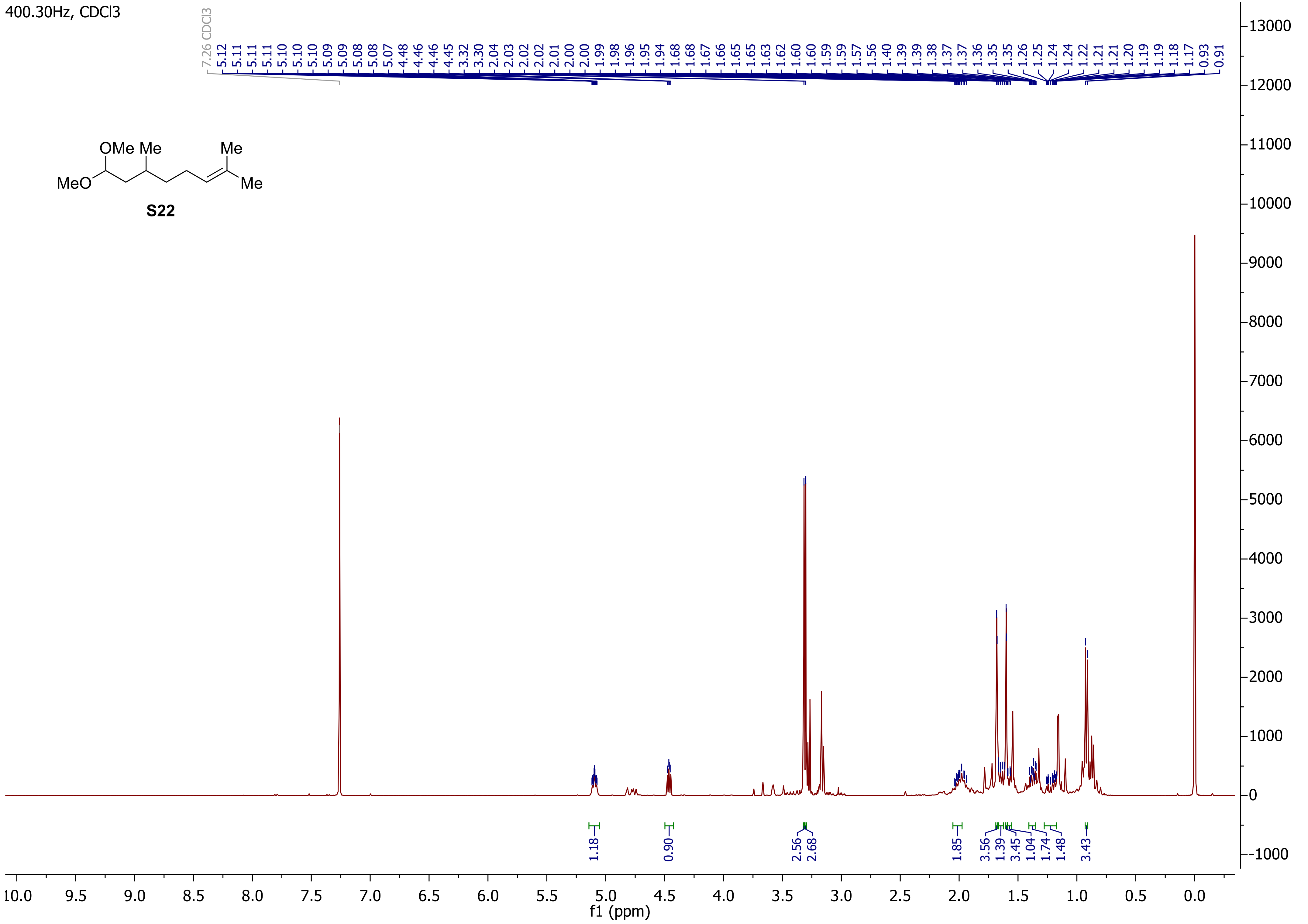

400.30Hz, CDCl3
OMe Me
Me
MeO
Me
S22
f1 (ppm)

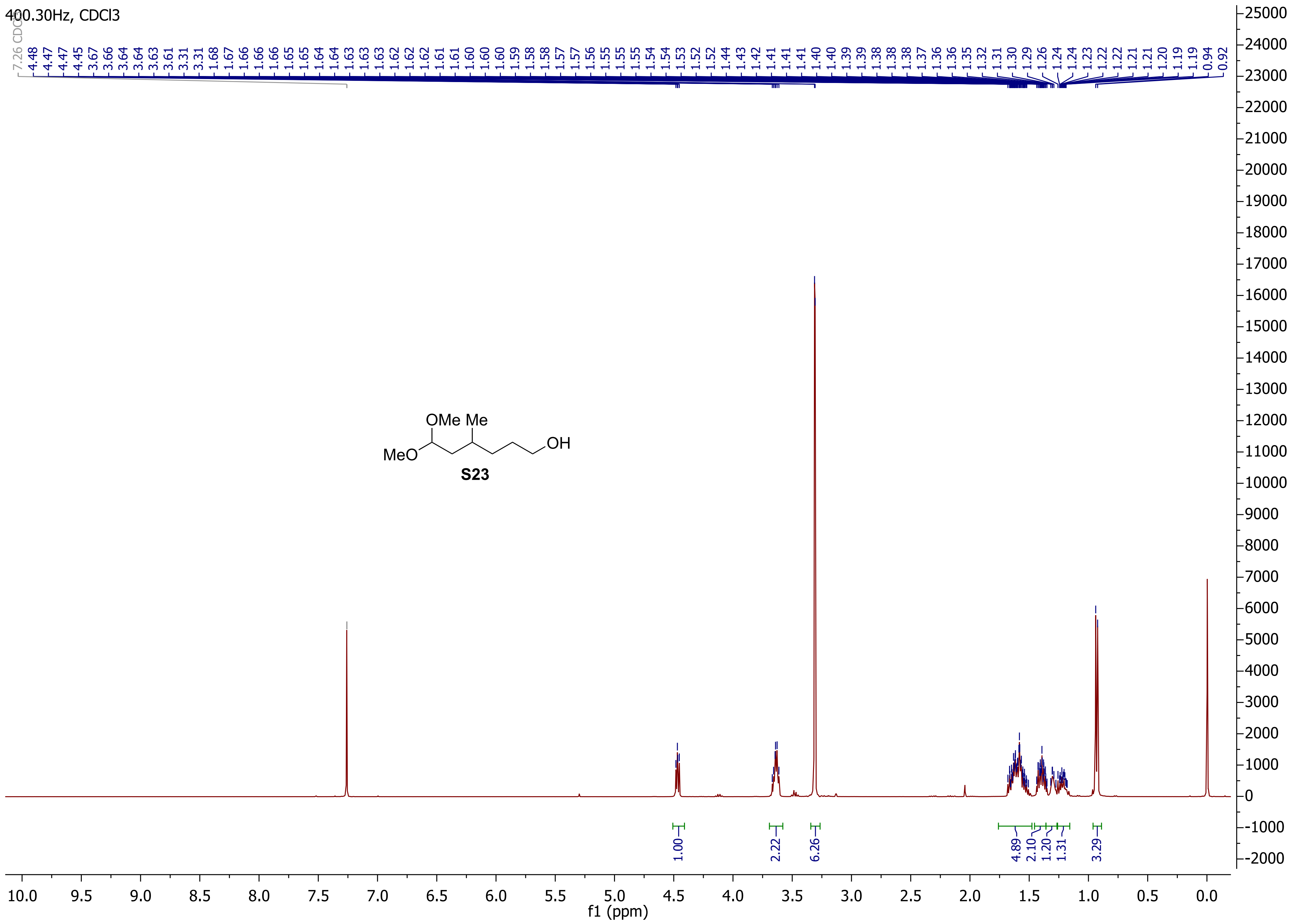

400.30Hz, CDCl3
7.26 CDCl3
4.48
4.47
4.47
4.45
3.67
3.66
3.64
3.64
3.63
3.61
3.31
3.31
1.68
1.67
1.66
1.66
1.66
1.65
1.65
1.64
1.64
1.63
1.63
1.63
1.62
1.62
1.62
1.61
1.61
1.60
1.60
1.60
1.59
1.58
1.58
1.57
1.57
1.56
1.55
1.55
1.55
1.54
1.54
1.53
1.52
1.52
1.44
1.43
1.42
1.41
1.41
1.41
1.40
1.40
1.39
1.39
1.38
1.38
1.38
1.37
1.36
1.36
1.35
1.32
1.31
1.30
1.29
1.26
1.24
1.24
1.23
1.22
1.22
1.21
1.21
1.20
1.19
1.19
0.94
0.92
OMe Me
MeO
OH
S23
1.00
2.22
6.26
4.89
2.10
1.20
1.31
3.29
f1 (ppm)

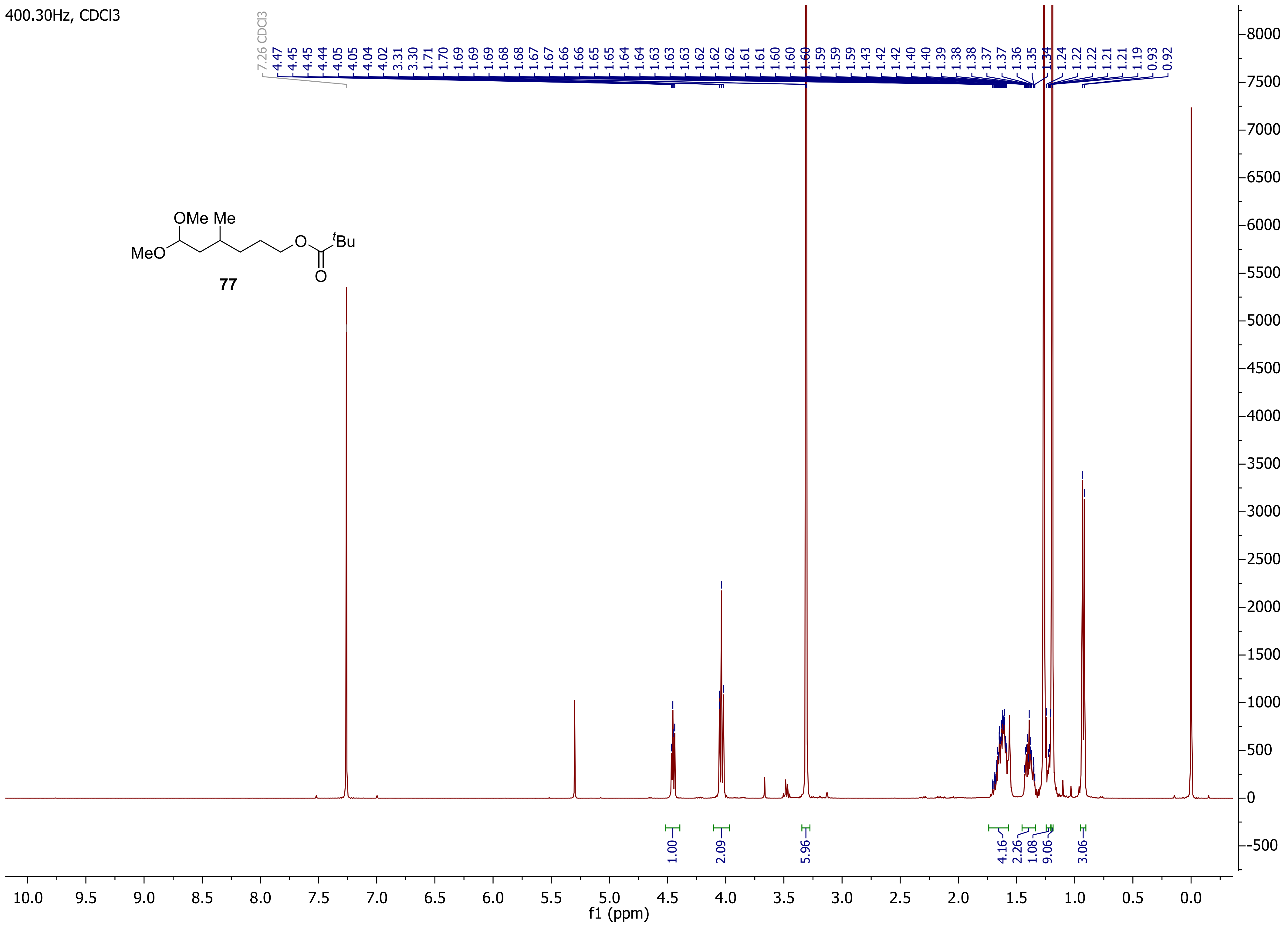

400.30Hz, CDCl3
7.26 CDCl3
4.47 4.45 4.45 4.44 4.05 4.05 4.04 4.02 3.31 3.30 1.71 1.70 1.69 1.69 1.69 1.68 1.68 1.67 1.67 1.66 1.66 1.65 1.65 1.64 1.64 1.63 1.63 1.63 1.62 1.62 1.62 1.61 1.61 1.60 1.60 1.60 1.59 1.59 1.59 1.43 1.42 1.42 1.40 1.40 1.39 1.38 1.38 1.37 1.37 1.36 1.35 1.34 1.24 1.22 1.22 1.21 1.21 1.19 0.93 0.92
OMe Me
MeO
O
tBu
O
77
1.00
2.09
5.96
4.16
2.26
1.08
9.06
3.06
f1 (ppm)

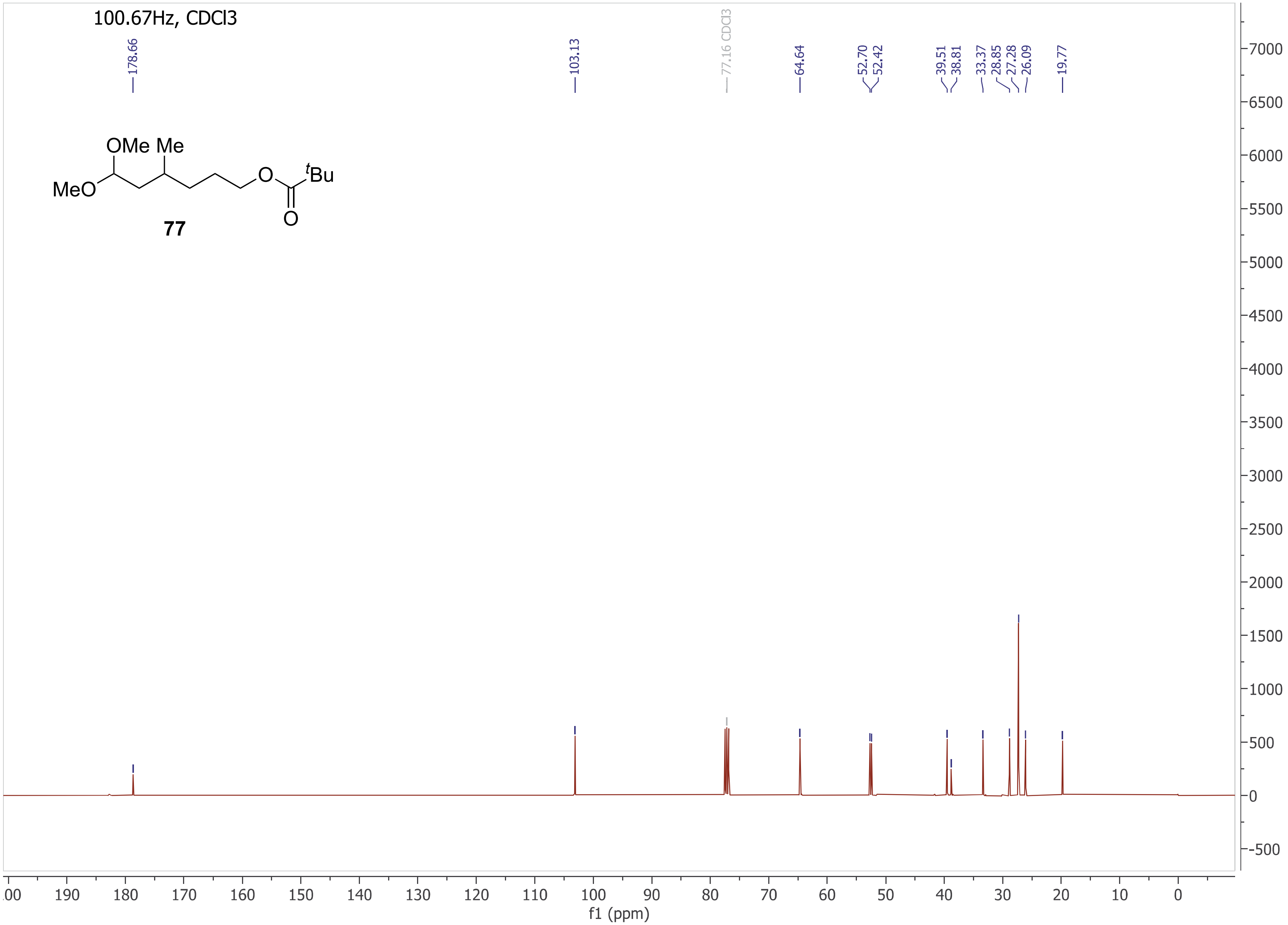
100.67Hz, CDCl3
178.66
103.13
77.16 CDCl3
64.64
52.70
52.42
39.51
38.81
33.37
28.85
27.28
26.09
19.77
OMe Me
MeO
O
tBu
O
77
f1 (ppm)

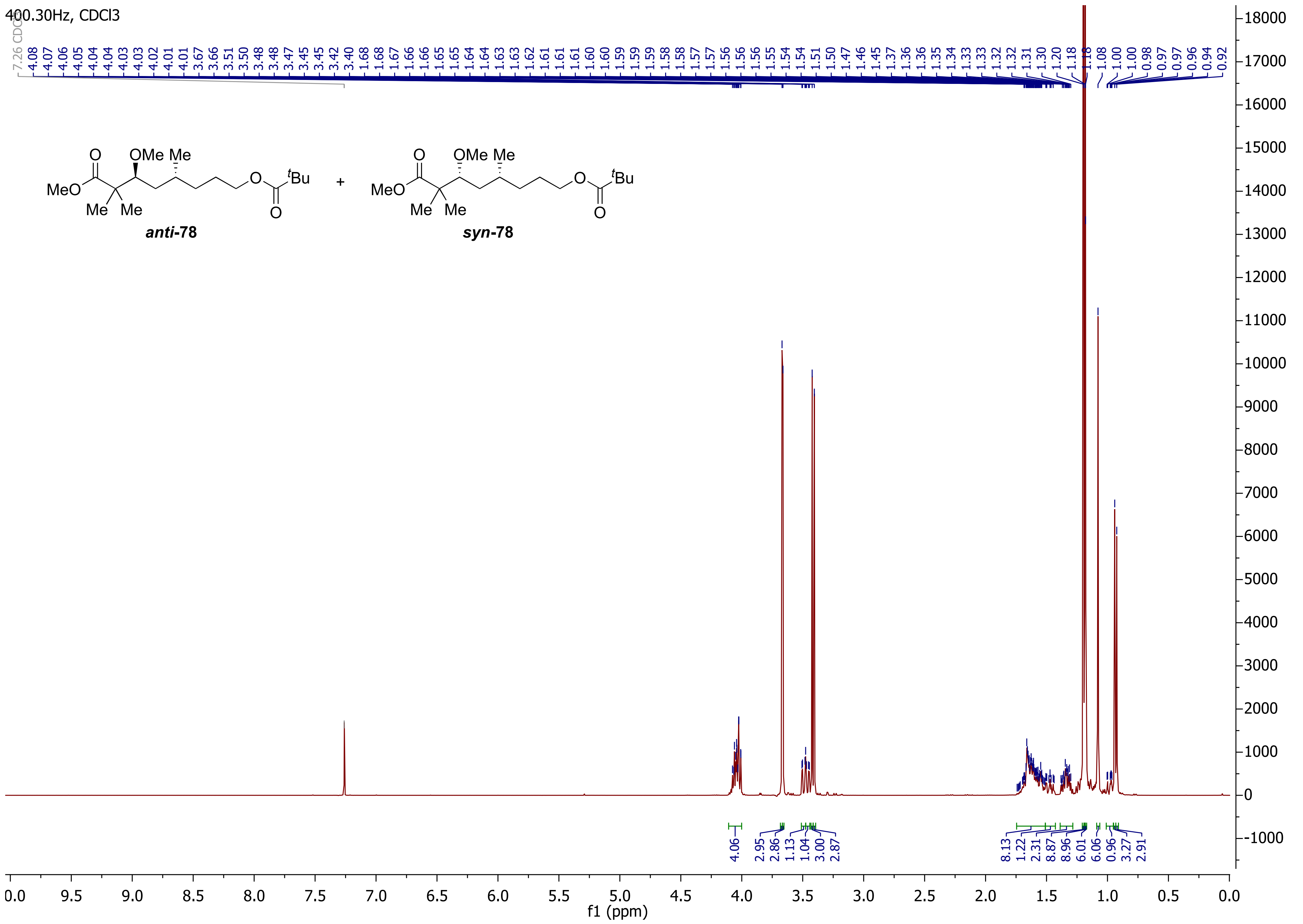

400.30Hz, CDCl3
anti-78
syn-78
f1 (ppm)

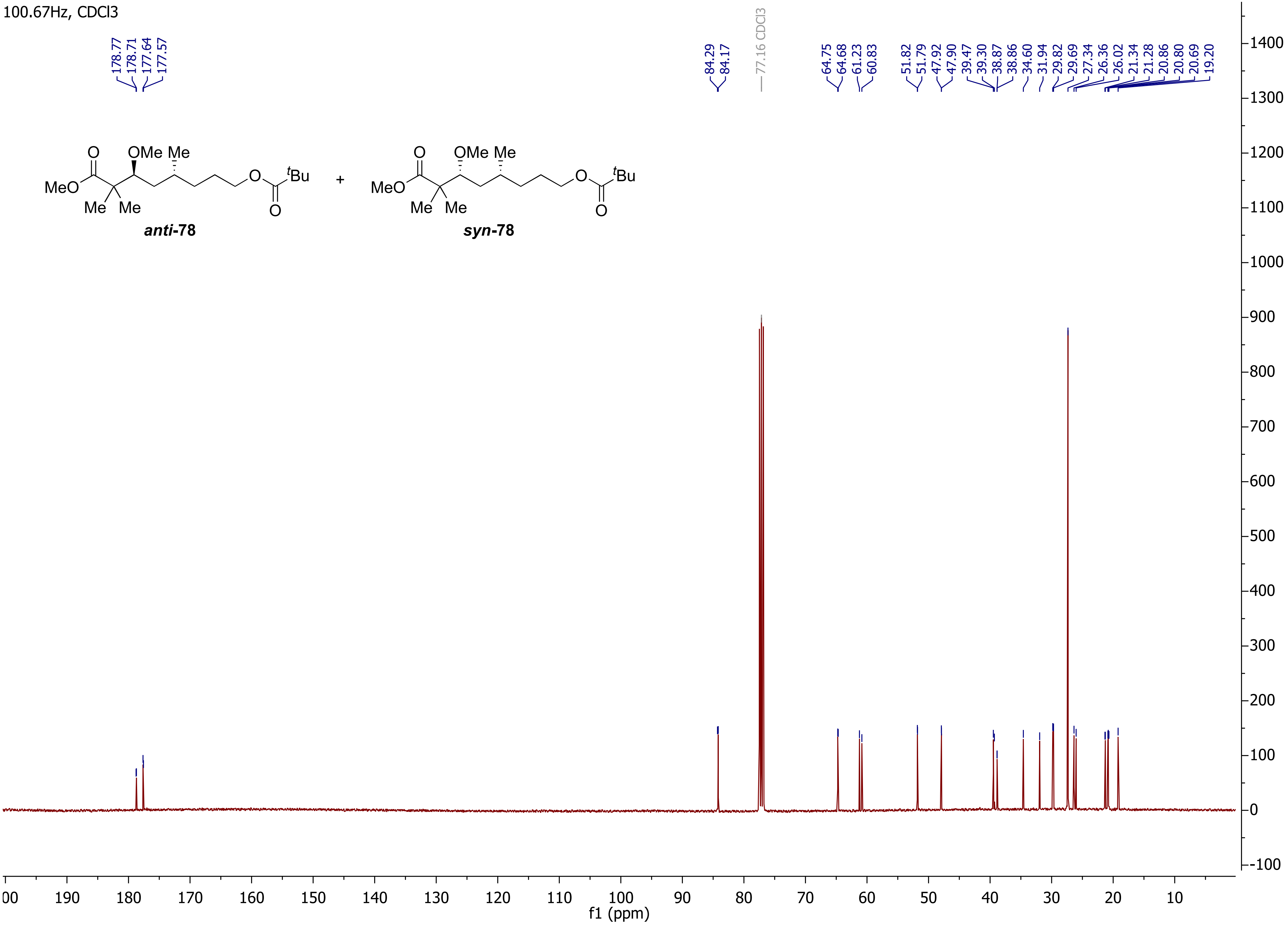
100.67Hz, CDCl3
178.77
178.71
177.64
177.57
84.29
84.17
77.16 CDCl3
64.75
64.68
61.23
60.83
51.82
51.79
47.92
47.90
39.47
39.30
38.87
38.86
34.60
31.94
29.82
29.69
27.34
26.36
26.02
21.34
21.28
20.86
20.80
20.69
19.20
anti-78
syn-78
f1 (ppm)

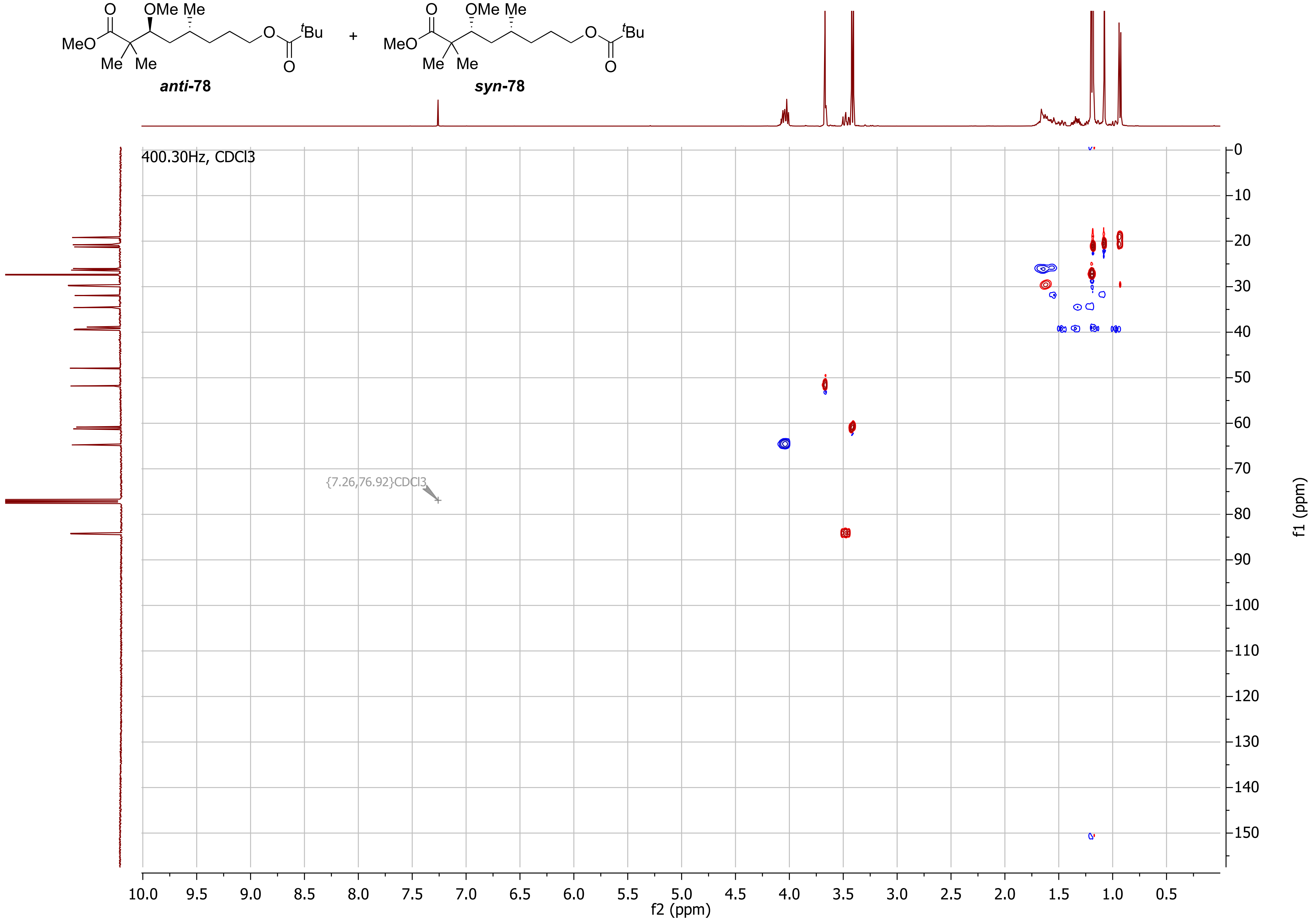

anti-78
syn-78
400.30Hz, CDCl3
{7.26,76.92}CDCl3
f2 (ppm)
f1 (ppm)

400.30Hz, CDCl3

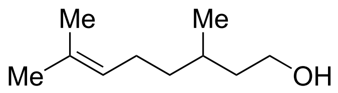

S24

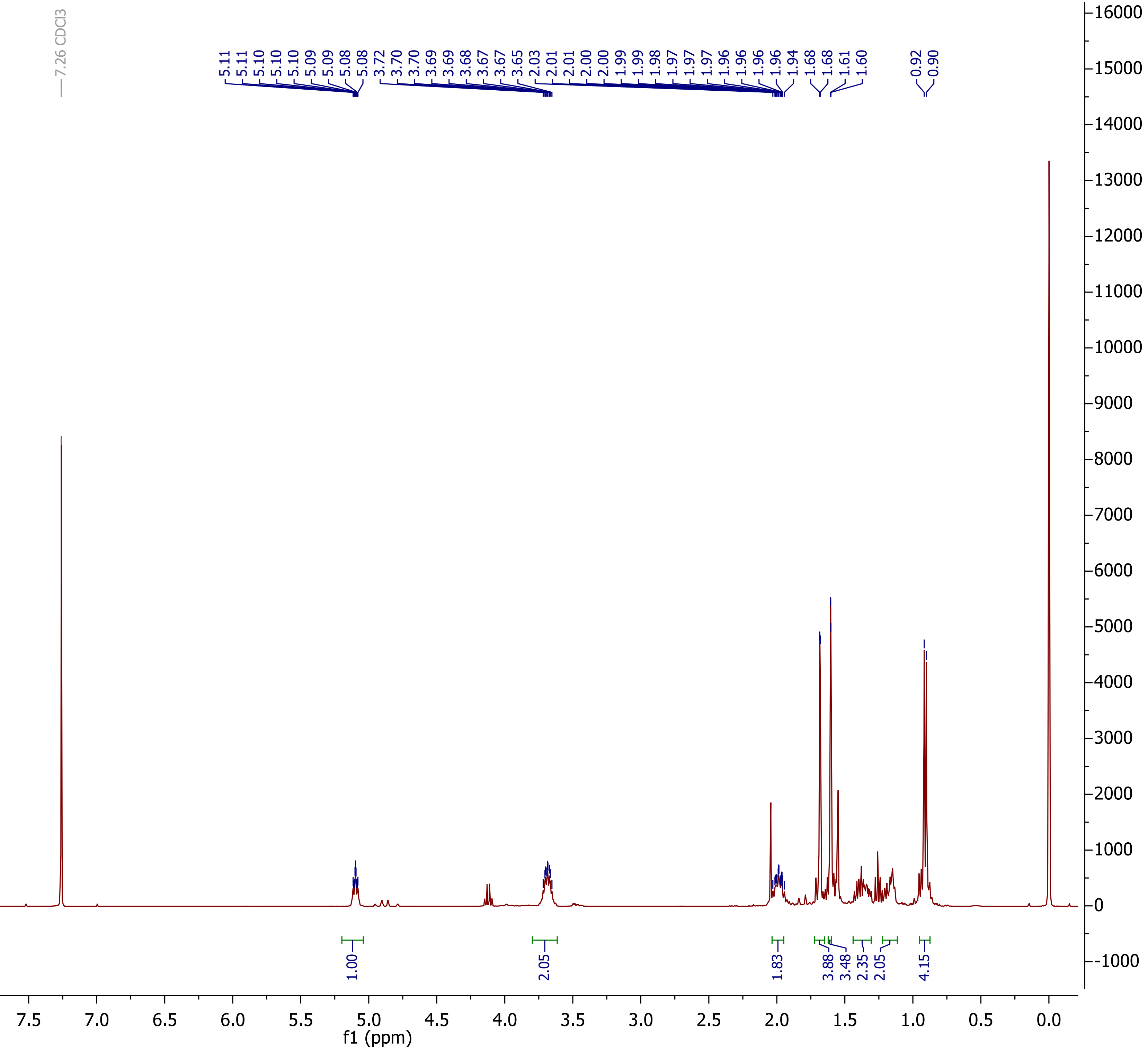

400.30Hz, CDCl3

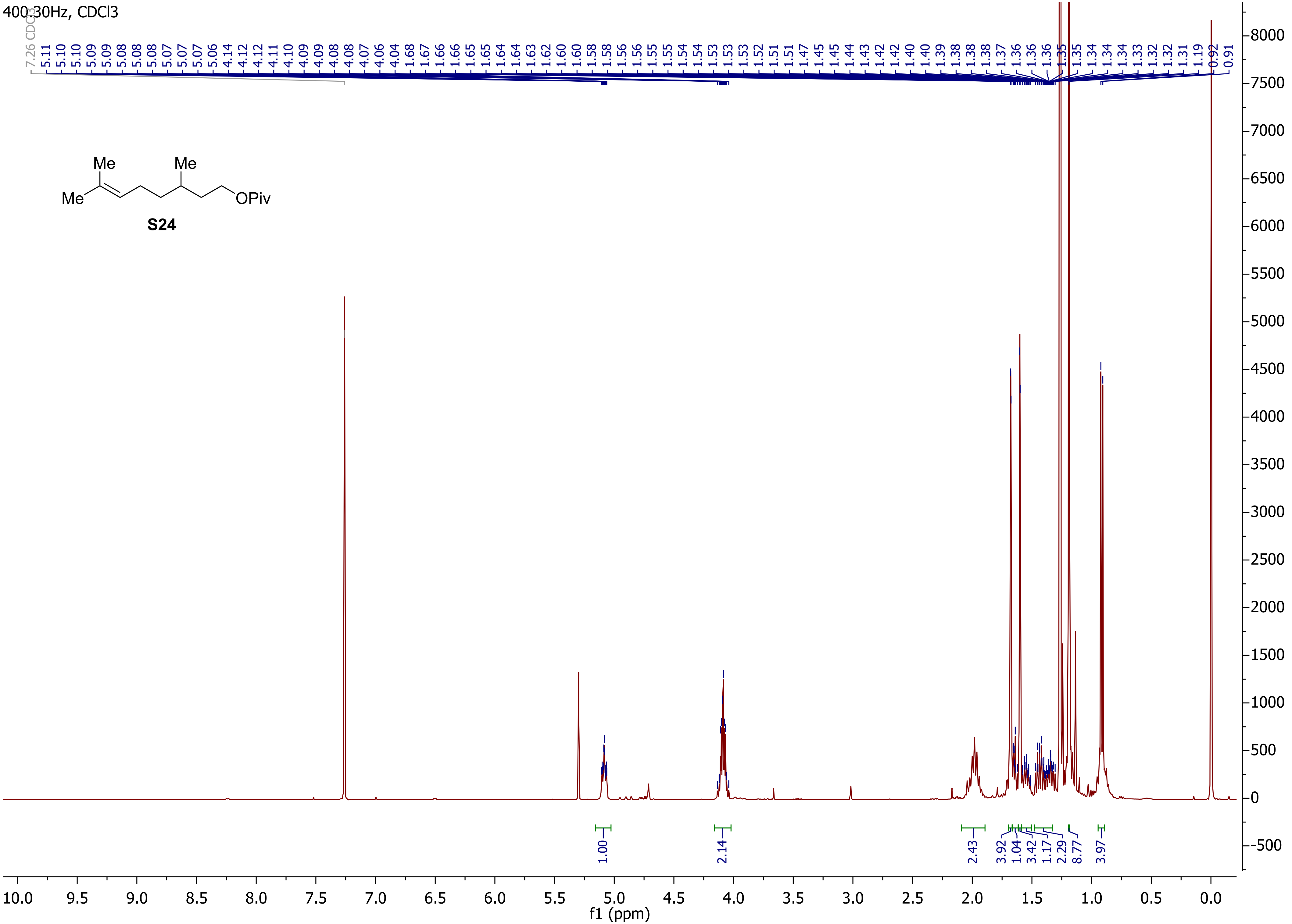

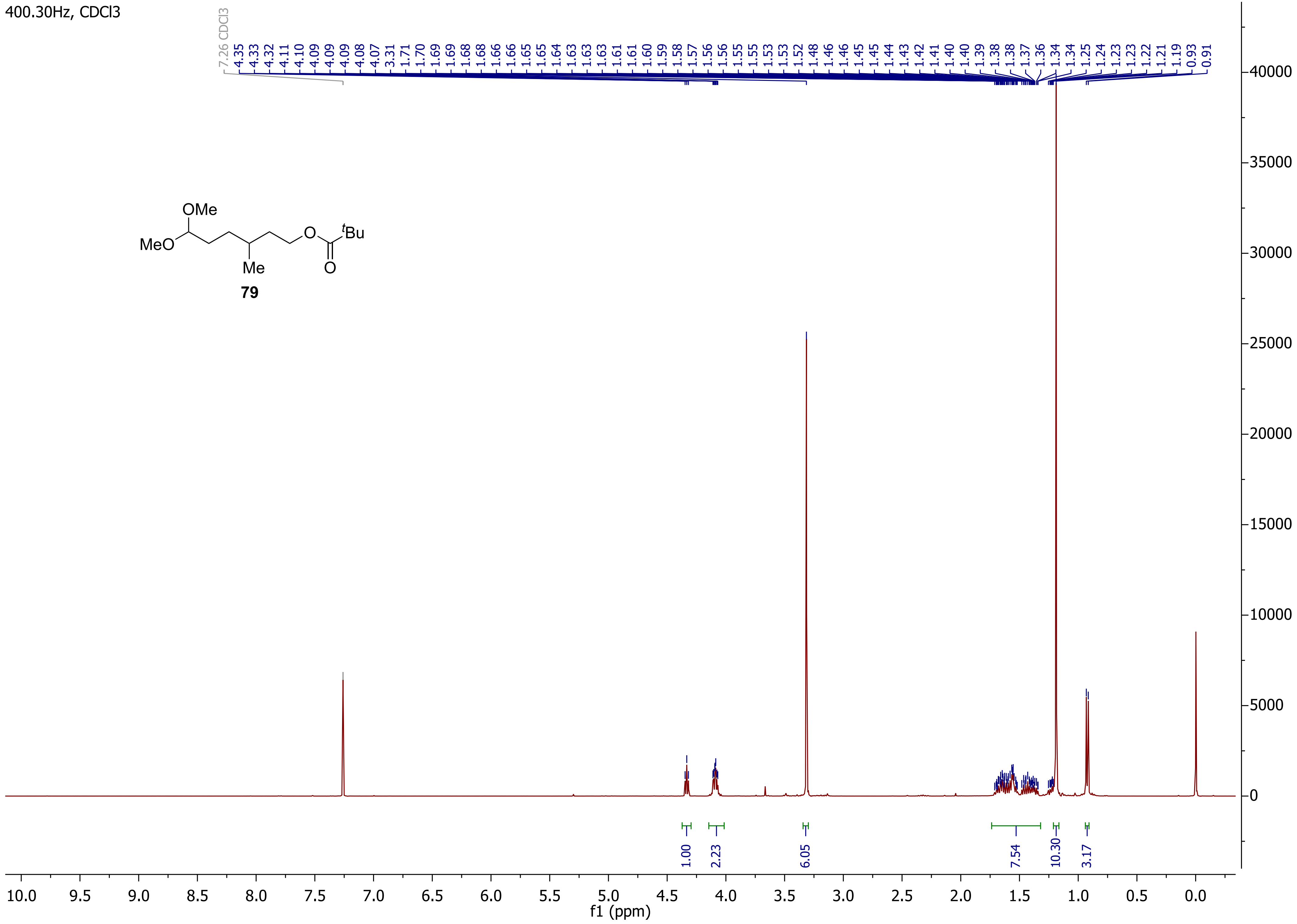
400.30Hz, CDCl3
7.26 CDCl3
4.35 4.33 4.32 4.11 4.10 4.09 4.09 4.09 4.08 4.07 3.31 1.71 1.70 1.69 1.69 1.68 1.68 1.66 1.66 1.65 1.65 1.64 1.63 1.63 1.63 1.61 1.61 1.60 1.59 1.58 1.57 1.56 1.56 1.55 1.55 1.53 1.53 1.52 1.48 1.46 1.46 1.45 1.45 1.44 1.43 1.42 1.41 1.40 1.40 1.39 1.38 1.38 1.37 1.36 1.34 1.34 1.25 1.24 1.23 1.23 1.22 1.21 1.19 0.93 0.91
OMe
MeO
O
tBu
Me
O
79
1.00
2.23
6.05
7.54
10.30
3.17
f1 (ppm)

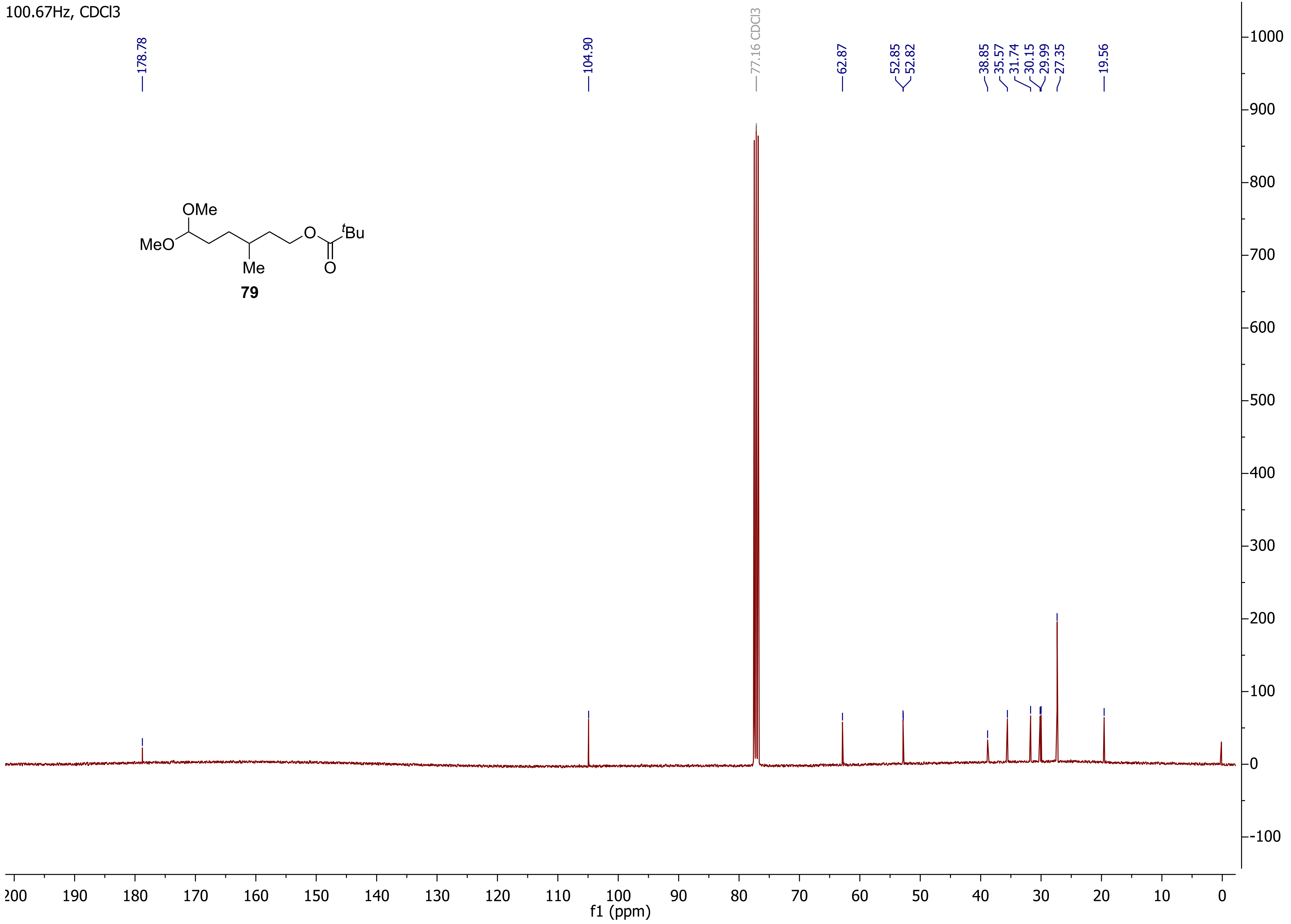
100.67Hz, CDCl3
178.78
104.90
77.16 CDCl3
62.87
52.85
52.82
38.85
35.57
31.74
30.15
29.99
27.35
19.56
OMe
MeO
O
tBu
Me
O
79
f1 (ppm)

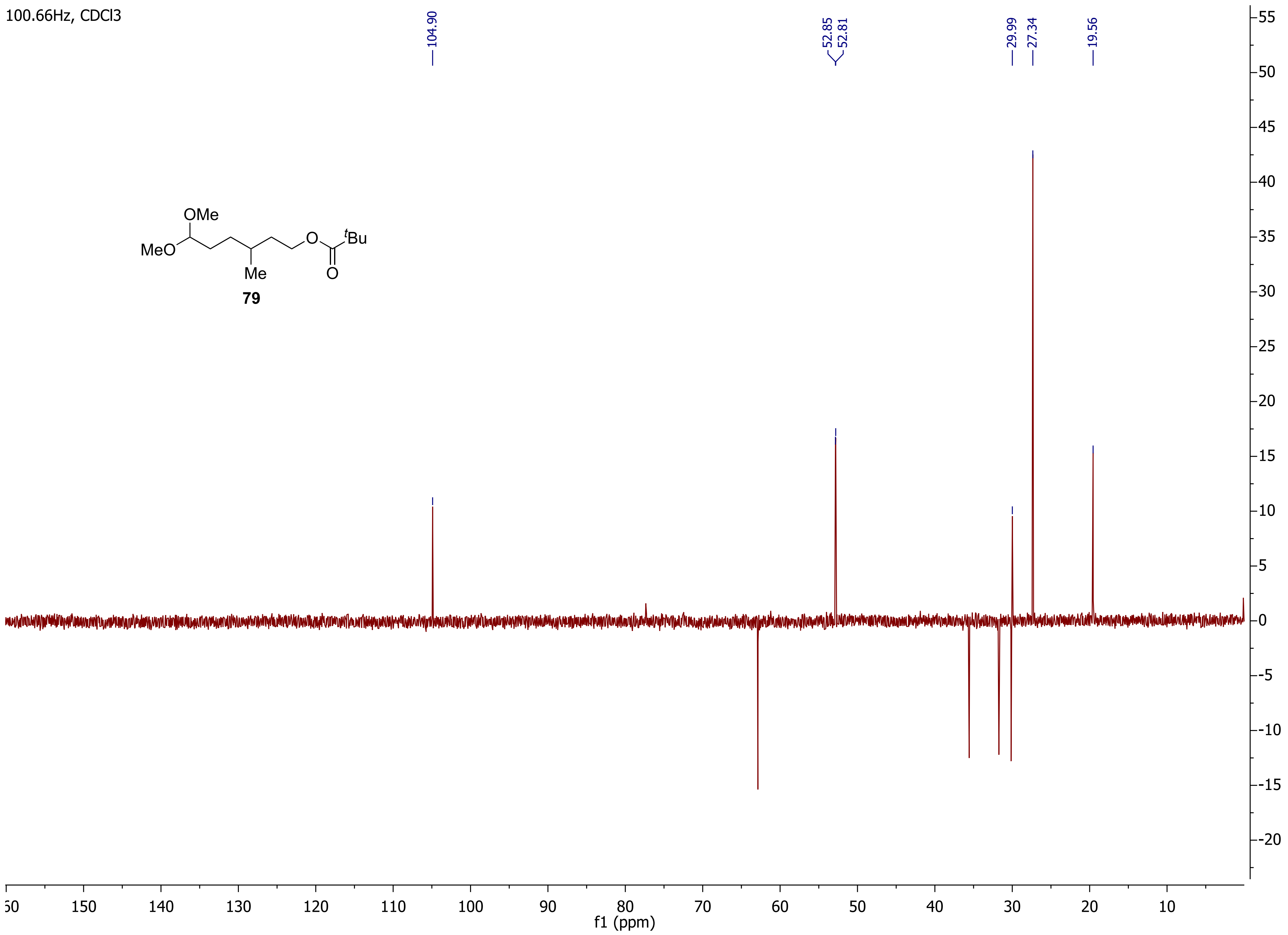
100.66Hz, CDCl3
104.90
52.85
52.81
29.99
27.34
19.56
OMe
MeO
Me
O
tBu
O
79
f1 (ppm)

400.30Hz, CDCl3

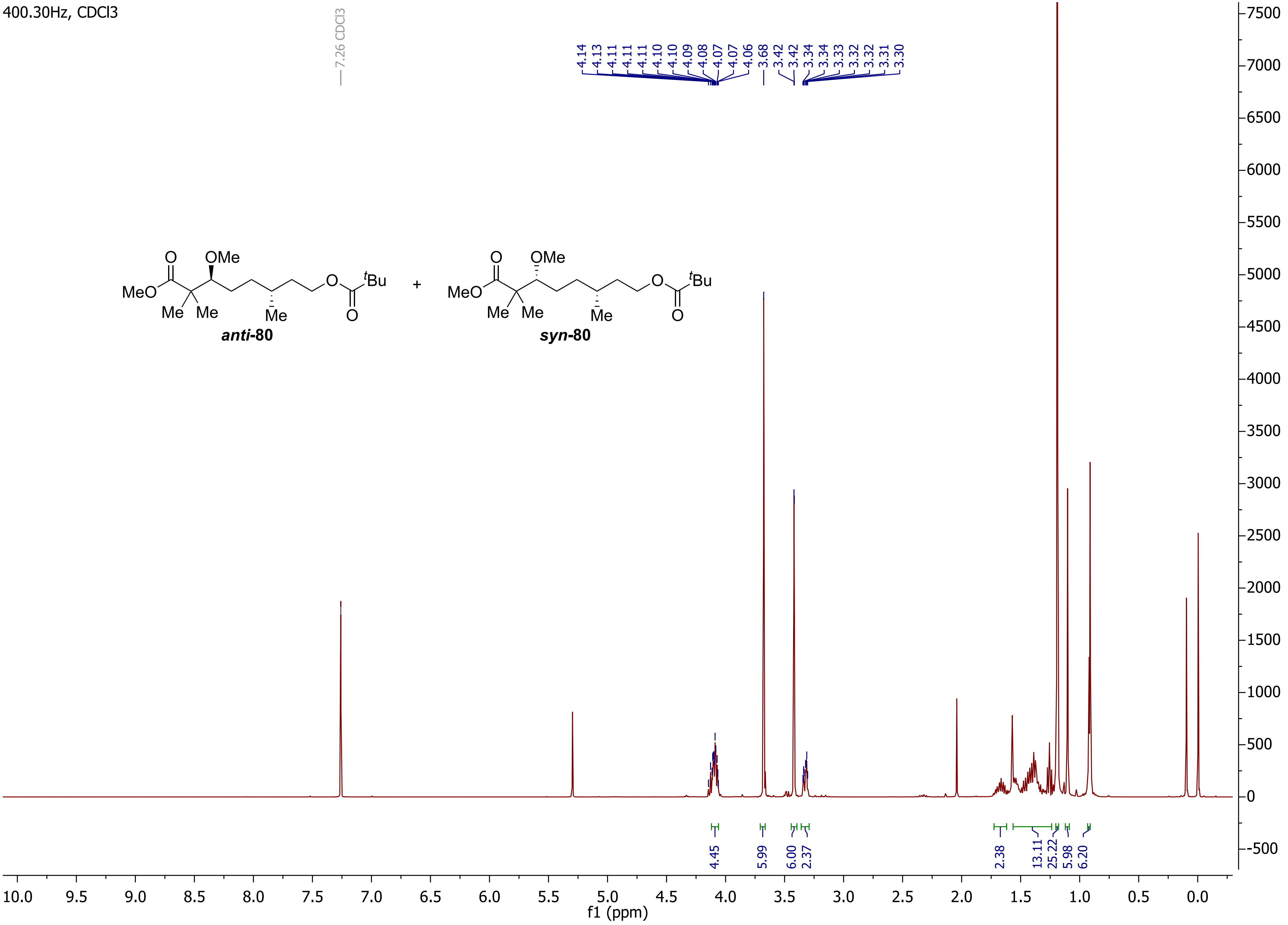

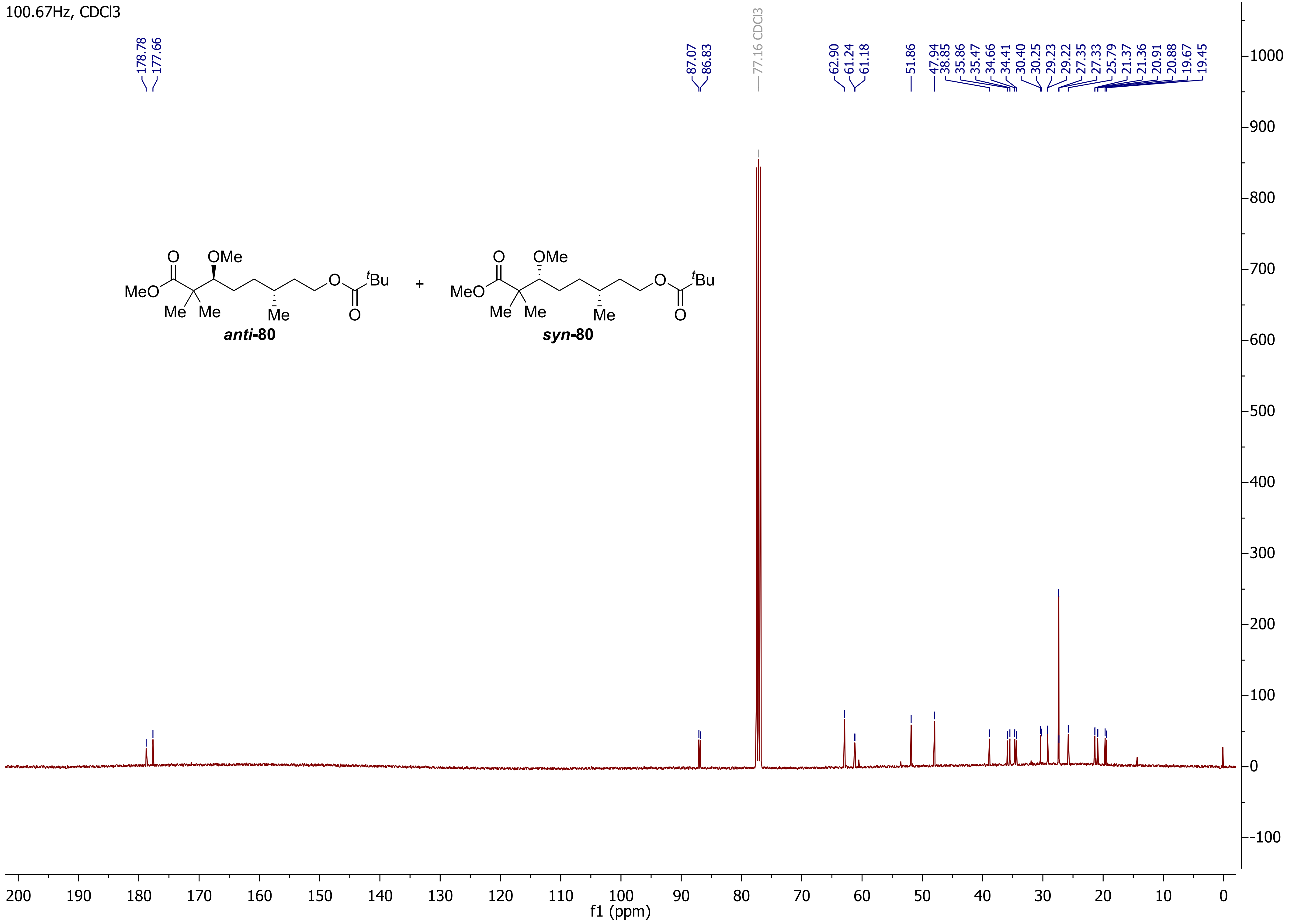

100.67Hz, CDCl3
178.78
177.66
87.07
86.83
77.16 CDCl3
62.90
61.24
61.18
51.86
47.94
38.85
35.86
35.47
34.66
34.41
30.40
30.25
29.23
29.22
27.35
27.33
25.79
21.37
21.36
20.91
20.88
19.67
19.45
anti-80
syn-80
f1 (ppm)

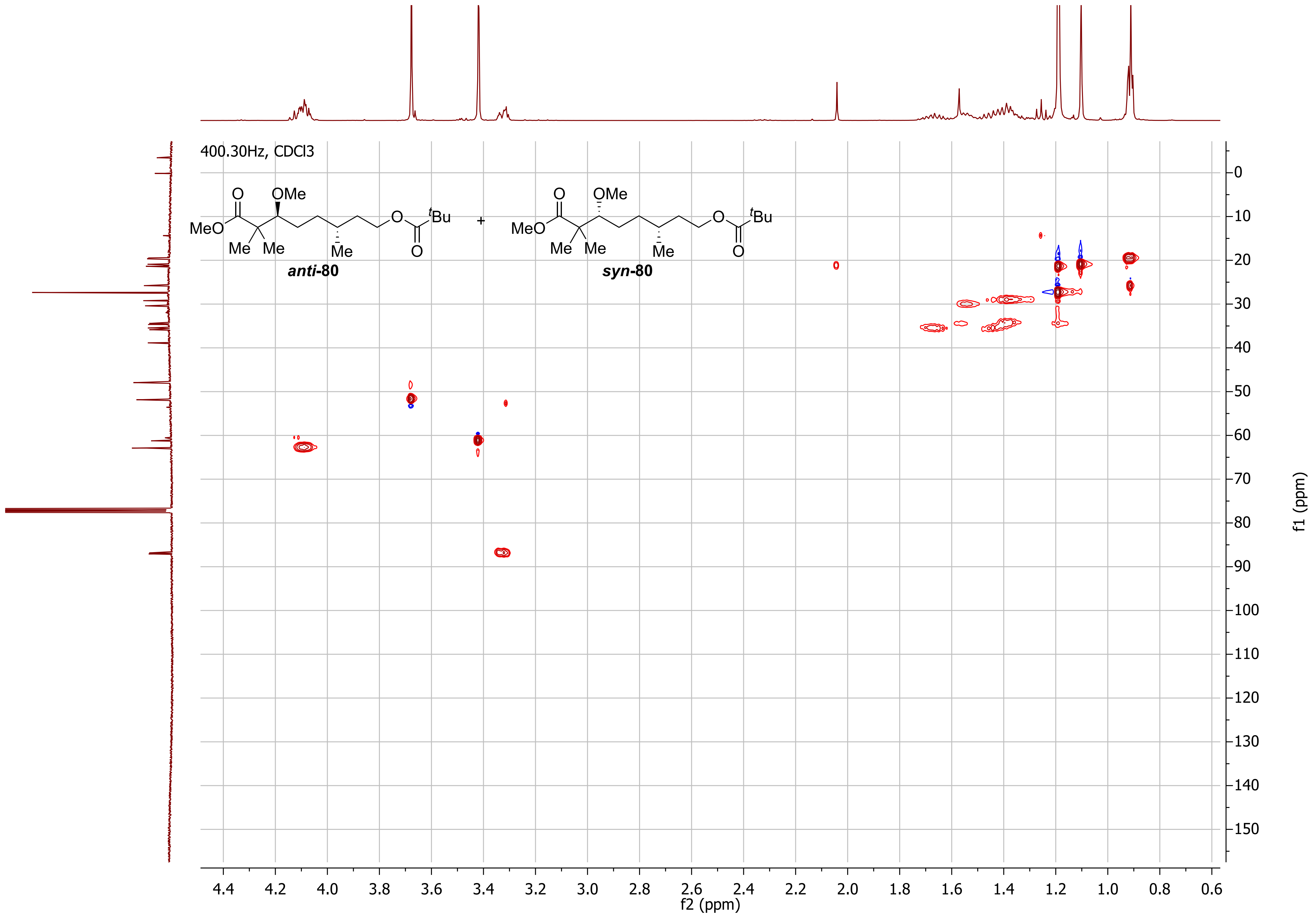

400.30Hz, CDCl3
MeO
O
OMe
Me
Me
Me
O
tBu
O
anti-80
+
syn-80
f2 (ppm)
f1 (ppm)

400.30Hz, CDCl3

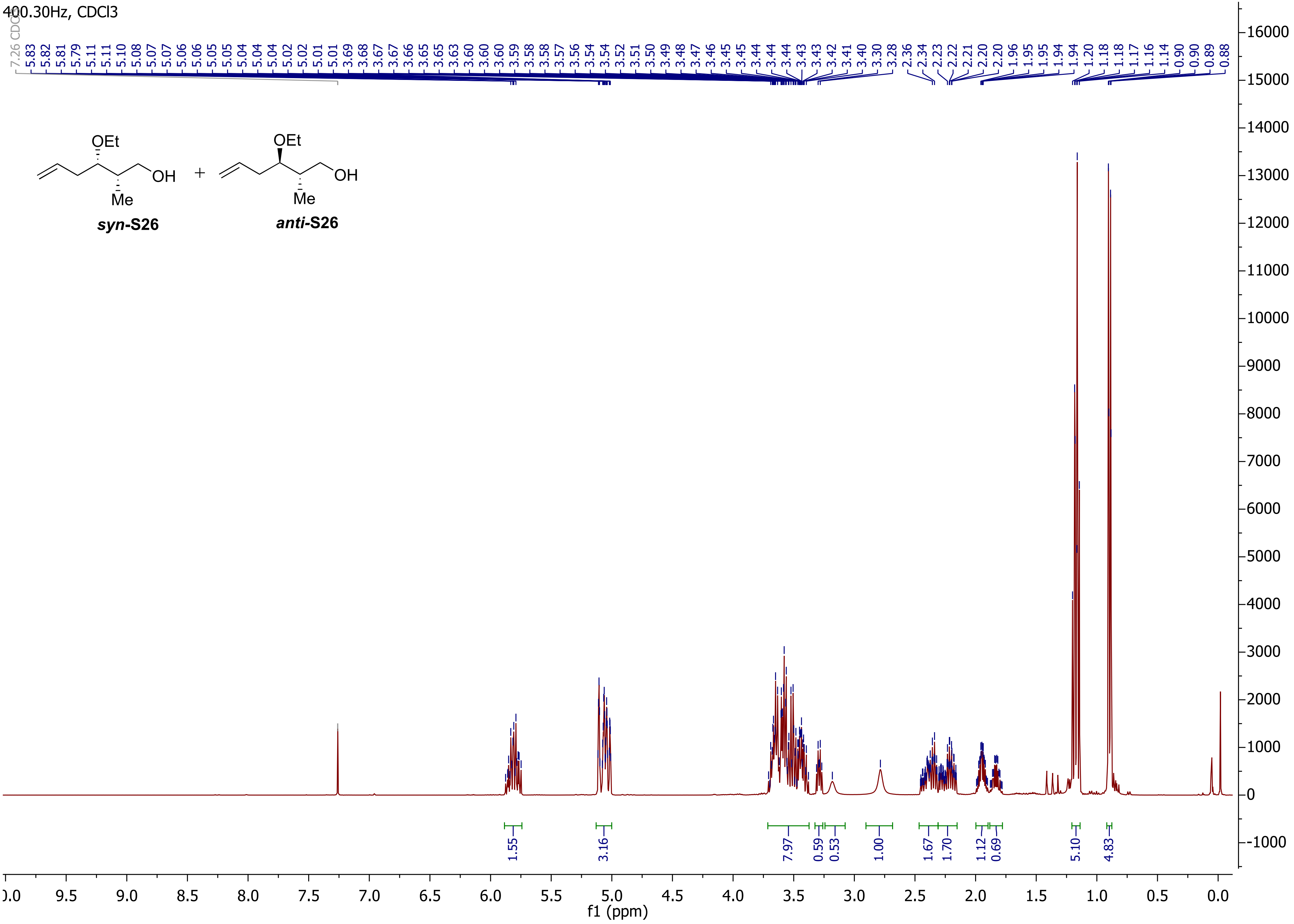

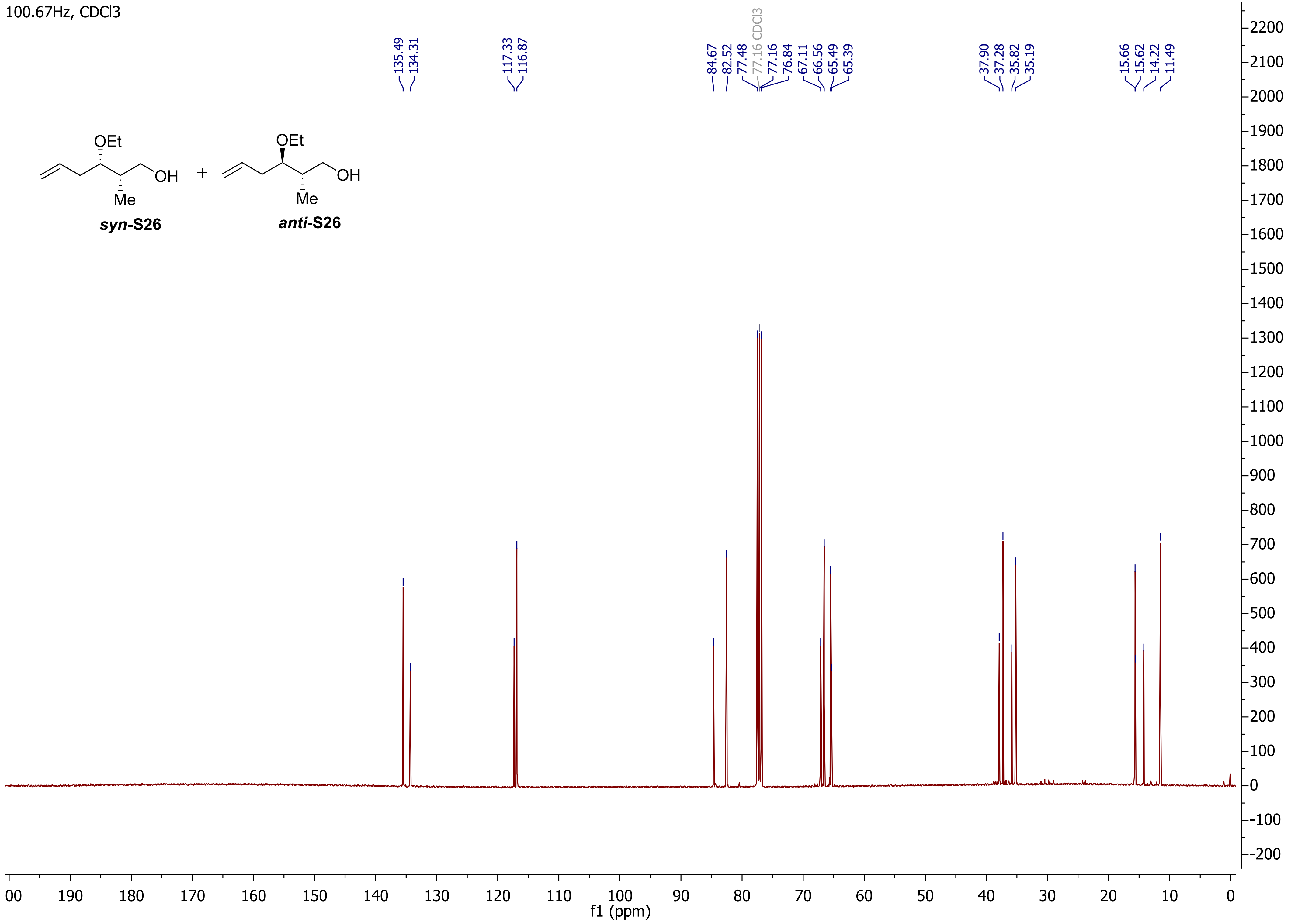
100.67Hz, CDCl3
135.49
134.31
117.33
116.87
84.67
82.52
77.48
77.16 CDCl3
77.16
76.84
67.11
66.56
65.49
65.39
37.90
37.28
35.82
35.19
15.66
15.62
14.22
11.49
OEt
OH
Me
+
syn-S26
anti-S26
f1 (ppm)

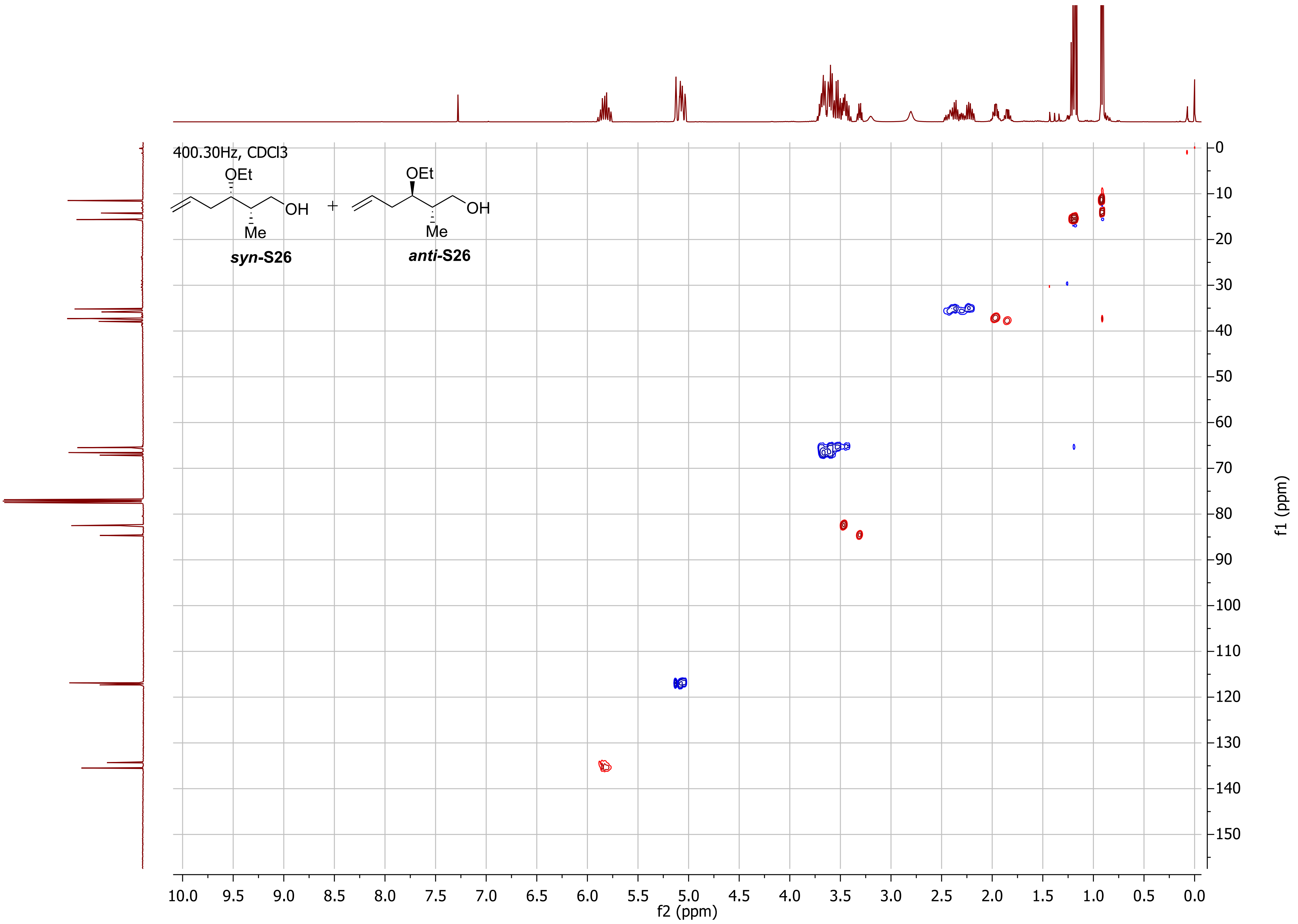
400.30Hz, CDCl3
OEt
OH
Me
syn-S26
+
anti-S26
f2 (ppm)
f1 (ppm)

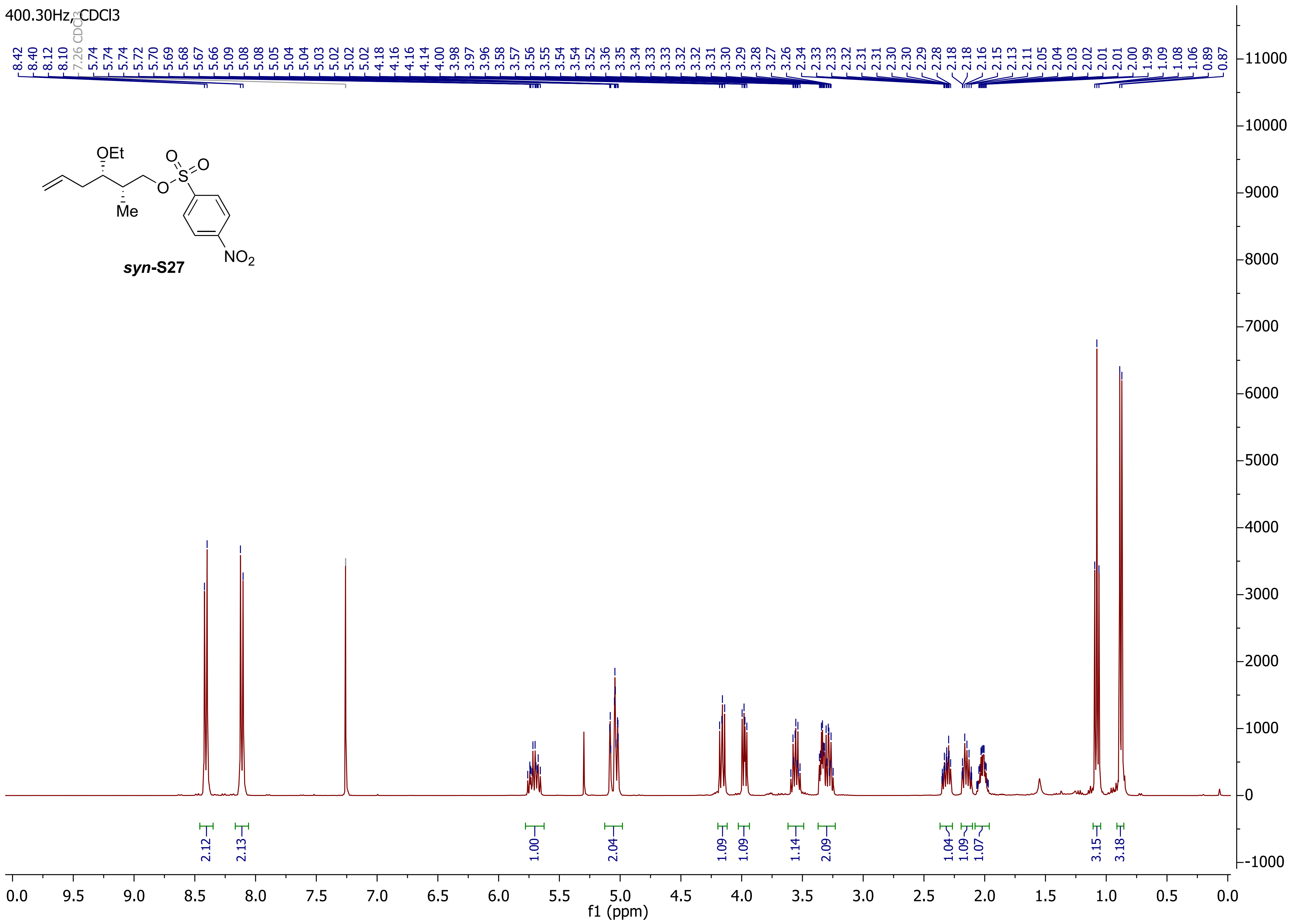
400.30Hz, CDCl3
8.42 8.40 8.12 8.10 7.26 CDCl3 5.74 5.74 5.74 5.72 5.70 5.69 5.68 5.67 5.66 5.09 5.08 5.08 5.05 5.04 5.04 5.03 5.02 5.02 5.02 4.18 4.16 4.16 4.14 4.00 3.98 3.97 3.96 3.58 3.57 3.56 3.55 3.54 3.54 3.52 3.36 3.35 3.34 3.33 3.33 3.32 3.32 3.31 3.30 3.29 3.28 3.27 3.26 2.34 2.33 2.33 2.32 2.31 2.31 2.30 2.30 2.29 2.28 2.18 2.18 2.16 2.15 2.13 2.11 2.05 2.04 2.03 2.02 2.01 2.01 2.00 1.99 1.09 1.08 1.06 0.89 0.87
OEt
O
S
O
O
Me
NO2
syn-S27
2.12
2.13
1.00
2.04
1.09
1.09
1.14
2.09
1.04
1.09
1.07
3.15
3.18
f1 (ppm)

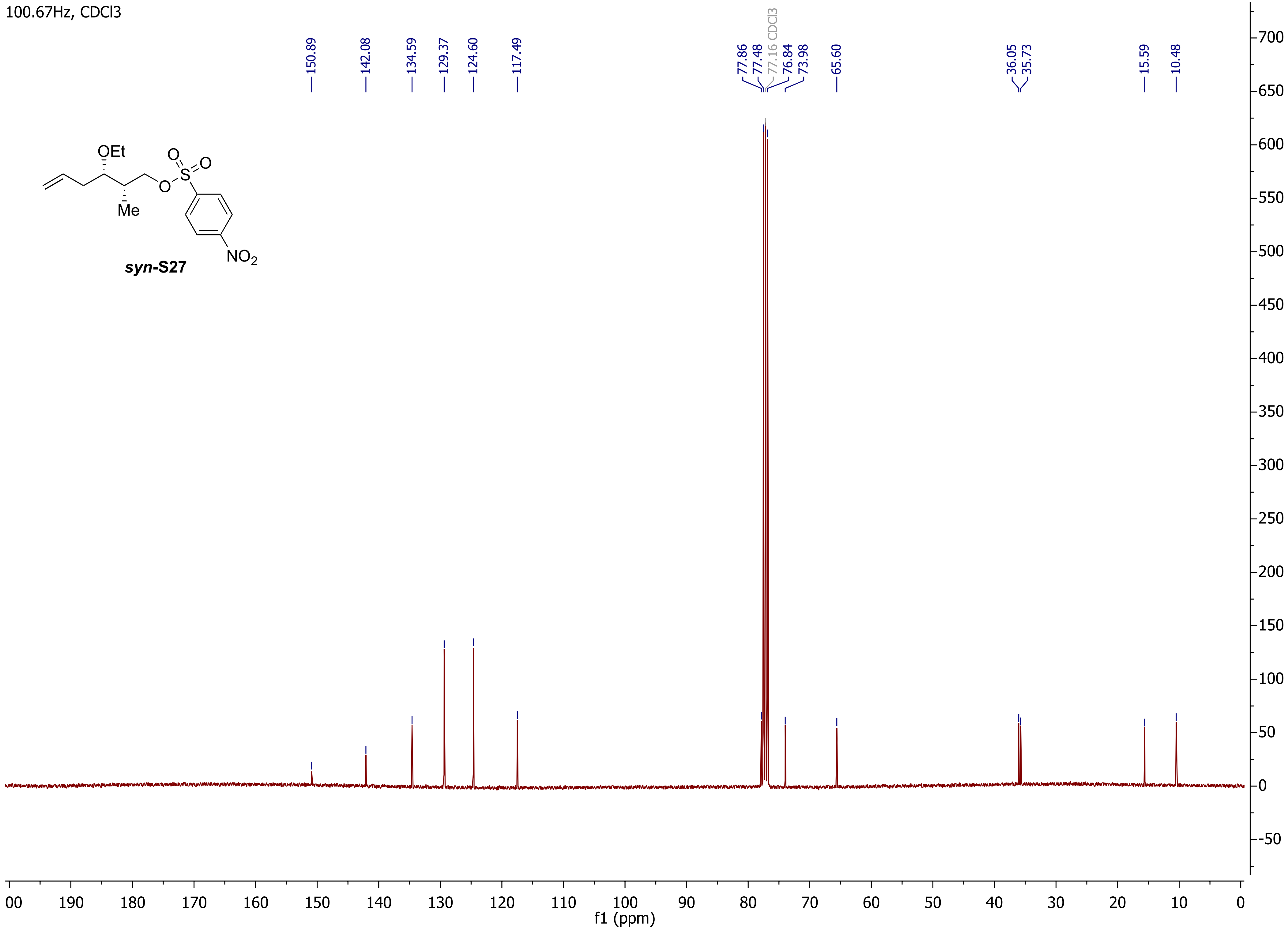
100.67Hz, CDCl3
150.89
142.08
134.59
129.37
124.60
117.49
77.86
77.48
77.16 CDCl3
76.84
73.98
65.60
36.05
35.73
15.59
10.48
OEt
Me
NO2
syn-S27
f1 (ppm)

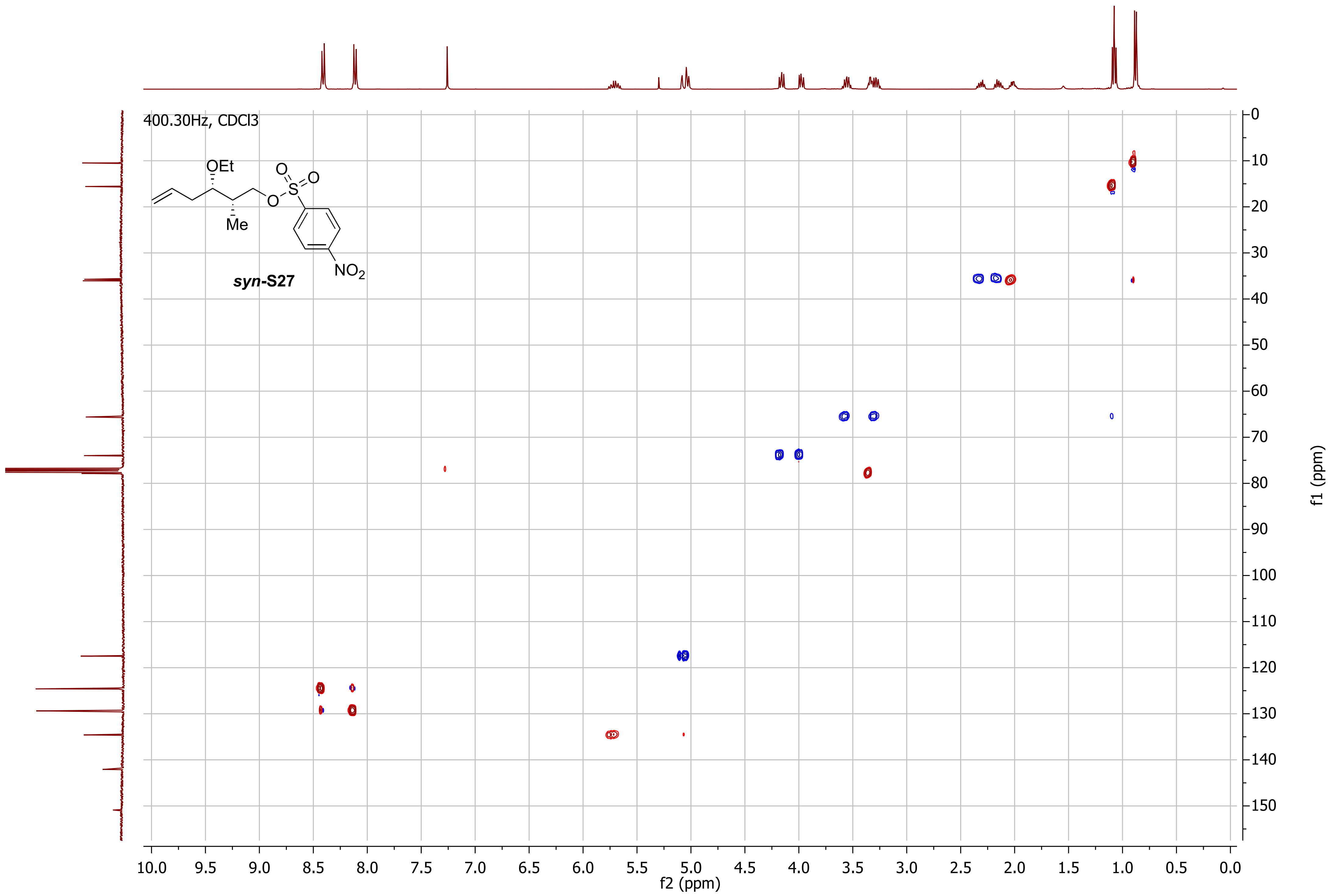
400.30Hz, CDCl3
OEt
Me
syn-S27
NO2
f2 (ppm)
f1 (ppm)

400.30Hz, CDCl3

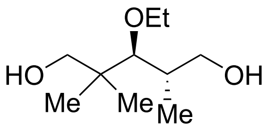

anti-S28

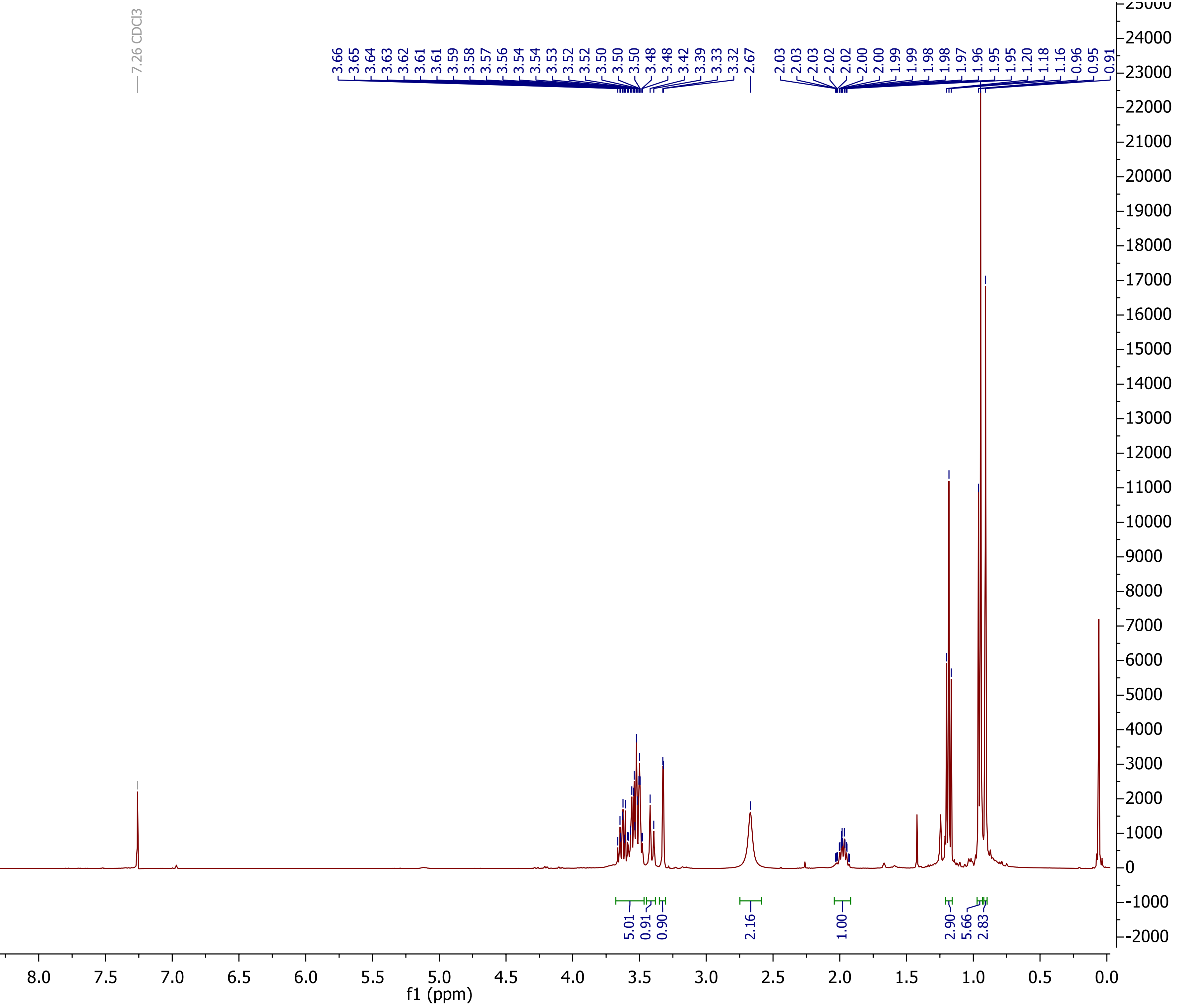

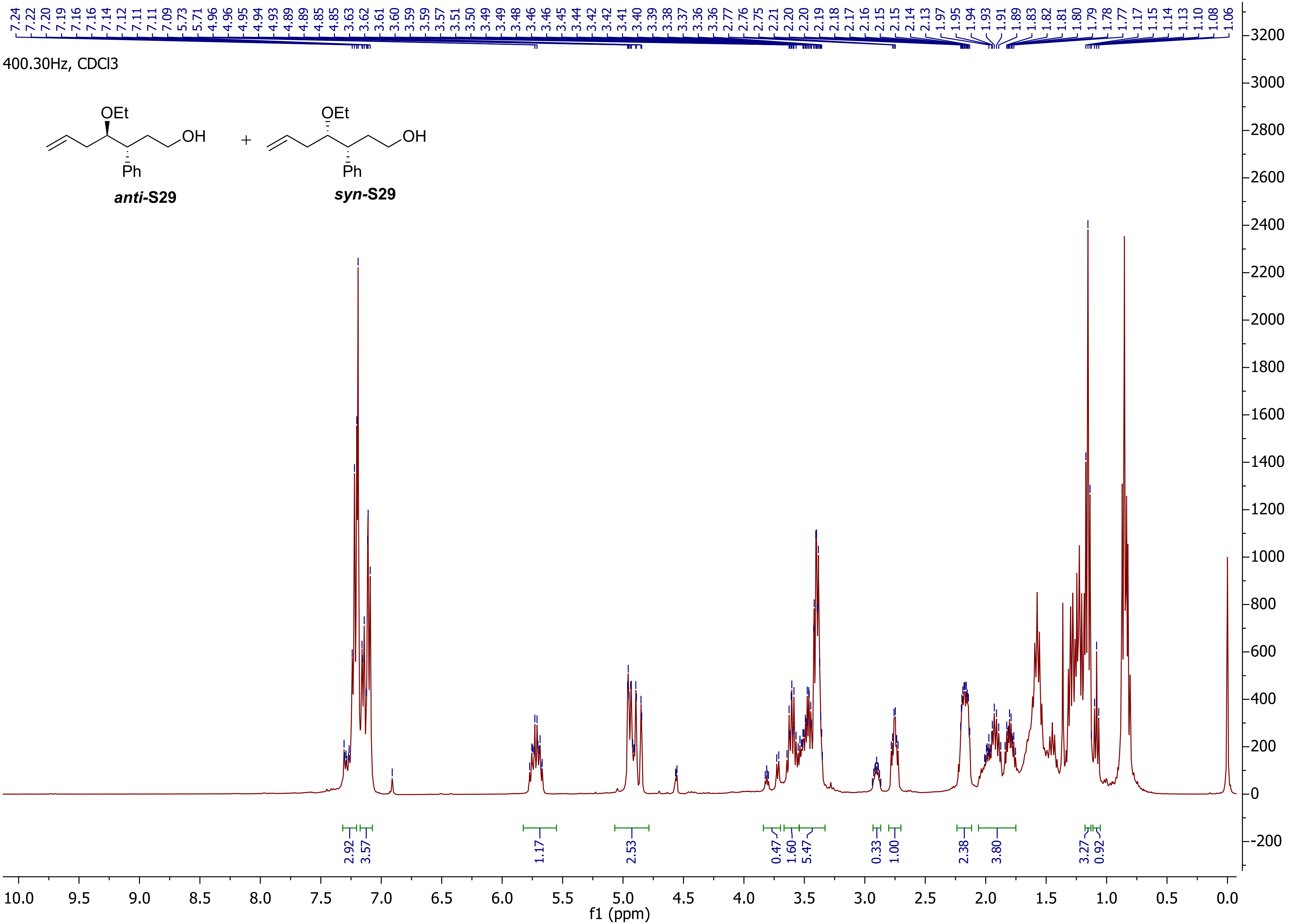

***anti*-S29**

***syn*-S29**

100.67Hz, CDCl3

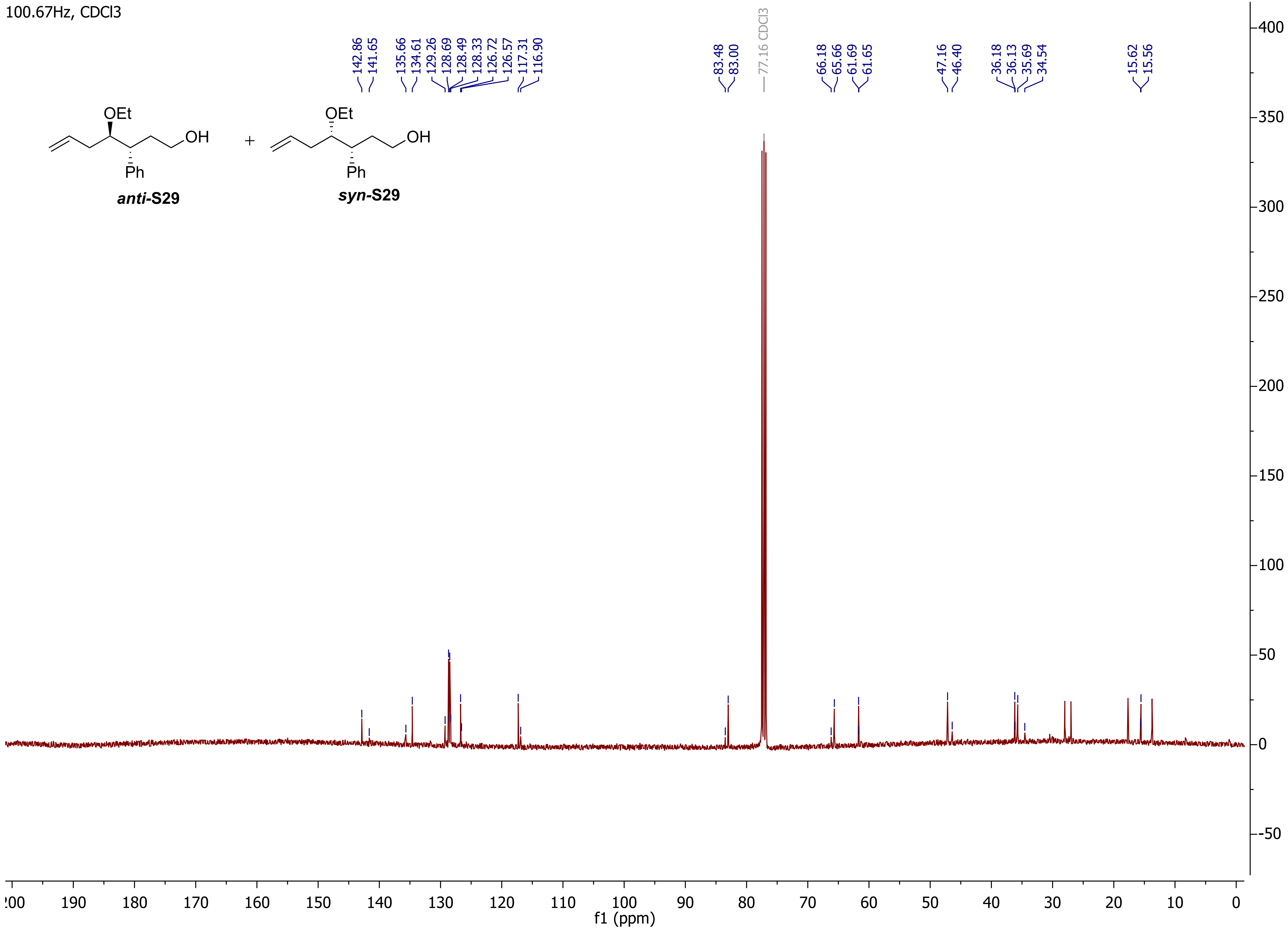

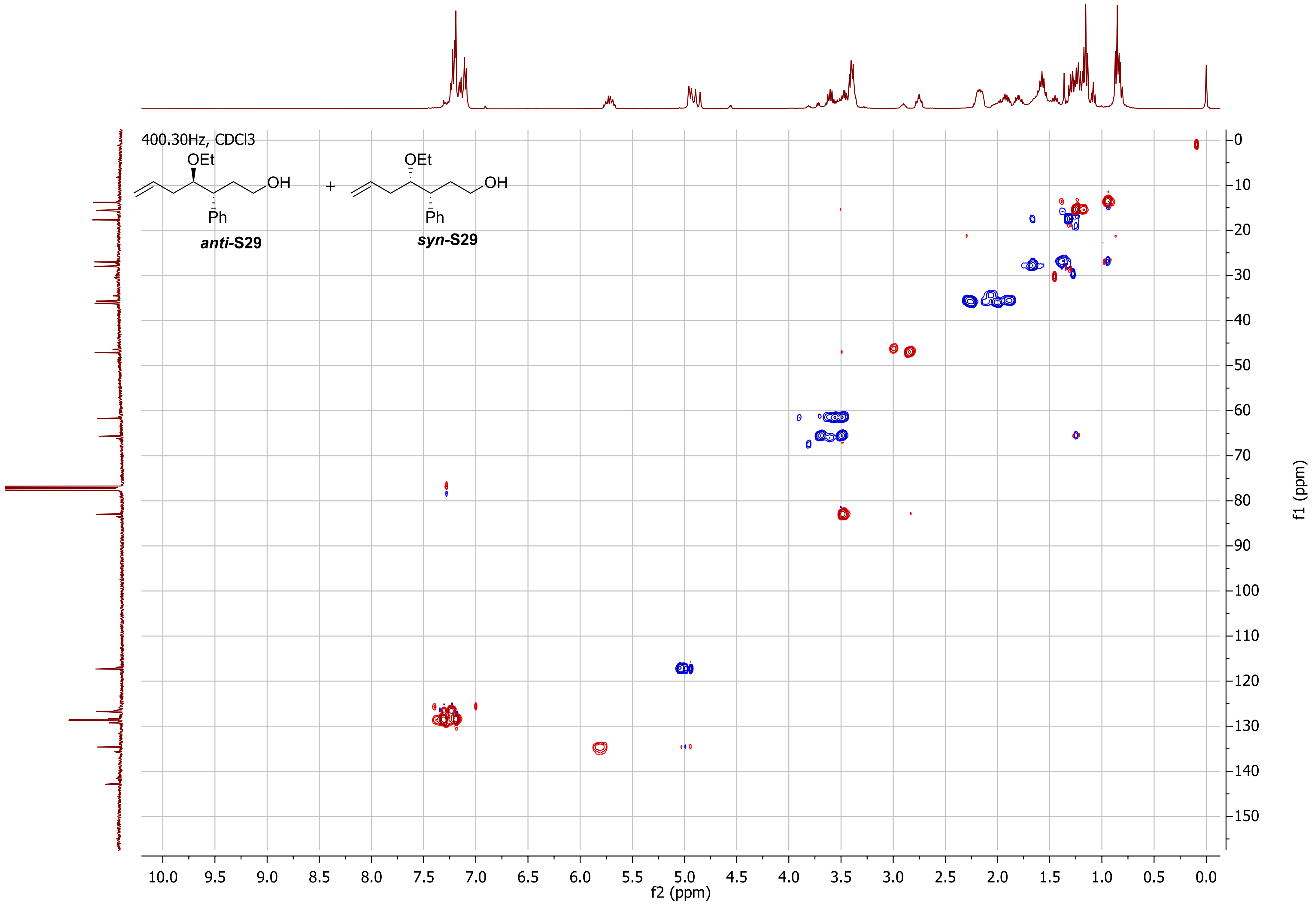
400.30Hz, CDCl3
OEt
OH
Ph
anti-S29
+
OEt
OH
Ph
syn-S29
f2 (ppm)
f1 (ppm)

400.30Hz, CDCl3

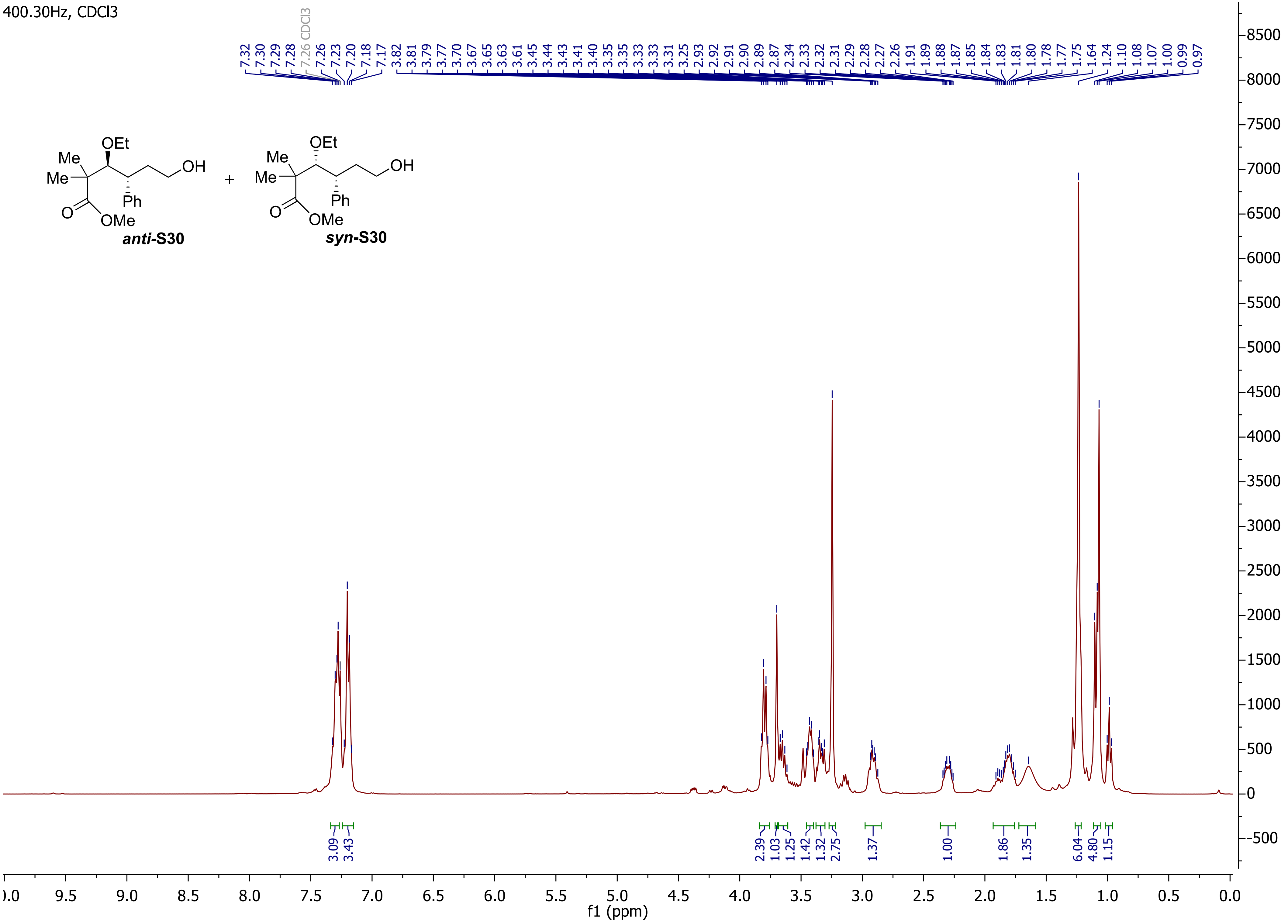

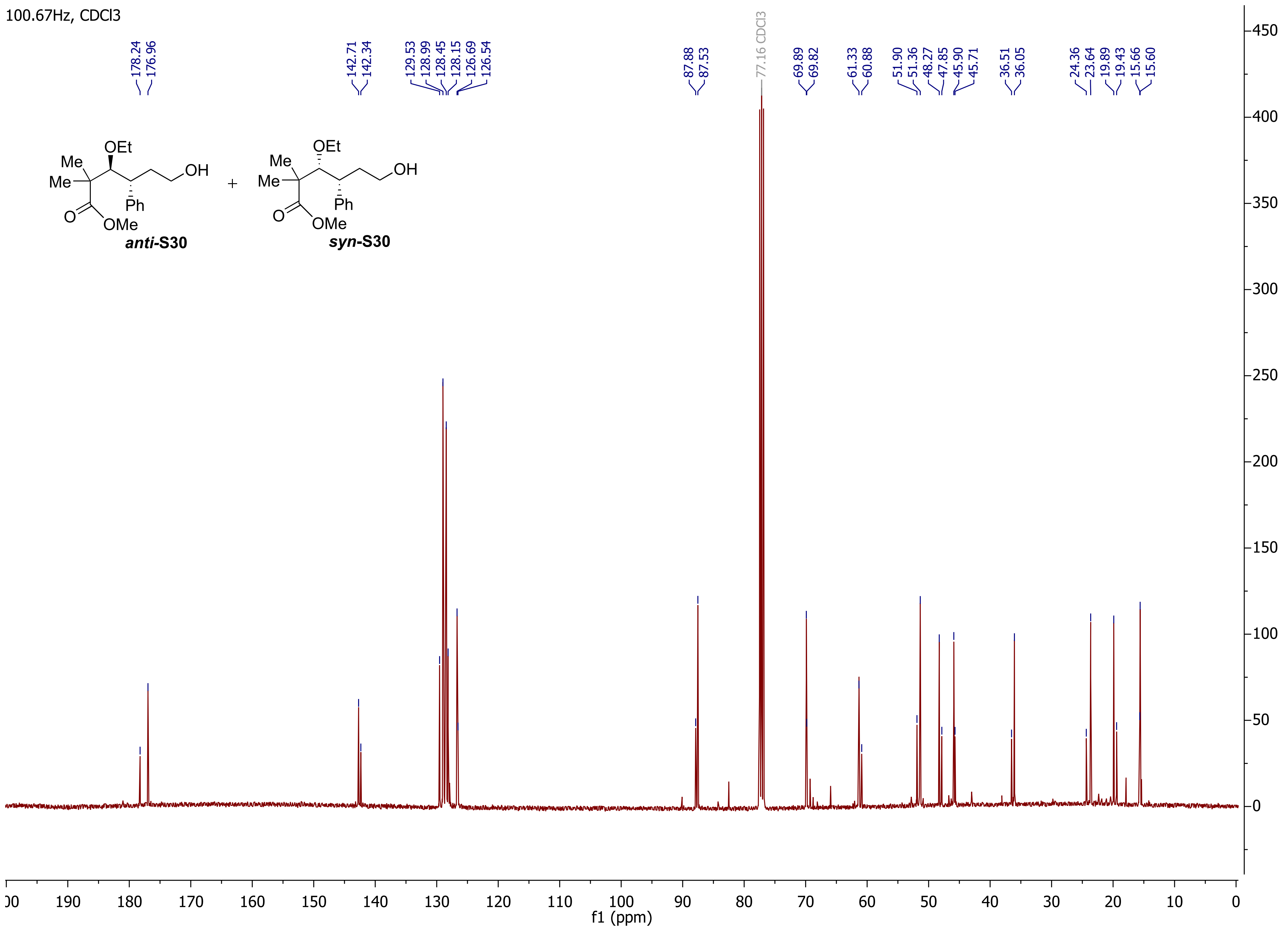
100.67Hz, CDCl3
178.24
176.96
142.71
142.34
129.53
128.99
128.45
128.15
126.69
126.54
87.88
87.53
77.16 CDCl3
69.89
69.82
61.33
60.88
51.90
51.36
48.27
47.85
45.90
45.71
36.51
36.05
24.36
23.64
19.89
19.43
15.66
15.60
OEt
Me
Me
OH
Ph
O
OMe
anti-S30
+
syn-S30
f1 (ppm)

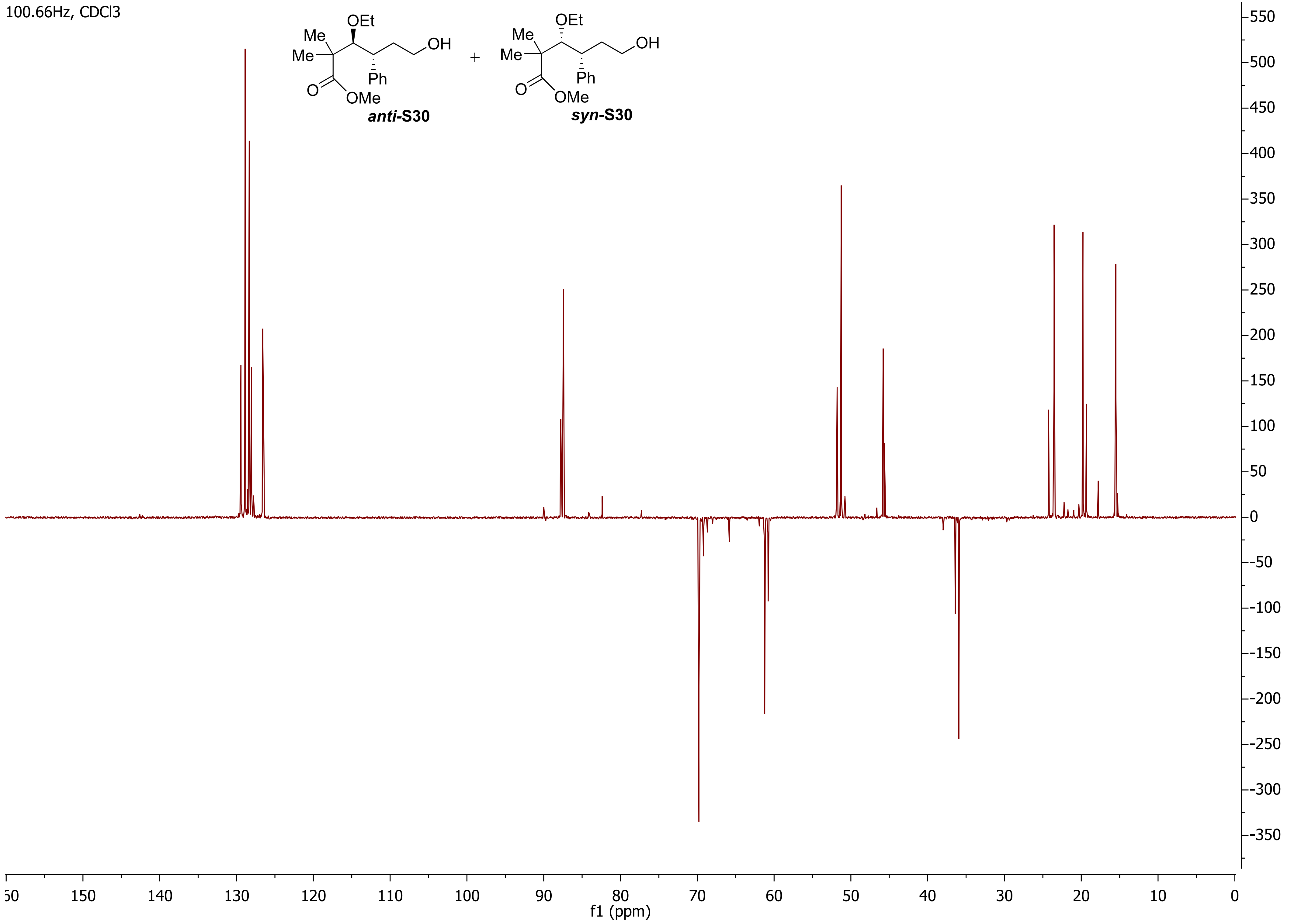
100.66Hz, CDCl3
OEt
Me
Me
OH
Ph
O
OMe
+
anti-S30
syn-S30
f1 (ppm)

400.30Hz, CDCl3

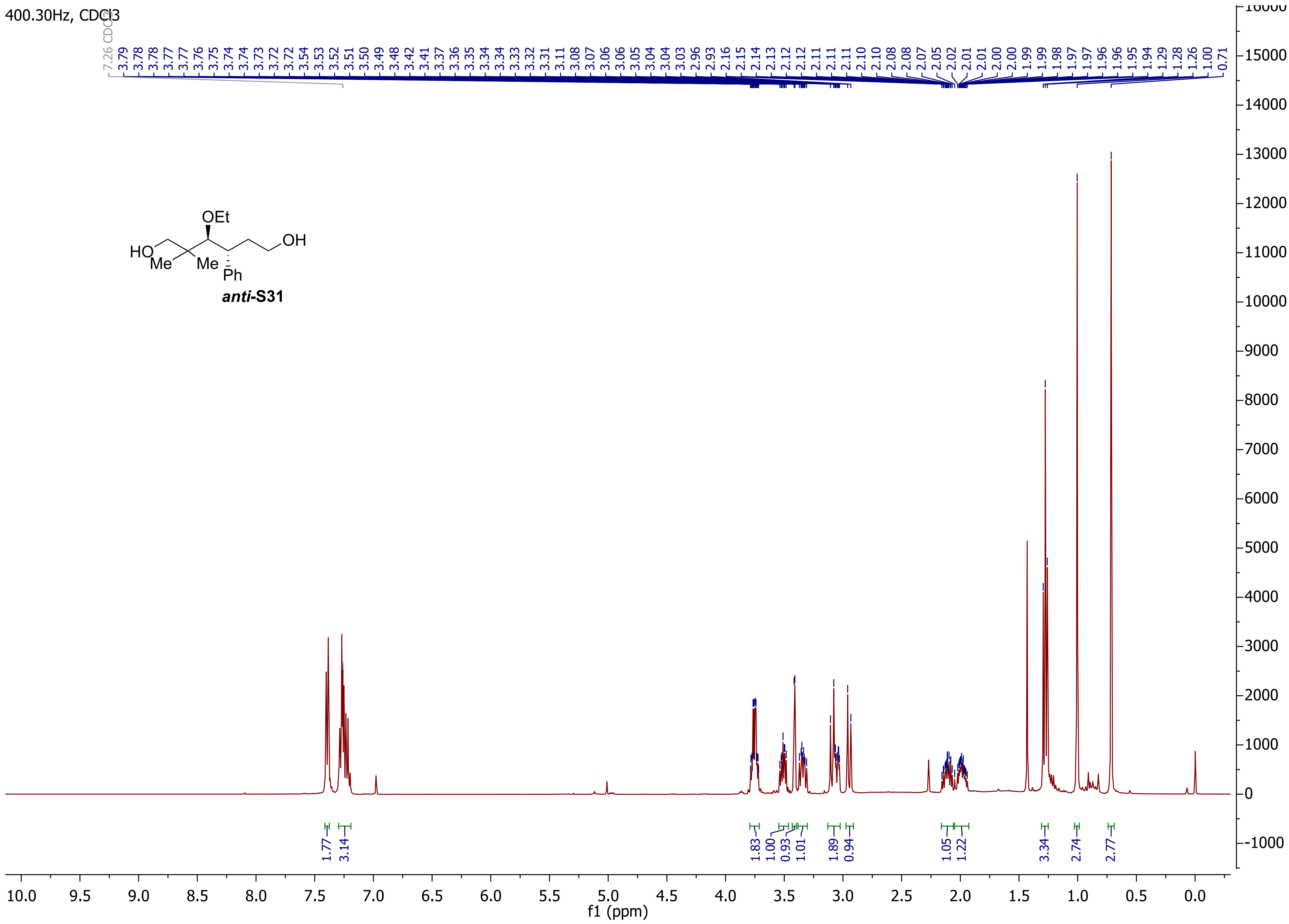

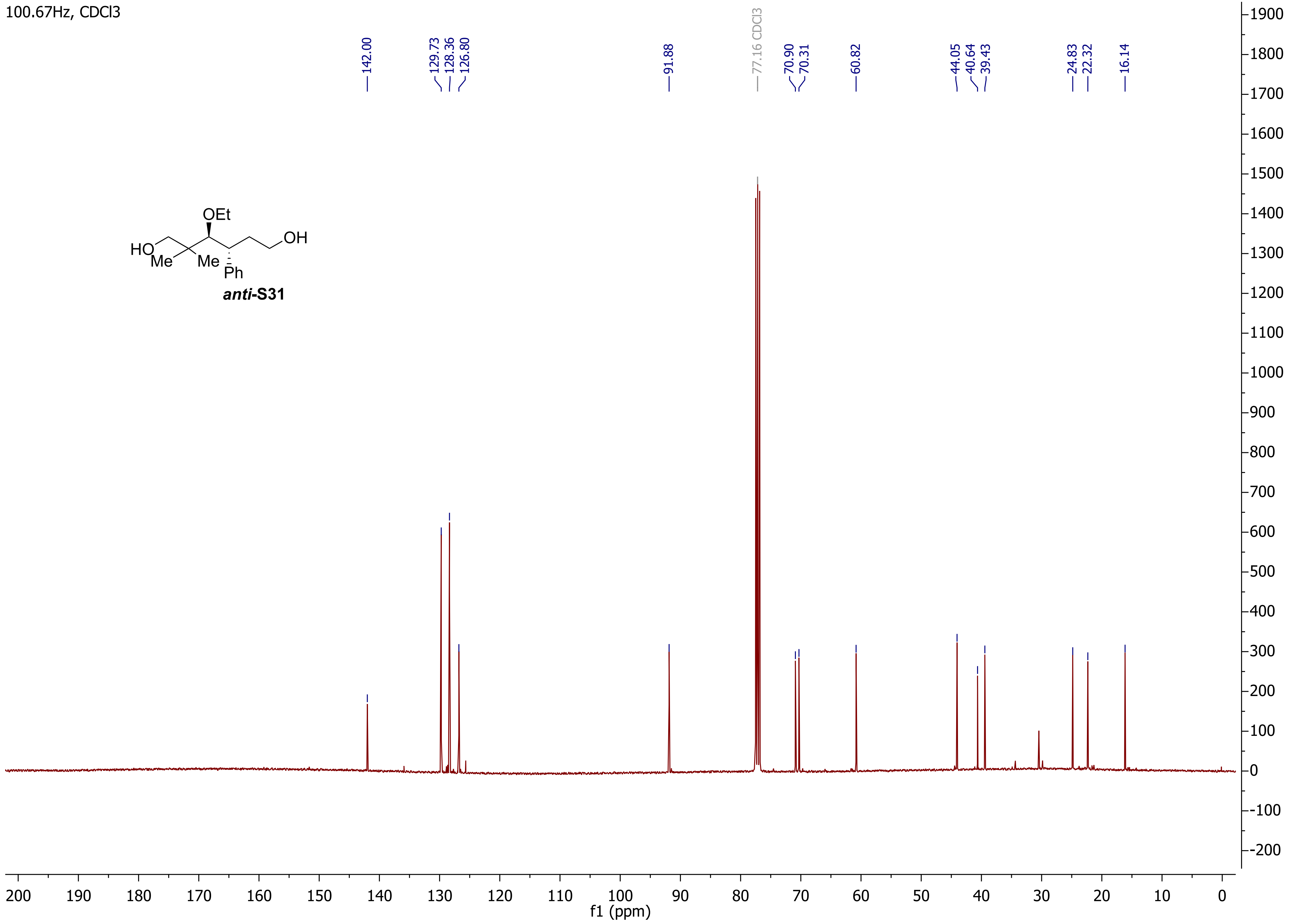
100.67Hz, CDCl3
142.00
129.73
128.36
126.80
91.88
77.16 CDCl3
70.90
70.31
60.82
44.05
40.64
39.43
24.83
22.32
16.14
OEt
HO
OH
Me
Me
Ph
anti-S31
f1 (ppm)
200 190 180 170 160 150 140 130 120 110 100 90 80 70 60 50 40 30 20 10 0
1900 1800 1700 1600 1500 1400 1300 1200 1100 1000 900 800 700 600 500 400 300 200 100 0 -100 -200

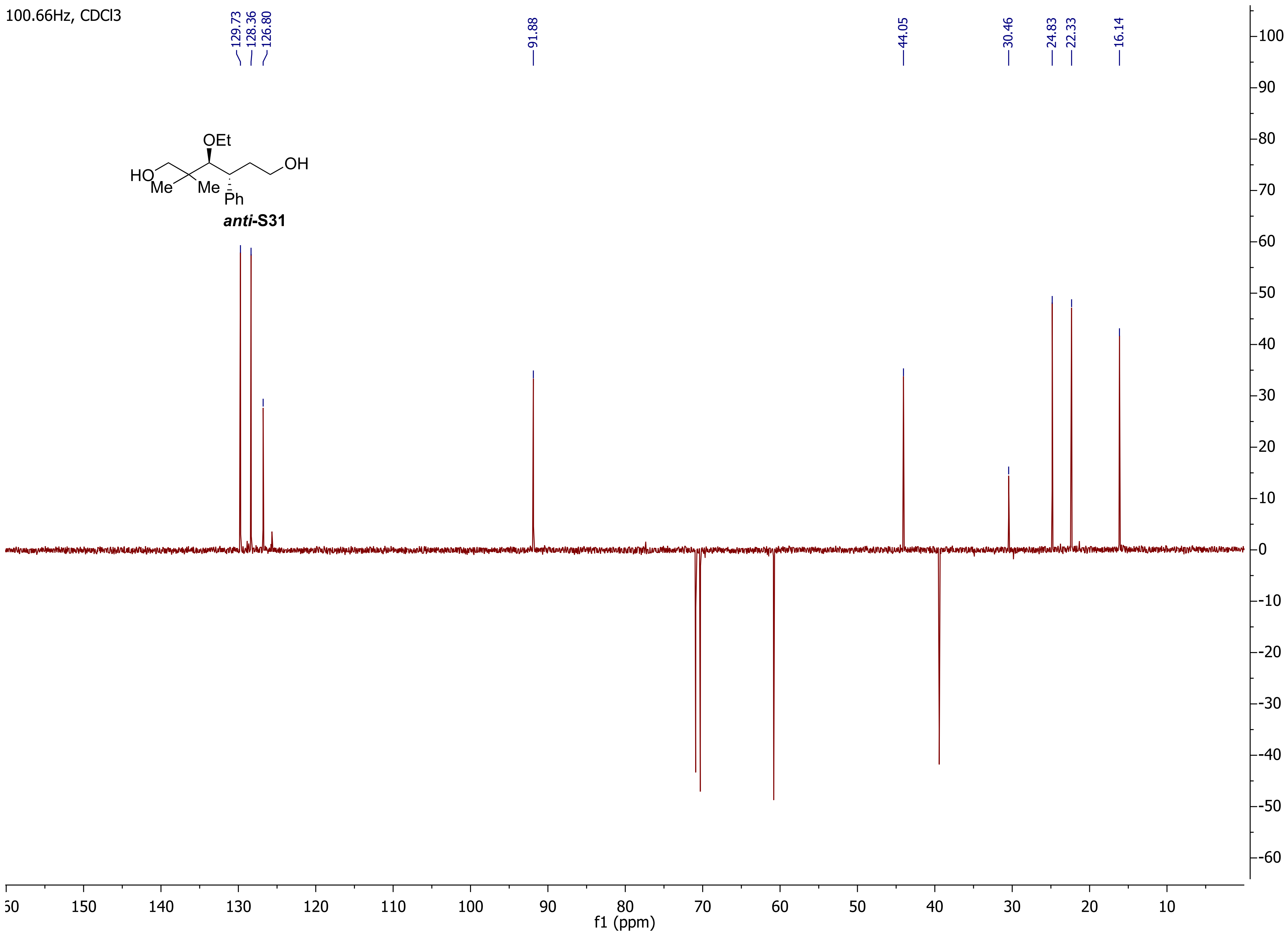
100.66Hz, CDCl3
129.73
128.36
126.80
91.88
44.05
30.46
24.83
22.33
16.14
OEt
HO
OH
Me
Me
Ph
anti-S31
f1 (ppm)

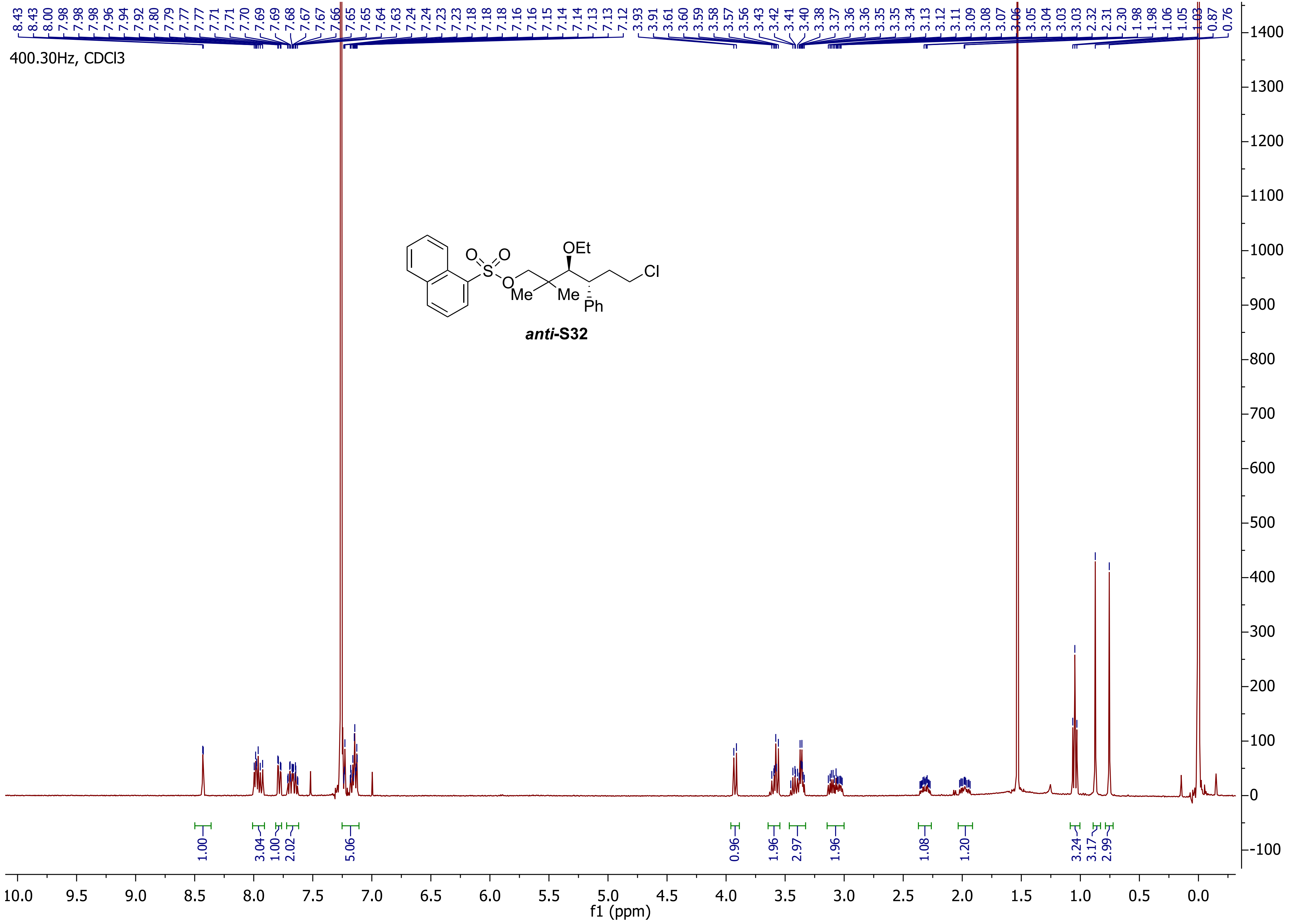
400.30Hz, CDCl3
OEt
Cl
Me
Me
Ph
O
O
S
O
anti-S32
f1 (ppm)

100.67Hz, CDCl3

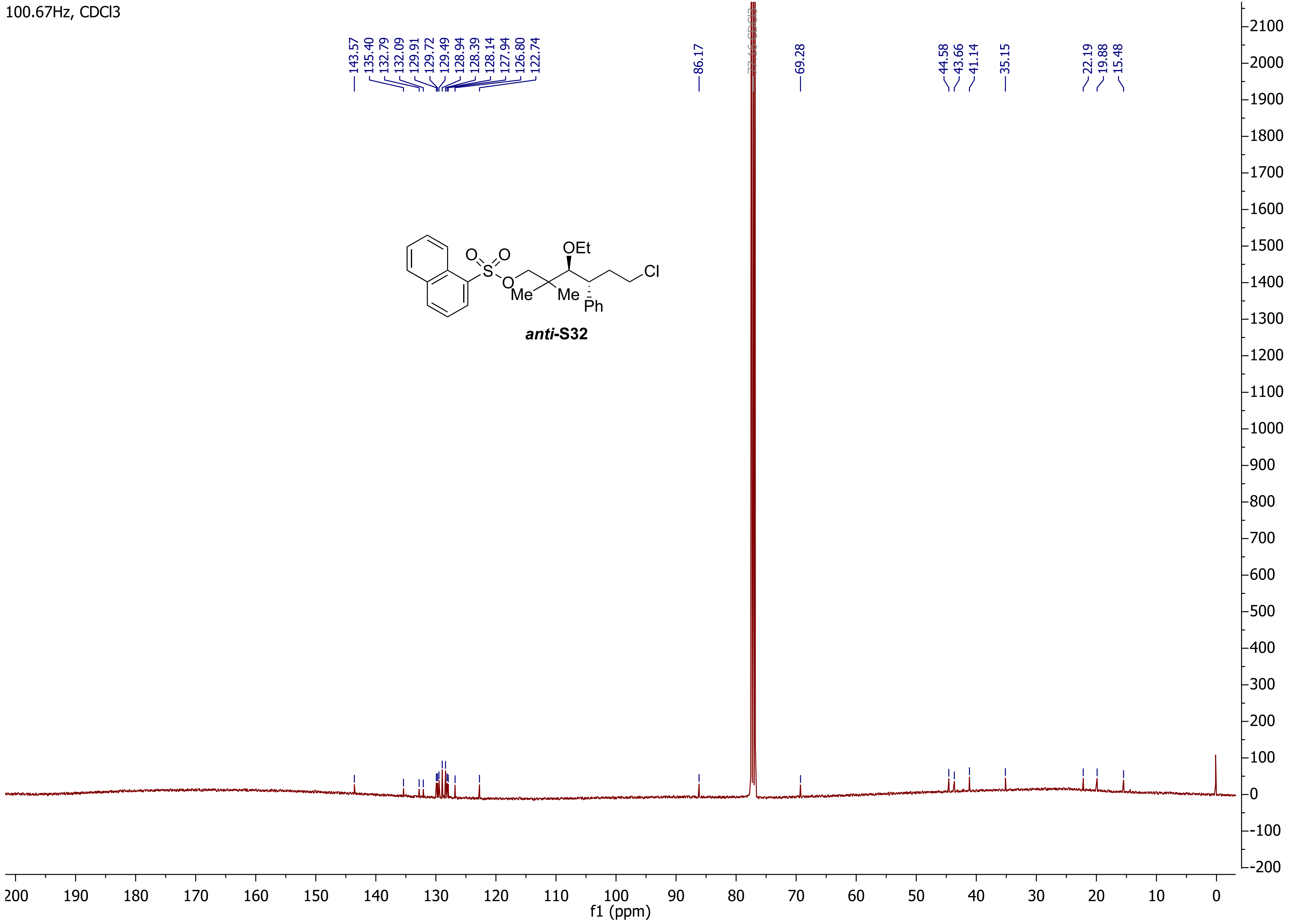

***anti*-S32**

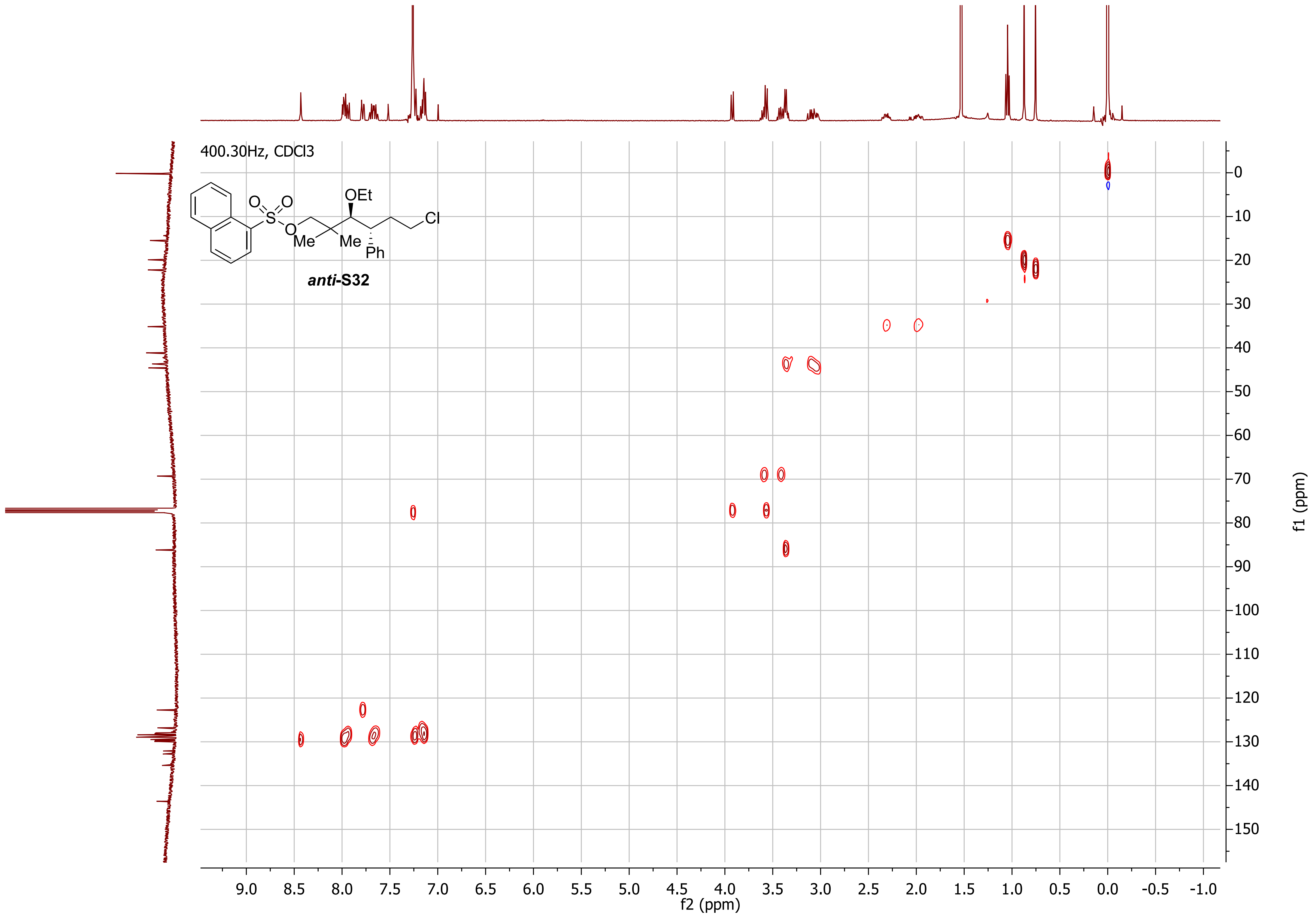

400.30Hz, CDCl3
anti-S32
f2 (ppm)
f1 (ppm)

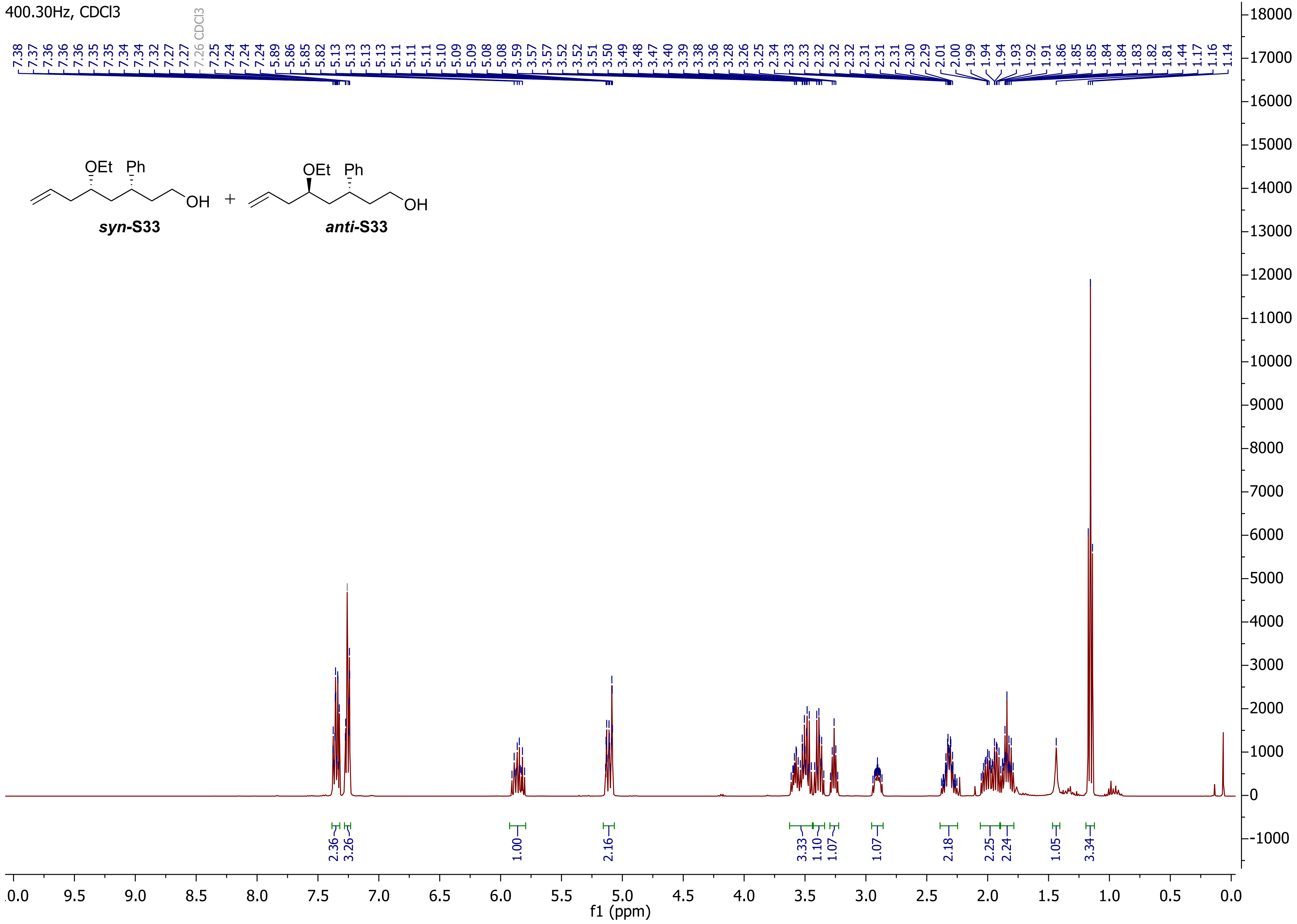

400.30Hz, CDCl3
OEt Ph
OH
+
syn-S33
anti-S33
f1 (ppm)

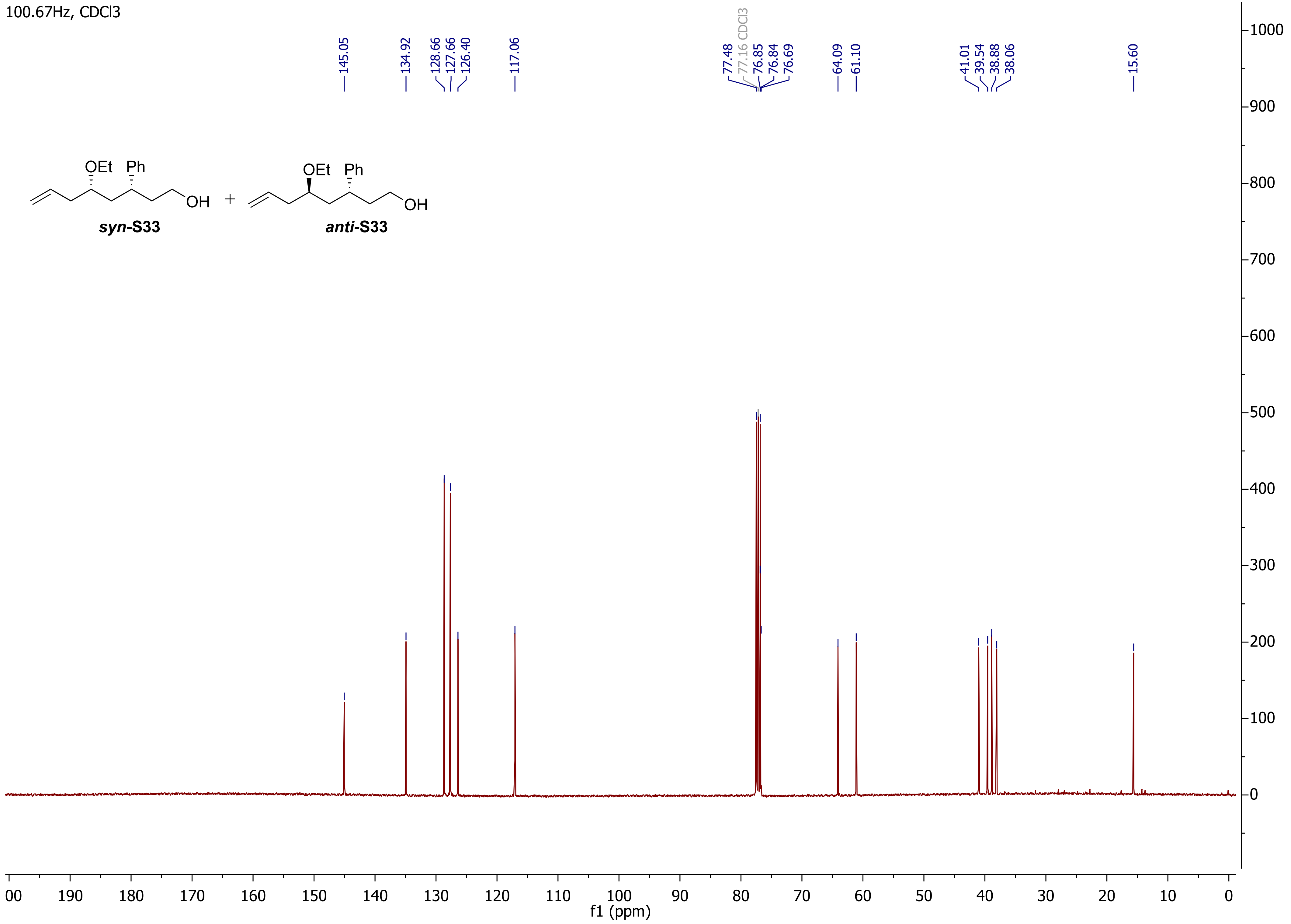
100.67Hz, CDCl3
145.05
134.92
128.66
127.66
126.40
117.06
77.48
77.16 CDCl3
76.85
76.84
76.69
64.09
61.10
41.01
39.54
38.88
38.06
15.60
OEt
Ph
OH
+
syn-S33
anti-S33
f1 (ppm)

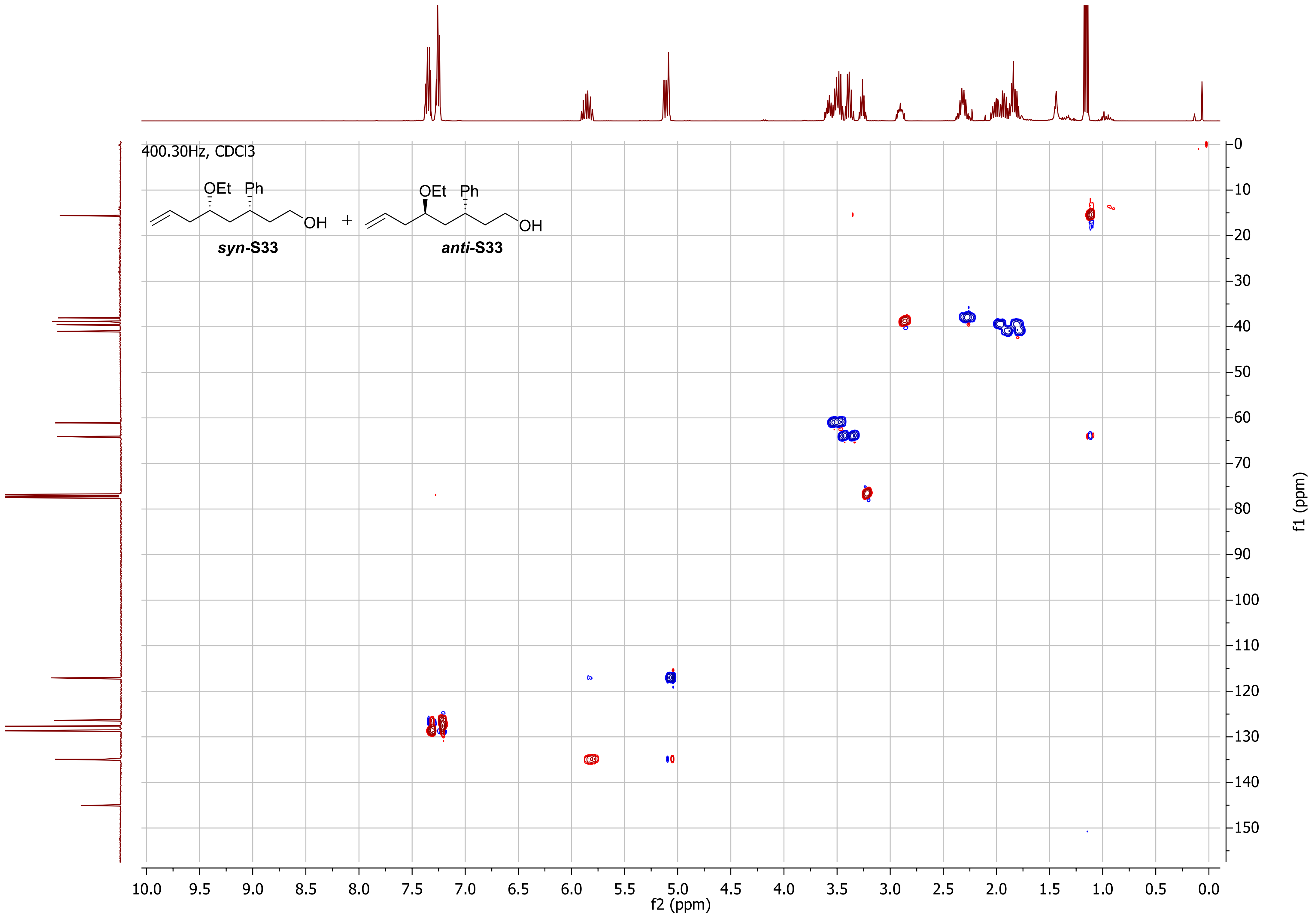

400.30Hz, CDCl3
OEt Ph
OH
+
syn-S33
anti-S33
f2 (ppm)
f1 (ppm)
